# Supplementary material for: Photoredox-catalyzed C–C bond cleavage of cyclopropanes for the formation of C(sp3)–heteroatom bonds
Source: Nat Commun. 2022 Oct 8;13:5938. doi: 10.1038/s41467-022-33602-4 (PMC9547854; doi:10.1038/s41467-022-33602-4)
Supplement: Supplementary file 1 — Supplementary Information [file 41467_2022_33602_MOESM1_ESM.pdf]

Supplementary Information

Photoredox-catalyzed C–C bond cleavage of aryl cyclopropanes for the formation of C(sp^3)–heteroatom bonds

Liang Ge, Chi Zhang, Chengkai Pan, Ding-Xing Wang, Dong-Ying Liu, Zhi-Qiang Li, Pingkang Shen, Lifang Tian & Chao Feng*

Technical Institute of Fluorochemistry, Institute of Advanced Synthesis, School of Chemistry and Molecular Engineering, State Key Laboratory of Material-Oriented Chemical Engineering, Nanjing Tech University, Nanjing, Jiangsu 211816, P. R. China

iamcfeng@njtech.edu.cn

Table of Contents

Supplementary Methods 2
General Information 2
Substrate Preparation 7
Reaction Condition Optimization 32
General Procedure 41
Supplementary Discussion 48
Light On/Off Experiment 48
Determination of the Reaction Quantum Yield (Φ) 48
Radical Inhibition Experiment with TEMPO 50
Radical Clock Experiment 51
Deuterium Isotope Labeling Studies 51
Reaction with Enantioenriched Substrate **114** 53
Kinetic Study 67
^{1}H NMR and ^{13}C NMR Spectrum of Structurally Novel Compounds 69
Characterization of Structurally Novel Compounds 182
HPLC Spectra 210
DFT Study 214
Supplementary References 221

Supplementary Methods

General Information

Unless otherwise noted, all reactions were carried out under nitrogen atmosphere. All commercially available reagents were used directly without further purification unless noted. All solvents were dried by passing through a column of neutral alumina under nitrogen prior to use. Organic solvents were concentrated under reduced pressure on an IKA RV 10 rotary evaporator. Chromatography was performed using silica gel with distilled solvents. Thin-layer chromatography (TLC) was performed on Silicycle 250 μm silica gel plates visualized under UV light (254 nm) and dyed with cerous molybdate solution by heating.

HRMS spectra were recorded on a Xevo G2-XS QTof (Waters Corporation). ^{1}H NMR and ^{13}C NMR spectra were recorded using Bruker Avance 400 MHz spectrometers. Chemical shifts for ^{1}H NMR spectra are reported as δ in units of parts per million (ppm) downfield from $SiMe_4$ (δ 0.0) and relative to the signal of chloroform-*d* (δ 7.26, singlet). Multiplicities were given as: s (singlet); d (doublet); t (triplet); q (quartet); dd (doublet of doublets); dt (doublet of triplets); m (multiplet), etc. Coupling constants are reported as a *J* value in Hz. Carbon nuclear magnetic resonance spectra (^{13}C NMR) are reported as δ in units of parts per million (ppm) downfield from $SiMe_4$ (δ 0.0) and relative to the signal of chloroform-d (δ 77.00, triplet).

The photocatalyst $[Ir(dF(CF_3)ppy)_2(4,4'\text{-}dtbbpy)]PF_6$ (PC-I)[1], $[Ir(dF(CF_3)ppy)_2bpy]PF_6$ (PC-II)[1], $[Ir(dF(CF_3)ppy)_2(4,4'\text{-}dCF_3bpy)]PF_6$ (PC-III)[1], $Ir(dF(CF_3)ppy)_2(5,5'\text{-}dCF_3bpy)]PF_6$ (PC-IV)[1] and PC-VIII[2] were prepared following literature procedures. Acr-Mes-$Me^+ClO_4^-$ (PC-V), Acr-Mes-$Me^+BF_4^-$ (PC-VI), PC-VII and 4CzIPN[3] (PC-IX) were purchased from Energy.

Preparation of PC-I to PC-IV

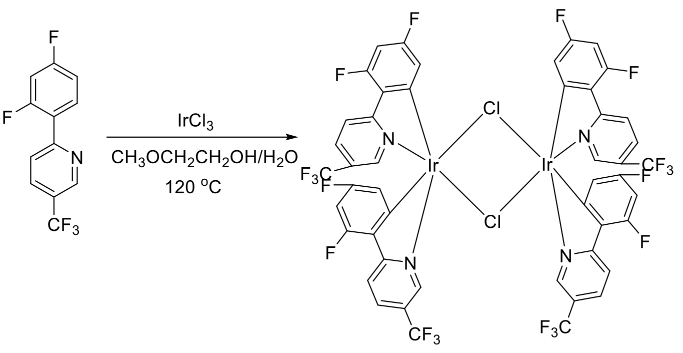

[(dFCF3ppy)2-Ir-μ-Cl)]2

IrCl3 (0.448 g, 1.5 mmol) and 2-(2,4-difluorophenyl)-5-(trifluoromethyl)pyridine (dFCF3ppy) (0.880 g, 3.4 mmol) were added to a mixture of 2-methoxyethanol (20 mL) and water (10 mL). The mixture was refluxed at 120 ºC for 12 h under N_2 atmosphere. After cooling to room temperature, an amorphous yellowish green precipitate was obtained, which was further filtered, washed, and recrystallized in methanol/acetone to yield bis-(μ)-chlorotetrakis(2-(4,6-difluoromethylphenyl)-pyridinato-C,N)diiridium(III) [(dFCF3ppy)2-Ir-μ-Cl]2.

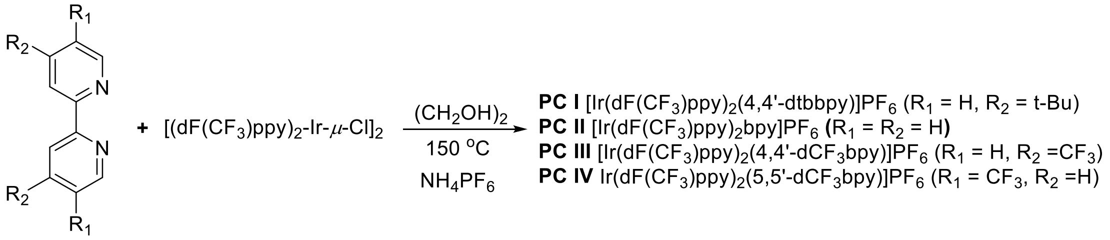

[(dFCF3ppy)2-Ir-μ-Cl]2 (0.263 g, 0.18 mmol) and corresponding dipyridine (0.40 mmol) were refluxed at 150 ºC for 15 h in ethylene glycol (18 mL) under N_2 atmosphere. After cooling to room temperature, the mixture was transferred to a separatory funnel with water (120 mL) and washed with hexane for three times (60 mL each time). The aqueous layer was then heated to 85 °C to remove residual hexane. Subsequently, 20 mL of aqueous solution of ammonium hexafluorophosphate solution (NH_4PF_6, 2 g) was added to above mixture, producing a yellow-green amorphous precipitate. The precipitate was filtered, dried, and recrystallized to yield the iridium-photocatalyst.

[Ir(dF(CF3)ppy)2(4,4'-dtbbpy)]PF6 (PC-I)

^{1}H NMR (400 MHz, *Acetone-d6*): δ 8.94 (d, *J* = 1.8 Hz, 2H), 8.62 (dd, *J* = 8.8, 2.5 Hz, 2H), 8.41 (dd, *J* = 8.8, 1.8 Hz, 2H), 8.19 (d, *J* = 5.9 Hz, 2H), 7.90 – 7.71 (m, 4H), 6.91 – 6.84 (m, 2H), 5.98 (dd, *J* = 8.4, 2.3 Hz, 2H), 1.44 (s, 18H) ppm.

[Ir(dF(CF$_3$)ppy)$_2$bpy]PF$_6$ (PC-II)

^{1}H NMR (400 MHz, Dimethyl sulfoxide-*d6*): δ 8.87 (d, *J* = 8.2 Hz, 2H), 8.46 – 8.38 (m, 4H), 8.32 (td, *J* = 7.9, 1.6 Hz, 2H), 7.97 – 7.93 (m, 2H), 7.71 (ddd, *J* = 7.6, 5.5, 1.2 Hz, 2H), 7.57 – 7.52 (m, 2H), 7.12 – 7.00 (m, 2H), 5.74 (dd, *J* = 8.3, 2.3 Hz, 2H) ppm.

[Ir(dF(CF$_3$)ppy)$_2$(4,4'-dCF$_3$bpy)]PF$_6$ (PC-III)

^{1}H NMR (400 MHz, *Acetone-d6*): δ 9.45 (s, 2H), 8.68 – 8.56 (m, 4H), 8.45 – 8.35 (m, 2H), 8.11 (s, 4H), 6.88 (t, *J* = 11.1 Hz, 2H), 5.92 (d, *J* = 8.6 Hz, 2H) ppm. **^{19}F NMR (377 MHz, *Acetone-d6*):** δ -63.3, -65.2, -71.6, -73.5 ppm.

Ir(dF(CF$_3$)ppy)$_2$(5,5'-dCF$_3$bpy)]PF$_6$ (PC-IV)

^{1}H NMR (400 MHz, *Acetone-d6*): δ 9.31 – 9.13 (m, 2H), 8.87 – 8.74 (m, 2H), 8.67 – 8.55 (m, 3H), 8.54 – 8.51 (m, 1H), 8.40 – 8.34 (m, 2H), 8.23 – 8.11 (m, 2H), 6.95 – 6.83 (m, 2H), 6.02 – 5.93 (m, 2H) ppm.

Preparation of PC-VIII:

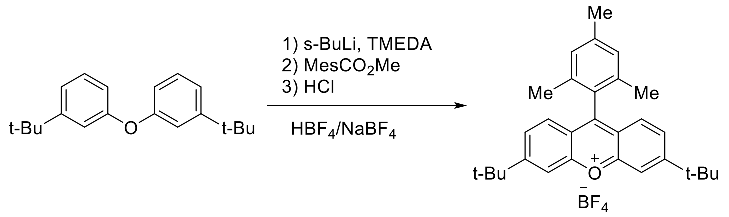

3,6-di-tert-butyl-9-mesitylxanthylium tetrafluoroborate:

To a 250 mL round bottom flask were added 3,3'-oxybis(tert-butylbenzene) (2.82 g, 10 mmol, 1 equiv.), TMEDA (3.0 mL, 20.5 mmol, 2.05 equiv.) and hexane (10 mL) under nitrogen atmosphere. The resulting solution was cooled to 0 °C by ice bath and s-BuLi (1.4 M in cyclohexane, 14.6 mL, 20.5 mmol, 2.05 equiv.) was added dropwise. The ice bath was then removed and the reaction mixture was stirred at ambient

temperature for 4 h. The reaction was cooled to –78 ºC and a solution of methyl 2,4,6-trimethylbenzoate (1.78 g, 10 mmol, 1.0 equiv.) in anhydrous hexane (10 mL) was added slowly via cannula. The reaction was then warmed to room temperature slowly and stirred for additional 12 h. The reaction was quenched with water (10 mL) and the biphasic mixture was stirred vigorously for 30 min. The mixture was diluted with 35 mL of Et_2O and the layers were separated. The organic layer was washed with water and brine. The organic layer was transferred to a 100 mL round bottom flask equipped with a stir bar. To the vigorously stirred solution was added conc. HCl (4.2 mL), resulting in a bright yellow precipitate that slowly turned brown over the course of addition. The brown suspension was stirred vigorously for 30 min then diluted with water (50 mL). The layers were separated, and the organic layer was extracted with water (3 x 50 mL or until the washings become colorless). To the combined aqueous layers was added solid $NaBF_4$ (3.29 g, 30 mmol, 3 equiv), resulting in a bright yellow precipitate. The resulting suspension was extracted with DCM (3 x 50 mL or until the washings become colorless). To the combined organic layers was added HBF_4-Et_2O complex (1.22 mL, 10 mmol, 1 equiv.). The solution was swirled to achieve homogeneity and then washed with water and $NaBF_4$ aq. (1 M, 35 mL). The organic layer was dried over solid $NaBF_4$, filtered, and concentrated under reduced pressure. The residue was purified by trituration with hexanes and filtered. The solid was dried in vacuo to give **3,6-di-tert-butyl-9-mesitylxanthylium tetrafluoroborate** as a yellow-orange solid.

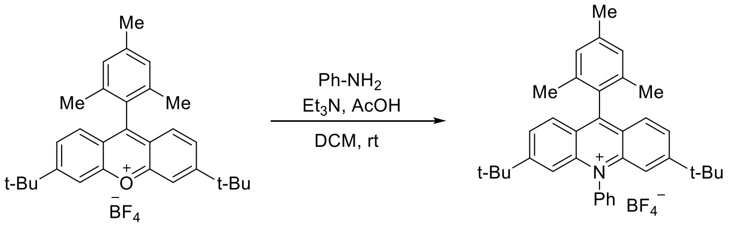

3,6-di-tert-butyl-9-mesityl-10-phenylacridin-10-ium tetrafluoroborate (PC VIII):

To a dry 50 mL round bottom flask under nitrogen were added 3,6-di-tert-butyl-9-mesitylxanthylium tetrafluoroborate (2.5 g, 5 mmol, 1 equiv.) and dry DCM (10 mL).

To the resulting solution were added acetic acid (0.85 mL, 15 mmol, 3 equiv.) followed by Et_3N (1.05 mL, 7.5 mmol, 1.5 equiv.). Aniline (0.55 mL, 6.0 mmol, 1.2 equiv.) was then added dropwise. The flask was covered with aluminum foil and stirred at room temperature for 12 h. The reaction was transferred to a separatory funnel and washed with water (15 mL) followed by sat. aq. $NaHCO_3$ (15 mL). To the organic layer was added HBF_4-Et_2O complex (0.61 mL, 5 mmol, 1 equiv.). The solution was swirled to achieve homogeneity then washed with water (15 mL) and aq. $NaBF_4$ (1 M, 15 mL). The organic layer was dried over solid $NaBF_4$, filtered and concentrated under reduced pressure. The residue was purified by trituration with 1:2 Et_2O/hexanes and filtered. The solid was dried in vacuo to give PC-VIII (2.44 g, 4.25 mmol, 85% yield) as a bright yellow solid. **^{1}H NMR (400 MHz, Chloroform-*d*):** δ 7.95 – 7.92 (m, 2H), 7.92 – 7.86 (m, 1H), 7.77 (s, 4H), 7.71 (d, *J* = 7.9 Hz, 2H), 7.39 (s, 2H), 7.15 (s, 2H), 2.47 (s, 3H), 1.84 (s, 6H), 1.27 (s, 9H) ppm; **^{13}C NMR (100 MHz, Chloroform-*d*):** δ 163.5, 162.2, 142.0, 140.1, 136.8, 136.0, 131.7, 131.6, 129.1, 128.8, 128.2, 128.0, 127.4, 124.0, 114.9, 36.5, 30.1, 21.3, 20.1 ppm

Substrate Preparation

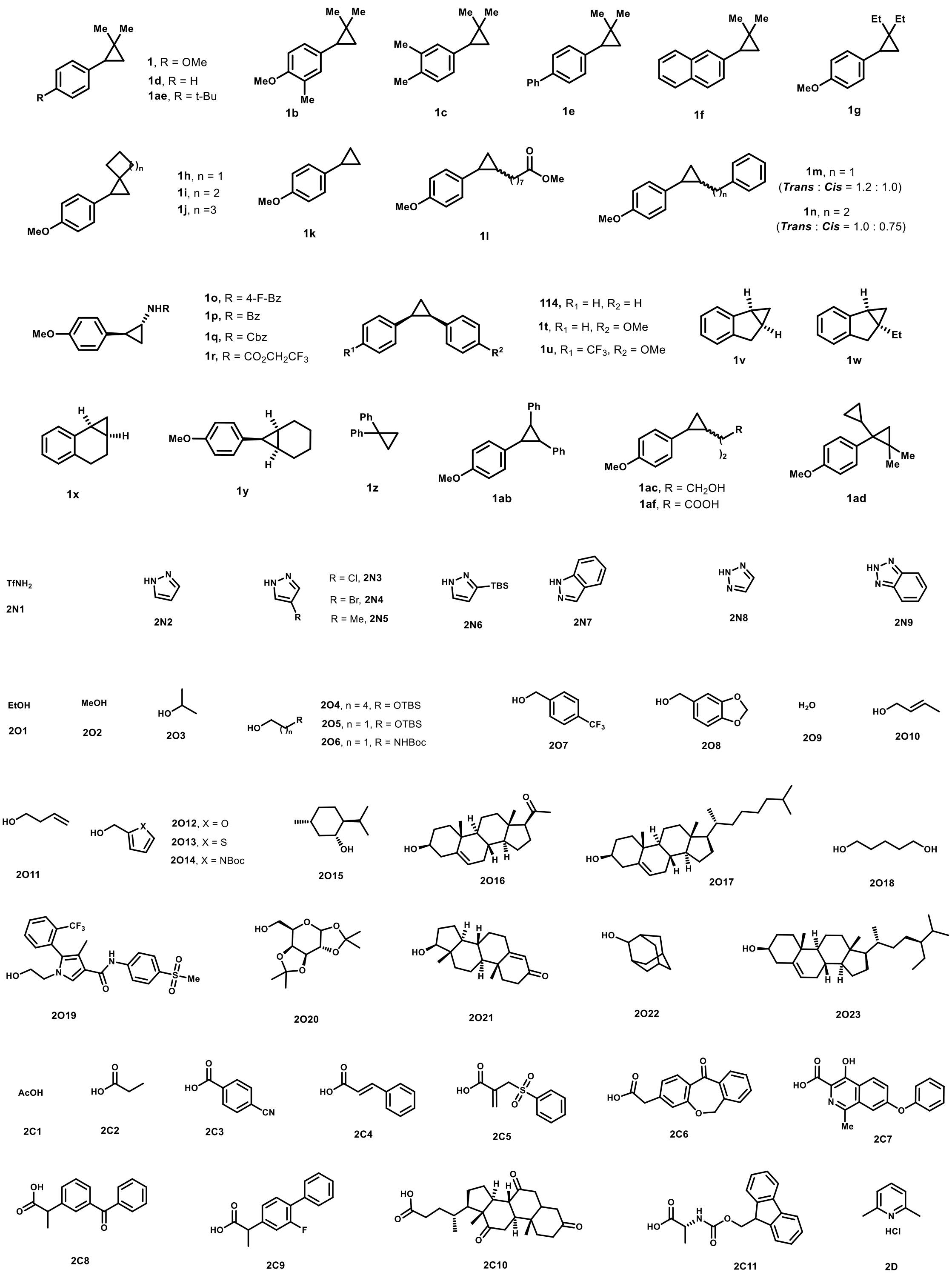

Compounds **1**, **1b-1ad**, **114**, **115** and **117** were prepared according to the literature procedures and typical synthetic protocols are shown below[6], all the spectroscopic data

are in agreement with the literature reports, including stereochemistry. **2N1-2N5, 2N6-2N9, 2O1-2O3, 2O5-2O22**, **2C1-2C11** were purchased from Energy.

General Procedure A for Aryl Cyclopropanes Synthesis

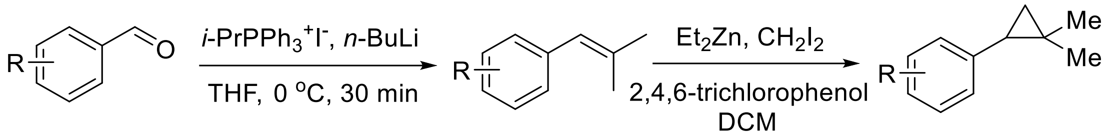

a) To a 150 mL oven-dried round-bottom flask equipped with a stir bar, the isopropyltriphenylphosphonium iodide (6.0 g, 13.88 mmol, 1.2 equiv.) and anhydrous THF (70 mL, 0.2 M) was added. The reaction flask was capped with rubber septum and charged with N_2 balloon and then the reaction mixture was cooled to 0 °C. *n*-BuLi (2.5M, 5.6 mL, 14 mmol, 1.2 equiv.) was added dropwise by syringe and the reaction mixture was stirred at this temperature for 30 min. The corresponding solution of aldehyde (11.6 mmol, 1.0 equiv.) in THF (20 mL) was added by syringe and the reaction mixture was allowed to warm to room temperature, and then stirred for 16 h. After the reaction reached completion according to the TLC analysis, the reaction mixture was quenched by sat. NH_4Cl (30 mL) and extracted with EtOAc (100 mL) for three times. The combined organic layers were washed with H_2O_2 (10 wt% in water, 10 mL) and brine (20 mL), dried over Na_2SO_4, and filtered. After the volatile materials were removed under reduced pressure, the crude residue was purified by column chromatography (PE : EtOAc = 50 : 1 to 10 : 1) to afford the desired alkene.

b) To a 150 mL oven-dried round bottom flask with a stir bar was added 2,4,6-trichlorophenol (1.18 g, 6.0 mmol, 2.5 equiv.) under nitrogen atmosphere. DCM (60 mL, 0.1 M) was added into the flask and the reaction mixture was cooled to -40 °C. $ZnEt_2$ (1.0 M, 6.0 mL, 6.0 mmol, 2.5 equiv.) was added slowly into the flask by syringe and the reaction mixture was stirred at this temperature for 15 min. CH_2I_2 (2.57 g, 9.6 mmol, 4.0 equiv.) was added slowly by syringe and the reaction mixture was stirred at this temperature for another 15 min. Next, the corresponding solution of alkene (2.4 mmol, 1.0 equiv.) in DCM (10 mL) was added by syringe and the reaction mixture was allowed to warm to room temperature and stirred for 16 h. After the reaction reached

completion (as judged by ^{1}H NMR of an aliquot removed from the reaction vessel and worked up by evaporation), the reaction mixture was quenched with saturated NH_4Cl (30 mL) and extracted with DCM (100mL) for three times. The combined organic layers were washed with aq. NaOH (1.0 M, 30 mL) and brine (20 mL), dried over Na_2SO_4 (20mL) and filtered. After the volatile materials were removed under reduced pressure, the crude residue was purified by column chromatography (PE/EtOAc) to afford the desired compound.

Compounds **1**, **1b-1d**, **1f, 1k**, and **1v-1x** were prepared following the General Procedure A.

1-(2,2-dimethylcyclopropyl)-4-methoxybenzene (**1**)

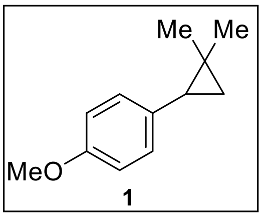

^{1}H NMR (400 MHz, Chloroform-*d*): δ 7.08 (d, *J* = 8.7 Hz, 2H), 6.81 (d, *J* = 8.7 Hz, 2H), 3.79 (s, 3H), 1.82 (dd, *J* = 8.2, 6.1 Hz, 1H), 1.20 (s, 3H), 0.78 (s, 3H), 0.75-0.68 (m, 2H) ppm; **^{13}C NMR (100 MHz, Chloroform-*d*):** δ 152.5, 132.4, 129.8, 113.3, 55.1, 28.8, 27.3, 20.4, 18.3.

4-(2,2-dimethylcyclopropyl)-1-methoxy-2-methylbenzene (1b)

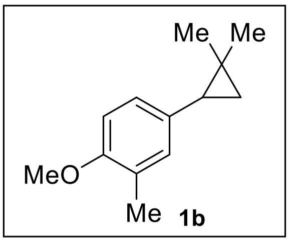

^{1}H NMR (400 MHz, Chloroform-*d*): δ 6.97 (dd, *J* = 2.0, 1.2 Hz, 1H), 6.96 – 6.93 (m, 1H), 6.74 (d, *J* = 8.1 Hz, 1H), 3.82 (s, 3H), 2.22 (s, 3H), 1.80 (t, *J* = 7.1 Hz, 1H), 1.22 (s, 3H), 0.81 (s, 3H), 0.75 – 0.70 (m, 2H) ppm; **^{13}C NMR (100 MHz, Chloroform-*d*):** δ 155.8, 131.9, 131.5, 126.8, 125.8, 109.4, 55.3, 28.9, 27.4, 20.5, 18.5, 18.3, 16.3 ppm; **HRMS (ESI, m/z):** calculated for $[M+H]^+$: 191.1436, found: 191.1435.

4-(2,2-dimethylcyclopropyl)-1,2-dimethylbenzene (1c)

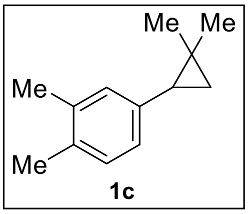

^{1}H NMR (400 MHz, Chloroform-*d*): δ 6.94 (d, *J*= 8.7 Hz, 2H), 6.73 (d, *J*= 8.1 Hz, 1H), 3.81 (s, 3H), 2.21 (s, 3H), 1.87 –1.68 (m, 1H), 1.21 (s, 3H), 0.80 (s, 3H), 0.72 (d, *J*= 1.3 Hz, 1H), 0.70 (s, 1H) ppm; **^{13}C NMR (100 MHz, Chloroform-*d*):** δ 137.7, 135.9,

133.5, 130.4, 129.1, 126.16, 29.4, 20.4, 19.8, 19.4, 18.7, 18.3 ppm; **HRMS (ESI, m/z):** calculated for $[M+H]^+$: 175.1487, found: 175.1482.

(2,2-dimethylcyclopropyl)benzene (1d)

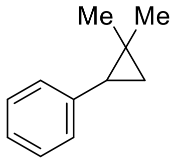

1d

^{1}H NMR (400 MHz, Chloroform-*d*): δ 7.30 – 7.24 (m, 2H), 7.20 – 7.14 (m, 3H), 1.93 – 1.88 (m, 1H), 1.24 (s, 3H), 0.84 – 0.75 (m, 5H), ppm; **^{13}C NMR (100 MHz, Chloroform-*d*):** δ 140.4, 129.0, 127.9, 125.6, 29.8, 27.6, 20.4, 19.1, 18.4 ppm.

2-(2,2-dimethylcyclopropyl)naphthalene (1f)

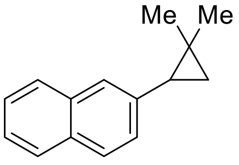

1f

^{1}H NMR (400 MHz, Chloroform-*d*): δ 7.85 – 7.79 (m, 2H), 7.78 (d, *J* = 8.5 Hz, 1H), 7.59 (s, 1H), 7.50 – 7.41 (m, 2H), 7.39 (dd, *J* = 8.4, 1.7 Hz, 1H), 2.07 (dd, *J* = 8.3, 5.9 Hz, 1H), 1.31 (s, 3H), 1.02 – 0.96 (m, 1H), 0.89 (dd, *J* = 8.4, 4.7 Hz, 1H), 0.85 (s, 3H) ppm; **^{13}C NMR (100 MHz, Chloroform-*d*):** δ 138.1, 133.5, 132.0, 128.4, 127.6, 127.5, 127.3, 126.5, 125.8, 125.0, 30.1, 27.5, 20.4, 19.4, 18.5 ppm; **HRMS (ESI, m/z):** calculated for $[M+Na]^+$: 219.1150, found: 219.1139.

1-cyclopropyl-4-methoxybenzene (1k)

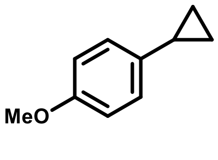

1k

^{1}H NMR (400 MHz, Chloroform-*d*): δ 7.05 – 6.98 (m, 2H), 6.85 – 6.78 (m, 2H), 3.78 (s, 3H), 1.92 – 1.80 (m, 1H), 0.94 – 0.85 (m, 2H), 0.67 – 0.58 (m, 2H) ppm; **^{13}C NMR (100 MHz, Chloroform-*d*):** δ 157.7, 136.0, 126.9, 113.8, 55.4, 14.7, 8.7 ppm.

1,1a,6,6a-tetrahydrocyclopropa[a]indene (1v)

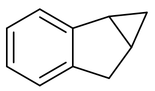

1v

^{1}H NMR (400 MHz, Chloroform-*d*): δ 7.30–7.28 (m, 1H), 7.18–7.03 (m, 3H), 3.17 (dd, *J* = 16.8, 6.8 Hz, 1 H), 2.92 (d, *J* = 16.8 Hz, 1 H), 2.36–2.32 (m, 1 H), 1.84– 1.81 (m, 1 H), 1.05 (dt, *J* = 8.0, 4.4 Hz, 1 H), 0.05 (q, *J* = 4.4 Hz, 1 H). **^{13}C NMR (100 MHz, Chloroform-*d*):** δ 147.2, 142.2, 126.0, 125.6, 125.5, 123.5, 35.6, 24.1, 16.8, 16.2 ppm.

6a-ethyl-1,1a,6,6a-tetrahydrocyclopropa[a]indene (1w)

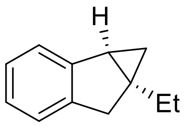

1w

^{1}H NMR (400 MHz, Chloroform-*d*): δ 7.25 (d, *J* = 7.9 Hz, 1H), 7.14 (d, *J* = 7.1 Hz, 1H), 7.12 – 7.03 (m, 2H), 3.01 (s, 2H), 2.14 (dd, *J* = 7.9, 2.8 Hz, 1H), 1.75 (dq, *J* = 14.5, 7.4 Hz, 1H), 1.54 (dq, *J* = 14.7, 7.5 Hz, 1H), 1.03 (t, *J* = 7.4 Hz, 3H), 1.01 – 0.98 (m, 1H), 0.24 (t, *J* = 3.6 Hz, 1H) ppm; **^{13}C NMR (100 MHz, Chloroform-*d*):** δ 147.8, 142.5, 125.8, 125.3, 125.1, 123.0, 39.3, 29.8, 29.6, 28.7, 22.8, 11.8 ppm; **HRMS (ESI, m/z):** calculated for $[M+Na]^+$: 181.0993, found: 181.0995.

1a,2,3,7b-tetrahydro-1H-cyclopropa[a]naphthalene (1x)

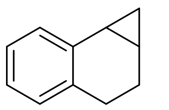

1x

^{1}H NMR (400 MHz, Chloroform-*d*): δ 7.30-6.98 (m, 4H), 2.64-2.41 (m, 2H), 2.16 – 2.08 (m, 1H), 1.97- 1.87 (m, 1H), 1.76 – 1.69 (m, 1H), 1.64 -1.52 (m, 1H), 0.85 (t, *J* = 7.2 Hz, 2H) ppm; **^{13}C NMR (100 MHz, Chloroform-*d*):** δ 139.1, 134.0, 128.7, 128.4, 126.1, 124.9, 25.7, 19.7, 15.8, 14.6, 8.6 ppm.

General Procedure B for Aryl Cyclopropanes Synthesis

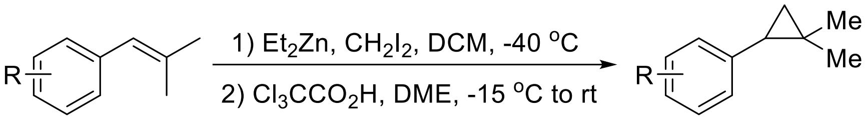

To a 50 mL oven-dried round-bottom flask equipped with a stir bar, was added DCM (3 mL, 0.67 M) under nitrogen atmosphere and was cooled to -40 °C. $ZnEt_2$(2.0 M, 2.5 mL, 5.0 mmol, 2.5 equiv.) was added followed by slow addition of a solution of CH_2I_2 (2.68 g, 10.0 mmol, 5.0 equiv.) in DCM (1 mL) by syringe. The reaction mixture was stirred at this temperature for 1 h followed by warming to -10 °C. Next, a solution of trichloroacetic acid (60 mg, 0.4 mmol, 0.2 equiv.) and DME (224 mg, 2.4 mmol, 1.2 equiv.) in DCM (1 mL) was added dropwise into and the reaction mixture by syringe and the resulting solution was allowed to stir at -10 °C for another 1 h. A solution of corresponding alkene (2.0mmol, 1.0 equiv.) in DCM (1 mL) was then added by syringe and the reaction mixture was allowed to warmed to room temperature and stirred for 16

h. After the reaction reached completion (judged by ^{1}H-NMR of an aliquot removed from the reaction vessel and worked up by evaporation), the reaction mixture was quenched with sat. NH_4Cl (30 mL) and extracted with DCM (20 mL) for three times. The combined organic layers were washed with aq. NaOH (1.0 M, 30 mL) and brine (20 mL), dried over Na_2SO_4 and filtered. After the volatile materials were removed under reduced pressure, the crude residue was purified by column chromatography (PE/ EtOAc) to afford the corresponding title compound.

Compounds **1e**, **1z** and **1ae** were prepared following the General Procedure B.

4-(2,2-dimethylcyclopropyl)-1,1'-biphenyl (1e)

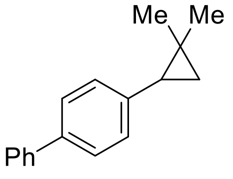

1e

^{1}H NMR (400 MHz, Chloroform-*d*): δ 7.57 (d, *J* = 7.6 Hz, 2H), 7.49 (d, *J* = 7.8 Hz, 2H), 7.39 (t, *J* = 7.6 Hz, 2H), 7.29 (t, *J* = 7.4 Hz, 1H), 7.21 (d, *J* = 7.8 Hz, 2H), 1.89 (t, *J* = 7.1 Hz, 1H), 1.23 (s, 3H), 0.83 (s, 3H), 0.82 – 0.78 (m, 1H) ppm; **^{13}C NMR (100 MHz, Chloroform-*d*):** δ 141.2, 139.6, 138.4, 129.4, 128.8, 127.0, 126.7, 29.6, 27.6, 20.5, 19.4, 18.7 ppm; **HRMS (ESI, m/z):** calculated for $[M+H]^+$: 223.1487, found: 223.1479.

cyclopropane-1,1-diyldibenzene (1z)

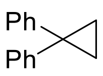

1z

^{1}H NMR (400 MHz, Chloroform-*d*): δ 7.39 – 7.22 (m, 10H), 1.40 (s, 4H) ppm; **^{13}C NMR (100 MHz, Chloroform-*d*):** δ 145.9, 128.6, 128.5, 126.1, 30.1, 16.7 ppm.

1-(*tert*-butyl)-4-(2,2-dimethylcyclopropyl)benzene (1ae)

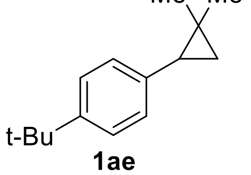

1ae

^{1}H NMR (400 MHz, Chloroform-*d*): δ 7.30 (d, *J* = 8.3 Hz, 2H), 7.11 (d, *J* = 8.3 Hz, 2H), 1.88 – 1.83 (m, 1H), 1.34 (d, *J* = 1.8 Hz, 9H), 1.24 (s, 3H), 0.84 (s, 3H), 0.81 – 0.73 (m, 2H) ppm; **^{13}C NMR (100 MHz, Chloroform-*d*):** δ 148.3, 137.4, 128.7, 124.8, 34.4, 31.6, 29.4, 27.6, 20.5, 19.0, 18.6 ppm; **HRMS (ESI, m/z):** calculated for $[M+H]^+$: 203.1800, found: 203.1793.

General Procedure C for Diaryl Cyclopropanes Synthesis

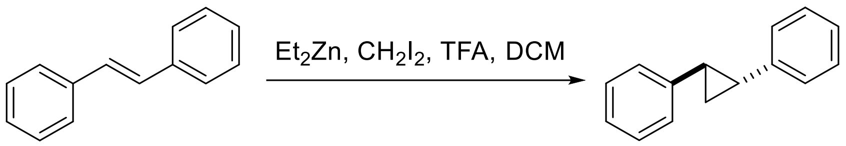

Following the literature method,[6] to a 50 mL oven-dried round-bottom flask with a stir bar was added DCM (3 mL, 0.67 M) under a nitrogen atmosphere. The flask was cooled to 0 °C. $ZnEt_2$ (2.0 M, 2.8 mL, 5.5 mmol, 5.5 equiv.) was added dropwise and followed by dropwise addition of a solution of TFA (0.5 mL, 6.0 mmol, 6.0 equiv.) in DCM (1 mL) by syringe over 15 min. The solution was then stirred at this temperature for 15 min. A solution of CH_2I_2(1.61 g, 6.0 mmol, 6.0 equiv.) in DCM (1 mL) was added by syringe and the reaction mixture was stirred at this temperature for 1 h. Then a solution of the corresponding alkene (1 mmol, 1.0 equiv.) in DCM (1 mL) was added by syringe and the reaction mixture was allowed to warm to room temperature and stirred for 20 h. After the reaction reached completion (judged by ^{1}H-NMR of an aliquot removed from the reaction vessel and worked up by evaporation), the reaction mixture was quenched with sat. NH_4Cl (30 mL) and extracted with DCM (50 mL) for three times. The combined organic layers were washed with sat. $NaHCO_3$ (50 mL) and brine (20 mL), dried over Na_2SO_4 and filtered. After the volatile materials were removed under reduced pressure, the crude residue was purified by column chromatography (PE/EtOAc) to afford the desired compound.

Racemic substrate **114** was prepared following the General Procedure C.

trans-1,2-diphenylcyclopropane (114)

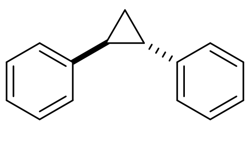

114

^{1}H NMR (400 MHz, Chloroform-*d*): δ 7.35 – 7.27 (m, 4H), 7.23 – 7.12 (m, 6H), 2.23 – 2.13 (m, 2H), 1.51 – 1.42 (m, 2H) ppm; **^{13}C NMR (100 MHz, Chloroform-*d*):** δ 142.6, 128.5, 125.9, 28.1, 18.4 ppm.

General Procedure D for Aryl Cyclopropanes Synthesis

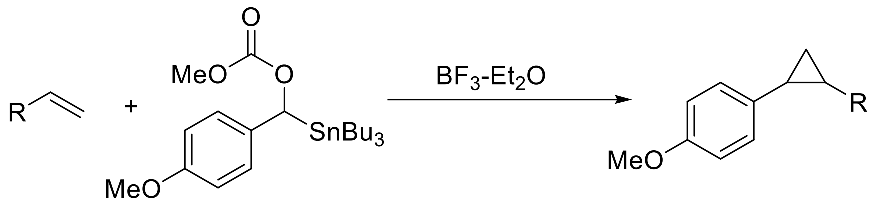

To a 50 mL oven-dried round-bottom flask equipped with a stir bar was added the solution of (4-methoxyphenyl)(tributylstannyl)methyl methyl carbonate (486 mg, 1.0 mmol, 1.0 equiv.) and the corresponding alkene (1.1 mmol, 1.0 equiv.) in toluene (3.5 mL) under nitrogen atmosphere at room temperature. The reaction vessel was cooled to -23 °C. $BF_3 \cdot OEt_2$(156 mg, 1.1 mmol, 1.1 equiv.) was added by syringe and stirred at this temperature for 2 h. After the reaction reached completion according to the TLC analysis, the reaction mixture was quenched wi8th sat. $NaHCO_3$ (10 mL) and extracted with EtOAc (30 mL) for three times. The combined organic layers were washed with brine (20 mL), dried over Na_2SO_4 and filtered. After the volatile materials were removed under reduced pressure, the crude residue was purified by column chromatography (PE/EtOAc) to afford the target compound.

Compounds **1l**, **1t**, **1u** and **1y** was prepared following the General Procedure D.

methyl 8-(2-(4-methoxyphenyl)cyclopropyl)octanoate (1l)

Only spectra of the major isomer is provided.

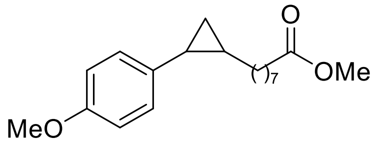

1l

***Cis* isomer ^{1}H NMR (400 MHz, Chloroform-d):** δ 7.10 (d, *J* = 8.3 Hz, 2H), 6.81 (d, *J* = 8.7 Hz, 2H), 3.78 (s, 3H), 3.66 (s, 3H), 2.31 – 2.25 (m, 2H), 2.03 (td, *J* = 8.5, 5.9 Hz, 1H), 1.58 (p, *J* = 7.5 Hz, 2H), 1.31 – 1.12 (m, 9H), 1.04 – 0.96 (m, 1H), 0.93 – 0.90 (m, 1H), 0.87 – 0.81 (m, 1H), 0.54 (q, *J* = 5.6 Hz, 1H) ppm; **^{13}C NMR (100 MHz, Chloroform-*d*):** δ 174.4, 157.7, 131.7, 130.1, 113.3, 55.3, 51.5, 34.2, 29.4, 29.2, 29.2, 28.7, 25.0, 20.2, 18.8, 9.7 ppm; **HRMS (ESI, m/z):** calculated for $[M+H]^+$: 305.2111, found: 305.2106.

1-methoxy-4-((1S,2S)-2-phenylcyclopropyl)benzene (1t)

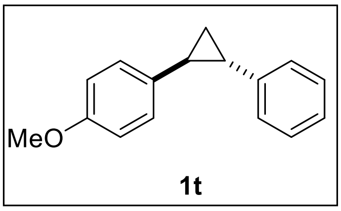

^{1}H NMR (400 MHz, Chloroform-d): δ 7.14 – 7.05 (m, 3H), 6.96 – 6.85 (m, 4H), 6.69 – 6.61 (m, 2H), 3.70 (s, 3H), 2.48 – 2.39 (m, 2H), 1.71 – 1.59 (m, 1H), 1.47 – 1.41 (m, 1H) ppm; **^{13}C NMR (100 MHz, Chloroform-*d*):** δ 157.7, 138.8, 130.4, 130.2, 128.9, 127.8, 125.5,113.2, 55.2, 28.0, 27.0, 17.6 ppm.

1-methoxy-4-((1S,2S)-2-(4-(trifluoromethyl)phenyl)cyclopropyl)benzene (1u)

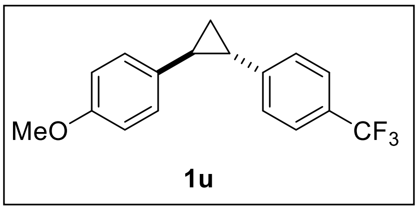

^{1}H NMR (400 MHz, Chloroform-d): δ 7.31 (d, *J* = 8.1 Hz, 2H), 6.95 (d, *J* = 8.0 Hz, 2H), 6.91 – 6.85 (m, 2H), 6.70 – 6.61 (m, 2H), 3.71 (s, 3H), 2.56 – 2.37 (m, 2H), 1.68 – 1.58 (m, 1H), 1.53 – 1.45 (m, 1H) ppm; **^{19}F NMR (377 MHz, Chloroform-*d*):** -62.10 ppm. **^{13}C NMR (100 MHz, Chloroform-*d*):** δ 157.9, 143.4, 130.4, 129.4, 128.7, 124.6 (q, *J* = 3.8 Hz), 124.4 (q, *J* = 272.0 Hz), 113.4, 55.2, 27.9, 27.0, 17.6 ppm.

(1R,6S,7s)-7-(4-methoxyphenyl)bicyclo[4.1.0]heptane (1y)

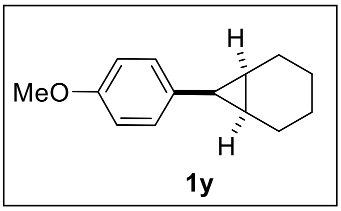

^{1}H NMR (400 MHz, Chloroform-d): δ 7.23 – 7.18 (m, 2H), 6.90 – 6.84 (m, 2H), 3.80 (s, 3H), 1.97 – 1.83 (m, 3H), 1.73 – 1.59 (m, 2H), 1.27 – 1.18 (m, 2H), 1.05 – 1.01 (m, 2H), 0.75 – 0.61 (m, 2H) ppm; **^{13}C NMR (100 MHz, Chloroform-*d*):** δ 157.7, 132.2, 130.6, 113.7, 55.3, 21.9, 21.3, 20.3, 12.8 ppm.

Synthetic Procedure for 1g

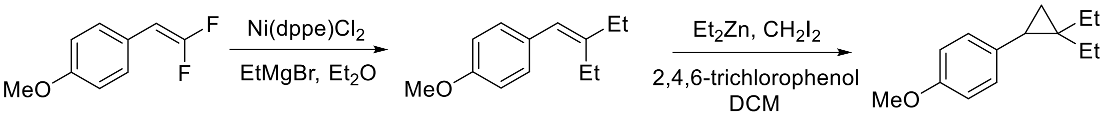

a) To anoven-dried 50 mL round-bottom flask equipped with a stir bar, 1-(2,2-difluorovinyl)-4-methoxybenzene (340 mg, 2.0 mmol, 1.0 equiv.), $NiCl_2$(dppe) (42 mg, 4 mol%) and Et_2O (15 mL) was added under nitrogen atmosphere at room temperature. To this stirring solution at room temperature, EtMgBr (1.0 M in THF, 4.8 mL, 4.8 mmol,

2.4 equiv.) was added slowly by syringe and the resulting mixture was stirred for an additional 2 h. Once the reaction was judged to be completed by TLC analysis, the reaction mixture was quenched with sat. NH_4Cl (20 mL) and extracted with EtOAc (30 mL) three times. The combined organic layers were washed with sat. $NaHCO_3$ (30 mL) and brine (20 mL), dried over Na_2SO_4 and filtered. After the volatile materials were removed under reduced pressure, the crude residue was purified by column chromatography (PE : EtOAc = 50 : 1 to 10: 1)to afford 1-(2-ethylbut-1-en-1-yl)-4-methoxybenzene as colorless oil (294 mg, 1.54 mmol, yield 77%).

b) Compound **1g** was prepared from 1-(2-ethylbut-1-en-1-yl)-4-methoxybenzene following the cyclopropanation method in General Procedure A and purified by column chromatography (PE : EtOAc = 50 : 1 to 10 : 1) and was obtained in 67% yield as a colorless liquid.

1-(2,2-diethylcyclopropyl)-4-methoxybenzene (1g)

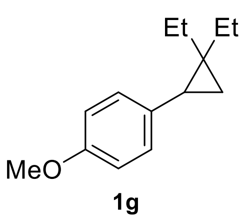

1g

^{1}H NMR (400 MHz, Chloroform-*d*): δ 7.10 (d, J = 8.6 Hz, 2H), 6.82 (d, J = 8.6 Hz, 2H), 3.79 (s, 3H), 1.85 (dd, J = 8.3, 6.1 Hz, 1H), 1.61 (dd, J = 14.0, 7.1 Hz, 1H), 1.24 – 1.13 (m, 2H), 0.99 (t, J = 7.4 Hz, 3H), 0.82 – 0.74 (m, 4H), 0.73 – 0.66 (m, 2H) ppm; **^{13}C NMR (100 MHz, Chloroform-*d*):** δ 157.5, 132.1, 130.0, 113.2, 55.2, 29.4, 28.7, 28.5, 22.8, 16.7, 10.9, 10.5 ppm; **HRMS (ESI, m/z):** calculated for $[M+H]^+$: 205.1592, found: 205.1590.

Synthetic Procedure for 1h

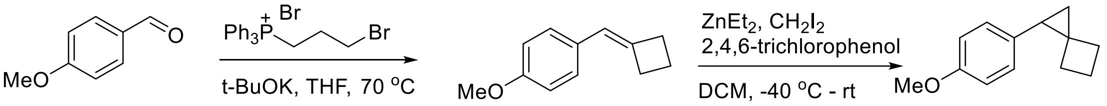

a) A flame-dried flask equipped with a stir bar was charged with (4-bromobutyl)triphenylphosphonium bromide (1.79 g, 3.75 mmol, 1.5 equiv.) and t-BuOK (842 mg, 7.50 mmol, 3.0 equiv.). The solids were suspended in THF (10 mL), and the mixture was allowed to stir at 70 °C for 1 h. After the solution had cooled to room temperature, p-anisaldehyde (340 mg, 2.5 mmol, 1.0 equiv.) was added to the suspension dropwise under nitrogen atmosphere. The vial was sealed and heated to

70 °C for 12 h. After cooling down to room temperature, the reaction was quenched with a saturated aq. NH_4Cl solution and extracted with EtOAc. The combined organic phases were dried over Na_2SO_4 and concentrated in vacuo. The resulting crude mixture was purified by flash chromatography (hexanes) to provide 1-(cyclobutylidenemethyl)-4-methoxybenzene (65% yield) as a colorless oil.

b) Compound **1h** was prepared from 1-(cyclobutylidenemethyl)-4-methoxybenzene following the cyclopropanation method in General Procedure A, purified by column chromatography (PE : EtOAc = 50 : 1 to 10 : 1) and was obtained in 75% yield as a colorless liquid.

1-(4-methoxyphenyl)spiro[2.3]hexane (1h)

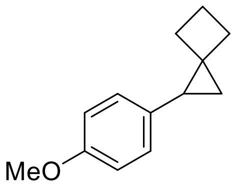

1h

^{1}H NMR (400 MHz, Chloroform-*d*): δ 6.92 – 6.86 (m, 2H), 6.84 – 6.76 (m, 2H), 3.77 (s, 3H), 2.29 – 1.85 (m, 6H), 1.77 (dd, *J* = 8.9, 5.9 Hz, 1H), 0.99 (dd, *J* = 9.0, 5.2 Hz, 1H), 0.75 (t, *J* = 5.6 Hz, 1H) ppm; **^{13}C NMR (100 MHz, Chloroform-*d*):** δ 157.4, 133.4, 127.7, 113.6, 55.4, 31.4, 28.0, 27.4, 26.5, 20.0, 16.7 ppm.

Synthetic Procedure for 1i

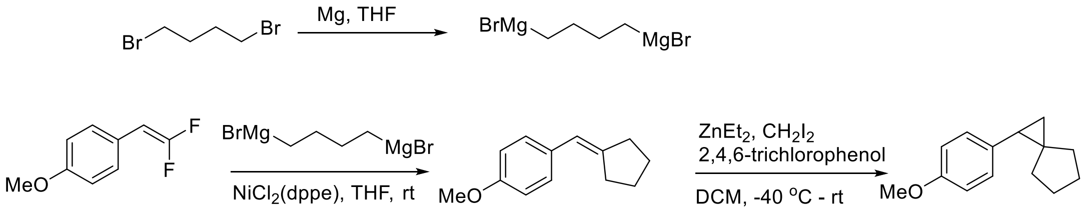

a) To an oven-dried 100 mL three-necked round-bottom flask equipped with a stir bar, magnesium turnings (2.7 g, 110 mmol, 2.2 equiv..) were added followed by anhydrous THF (15mL) under nitrogen atmosphere. Then a solution of 1,4-dibromobutane (50.0 mmol, 1.0 equiv.) in anhydrous THF (5 mL) was added dropwise by syringe at room temperature and the reaction mixture was further stirred at room temperature for 2 h to give the Grignard reagent (1.0 M). To a separate 50 mL oven-dried three-necked round-bottom flask equipped with a stir bar was added 1-(2,2-difluorovinyl)-4-

methoxybenzene (340 mg, 2.0 mmol, 1.0 equiv.), $NiCl_2$(dppe) (43 mg, 4 mol%) and THF (10 mL) under nitrogen atmosphere. Then the freshly prepared Grignard reagent (1.0 M, 2.0 mL, 2.0 mmol, 1.0 equiv.) was added by syringe and the reaction mixture was stirred at room temperature for 1 h. After the reaction reached completion according to the TLC analysis, the reaction mixture was quenched with sat. NH_4Cl (20 mL) and extracted with EtOAc (50 mL) for 3 times. The combined organic layers were washed with H_2O (50 mL) and brine (20 mL), dried over Na_2SO_4 and filtered. After the volatile materials were removed under reduced pressure, the crude residue was purified by column chromatography (PE: EtOAc = 50 : 1 to 10 : 1) to afford the 1-(cyclopentylidenemethyl)-4-methoxybenzene as a colorless oil.

b) Compound **1i** was prepared from 1-(cyclopentylidenemethyl)-4-methoxybenzene following the cyclopropanation method in General Procedure A, purified by column chromatography (PE : EtOAc = 50 : 1 to 10 : 1) and was obtained in 71% yield as a colorless liquid.

1-(4-methoxyphenyl)spiro[2.4]heptane (1i)

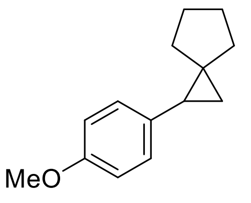

1i

^{1}H NMR (400 MHz, Chloroform-*d*): δ 7.02 (d, J = 8.6 Hz, 2H), 6.82 (d, J = 8.7 Hz, 2H), 3.79 (s, 3H), 1.93 (dd, J = 8.7, 6.0 Hz, 1H), 1.65 (dtt, J = 12.0, 8.3, 3.7 Hz, 4H), 1.61 – 1.53 (m, 2H), 1.38 – 1.28 (m, 1H), 1.27 – 1.18 (m, 1H), 1.00 (dd, J = 8.7, 4.8 Hz, 1H), 0.95 – 0.85 (m, 1H) ppm; **^{13}C NMR (100 MHz, Chloroform-*d*):** δ 157.5, 133.0, 129.1, 113.5, 55.3, 38.0, 30.7, 30.1, 29.1, 26.5, 26.5, 18.2 ppm; **HRMS (ESI, m/z):** calculated for $[M+H]^+$: 203.1436, found: 203.1432.

Synthetic Procedure for 1j

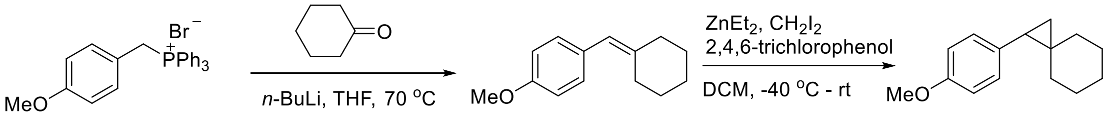

To an oven-dried 50 mL round-bottom flask with a stir bar, (4-methoxybenzyl) tris(phenyl) phosphonium bromide (2.0 g, 4.3 mmol, 1.0 equiv.) was added followed by anhydrous THF (10 mL, 0.4 M) under nitrogen atmosphere at room temperature. Next, *n*-BuLi (2.5 M in THF, 2.1 mL, 5.2 mmol, 1.2 equiv.) was added by syringe and

the reaction mixture was stirred at room temperature for 1 h. To the reaction mixture cyclohexanone (510 mg, 5.20 mmol, 1.2 equiv.) was added by syringe and the reaction mixture was stirred under reflux for 4 h. After the reaction reached completion according to the TLC analysis, the reaction mixture was cooled down to room temperature, quenched with sat. NH_4Cl (10 mL) with and extracted with EtOAc. The combined organic layers were washed with H_2O_2 (10 wt% in water, 10 mL) and brine, dried over Na_2SO_4 and filtered. After the volatile was removed under reduced pressure, the crude residue was purified by column chromatography (PE : EtOAc = 50 : 1 to 10 : 1) to afford the **1j** as a colorless oil (500 mg, 57%).

1-(cyclohexylidenemethyl)-4-methoxybenzene (**1j**)

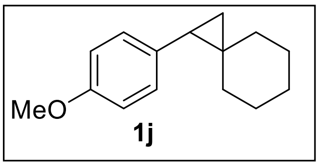

^{1}H NMR (400 MHz, Chloroform-*d*): δ 7.12 (d, *J* = 8.8 Hz, 2H), 6.82 (d, *J* = 8.7 Hz, 2H), 3.80 (s, 3H), 1.82 (dd, *J* = 8.2, 5.9 Hz, 1H), 1.59 (q, *J* = 7.1, 6.6 Hz, 2H), 1.45 (dq, *J* = 13.8, 5.8 Hz, 4H), 1.30 (dd, *J* = 10.2, 5.4 Hz, 2H), 1.12 – 1.01 (m, 2H), 0.76 (t, *J* = 5.2 Hz, 1H), 0.69 (dd, *J* = 8.4, 4.7 Hz, 1H) ppm; **^{13}C NMR (100 MHz, Chloroform-*d*):** δ 157.60, 131.98, 129.89, 113.28, 55.31, 38.02, 30.58, 28.71, 26.41, 26.29, 25.96, 25.13, 16.75 ppm; **HRMS (ESI, m/z):** calculated for $[M+H]^+$: 217.1592, found: 217.1586.

Synthetic Procedure for 1m and 1n

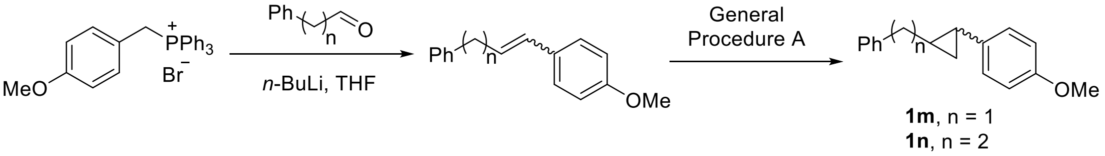

To an oven-dried 50 mL round-bottom flask with a stir bar, (4-methoxybenzyl)tris (phenyl)phosphonium bromide (1.0 g, 2.15 mmol,1.0 equiv.) was added followed by anhydrous THF (5mL, 0.4 M) under nitrogen atmosphere at room temperature. Next, *n*-BuLi (2.5 M in THF, 1.1 mL, 2.75 mmol, 1.2 equiv.) was added by syringe and the reaction mixture was stirred at room temperature for 1 h. To the reaction mixture,2-phenylacetaldehyde (280 mg, 2.36 mmol, 1.1equiv.) was added by syringe and the reaction mixture was stirred under reflux for 4 h. After the reaction reached completion

according to the TLC analysis, the reaction mixture was cooled to room temperatue, quenched with sat. NH_4Cl (10 mL) and extracted with EtOAc (50 mL) three times. The combined organic layers were washed with H_2O_2 (10 wt% in water, 10 mL) and brine, dried over Na_2SO_4 and filtered. After the volatile materials were removed under reduced pressure, the crude residue was purified by column chromatography (PE : EtOAc = 50 : 1 to 10 : 1) to afford 1-methoxy-4-(3-phenylprop-1-en-1-yl)benzene as a yellow oil (inseparable *E*- and *Z*-isomer mixture, 292 mg, 1.30 mmol, yield 60.5%).

1-methoxy-4-(4-phenylbut-1-en-1-yl)benzene was obtained from 3-phenylpropanal by the same reaction and was purified by column chromatography (PE : EtOAc = 50 : 1 to 10 : 1) to afford a yellow oil in 55% yield (*E-and Z-isomer* mixture).

Compound **1m** and **1n** were prepared following the General Procedure A from corresponding alkene.

1-(2-benzylcyclopropyl)-4-methoxybenzene (1m)

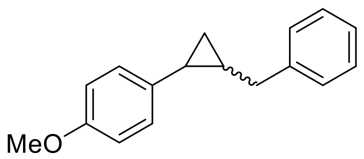

1m

(***Trans*** : ***Cis*** = 1.2 : 1.0)

***Cis* isomer ^{1}H NMR (400 MHz, Chloroform-*d*):** (resolved signals only) δ 7.33 – 7.15 (m, 5H), 6.99 – 6.95 (m, 2H), 6.81 – 6.76 (m, 2H), 3.76 (s, 3H), 2.77 (dd, *J* = 14.8, 6.7 Hz, 1H), 2.67 (dd, *J* = 14.8, 6.9 Hz, 1H), 1.75 (dt, *J* = 8.6, 4.9 Hz, 1H), 1.31 – 1.20 (m, 1H), 0.89 (ddt, *J* = 16.2, 8.5, 5.0 Hz, 2H) ppm; **^{13}C NMR (100 MHz, Chloroform-*d*):** δ 157.6, 141.5, 135.4, 130.2, 128.4, 126.9, 126.0, 113.8, 55.3, 40.0, 23.6, 22.6, 15.5 ppm.

***Trans* isomer ^{1}H NMR (400 MHz, Chloroform-*d*):** (resolved signals only) δ 7.33 – 7.15 (m, 5H), 7.09 (dd, *J* = 7.7, 1.2 Hz, 2H), 6.85 – 6.81 (m, 2H), 3.76 (s, 3H), 2.50 (dd, *J* = 15.0, 6.3 Hz, 1H), 2.23 – 2.10 (m, 2H), 1.35 (qt, *J* = 8.6, 6.1 Hz, 1H), 1.05 (td, *J* = 8.4, 5.1 Hz, 1H), 0.77 (q, *J* = 5.6 Hz, 1H) ppm; **^{13}C NMR (100 MHz, Chloroform-*d*):** δ 157.9, 142.2, 131.1, 128.4, 128.4, 128.2, 125.7, 113.4, 55.3, 34.6, 20.5, 19.6, 10.0 ppm; **HRMS (ESI, m/z):** calculated for $[M+H]^+$: 239.1436, found: 239.1435.

1-methoxy-4-(2-phenethylcyclopropyl)benzene (1n)

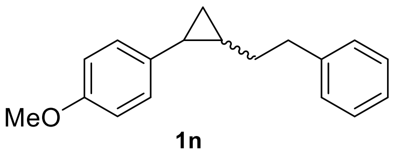

1n
(***Trans*** : ***Cis*** = 1.0 : 0.75)

***Cis* isomer ^{1}H NMR (400 MHz, Chloroform-*d*):** (resolved signals only) δ 7.30 – 7.13 (m, 5H), 7.07 – 7.00 (m, 2H), 6.83 (d, *J* = 8.7 Hz, 2H), 3.80 (s, 3H), 2.56 (ddd, *J* = 8.8, 6.8, 1.9 Hz, 2H), 2.10 (td, *J* = 8.6, 5.8 Hz, 1H), 1.41 (ddd, *J* = 13.9, 8.6, 7.0 Hz, 1H), 1.26 (ddd, *J* = 14.1, 6.9, 1.5 Hz, 1H), 1.14 – 1.03 (m, 1H), 0.95 (td, *J* = 8.4, 4.9 Hz, 1H), 0.61 (q, *J* = 5.5 Hz, 1H) ppm; **^{13}C NMR (100 MHz, Chloroform-*d*):** δ 157.7, 142.6, 131.3, 130.0, 128.4, 128.2, 125.6, 113.4, 55.3, 35.7, 31.0, 22.8, 20.2, 9.6 ppm.

***Trans* isomer ^{1}H NMR (400 MHz, Chloroform-*d*):** (resolved signals only) δ 7.30 – 7.13 (m, 5H), 6.96 (d, *J* = 8.7 Hz, 2H), 6.81 (d, *J* = 8.7 Hz, 2H), 3.78 (s, 3H), 2.77 (dd, *J* = 8.7, 6.6 Hz, 2H), 1.74 – 1.70 (m, 1H), 1.70 – 1.66 (m, 1H), 1.60 (dt, *J* = 9.1, 4.8 Hz, 1H), 1.02 (ddd, *J* = 12.0, 5.2, 2.4 Hz, 1H), 0.83 (dt, *J* = 8.4, 4.9 Hz, 1H), 0.73 (ddd, *J* = 8.6, 5.6, 4.7 Hz, 1H) ppm; **^{13}C NMR (100 MHz, Chloroform-*d*):** δ 157.5, 142.4, 135.7, 128.5, 128.3, 126.8, 125.7, 113.7, 55.3, 36.5, 35.9, 22.6, 18.3, 15.6 ppm; **HRMS (ESI, m/z):** calculated for [M+H]+: 253.1592, found: 253.1585.

Procedure for 1o-1r Synthesis

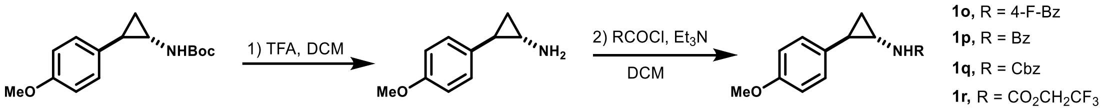

1) To a 150 mL oven-dried round-bottom flask equipped with a stir bar tert-butyl ((1S,2R)-2-(4-methoxyphenyl)cyclopropyl)carbamate[6] (2.0 g, 7.6 mmol, 1.0 equiv.), DCM (10 mL) and TFA (4.3 g, 38.0 mmol, 5.0 equiv.) was added. The reaction was stirred at room temperature for 16 h. After the reaction reached completion as indicated by TLC, the reaction mixture was quenched with sat. $NaHCO_3$ (30 mL) and extracted with DCM (50 mL) three times. The combined organic layers were dried over Na_2SO_4 and filtered. After volatile removed under reduced pressure, the crude residue was purified by column chromatography (PE : EtOAc = 1 : 1) to afford the desired (1S,2R)-2-(4-methoxyphenyl)cyclopropan-1-amine (930 mg, 5.7 mmol, 75%).

2) To a 50 mL oven-dried round-bottom flask with a stir bar was added (1S,2R)-2-(4-methoxyphenyl)cyclopropan-1-amine (200 mg, 1.2 mmol, 1.0 equiv.), DCM (4 mL), Et_3N (0.5 mL, 3.6 mmol, 3.0 equiv.). Then corresponding benzoyl chloride (1.8 mmol, 1.5 equiv.) was added dropwise by syringe and the reaction mixture was stirred at this temperature for 16 h. After the reaction reached completion as indicated by TLC, the reaction mixture was quenched with sat. NH_4Cl (30 mL) and extracted with DCM (50mL) three times. The combined organic layers were washed with water (30 mL) and brine (30 mL) successively, dried over Na_2SO_4 and filtered. After volatile removed under reduced pressure, the crude residue was purified by column chromatography to afford the desired compound **1o-1r** (**1o** 90%**, 1p** 82%**, 1q** 75%**, 1r** 86%).

N-((1S,2R)-2-(4-methoxyphenyl)cyclopropyl)benzamide (1o)

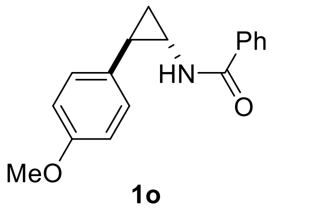

1o

^{1}H NMR (400 MHz, Chloroform-*d*): δ 7.76 (d, *J* = 7.3 Hz), 7.51 – 7.43 (m, 1H), 7.42 – 7.35 (m, 2H), 7.12 (d, *J* = 8.6 Hz), 6.81 (d, *J* = 8.6 Hz), 6.64 (br, 1H), 3.76 (s, 3H), 3.02 – 2.92 (m, 1H), 2.17 – 2.04 (m, 1H), 1.29 – 1.14 (m, 2H) ppm; **^{13}C NMR (100 MHz, Chloroform-*d*):** δ 168.8, 158.2, 134.3, 132.5, 131.7, 128.7, 128.0, 127.0, 113.9, 55.4, 32.3, 24.3, 15.9 ppm.

4-fluoro-N-((1S,2R)-2-(4-methoxyphenyl)cyclopropyl)benzamide (1p)

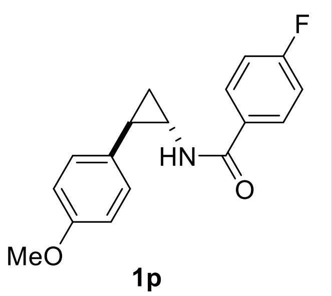

1p

^{1}H NMR (400 MHz, DMSO-*d6*): δ 7.97 – 7.84 (m, 2H), 7.27 – 7.21 (m, 2H), 7.07 – 7.01 (m, 2H), 6.83 – 6.76 (m, 2H), 5.23 (br, 1H), 4.45 (q, *J* = 8.5 Hz, 2H), 3.67 (s, 3H), 2.92 – 2.83 (m, 1H), 2.48 – 2.42 (m, 1H), 2.02 – 1.94 (m, 1H), 1.26 – 1.20 (m, 1H) ppm; **^{19}F NMR (377 MHz, DMSO-*d6*):** δ -109.44 (m) ppm; **^{13}C NMR (100 MHz, DMSO-*d6*):** 170.9, 158.0, 133.7, 132.62 (d, *J* = 9.5 Hz), 131.2, 130.34 (d, *J* = 8.9 Hz), 127.6, 115.65 (d, *J* = 21.7 Hz), 114.2, 55.5, 33.2 (d, *J* = 12.7 Hz), 23.7, 15.2 ppm.

benzyl ((1S,2R)-2-(4-methoxyphenyl)cyclopropyl)carbamate (1q)

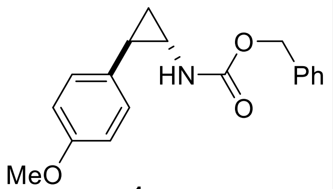

1q

^{1}H NMR (400 MHz, Chloroform-*d*): δ 7.43 – 7.28 (m, 5H), 7.17 – 6.88 (m, 2H), 6.87 – 6.69 (m, 2H), 5.32 – 5.00 (m, 3H), 3.77 (s, 3H), 2.77 – 2.54 (m, 1H), 2.09 – 1.95 (m, 1H), 1.20 – 1.00 (m, 2H) ppm; **^{13}C NMR (100 MHz, Chloroform-*d*):** δ 158.1, 141.1, 136.5, 132.5, 128.6, 128.3, 128.0, 127.7, 127.1, 113.9, 65.3, 55.4, 32.4, 24.4, 15.7 ppm.

2,2,2-trifluoroethyl ((1S,2R)-2-(4-methoxyphenyl)cyclopropyl)carbamate (1r)

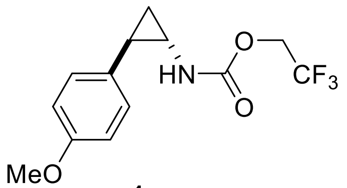

1r

^{1}H NMR (400 MHz, Chloroform-*d*): δ 7.14 – 6.99 (m, 2H), 6.85 – 6.76 (m, 2H), 5.23 (br, 1H), 4.45 (q, *J* = 8.5 Hz, 2H), 3.77 (s, 3H), 2.74 – 2.62 (m, 1H), 2.12 – 2.00 (m, 1H), 1.22 – 1.06 (m, 2H) ppm; **^{19}F NMR (377 MHz, Chloroform-*d*):** δ -74.17 (t, *J* = 8.4 Hz) ppm.

Procedure for 1ab Synthesis

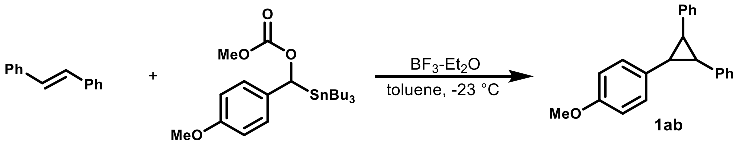

To a 50 mL oven-dried round-bottom flask equipped with a stir bar was added the solution of (4-methoxyphenyl)(tributylstannyl)methyl methyl carbonate[9] (486 mg, 1.0 mmol, 1.0 equiv.) and the (E)-1,2-diphenylethene (180 mg, 1.0 mmol, 1.0 equiv.) in toluene (3.5 mL) under nitrogen atmosphere at room temperature. The reaction vessel was cooled to -23 °C. BF_3-Et_2O (156 mg, 1.1 mmol, 1.1 equiv.) was added by syringe and stirred at the same temperature for 2 h. After the reaction reached completion as indicated by TLC, the reaction mixture was quenched with sat. $NaHCO_3$ (10 mL) and extracted with EtOAc (30 mL) three times. The combined organic layers were washed with brine (20 mL), dried over Na_2SO_4 and filtered. After volatile removed under reduced pressure, the crude residue was purified by column chromatography (PE : EtOAc = 50 : 1 to 10 : 1) to afford (3-(4-methoxyphenyl)cyclopropane-1,2-diyl)dibenzene **1ab** (243 mg, 0.81mmol, 81%) as a white solid.

(3-(4-methoxyphenyl)cyclopropane-1,2-diyl)dibenzene (1ab)

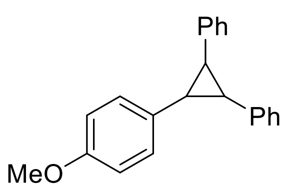

1ab

^{1}H NMR (400 MHz, Chloroform-*d*): δ 7.55 – 6.99 (m, 12H), 6.90 – 6.73 (m, 2H), 3.81 (s, 3H), 2.99 – 2.81 (m, 3H) ppm; **^{13}C NMR (100 MHz, Chloroform-*d*):** δ 158.1, 142.4, 138.2, 130.4, 129.8, 129.1, 128.8, 128.1, 126.6, 126.3, 126.1, 113.6, 34.6, 34.1, 31.1 ppm.

Procedure for 1ad Synthesis

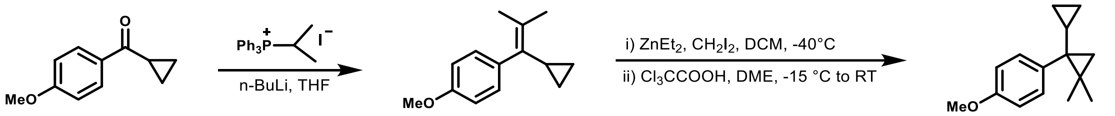

1ad

a) To a 150 mL oven-dried round-bottom flask equipped with a stir bar, the isopropyltriphenylphosphonium iodide (4.9 g, 11.35 mmol, 2.0 equiv.) and anhydrous THF (35 mL) was added. The reaction flask was capped with rubber septum and charged with N_2 balloon and then the reaction mixture was cooled to 0 °C. *n*-BuLi (2.5 M, 4.6 mL, 11.35 mmol, 2.0 equiv.) was added dropwise by syringe and the reaction mixture was stirred at this temperature for 1 h. A solution of cyclopropyl(4-methoxyphenyl)methanone (1.0 g, 5.67 mmol, 1.0 equiv.) in THF (5 mL) was then added by syringe and the reaction mixture was allowed to warm to room temperature, and then stirred at 40 °C for 16 h. After the reaction reached completion as indicated by TLC, the reaction mixture was quenched by sat. NH_4Cl (30 mL) and extracted with EtOAc (100mL) three times. The combined organic layers were washed with H_2O_2 (10 wt% in water, 10 mL) and brine (20 mL), dried over Na_2SO_4, and filtered. After volatile removed under reduced pressure, the crude residue was purified by column chromatography (PE : EtOAc = 50 : 1 to 10 : 1) to afford the 1-(1-cyclopropyl-2-methylprop-1-en-1-yl)-4-methoxybenzene (883 mg, 4.36 mmol, 77%).

b) To a 50 mL oven-dried round-bottom flask equipped with a stir bar was added DCM (3 mL, 0.67 M) under nitrogen atmosphere and was cooled to -40 °C. $ZnEt_2$ (2.0 M, 2.5 mL, 5.0 mmol, 2.5 equiv.) was added followed by slow addition of a solution of CH_2I_2

(2.68 g, 10.0 mmol, 5.0 equiv.) in DCM (1 mL) by syringe. The reaction mixture was stirred at the same temperature for 1 h and then warmed to -10 °C. Next, a solution of trichloroacetic acid (60 mg, 0.4 mmol, 0.2 equiv.) and DME (224 mg, 2.4 mmol, 1.2 equiv.) in DCM (1 mL) was added dropwise into the reaction mixture by syringe and the resulting solution was allowed to stir at -10 °C for another 1 h. A solution of 1-(1-cyclopropyl-2-methylprop-1-en-1-yl)-4-methoxybenzene (400 mg, 2.0 mmol, 1.0 equiv.) in DCM (1 mL) was then added by syringe and the reaction mixture was allowed to warmed to room temperature and stirred for 16 h. After the reaction reached completion as judged by ^{1}H-NMR, the reaction mixture was quenched with sat. NH_4Cl (30 mL) and extracted with DCM (20 mL) three times. The combined organic layers were washed with aq. NaOH (1.0 M, 30 mL) and brine (20 mL), dried over Na_2SO_4 and filtered. After volatile removed under reduced pressure, the crude residue was purified by column chromatography (PE : EtOAc = 50 : 1 to 10 : 1) to afford the 1-(cyclopropyl(2,2-dimethylcyclopropyl)methyl)-4-methoxybenzene **1ad** (322 mg, 1.40 mmol, 70%).

1-(4-methoxyphenyl)-2,2-dimethyl-1,1'-bi(cyclopropane) (1ad)

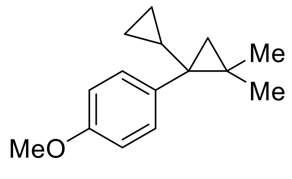

1ad

^{1}H NMR (400 MHz, Chloroform-*d*): δ 7.10 – 7.02 (m, 2H), 6.83 – 6.77 (m, 2H), 3.78 (s, 3H), 1.31 (s, 3H), 1.20 – 1.10 (m, 1H), 0.75 (s, 3H), 0.55 (d, *J* = 4.6 Hz), 0.49 – 0.41 (m, 1H), 0.40 (d, *J* = 4.7 Hz), 0.38 – 0.29 (m, 1H), 0.18 – 0.10 (m, 1H), 0.05 – -0.02 (m, 1H) ppm; **^{13}C NMR (100 MHz, Chloroform-*d*):** δ 157.5, 137.2, 130.5, 113.2, 35.1, 25.3, 21.8, 21.8, 21.6, 16.3, 6.6, 3.9.

Procedure for 1ac and 1af Synthesis

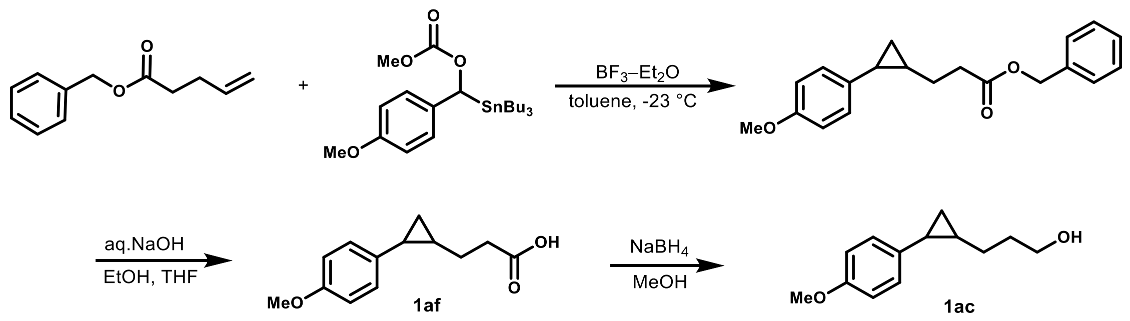

a) To a 50 mL oven-dried round-bottom flask equipped with a stir bar was added a solution of (4-methoxyphenyl)(tributylstannyl)methyl methyl carbonate (486 mg, 1.0 mmol, 1.0 equiv.) and benzyl pent-4-enoate (1.0 mmol, 1.0 equiv.) in toluene (3.5 mL) under nitrogen atmosphere at room temperature. The reaction vessel was cooled to -23 °C. BF_3-Et_2O (156 mg, 1.1 mmol, 1.1 equiv.) was added by syringe and stirred at the same temperature for 2 h. After the reaction reached completion as indicated by TLC, the reaction mixture was quenched with sat. $NaHCO_3$ (10 mL) and extracted with EtOAc (30 mL) three times. The combined organic layers were washed with brine (20 mL), dried over Na_2SO_4 and filtered. After volatile removed under reduced pressure, the crude residue was purified by column chromatography (PE : EtOAc = 50 : 1 to 10 : 1) to afford the benzyl 3-(2-(4-methoxyphenyl)cyclopropyl)propanoate (223.5 mg, 0.72mmol, 72%).

b) To a 50 mL round-bottom flask equipped with a stir bar was added benzyl 3-(2-(4-methoxyphenyl)cyclopropyl)propanoate (223.5 mg, 0.72 mmol, 1.0 equiv.), EtOH (2 mL), THF (5 mL) and 2M NaOH aqueous solution (3.6 mL, 7.2 mmol, 10 equiv.). The reaction vessel was stirred at room temperature for 16 h. After the reaction reached completion as indicated by TLC, the reaction mixture was quenched with 2M HCl (5 mL) and extracted with EtOAc (30 mL) three times. The combined organic layers were washed with brine (20 mL), dried over Na_2SO_4 and filtered. After volatile removed under reduced pressure, the crude residue was purified by column chromatography (PE : EtOAc = 1 : 1 to EA 100%) to afford 3-(2-(4-methoxyphenyl)cyclopropyl)propanoic acid **1af** (143 mg, 0.65 mmol, 90%).

3-(2-(4-methoxyphenyl)cyclopropyl)propanoic acid (1af)

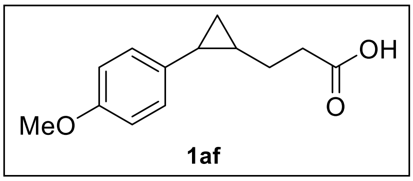

^{1}H NMR (400 MHz, Chloroform-*d*): δ 10.21 (br, 1H), 7.10 (d, *J* = 8.7 Hz, 2H), 6.81 (d, *J* = 8.7 Hz, 2H), 3.77 (s, 3H), 2.28 (t, *J* = 7.3 Hz, 2H), 2.13 – 2.04 (m, 1H), 1.49 – 1.38 (m, 1H), 1.26 – 1.19 (m, 1H), 1.13 – 1.02 (m, 1H), 0.98 – 0.90 (m, 1H), 0.61 (q, *J* = 5.5 Hz, 1H). **^{13}C NMR (100 MHz, Chloroform-*d*):** δ 180.3, 157.9, 130.8, 130.1, 113.6, 55.3, 33.9, 24.2, 20.3, 17.9, 9.5 ppm.

c) To a 50 mL round-bottom flask equipped with a stir bar was added 3-(2-(4-methoxyphenyl)cyclopropyl)propanoic acid **1af** (100 mg, 0.45 mmol, 1.0 equiv.) and MeOH (10 mL). Then $NaBH_4$ (43 mg, 1.13 mmol, 2.5 equiv.) was added with stirring. The reaction vessel was then heated to reflux for 6 h. After the reaction reached completion as indicated by TLC, the reaction mixture was cooled to room temperature and quenched with 1M HCl (5 mL). The volatile was removed under reduced pressure and the residue was extracted with EtOAc (30 mL) three times. The combined organic layers were washed with brine (20 mL), dried over Na_2SO_4 and filtered. After volatile removed under reduced pressure, the crude residue was purified by column chromatography (PE : EtOAc = 4 : 1 to 1 : 1) to afford 3-(2-(4-methoxyphenyl)cyclopropyl)propan-1-ol **1ac** (74 mg, 0.36 mmol, 80%).

3-(2-(4-methoxyphenyl)cyclopropyl)propan-1-ol (1ac)

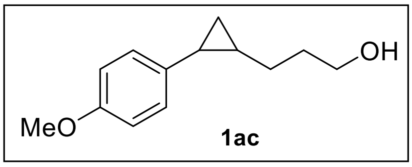

^{1}H NMR (400 MHz, Chloroform-*d*): δ 7.09 (d, *J* = 8.5 Hz, 2H), 6.80 (d, *J* = 8.6 Hz, 2H), 3.76 (s, 3H), 3.48 (t, *J* = 6.6 Hz, 2H), 2.10 – 1.99 (m, 1H), 1.62 – 1.45 (m, 2H), 1.21 – 1.10 (m, 1H), 1.05 – 0.88 (m, 3H), 0.56 (q, *J* = 5.3 Hz, 1H). **^{13}C NMR (100 MHz, Chloroform-*d*):** δ 157.7, 131.4, 130.0, 113.4, 62.7, 55.3, 32.5, 24.9, 20.3, 18.5, 9.7 ppm.

Synthesis of (1R, 2R)-1,2-diphenylcyclopropane 114

a) To an oven-dried 100mL round-bottom flask equipped with a stir bar, (E)-styrylboronic acid (0.75g, 5 mmol, 1.0 equiv.), (+)TMTA (1.05 g, 5 mmol, 1.0 equiv.)

and dry DCM (20mL) was added at room temperature. The reaction mixture was stirred for 2 h and then cooled to -78 °C. In a separate 100 mL flask, Et_2Zn (1.0 M, 15 mL,15 mmol, 3.0 equiv.) was dissolved in DCM (20 mL), cooled to –78 °C and treated dropwise with CH_2I_2 (1.0 ml, 12 mmol, 4.8 equiv.), then stirred vigorously for 10 min to generate the carbenoid (ineffective stirring due to precipitation of zinc salt or CH_2I_2 did not affect the reaction). The pre-chilled -78 °C solution was then quickly added *via syringe* over 2 min. The mixture was stirred at -78 °C for 8 h. 20 mL of saturated aqueous NH_4Cl solution was carefully added to quench the reaction. After addition of NH_4Cl, the mixture was stirred at -78 °C for 5 min, taken out of the cooling bath and warmed to ambient temperature. After phase separation,1M HCl was added to dissolve precipitate in the aqueous phase (pH was 5-6 at this point). The aqueous phase was extracted with 50 mL of DCM three times. The combined organic phases were dried with $MgSO_4$, filtered and concentrated and pumped to afford crude **((1R,2R)-2-phenylcyclopropyl)boronic acid** (directly used for the next step).

b) To an oven-dried 50 mL round-bottom flask equipped with a stir bar, crude **((1R,2R)-2-phenylcyclopropyl)boronic acid**, bromobenzene (720 mg, 4.5 mmol, 0.9 equiv.), K_3PO_4 (3.2 g, 15 mmol, 3.3 equiv.), $Pd(PPh_3)_4$ (156 mg, 0.03 equiv.) and toluene (20 mL) was added under nitrogen atmosphere. The reaction mixture was stirred at 100 °C for 16 h. Once the reaction was judged to be completed by TLC analysis, the reaction mixture was filtered to remove the solids and the volatile materials of the reaction mixture were removed under reduced pressure, the crude residue was purified by column chromatography (PE 100%) to obtain **(1S,2S)-1,2-diphenylcyclopropane 114**as a colorless oil (306 mg, 1.57 mmol, overall yield 35%, ee 90%).

2-((1R,2R)-2-phenylcyclopropyl)benzo[d]thiazole ((1R, 2R)-115) was prepared by a similar Suzuki coupling of ((1R,2R)-2-phenylcyclopropyl)boronic acid and 2-iodo-1,3-benzothiazole (22% overall yield, 90% ee).

2-((1R,2R)-2-phenylcyclopropyl)benzo[d]thiazole ((1R, 2R)-115)

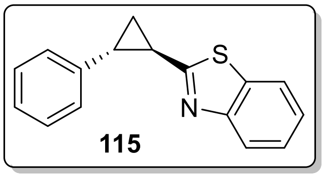

115

light yellow oil, **^{1}H NMR (400 MHz, Chloroform-*d*)** δ 7.92 (d, *J* = 8.1 Hz, 1H), 7.80 (d, *J* = 8.0 Hz, 1H), 7.47 – 7.41 (m, 1H), 7.36 – 7.28 (m, 3H), 7.26 – 7.14 (m, 3H), 2.79 – 2.71 (m, 1H), 2.65 - 2.57 (m, 1H), 2.00 – 1.91 (m, 1H), 1.74 – 1.67 (m, 1H). **^{13}C NMR (100 MHz, Chloroform-*d*)** δ 172.5, 153.4, 140.6, 134.4, 128.7, 126.6, 126.2, 126.1, 124.5, 122.3, 121.5, 30.1, 26.7, 20.4. **HRMS (ESI, m/z)**: calculated for $[M+H]^+$: 252.0847, found: 252.0854.

Preparation of ((1S,2S)-2-(4-methoxyphenyl)cyclopropyl)methanol (117)[7]

To a solution of 2.2 mL (2.2 mmol, 2.2 equiv.) of diethylzinc in 5.0 mL of anhydrous CH_2Cl_2 at 0 °C was added 0.36 mL (4.4 mmol, 4.4 equiv.) of freshly distilled diiodomethane. The mixture is stirred at 0 °C for 10 min (white precipitate formed) and a solution of 297 mg (1.1 mmol, 1.1 equiv.) of chiral dioxaborolane and 164 mg (1.0 mmol, 1.0 equiv.) of (*E*)-3-(4-methoxyphenyl)prop-2-en-1-ol in 7 mL of anhydrous CH_2Cl_2 was added rapidly via a cannula. The resulting mixture was stirred at room temperature for 2 h and then cooled to 0 °C. Saturated aqueous NH_4Cl was added and the mixture was washed with three portions of ethyl acetate. The combined organic layers were washed with sat. NaCl, dried over $MgSO_4$, and concentrated under reduced pressure. The residue was purified by column chromatography (PE / EtOAc = 4:1) to afford **117** as a yellow oil which solidified upon storage (160 mg, 90% yield, 90% ee).

((1S,2S)-2-(4-methoxyphenyl)cyclopropyl)methanol (117)

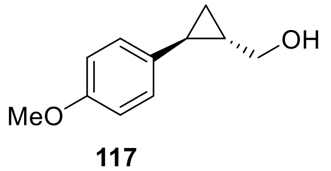

117

^{1}H NMR (400 MHz, Chloroform-*d*) δ 7.08 – 6.92 (m, 2H), 6.87 – 6.68 (m, 2H), 3.77 (s, 3H), 3.63 – 3.51 (m, 2H), 1.85 – 1.71 (m, 1H), 1.50 – 1.31 (m, 1H), 0.95 – 0.76 (m, 2H). **^{13}C NMR (100 MHz, Chloroform-*d*)** δ 157.8, 134.6, 127.1, 113.9, 76.3, 58.5, 55.4, 22.0, 20.8, 13.5.

Procedure for 2O4 Synthesis

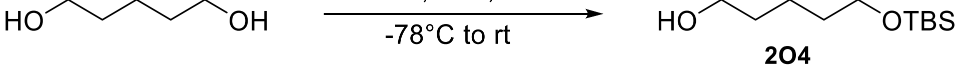

5-((tert-butyldimethylsilyl)oxy)pentan-1-ol **2O4** was prepared according to the literature procedure[10]: To a solution of 1,

5-pentanediol (520 mg, 5.0 mmol) in THF (8 mL, 0.62 M) at -78 °C under argon was added n-BuLi (2.1 mL, 2.4 M in hexanes, 5.0 mmol), and the resulting solution was stirred for 30 min at -78 °C. A solution of TBSCl (750 mg, 5 mmol) in THF (2 mL) was added rapidly, and the resulting mixture was stirred at -78 °C for 10 min, and then warmed to room temperature and stirred for 3 h. The reaction was diluted with water and extracted with EtOAc. The aqueous phase was extracted with EtOAc and the combined organic layers were washed with brine and dried over $MgSO_4$. The solvent was removed via rotary evaporation and residue was purified by column chromatography on silica gel (PE/EtOAc = 5:1) to give **2O4** as a colorless oil in 68% yield. **^{1}H NMR (400 MHz, Chloroform-*d*)** δ 3.68 – 3.54 (m, 4H), 1.62 – 1.46 (m, 4H), 1.43 – 1.31 (m, 2H), 0.86 (s, 9H), 0.02 (s, 6H). **^{13}C NMR (100 MHz, Chloroform-*d*)** δ 63.2, 62.9, 32.5, 32.5, 26.0, 22.1, 18.5, -5.2.

Procedure for 2N6 Synthesis[11]

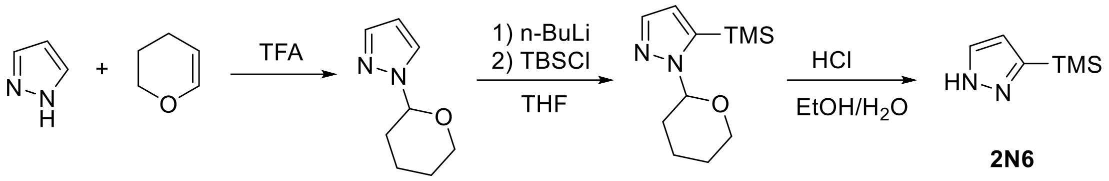

2N6

To a solution of pyrazole (340 mg, 5 mmol) in 3,4-dihydro-2H-pyran (547 mg, 6.5 mmol, 1.3 equiv.) was added 0.1 mL TFA, and the mixture was stirred at 60 °C for 2 h. After pyrazole was consumed as indicated by TLC analysis, water was added and the resulting mixture was extracted with EtOAc. The combined organic layers were washed with brine and dried over $MgSO_4$. Solvents were removed under reduced pressure and the residue was purified by column chromatography on silica gel (50% EtOAc/PE) to afford the protected pyrazole as an oil (647 mg, 85%).

A solution of the product obtained in the last step (4.25 mmol) in THF (5 mL) was cooled to –35 °C. To this solution n-BuLi (2.1 mL, 2.4 M in hexane, 5.1 mmol, 1.2

equiv) was added dropwise and the mixture was stirred for 1 h at the same temperature. A solution of TMSCl (705 mg, 4.67 mmol, 1.1 equiv.) in THF (1 mL) was added dropwise to the resulting mixture, which was then warmed to room temperature and stirred for another 2.5 h. Saturated NH_4Cl solution was then added and the aqueous phase was extracted with EtOAc. The combined organic layers were washed with brine and dried over $MgSO_4$. Solvents were removed under reduced pressure and the residue was purified by column chromatography on silica gel (40% EtOAc/PE) to afforded the TBS substituted pyrazole (839 mg, 88% yield) as a colorless oil.

A solution of the TBS substituted pyrazole (839 mg, 3.74 mmol) in 1 mL of EtOH, 1 mL of water, and 1 mL of conc. HCl was stirred at 50 °C for 3 h. After the reaction was completed, saturated $NaHCO_3$ was added to neutralize the acid, the aqueous phase was extracted with EtOAc. The combined organic layers were washed with brine, dried over $MgSO_4$. Solvents were removed under reduced pressure and the residue was purified by column chromatography on silica gel (40% EtOAc/PE) afforded 3-(trimethylsilyl)-1H-pyrazole **2N6** (472 mg, 90% yield) as a white solid.

^{1}H NMR (400 MHz, Chloroform-*d*) δ 7.62 (d, *J* = 2.2 Hz, 1H), 6.48 (d, *J* = 2.0 Hz, 1H), 0.34 (s, 9H); **^{13}C NMR (100 MHz, Chloroform-*d*)** δ 142.6, 139.0, 112.2, -0.9.

Reaction Condition Optimization

Supplementary Table 1: Photocatalyst screening[a]

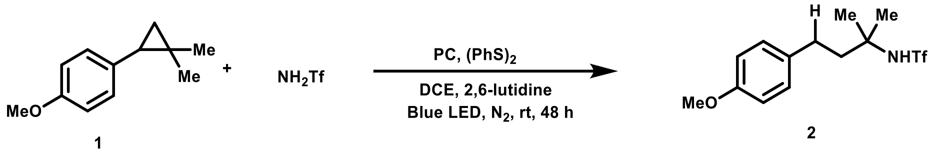

1 2

Entry	PC	Yield of 2 (%)	Conversion (%)
1	PC-I	38	45
2	PC-II	25	44
3	PC-III	47	68
4	PC-IV	40	69
5	PC-V	73	100
6	PC-VI	68	90
7	PC-VII	63	100
8	PC-VIII	77	100
9	PC-IX	NR	0
10	PC-X	NR	10
11	PC-XI	trace	10
16 [b]	PC- VIII	NR	0
17 [c]	-	NR	0
18[d]	PC- VIII	44	63
19[e]	PC- VIII	86	100

Experiments were performed with **1** (0.1 mmol), $TfNH_2$ (0.15 mmol), photocatalyst (2 mol%), $(PhS)_2$ (20 mol%), 2,6-lutidine (25 mol%) in DCE (0.5 mL), irradiating with 15 W blue LEDs under N_2 atmosphere at room temperature for 48 h. Yield and conversion were determined by ^1H NMR using 1,1,2,2-tetrachloroethane as internal standard. NR, no reaction. NDP, no desired product. [b] Without irradiation. [c] Without photocatalyst. [d] Without 2,6-lutidine. [e] 2,6-ditBu-Py (25 mol%) instead of 2,6-lutidine.

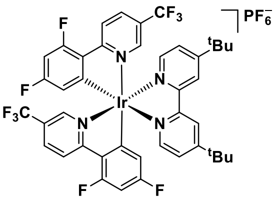

PC-I

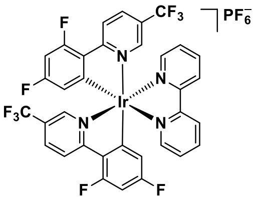

PC-II

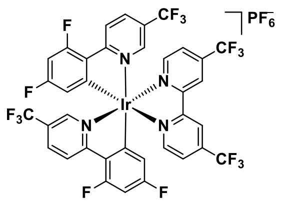

PC III

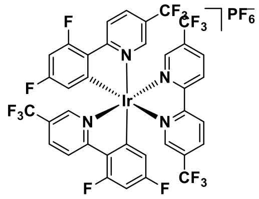

PC-IV

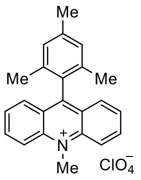

PC-V

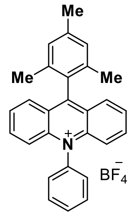

PC VI

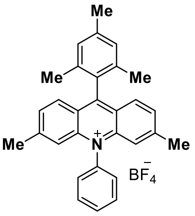

PC-VII

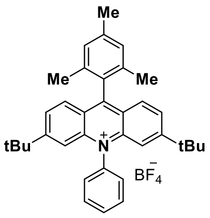

PC VIII

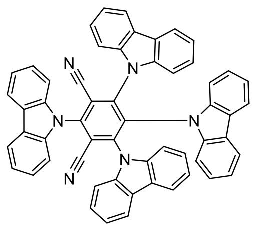

PC IX

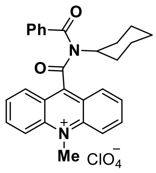

PC-X

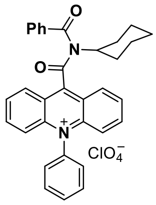

PC-XI

Supplementary Table 2: HAT reagent screening[a]

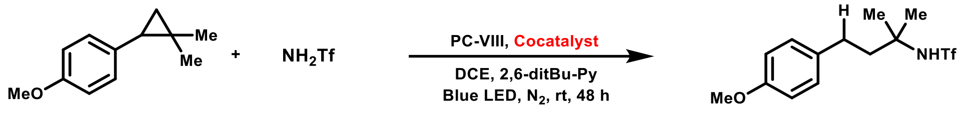

Entry	HAT reagent	Yield of 2 (%)	Conversion (%)
1	HAT reagent-I	87	100
2	HAT reagent-II	85	100
3	HAT reagent-III	14	27
4	HAT reagent-IV	67	78
5	HAT reagent-V	9	25
6	HAT reagent-VI	70	90
7	HAT reagent-VII	41	55
8	HAT reagent-VIII	81	100
9	HAT reagent-IX	86	96
10	HAT reagent-X	12	22
11	HAT reagent-XI	82	99
12[b]	HAT reagent-I	55	70
13[c]	HAT reagent-I	77	90
14[d]	HAT reagent-I	83	95
15[e]	-	0	<5

[a] Experiments were performed with **1** (0.1 mmol), $TfNH_2$ (0.15 mmol), photocatalyst PC-VIII (2 mol%), HAT reagent (20 mol%), 2,6-ditBu-Py (25 mol%) in DCE (0.5 mL), irradiating with 15 W blue LEDs under N_2 atmosphere at room temperature for 48 h. Yield and conversion were determined by ^1H NMR using 1,1,2,2-tetrachloroethane as internal standard. NR, no reaction. NDP, no desired product. [b] $(PhS)_2$ (10 mol%). [c] $(PhS)_2$ (50 mol%). [d] $(PhS)_2$ (100 mol%). [e] without HAT reagent.

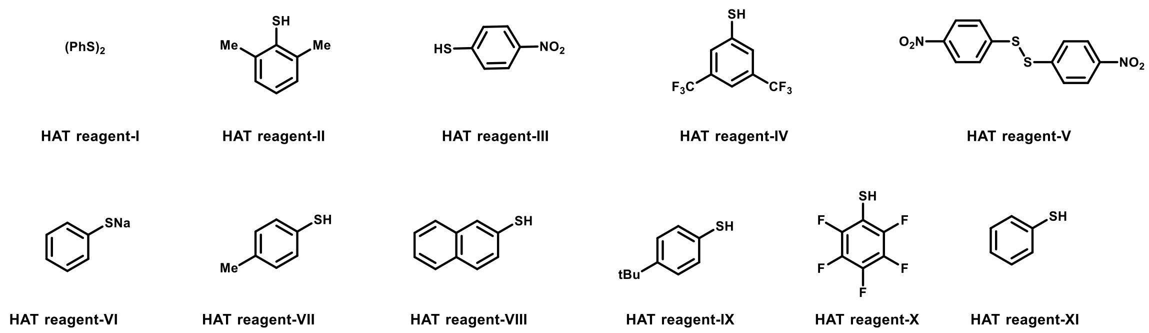

Supplementary Table 3: Solvent screening[a]

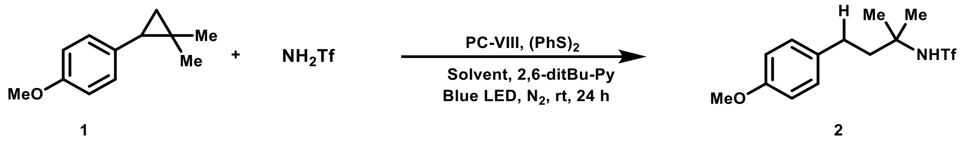

Entry	solvent	Yield of 2 (%)	Conversion (%)
1	DCE	85	100
2	DMF	35	48
3	THF	21	37
4	MeCN	NR	0

[a] Experiments were performed with **1** (0.1 mmol), $TfNH_2$ (0.15 mmol), PC-VIII (2 mol%), $(PhS)_2$ (20 mol%), 2,6-ditBu-Py (25 mol%) in Solvent (0.5 mL), irradiating with 15 W blue LEDs under N_2 atmosphere at room temperature for 24 h. Yield and conversion were determined by ^1H NMR using 1,1,2,2-tetrachloroethane as internal standard. NR, no reaction.

Supplementary Table 4: Light source screening[a]

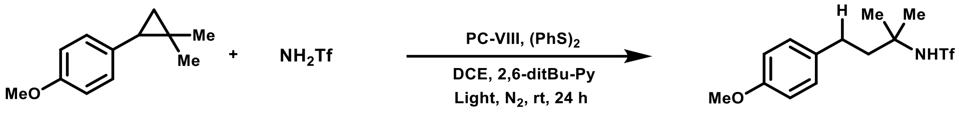

1
2

Entry	Light source	Yield of 2 (%)	Conversion (%)
1	bule-LEDs	85	100
2	green-LEDs	1.5	5
3	white-LEDs	25	40
4	dark	0	NR

[a] Experiments were performed with **1** (0.1 mmol), $TfNH_2$ (0.15 mmol), PC-VIII (2 mol%), $(PhS)_2$ (20 mol%), 2,6-ditBu-Py (25 mol%) in DCE (0.5 mL), irradiating with 15 W LEDs under N_2 atmosphere at room temperature for 24 h. Yield and conversion were determined by ^1H NMR using 1,1,2,2-tetrachloroethane as internal standard. NR, no reaction.

Supplementary Table 5: Screening of reaction atmosphere[a]

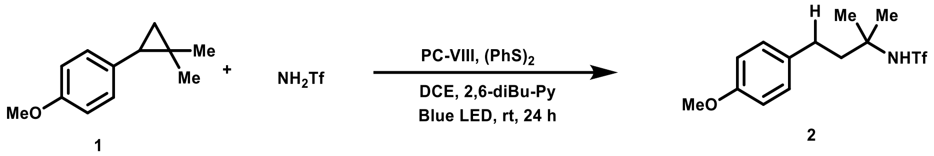

Entry	Atmosphere	Yield of 2 (%)	Conversion (%)
1	N_2	87	100
2	air	NDP	70

[a] Experiments were performed with **1** (0.1 mmol), $TfNH_2$ (0.15 mmol), PC-VIII (2 mol%), $(PhS)_2$ (20 mol%), 2,6-ditBu-Py (25 mol%) in DCE (0.5 mL), irradiating with 15 W blue LEDs under N_2 or air atmosphere at room temperature for 24 h. Yield and conversion were determined by ^1H NMR using 1,1,2,2-tetrachloroethane as internal standard. NDP, no desired product.

Supplementary Table 6: Screening photocatalyst loading[a]

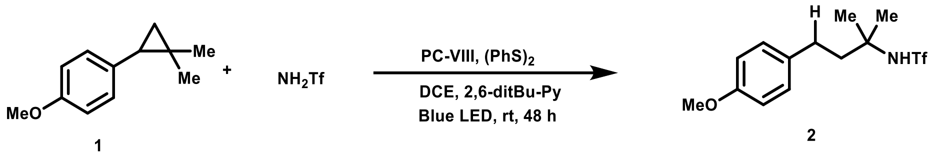

Entry	mol%	Yield of 2 (%)	Conversion (%)
1	0.5	72	88
2	1.0	77	95
3	2.0	86	100
4	5.0	91	100

[a] Experiments were performed with **1** (0.1 mmol), $TfNH_2$ (0.15 mmol), PC-VIII, $(PhS)_2$ (20 mol%), 2,6-ditBu-Py (25 mol%) in DCE (0.5 mL), irradiating with 15 W blue LEDs under N_2 atmosphere at room temperature for 48 h. Yield and conversion were determined by ^1H NMR using 1,1,2,2-tetrachloroethane as internal standard.

Supplementary Table 7: Optimization of reaction concentration and time[a]

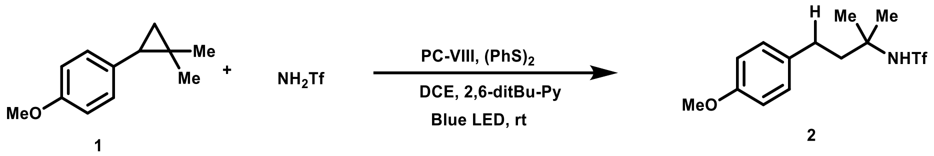

Entry	Concentration (M)	Time (h)	Yield of 2 (%)	Conversion (%)
1	0.1	24	78	90
2	0.2	24	88	100
3	0.5	24	72	90
4	0.2	48	93	100
5	0.2	72	92	100
6	0.2	16	58	68

[a] Experiments were performed with **1** (0.1 mmol), $TfNH_2$ (0.15 mmol), PC-VIII (5 mol%), $(PhS)_2$ (20 mol%), 2,6-ditBu-Py (25 mol%) in DCE (0.5 mL), irradiating with 15 W blue LEDs under N_2 atmosphere at room temperature. Yield and conversion were determined by ^1H NMR using 1,1,2,2-tetrachloroethane as internal standard.

Supplementary Table 8: HAT reagent screening for etherification [a]

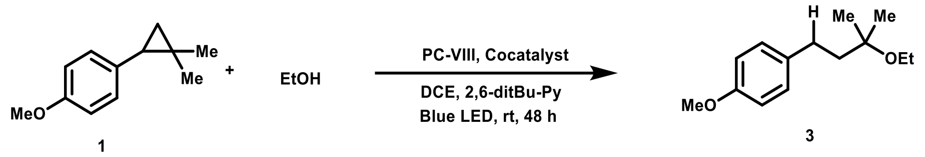

Entry	Cocatalyst	Yield of 3 (%)	Conversion (%)
1	HAT reagent-I	92	100
2	HAT reagent-II	45	55
3	HAT reagent-III	NR	0
4	HAT reagent-IV	NR	0
5	HAT reagent-V	NR	0
6	HAT reagent-VI	NR	0
7[b]	HAT reagent-I	91	100
8[c]	HAT reagent-I	77	85

[a] Experiments were performed with **1** (0.1 mmol), EtOH (1.0 mmol), PC-VIII (2 mol%), HAT reagent (20 mol%), 2,6-ditBu-Py (25 mol%) in DCE (0.5 mL), irradiating with 15 W blue LEDs under N_2 atmosphere at room temperature for 48 h. Yield and conversion were determined by ^1H NMR using 1,1,2,2-tetrachloroethane as internal standard. [b] $(PhS)_2$ (10 mol%). [c] $(PhS)_2$ (50 mol%).

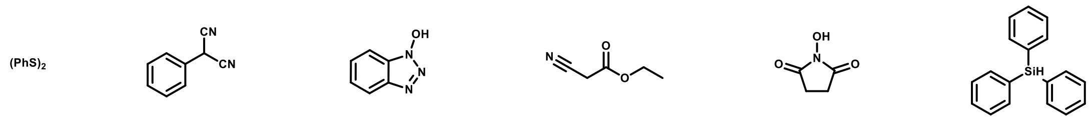

HAT reagent-I HAT reagent-II HAT reagent-III HAT reagent-IV HAT reagent-V HAT reagent-VI

General Procedure

General procedure A for hydrogen-amination of aryl cyclopropanes

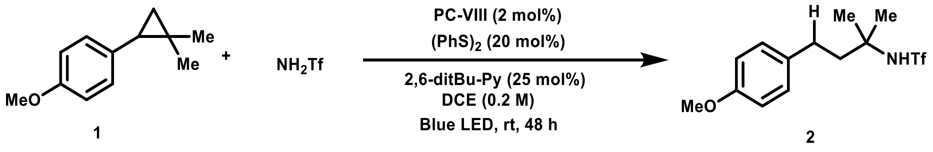

To an oven-dried 10 mL Schlenk tube equipped with a stir bar, $(PhS)_2$ (9.0 mg, 0.04 mmol, 20 mol%), $TfNH_2$ (44.7 mg, 0.3 mmol, 1.5 equiv.), photocatalyst (PC-VIII) (2.3 mg, 0.004 mmol, 2 mol%) were added. The Schlenk tube was purged with nitrogen three times, then 2,6-ditBu-Py (9.6 mg, 0.05 mmol, 25 mol%), **1** (35.2 mg, 0.2 mmol, 1.0 equiv.) and 1,2-dichloroethane (1.0 mL, 0.2 M) were added under nitrogen atmosphere. The Schlenk tube was sealed and irradiated with a 15W blue LED lamp (l = 459 nm) at room temperature for 48 h. When the reaction was determined to be completed by TLC analysis, the mixture was evaporated to dryness under reduced pressure and the crude residue was purified by column chromatography on silica gel (DCM : PE = 1 : 4 and then PE : EtOAc = 20 : 1 to 10 : 1) to afford the desired product **2**.

Procedure for deprotection of 1,1,1-trifluoromethanesulfonamide

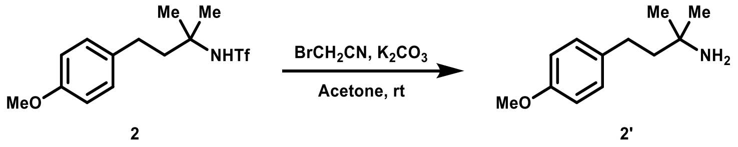

To a mixture of **2** (1.0 equiv.) and K_2CO_3 (1.2 equiv.) in acetone (0.25 M) was added bromoacetonitrile (1.2 equiv.). The reaction mixture was stirred at room temperature overnight, and the solvent was removed in vacuo thereafter. Water was added to quench the reaction, and the mixture was extracted with ethyl acetate, washed with brine, dried over Na_2SO_4, filtered, and concentrated in vacuo. The crude residue was purified by column chromatography on silica gel (PE : EtOAc = 5 : 1 to 1 : 1) to afford the desired product **2'** in 75% yield.

General Procedure B for hydrogen-etherification of aryl cyclopropanes

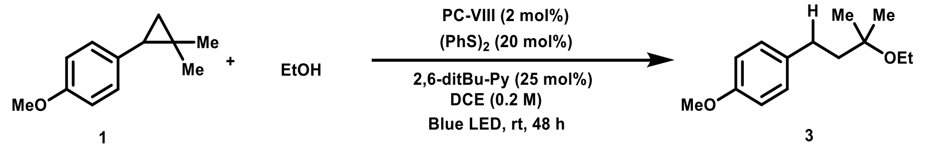

1 3

To an oven-dried 10 mL Schlenk tube equipped with a stir bar, $(PhS)_2$ (9.0 mg, 0.04 mmol, 20 mol%), photocatalyst (PC-VIII) (2.3 mg, 0.004 mmol, 2 mol%) were added. The Schlenk tube was purged with nitrogen three times, then EtOH (92 mg, 2.0 mmol, 10 equiv.), 2,6-ditBu-Py (9.6 mg, 0.05 mmol, 25 mol%), **1** (35.2 mg, 0.2 mmol, 1.0 equiv.), and 1,2-dichloroethane (1.0 mL, 0.2 M) were added under nitrogen atmosphere. The Schlenk tube was sealed and irradiated with a 15W blue LED lamp (l = 459 nm) at room temperature for 48 h. When the reaction was determined to be completed by TLC analysis, the mixture was evaporated to dryness under reduced pressure and the crude residue was purified by column chromatography on silica gel (DCM : PE = 1 : 4 and then PE : EtOAc = 20 : 1 to 10 : 1) to afford the desired product **3**.

General Procedure C for hydrogen-etherification of aryl cyclopropanes

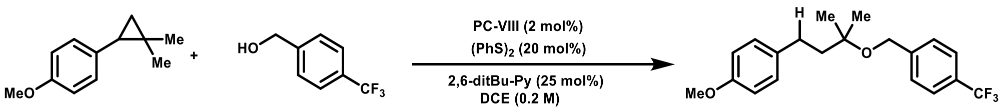

1 2O7 54

To an oven-dried 10 mL Schlenk tube equipped with a stir bar, $(PhS)_2$ (9.0 mg, 0.04 mmol, 20 mol%), photocatalyst (PC-VIII) (2.3 mg, 0.004 mmol, 2 mol%) and **2O7** (70.5 mg, 0.4 mmol, 2.0 equiv.) were added. The Schlenk tube was purged with nitrogen three times, then 2,6-ditBu-Py (9.6 mg, 0.05 mmol, 25 mol%), **1** (35.2 mg, 0.2 mmol, 1.0 equiv.), and 1,2-dichloroethane (1.0 mL, 0.2 M) were added under nitrogen atmosphere. The Schlenk tube was sealed and irradiated with a 15W blue LED lamp (l = 459 nm) at room temperature for 48 h. When the reaction was determined to be completed by TLC analysis, the mixture was evaporated to dryness under reduced pressure and the crude residue was purified by column chromatography on silica gel (DCM : PE = 1 : 4 and then PE : EtOAc = 20 : 1 to 10 : 1) to afford the desired product **54**.

Procedure for reaction of 1 with 2O18

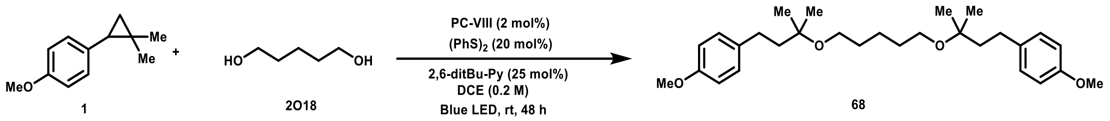

To an oven-dried 10 mL Schlenk tube equipped with a stir bar, $(PhS)_2$ (9.0 mg, 0.04 mmol, 20 mol%), photocatalyst (PC-VIII) (2.3 mg, 0.004 mmol, 2 mol%) and **2O18** (10.4 mg, 0.1 mmol, 0.5 equiv.) were added. The Schlenk tube was purged with nitrogen three times, then 2,6-ditBu-Py (9.6 mg, 0.05 mmol, 25 mol%), **1** (35.2 mg, 0.2 mmol, 1.0 equiv.), and 1,2-dichloroethane (1.0 mL, 0.2 M) were added under nitrogen atmosphere. The Schlenk tube was sealed and irradiated with a 15W blue LED lamp (l = 459 nm) at room temperature for 48 h. When the reaction was determined to be completed by TLC analysis, the mixture was evaporated to dryness under reduced pressure and the crude residue was purified by column chromatography on silica gel (DCM : PE = 1 : 4 and then PE : EtOAc = 20 : 1 to 10 : 1) to afford the desired product **68**.

General Procedure D for intramolecular hydrogen-etherification/esterification of aryl cyclopropanes

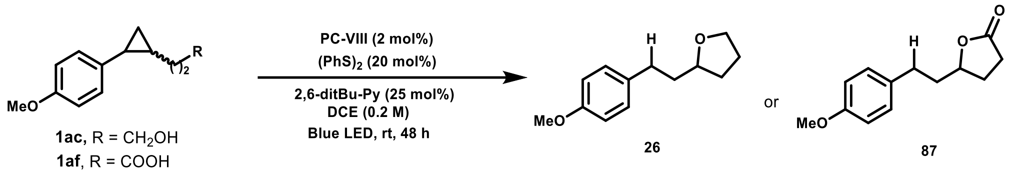

To an oven-dried 10 mL Schlenk tube equipped with a stir bar, $(PhS)_2$ (9.0 mg, 0.04 mmol, 20 mol%), photocatalyst (PC-VIII) (2.3 mg, 0.004 mmol, 2 mol%) were added. The Schlenk tube was purged with nitrogen three times, then 2,6-ditBu-Py (9.6 mg, 0.05 mmol, 25 mol%), **1ac** or **1af** (0.2 mmol, 1.0 equiv.), and 1,2-dichloroethane (1.0 mL, 0.2 M) were added under nitrogen atmosphere. The Schlenk tube was sealed and irradiated with a 15W blue LED lamp (l = 459 nm) at room temperature for 48 h. When

the reaction was determined to be completed by TLC analysis, the mixture was evaporated to dryness under reduced pressure and the crude residue was purified by column chromatography on silica gel (DCM : PE = 1 : 4 and then PE : EtOAc = 20 : 1 to 10 : 1) to afford the desired product **26** or **87**.

General Procedure E for hydrogen-esterification of aryl cyclopropanes

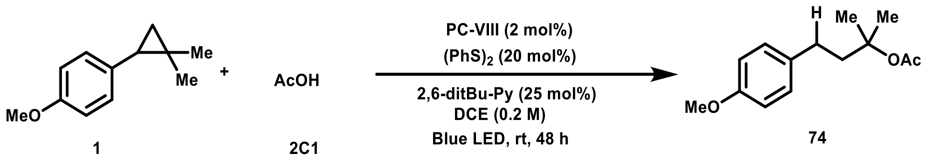

1 2C1 74

To an oven-dried 10 mL Schlenk tube equipped with a stir bar, $(PhS)_2$ (9.0 mg, 0.04 mmol, 20 mol%), photocatalyst (PC-VIII) (2.3 mg, 0.004 mmol, 2 mol%) were added. The Schlenk tube was purged with nitrogen three times, then AcOH **2C1** (24 mg, 0.4 mmol, 2.0 equiv.), 2,6-ditBu-Py (9.6 mg, 0.05 mmol, 25 mol%), **1** (35.2 mg, 0.2 mmol, 1.0 equiv.), and 1,2-dichloroethane (1.0 mL, 0.2 M) were added. The Schlenk tube was sealed and irradiated with a 15W blue LED lamp (l = 459 nm) at room temperature for 48 h. When the reaction was determined to be completed by TLC analysis, the mixture was evaporated to dryness under reduced pressure and the crude residue was purified by column chromatography on silica gel (DCM : PE = 1 : 4 and then PE : EtOAc = 20 : 1 to 10 : 1) to afford the desired product **74**.

General Procedure F for hydrogen-chlorination of aryl cyclopropanes

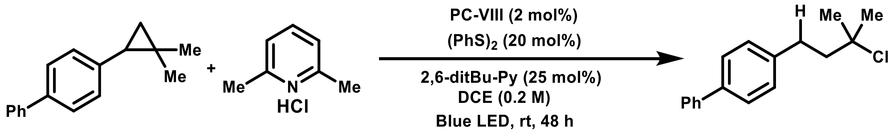

1e 2D 99

To an oven-dried 10 mL Schlenk tube equipped with a stir bar, $(PhS)_2$ (9.0 mg, 0.04 mmol, 20 mol%), photocatalyst (PC-VIII) (2.3 mg, 0.004 mmol, 2 mol%), 2,6-lutidine hydrochloride (57.4 mg, 0.4 mmol, 2.0 equiv.), **1e** (44.4 mg, 0.2 mmol, 1.0 equiv.) were added. The Schlenk tube was purged with nitrogen three times, then 2,6-ditBu-Py (9.6

mg, 0.05 mmol, 25 mol%), 1,2-dichloroethane (1.0 mL, 0.2 M) were added. The Schlenk tube was sealed and irradiated with a 15W blue LED lamp (l = 459 nm) at room temperature for 48 h. When the reaction was determined to be completed by TLC analysis, the mixture was evaporated to dryness under reduced pressure and the crude residue was purified by column chromatography on silica gel (PE : EtOAc = 100 : 1 to 50 : 1) to afford the desired product **99**.

General Procedure G for hydrogen-hydroxylation of aryl cyclopropanes

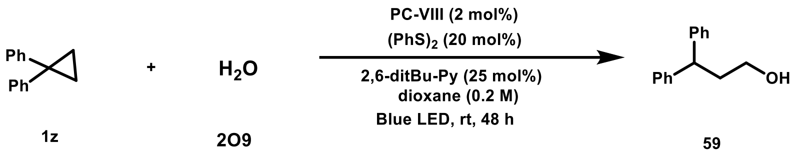

To an oven-dried 10 mL Schlenk tube equipped with a stir bar, $(PhS)_2$ (9.0 mg, 0.04 mmol, 20 mol%), photocatalyst (PC-VIII) (2.3 mg, 0.004 mmol, 2 mol%) were added. The Schlenk tube was purged with nitrogen three times, then **1z** (38.8 mg, 0.2 mmol, 1.0 equiv.), 2,6-ditBu-Py (9.6 mg, 0.05 mmol, 25 mol%), H_2O (36 mg, 2.0 mmol, 10.0 equiv.) and dioxane (1.0 mL, 0.2 M) were added under nitrogen atmosphere. The Schlenk tube was sealed and irradiated with a 15W blue LED lamp (l = 459 nm) at room temperature for 48 h. When the reaction was determined to be completed by TLC analysis, the mixture was evaporated to dryness directly under reduced pressure and the crude residue was purified by column chromatography on silica gel (PE : EtOAc = 10 : 1 to 5 : 1) to afford the desired product **59**.

General Procedure H for hydrogen-fluorination of aryl cyclopropanes

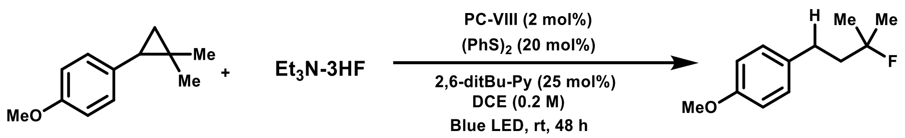

To an oven-dried 10 mL Schlenk tube equipped with a stir bar, $(PhS)_2$ (9.0 mg, 0.04 mmol, 20 mol%), photocatalyst (PC-VIII) (2.3 mg, 0.004 mmol, 2 mol%) were added. The Schlenk tube was purged with nitrogen three times, then **1** (35.2 mg, 0.2 mmol, 1.0 equiv.), 2,6-ditBu-Py (9.6 mg, 0.05 mmol, 25 mol%), triethylamine trihydrofluoride (64 mg, 0.4 mmol, 2.0 equiv.) and 1,2-dichloroethane (1.0 mL, 0.2 M) were added. The

Schlenk tube was sealed and irradiated with a 15W blue LED lamp (l = 459 nm) at room temperature for 48 h. When the reaction was determined to be completed by TLC analysis, the mixture was evaporated to dryness under reduced pressure and the crude residue was purified by column chromatography on silica gel (PE : EtOAc = 50 : 1 to 20 : 1) to afford the desired product **102**.

General Procedure I for hydrogen-azidation of aryl cyclopropanes

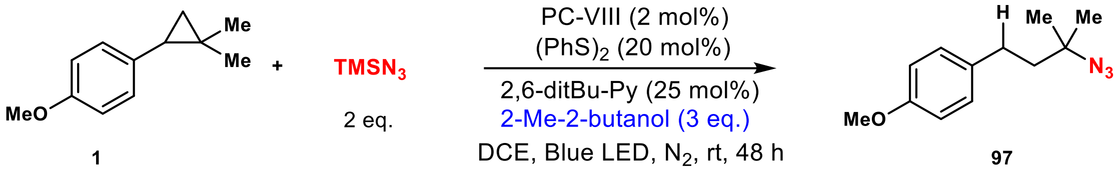

To an oven-dried 10 mL Schlenk tube equipped with a stir bar, $(PhS)_2$ (9.0 mg, 0.04 mmol, 20 mol%), photocatalyst (PC-VIII) (2.3 mg, 0.004 mmol, 2 mol%) were added. The Schlenk tube was purged with nitrogen three times, then **1** (35.2 mg, 0.2 mmol, 1.0 equiv.), 2,6-ditBu-Py (9.6 mg, 0.05 mmol, 25 mol%), $TMSN_3$ (46 mg, 0.4 mmol, 2.0 equiv.), 2-Methyl-2-butanol (52.9 mg. 0.6 mmol, 3.0 equiv.) and 1,2-dichloroethane (1.0 mL, 0.2 M) were added. The Schlenk tube was sealed and irradiated with a 15W blue LED lamp (l = 459 nm) at room temperature for 48 h. When the reaction was determined to be completed by TLC analysis, the mixture was evaporated to dryness under reduced pressure and the crude residue was purified by column chromatography on silica gel (PE : EtOAc = 50 : 1 to 20 : 1) to afford the desired product **97**.

General Procedure J for hydrogen-thiocyanation of aryl cyclopropanes

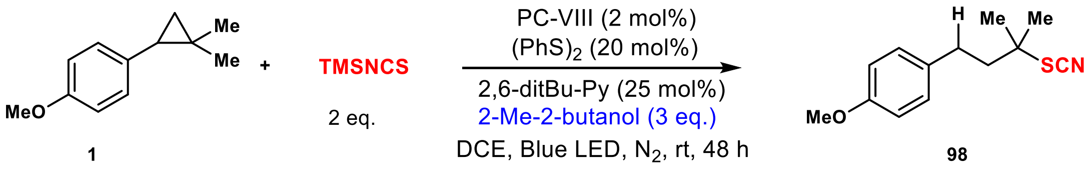

To an oven-dried 10 mL Schlenk tube equipped with a stir bar, $(PhS)_2$ (9.0 mg, 0.04 mmol, 20 mol%), photocatalyst (PC-VIII) (2.3 mg, 0.004 mmol, 2 mol%) were added. The Schlenk tube was purged with nitrogen three times, then **1** (35.2 mg, 0.2 mmol, 1.0 equiv.), 2,6-ditBu-Py (9.6 mg, 0.05 mmol, 25 mol%), TMSNCS (52.4 mg, 0.4 mmol, 2.0 equiv.), 2-Methyl-2-butanol (52.9 mg. 0.6 mmol, 3.0 equiv.) and 1,2-dichloroethane (1.0 mL, 0.2 M) were added. The Schlenk tube was sealed and irradiated with a 15W blue LED lamp (l = 459 nm) at room temperature for 48 h. When the reaction was determined to be completed by TLC analysis, the mixture was evaporated to dryness under reduced pressure and the crude residue was purified by column chromatography on silica gel (PE : EtOAc = 50 : 1 to 20 : 1) to afford the desired product **98**. The structure of **98** was assigned by comparing ^{13}C NMR of **98** with literature reports (*Org. Chem. Front.* **2021**, *8*, 3076; *Org. Lett.* **2021**, *23*, 4342; *Org. Chem. Front.* **2022**, *9*, 2963; *Org. Lett.* **2022**, *24*, 1742).

Supplementary Discussion

Light On/Off Experiment

To an over dried 5 mL NMR tube were added EtOH (1.0 mmol), **1** (0.1 mmol), PC-VIII (1.1 mg, 2 mol%), $(PhS)_2$ (0.02 mmol), 2,6-ditBu-Py (0.025 mmol), 1,1,2,2-tetrachloroethane as internal standard and $CDCl_3$ (1.0 mL, 0.2 M) in the glove box. The NMR tube was capped and the resulting mixture was subjected to alternating intervals of irradiation with blue light and dark. The reaction profile is shown below and the yield of product **3** as a function of time was determined by ^{1}H-NMR using 1,1,2,2-tetrachloroethane as internal standard. These results indicated that continuous irradiation with light was essential for the progress of this reaction.

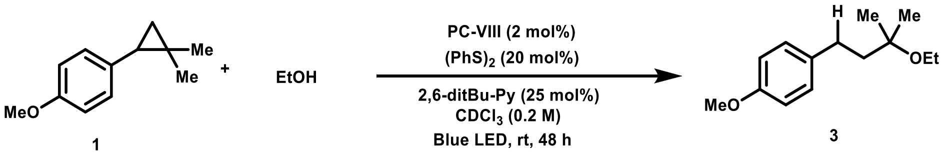

Supplementary Figure 1. Light on/off experiment of **1** with EtOH

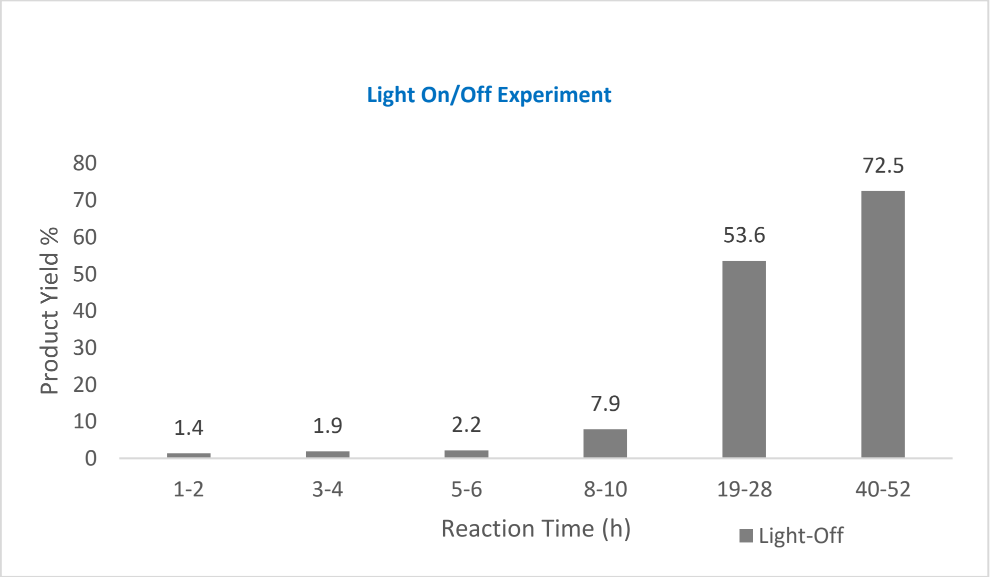

Determination of the Reaction Quantum Yield (Φ)

Emission spectrum of blue LED used for quantum yield experiments (λ_{max}= 459 nm).

Recorded using a F-4600 FL Spectrophotometer.

Determination of the reaction quantum yield

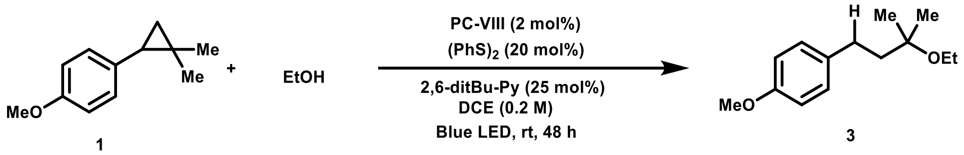

To a 1 mL quartz cuvette with two sides taped over with electrical tape, **1** (21 mg, 0.12 mmol, 1.0 equiv.), EtOH (55 mg, 1.2 mmol, 10 equiv.), PC-VIII (1.4 mg, 2 mol%), $(PhS)_2$ (0.024 mmol, 20 mol%), 2,6-ditBu-Py (0.03 mmol, 25 mol%), anhydrous DCE (0.6 mL, 0.2 M), and a small stir bar were added in the glove box, then the quartz cuvette was capped. The sample was stirred and irradiated for 18000 s (5.0 h) at λ_{max} = 459 nm at rt under N_2 atmosphere. After irradiation, the yield of product **3** was determined to be 62.0% (7.44×10^{-5} mol of **3**) by ^{1}H NMR integration with 1,1,2,2-tetrachloroethane as internal standard. The reaction quantum yield (Φ) was determined by using eq 2 where the photon flux for this light source had been previously determined to be 2.351×10^{-8} einstein s^{-1} using potassium ferrioxalate as a chemical actinometer (see reference for details) [6]. t is the reaction time (18000 s) and f is the fraction of incident light absorbed by the reaction mixture, determined using eq 1. An absorbance of the reaction mixture at 459 nm was measured to be 1.533.

$$f = 1 - 10^{-A} \qquad (1)$$

$$\Phi = \frac{\text{mol of product formed}}{\text{photon flux}\cdot t\cdot f} \qquad (2)$$

Sample quantum yield calculation

$f = 1\text{-}10^{(-1.533)} = 0.971$

$\Phi = 7.44\times10^{-5}$ mol/(2.351×10^{-8} einstein s^{-1} $\times$18000 s$\times$0.971) = 0.182

The reaction quantum yield (Φ) was thus determined to be 0.182.

Supplementary Figure 2. Absorbance of the reaction mixture solution.

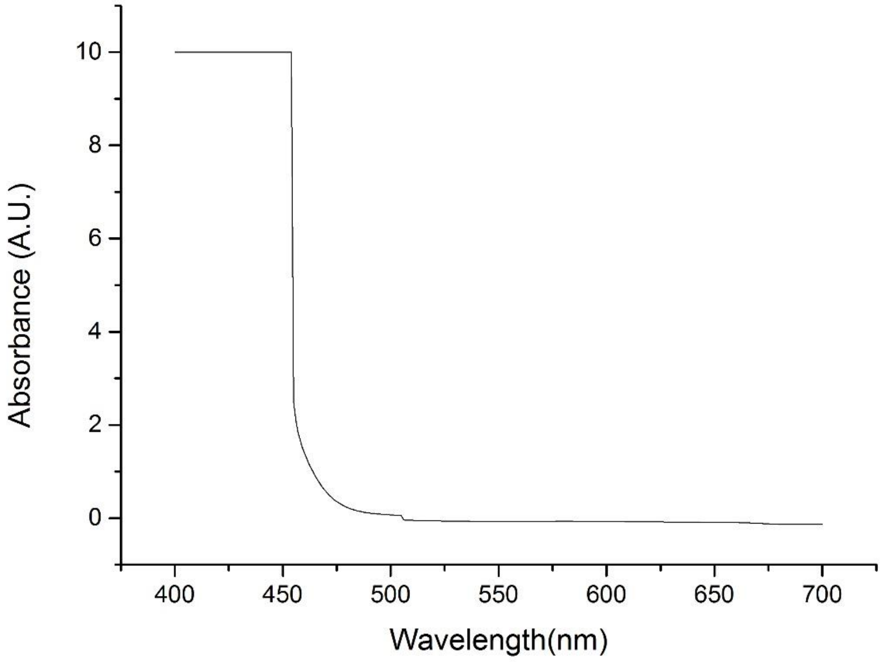

Radical Inhibition Experiment with TEMPO

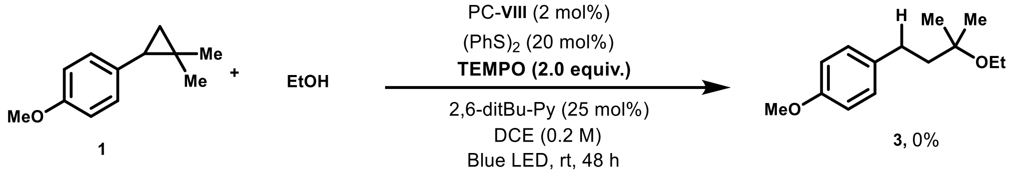

To an oven-dried 10 mL Schlenk tube equipped with a stir bar, $(PhS)_2$ (9.0 mg, 0.04 mmol, 20 mol%), photocatalyst (PC-VIII) (2.3 mg, 0.004 mmol, 2 mol%), TEMPO (62.5 mg, 0.4 mmol, 2.0 equiv.) were added. The Schlenk tube was purged with nitrogen three time, then EtOH (92 mg, 2.0 mmol, 10 equiv.), 2,6-ditBu-Py (9.6 mg, 0.05 mmol, 25 mol%), **1** (35.2 mg, 0.2 mmol, 1.0 equiv.), and 1,2-dichloroethane (1.0 mL, 0.2 M) were added. The Schlenk tube was sealed and irradiated with a 15W blue LED lamp (l = 459 nm) at room temperature for 48 h. The reaction solution was monitored by TLC, which showed no desired product formation.

Radical Clock Experiment

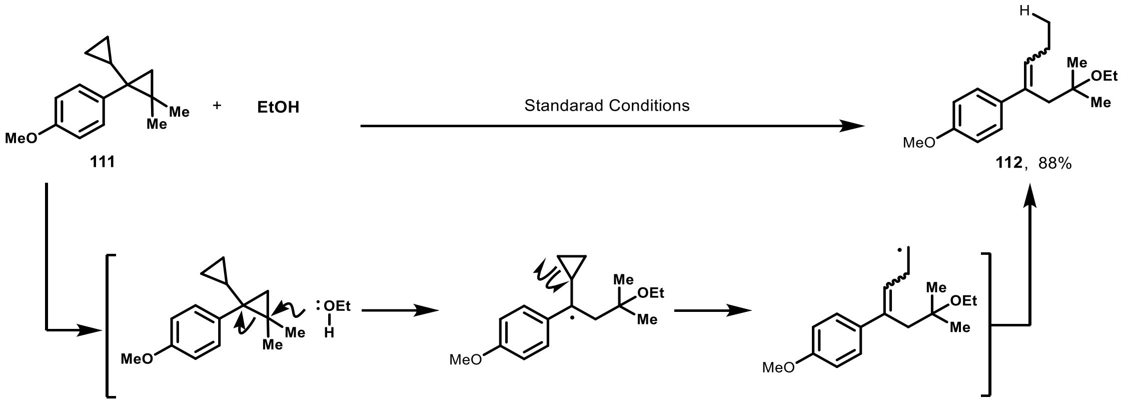

To an oven-dried 10 mL Schlenk tube equipped with a stir bar, $(PhS)_2$ (9.0 mg, 0.04 mmol, 20 mol%), photocatalyst (PC-VIII) (2.3 mg, 0.004 mmol, 2 mol%) were added. The Schlenk tube was purged with nitrogen three times, then EtOH (92 mg, 2.0 mmol, 10 equiv.), 2,6-ditBu-Py (9.6 mg, 0.05 mmol, 25 mol%), **111** (43.2 mg, 0.2 mmol, 1.0 equiv.), and dichloroethane (1.0 mL, 0.2 M) were added. The Schlenk tube was sealed and irradiated with a 15W blue LED lamp (l = 459 nm) at room temperature for 48 h. When the reaction was determined to be completed by TLC analysis, the mixture was evaporated to dryness under reduced pressure and the crude residue was purified by column chromatography on silica gel (DCM : PE = 1 : 4 and then PE : EtOAc = 20 : 1 to 10 : 1) to afford the desired product **112 (**3:1 alkene isomeric mixture**)**.

Deuterium Isotope Labeling Studies

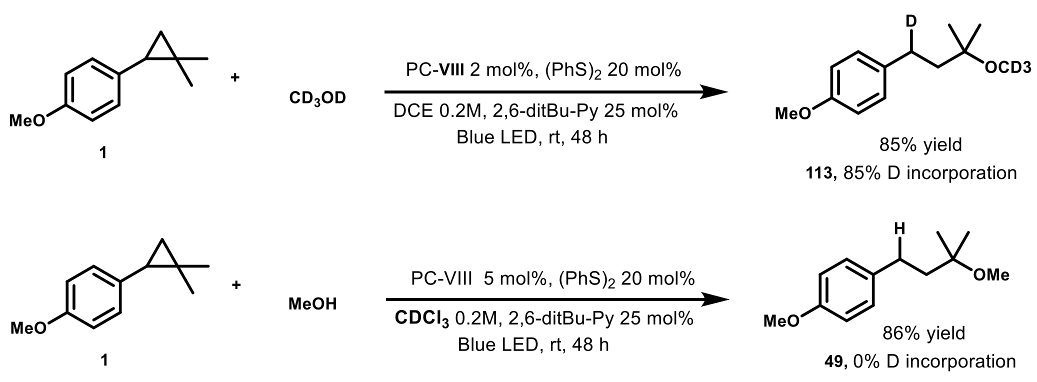

a) To an oven-dried 10 mL Schlenk tube equipped with a stir bar, $(PhS)_2$ (9.0 mg, 0.04 mmol, 20 mol%), photocatalyst (PC-VIII) (2.3 mg, 0.004 mmol, 2 mol%) were added. The Schlenk tube was purged with nitrogen three times, then CD_3OD (2.0 mmol, 10 equiv.), 2,6-ditBu-Py (9.6 mg, 0.05 mmol, 25 mol%), **1** (35.2 mg, 0.2 mmol, 1.0 equiv.), and 1,2-dichloroethane (1.0 mL, 0.2 M) were added. The Schlenk tube was sealed and irradiated with a 15W blue LED lamp (l = 459 nm) at room temperature for 48 h. When the reaction was determined to be completed by TLC analysis, the mixture was evaporated to dryness under reduced pressure and the crude residue was purified by column chromatography on silica gel (DCM : PE = 1 : 4 and then PE : EtOAc = 20 : 1 to 10 : 1) to afford the desired product **113** in 85% yield (85% D incorporation).

b) To an oven-dried 10 mL Schlenk tube equipped with a stir bar, $(PhS)_2$ (9.0 mg, 0.04 mmol, 20 mol%), photocatalyst (PC-VIII) (2.3 mg, 0.004 mmol, 2 mol%) were added. The Schlenk tube was purged with nitrogen three times, then MeOH (2.0 mmol, 10 equiv.), 2,6-ditBu-Py (9.6 mg, 0.05 mmol, 25 mol%), **1** (35.2 mg, 0.2 mmol, 1.0 equiv.), and $CDCl_3$ (1.0 mL, 0.2 M) were added. The Schlenk tube was sealed and irradiated with a 15W blue LED lamp (l = 459 nm) at room temperature for 48 h. When the reaction was determined to be completed by TLC analysis, the mixture was evaporated to dryness under reduced pressure and the crude residue was purified by column chromatography on silica gel (DCM : PE = 1 : 4 and then PE : EtOAc = 20 : 1 to 10 : 1) to afford the desired product **49** in 86% yield (0% D incorporation).

These two comparing experiments indicated that the proton of the nucleophile, other than the solvent, was transferred to the benzylic site of the product.

Reaction with Enantioenriched Substrate 114

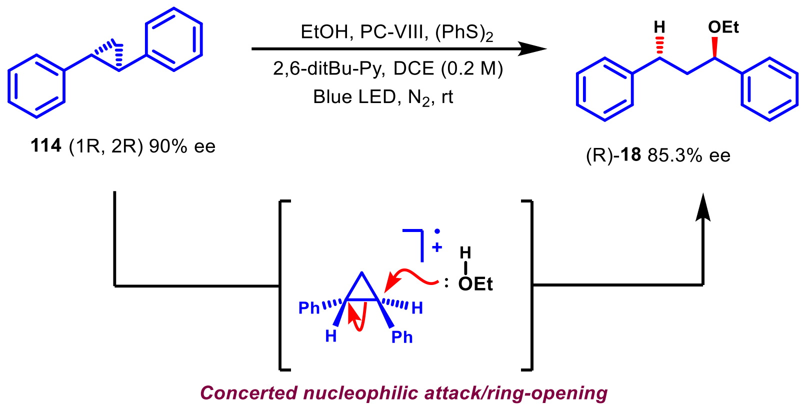

The starting material **(1R,2R)-114** (90.45% ee**)** slightly racemized to 83.1% ee after 60 min's irradiation by Blue-LEDs under N_2 without PC. Under standard reaction condition, the ee value of **(1R,2R)-114** and **(R)-18** also decline as time goes on.

Supplementary Figure 3. The evolvement of ee values of substrate **(1R,2R)-114** and product **(R)-18** as the reaction progresses.

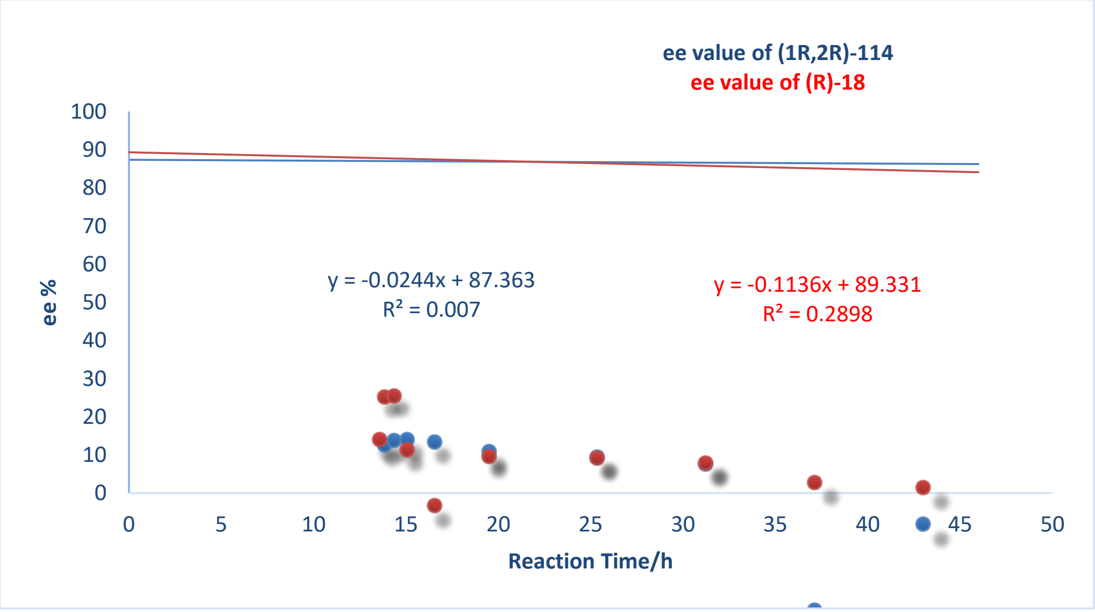

HPLC Chromatography of the Starting materials and Products

Supplementary Figure 4. HPLC chromatography of racemic 1,2-diphenylcyclopropane (**114**)
(Chiralpak OJ-H 250*4.6 mm/5 μm column, hexane/isopropanol = 95/5, flow rate = 1.0 mL/min, λ = 210 nm)

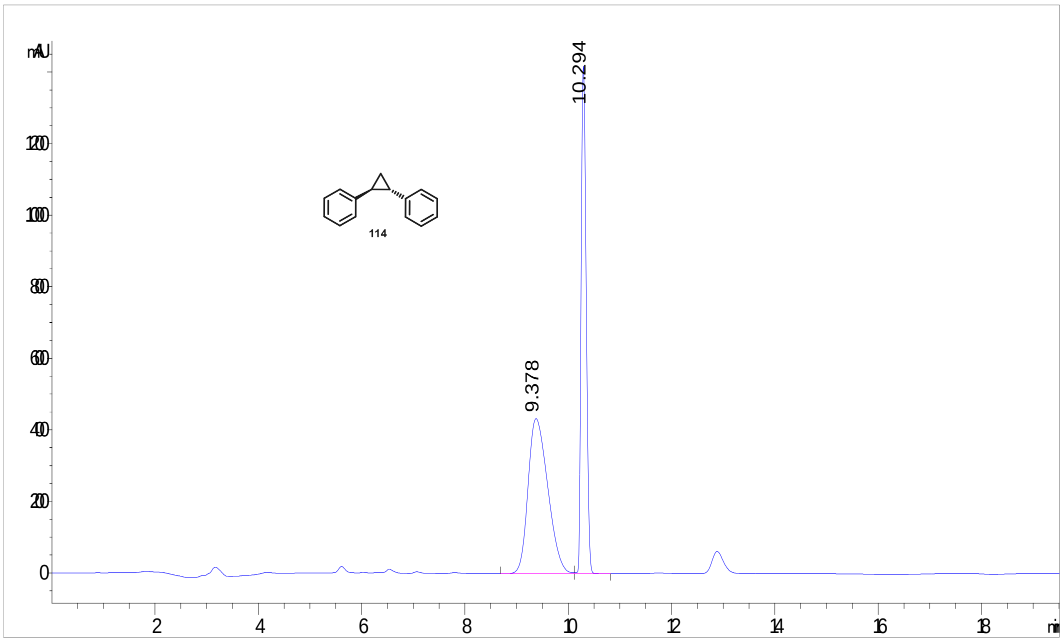

Peak #	RetTime [min]	Type	Width [min]	Area [mAU*s]	Height [mAU]	Area %
1	9.378	BV	0.4198	11507.9	433.3	54.383
2	10.294	VB	0.1073	9653	1419.3	45.617
Total				21160.9	1852.6	100%

Supplementary Figure 5. HPLC chromatography of (1R,2R)-1,2-diphenylcyclopropane (**(1R,2R)-114**).
(Chiralpak OJ-H 250*4.6 mm/5 μm column, hexane/isopropanol = 95/5, flow rate = 1.0 mL/min, λ = 210 nm)

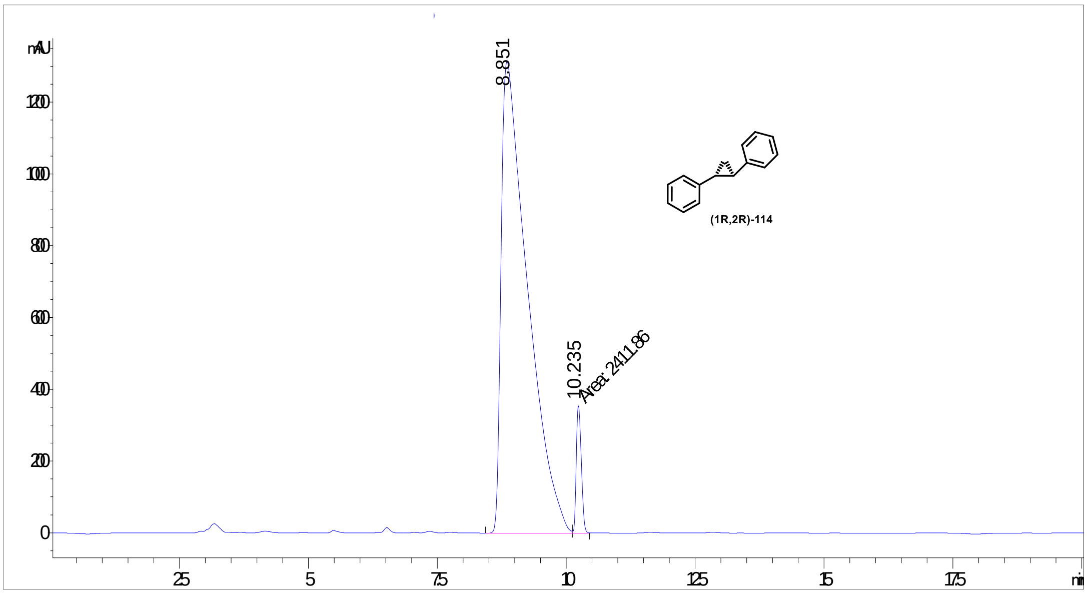

Peak #	RetTime [min]	Type	Width [min]	Area [mAU*s]	Height [mAU]	Area %
1	8.851	BV	0.4981	46632.7	1313.9	95.082

2	10.235	MF	0.1131	2411.9	355.4	4.918
Total				49044.6	1669.3	100%
						(ee 90.16%)

Supplementary Figure 6. HPLC chromatography of racemic (1-ethoxypropane-1,3-diyl)dibenzene (**18**)
(Chiralpak OJ-H 250*4.6 mm/5 μm column, hexane/isopropanol = 95/5, flow rate = 1.0 mL/min, λ = 210 nm)

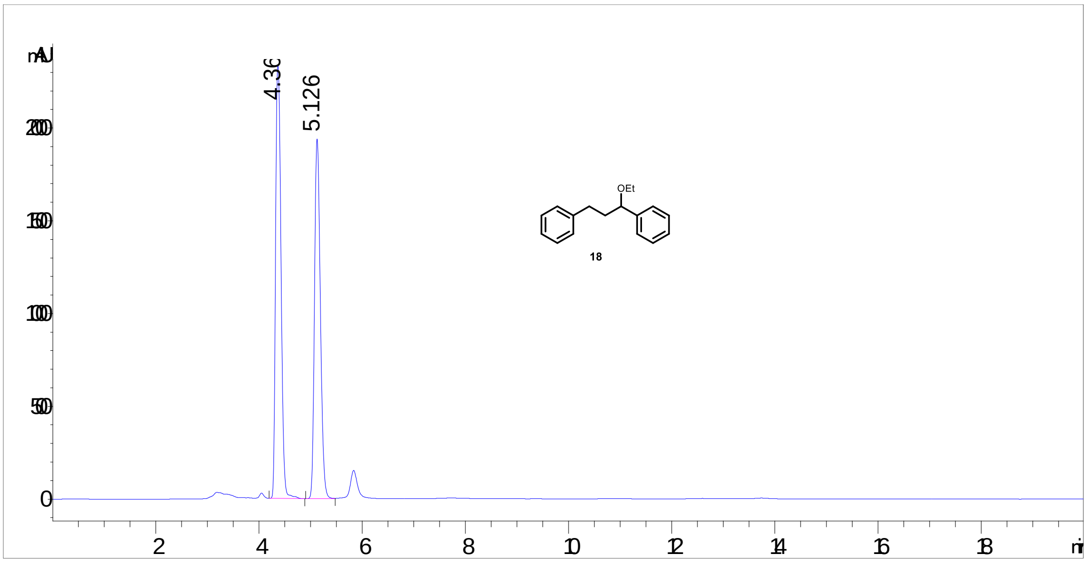

Peak #	RetTime [min]	Type	Width [min]	Area [mAU*s]	Height [mAU]	Area %
1	4.366	VB	0.1153	17113.8	2339.4	52.774
2	5.126	BV	0.124	15314.4	1942.4	47.226
Total				32428.2	4281.8	100%

Supplementary Figure 7. HPLC chromatography of reaction solution with reaction time of 5 min.
(Chiralpak OJ-H 250*4.6 mm/5 μm column, hexane/isopropanol = 95/5, flow rate = 1.0 mL/min, λ = 210 nm)

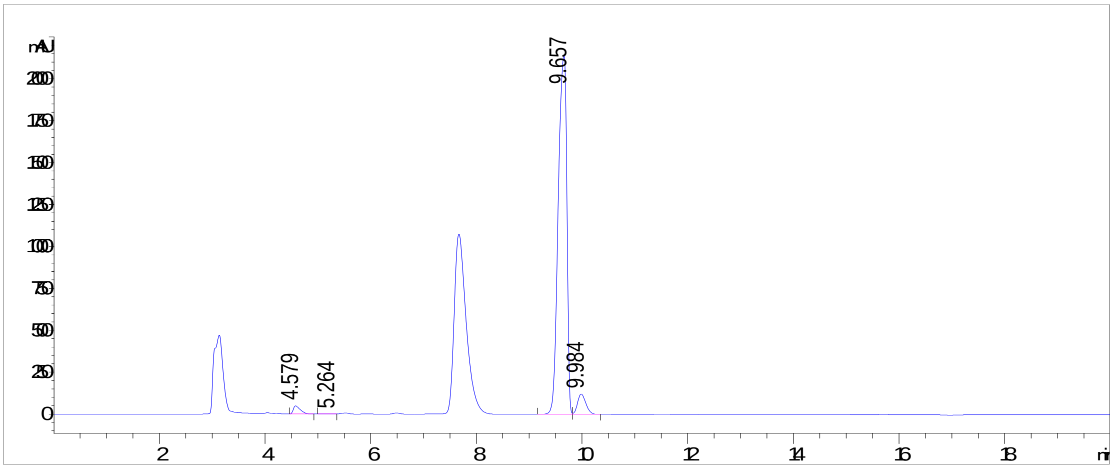

Peak #	RetTime [min]	Type	Width [min]	Area [mAU*s]	Height [mAU]	Area %
1	4.579	BB	0.1304	432.5	48.3	1.675
2	5.264	BV	0.1488	13.4	1.3	0.052
3	9.657	BV	0.1849	24063.3	2145	93.201
4	9.984	VB	0.1686	1309.5	120.7	5.072
Total				25818.7	2315.3	100%

((**R)-18** ee 93.98%)

((**1R,2R)-114** ee 89.68%)

Supplementary Figure 8. HPLC chromatography of reaction solution with reaction time of 15 min.

(Chiralpak OJ-H 250*4.6 mm/5 μm column, hexane/isopropanol = 95/5, flow rate = 1.0 mL/min, λ = 210 nm)

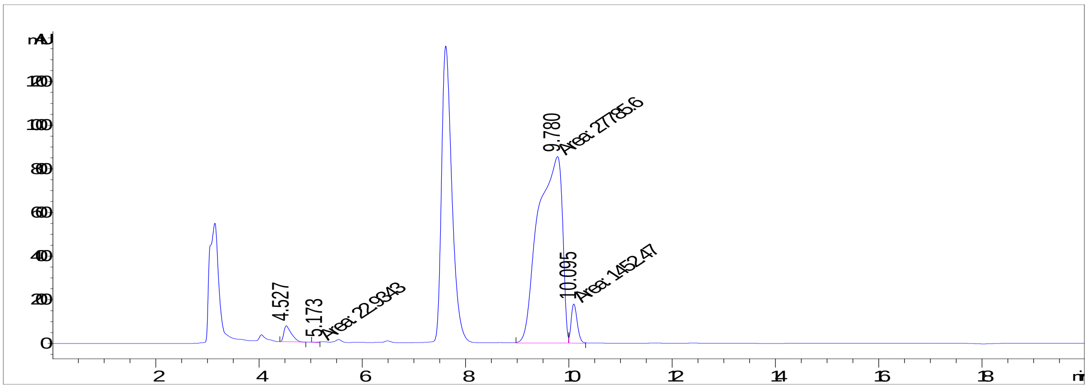

Peak #	RetTime [min]	Type	Width [min]	Area [mAU*s]	Height [mAU]	Area %
1	4.527	BB	0.1453	739.4	73.5	2.465
2	5.173	MM	0.1058	22.9	3.6	0.076
3	9.78	FM	0.5416	27785.6	855	92.618
4	10.095	MF	0.1348	1452.5	179.6	4.842
Total				30000.4	1111.7	100%

((**R)-18** ee 94.02%)

((**1R,2R)-114** ee 90.06%)

Supplementary Figure 9. HPLC chromatography of reaction solution with reaction time of 30 min.

(Chiralpak OJ-H 250*4.6 mm/5 μm column, hexane/isopropanol = 95/5, flow rate = 1.0 mL/min, λ = 210 nm)

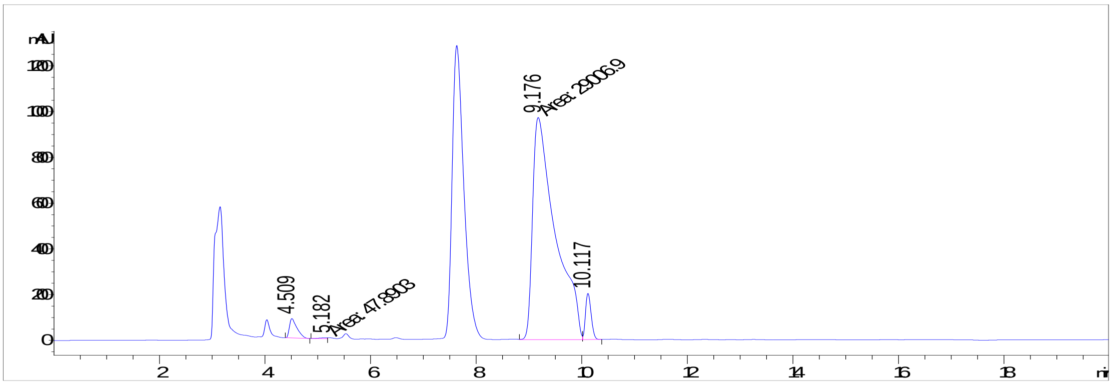

Peak #	RetTime [min]	Type	Width [min]	Area [mAU*s]	Height [mAU]	Area %
1	4.509	BB	0.1415	844	85.3	2.688
2	5.182	MF	0.1732	47.9	4.6	0.153
3	9.176	FM	0.4978	29006.9	971.3	92.389
4	10.117	VB	0.1185	1497.6	201.9	4.770
Total				31396.4	1263.1	100%

((**R**)-**18** ee 89.23%)

((**1R,2R**)-**114** ee 90.18%)

Supplementary Figure 10. HPLC chromatography of reaction solution with reaction time of 1 h.
(Chiralpak OJ-H 250*4.6 mm/5 μm column, hexane/isopropanol = 95/5, flow rate = 1.0 mL/min, λ = 210 nm)

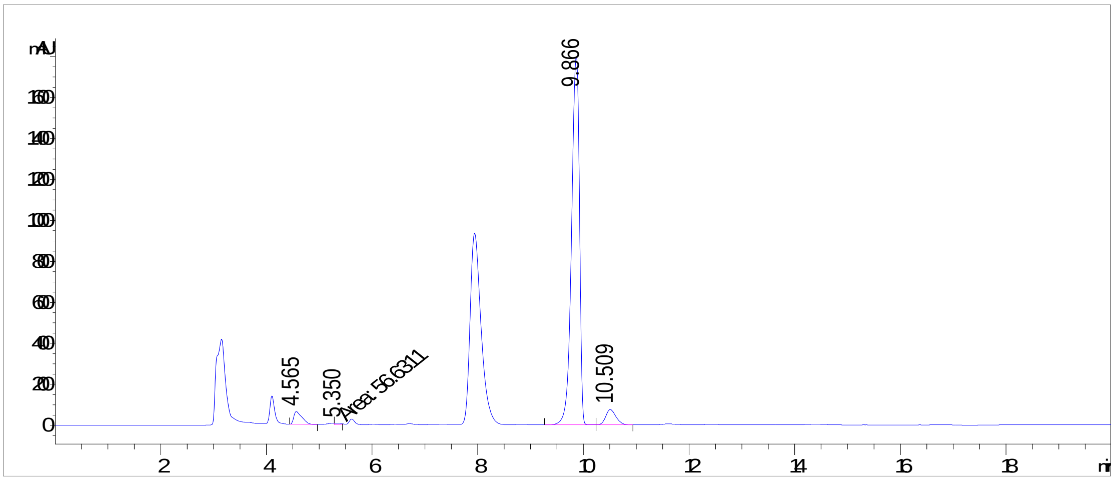

Peak #	RetTime [min]	Type	Width [min]	Area [mAU*s]	Height [mAU]	Area %
1	4.565	BB	0.153	663.4	61.9	3.252
2	5.350	FM	0.278	56.6	6.7	0.278
3	9.866	BV	0.1653	18688.1	1798	91.600
4	10.509	VB	0.2065	993.5	74	4.870
Total				20431.5	1940.6	100%

((**R**)-**18** ee 84.25%)

((**1R,2R)-114** ee 89.90%)

Supplementary Figure 11. HPLC chromatography of reaction solution with reaction time of 2 h.
(Chiralpak OJ-H 250*4.6 mm/5 μm column, hexane/isopropanol = 95/5, flow rate = 1.0 mL/min, λ = 210 nm)

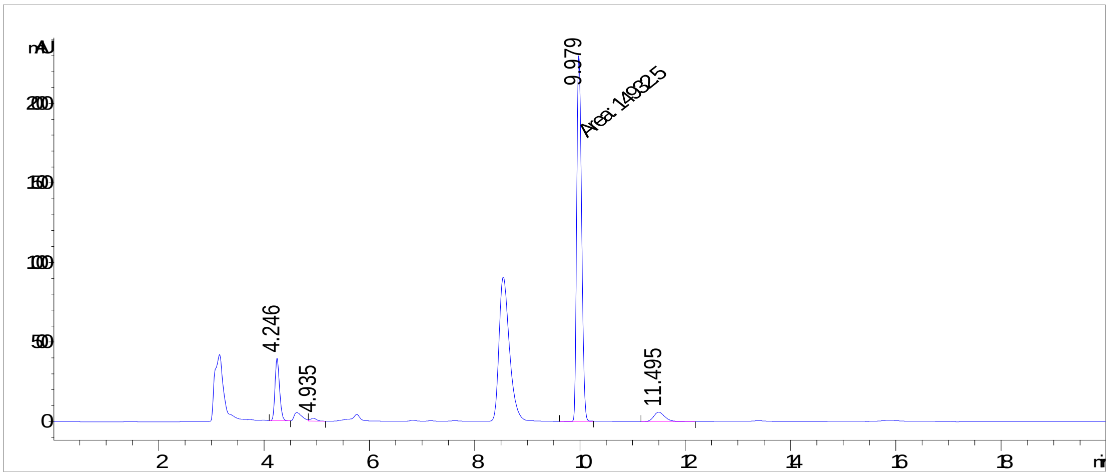

Peak #	RetTime [min]	Type	Width [min]	Area [mAU*s]	Height [mAU]	Area %
1	4.246	BB	0.0915	2339.1	392.4	12.798
2	4.935	VB	0.1242	141.3	17.5	0.773
3	9.979	MF	0.1082	14932.5	2300.6	81.697
4	11.495	BB	0.226	865.1	58.7	4.733
Total				18278	2769.2	100%

((**R)-18** ee 88.61%)

((**1R,2R)-114** ee 89.05%)

Supplementary Figure 12. HPLC chromatography of reaction solution with reaction time of 4 h.
(Chiralpak OJ-H 250*4.6 mm/5 μm column, hexane/isopropanol = 95/5, flow rate = 1.0 mL/min, λ = 210 nm)

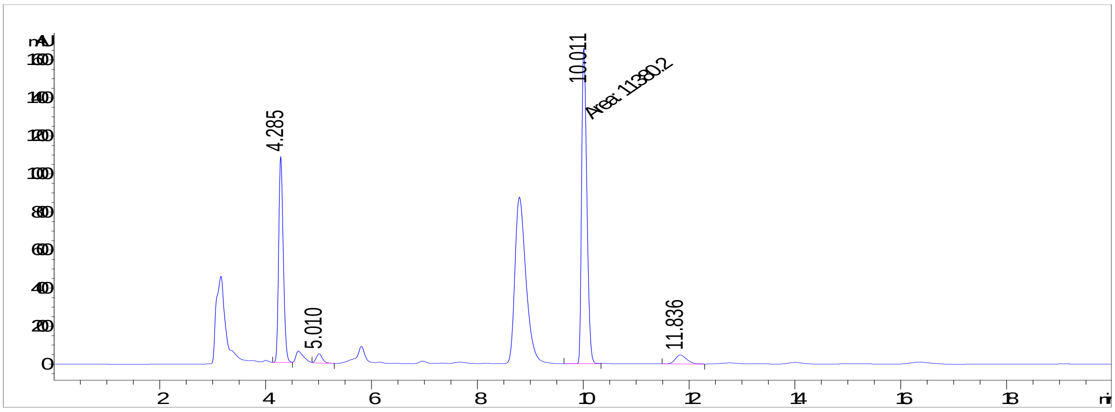

Peak #	RetTime [min]	Type	Width [min]	Area [mAU*s]	Height [mAU]	Area %

1	4.285	BV	0.0923	6529.4	1083.1	34.368
2	5.01	VB	0.1252	399.2	48.9	2.101
3	10.011	MF	0.1143	11380.2	1660	59.901
4	11.836	BB	0.2263	689.5	46.7	3.629
Total				18998.3	2838.7	100%

((**R)-18** ee 88.48%)

((**1R,2R)-114** ee 88.58%)

Supplementary Figure 13. HPLC Chromatography of reaction solution with reaction time of 6 h.

(Chiralpak OJ-H 250*4.6 mm/5 μm column, hexane/isopropanol = 95/5, flow rate = 1.0 mL/min, λ = 210 nm)

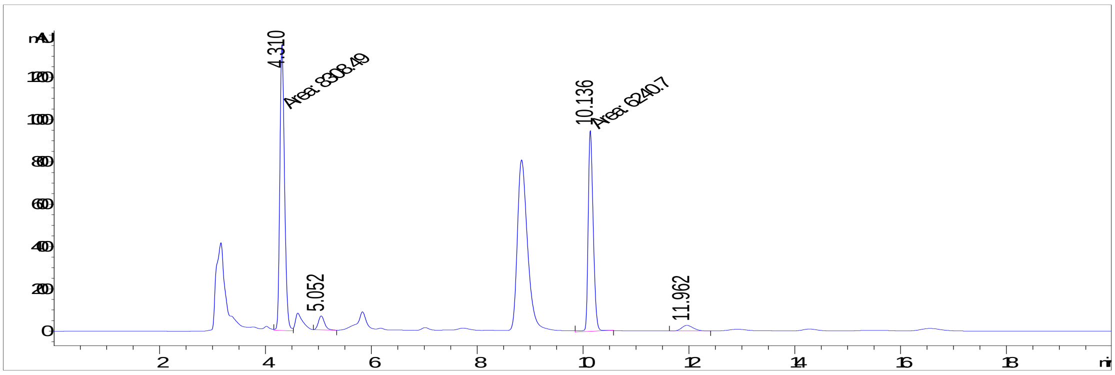

Peak #	RetTime [min]	Type	Width [min]	Area [mAU*s]	Height [mAU]	Area %
1	4.31	MM	0.1023	8308.5	1353.3	53.683
2	5.052	BB	0.1233	527.6	65.9	3.409
3	10.136	MM	0.1094	6240.7	950.6	40.322
4	11.962	BB	0.2324	400.3	26.5	2.586
Total				15477.1	2396.3	100%

((**R)-18** ee 88.06%)

((**1R,2R)-114** ee 87.95%)

Supplementary Figure 14. HPLC Chromatography of reaction solution with reaction time of 8 h.

(Chiralpak OJ-H 250*4.6 mm/5 μm column, hexane/isopropanol = 95/5, flow rate = 1.0 mL/min, λ = 210 nm)

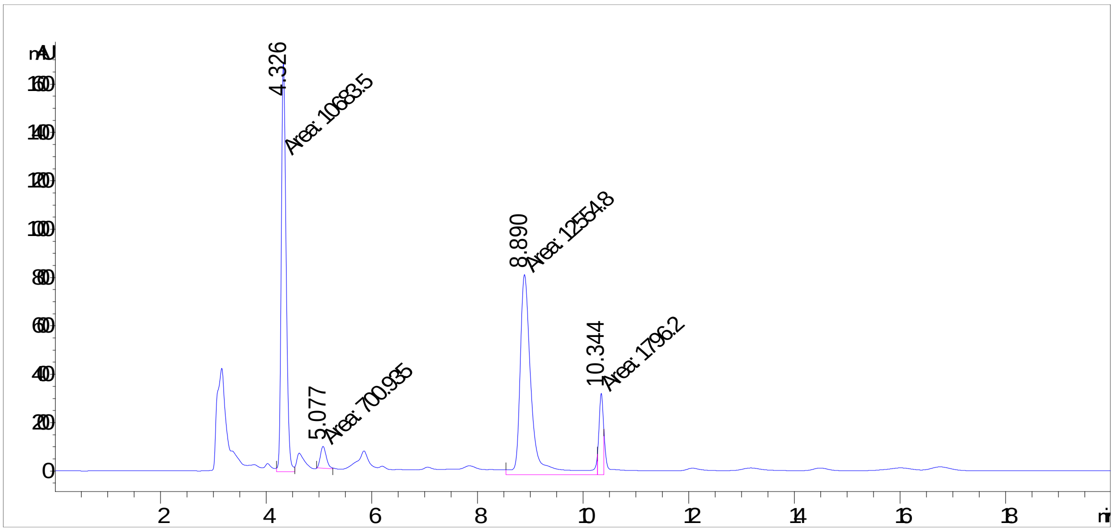

Peak #	RetTime [min]	Type	Width [min]	Area [mAU*s]	Height [mAU]	Area %
1	4.326	MM	0.1049	10683.5	1697.1	41.513
2	5.077	MM	0.1288	700.9	90.7	2.724
3	8.89	MF	0.2526	12554.8	828.2	48.784
4	10.344	FM	0.0889	1796.2	336.6	6.979
Total				23961.9	2911.6	100%

((**R**)-**18** ee 87.68%)

((**1R,2R**)-**114** ee 74.97%)

Supplementary Figure 15. HPLC chromatography of reaction solution with reaction time of 10 h.

(Chiralpak OJ-H 250*4.6 mm/5 μm column, hexane/isopropanol = 95/5, flow rate = 1.0 mL/min, λ = 210 nm)

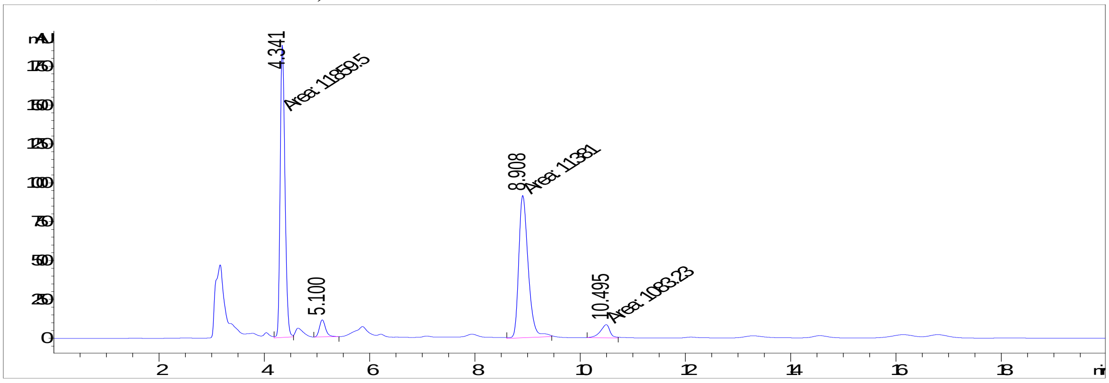

Peak #	RetTime [min]	Type	Width [min]	Area [mAU*s]	Height [mAU]	Area %
1	4.341	MM	0.1051	11859.5	1880.4	47.018
2	5.1	BB	0.1252	899.4	110.2	3.566
3	8.908	MM	0.2073	11381	915.2	45.121
4	10.495	FM	0.2124	1083.2	85	4.295
Total				25223.1	2990.8	100%

((**R**)-**18** ee 85.90%)

((**1R,2R)-114** ee 82.62%)

Supplementary Figure 16. HPLC chromatography of reaction solution with reaction time of 22 h.
(Chiralpak OJ-H 250*4.6 mm/5 μm column, hexane/isopropanol = 95/5, flow rate = 1.0 mL/min, λ = 210 nm)

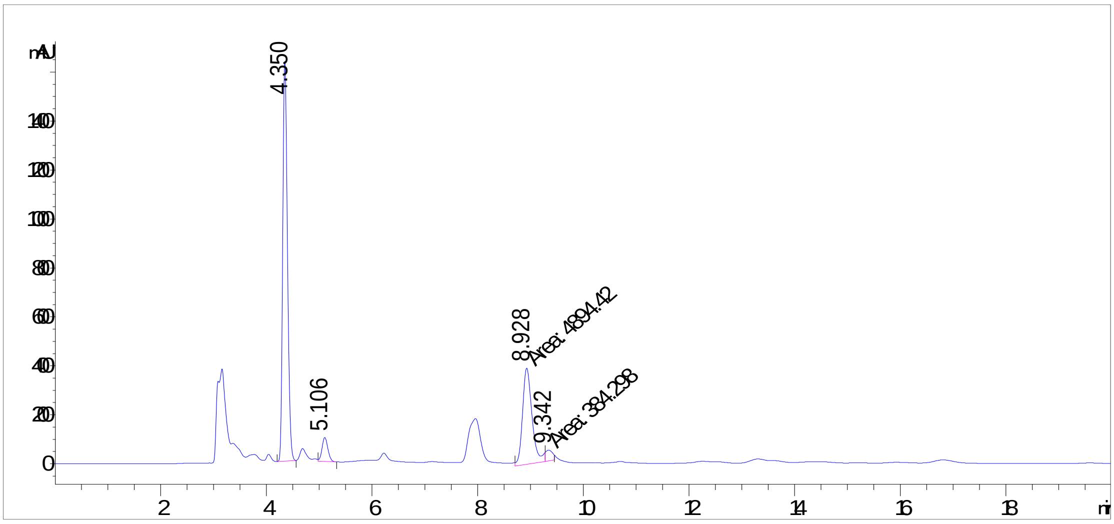

Peak #	RetTime [min]	Type	Width [min]	Area [mAU*s]	Height [mAU]	Area %
1	4.35	BB	0.0958	10055.4	1633.9	62.498
2	5.106	VB	0.1177	754.9	98.2	4.692
3	8.928	MF	0.2076	4894.4.1	392.9	30.421
4	9.342	FM	0.1456	384.3	44	2.389
Total				16338.2	2170.3	100%

((**R)-18** ee 86.03%)
((**1R,2R)-114** ee 85.44%)

Supplementary Figure 17. HPLC chromatography of reaction solution with reaction time of 34 h.
(Chiralpak OJ-H 250*4.6 mm/5 μm column, hexane/isopropanol = 95/5, flow rate = 1.0 mL/min, λ = 210 nm)

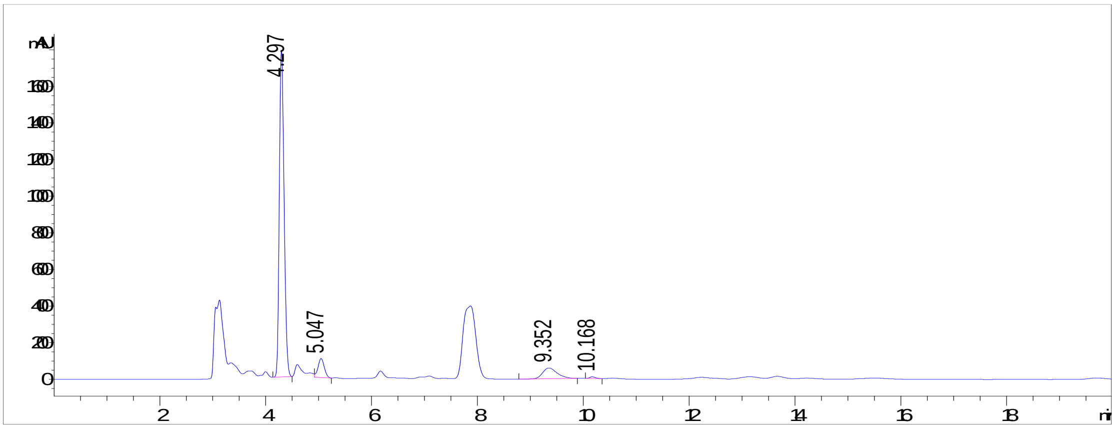

Peak #	RetTime [min]	Type	Width [min]	Area [mAU*s]	Height [mAU]	Area %
1	4.297	BB	0.0969	11158.3	1786.7	84.350
2	5.047	VB	0.1261	863.7	104.9	6.529
3	9.352	BB	0.2947	1137.3	59	8.597
4	10.168	BB	0.1132	69.3	9.5	0.524
Total				13228.6	1960.1	100%

((**R)-18** ee 85.63%)

((**1R,2R)-114** ee 88.51%)

Supplementary Figure 18. HPLC Chromatography of reaction solution with reaction time of 46 h.
(Chiralpak OJ-H 250*4.6 mm/5 μm column, hexane/isopropanol = 95/5, flow rate = 1.0 mL/min, λ = 210 nm)

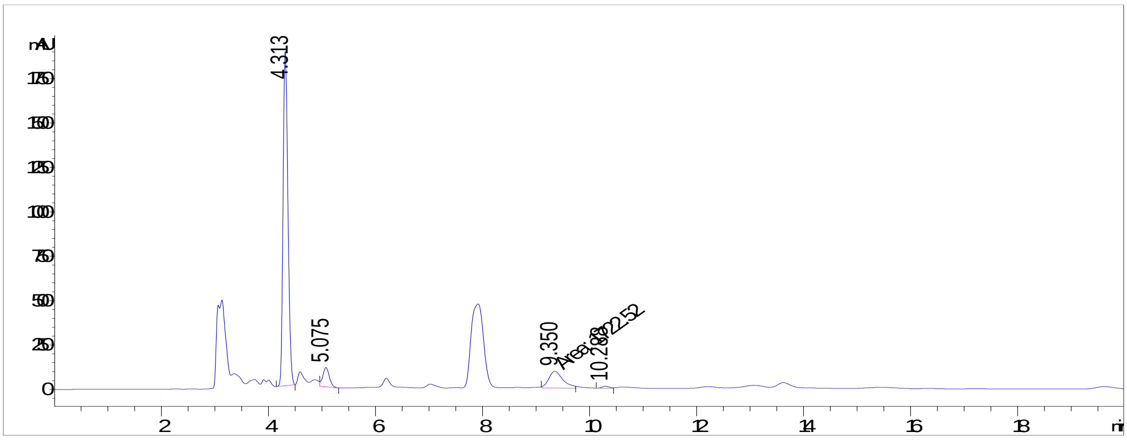

Peak #	RetTime [min]	Type	Width [min]	Area [mAU*s]	Height [mAU]	Area %
1	4.313	BB	0.0974	11821.9	1879.2	81.079
2	5.075	VB	0.1314	927.9	108.9	6.364
3	9.35	FM	0.3054	1722.5	94	11.814
4	10.288	VV	0.1375	108.5	11.8	0.744
Total				14580.8	2093.9	100%

((**R)-18** ee 85.44%)

((**1R,2R)-114** ee 88.15%)

Supplementary Figure 19. HPLC chromatography of **(R)-18** after 46 h reaction.
(Chiralpak OJ-H 250*4.6 mm/5 μm column, hexane/isopropanol = 95/5, flow rate = 1.0 mL/min, λ = 210 nm)

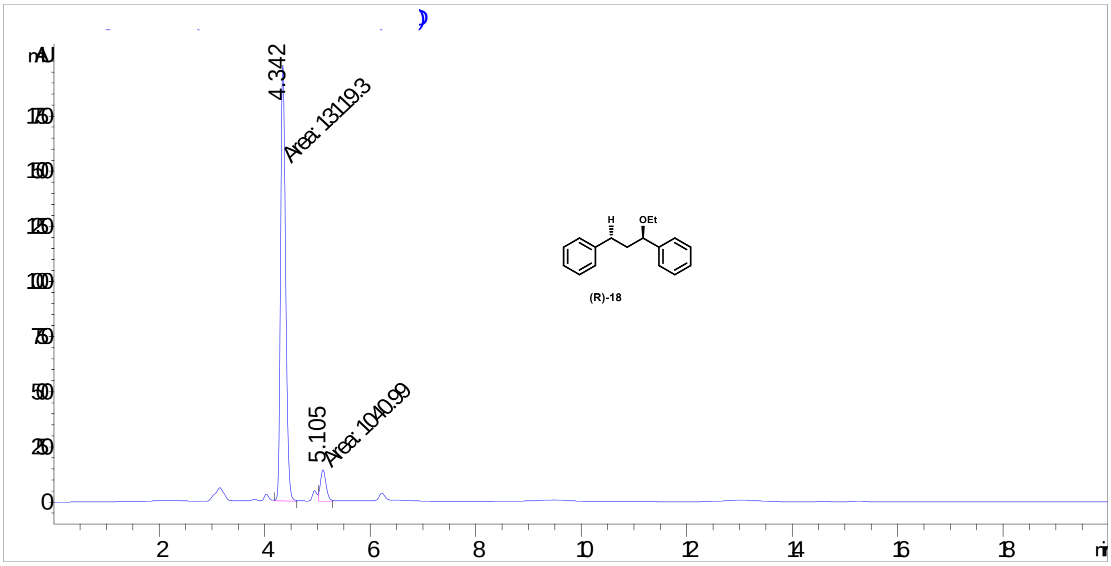

Peak #	RetTime [min]	Type	Width [min]	Area [mAU*s]	Height [mAU]	Area %
1	4.342	MF	0.1106	13119.3	1976.9	92.649
2	5.105	FM	0.122	1041	142.2	7.351
Total				14160.3	2119.1	100%

((**R**)-**18** ee 85.30%)

Supplementary Figure 20. HPLC chromatography of mixture of **(R)-18** and racemic **18**.
(Chiralpak OJ-H 250*4.6 mm/5 μm column, hexane/isopropanol = 95/5, flow rate = 1.0 mL/min, λ = 210 nm)

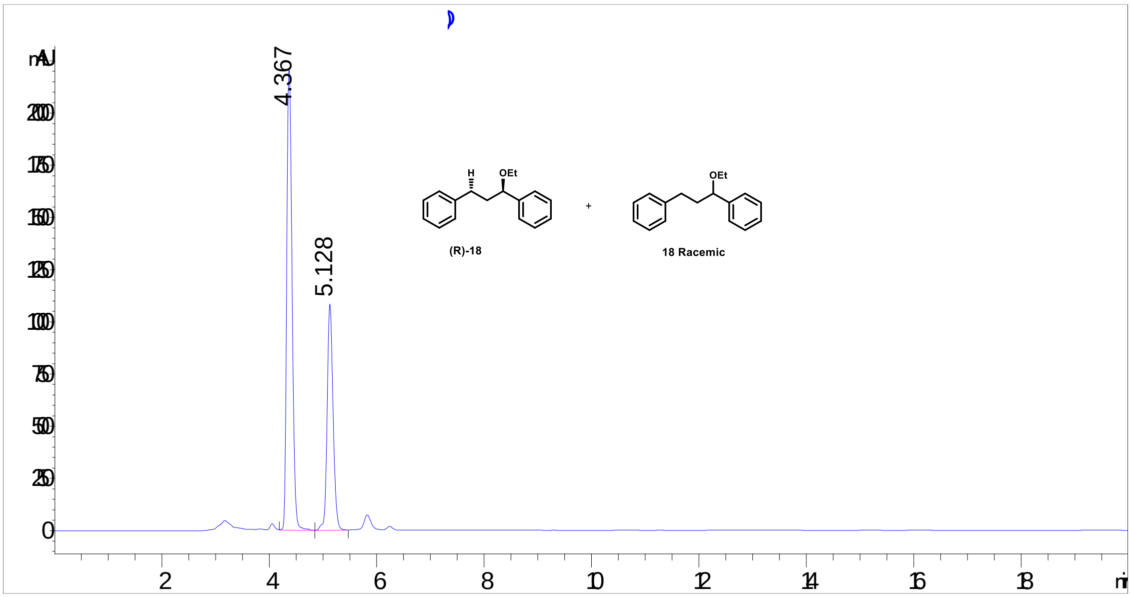

Peak #	RetTime [min]	Type	Width [min]	Area [mAU*s]	Height [mAU]	Area %
1	4.367	VB	0.1087	15279.8	2209.4	64.782
2	5.128	BV	0.1174	8306.7	1084.1	35.218

Total	23586.5	3293.5	100%

Assignment of absolute configuration of 18

In order to determine the absolute configuration of **18**, we have prepared (S)-1,3-diphenylpropan-1-ol in 99% ee from (R)-phenyloxirane and benzylmagnesium chloride following the literature's method[12].

Supplementary Figure 21. HPLC chromatography of racemic 1,3-diphenylpropan-1-ol:

(Chiralpak OJH 250*4.6 mm/5 μm column, hexane/isopropanol = 90 : 10, flow rate = 0.5 mL/min, λ = 214 nm, retention time: t_1 = 20.0 min, t_2 = 22.0 min)

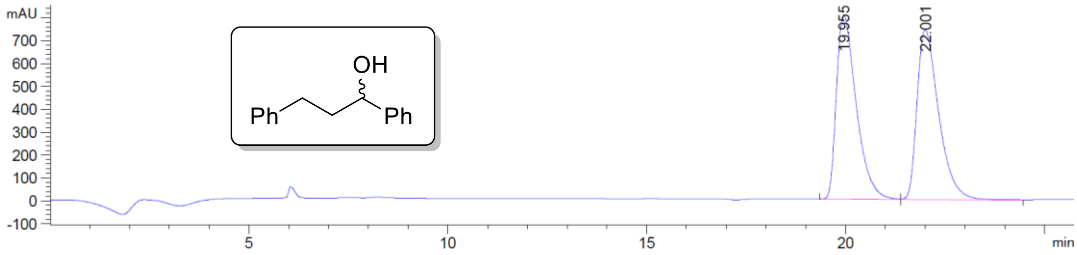

Signal 2: DAD1 B, Sig=214,4 Ref=off

Peak #	RetTime [min]	Type	Width [min]	Area [mAU*s]	Height [mAU]	Area %
1	19.955	BV	0.5285	2.79383e4	805.88489	49.8789
2	22.001	VB	0.5794	2.80739e4	742.02374	50.1211
Totals :				5.60122e4	1547.90863	

Supplementary Figure 22. HPLC chromatography of (S)-1,3-diphenylpropan-1-ol (99% ee):

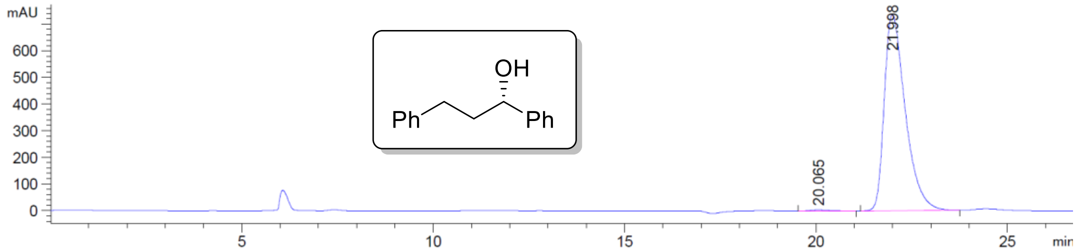

Signal 2: DAD1 B, Sig=214,4 Ref=off

Peak #	RetTime [min]	Type	Width [min]	Area [mAU*s]	Height [mAU]	Area %
1	20.065	BB	0.4751	156.75536	4.70053	0.5557
2	21.998	BB	0.5800	2.80512e4	737.13208	99.4443

Totals : 2.82080e4 741.83261

(S)-(1-ethoxypropane-1,3-diyl)dibenzene [**(S)-18**] was prepared in 95% ee from (S)-1,3-diphenylpropan-1-ol by ethylation:

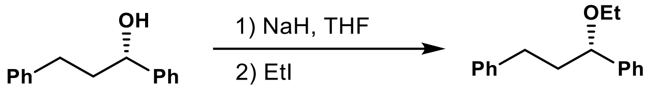

Supplementary Figure 23. HPLC chromatography of (S)-(1-ethoxypropane-1,3-diyl)dibenzene ([**(S)-18**]):
(Chiralpak OJH 250*4.6 mm/5 μm column, isopropanol / hexane = 5 : 95, flow rate = 1.0 mL/min, λ = 210 nm, retention time: t_1 = 4.5 min, t_2 = 5.5 min)

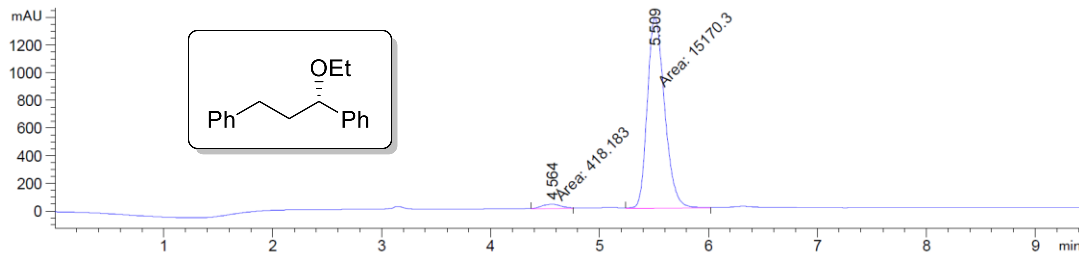

Signal 3: DAD1 C, Sig=210,4 Ref=off

Peak #	RetTime [min]	Type	Width [min]	Area [mAU*s]	Height [mAU]	Area %
1	4.564	MM	0.2119	418.18341	32.88498	2.6826
2	5.509	MM	0.1838	1.51703e4	1375.79321	97.3174

Totals : 1.55885e4 1408.67819

Supplementary Figure 24. HPLC chromatography of **18** from reaction of (1R,2R)-**114**:

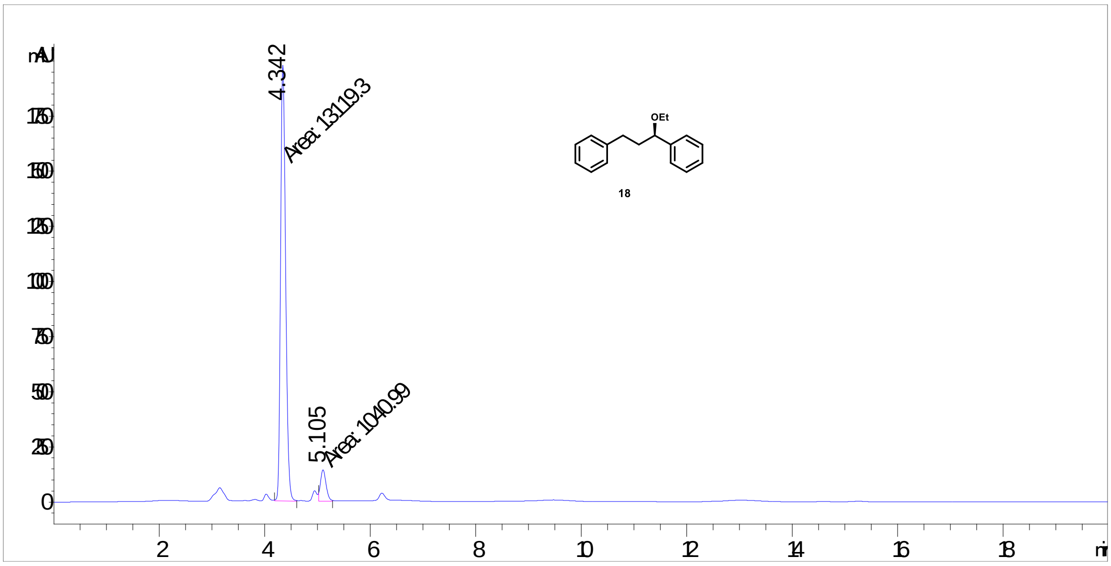

Peak #	RetTime [min]	Type	Width [min]	Area [mAU*s]	Height [mAU]	Area %
1	4.342	MF	0.1106	13119.3	1976.9	92.649
2	5.105	FM	0.122	1041	142.2	7.351
Total				14160.3	2119.1	100%

The absolute configuration of **18** was therefore assigned to be R.

Kinetic Study

To gain better insight into the kinetic of the reaction, a set of competition reactions were executed. First, the influence of nucleophile was investigated by competition reaction of two arylcyclopropanes. The chloride ion exhibited much higher reactivity over trifluoromethanesulfonamide and acetic acid, two nucleophiles also employed in this work. This result indicated that the nucleophilicity of nucleophile had a significant impact on the reaction rate.

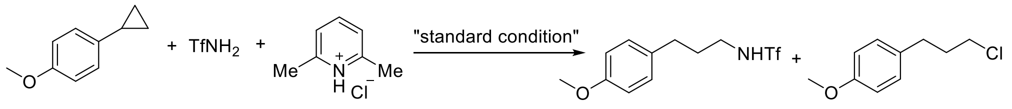

118 (2.0 equiv) (2.0 equiv) **37** **100**

100/37 > 20 : 1

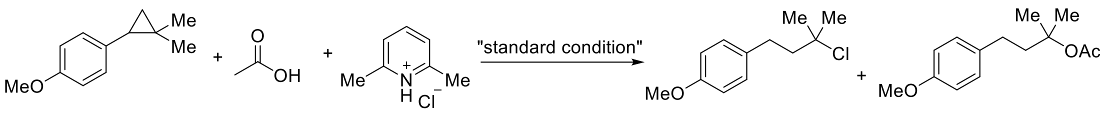

1 (2.0 equiv) (2.0 equiv) **108** **74**

108/74 > 20 : 1

We have also compared reactivity of arylcyclopropanes with different alkyl substitution pattern by a similar competitive experiment. Substrate with two methyl substituents on the cyclopropane reacted faster than counterpart with one methyl substituent upon reaction with ethanol, which is in consistent with the trend we concluded in our manuscript, "…demanding substituents on cyclopropane are not detrimental but conducive to the ring-opening cleavage…".

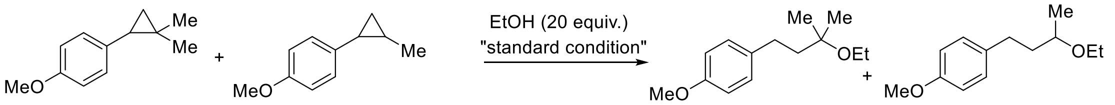

1 **119** **3** **120**

3/120 = 7.7:1

Finally, the substitution effect on the aryl ring is surveyed by comparing relative reaction rate of structurally similar arylcyclopropanes with different aryl substituents. In these cases we choose chloride as the nucleophile because electron-poor arylcyclopropane reacted sluggishly with nucleophiles such as ethanol and $TfNH_2$. The reaction outcome showed that a strong electron-donating methoxy group could significantly accelerate the reaction rate to nearly one magnitude, whereas other weak electron-donating or electron-withdrawing groups reacted in similar rates, with no obvious trend. Unfortunately, no Hammett plot could be fitted from the current data,

which might be caused by the high nucleophilicity of chloride that minimize the effect of substituents with weak electron property. The extraordinary reaction rate of the methoxy substituted cyclopropane might be due to the relative lower oxidation potential of this particular substrate compared with the unsubstituted and *p*-bromide substituted ones (1.30 V *vs* 1.55 V and 1.60 V). From another point of view, the high reactivity of methoxy substituted cyclopropane could also be rationalized by more effective molecular interaction between SOMO of the radical cation intermediate and HOMO of the nucleophile (*Angew. Chem. Int. Ed.* **2012**, *51*, 7259; **2022**, *61*, e202206064).

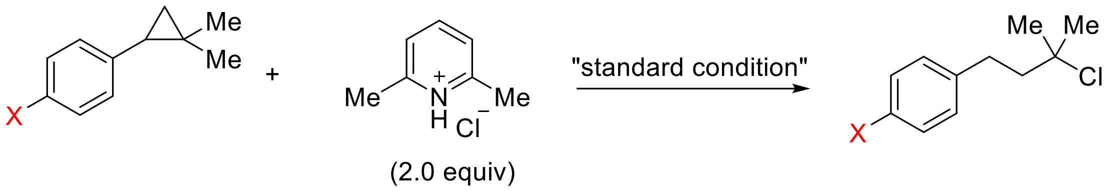

X:	H	F	Br	Cl	Me	t-Bu	OMe
relative rate:	1	1.40	2.30	1.93	2.20	3.23	9.9

In conclusion, three factors: nucleophilicity of nucleophile, alkyl substituent on the cyclopropane and substituent on the aryl ring have been demonstrated to significantly affect kinetic of the reaction. Unfortunately, we are unable to quantification the impact of each factor at current stage. As the three factors are separately involved in the initial single-electron-oxidation step and the ring-opening nucleophilic attack step, we are also unable to identify the rate-determining step for individual reactions. More research in depth about the kinetic of the reaction may help to gain insight into a deeper understanding of the mechanism.

^{1}H NMR and ^{13}C NMR Spectrum of Structurally Novel Compounds

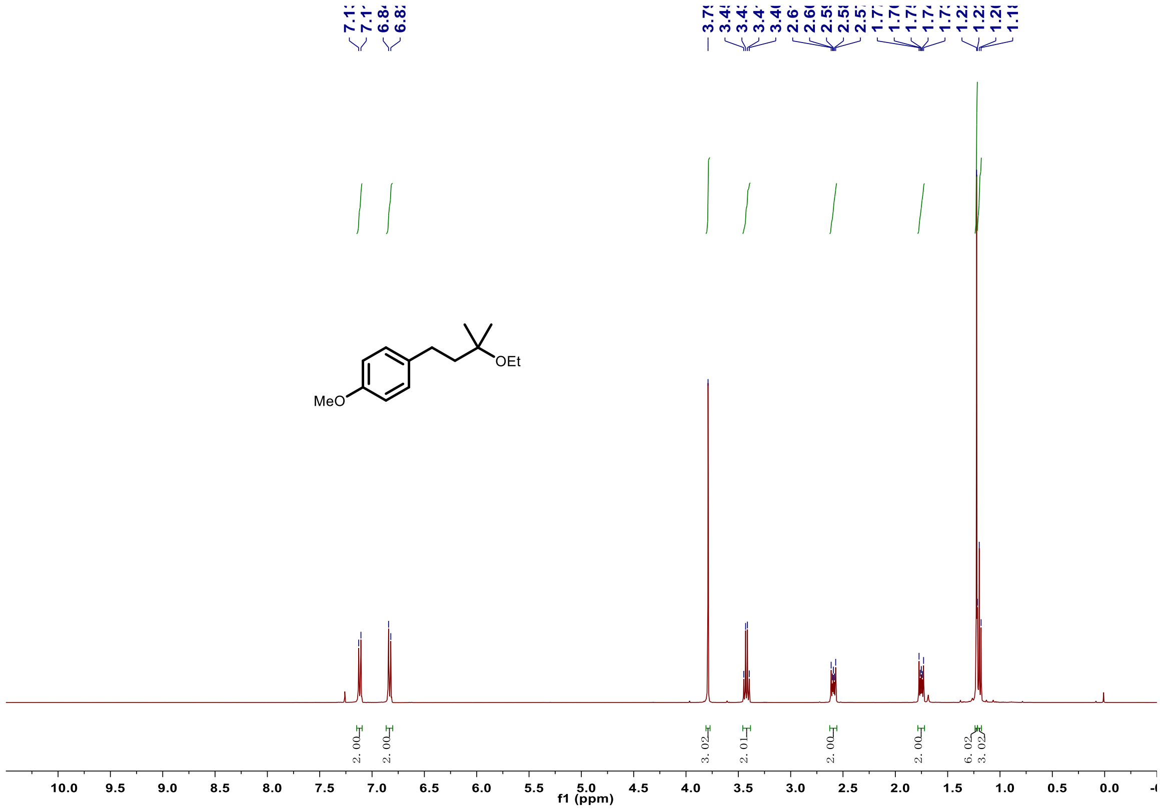

Supplementary Figure 25. ^{1}H NMR (400 MHz, $CDCl_3$) spectrum for 3

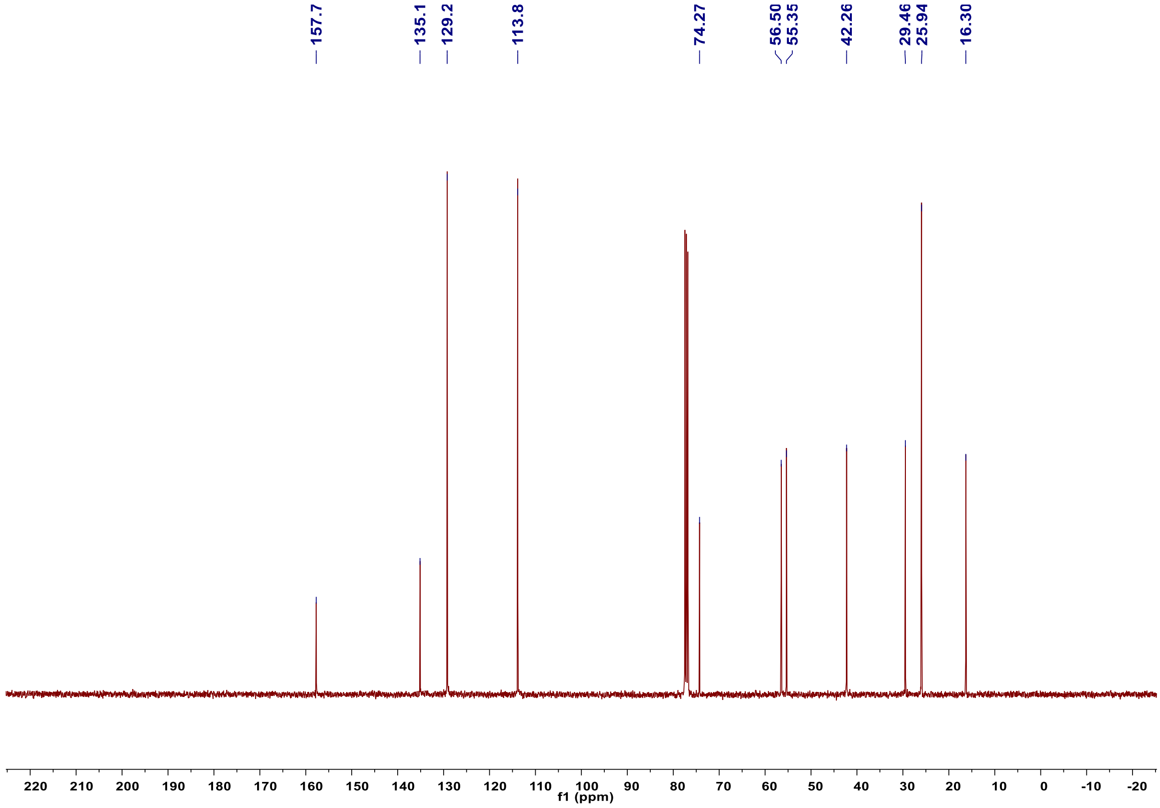

Supplementary Figure 26. ^{13}C NMR (100 MHz, $CDCl_3$) spectrum for 3

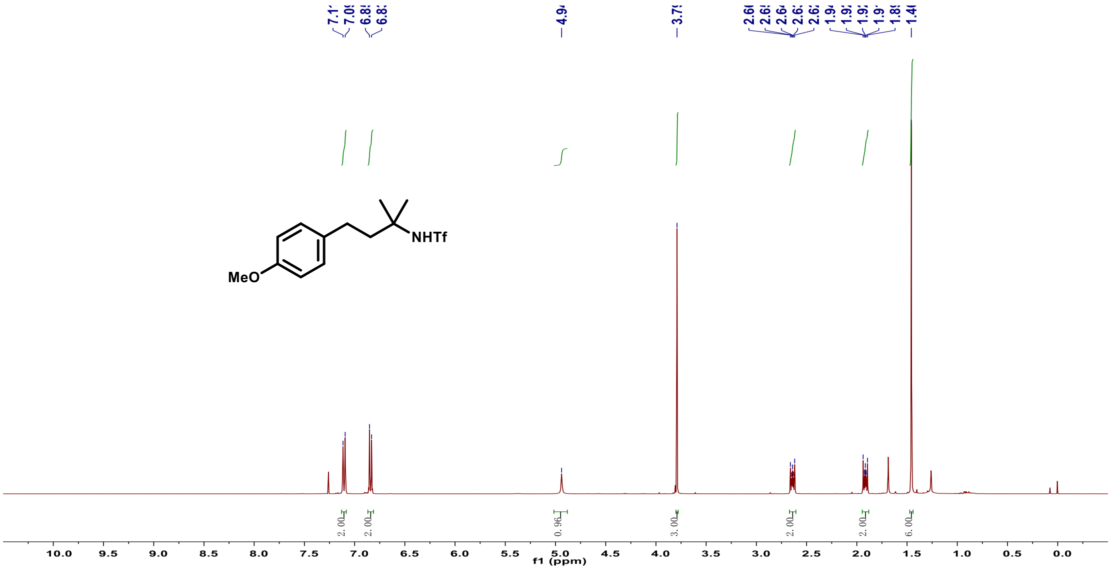

Supplementary Figure 27. ^{1}H NMR (400 MHz, $CDCl_3$) spectrum for 2

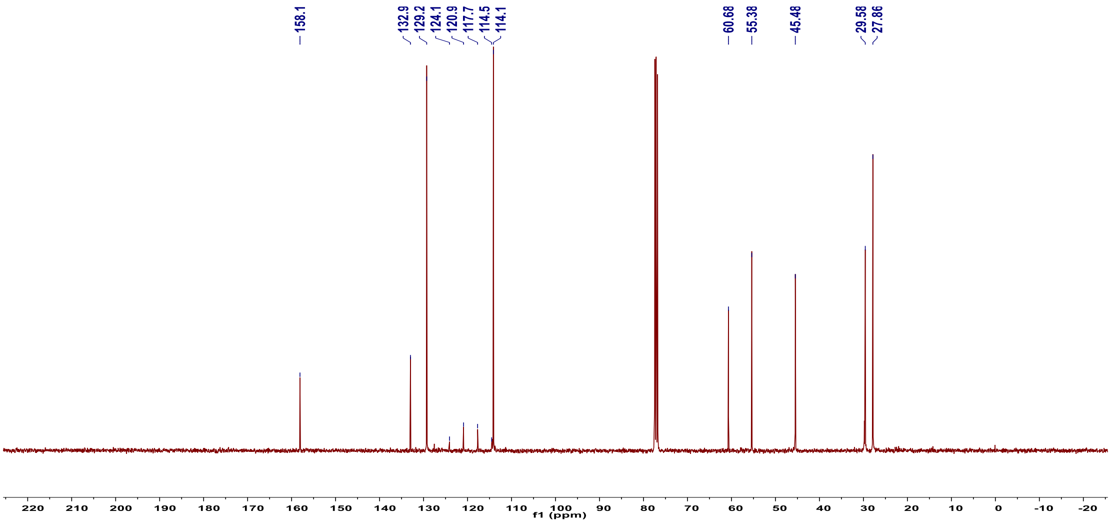

Supplementary Figure 28. ^{13}C NMR (100 MHz, $CDCl_3$) spectrum for 3aN1

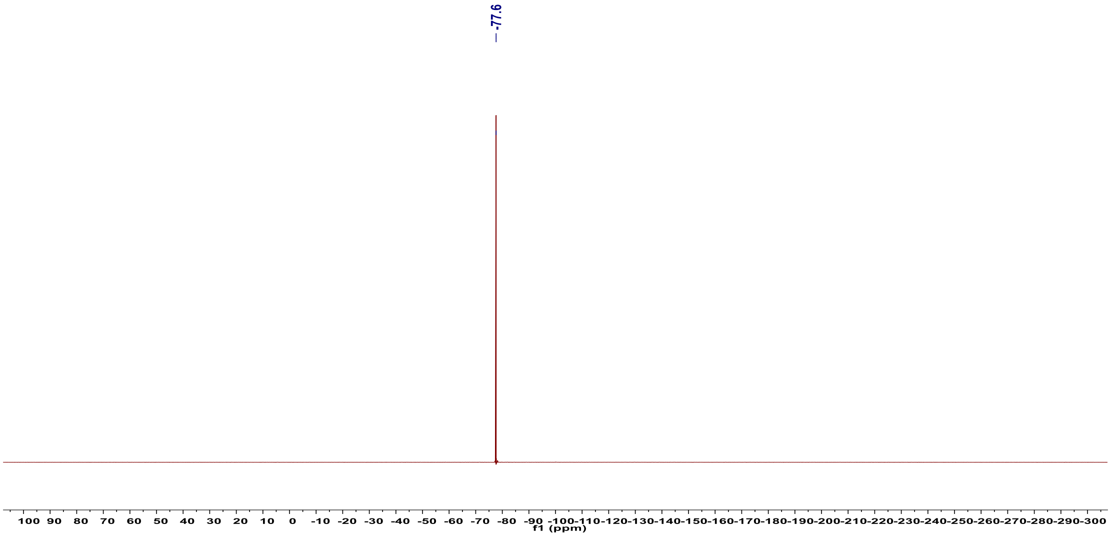

Supplementary Figure 29. ^{19}F NMR (376 MHz, $CDCl_3$) spectrum for 2

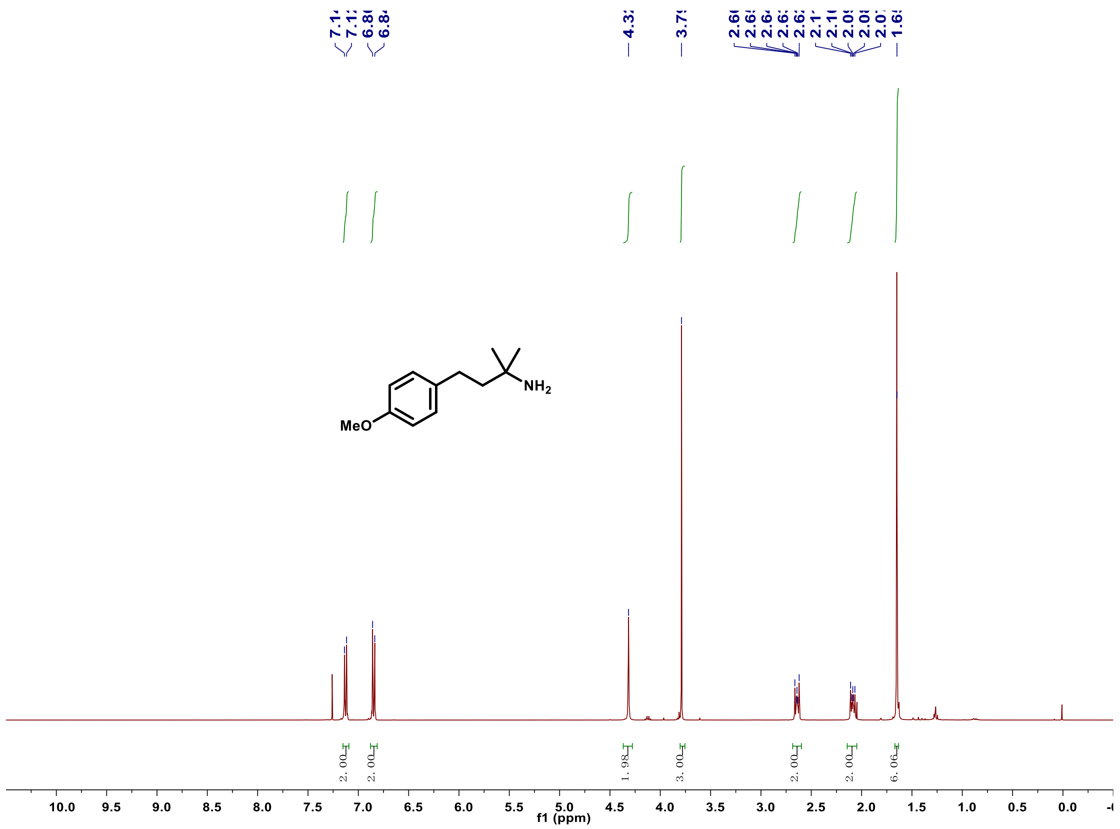

Supplementary Figure 30. ^{1}H NMR (400 MHz, $CDCl_3$) spectrum for 2'

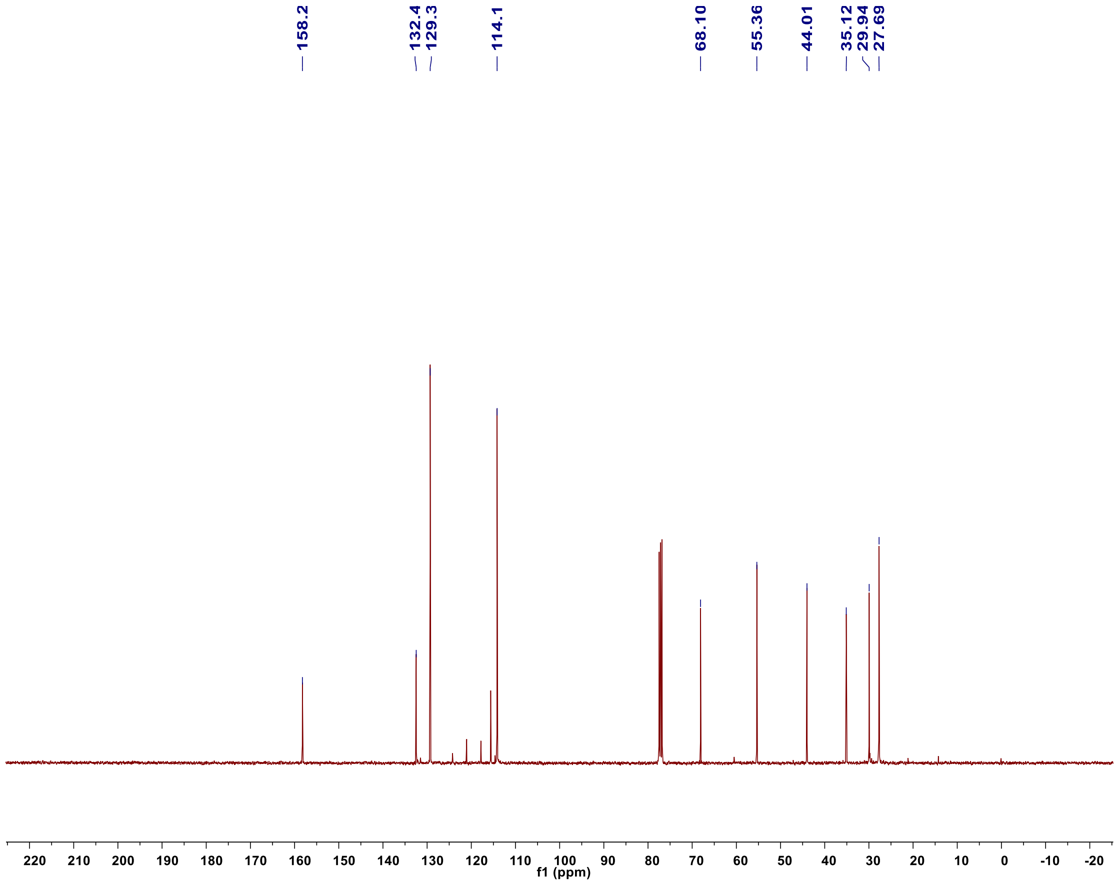

Supplementary Figure 31. ^{13}C NMR (100 MHz, $CDCl_3$) spectrum for 2'

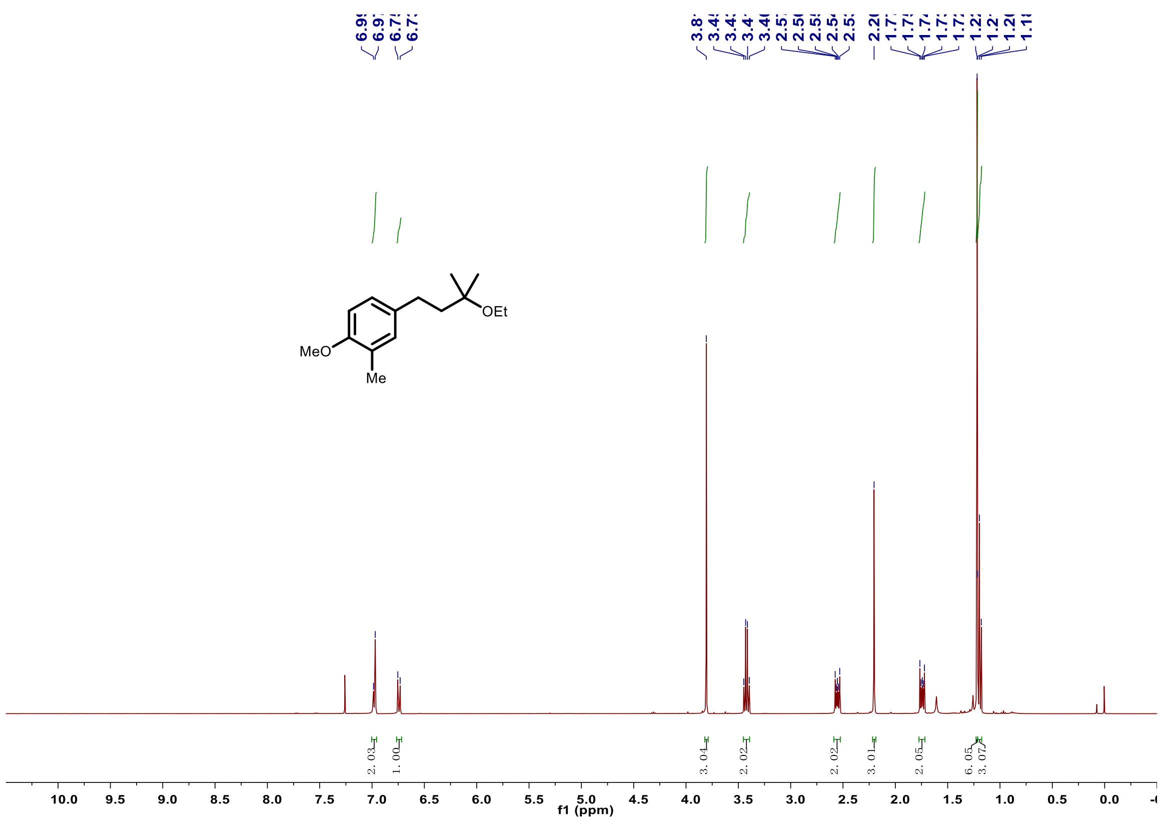

Supplementary Figure 32. ^{1}H **NMR (400 MHz,** $CDCl_3$**) spectrum for 4**

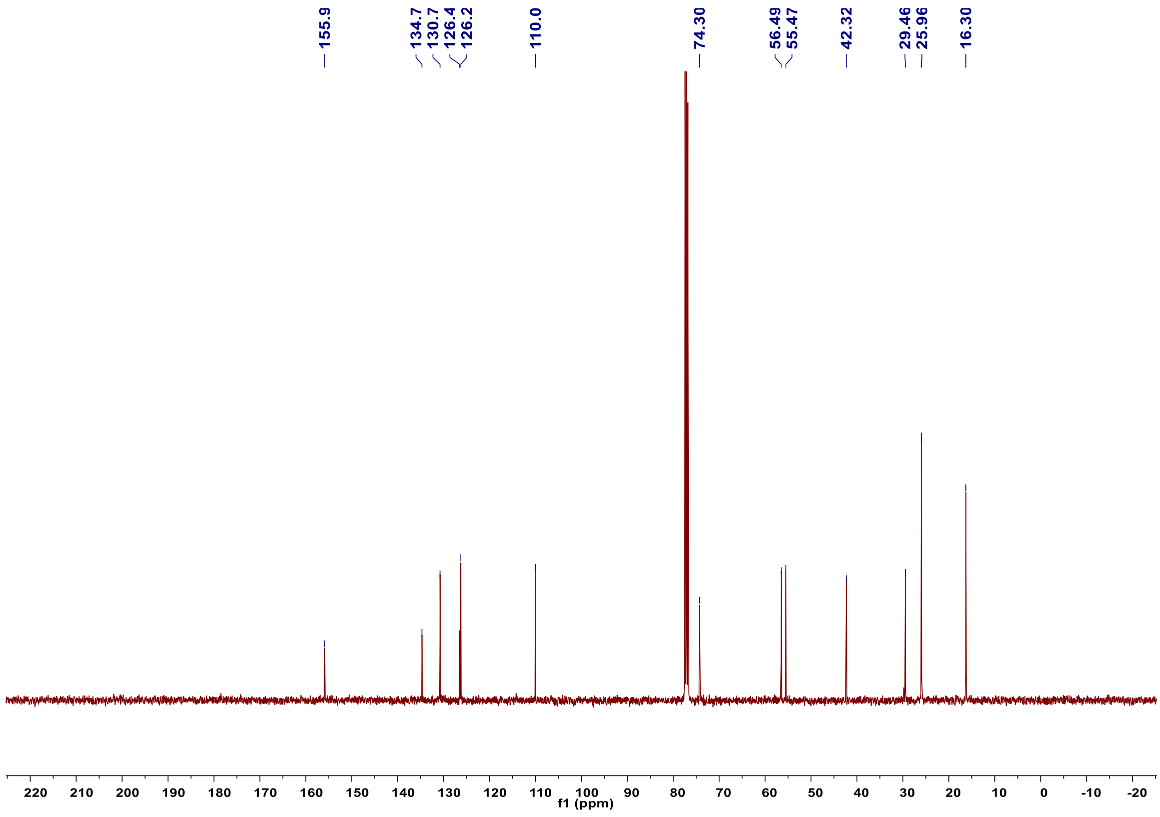

Supplementary Figure 33. ^{13}C **NMR (100 MHz,** $CDCl_3$**) spectrum for 4**

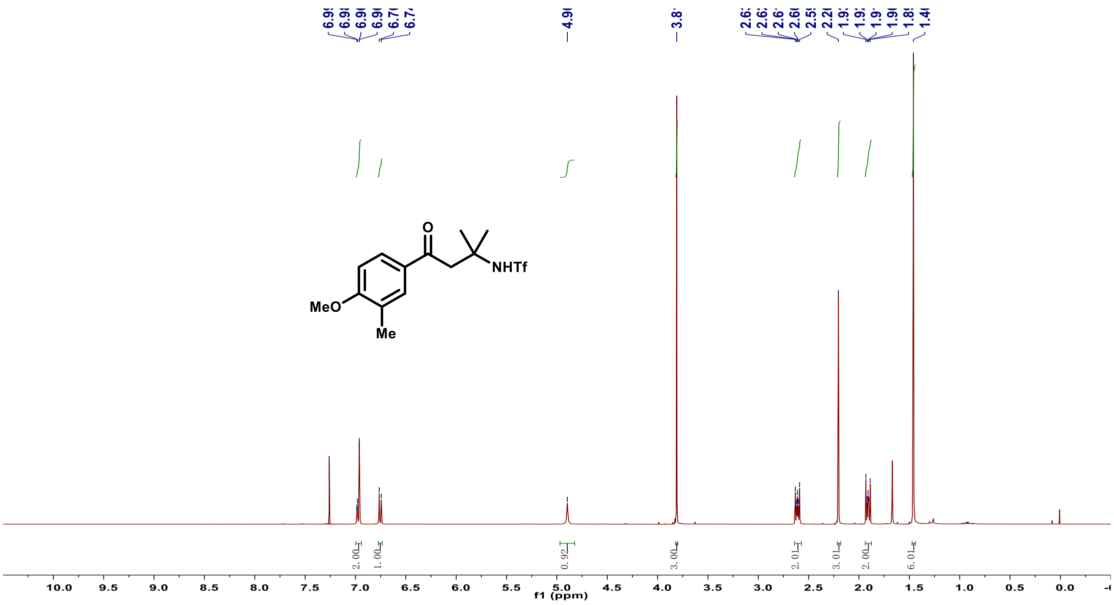

Supplementary Figure 34. ^{1}H NMR (400 MHz, $CDCl_3$) spectrum for 28

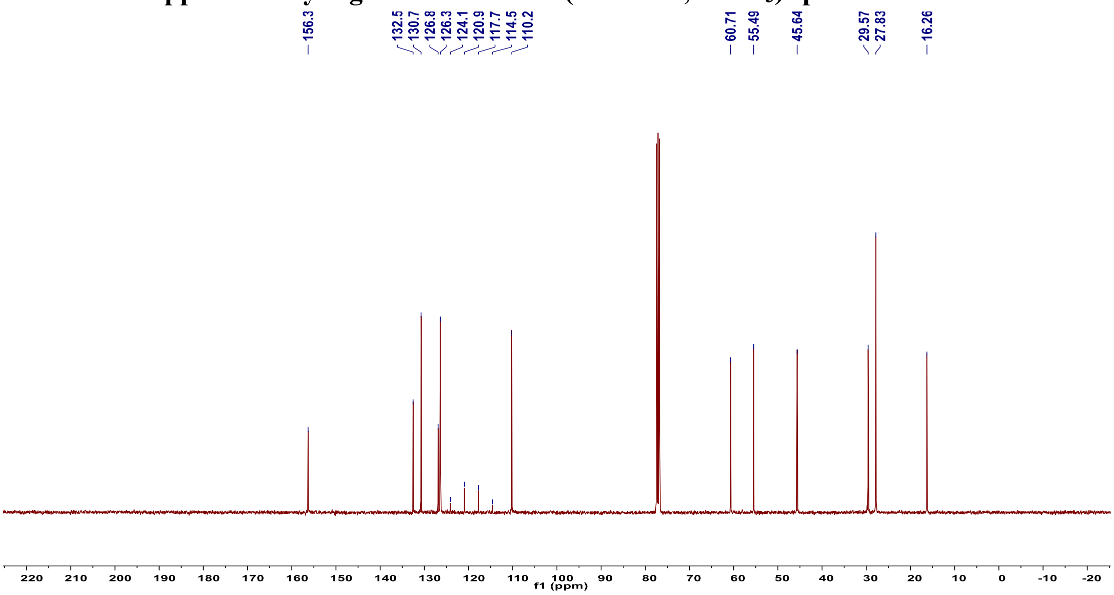

Supplementary Figure 35. ^{13}C NMR (100 MHz, $CDCl_3$) spectrum for 28

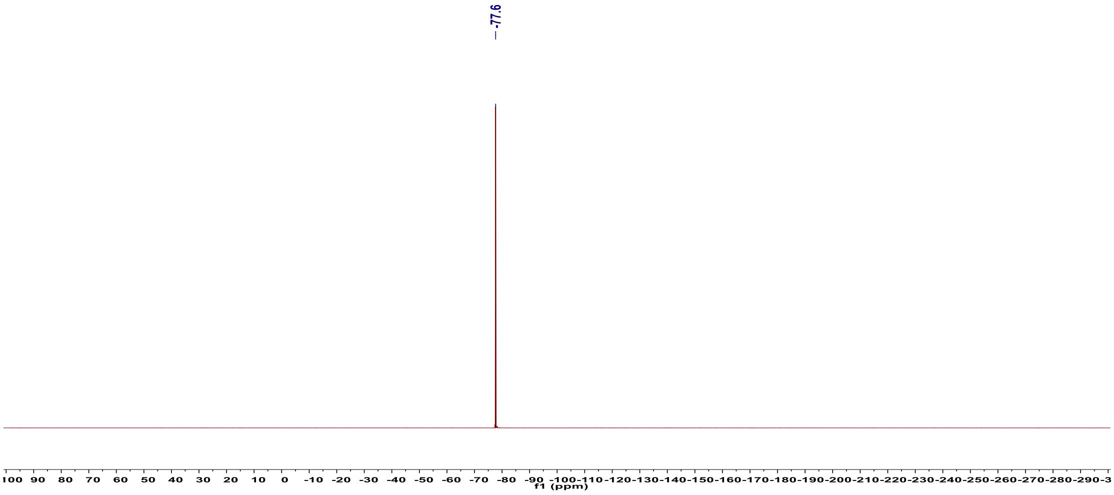

Supplementary Figure 36. ^{19}F NMR (100 MHz, $CDCl_3$) spectrum for 28

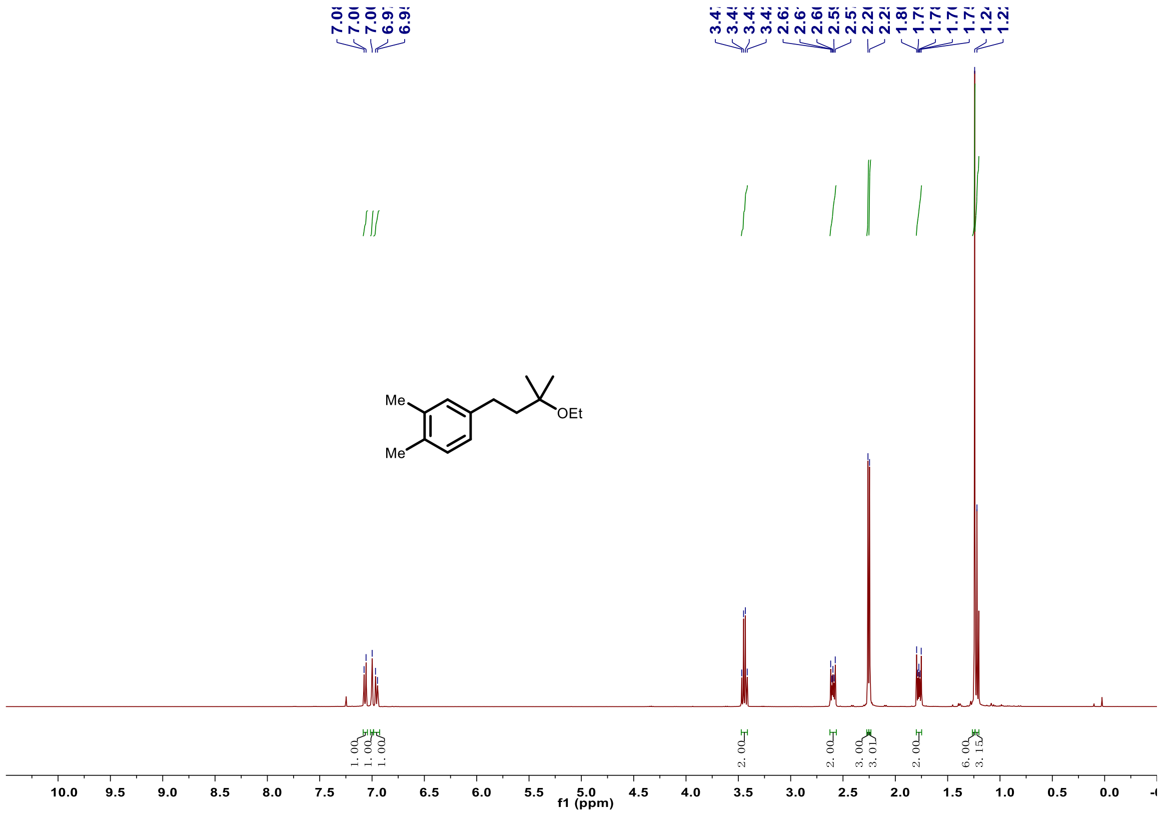

Supplementary Figure 37. ^{1}H NMR (400 MHz, $CDCl_3$) spectrum for 5

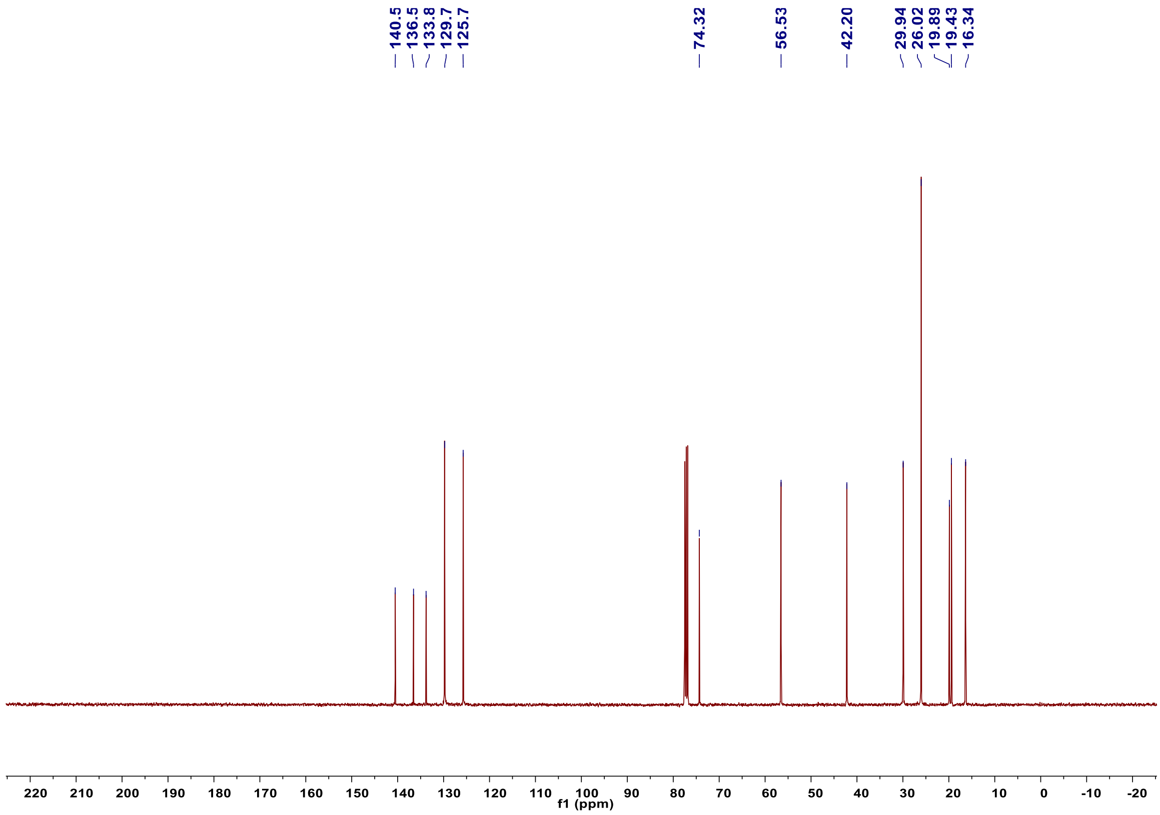

Supplementary Figure 38. ^{13}C NMR (100 MHz, $CDCl_3$) spectrum for 5

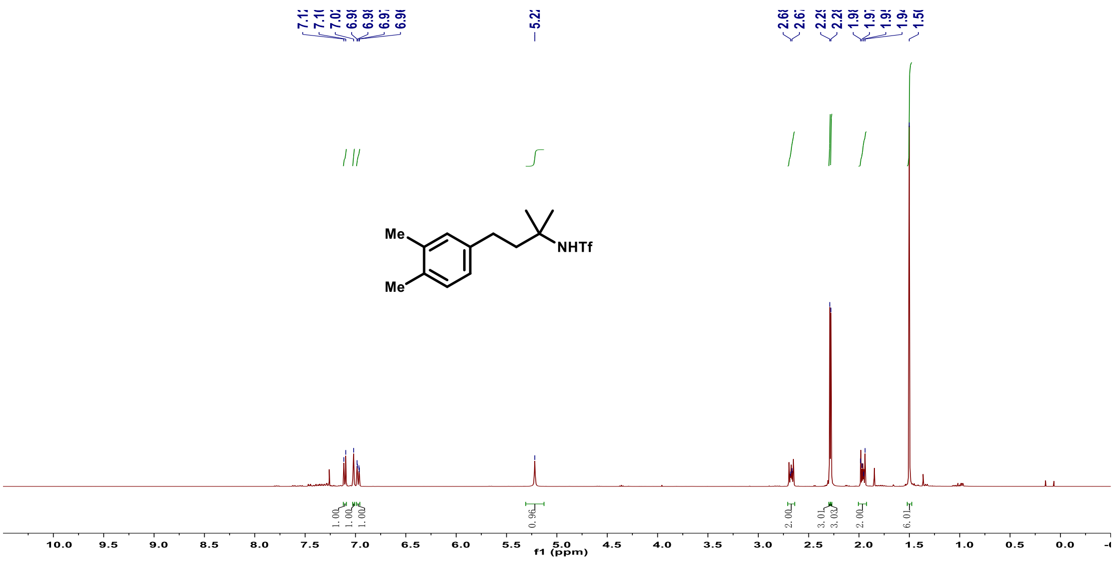

Supplementary Figure 39. ^{1}H **NMR (400 MHz,** $CDCl_3$**) spectrum for 30**

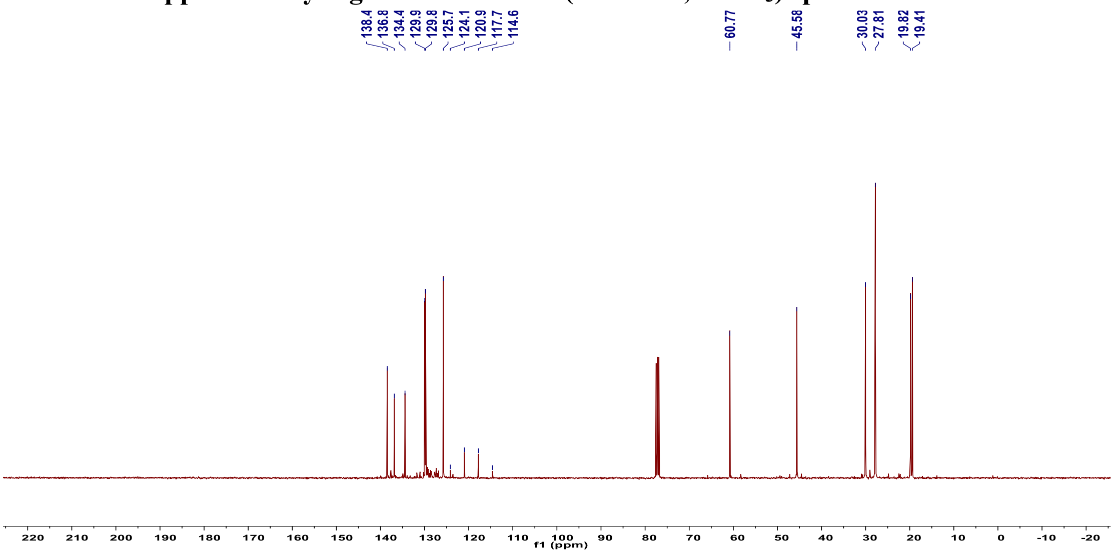

Supplementary Figure 40. ^{13}C **NMR (100 MHz,** $CDCl_3$**) spectrum for 30**

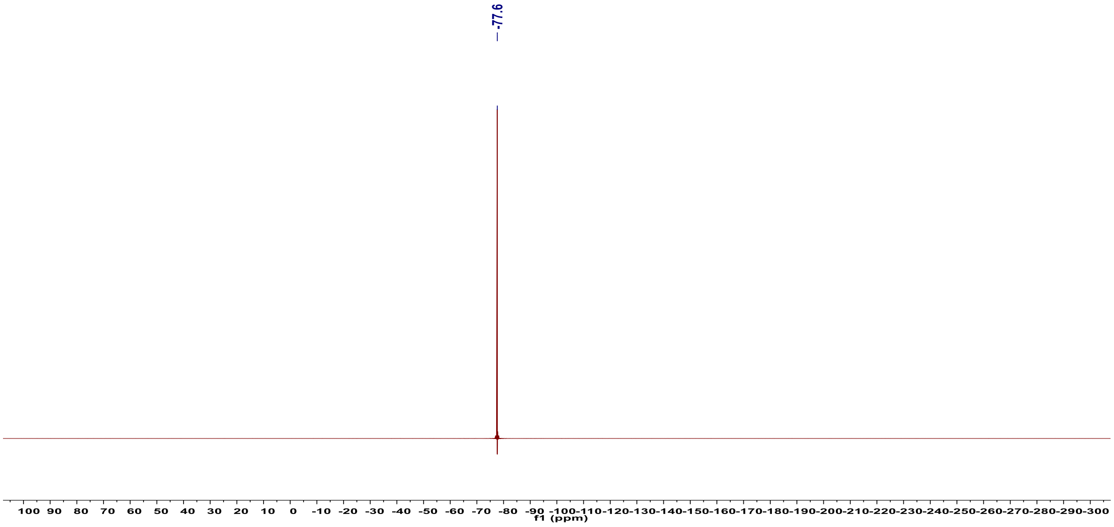

Supplementary Figure 41. ^{19}F **NMR (376 MHz,** $CDCl_3$**) spectrum for 30**

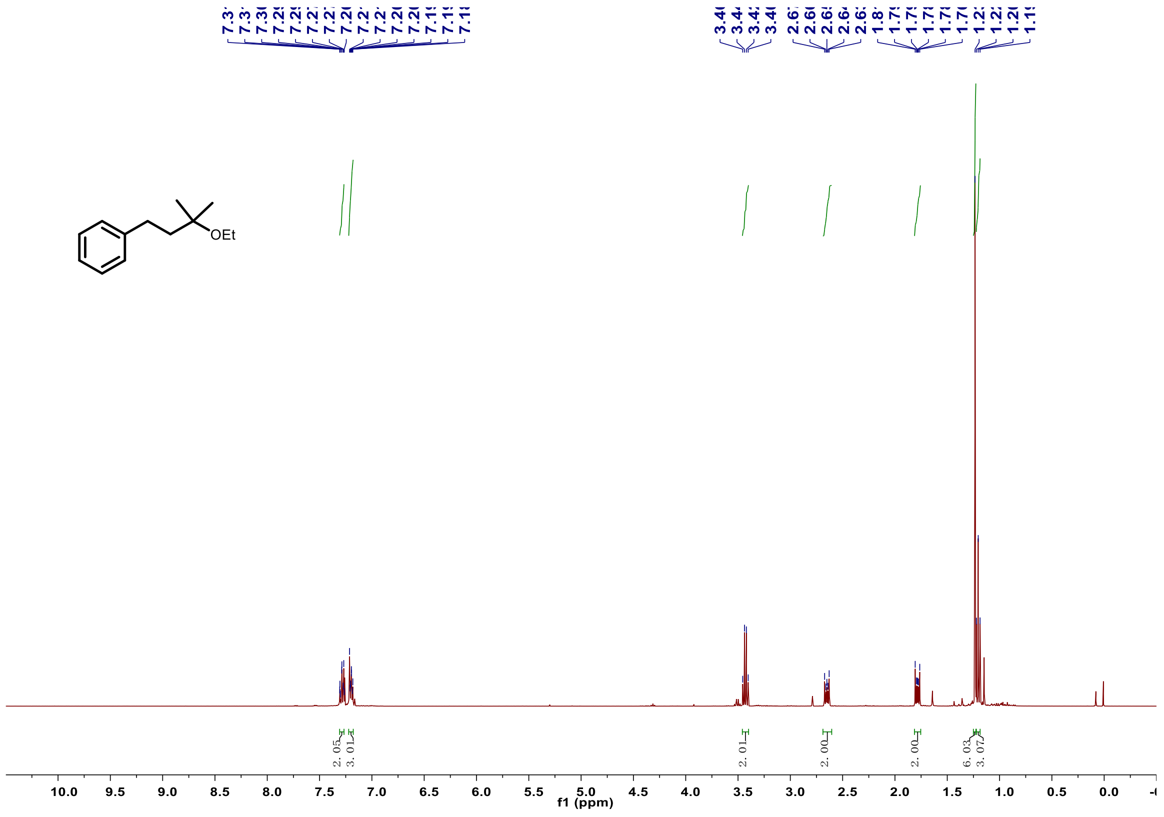

Supplementary Figure 42. ^{1}H NMR (400 MHz, $CDCl_3$) spectrum for 6

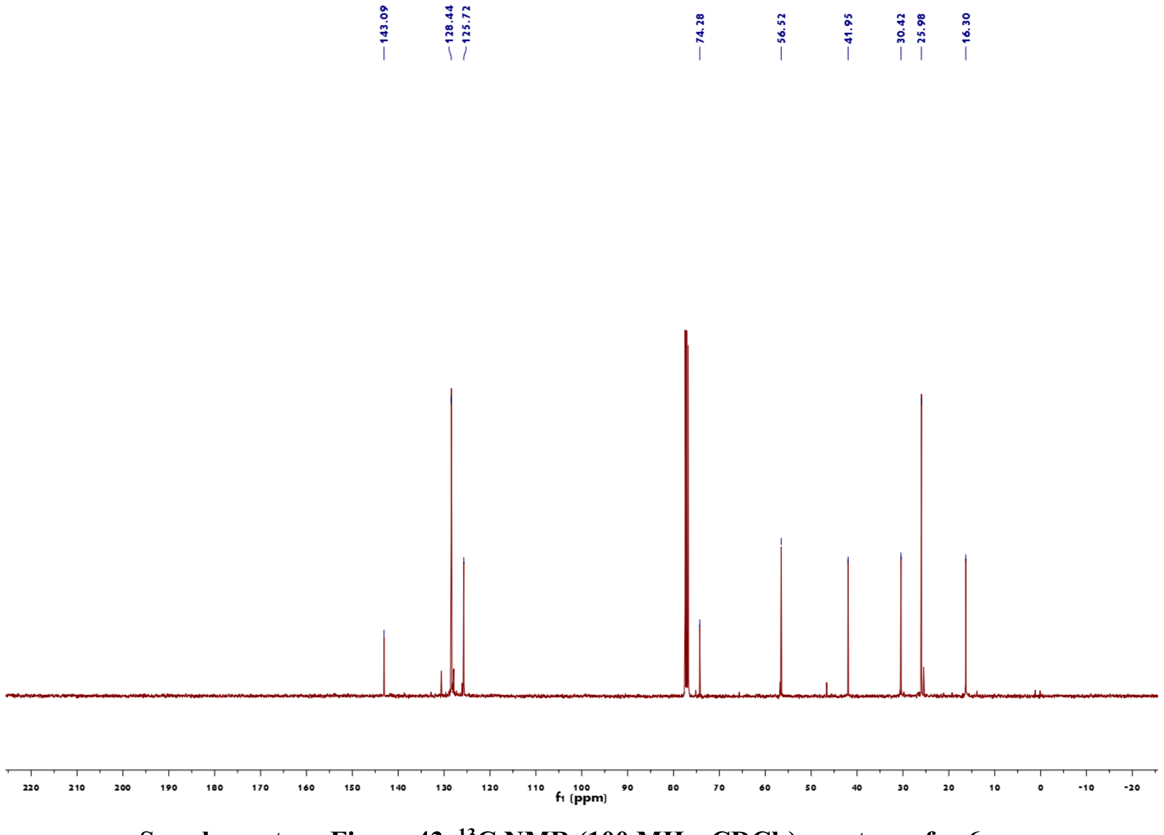

Supplementary Figure 43. ^{13}C NMR (100 MHz, $CDCl_3$) spectrum for 6

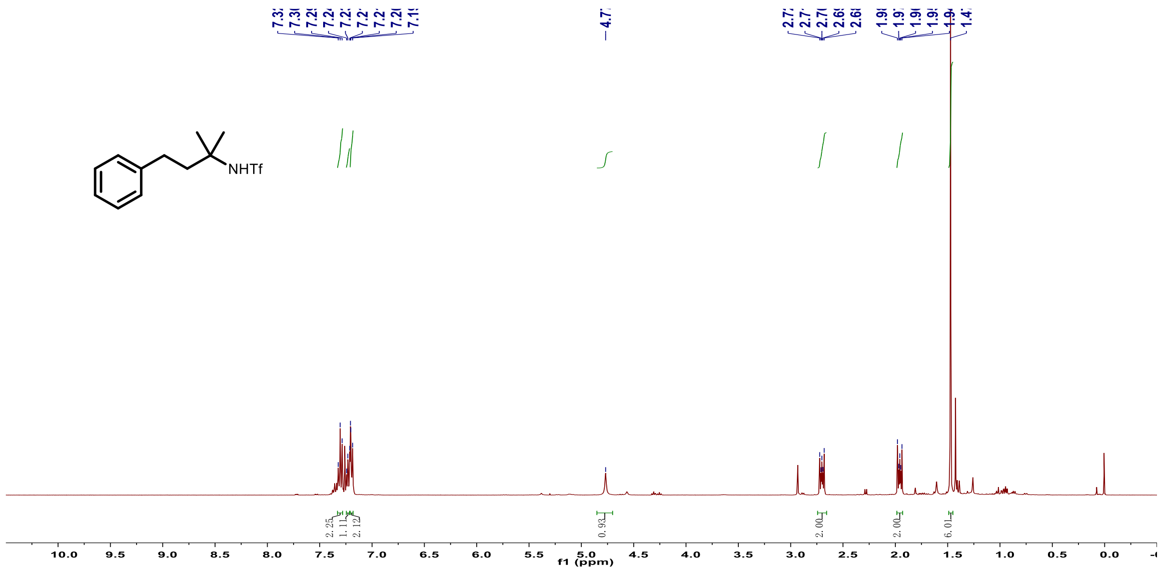

Supplementary Figure 44. **^{1}H NMR (400 MHz, CDCl$_3$) spectrum for 31**

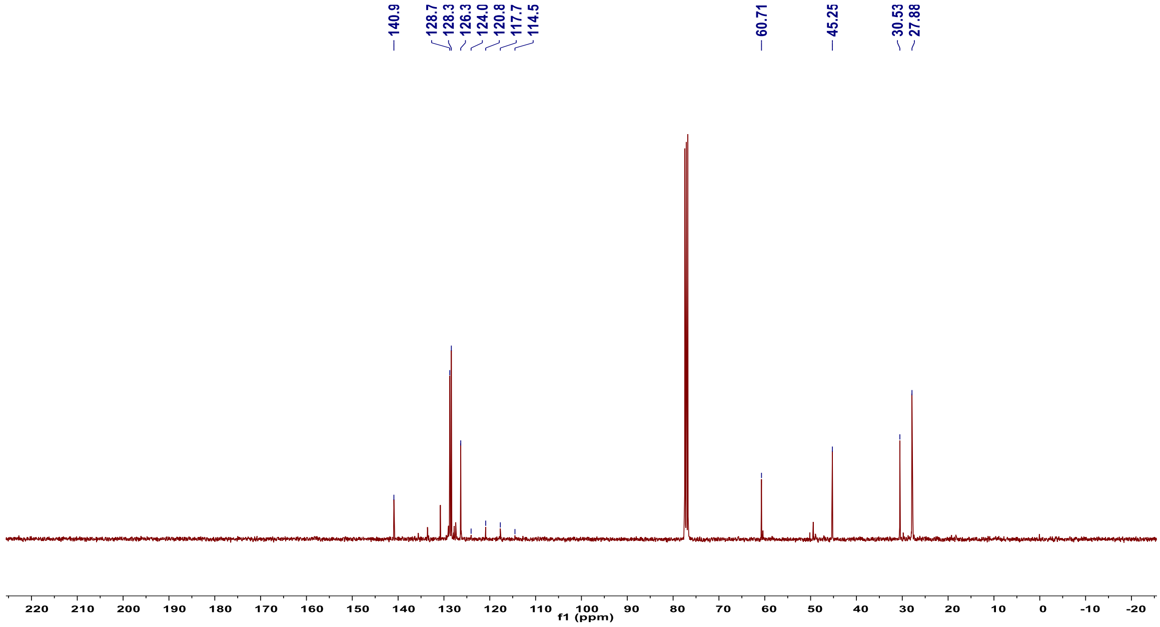

Supplementary Figure 45. **^{13}C NMR (100 MHz, CDCl$_3$) spectrum for 31**

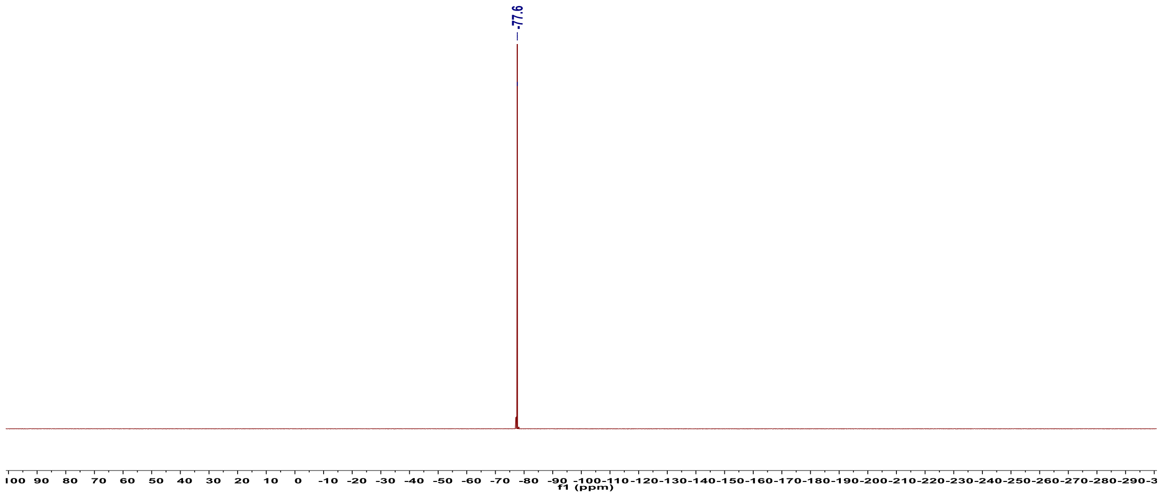

Supplementary Figure 46. **^{19}F NMR (377 MHz, CDCl$_3$) spectrum for 31**

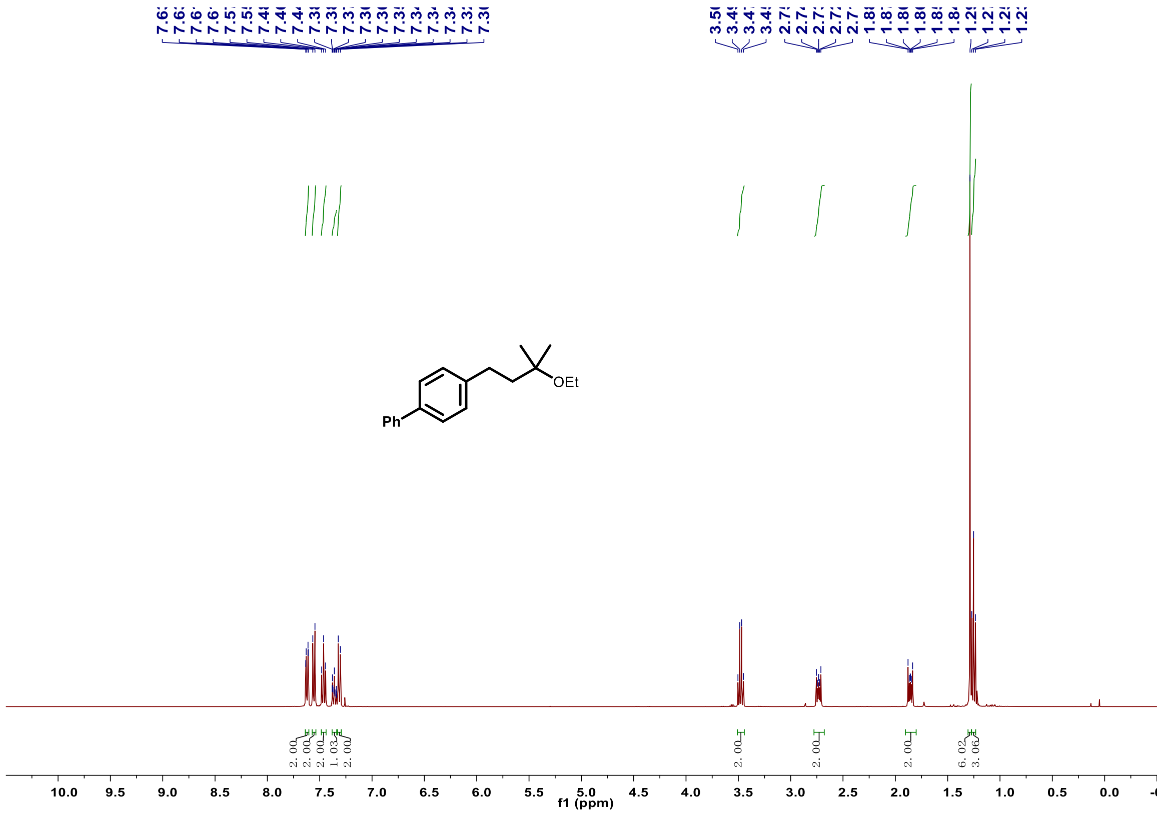

Supplementary Figure 47. **^{1}H NMR (400 MHz, $CDCl_3$) spectrum for 7**

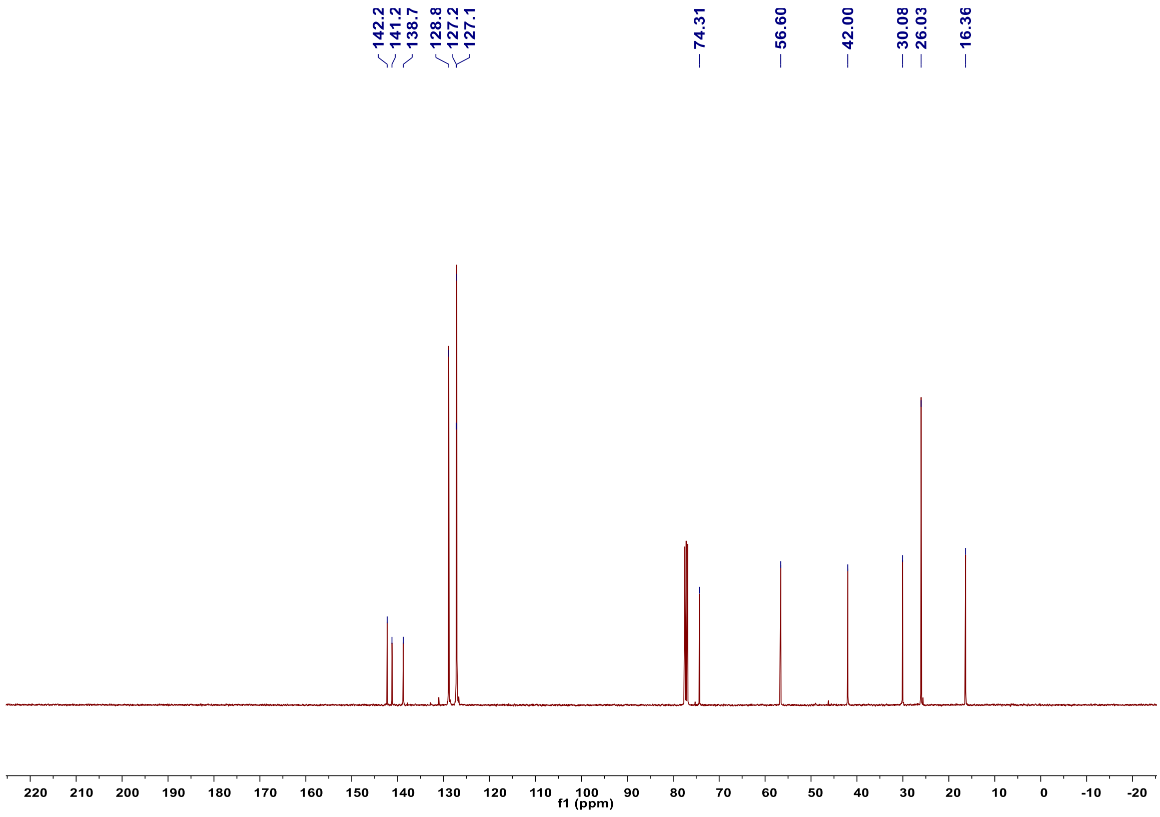

Supplementary Figure 48. **^{13}C NMR (100 MHz, $CDCl_3$) spectrum for 7**

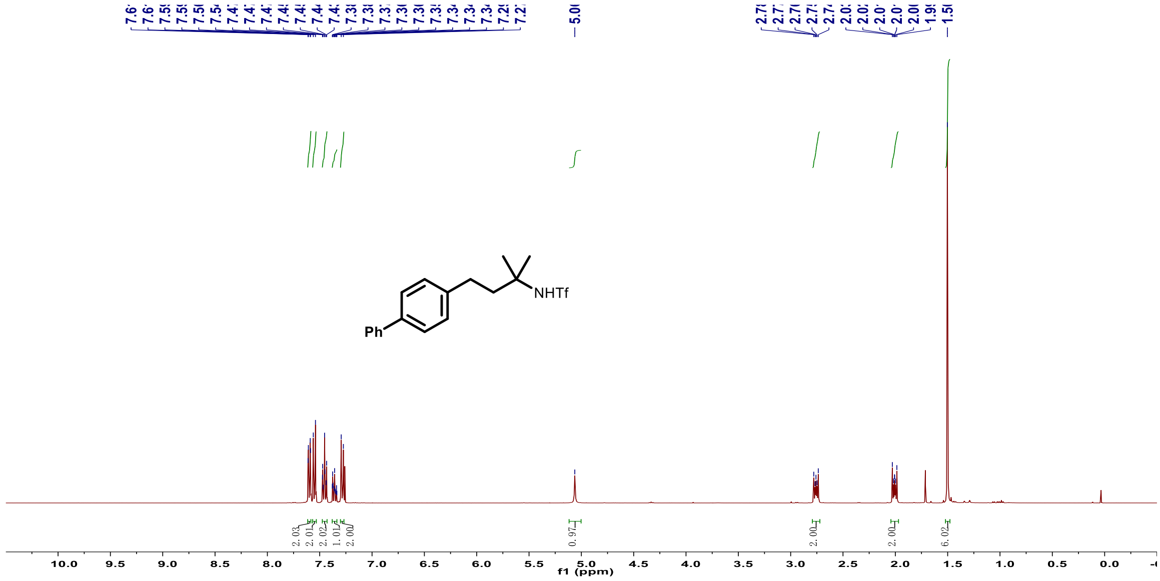

Supplementary Figure 49. ^{1}H NMR (400 MHz, $CDCl_3$) spectrum for 32

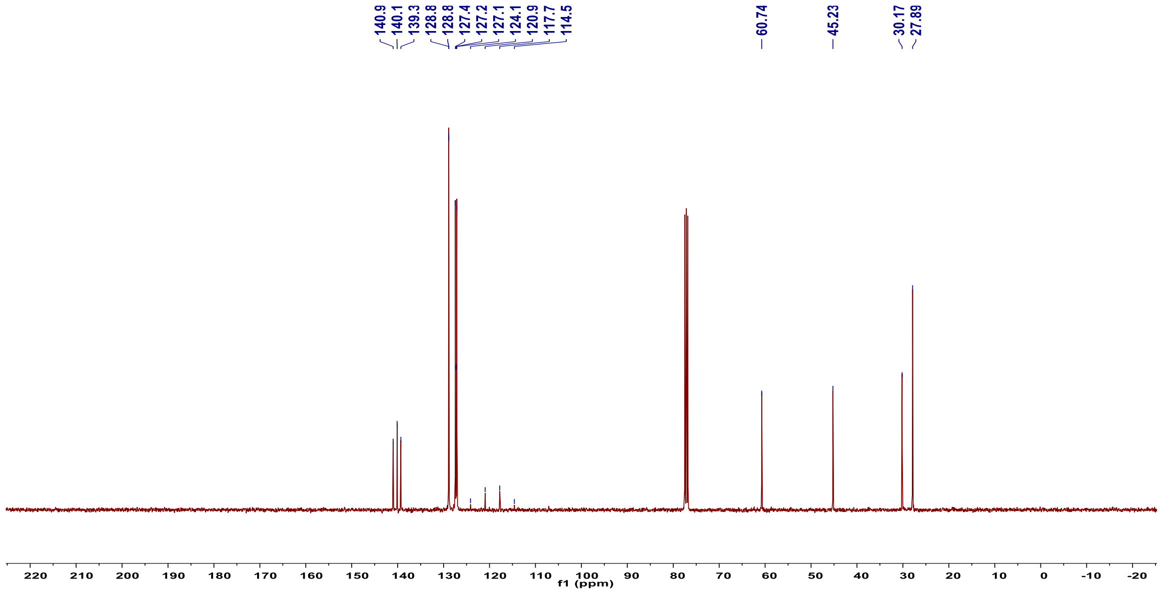

Supplementary Figure 50. ^{13}C NMR (100 MHz, $CDCl_3$) spectrum for 32

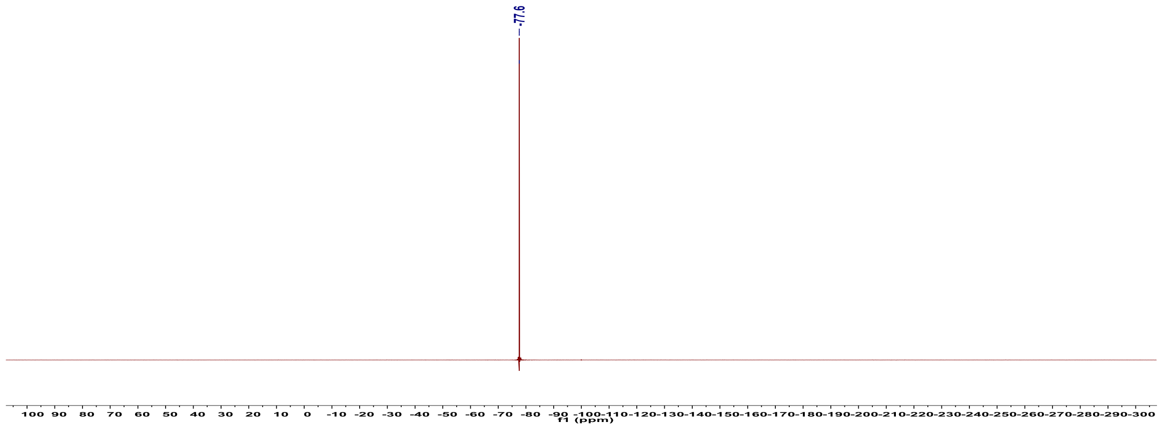

Supplementary Figure 51. ^{19}F NMR (377 MHz, $CDCl_3$) spectrum for 32

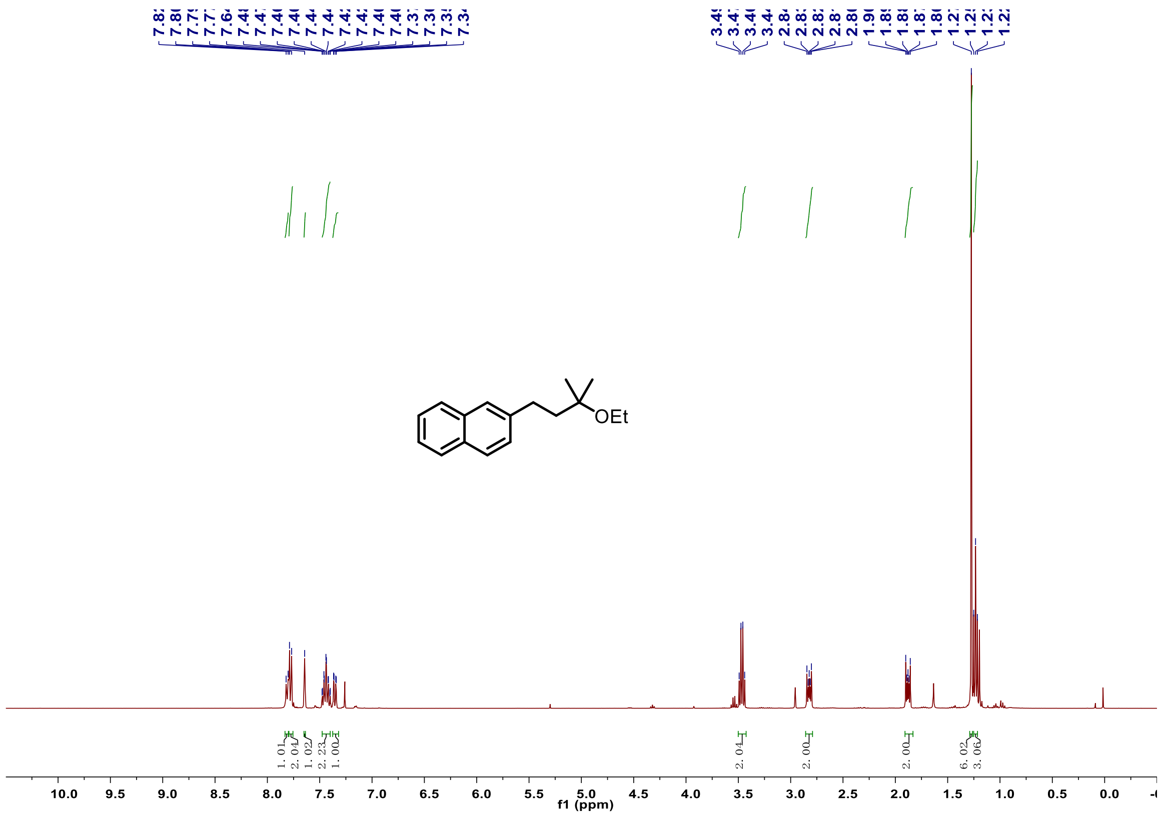

Supplementary Figure 52. ^{1}H NMR (400 MHz, $CDCl_3$) spectrum for **8**

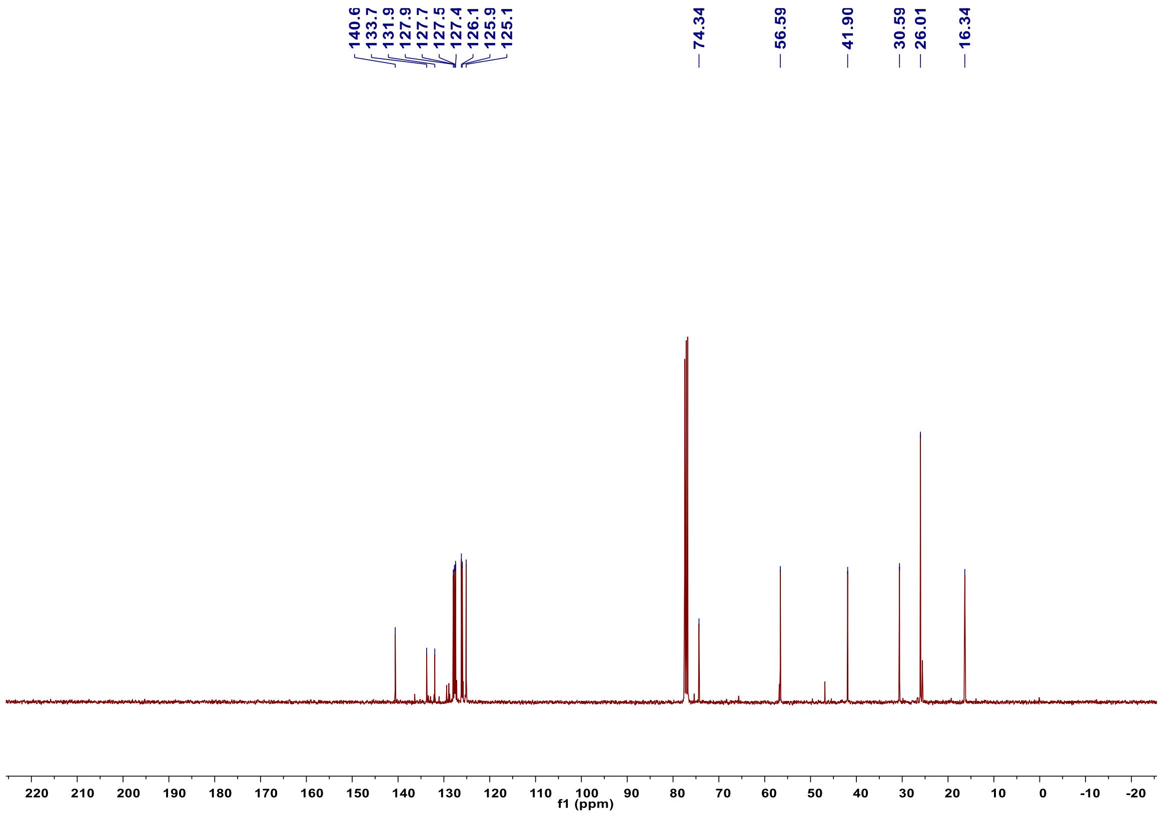

Supplementary Figure 53. ^{13}C NMR (100 MHz, $CDCl_3$) spectrum for **8**

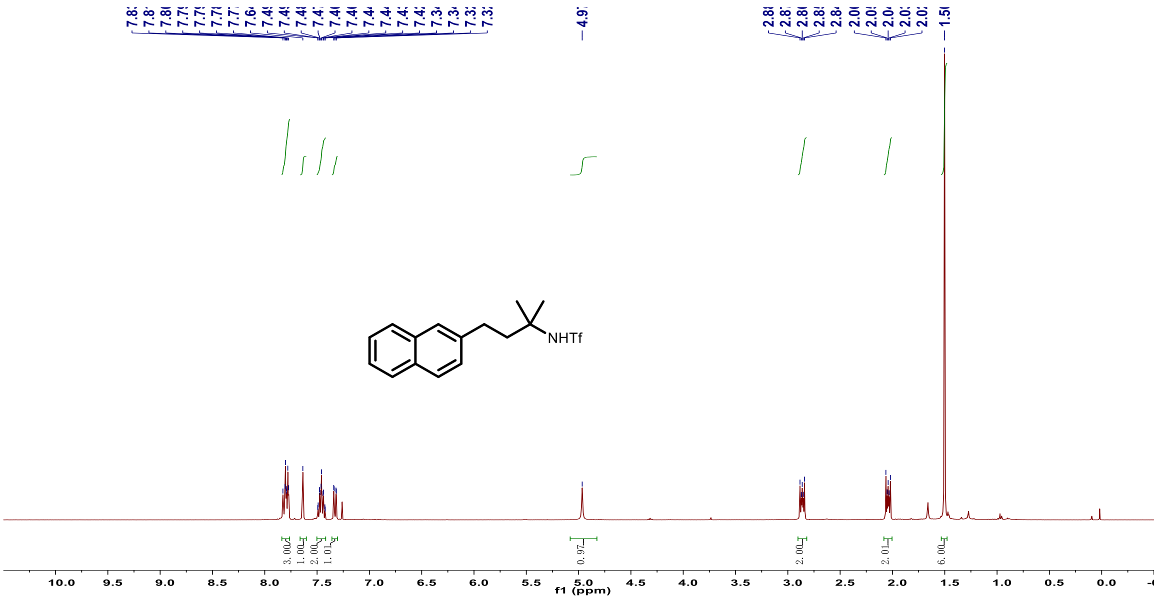

Supplementary Figure 54. ^{1}H **NMR (400 MHz,** $CDCl_3$**) spectrum for 33**

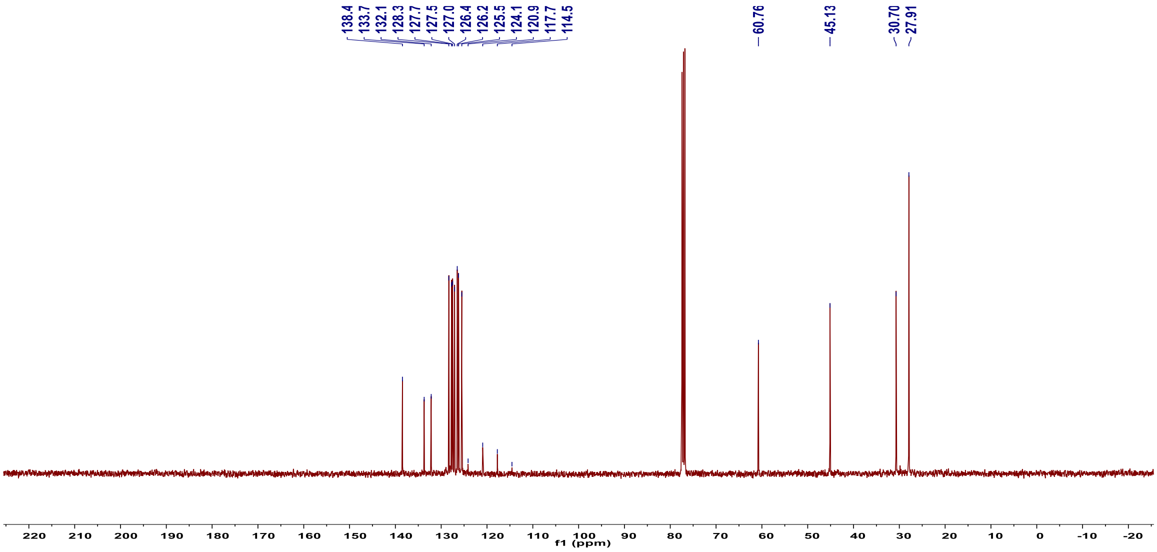

Supplementary Figure 55. ^{13}C **NMR (100 MHz,** $CDCl_3$**) spectrum for 33**

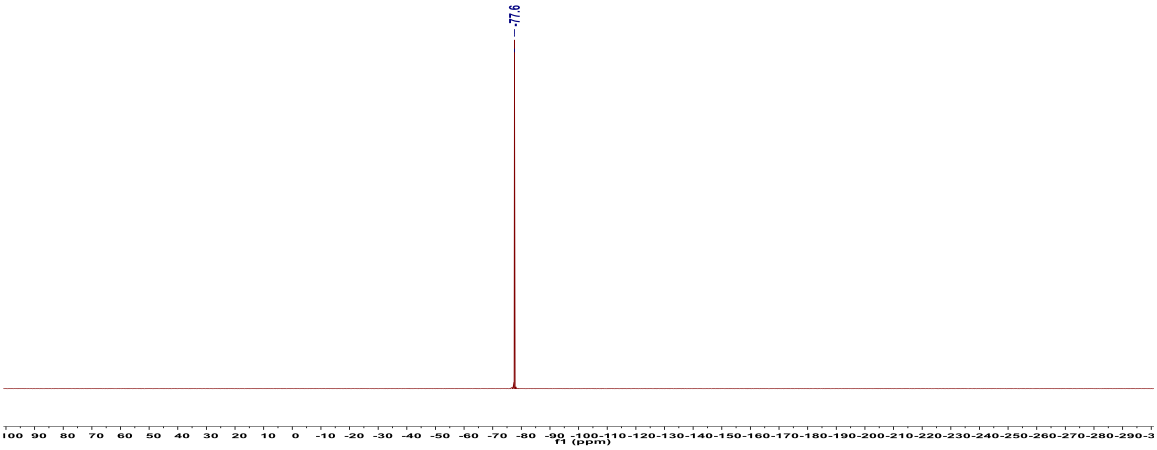

Supplementary Figure 56. ^{19}F **NMR (377 MHz,** $CDCl_3$**) spectrum for 33**

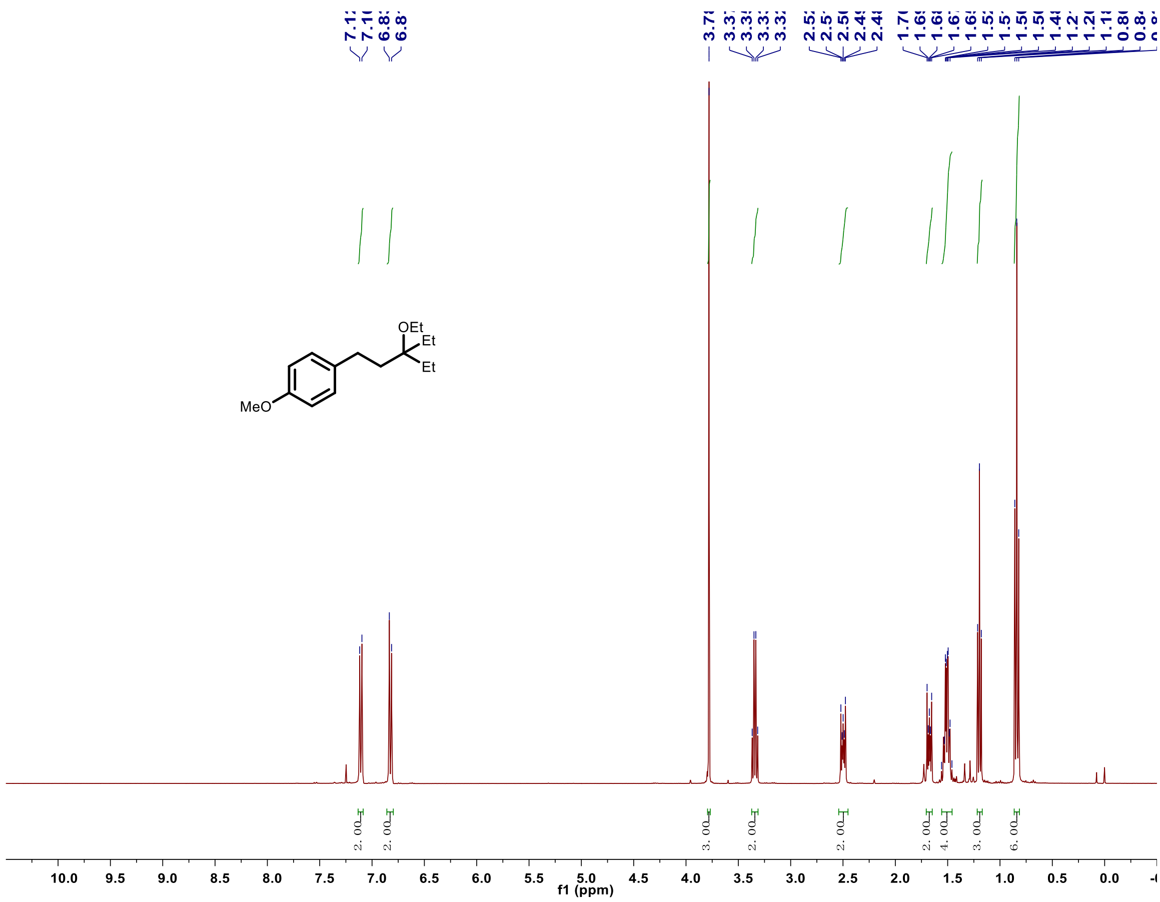

Supplementary Figure 57. **^{1}H NMR (400 MHz, $CDCl_3$) spectrum for 9**

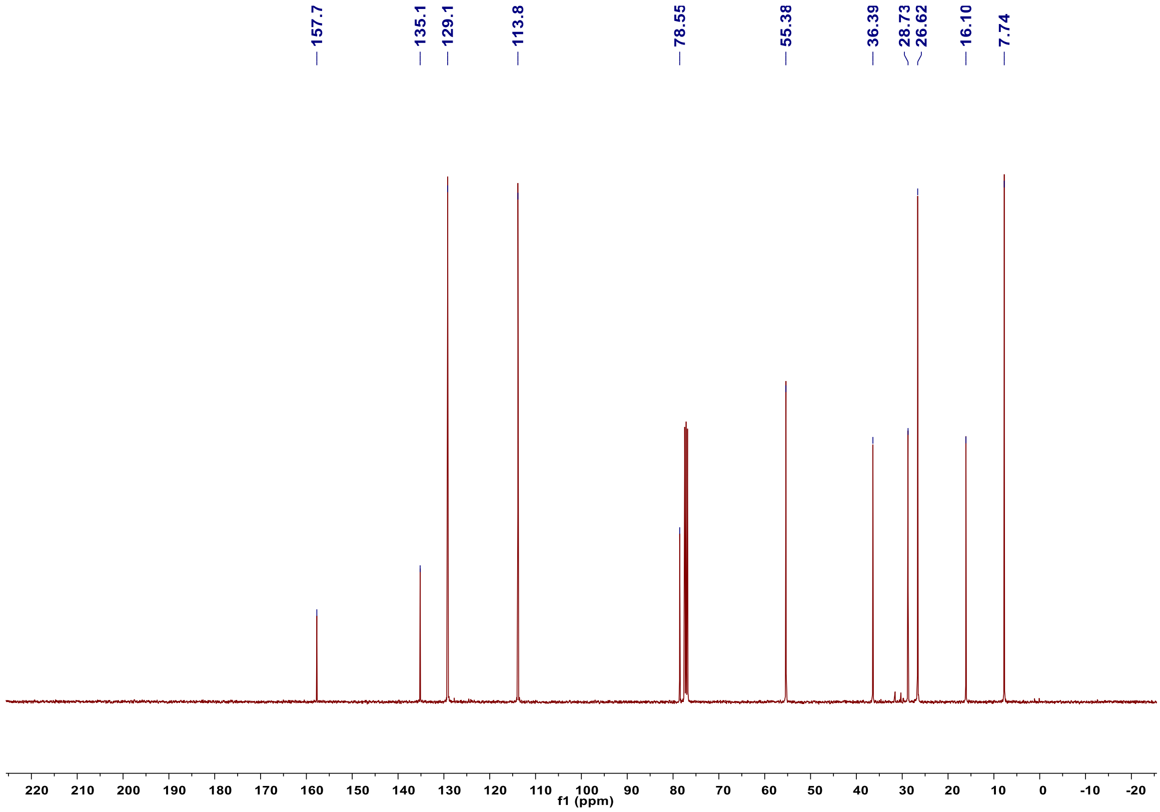

Supplementary Figure 58. **^{13}C NMR (100 MHz, $CDCl_3$) spectrum for 9**

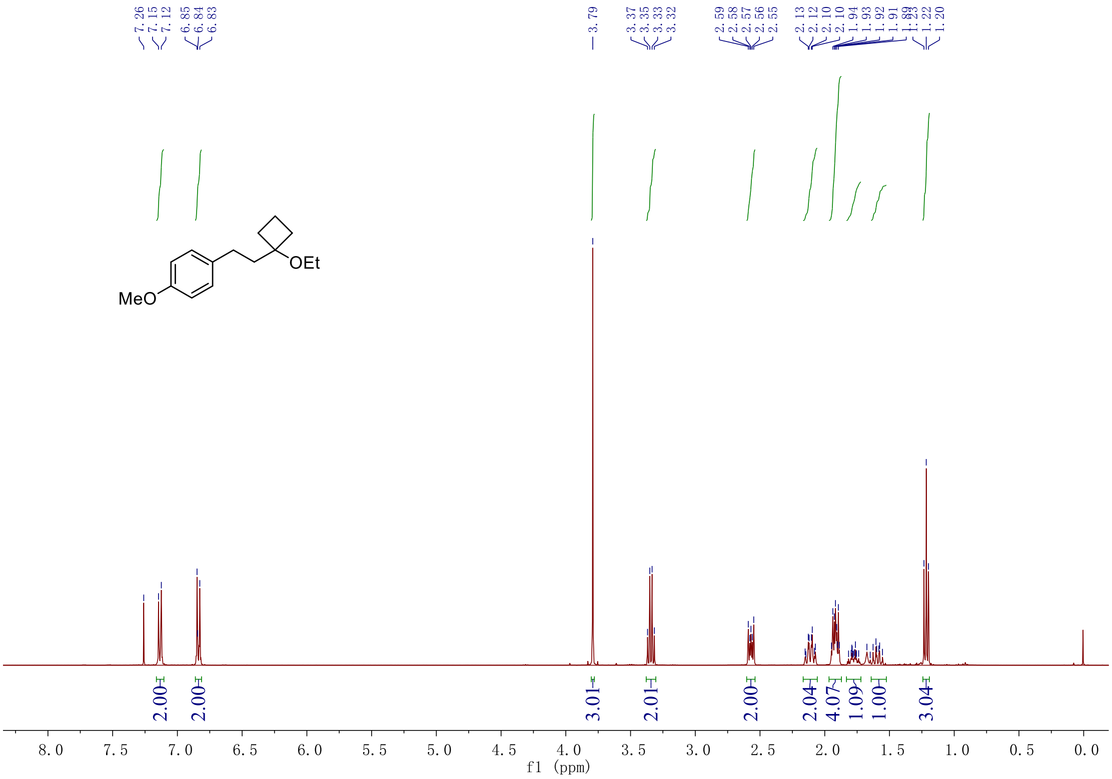

Supplementary Figure 59. ^{1}H NMR (400 MHz, $CDCl_3$) spectrum for 10

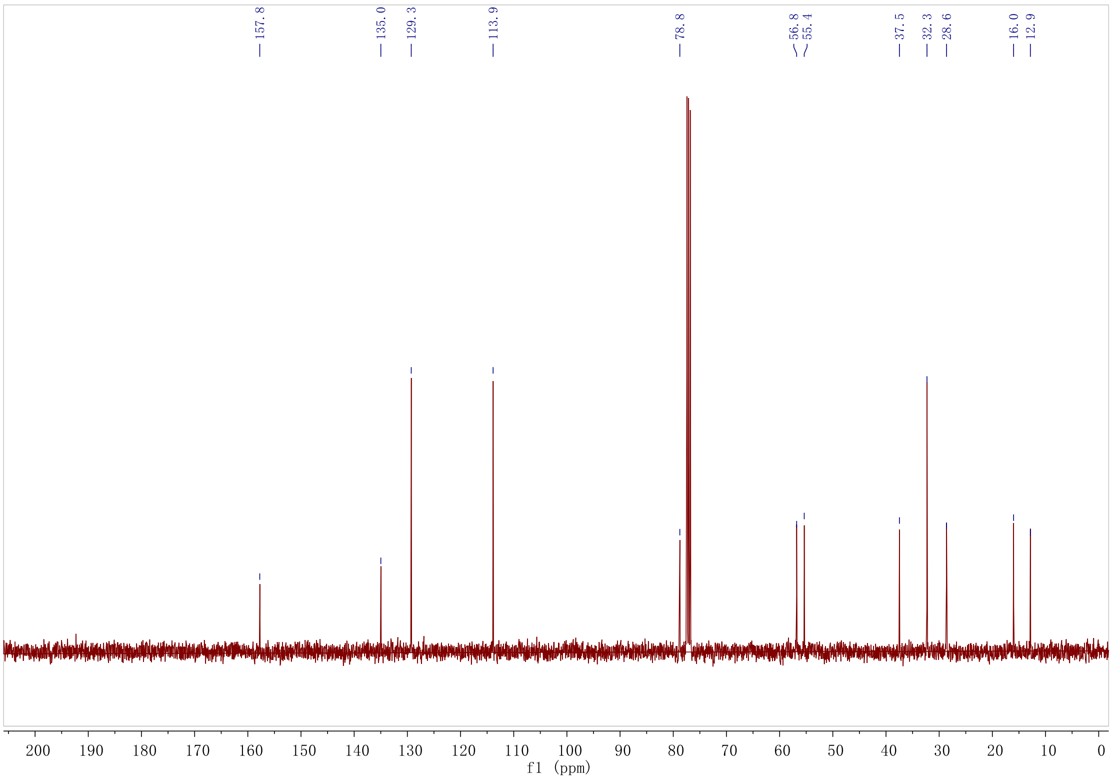

Supplementary Figure 60. ^{13}C NMR (100 MHz, $CDCl_3$) spectrum for 10

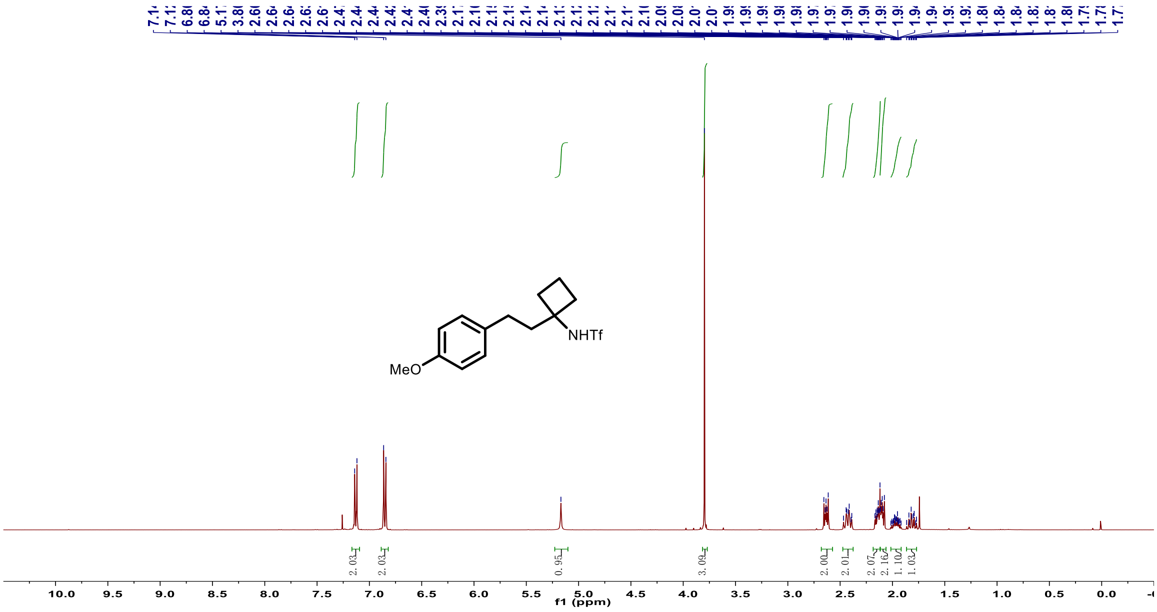

Supplementary Figure 61. ^{1}**H NMR (400 MHz, CDCl$_3$) spectrum for 34**

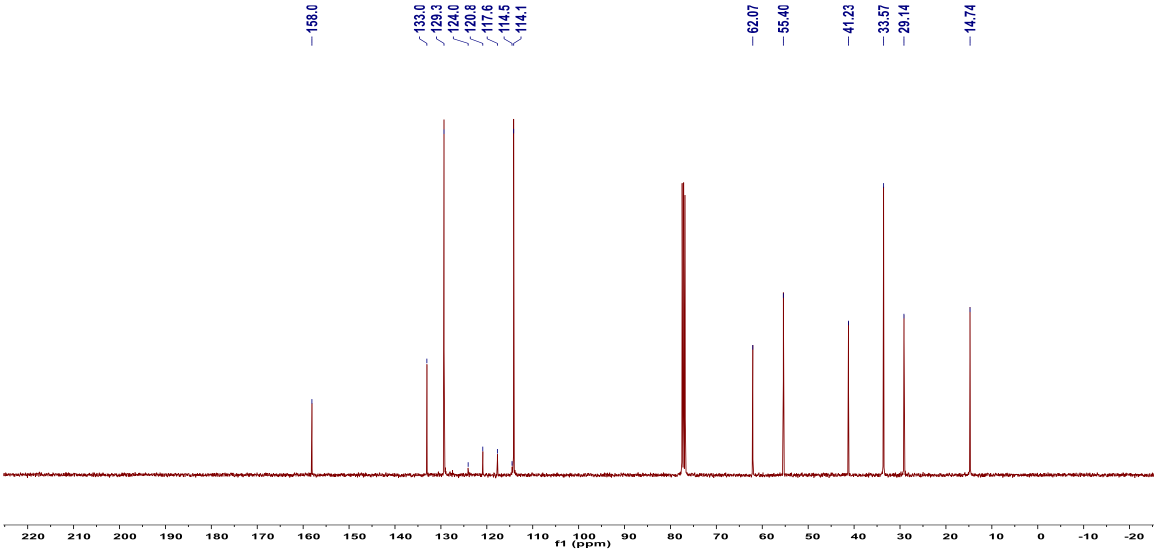

Supplementary Figure 62. ^{13}**C NMR (100 MHz, CDCl$_3$) spectrum for 34**

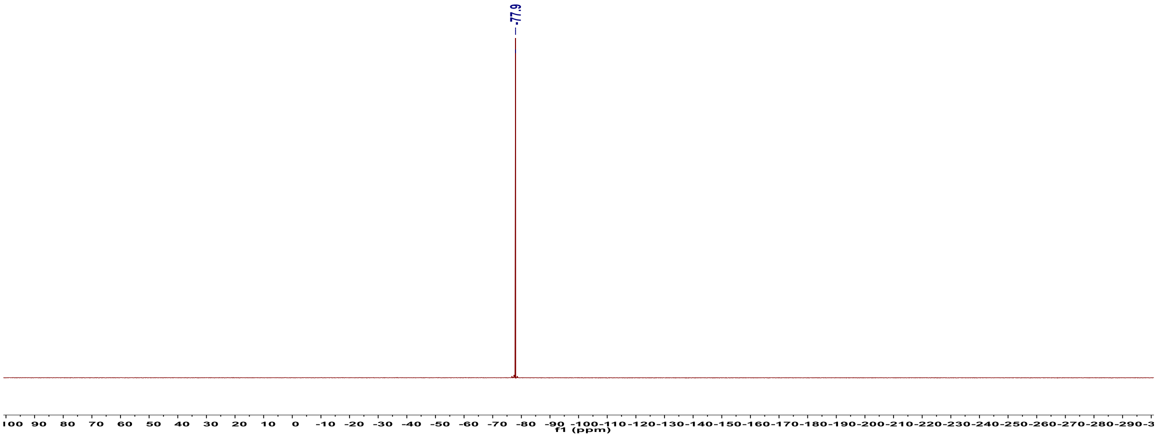

Supplementary Figure 63. ^{19}**F NMR (377 MHz, CDCl$_3$) spectrum for 34**

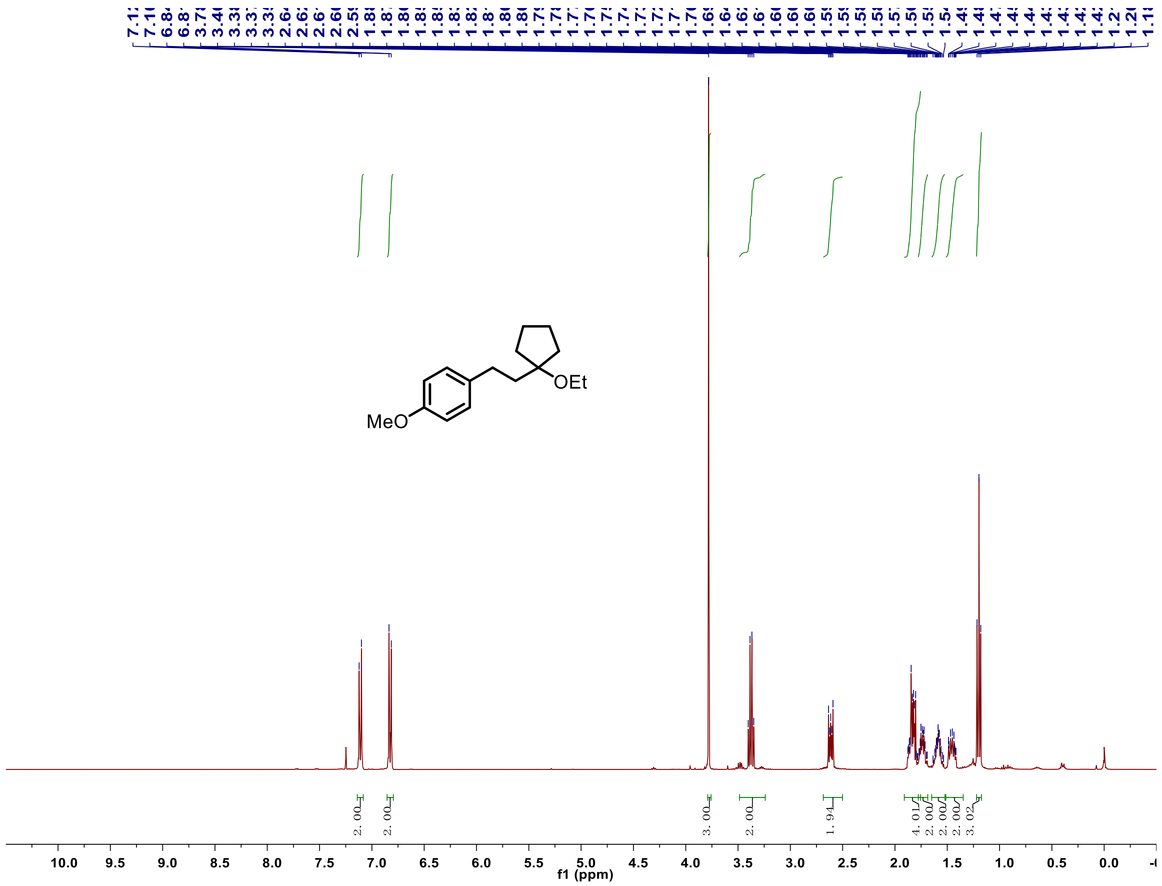

Supplementary Figure 64. ^{1}H NMR (400 MHz, $CDCl_3$) spectrum for 11

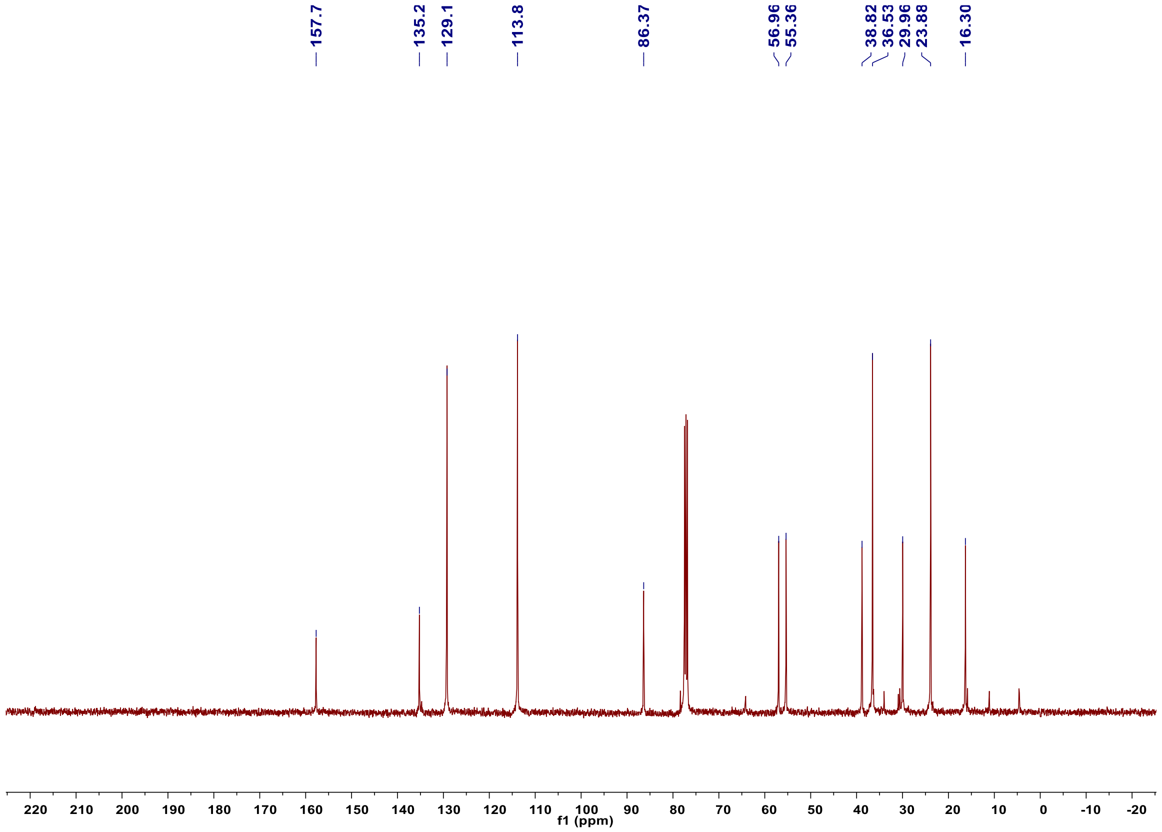

Supplementary Figure 65. ^{13}C NMR (100 MHz, $CDCl_3$) spectrum for 11

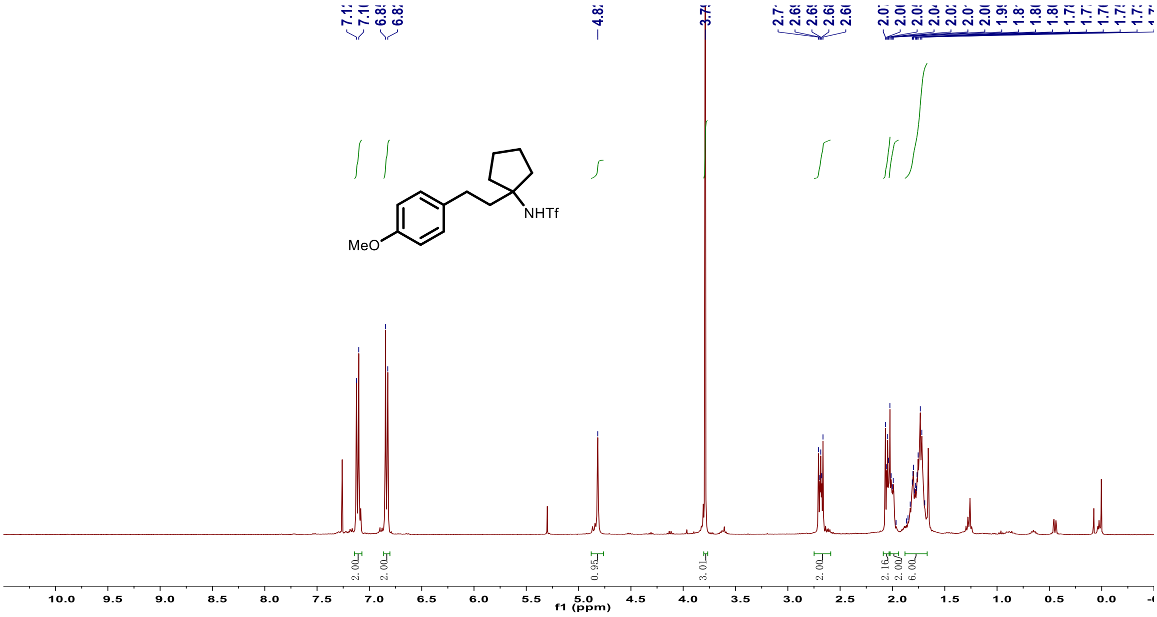

Supplementary Figure 66. ^{1}H NMR (400 MHz, $CDCl_3$) spectrum for 35

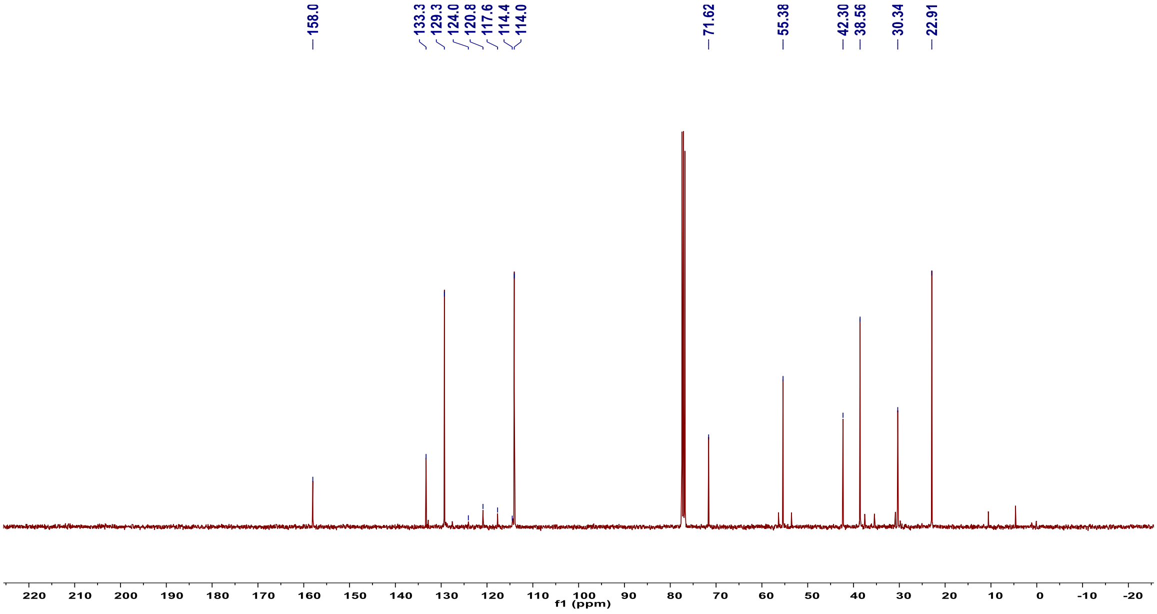

Supplementary Figure 67. ^{13}C NMR (100 MHz, $CDCl_3$) spectrum for 35

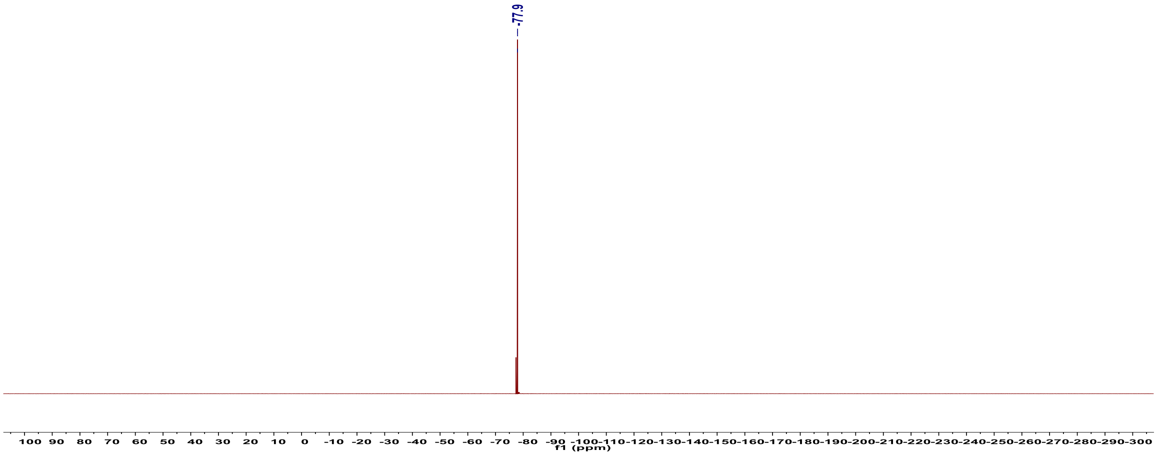

Supplementary Figure 68. ^{19}F NMR (377 MHz, $CDCl_3$) spectrum for 35

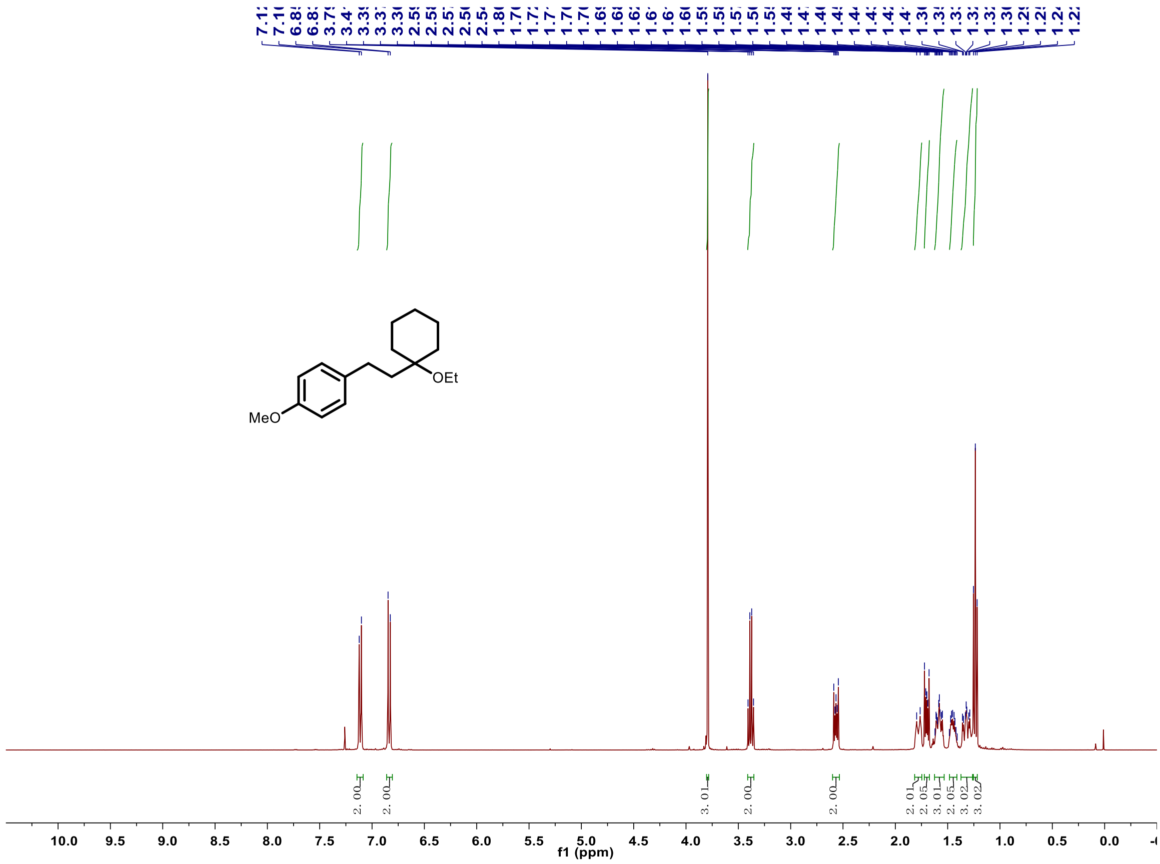

Supplementary Figure 69. ^{1}H NMR (400 MHz, $CDCl_3$) spectrum for 12

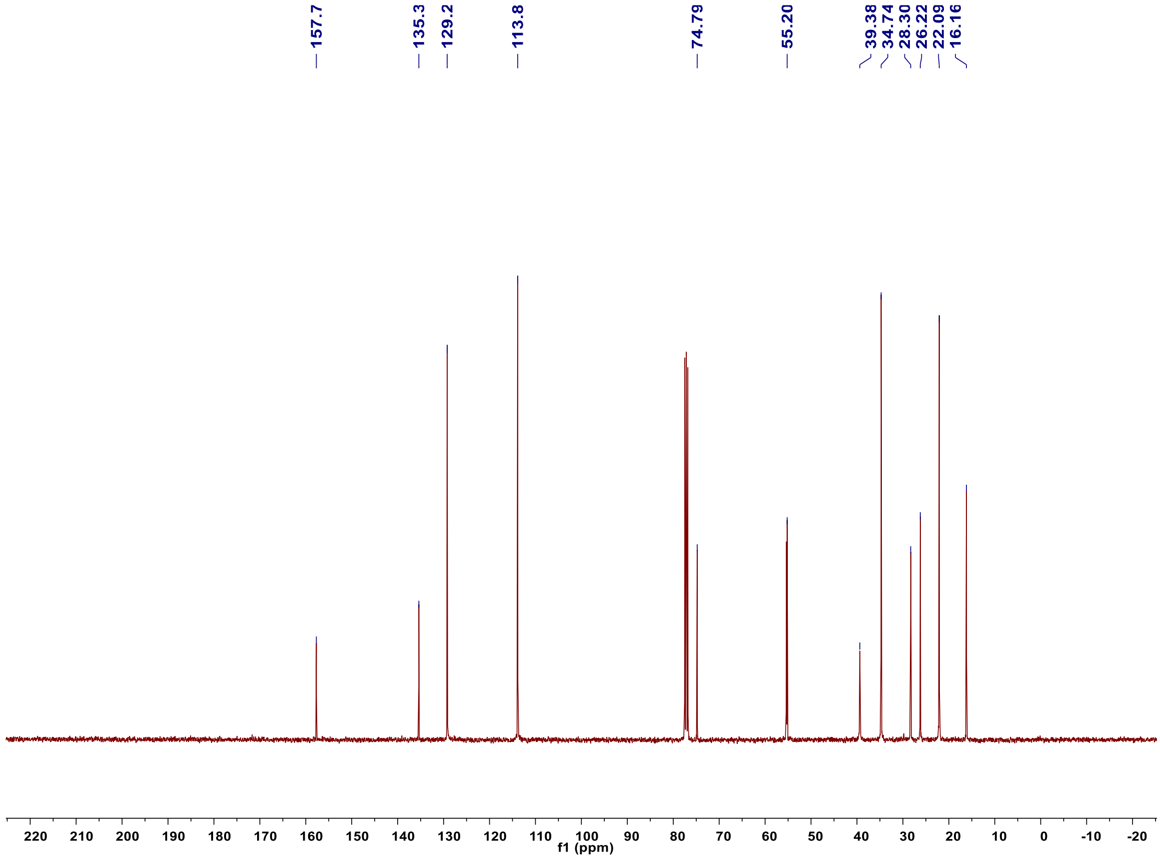

Supplementary Figure 70. ^{13}C NMR (100 MHz, $CDCl_3$) spectrum for 12

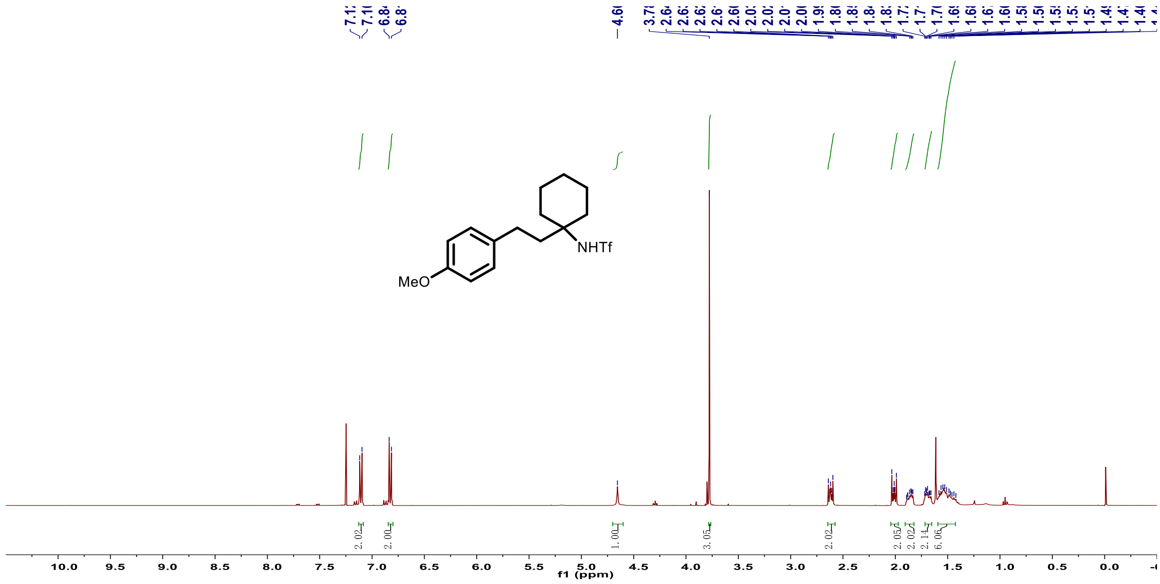

Supplementary Figure 71. ^{1}H **NMR (400 MHz, $CDCl_3$) spectrum for 36**

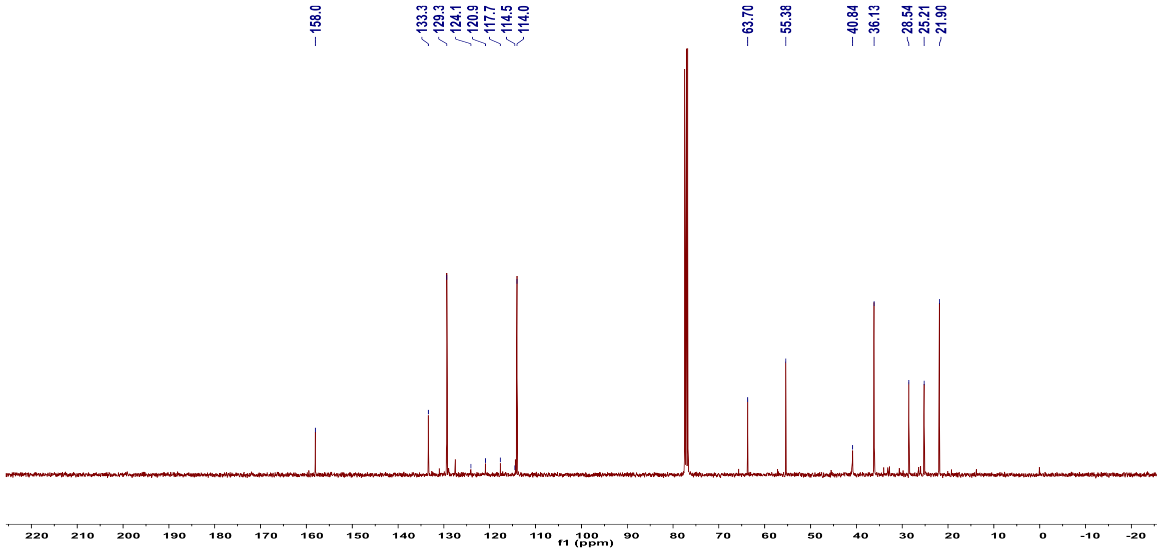

Supplementary Figure 72. ^{13}C **NMR (100 MHz, $CDCl_3$) spectrum for 36**

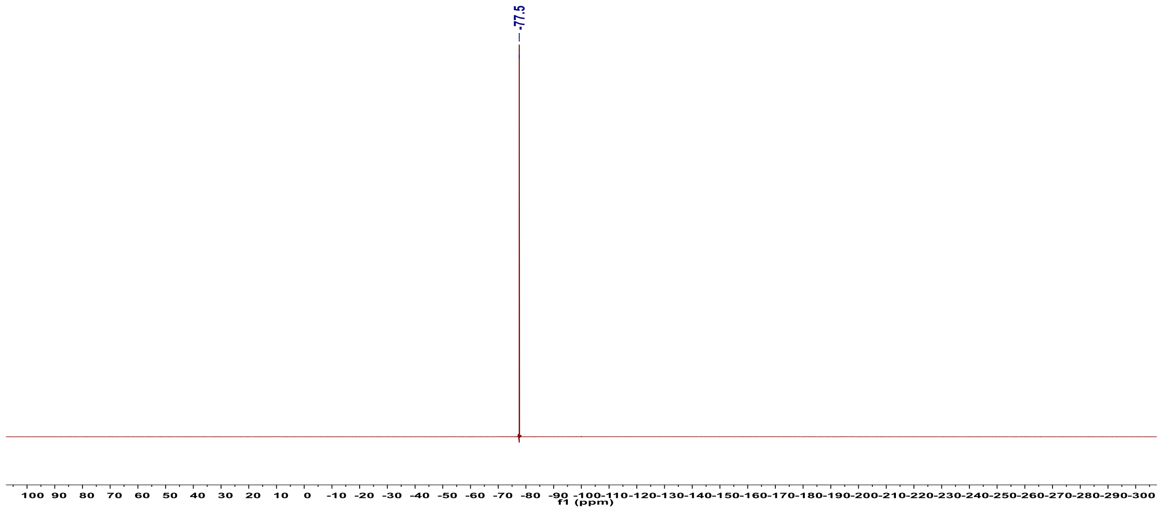

Supplementary Figure 73. ^{19}F **NMR (377 MHz, $CDCl_3$) spectrum for 36**

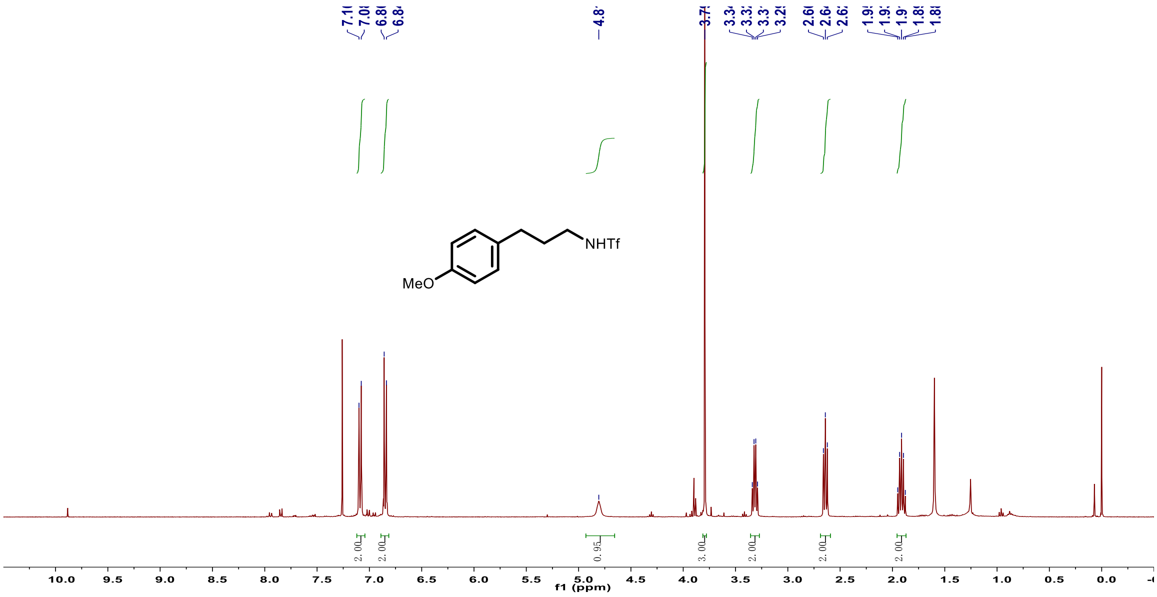

Supplementary Figure 74. ^{1}H **NMR (400 MHz,** $CDCl_3$**) spectrum for 37**

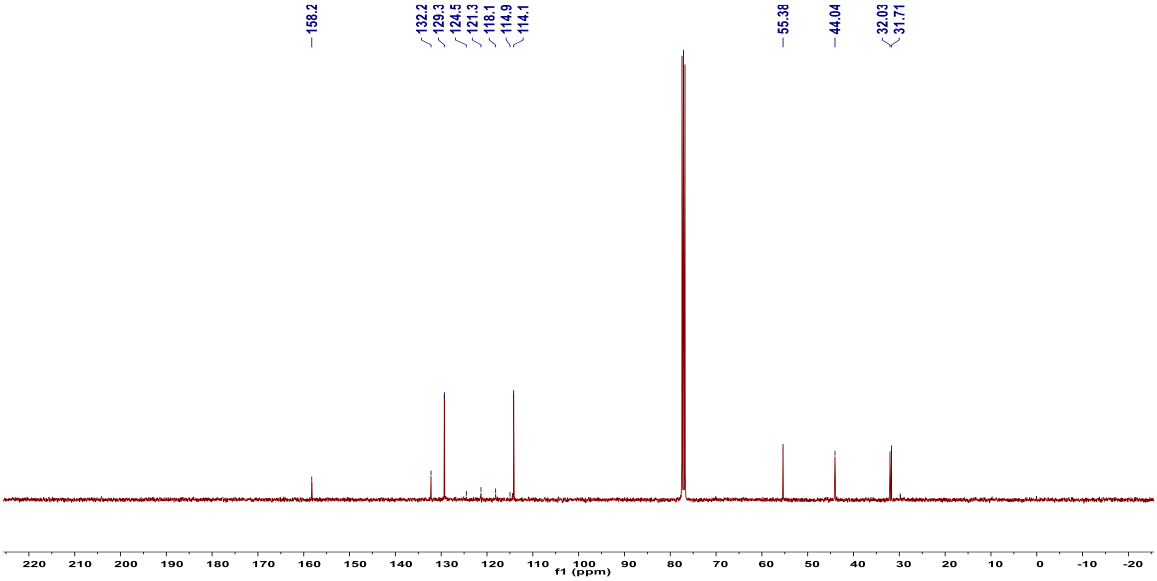

Supplementary Figure 75. ^{13}C **NMR (100 MHz,** $CDCl_3$**) spectrum for 37**

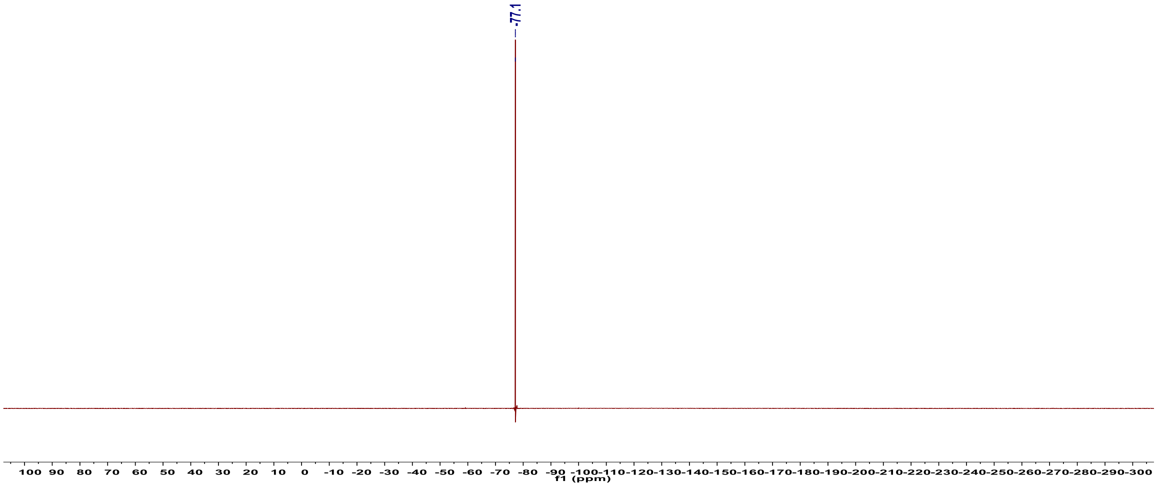

Supplementary Figure 76. ^{19}F **NMR (377 MHz,** $CDCl_3$**) spectrum for 37**

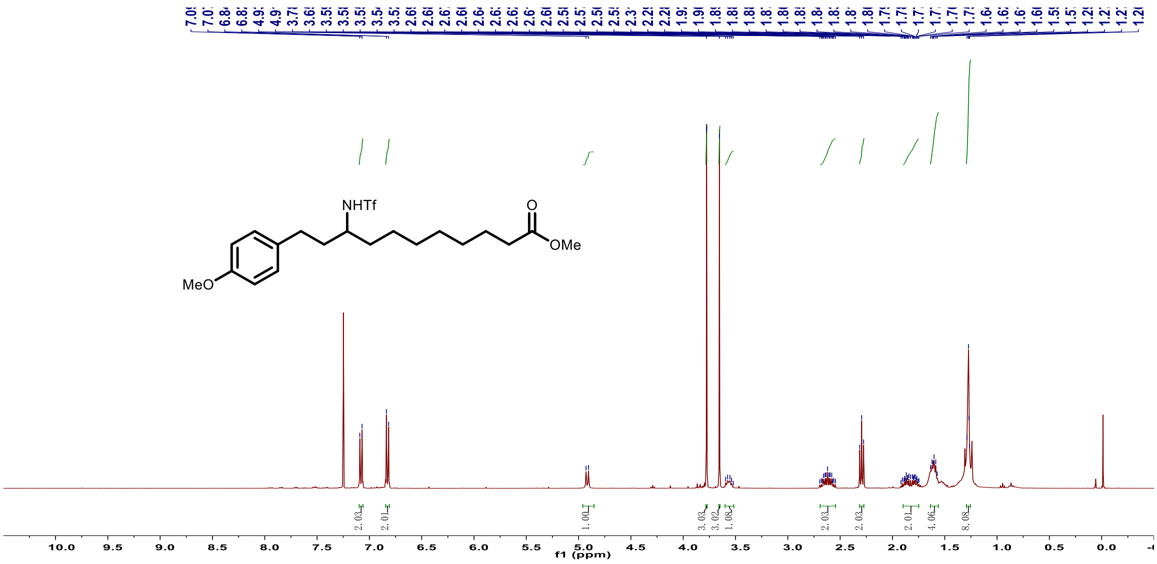

Supplementary Figure 77. ^{1}H NMR (400 MHz, CDCl$_3$) spectrum for 38

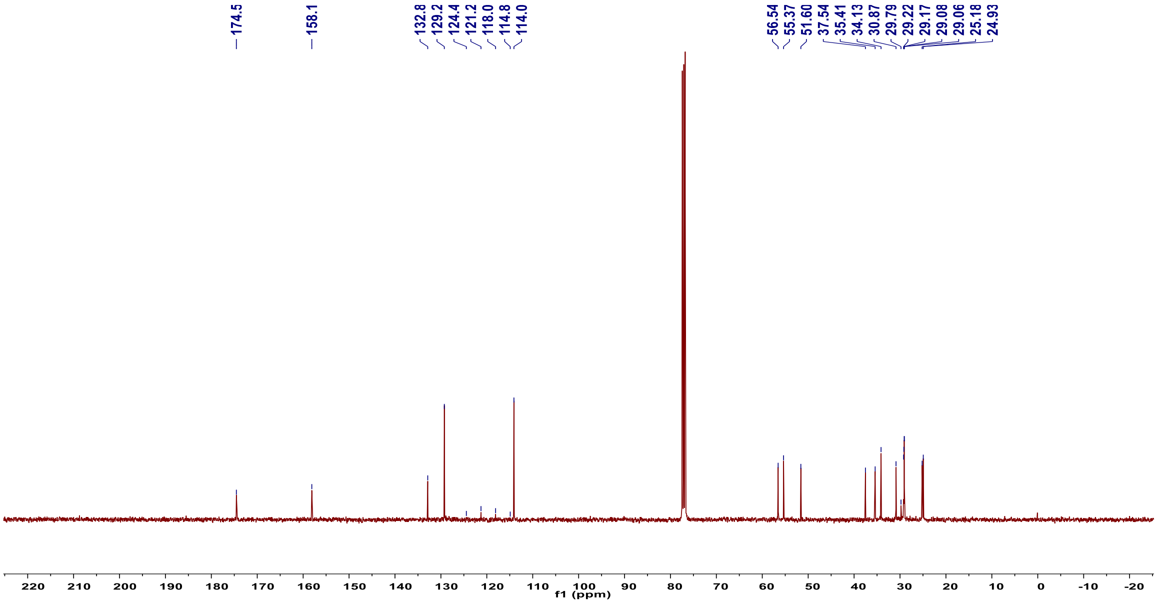

Supplementary Figure 78. ^{13}C NMR (100 MHz, CDCl$_3$) spectrum for 38

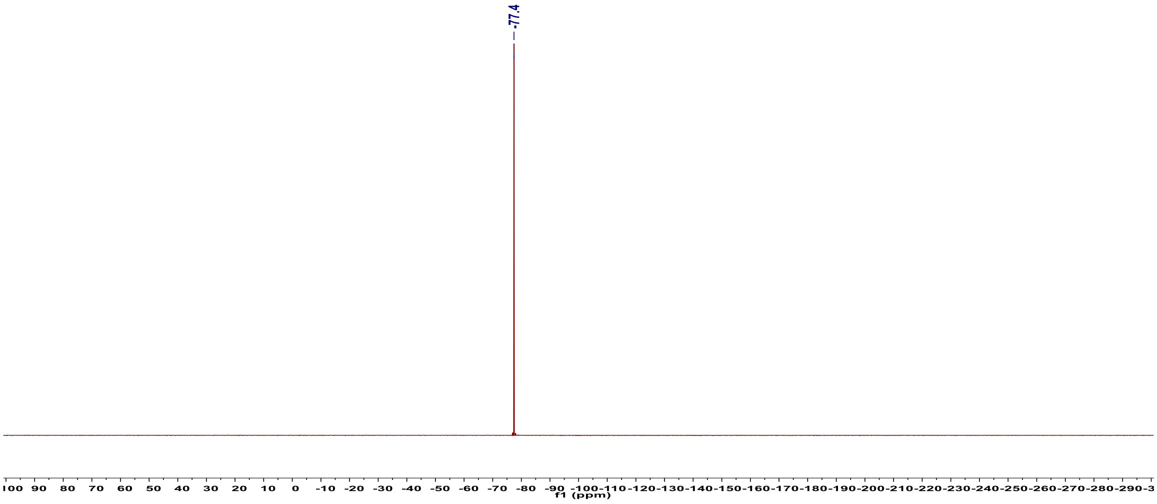

Supplementary Figure 79. ^{19}F NMR (377 MHz, CDCl$_3$) spectrum for 38

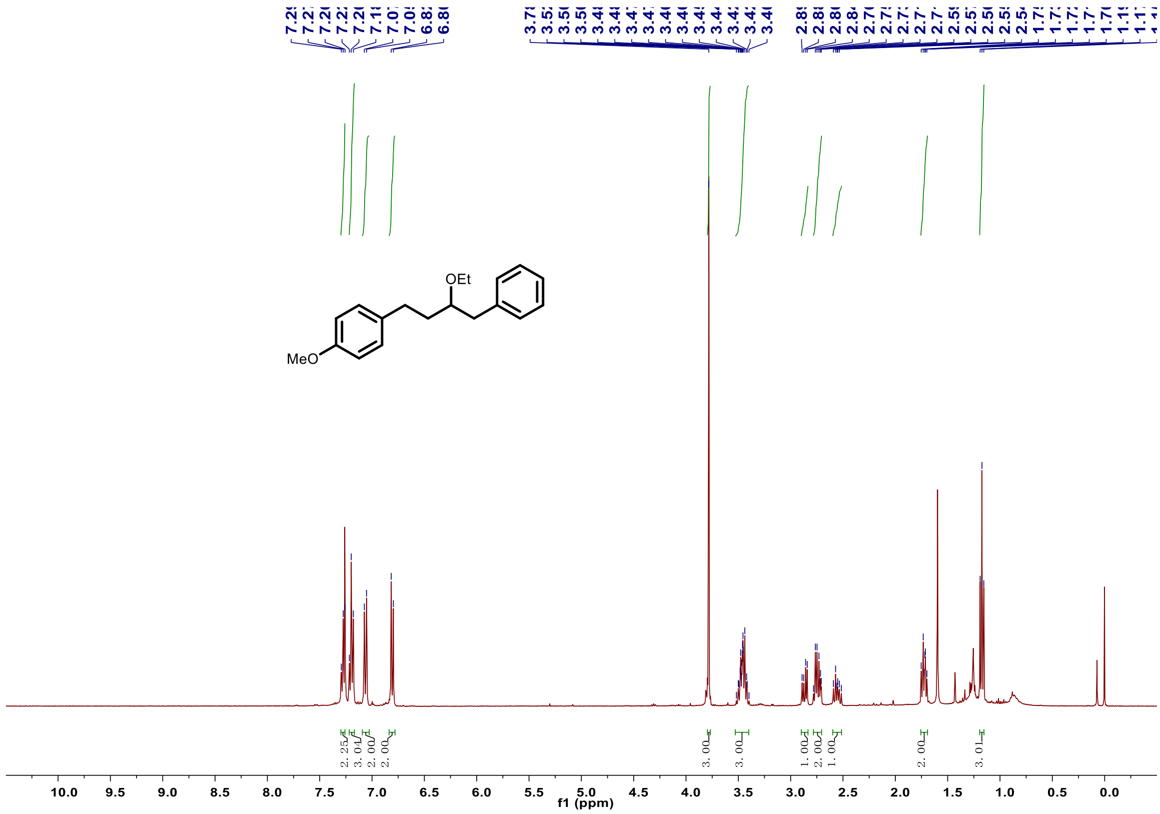

Supplementary Figure 80. ^{1}H **NMR (400 MHz,** $CDCl_3$**) spectrum for 13**

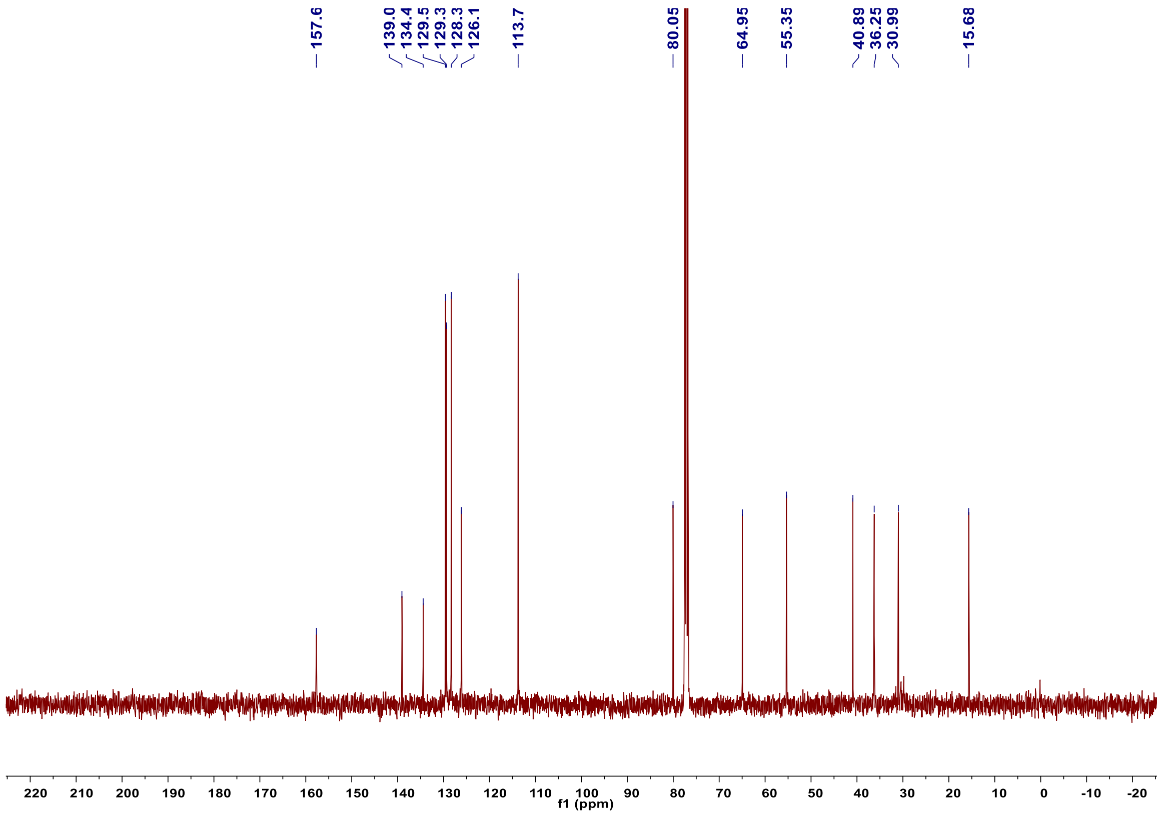

Supplementary Figure 81. ^{13}C **NMR (100 MHz,** $CDCl_3$**) spectrum for 13**

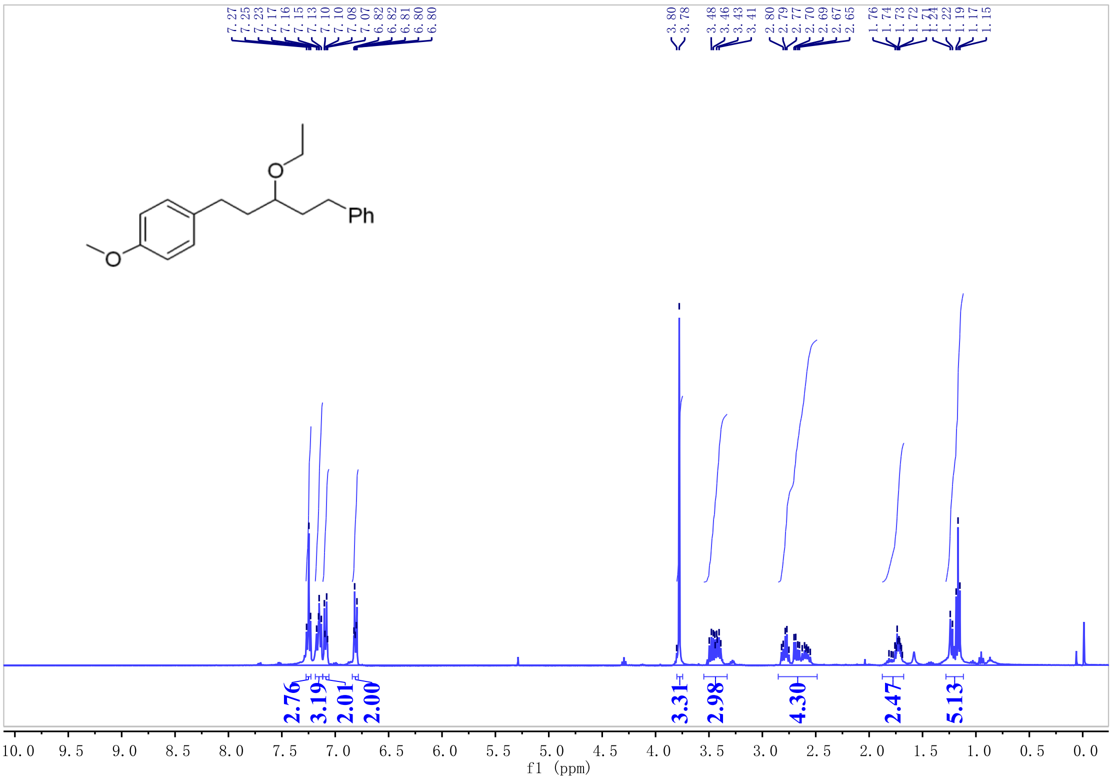

Supplementary Figure 82. ^{1}H NMR (400 MHz, $CDCl_3$) spectrum for 14

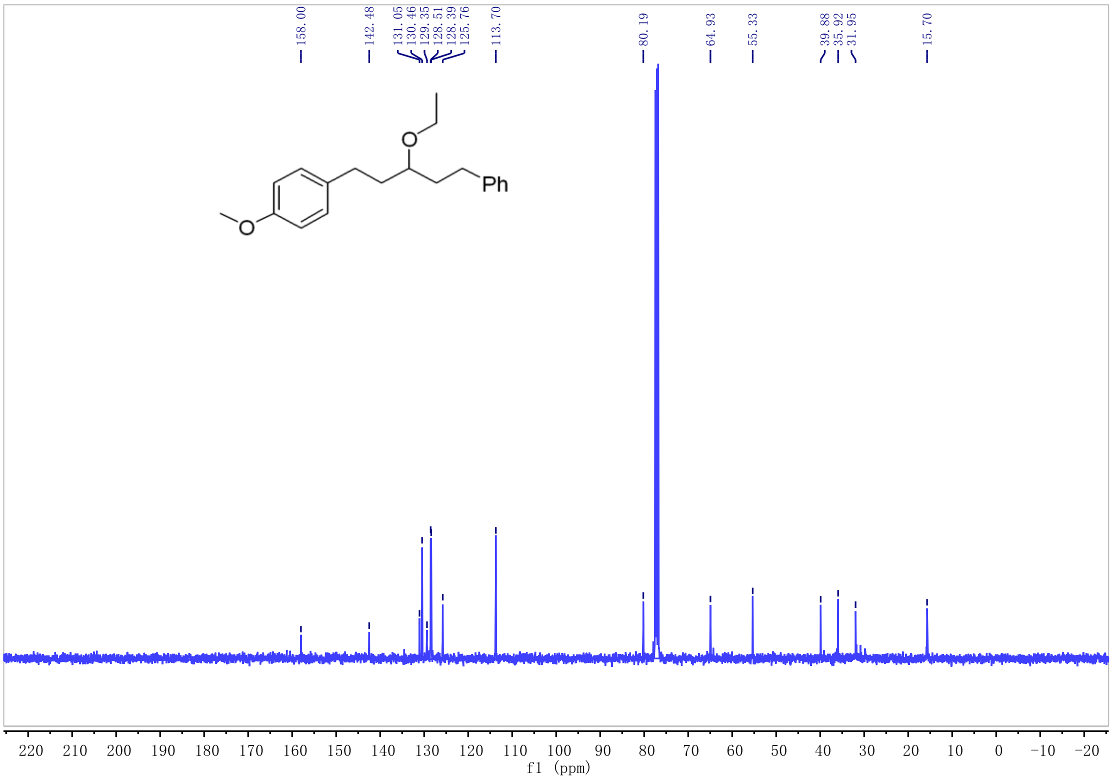

Supplementary Figure 83. ^{13}C NMR (100 MHz, $CDCl_3$) spectrum for 14

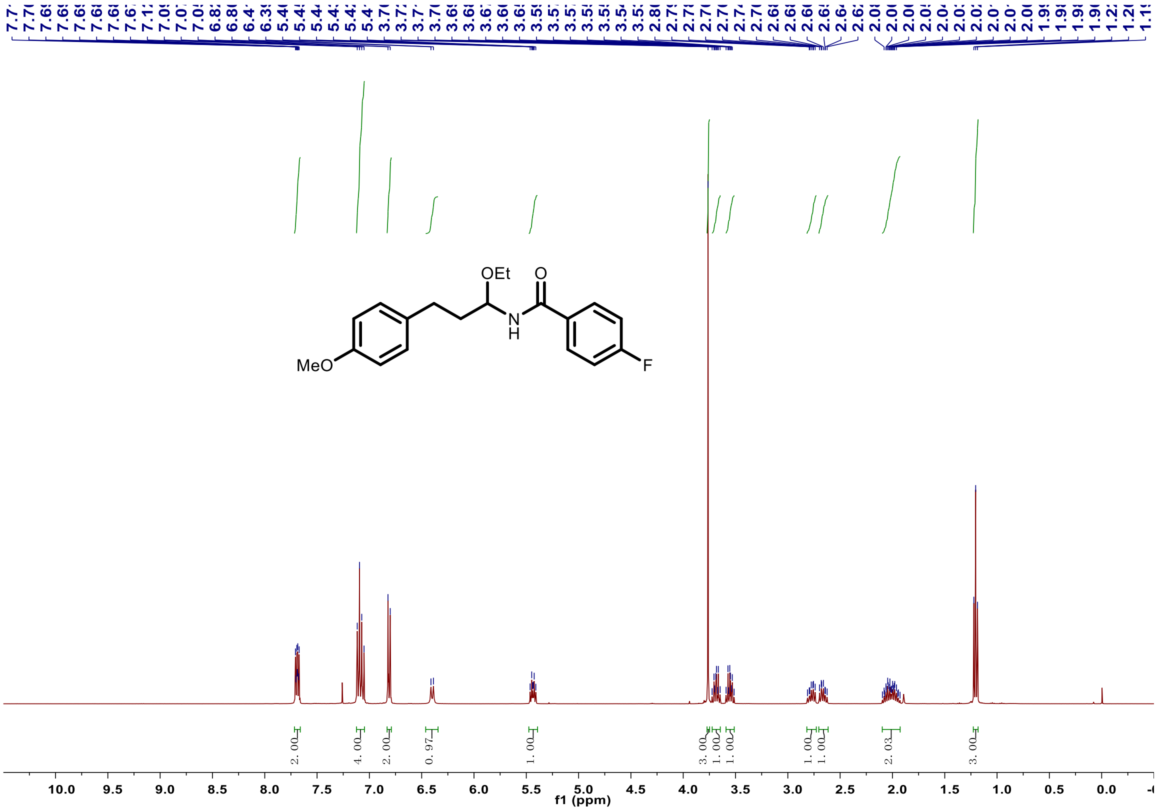

Supplementary Figure 84. ^{1}H NMR (400 MHz, $CDCl_3$) spectrum for 15

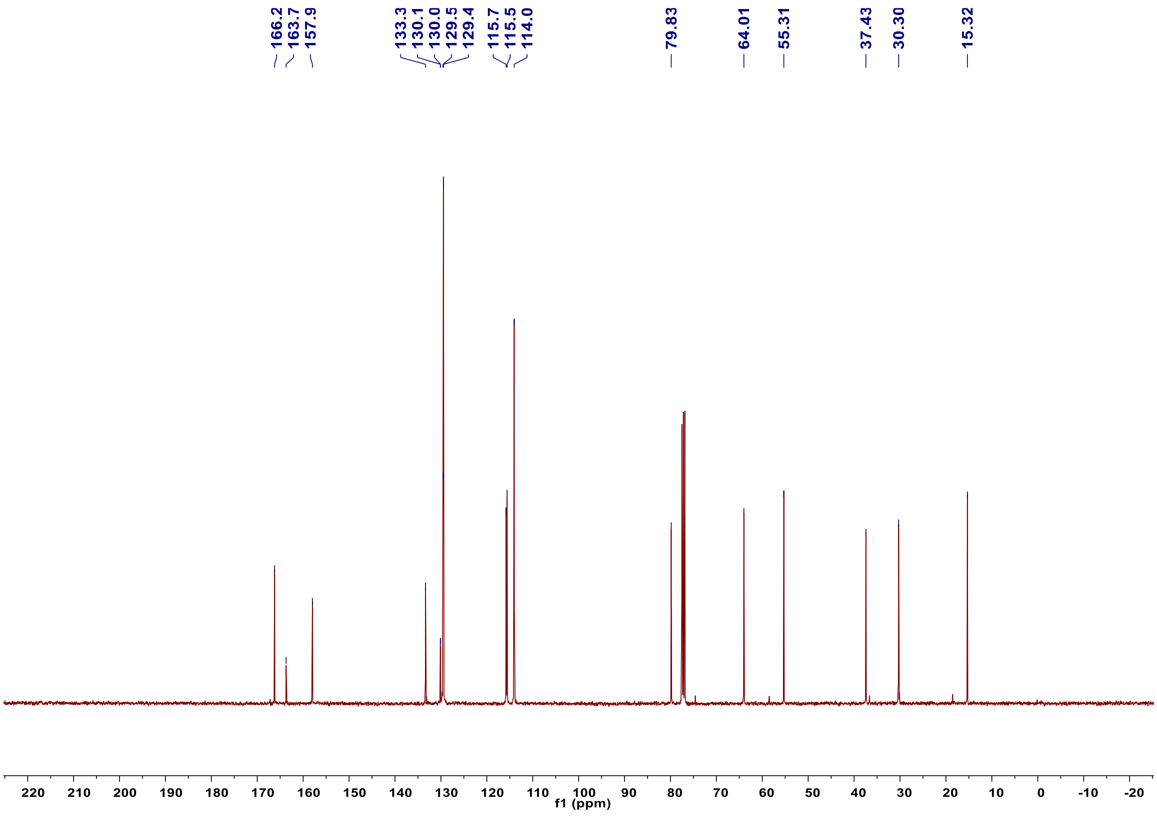

Supplementary Figure 85. ^{13}C NMR (100 MHz, $CDCl_3$) spectrum for 15

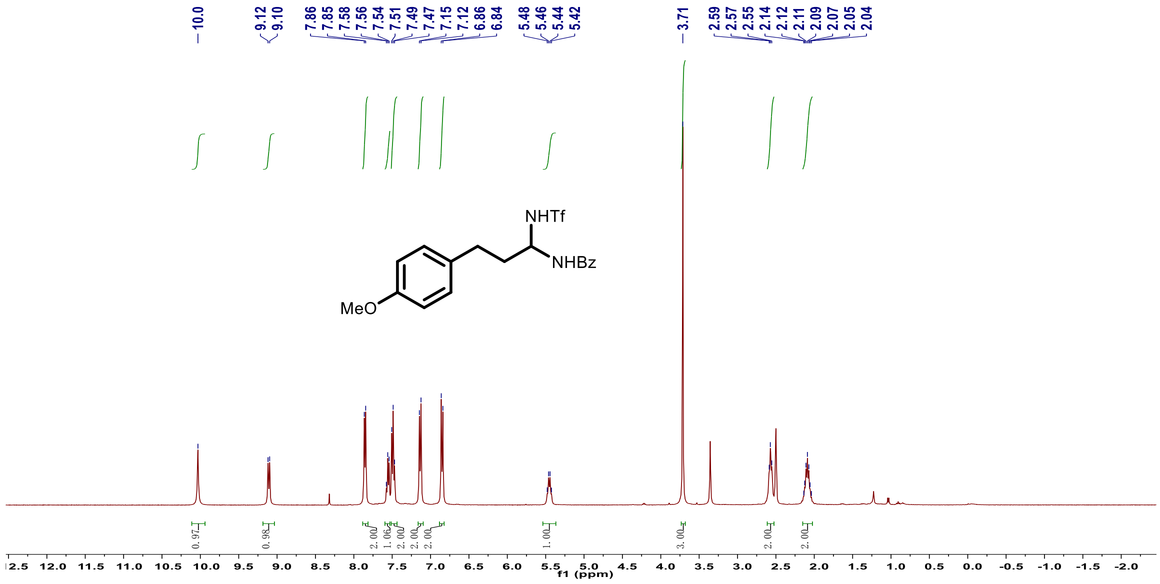

Supplementary Figure 86. ^{1}H NMR (400 MHz, DMSO-*d6*) spectrum for 39

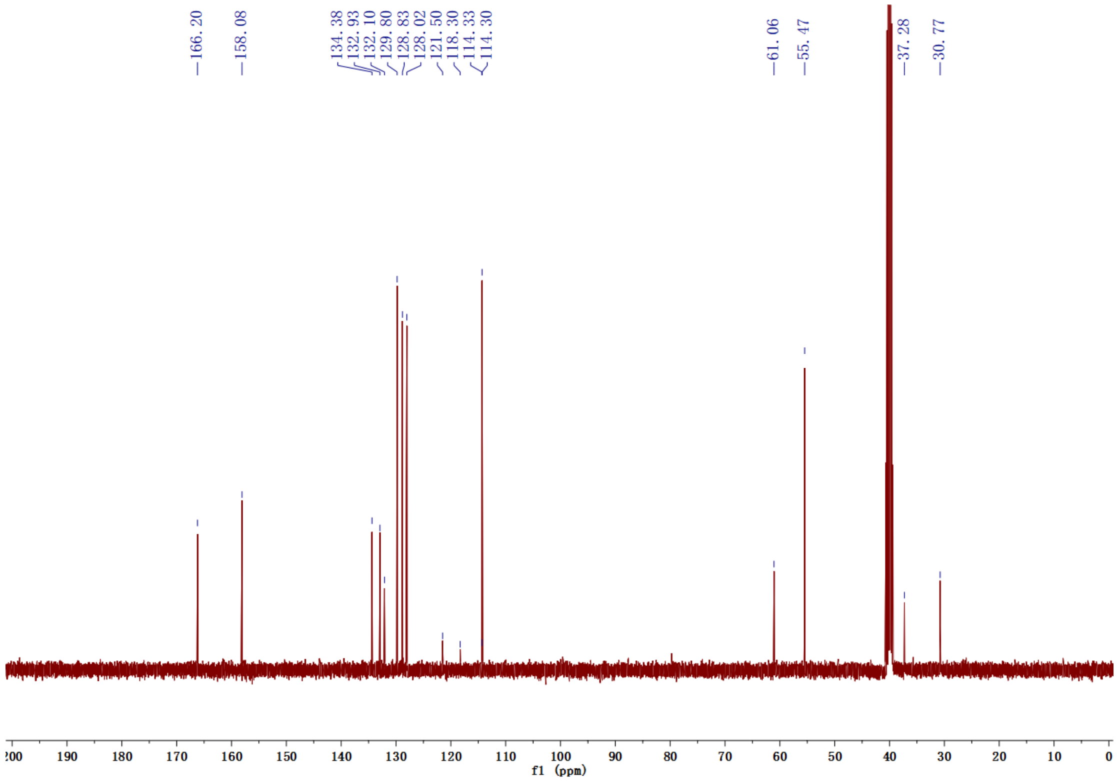

Supplementary Figure 87. ^{13}C NMR (100 MHz, DMSO-*d6*) spectrum for 39

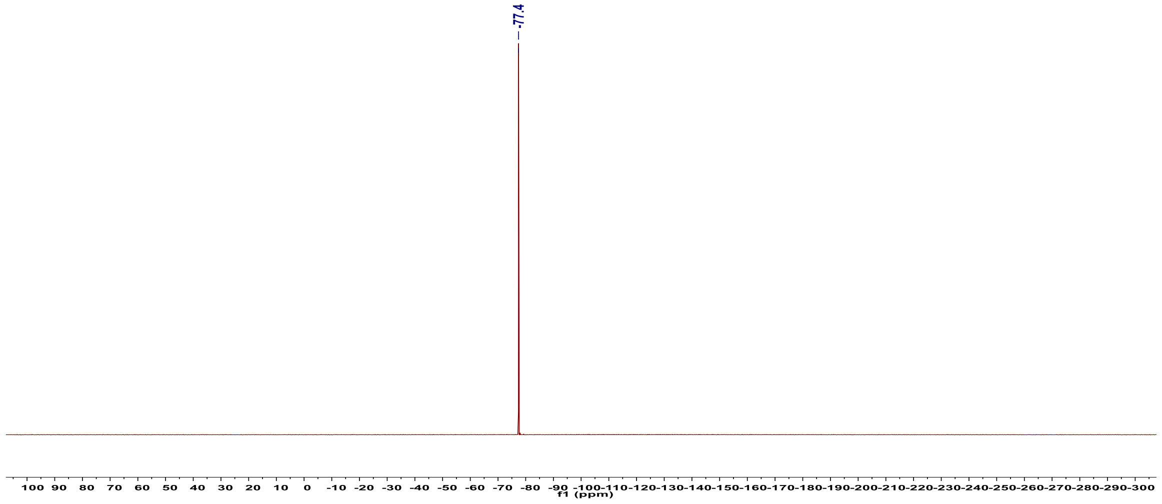

Supplementary Figure 88. ^{19}F NMR (377 MHz, DMSO-*d6*) spectrum for 39

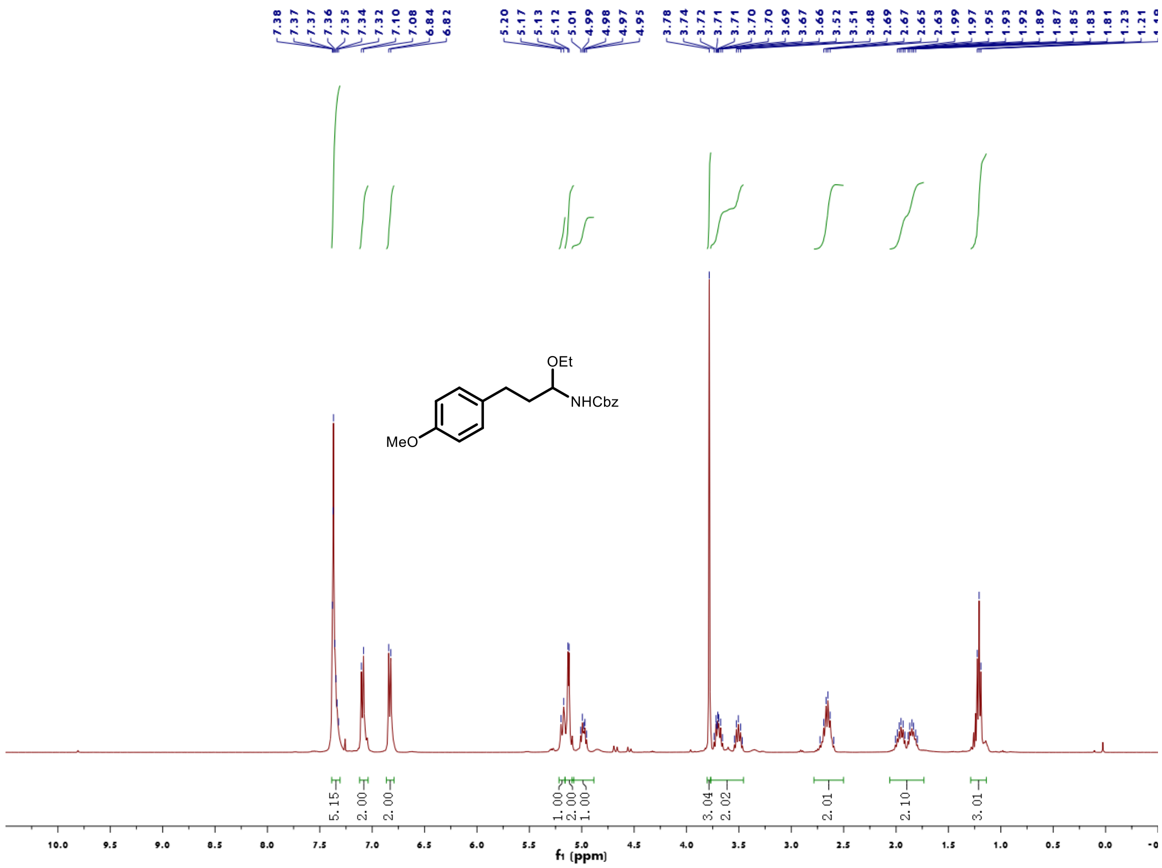

Supplementary Figure 89. ^{1}H NMR (400 MHz, $CDCl_3$) spectrum for 16

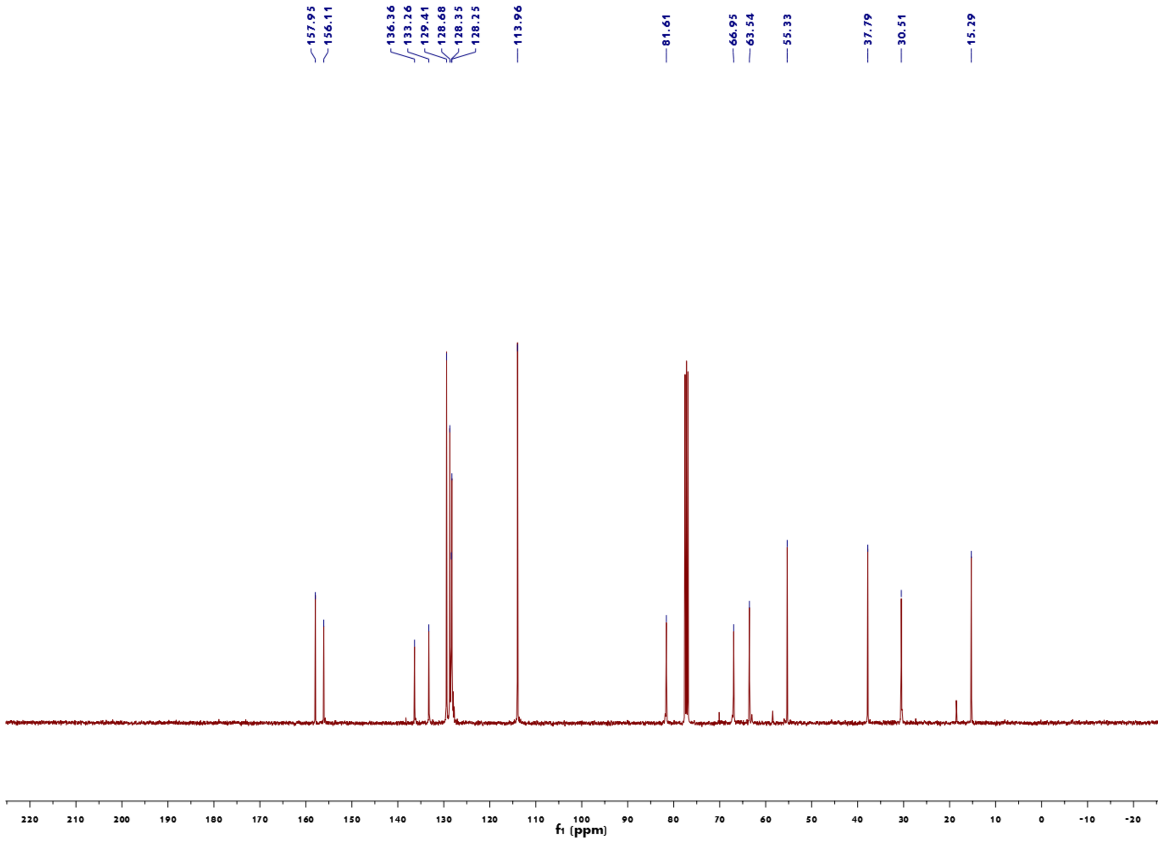

Supplementary Figure 90. ^{13}C NMR (100 MHz, $CDCl_3$) spectrum for 16

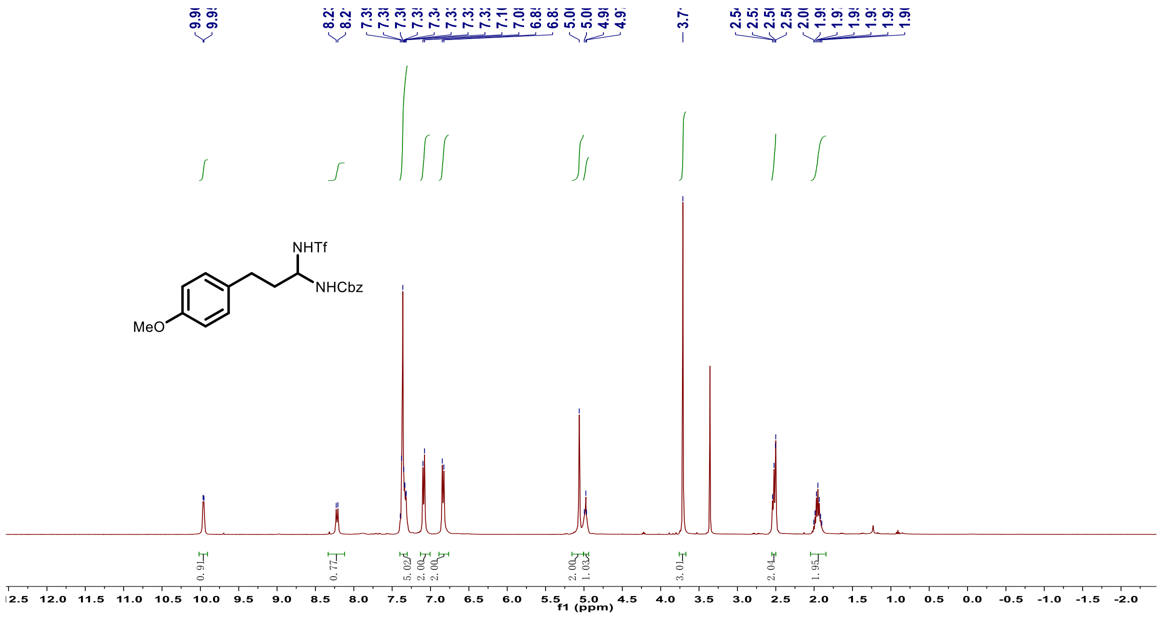

Supplementary Figure 91. ^{1}H NMR (400 MHz, DMSO-*d6*) spectrum for 40

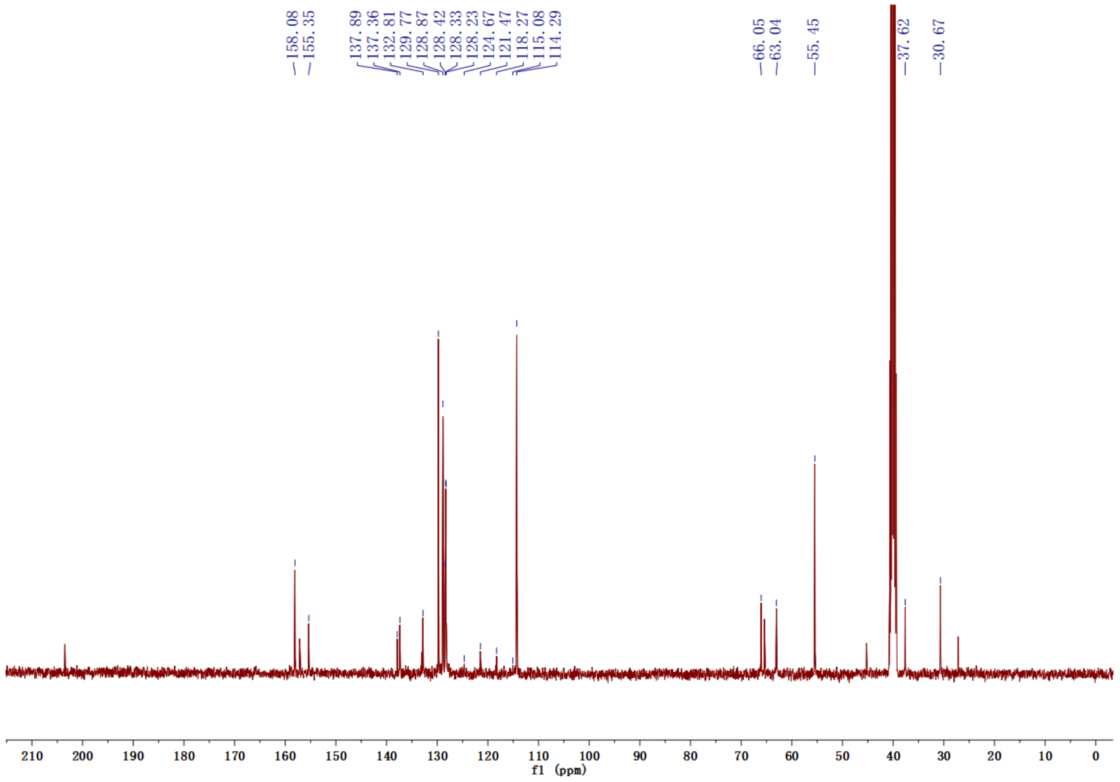

Supplementary Figure 92. ^{13}C **NMR (100 MHz, DMSO-*d6*) spectrum for 40**

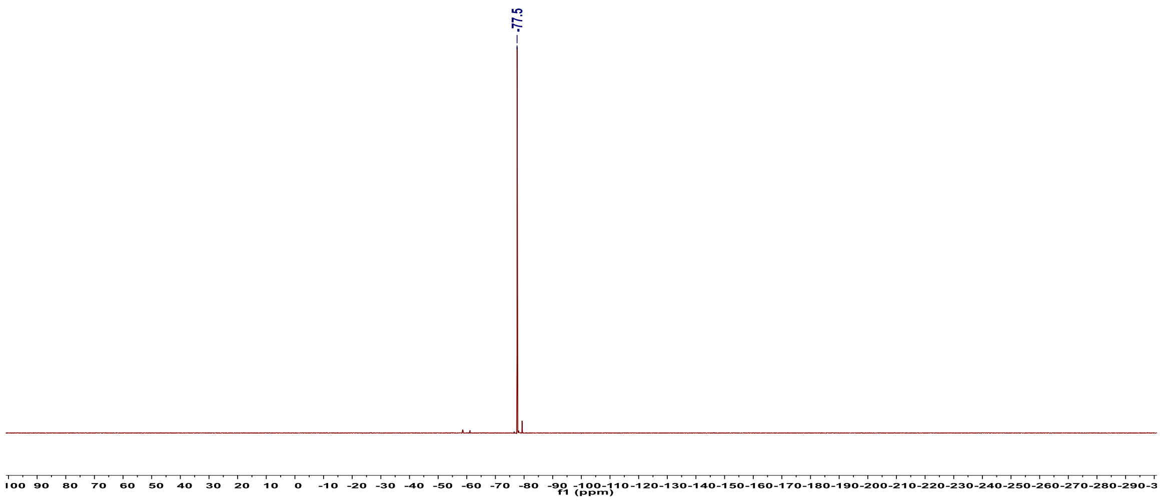

Supplementary Figure 93. ^{19}F **NMR (377 MHz, DMSO-*d6*) spectrum for 40**

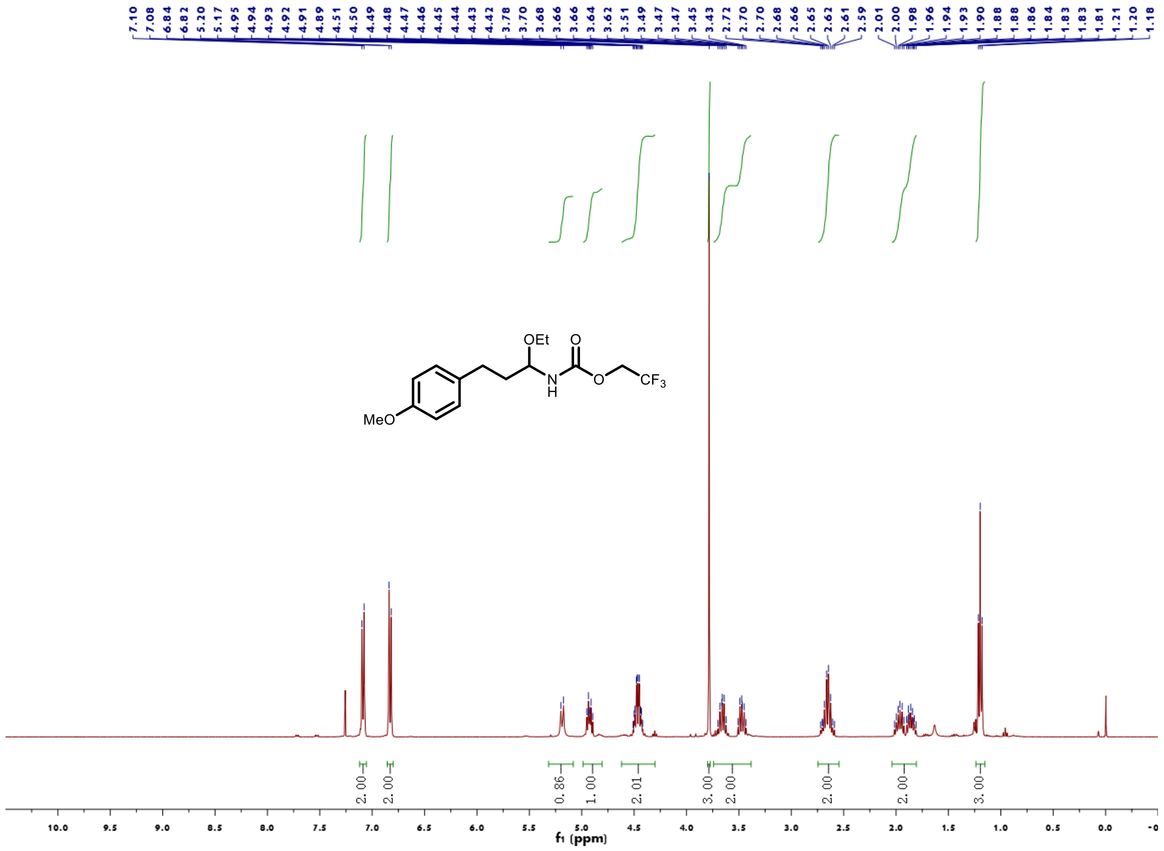

Supplementary Figure 94. ^{1}H NMR (400 MHz, $CDCl_3$) spectrum for 17

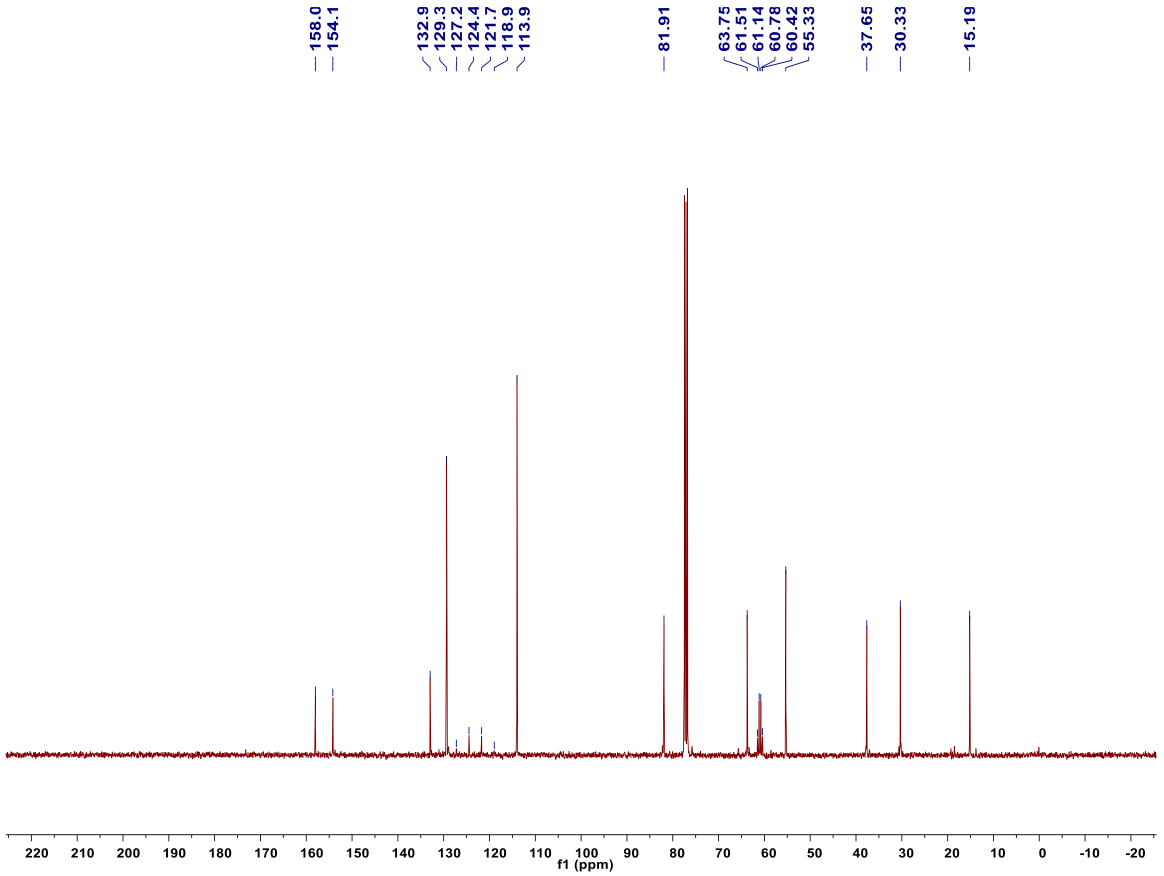

Supplementary Figure 95. ^{13}C NMR (100 MHz, $CDCl_3$) spectrum for 17

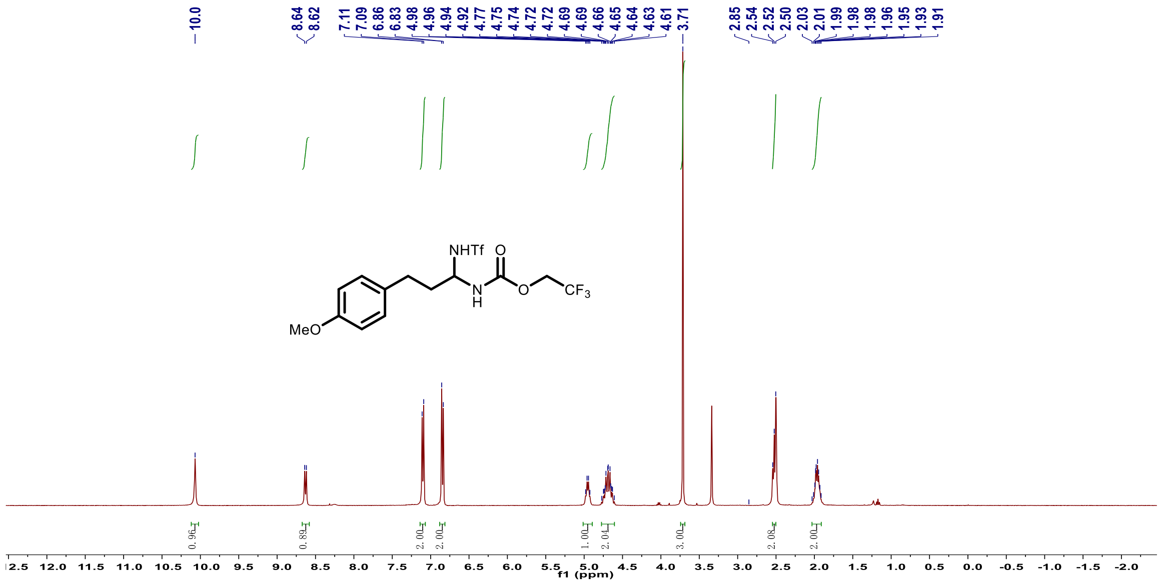

Supplementary Figure 96. ^{1}H NMR (400 MHz, DMSO-*d*6) spectrum for 41

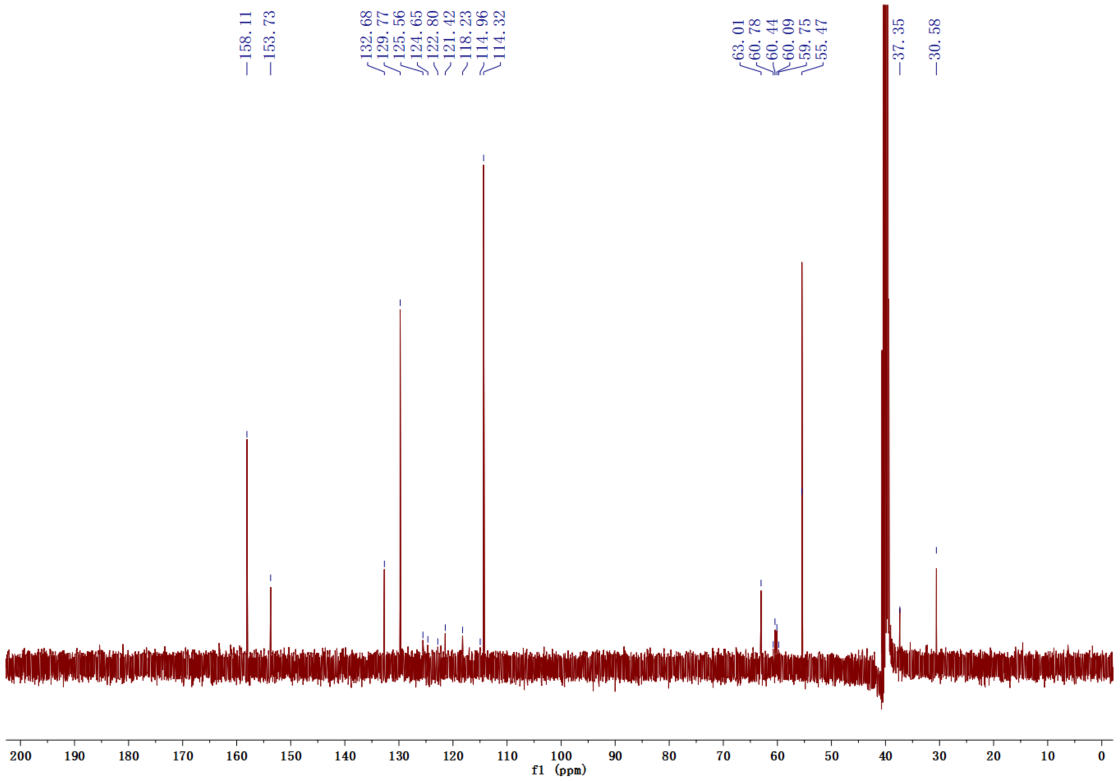

Supplementary Figure 97. ^{13}C NMR (100 MHz, DMSO-*d*6) spectrum for 41

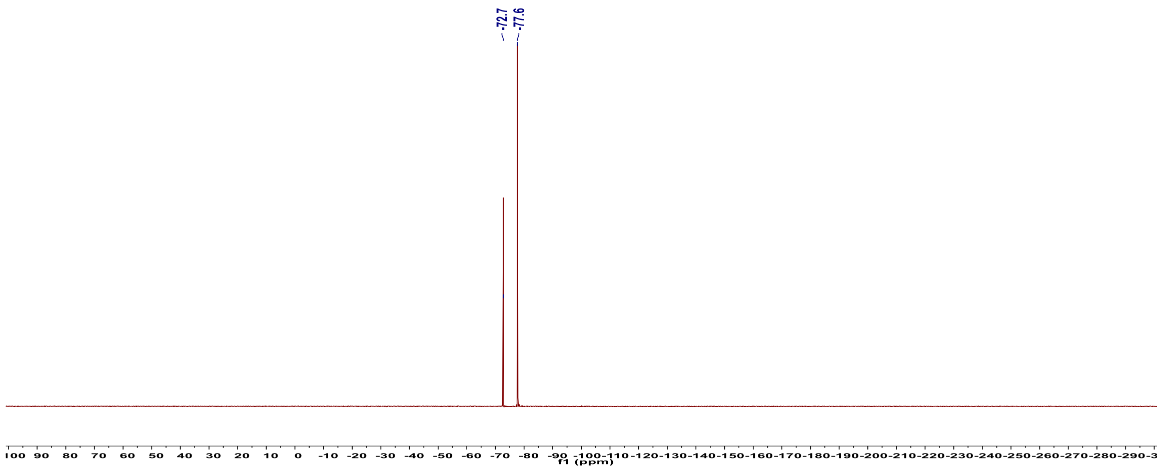

Supplementary Figure 98. ^{19}F NMR (377 MHz, DMSO-*d6*) spectrum for 41

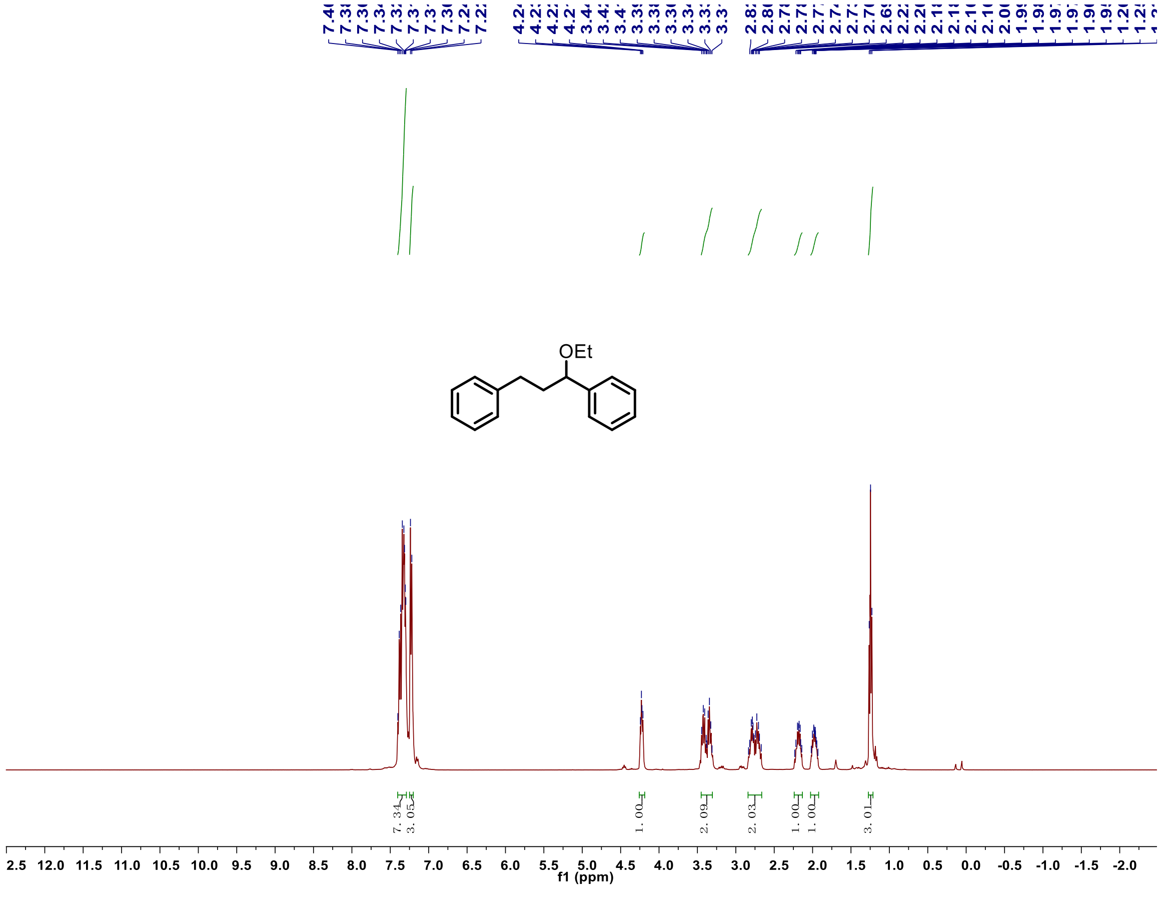

Supplementary Figure 99. ^{1}H NMR (400 MHz, $CDCl_3$) spectrum for 18

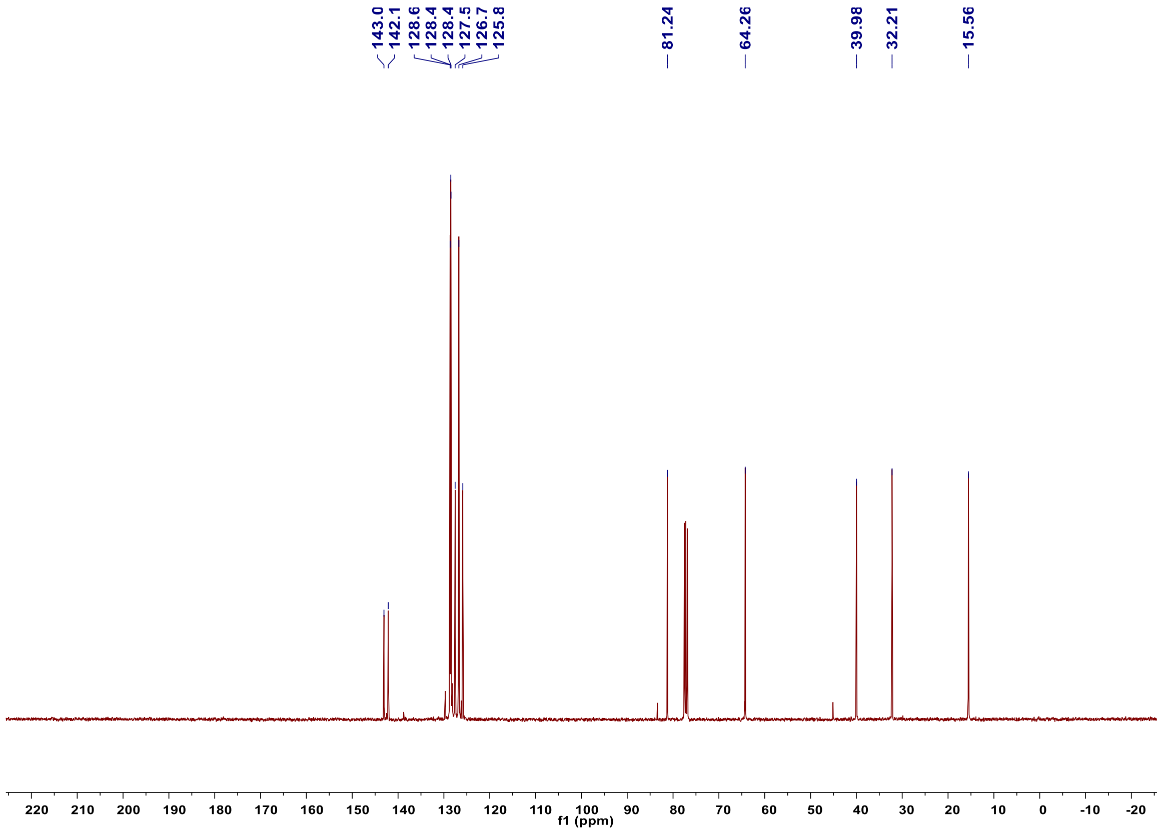

Supplementary Figure 100. ^{13}C NMR (100 MHz, $CDCl_3$) spectrum for 18

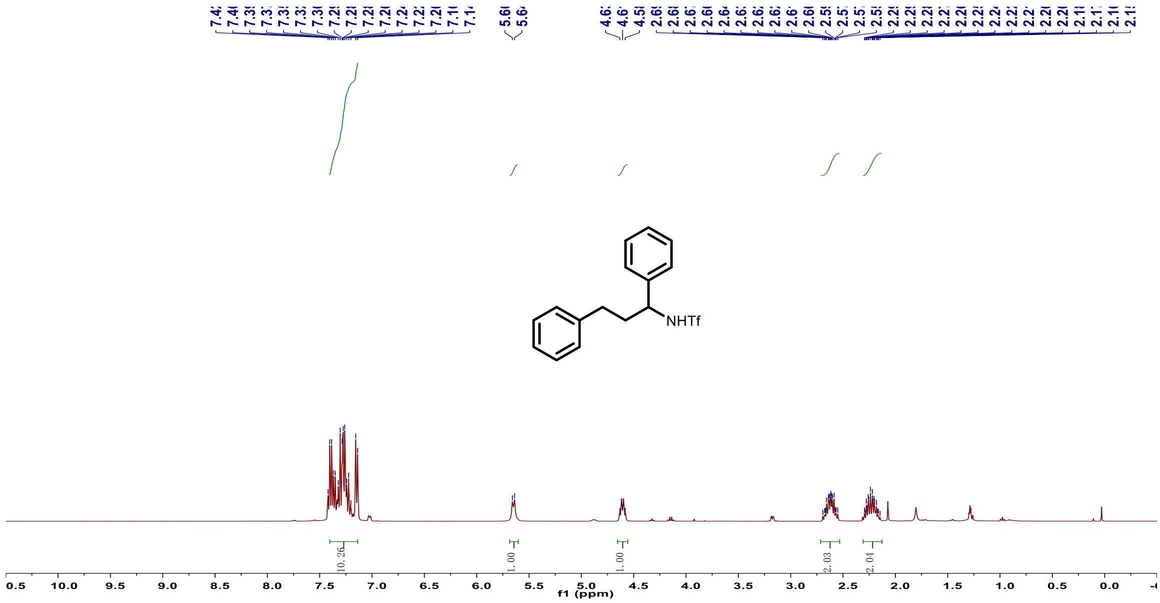

Supplementary Figure 101. ^{1}H NMR (400 MHz, $CDCl_3$) spectrum for 42

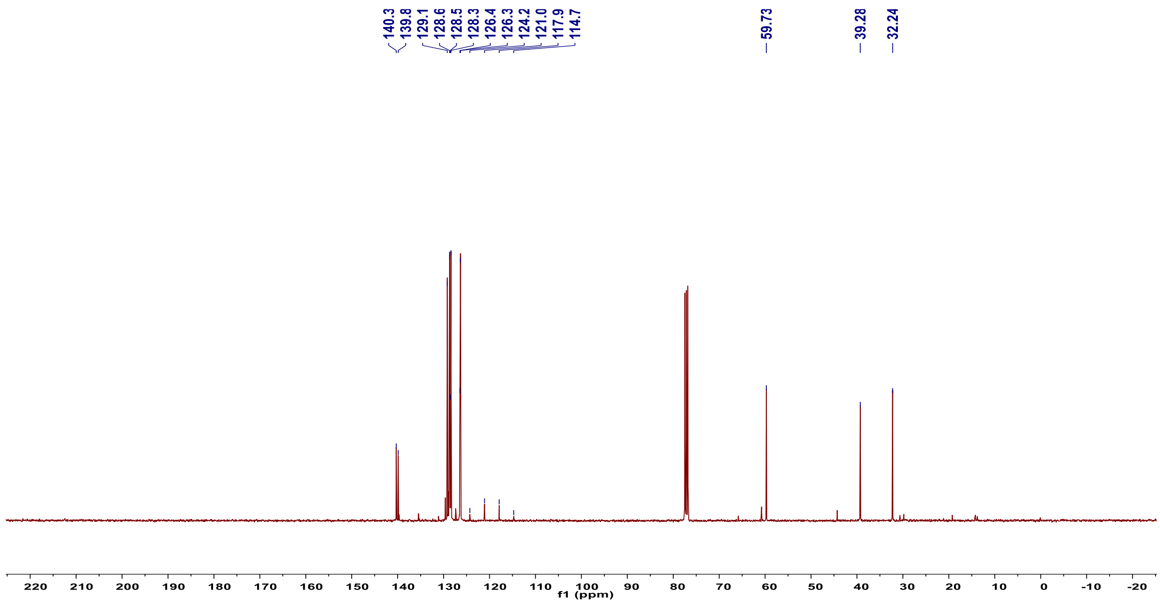

Supplementary Figure 102. ^{13}C NMR (100 MHz, $CDCl_3$) spectrum for 42

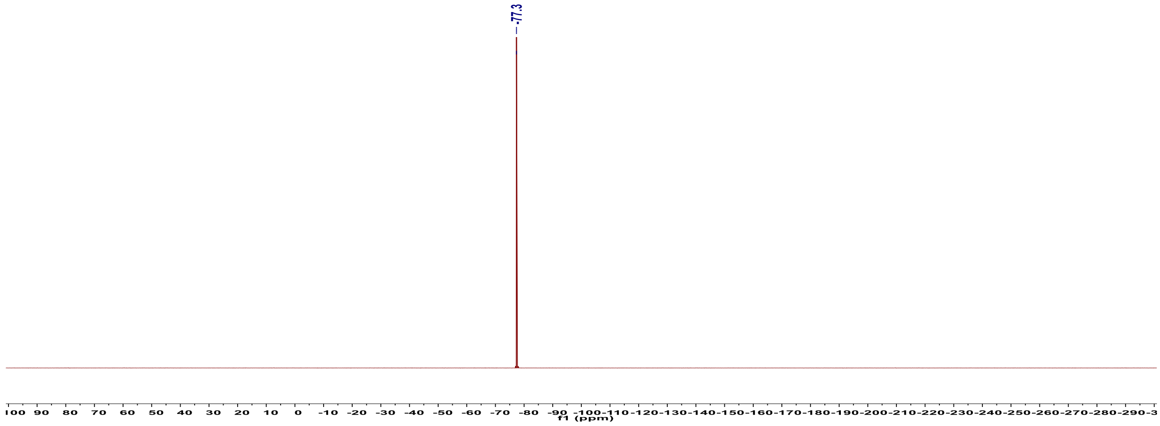

Supplementary Figure 103. ^{19}F NMR (377 MHz, $CDCl_3$) spectrum for 42

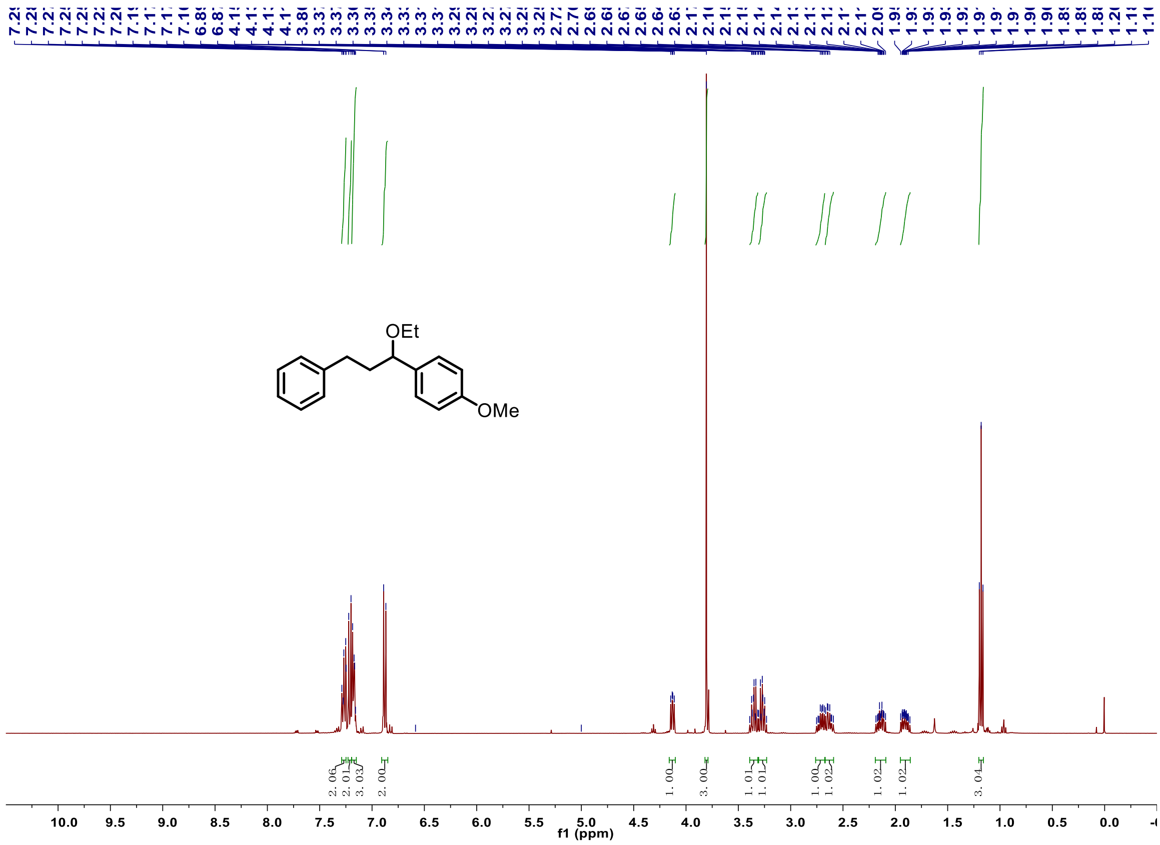

Supplementary Figure 104. ^{1}H NMR (400 MHz, $CDCl_3$) spectrum for 19

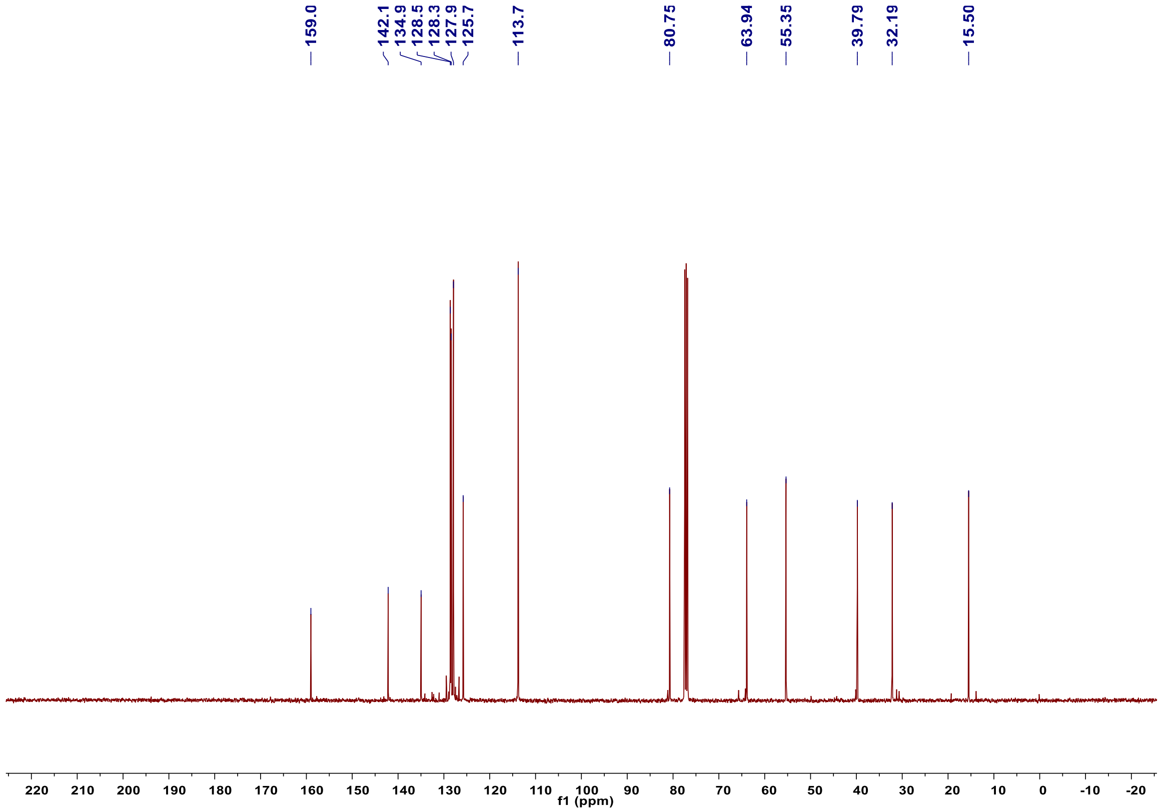

Supplementary Figure 105. ^{13}C NMR (100 MHz, $CDCl_3$) spectrum for 19

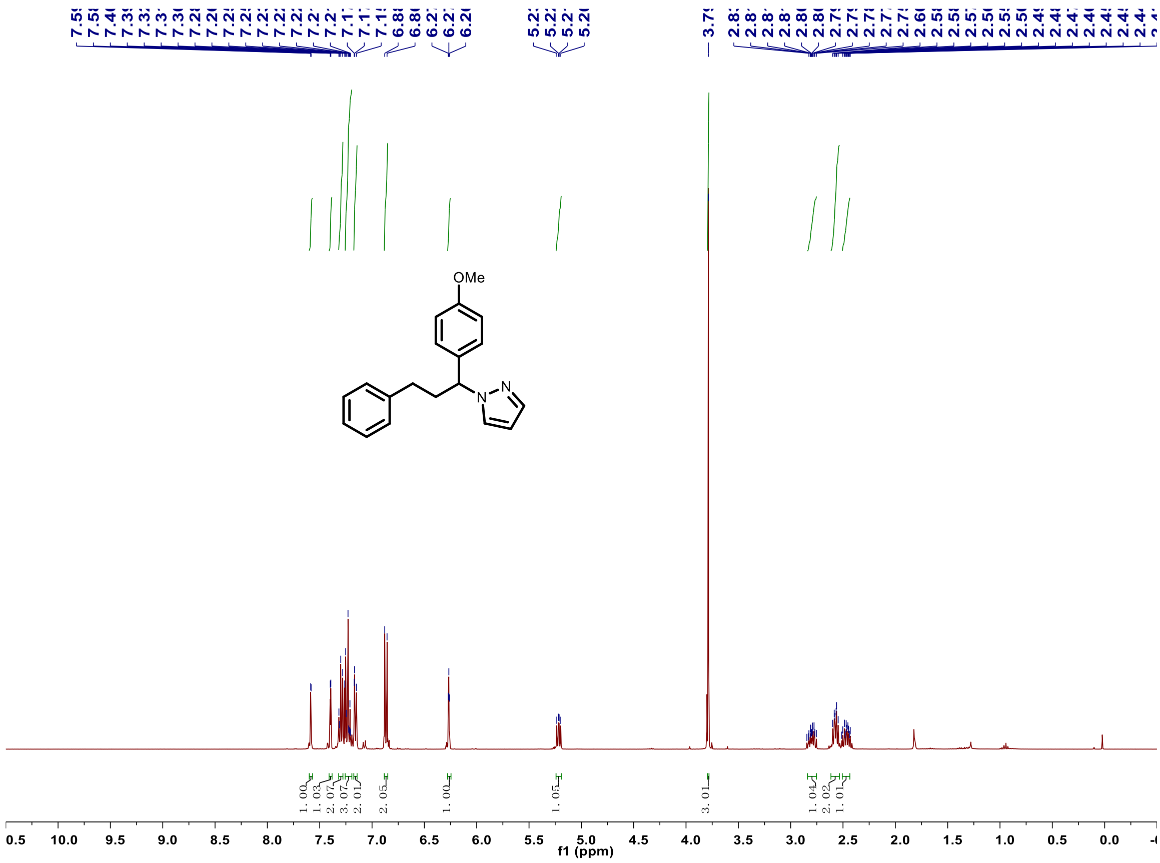

Supplementary Figure 106. ^{1}H NMR (400 MHz, $CDCl_3$) spectrum for 43

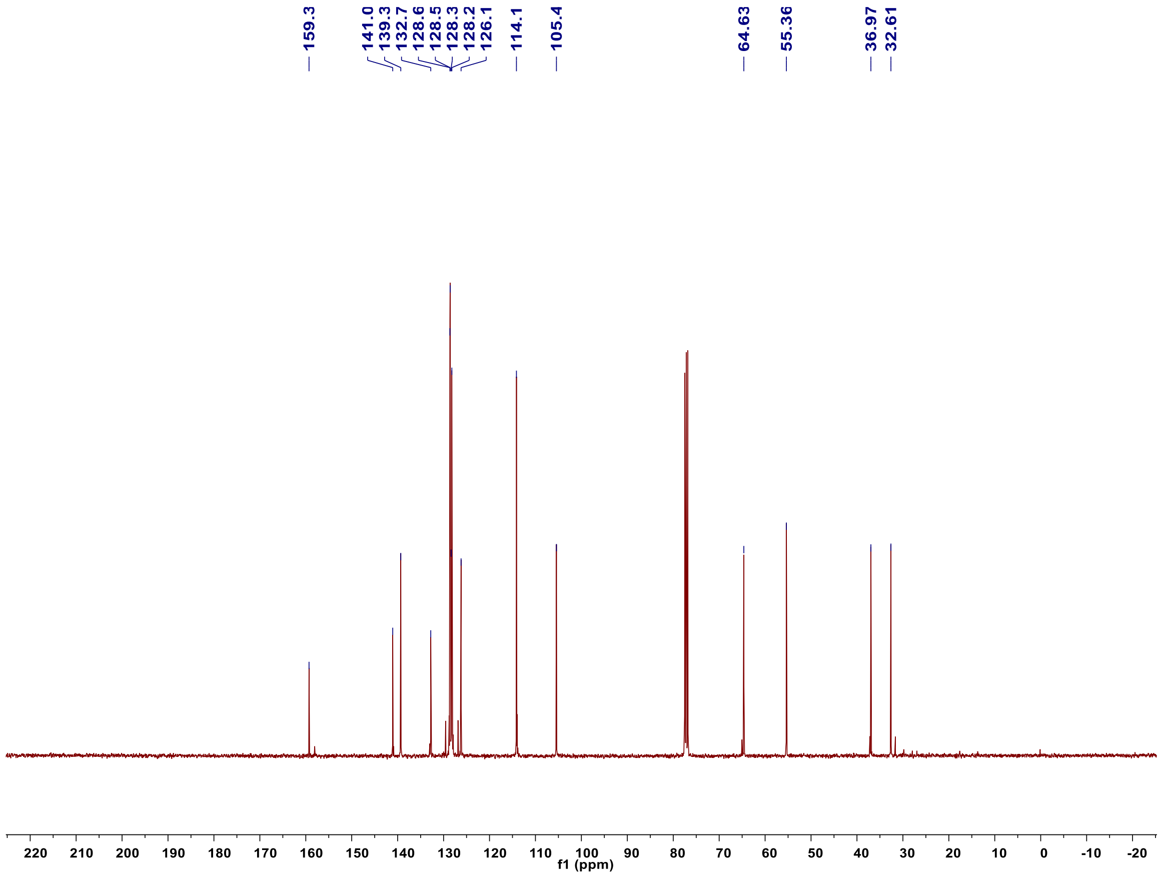

Supplementary Figure 107. ^{13}C NMR (100 MHz, $CDCl_3$) spectrum for 43

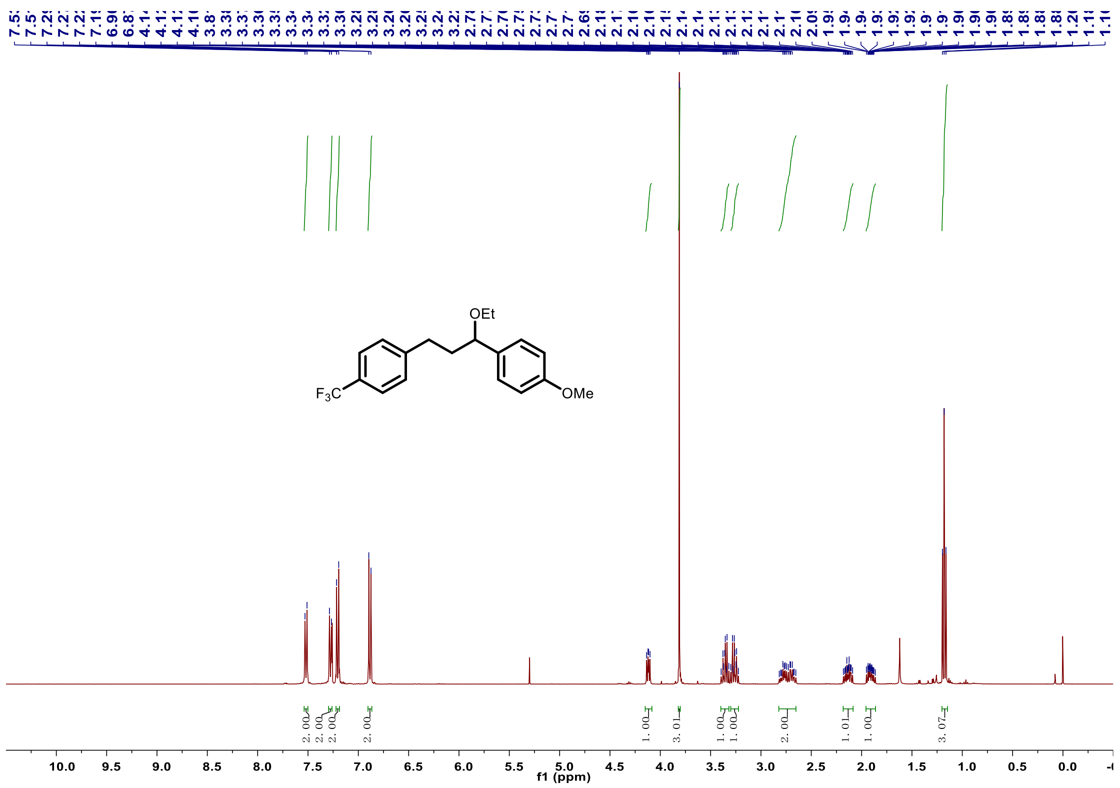

Supplementary Figure 108. ^{1}H NMR (400 MHz, $CDCl_3$) spectrum for 20

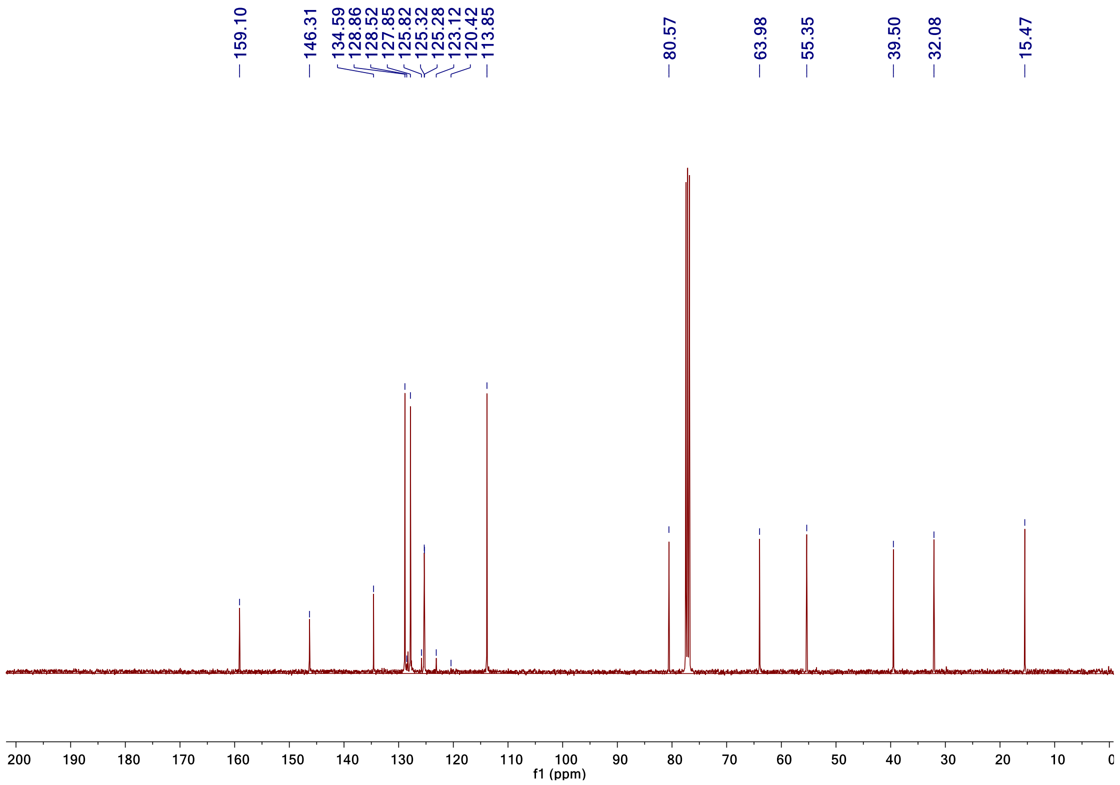

Supplementary Figure 109. ^{13}C NMR (100 MHz, $CDCl_3$) spectrum for 20

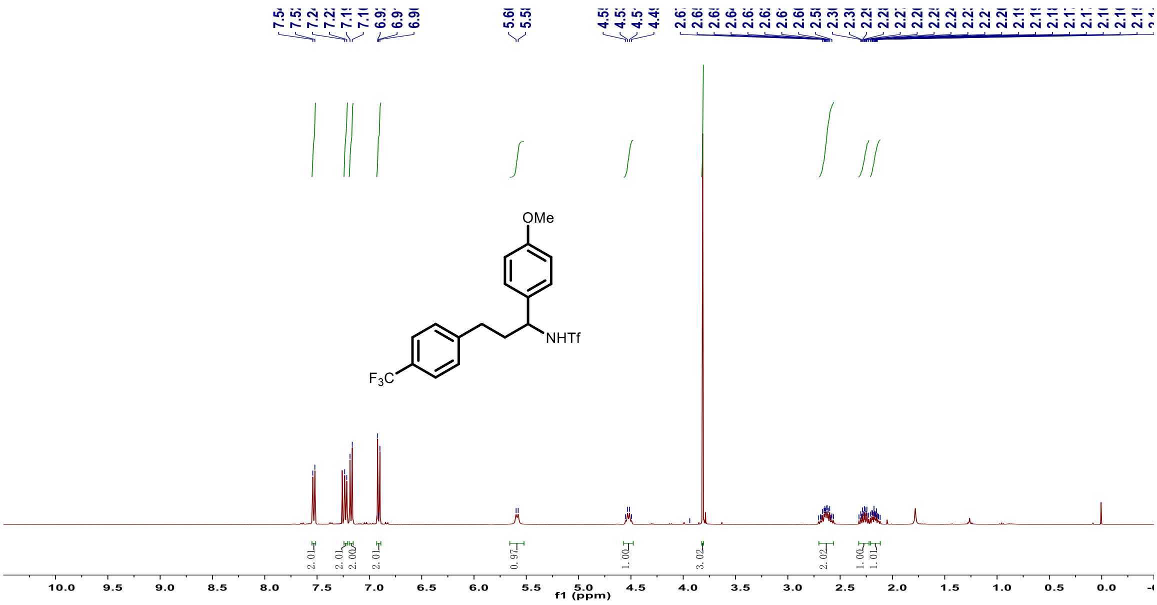

Supplementary Figure 110. ^{1}H NMR (400 MHz, $CDCl_3$) spectrum for 44

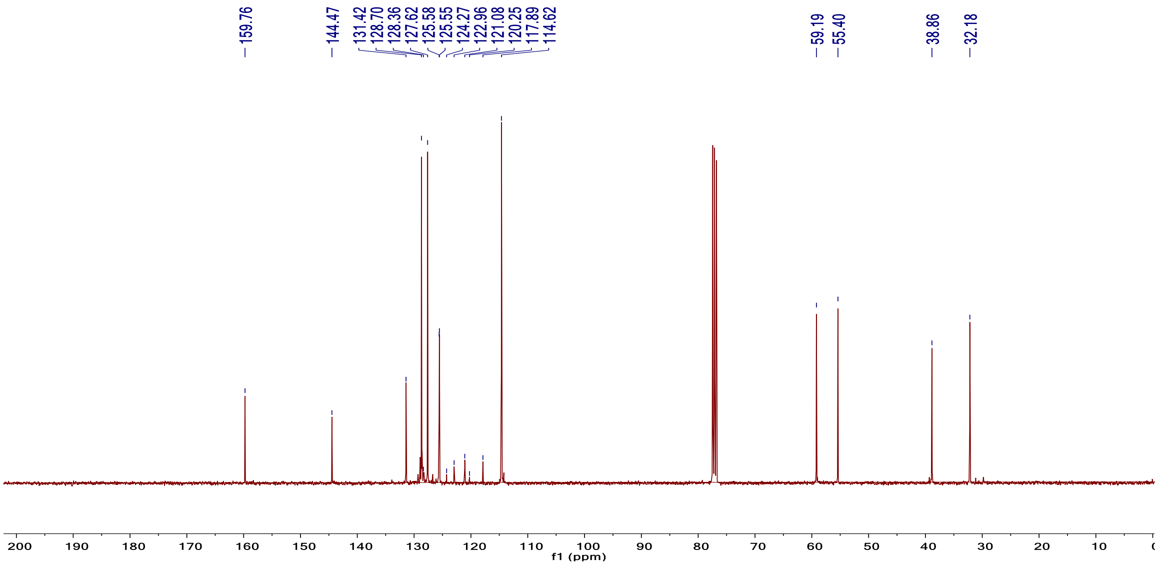

Supplementary Figure 111. ^{13}C NMR (100 MHz, $CDCl_3$) spectrum for 44

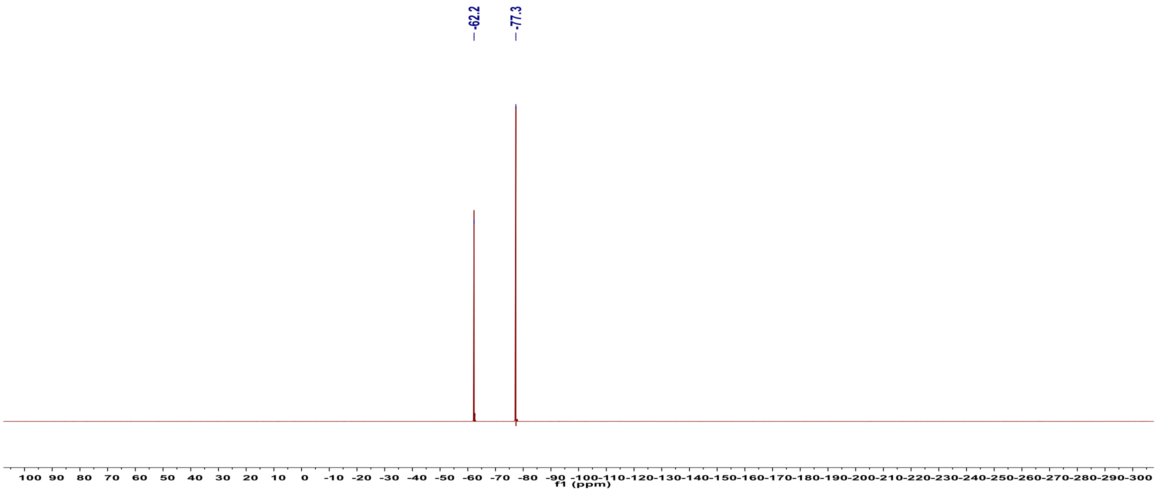

Supplementary Figure 112. ^{19}F NMR (377 MHz, $CDCl_3$) spectrum for 44

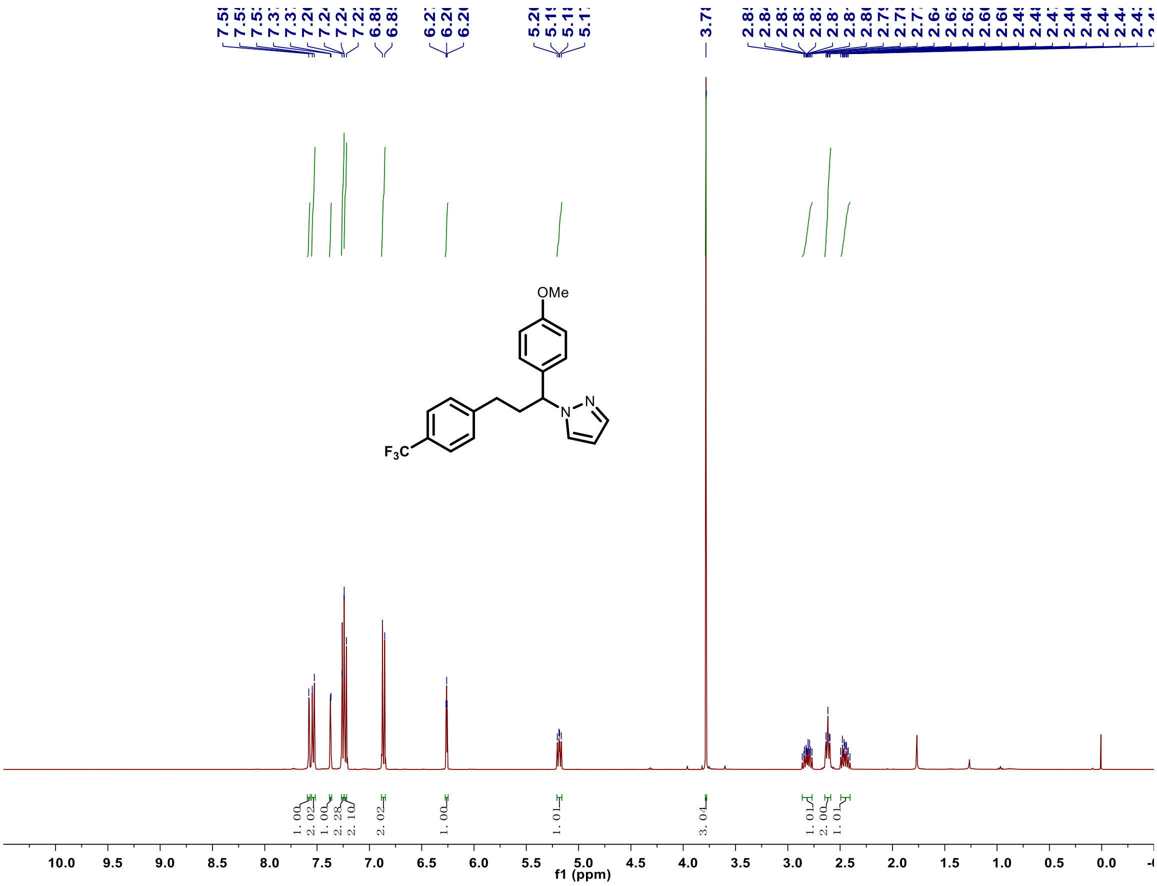

Supplementary Figure 113. ^{1}H NMR (400 MHz, $CDCl_3$) spectrum for 45

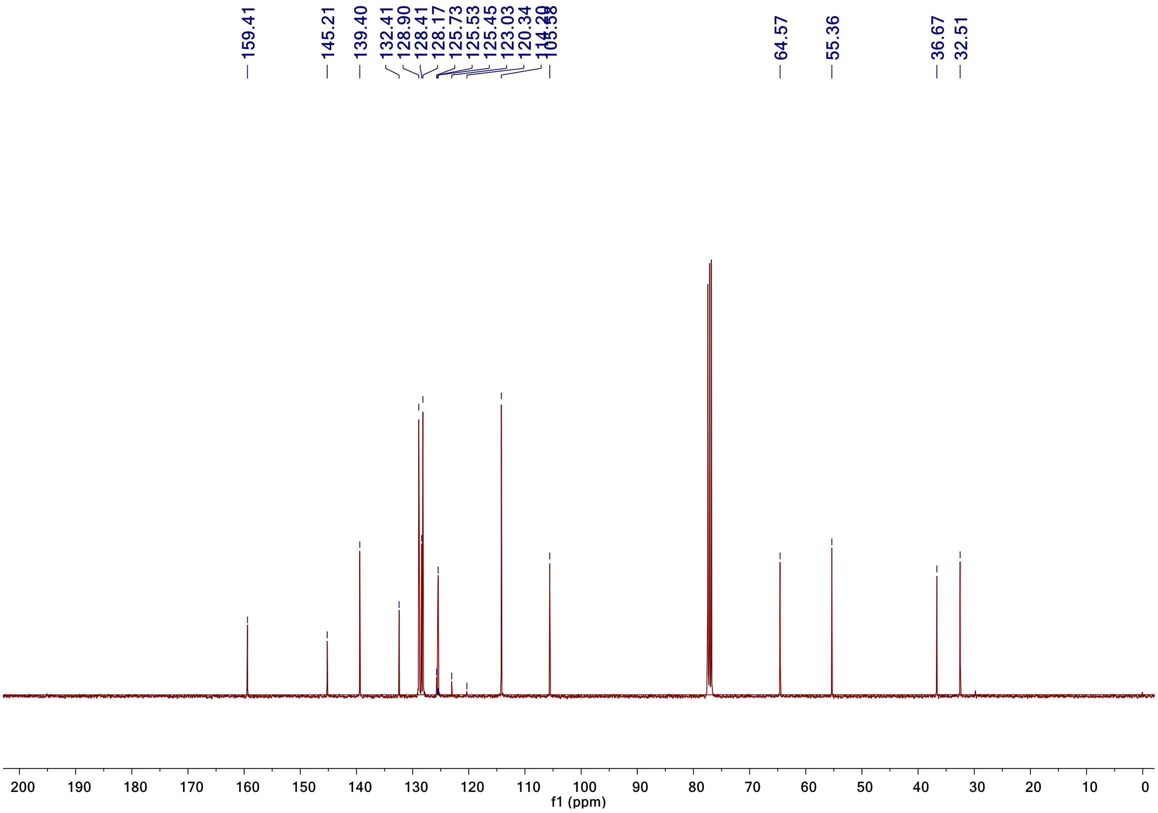

Supplementary Figure 114. ^{13}C NMR (100 MHz, $CDCl_3$) spectrum for 45

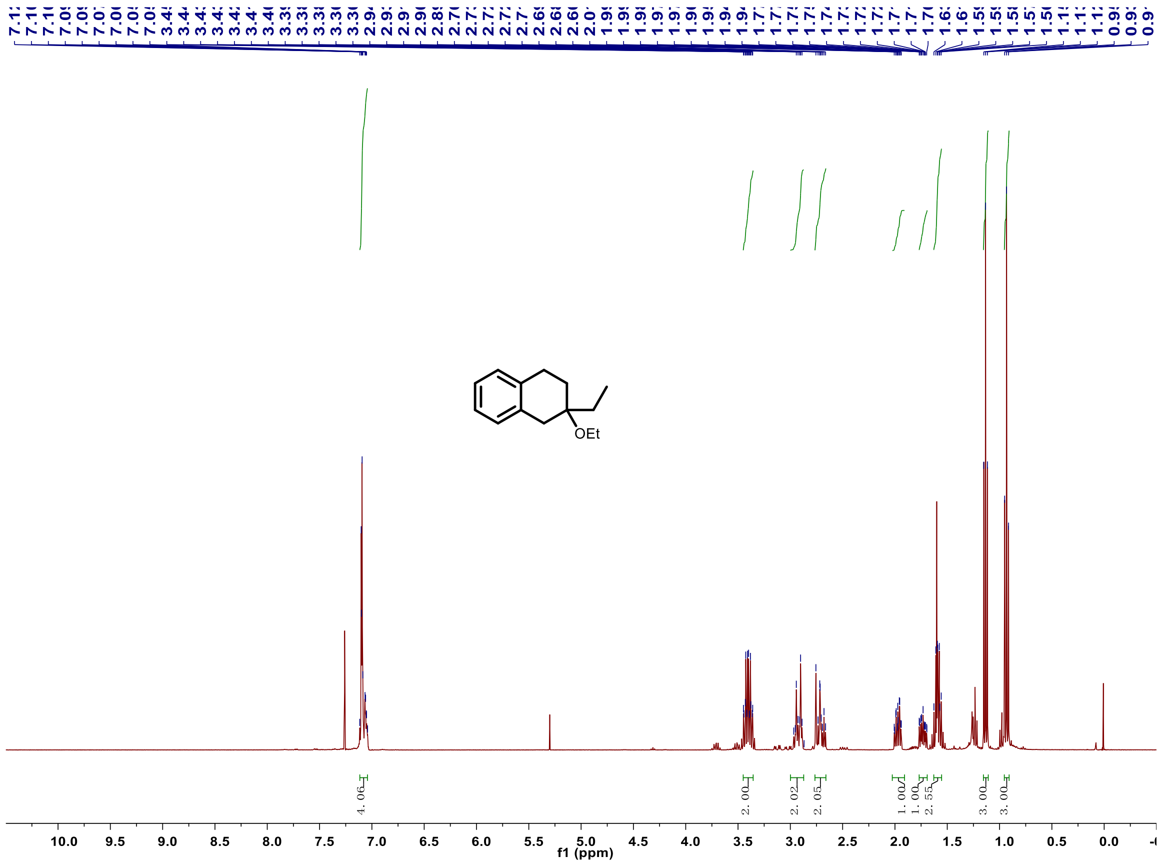

Supplementary Figure 115. ^{1}H **NMR (400 MHz,** $CDCl_3$**) spectrum for 21**

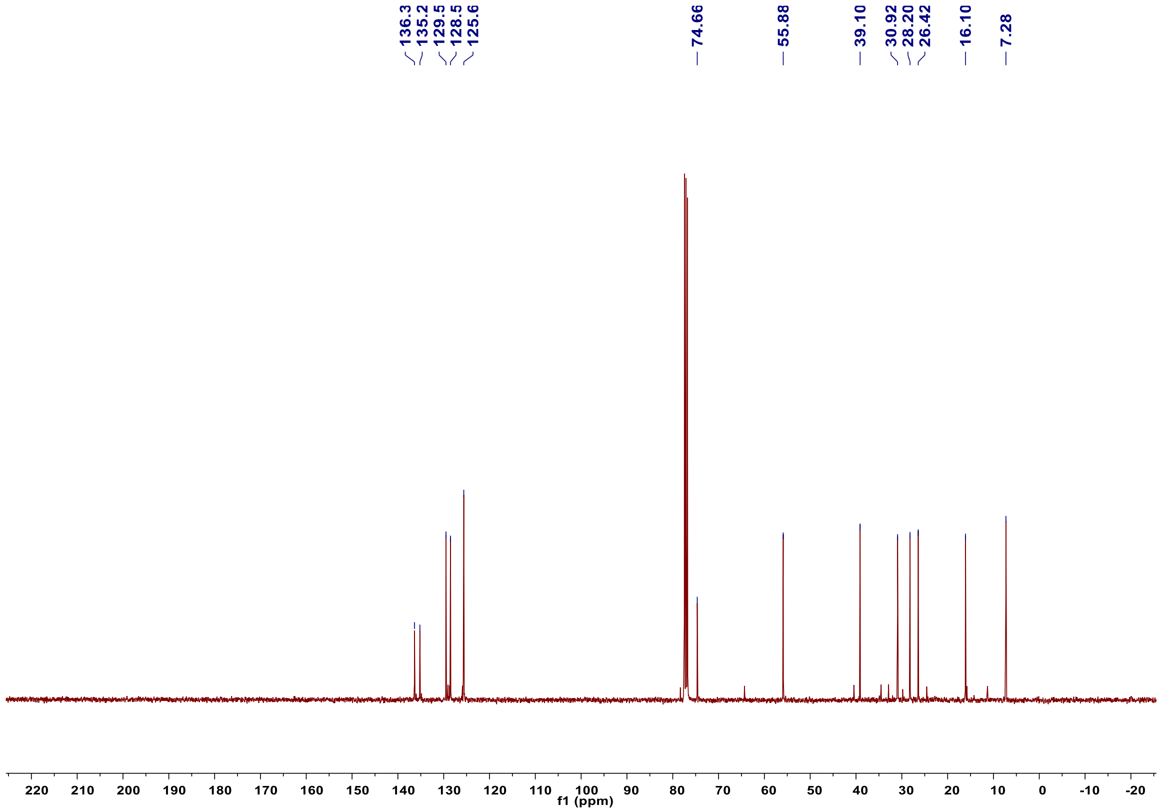

Supplementary Figure 116. ^{13}C **NMR (100 MHz,** $CDCl_3$**) spectrum for 21**

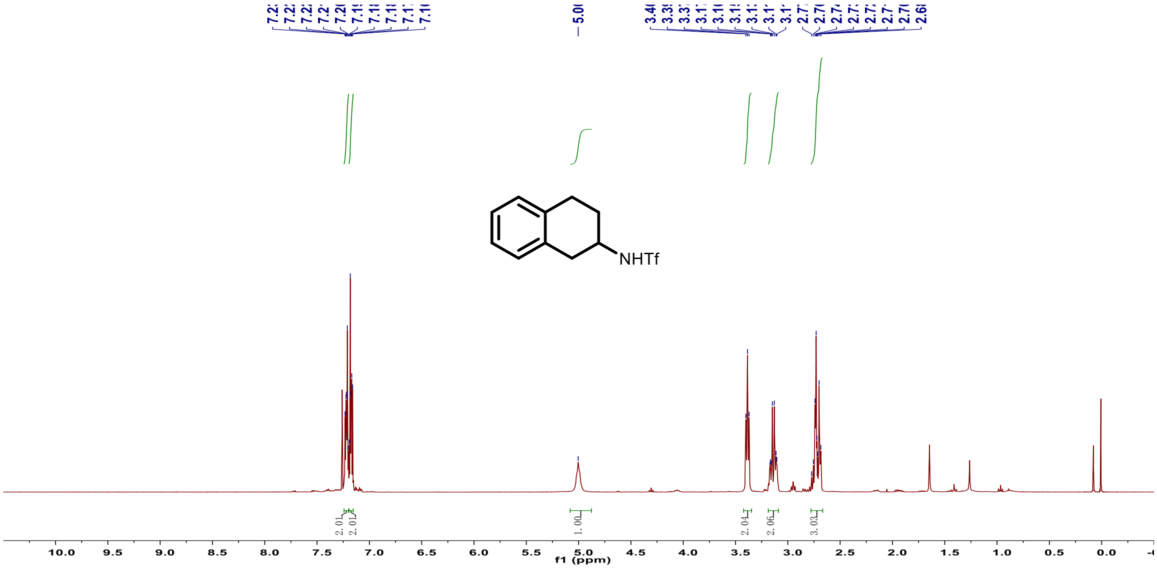

Supplementary Figure 117. ^{1}H NMR (400 MHz, $CDCl_3$) spectrum for 46

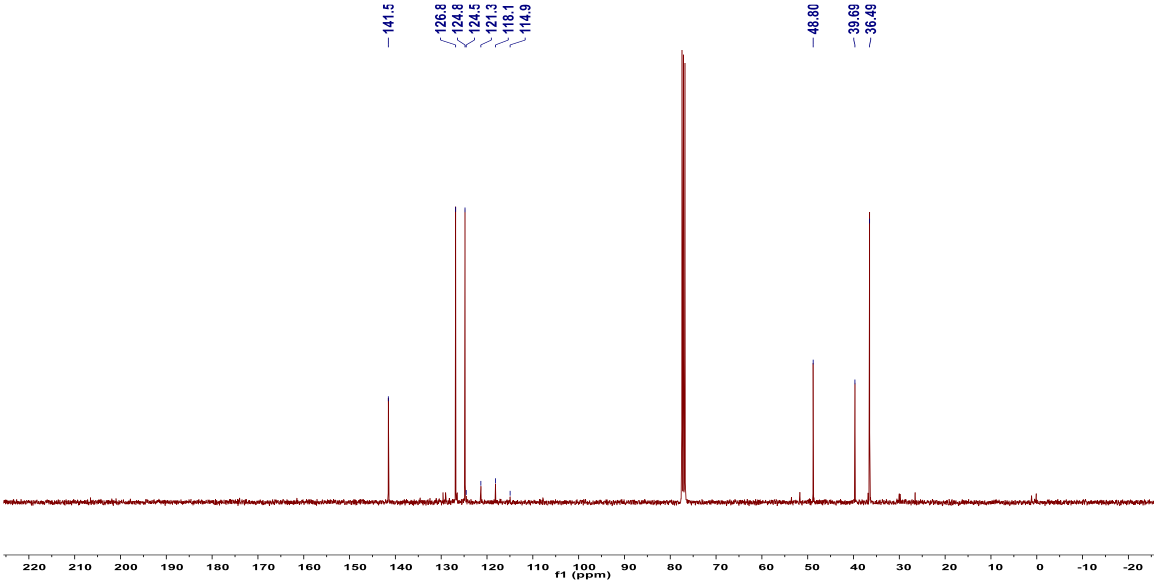

Supplementary Figure 118. ^{13}C NMR (100 MHz, $CDCl_3$) spectrum for 46

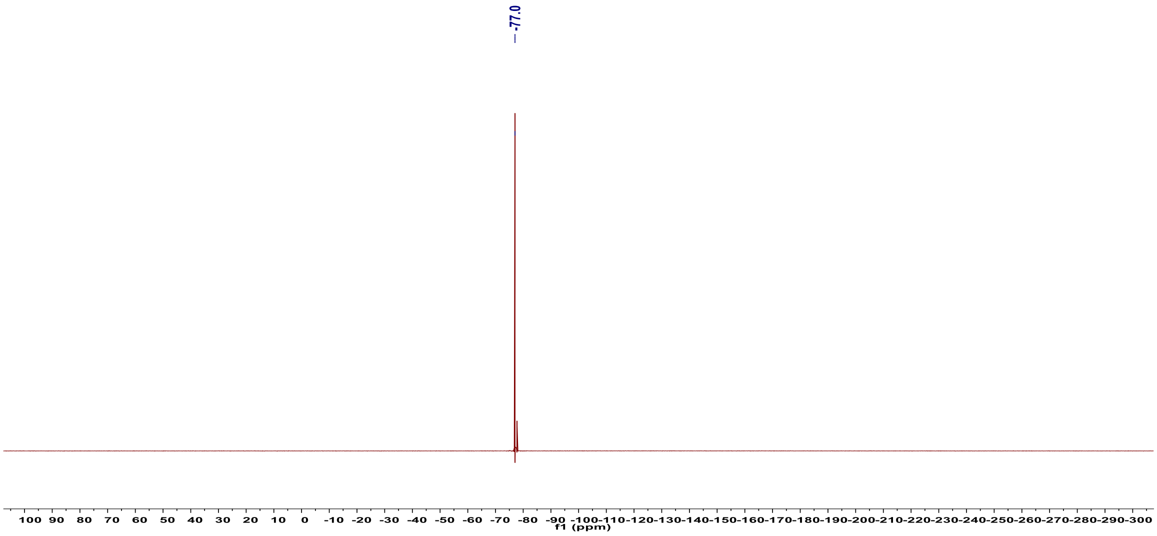

Supplementary Figure 119. ^{19}F NMR (377 MHz, $CDCl_3$) spectrum for 46

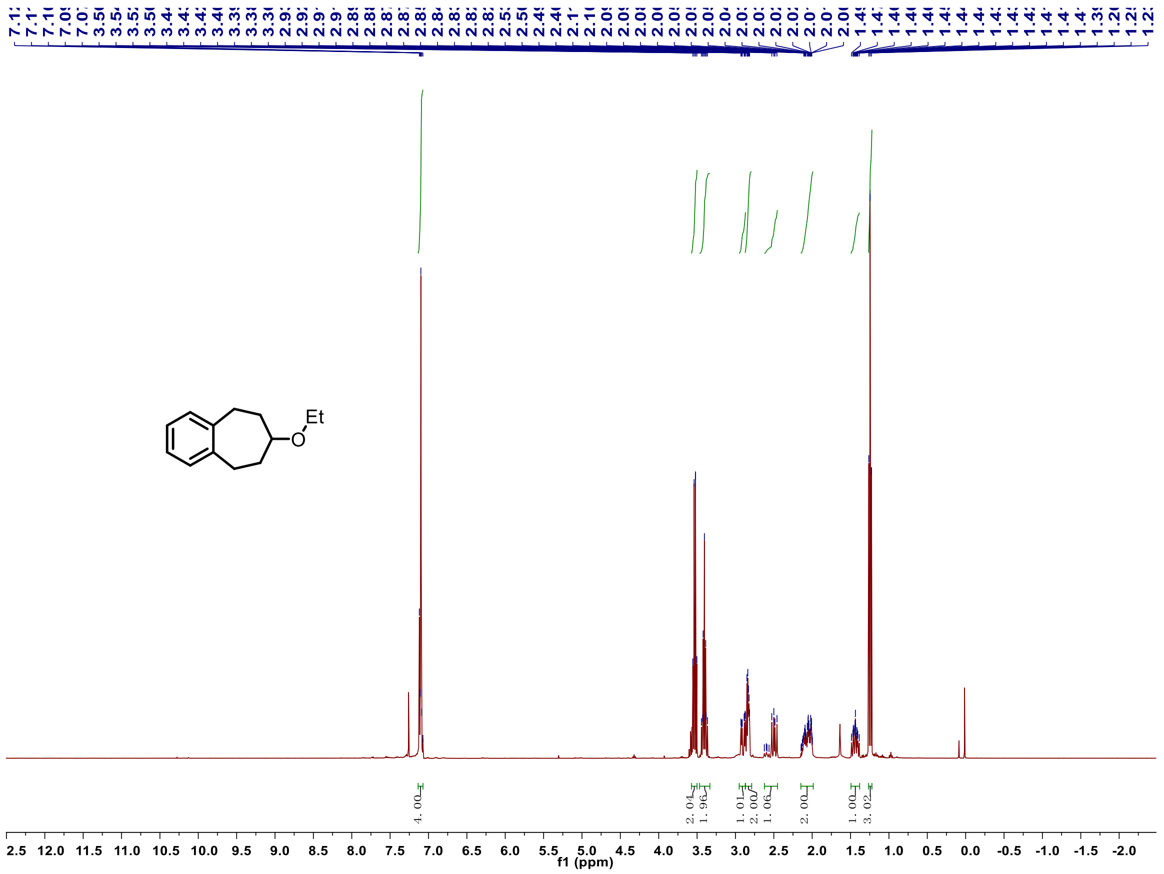

Supplementary Figure 120. ^{1}H NMR (400 MHz, $CDCl_3$) spectrum for 22

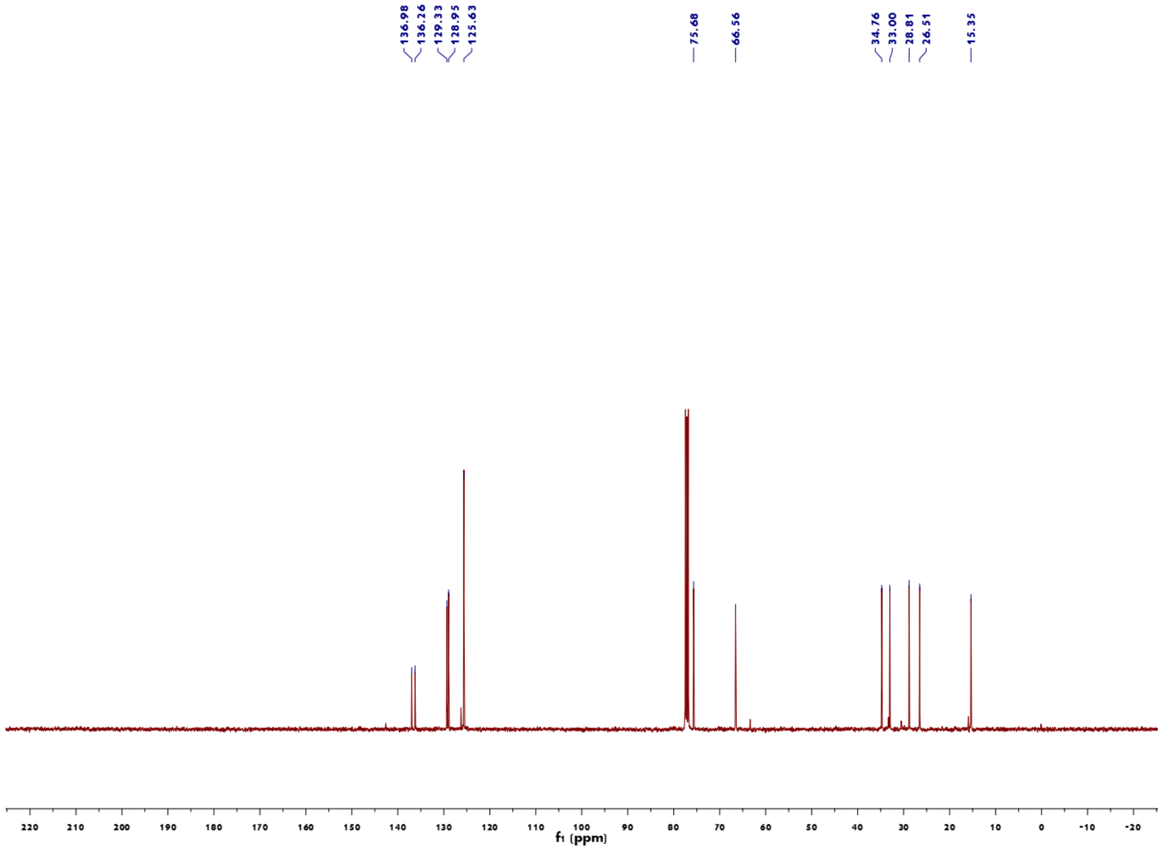

Supplementary Figure 121. ^{13}C NMR (100 MHz, $CDCl_3$) spectrum for 22

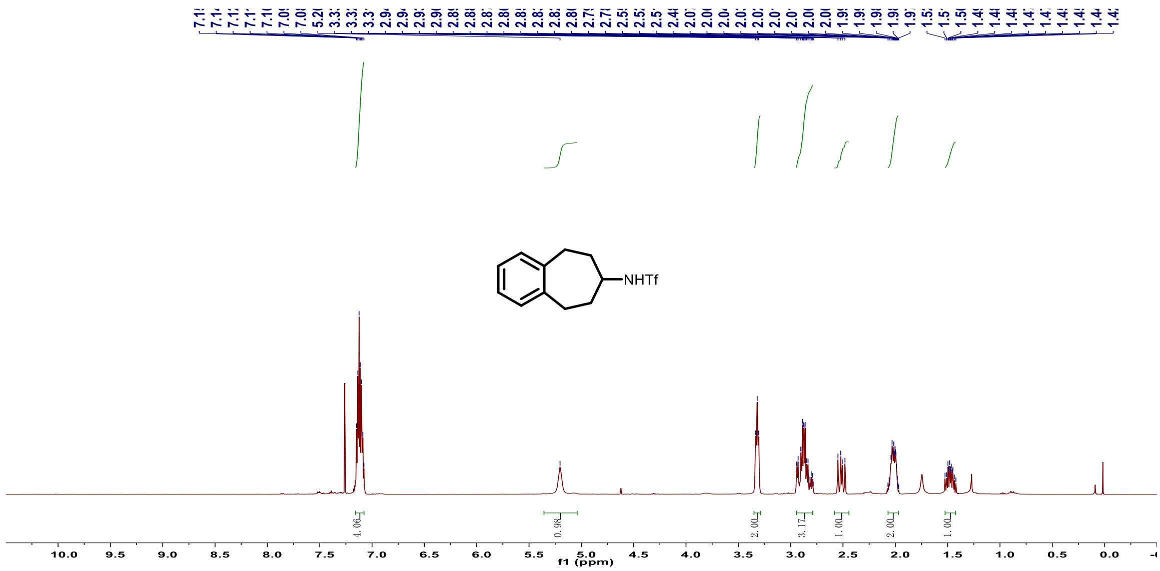

Supplementary Figure 122. ^{1}H NMR (400 MHz, $CDCl_3$) spectrum for 47

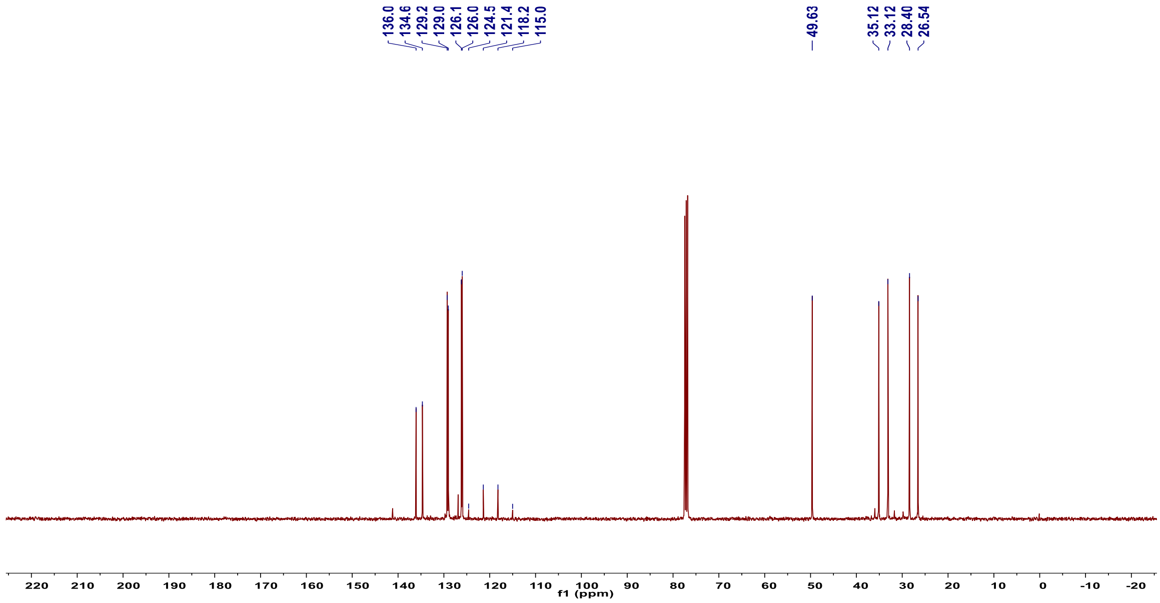

Supplementary Figure 123. ^{13}C NMR (100 MHz, $CDCl_3$) spectrum for 47

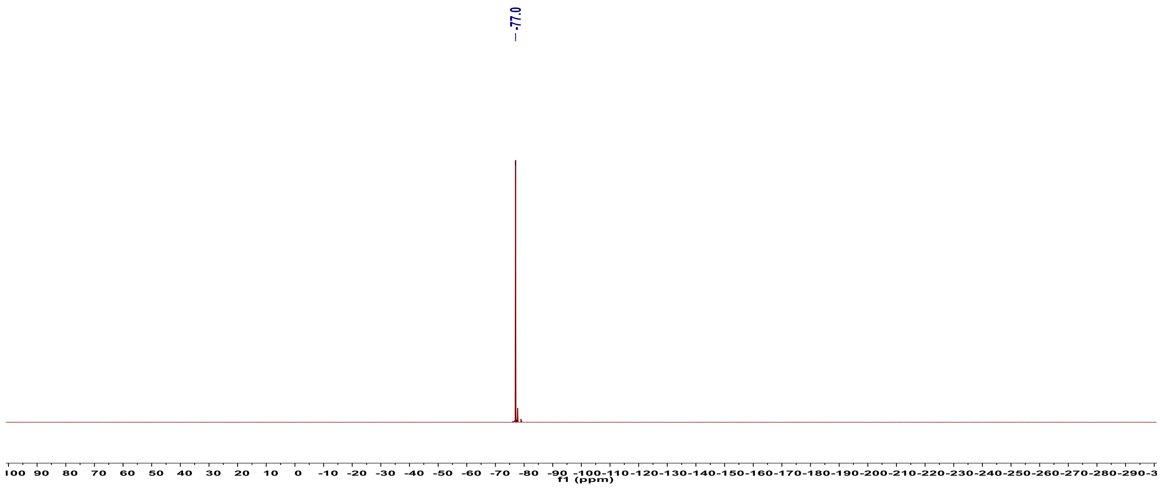

Supplementary Figure 124. ^{19}F NMR (377 MHz, $CDCl_3$) spectrum for 47

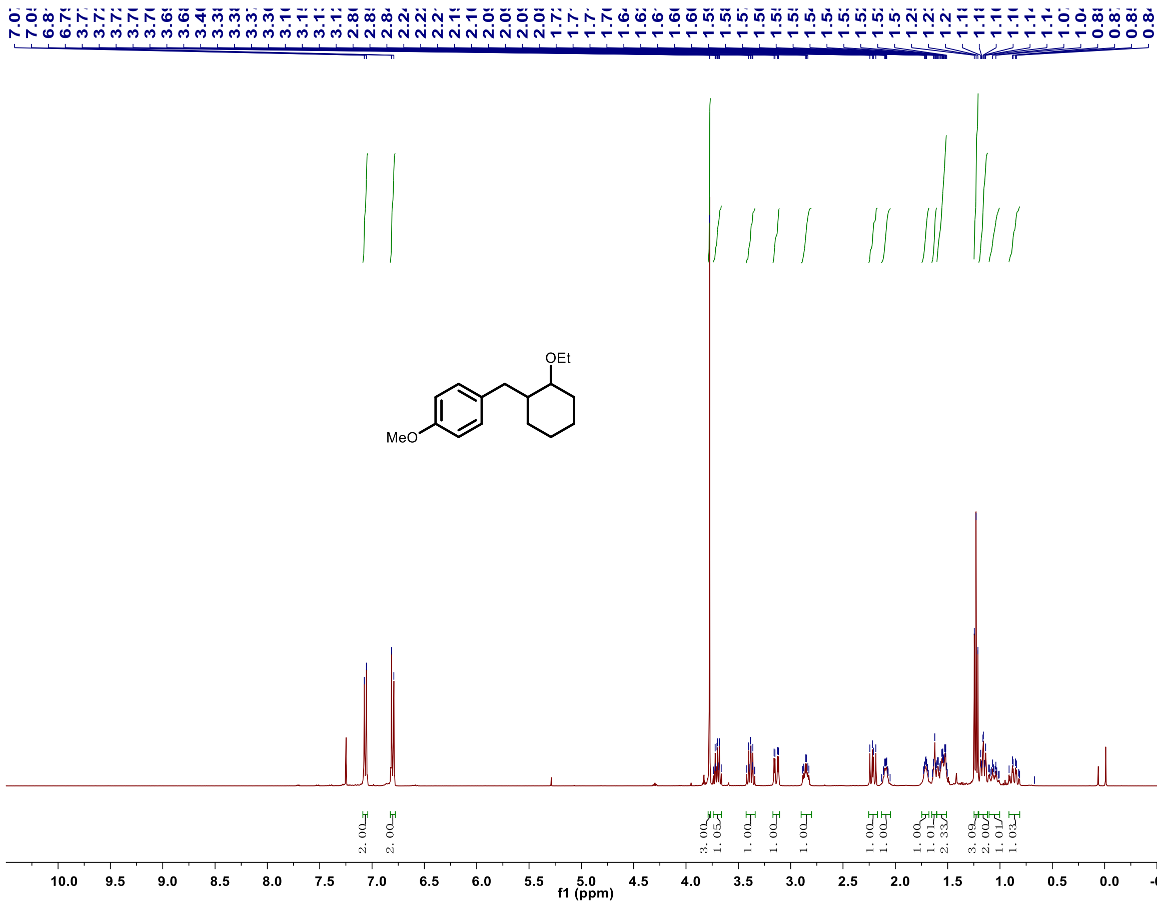

Supplementary Figure 125. **^{1}H NMR (400 MHz, $CDCl_3$) spectrum for 23**

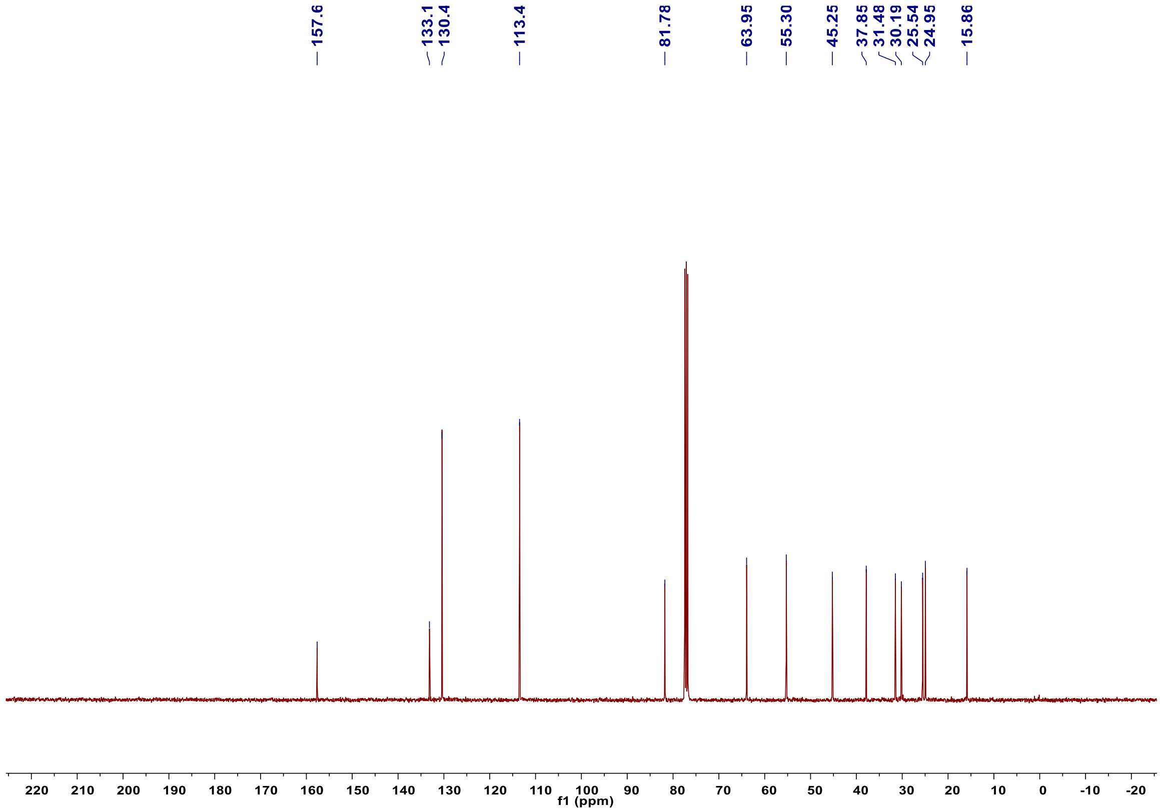

Supplementary Figure 126. **^{13}C NMR (100 MHz, $CDCl_3$) spectrum for 23**

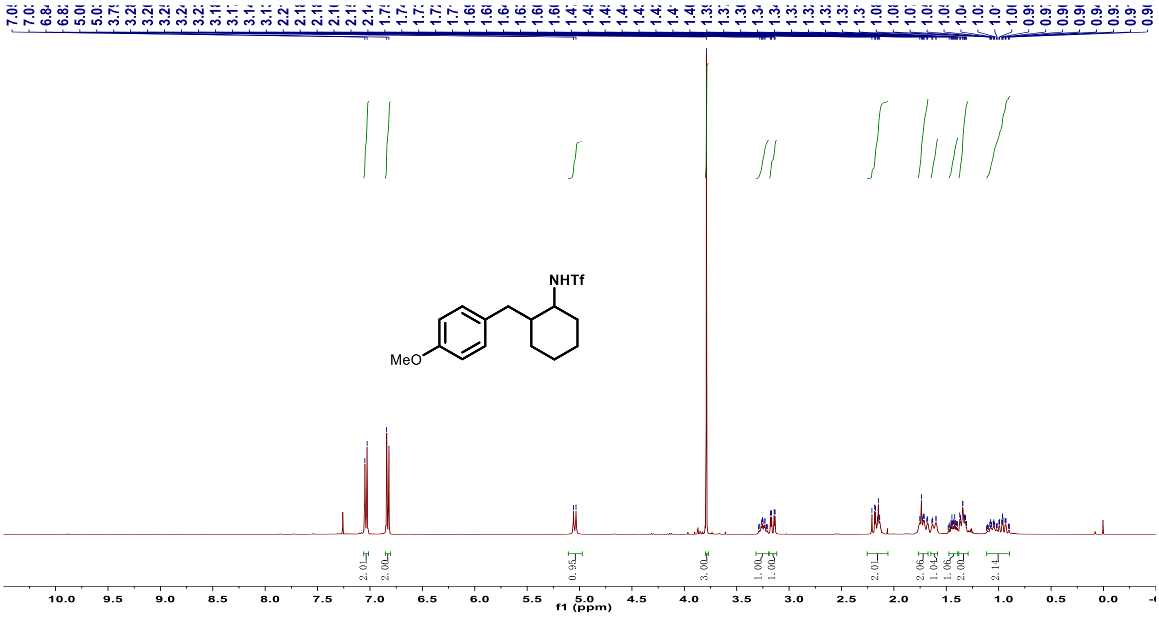

Supplementary Figure 127. ^{1}**H NMR (400 MHz, CDCl$_3$) spectrum for 48**

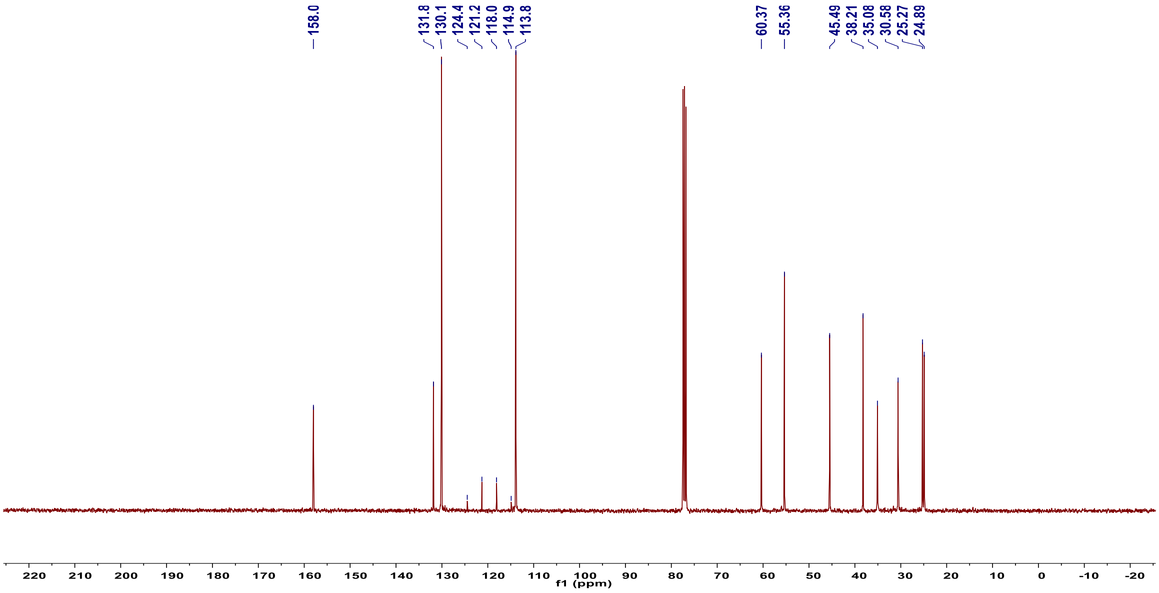

Supplementary Figure 128. ^{13}**C NMR (100 MHz, CDCl$_3$) spectrum for 48**

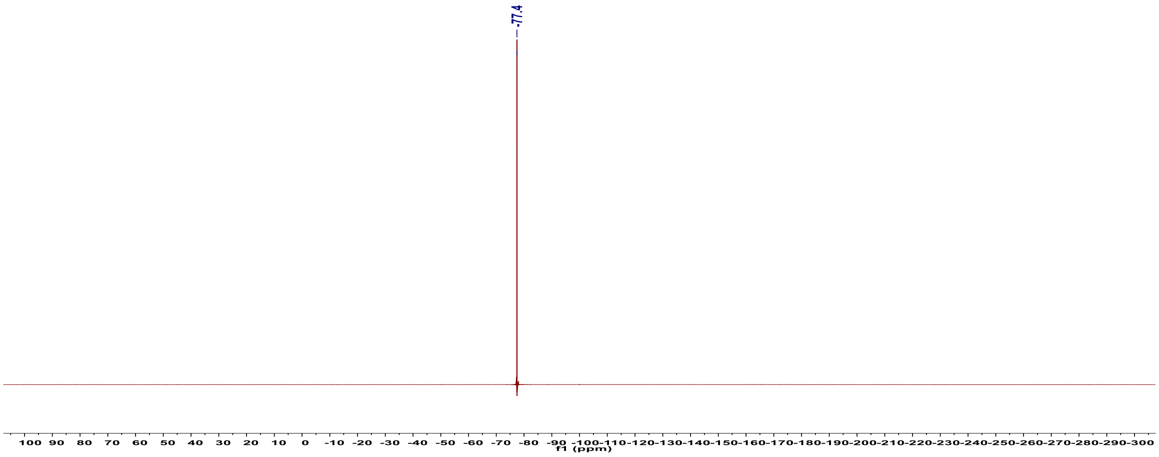

Supplementary Figure 129. ^{19}**F NMR (377 MHz, CDCl$_3$) spectrum for 48**

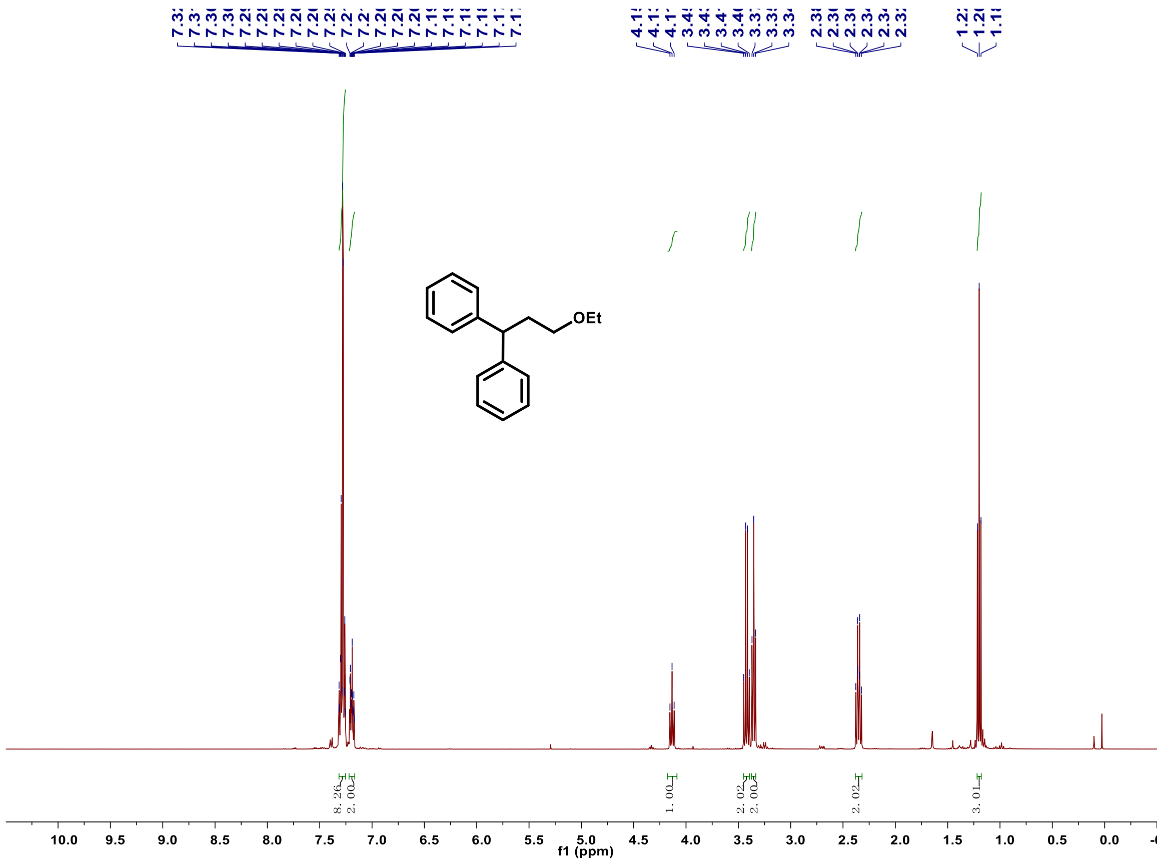

Supplementary Figure 130. ^{1}H NMR (400 MHz, $CDCl_3$) spectrum for 24

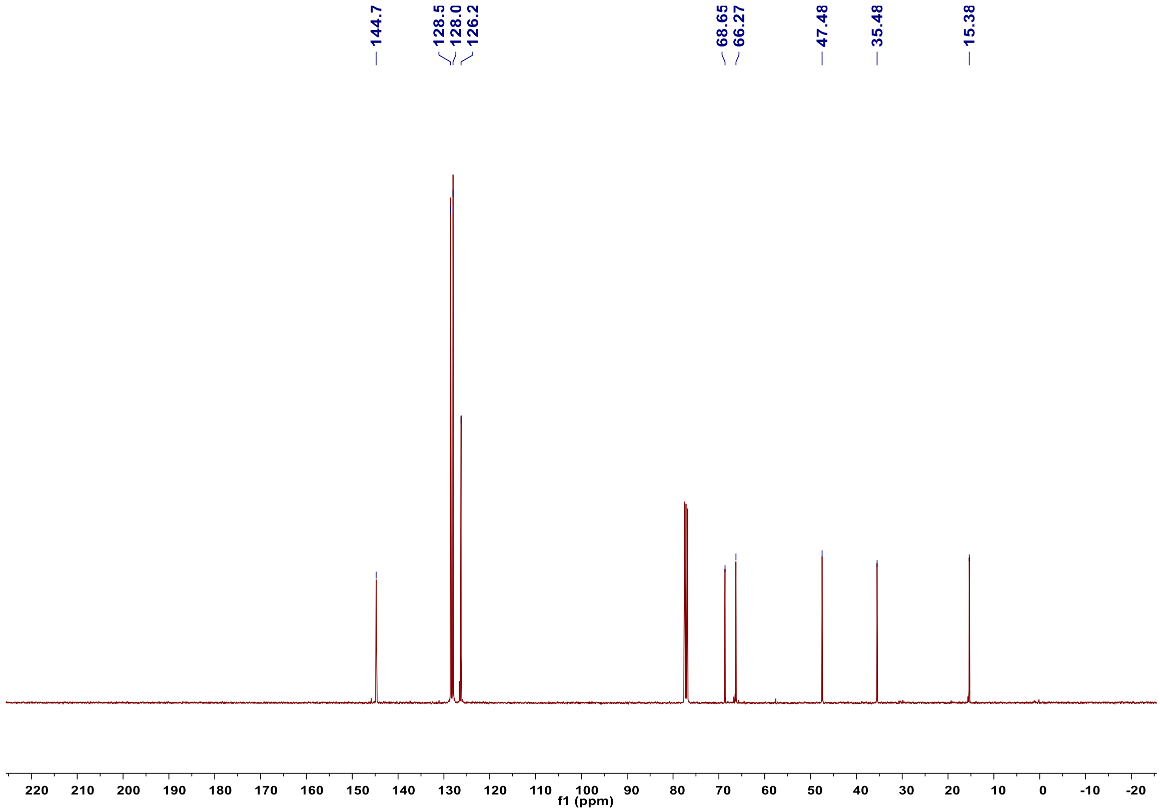

Supplementary Figure 131. ^{13}C NMR (100 MHz, $CDCl_3$) spectrum for 24

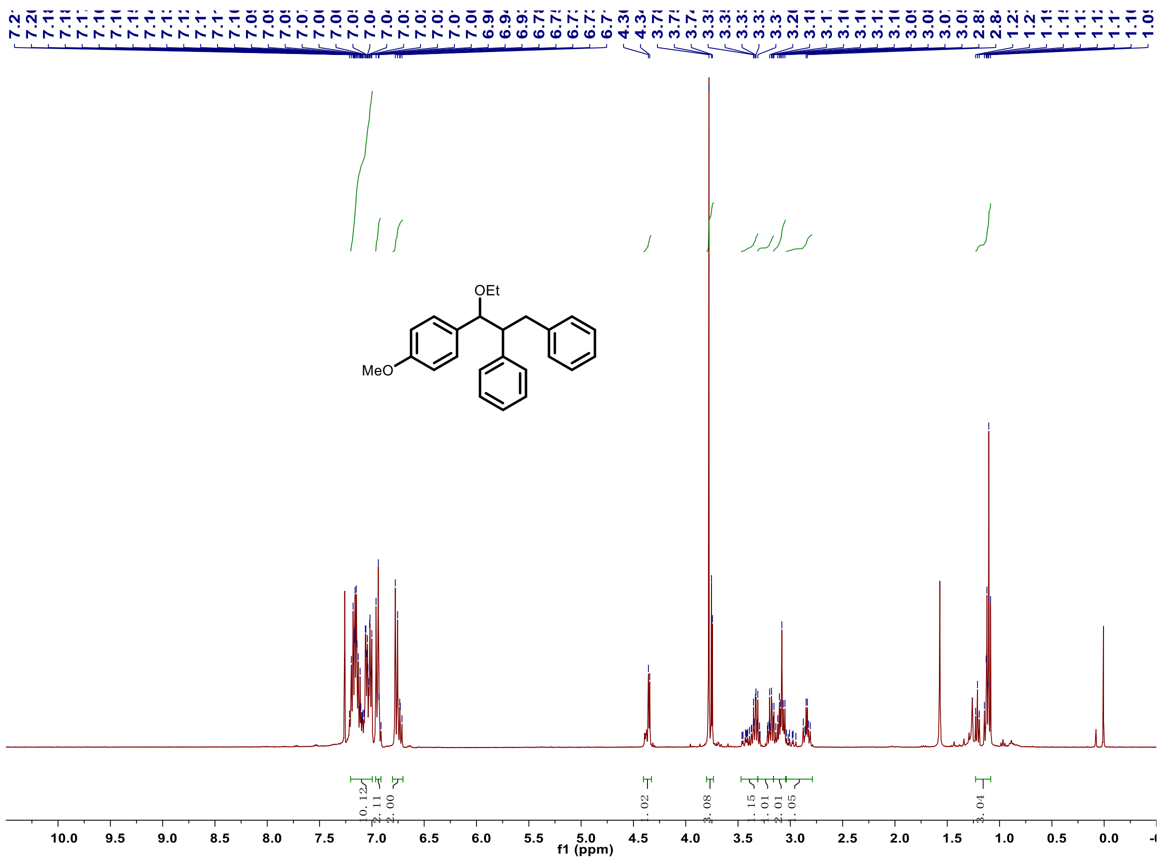

Supplementary Figure 132. ^{1}H NMR (400 MHz, $CDCl_3$) spectrum for 25

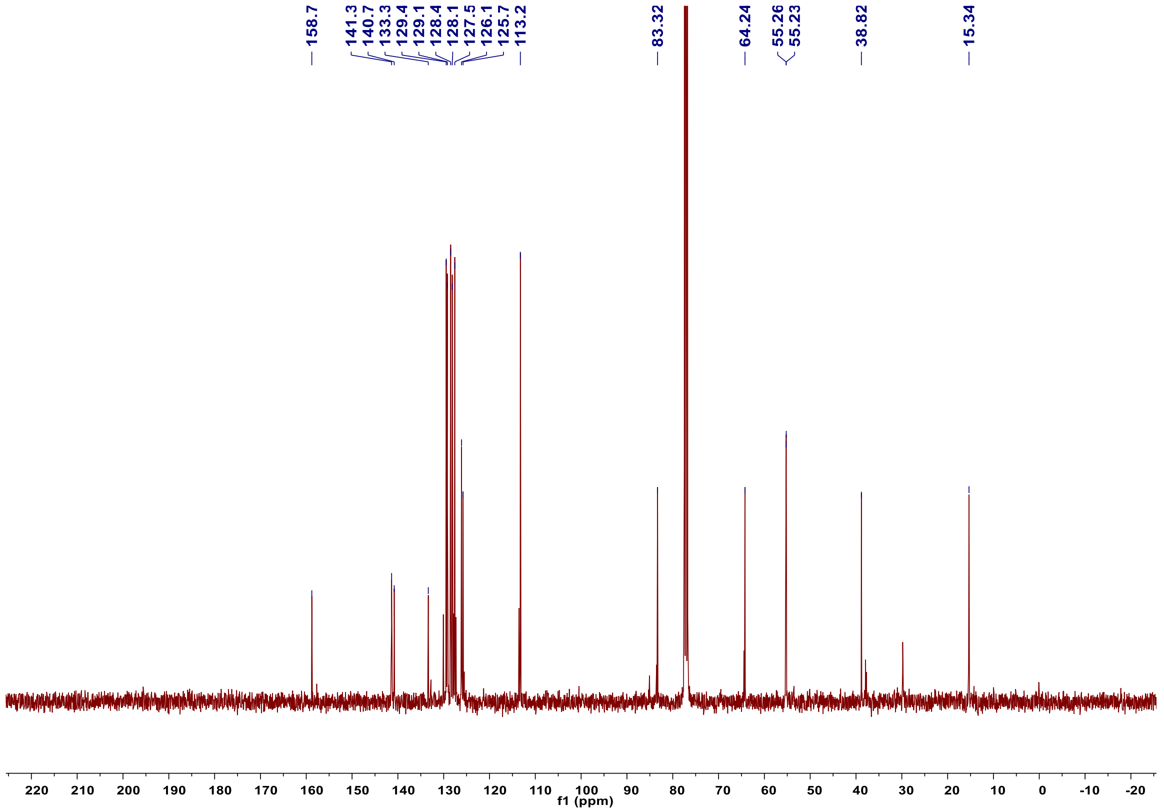

Supplementary Figure 133. ^{13}C NMR (100 MHz, $CDCl_3$) spectrum for 25

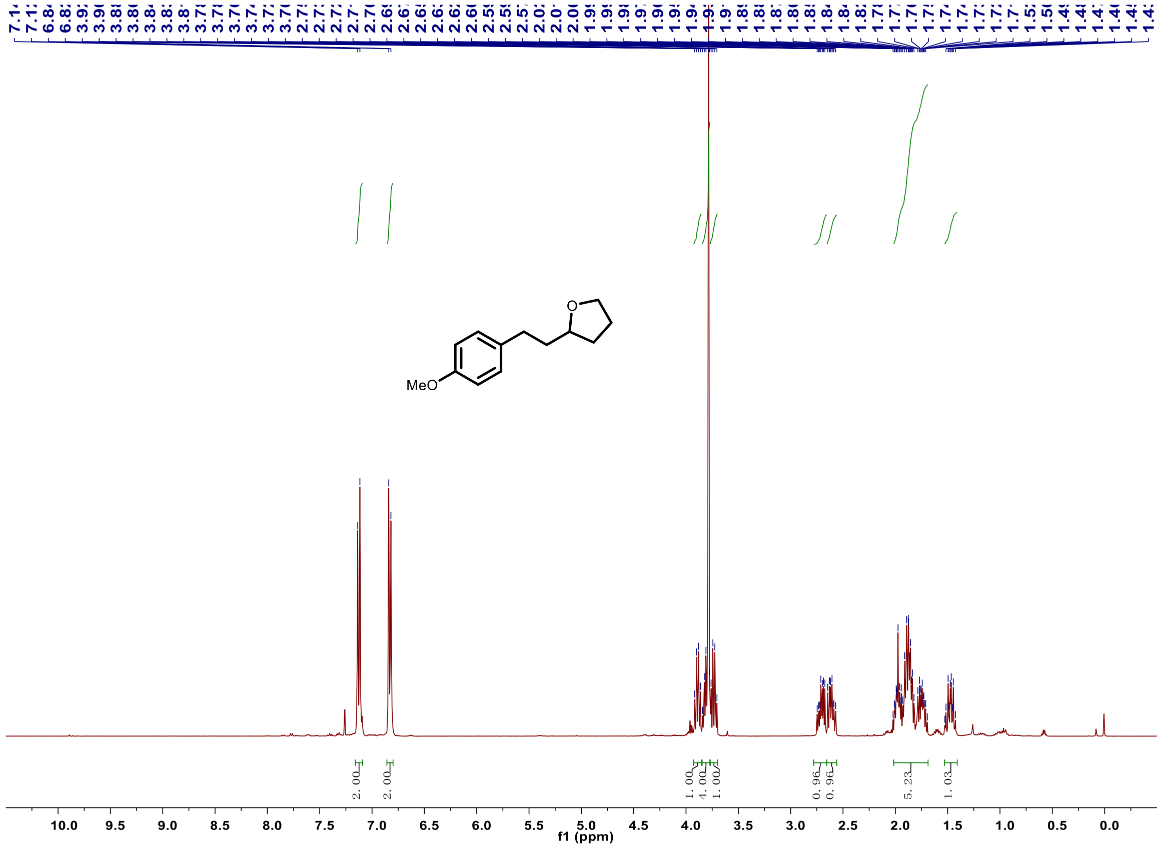

Supplementary Figure 134. ^{1}H NMR (400 MHz, $CDCl_3$) spectrum for 26

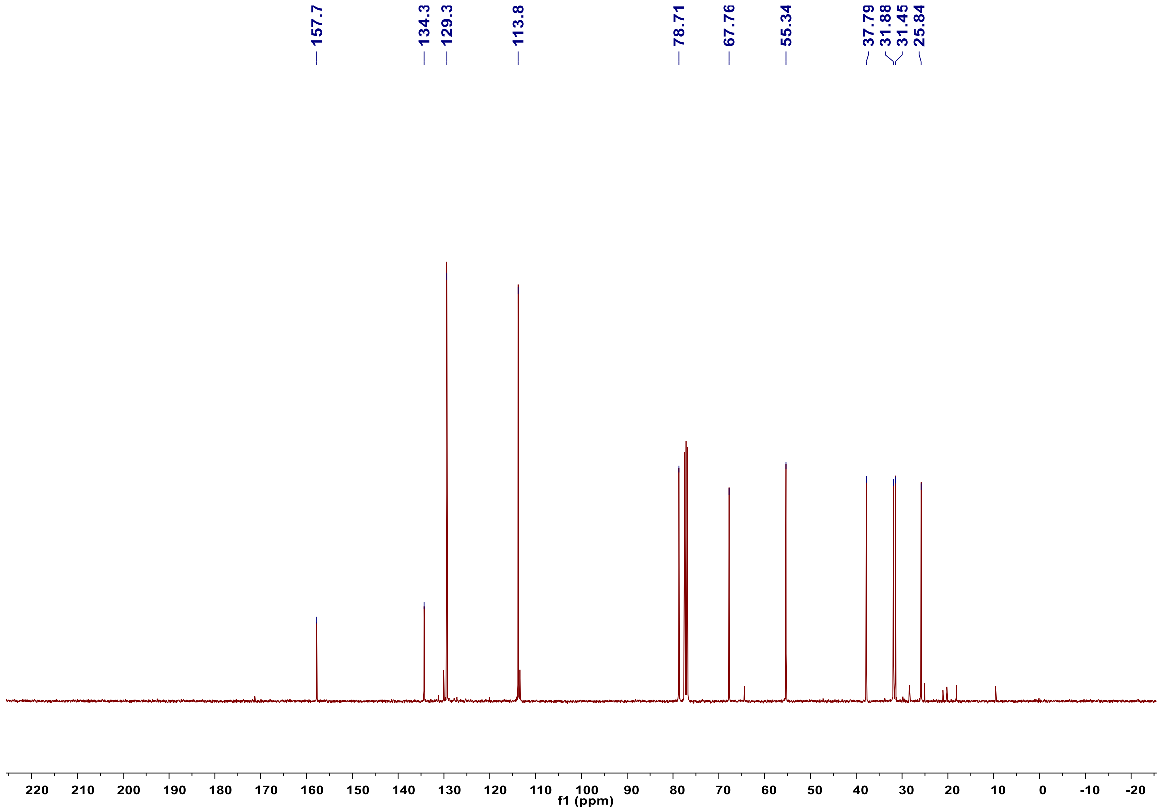

Supplementary Figure 135. ^{13}C NMR (100 MHz, $CDCl_3$) spectrum for 26

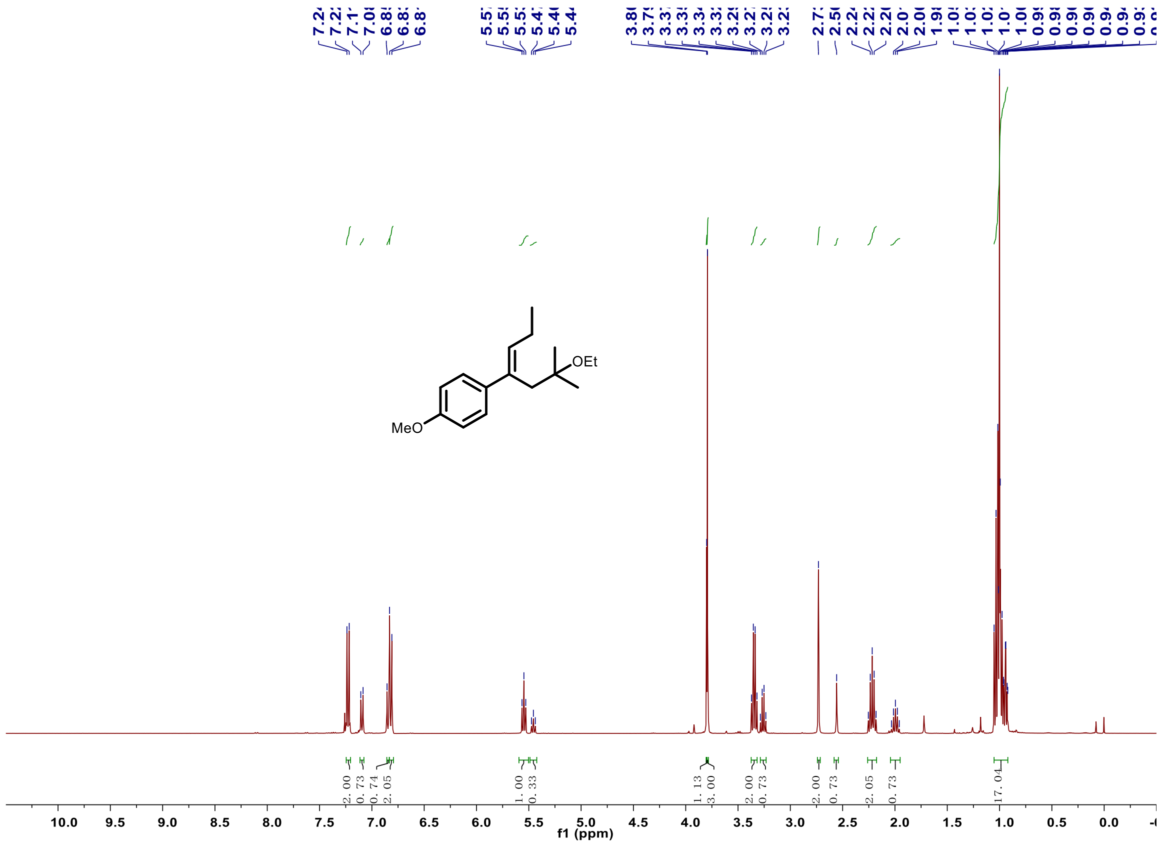

Supplementary Figure 136. ^{1}H NMR (400 MHz, $CDCl_3$) spectrum for 112

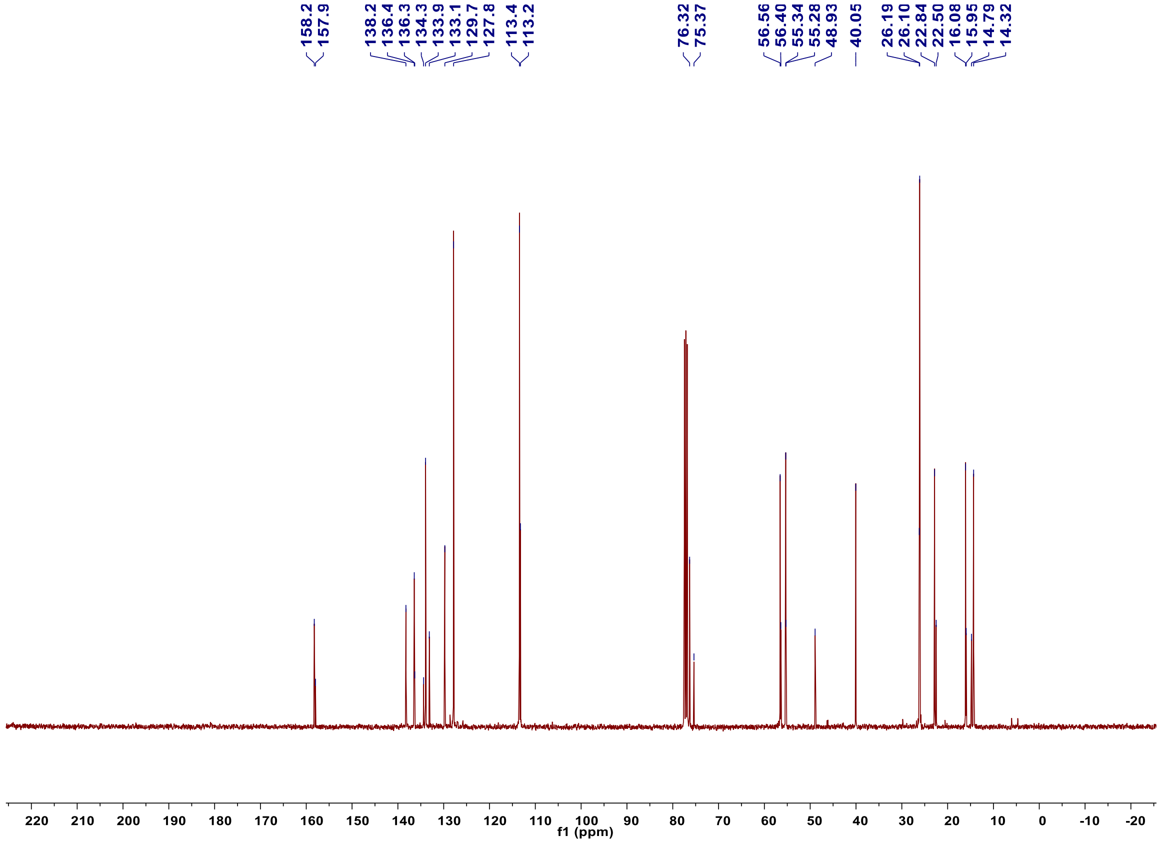

Supplementary Figure 137. ^{13}C NMR (100 MHz, $CDCl_3$) spectrum for 112

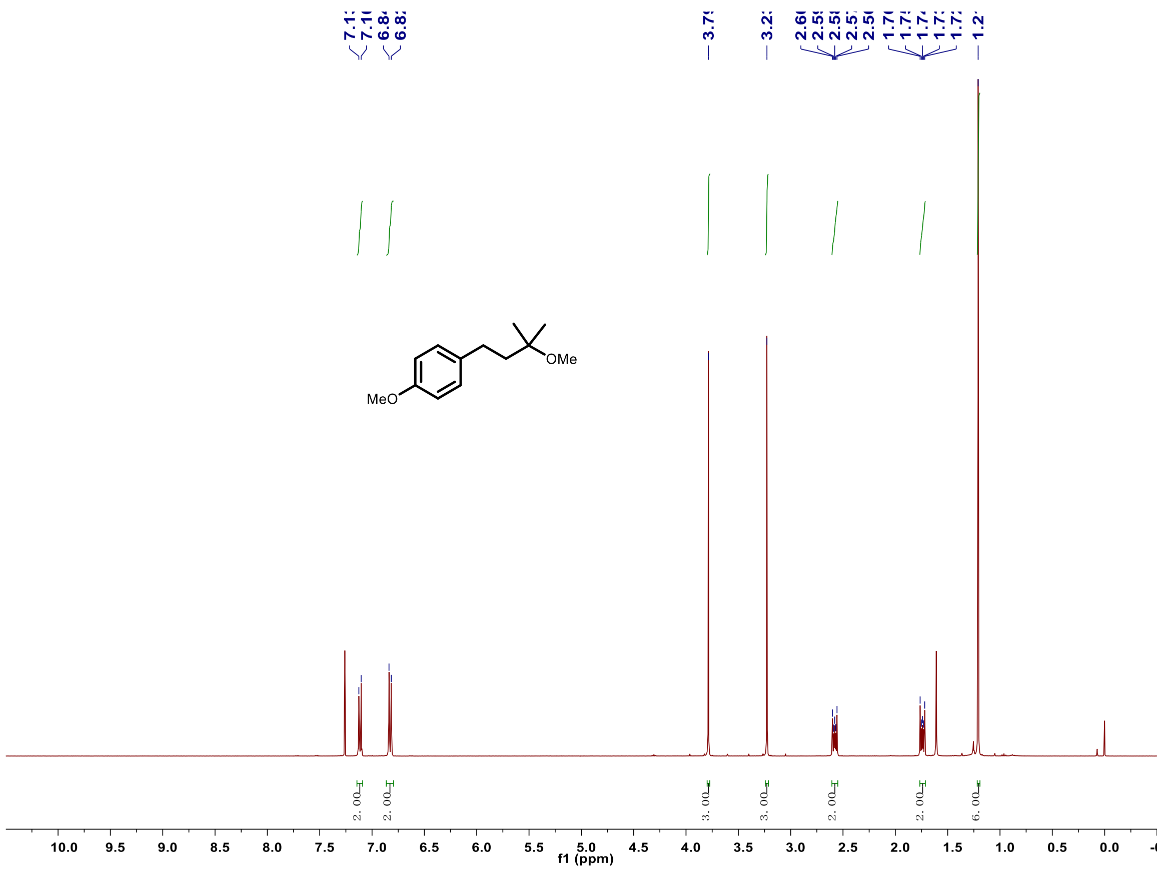

Supplementary Figure 138. ^{13}C NMR (100 MHz, $CDCl_3$) spectrum for 49

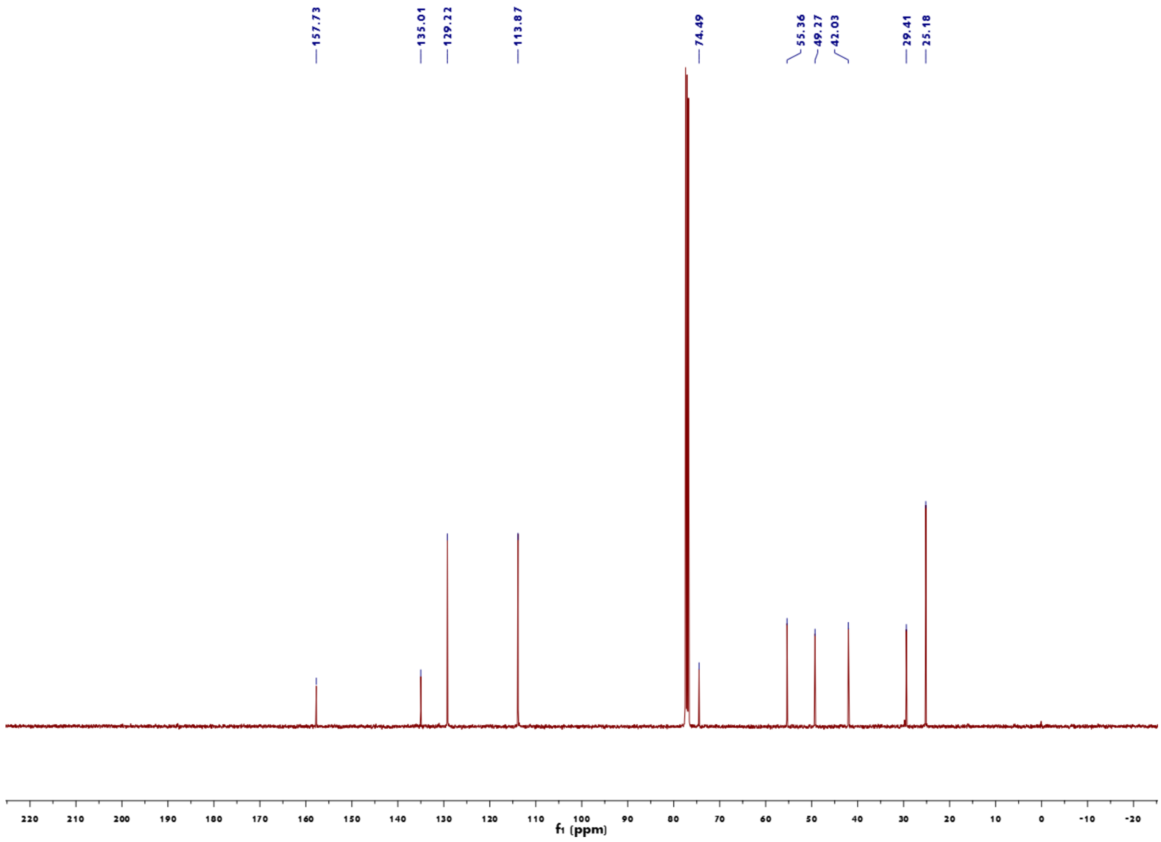

Supplementary Figure 139. ^{13}C NMR (100 MHz, $CDCl_3$) spectrum for 49

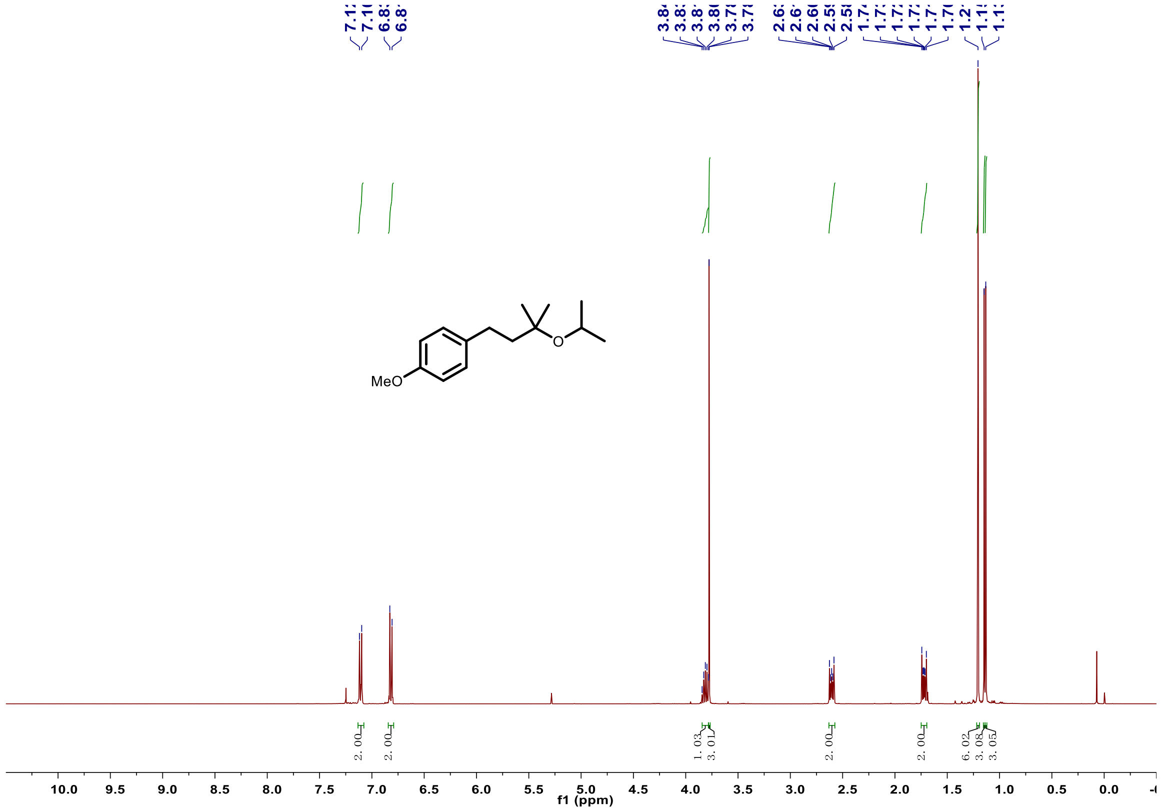

Supplementary Figure 140. ^{1}H NMR (400 MHz, $CDCl_3$) spectrum for 50

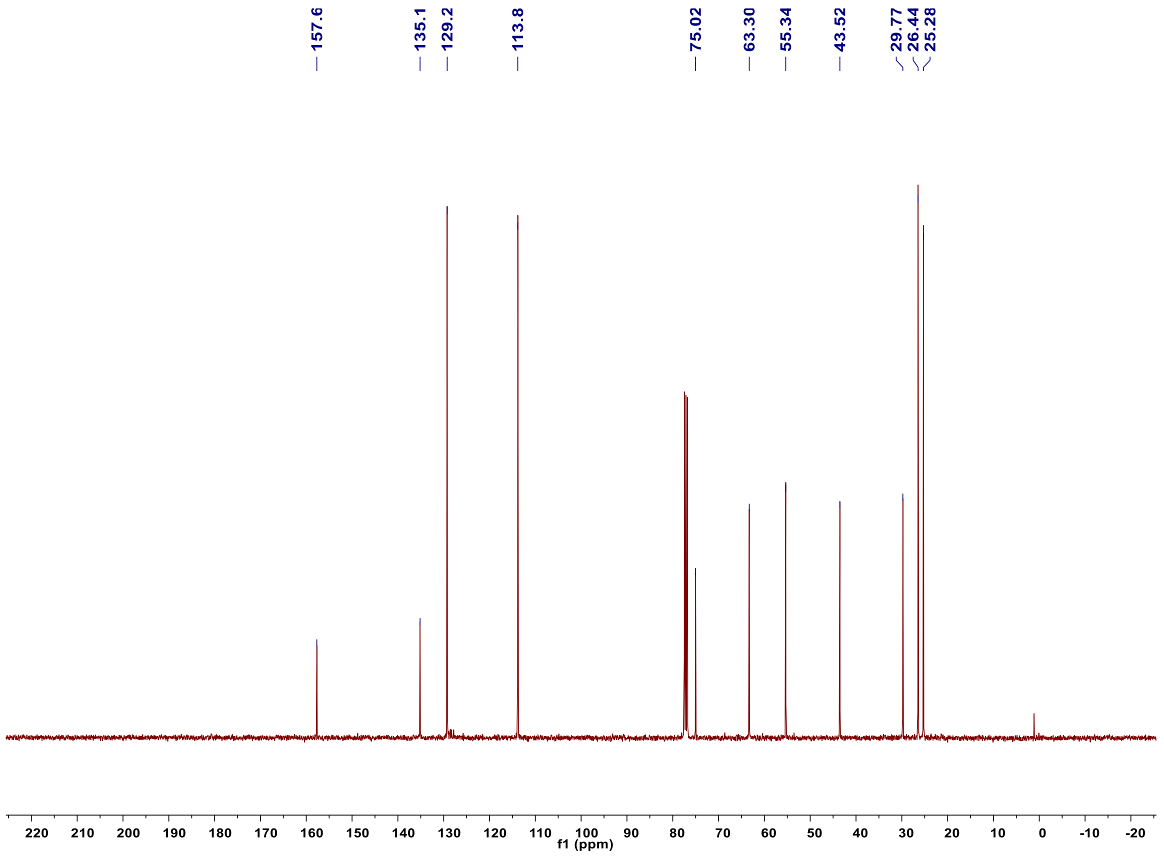

Supplementary Figure 141. ^{13}C NMR (100 MHz, $CDCl_3$) spectrum for 50

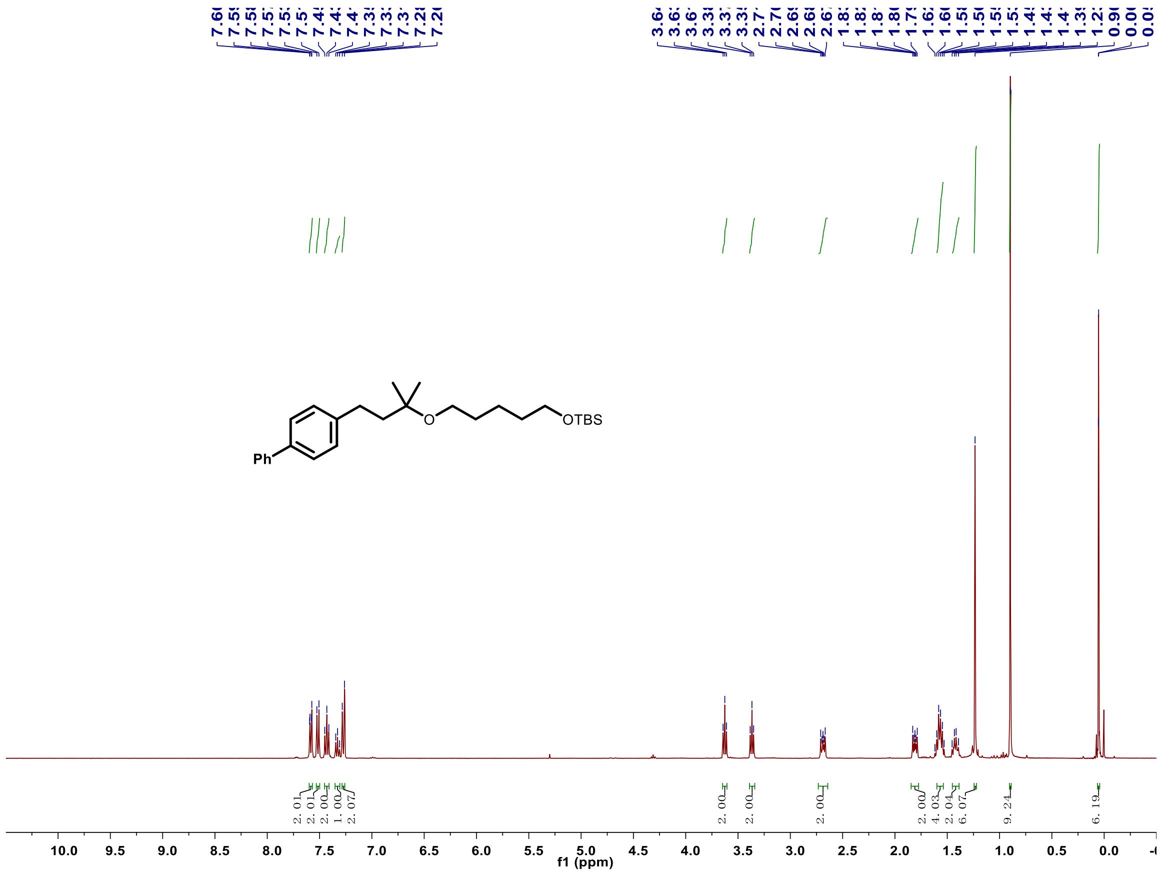

Supplementary Figure 142. ^{1}H NMR (400 MHz, $CDCl_3$) spectrum for 51

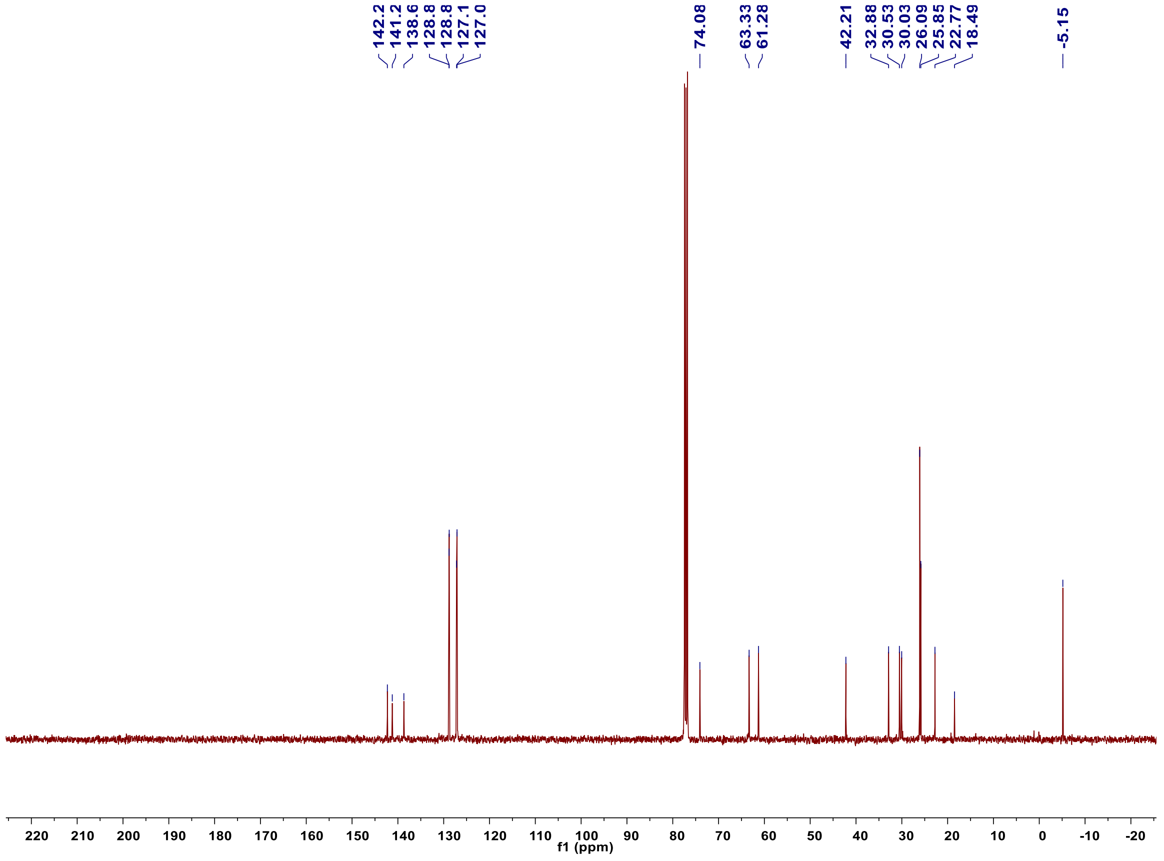

Supplementary Figure 143. ^{13}C NMR (100 MHz, $CDCl_3$) spectrum for 51

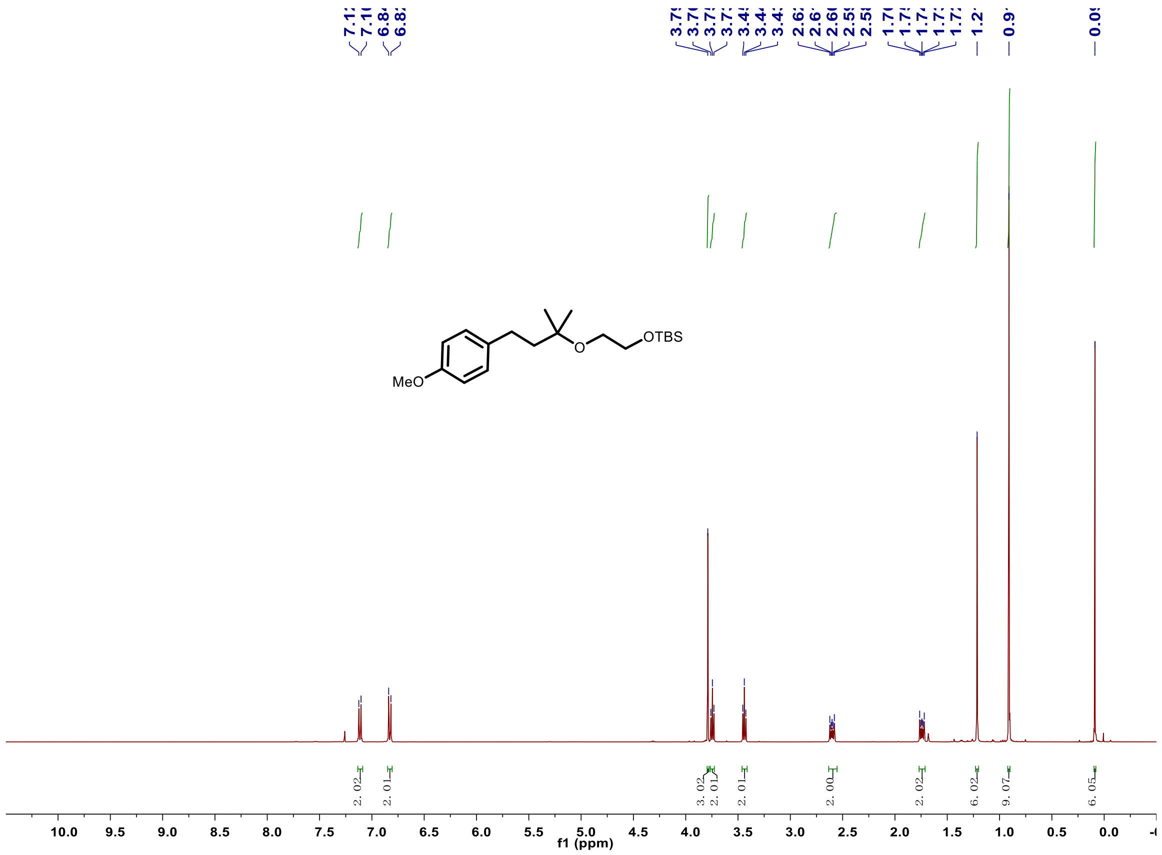

Supplementary Figure 144. ^{1}H NMR (400 MHz, $CDCl_3$) spectrum for 52

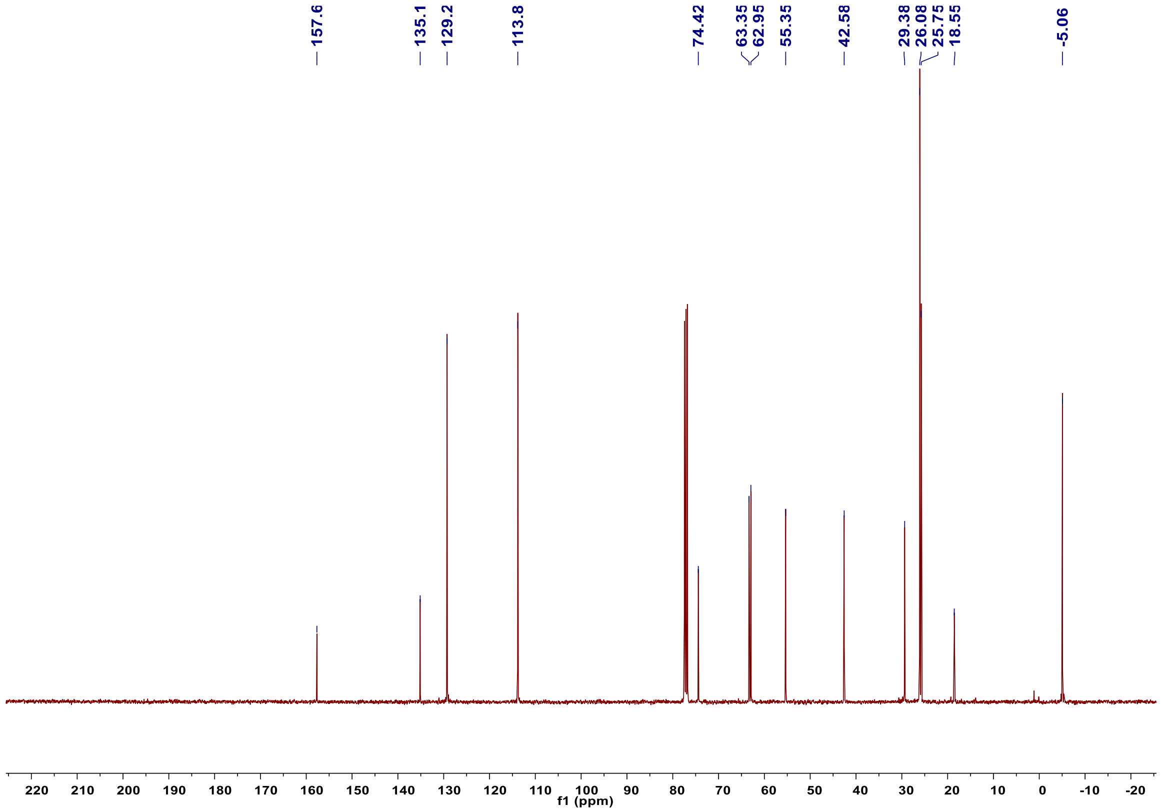

Supplementary Figure 145. ^{13}C NMR (100 MHz, $CDCl_3$) spectrum for 52

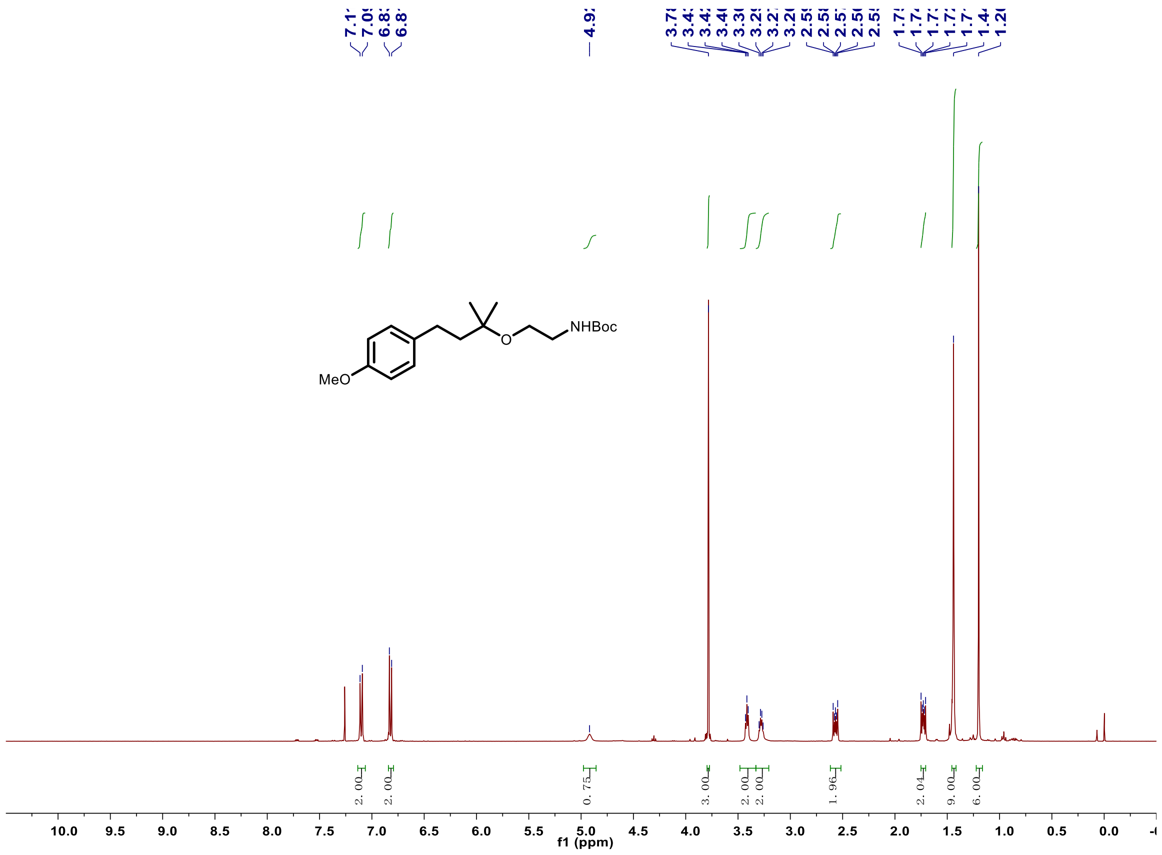

Supplementary Figure 146. ^{1}H NMR (400 MHz, CDCl$_3$) spectrum for 53

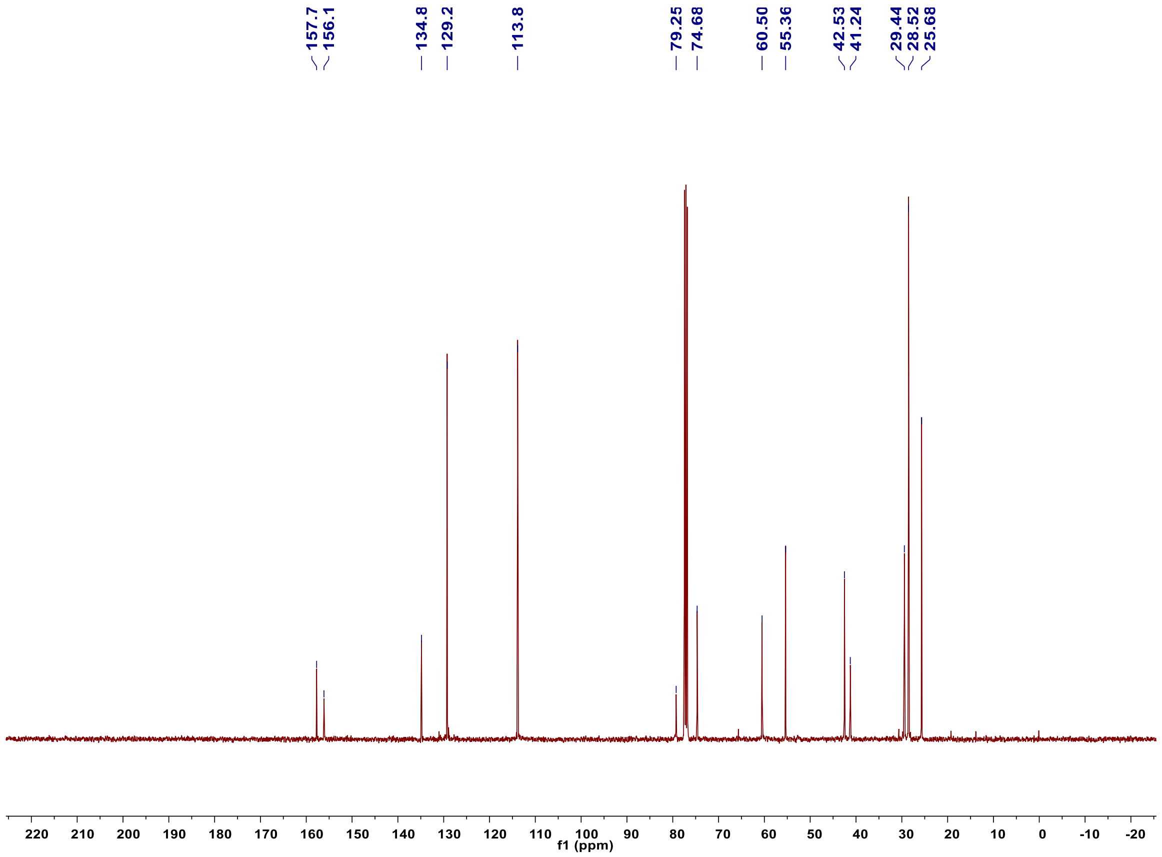

Supplementary Figure 147. ^{13}C NMR (100 MHz, CDCl$_3$) spectrum for 53

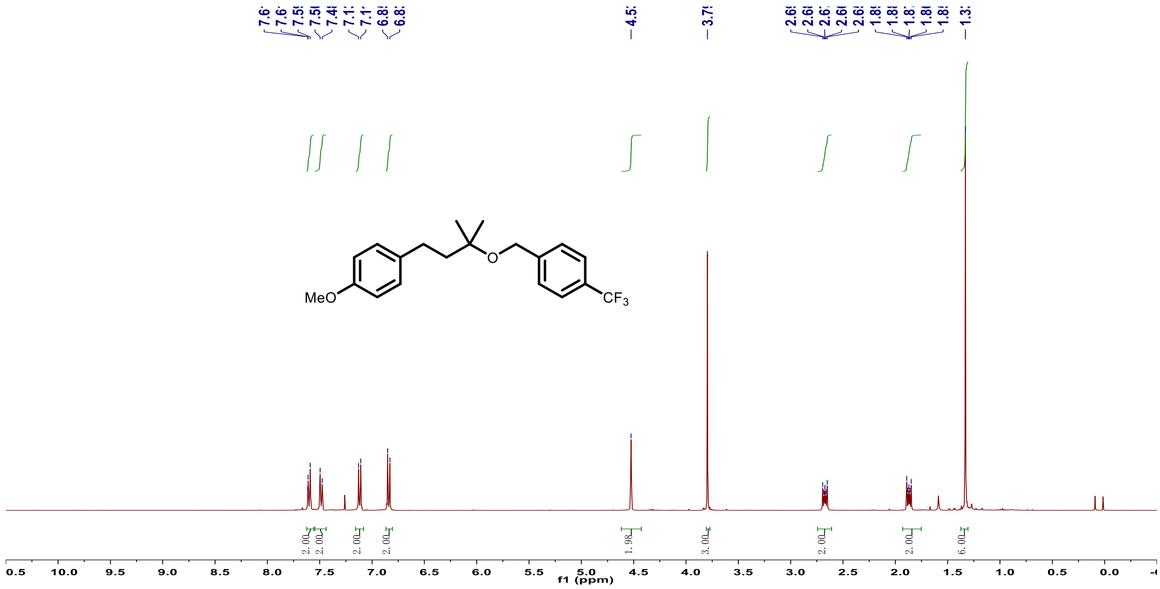

Supplementary Figure 148. ^{1}H NMR (400 MHz, $CDCl_3$) spectrum for 54

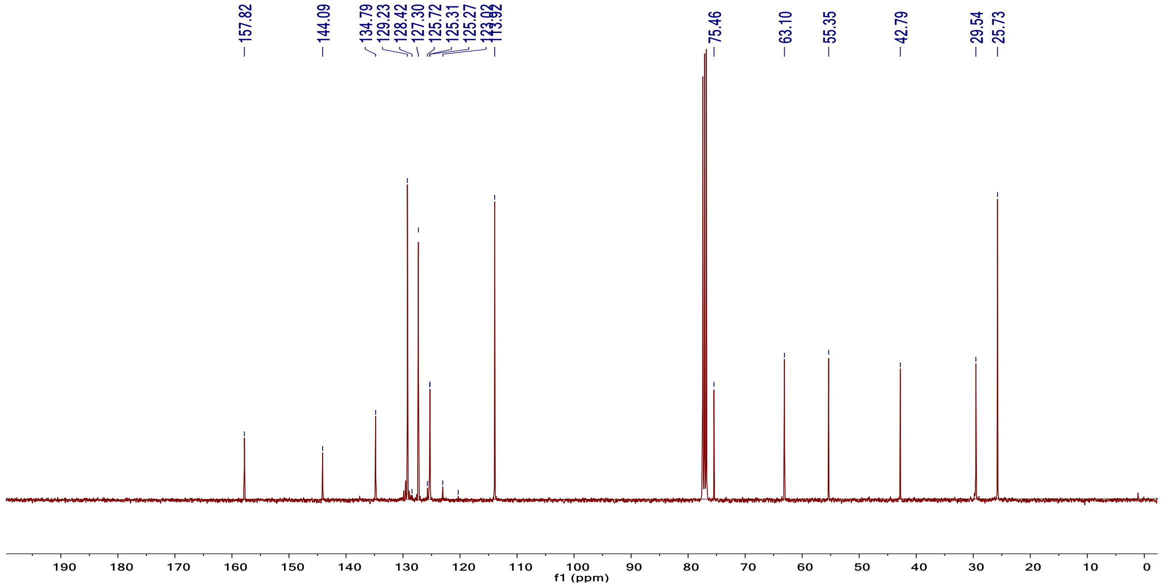

Supplementary Figure 149. ^{13}C NMR (100 MHz, $CDCl_3$) spectrum for 54

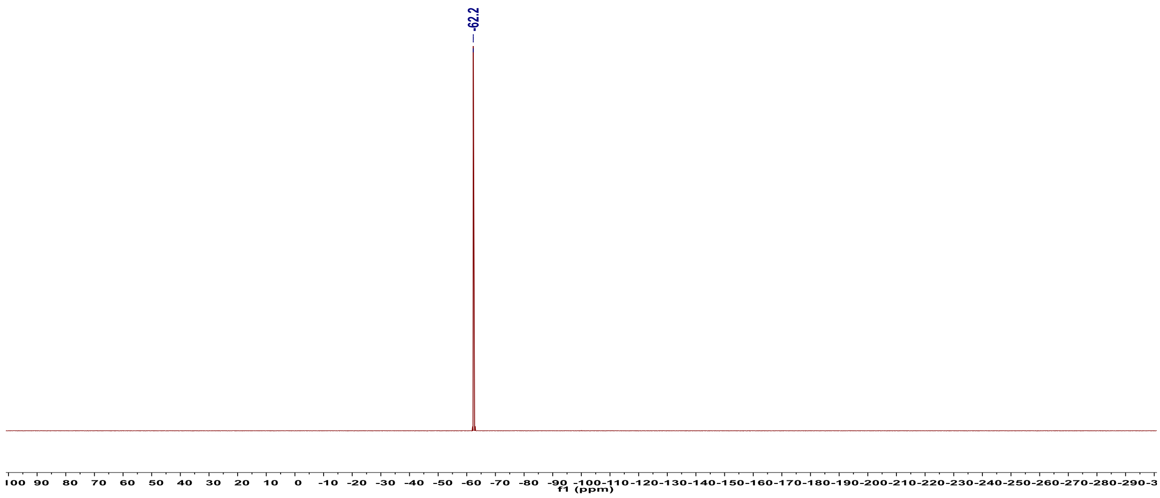

Supplementary Figure 150. ^{19}F NMR (377 MHz, $CDCl_3$) spectrum for 54

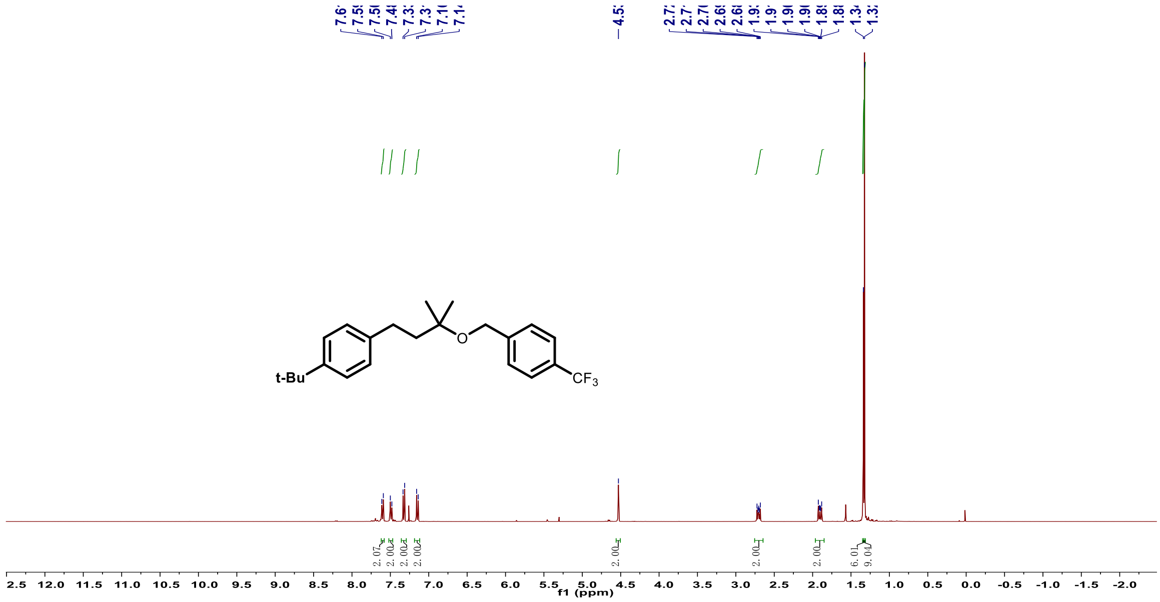

Supplementary Figure 151. ^{1}H NMR (400 MHz, $CDCl_3$) spectrum for 55

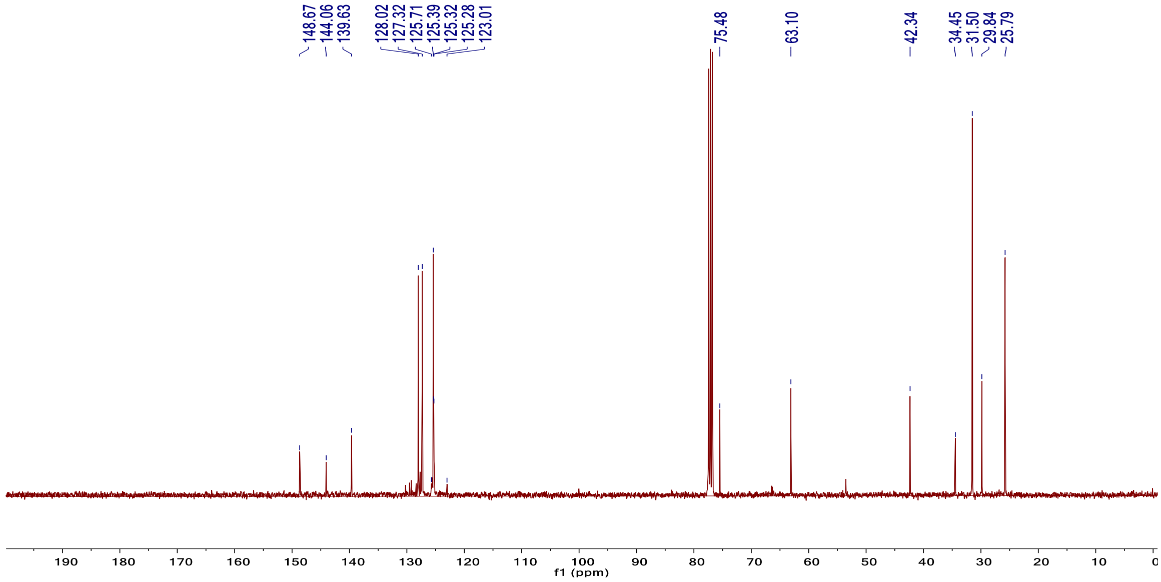

Supplementary Figure 152. ^{13}C NMR (100 MHz, $CDCl_3$) spectrum for 55

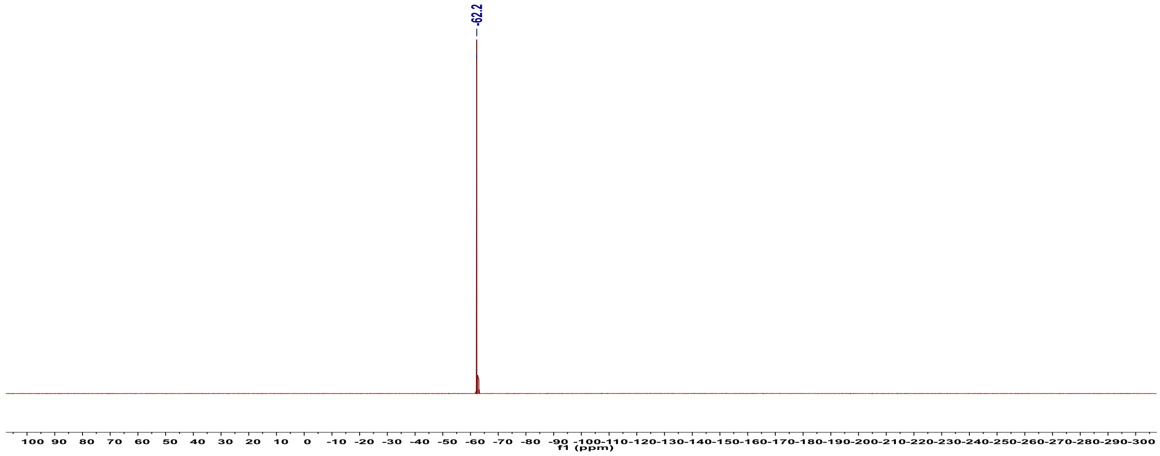

Supplementary Figure 153. ^{19}F NMR (377 MHz, $CDCl_3$) spectrum for 55

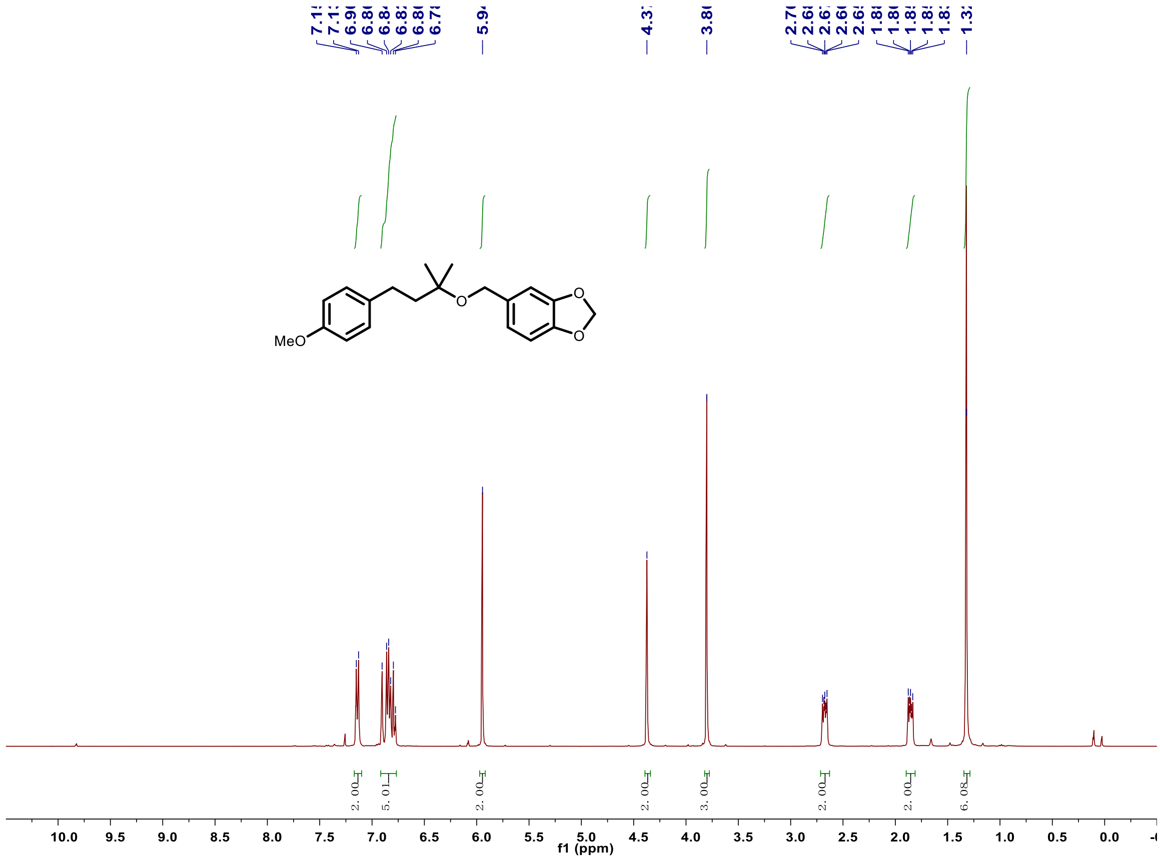

Supplementary Figure 154. ^{1}H NMR (400 MHz, $CDCl_3$) spectrum for 56

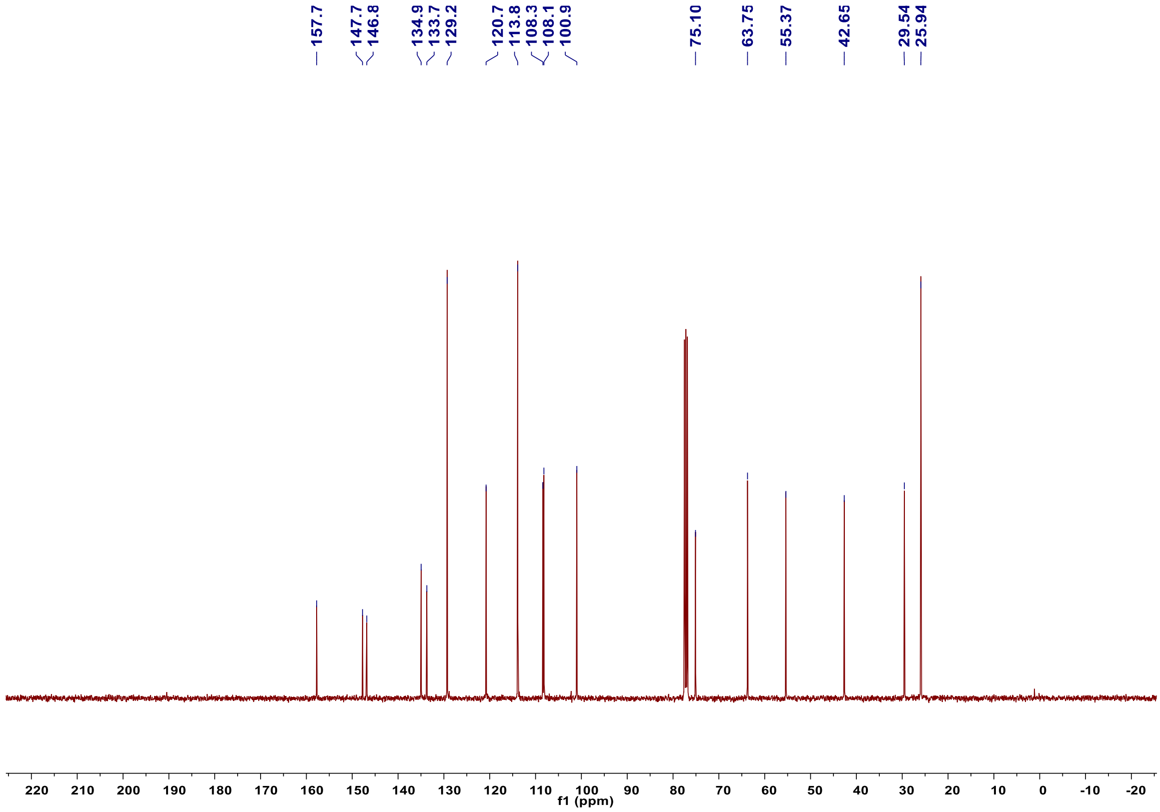

Supplementary Figure 155. ^{13}C NMR (100 MHz, $CDCl_3$) spectrum for 56

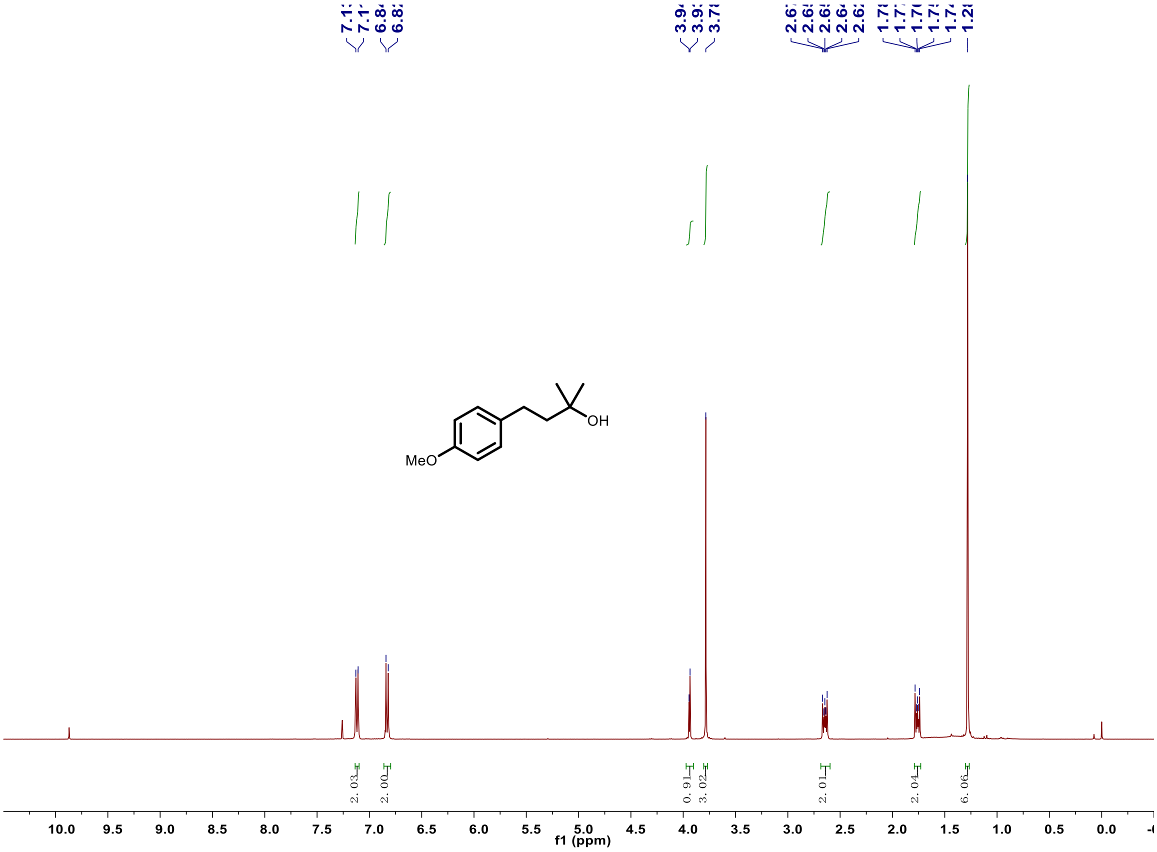

Supplementary Figure 156. ^{1}H **NMR (400 MHz,** $CDCl_3$**) spectrum for 57**

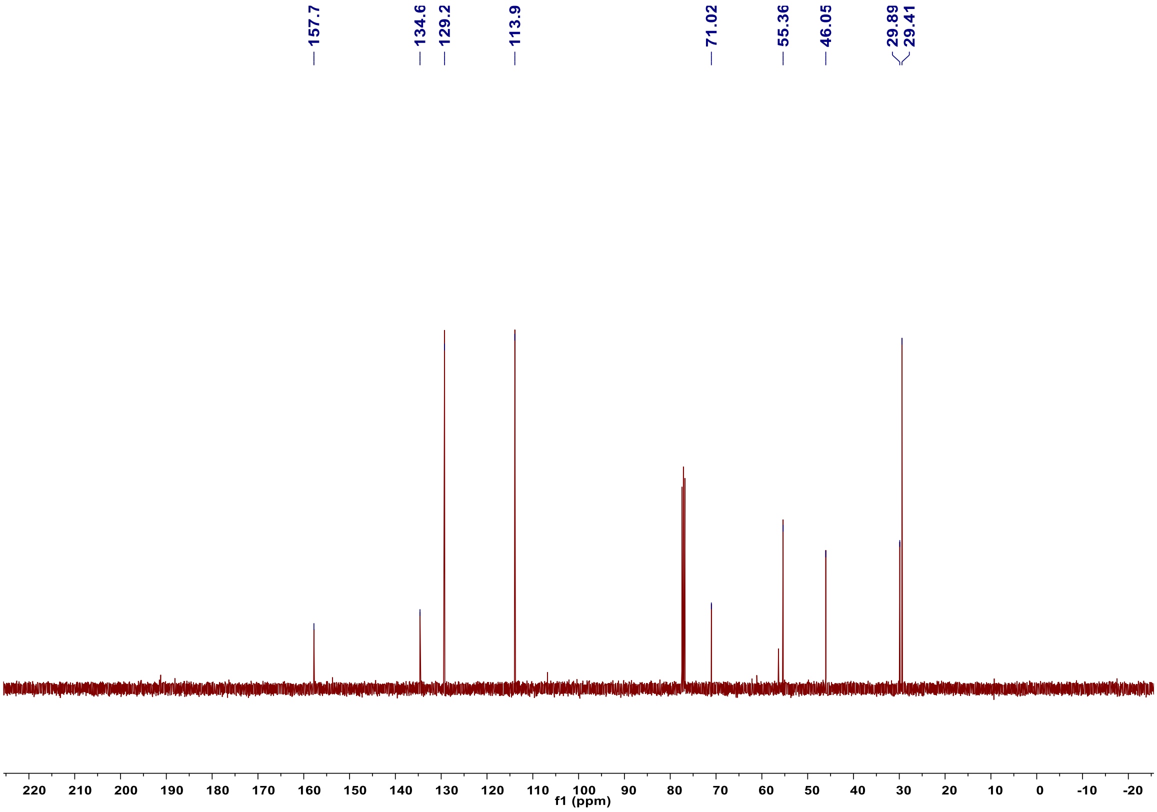

Supplementary Figure 157. ^{13}C **NMR (100 MHz,** $CDCl_3$**) spectrum for 57**

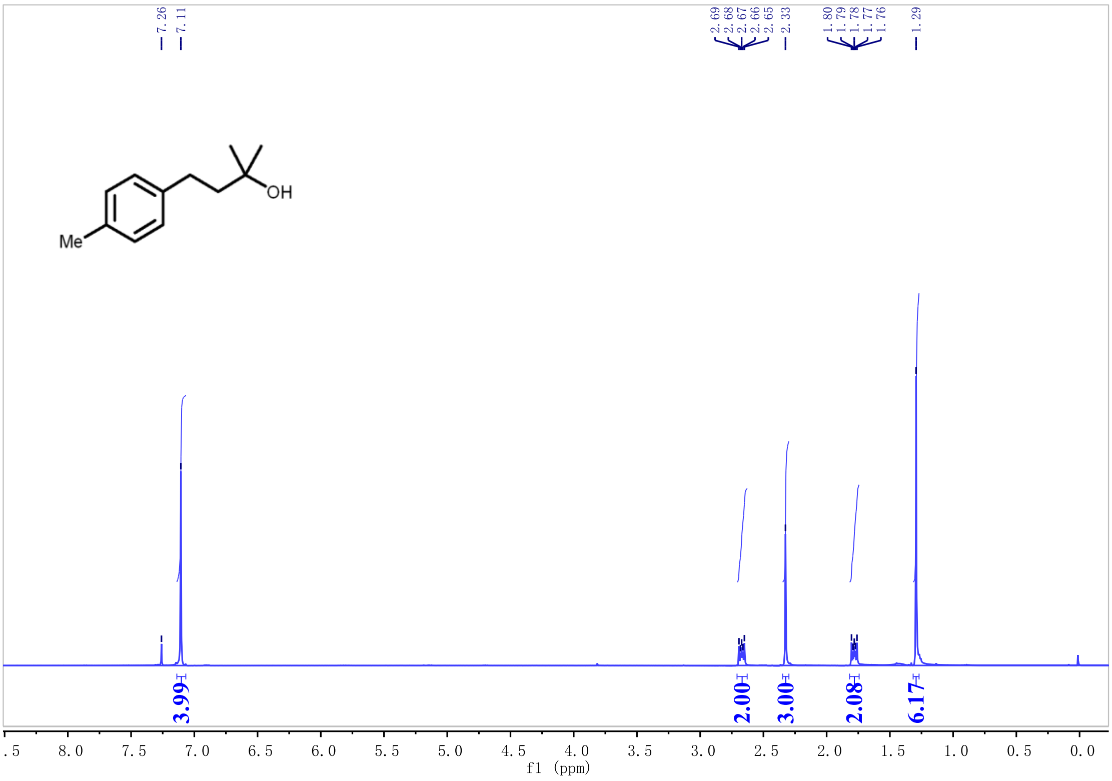

Supplementary Figure 158. ^{1}H NMR (400 MHz, $CDCl_3$) spectrum for 58

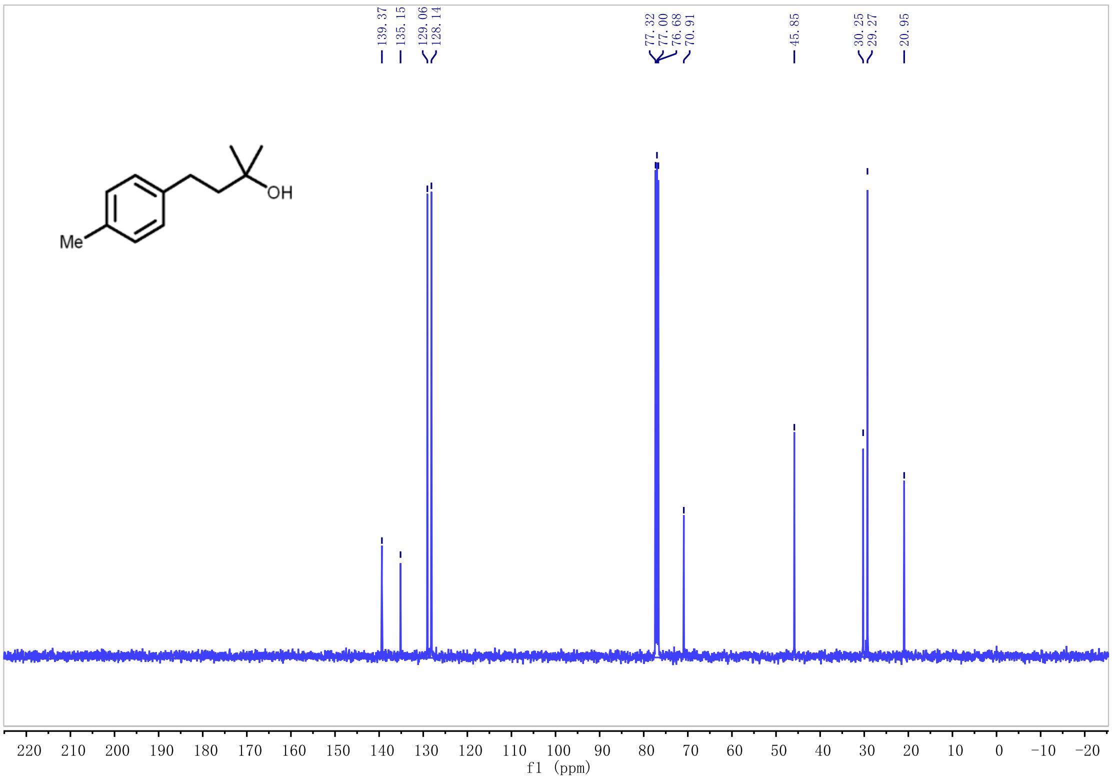

Supplementary Figure 159. ^{13}C NMR (100 MHz, $CDCl_3$) spectrum for 58

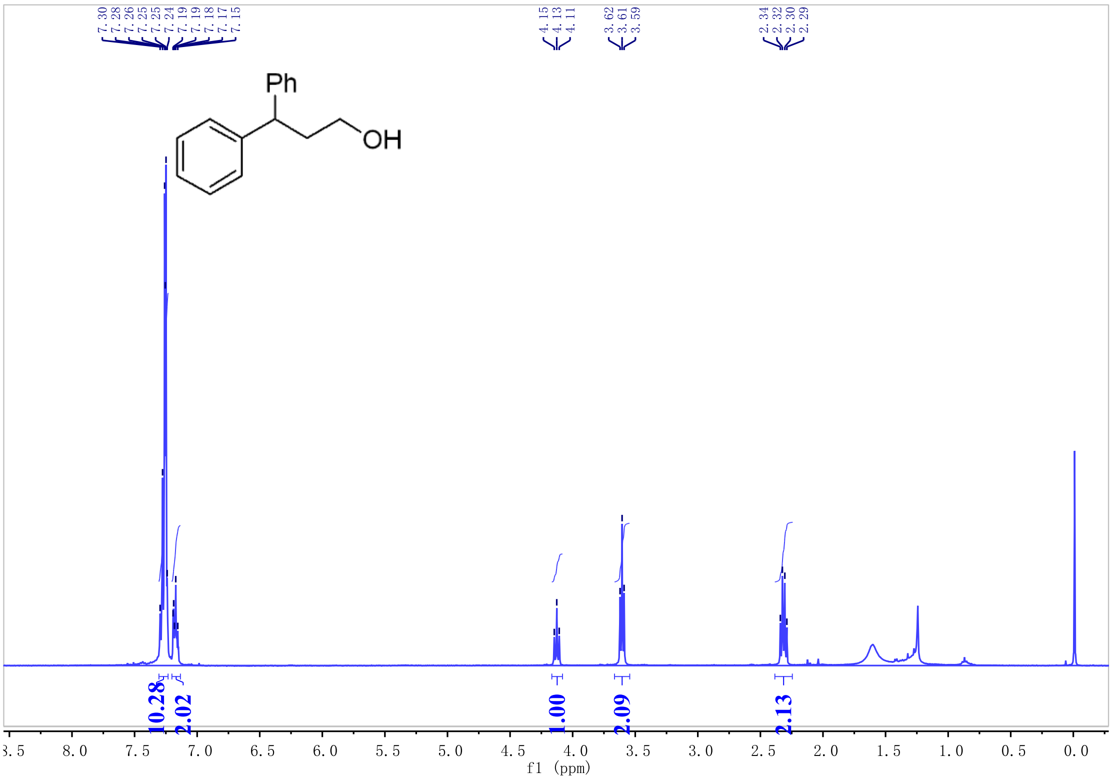

Supplementary Figure 160. ^{1}H NMR (400 MHz, $CDCl_3$) spectrum for 59

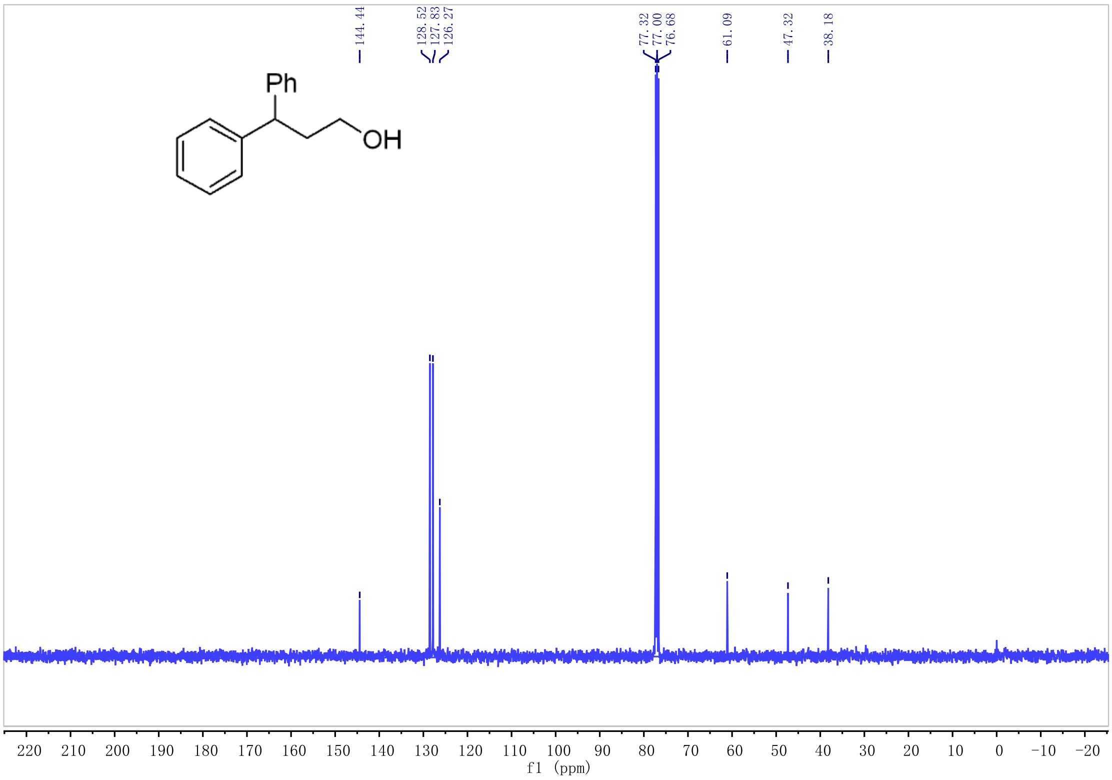

Supplementary Figure 161. ^{13}C NMR (100 MHz, $CDCl_3$) spectrum for 59

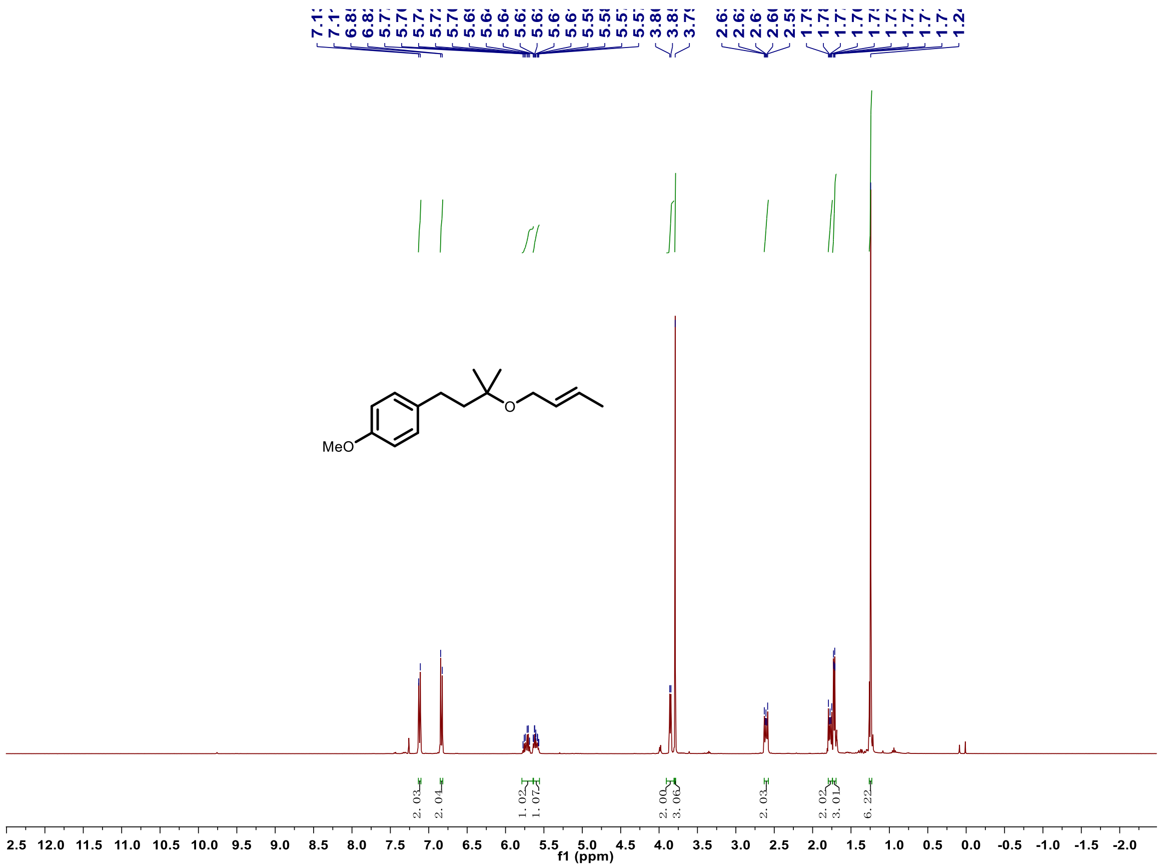

Supplementary Figure 162. ^{1}H **NMR (400 MHz, $CDCl_3$) spectrum for 60**

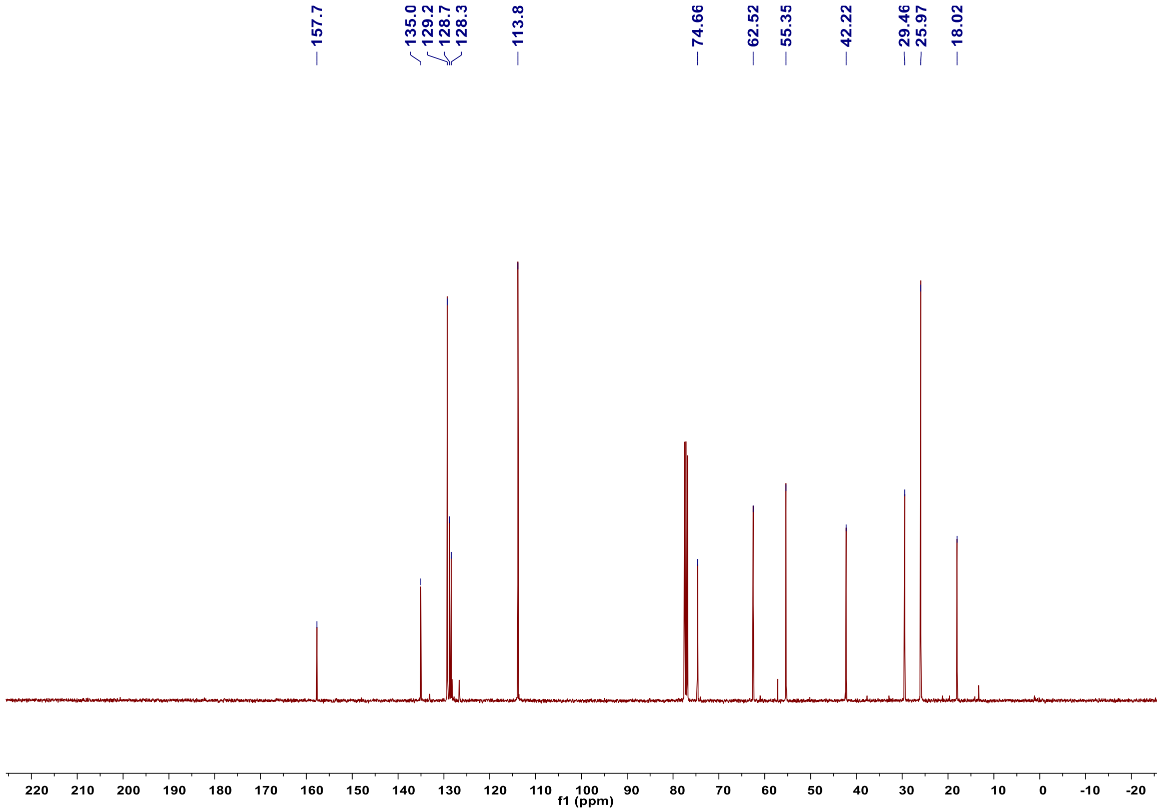

Supplementary Figure 163. ^{13}C **NMR (100 MHz, $CDCl_3$) spectrum for 60**

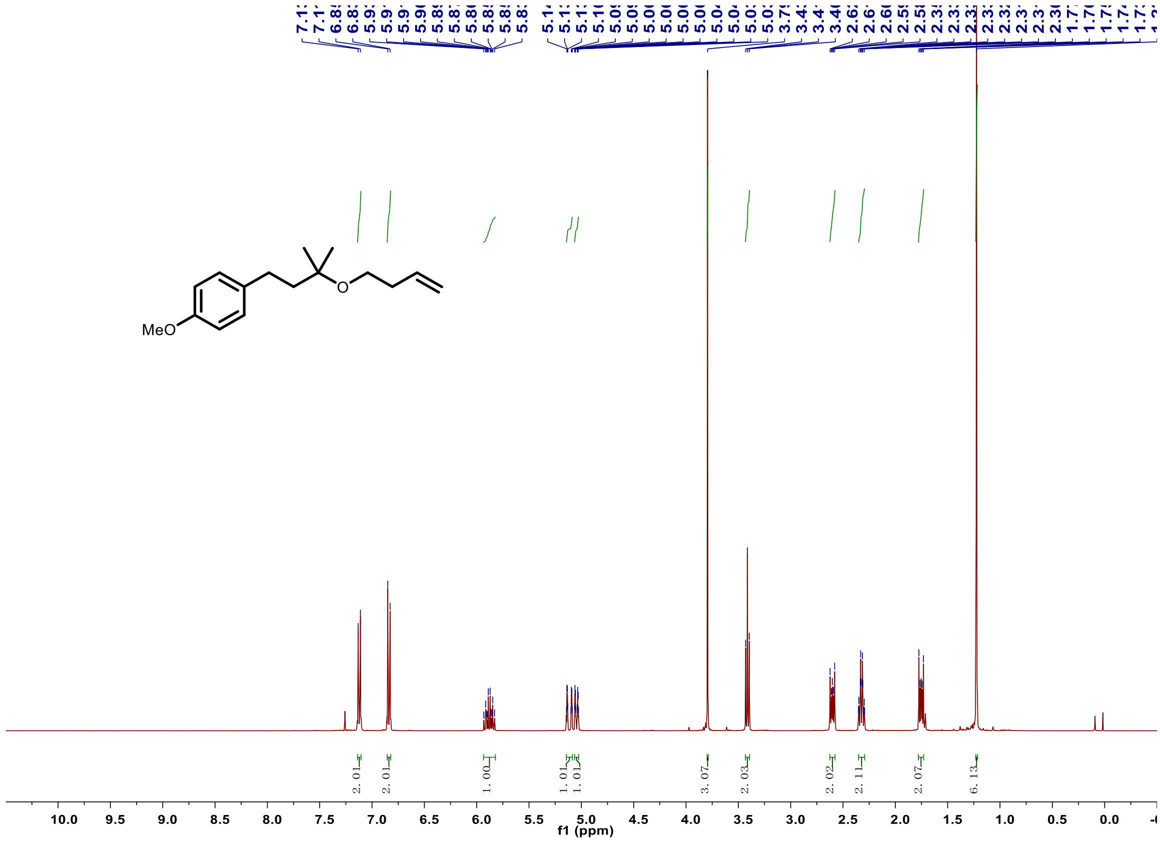

Supplementary Figure 164. ^{1}H NMR (400 MHz, $CDCl_3$) spectrum for 61

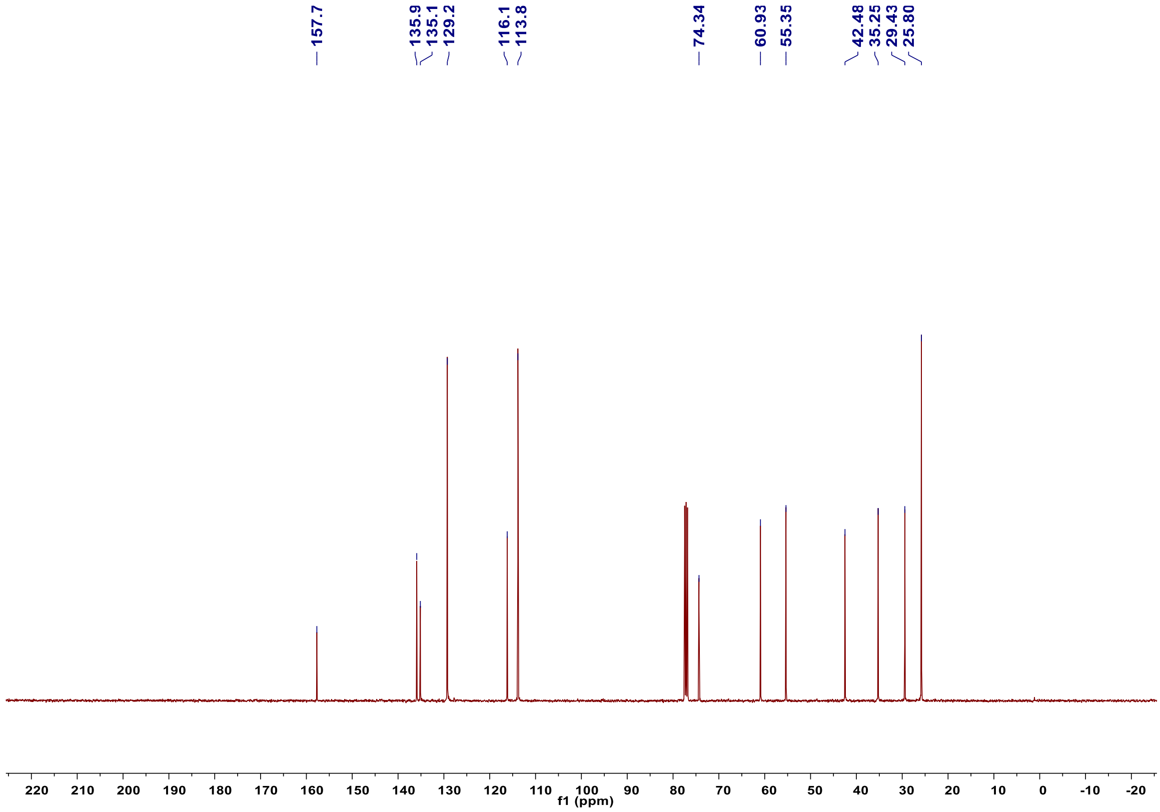

Supplementary Figure 165. ^{13}C NMR (100 MHz, $CDCl_3$) spectrum for 61

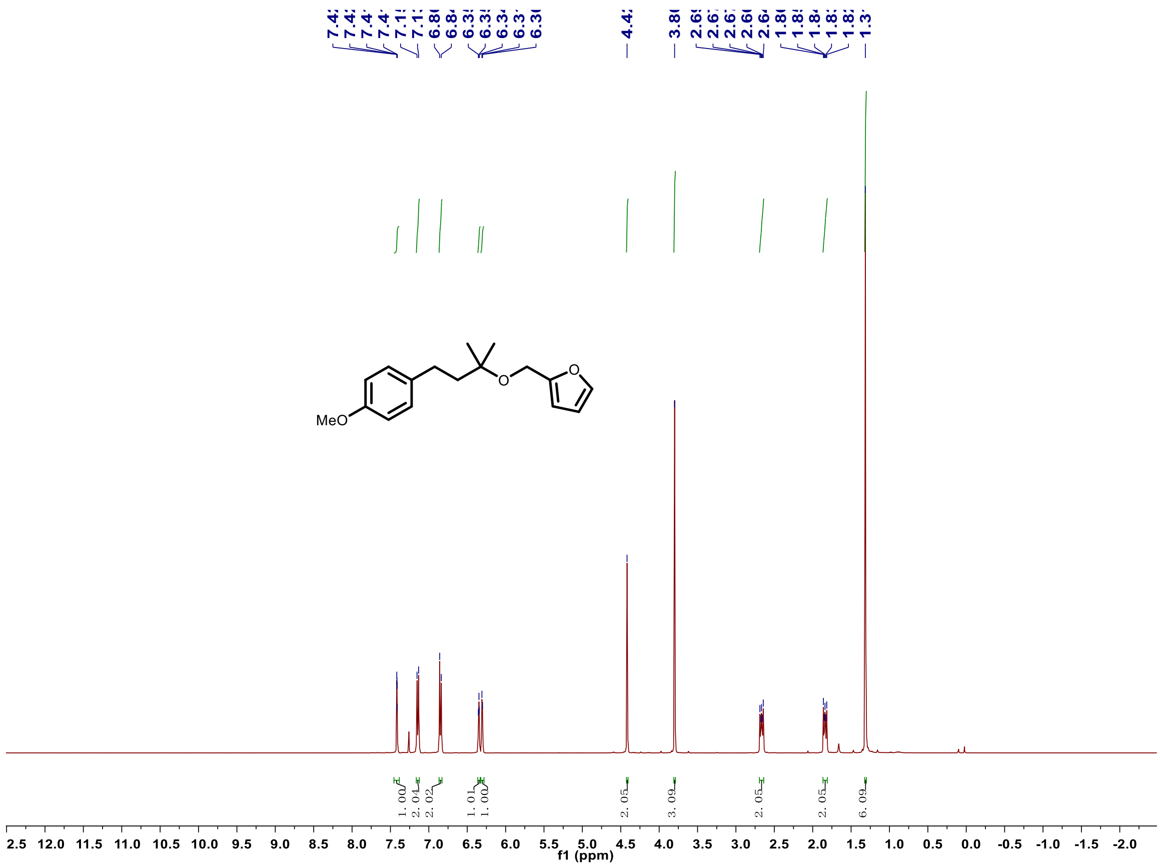

Supplementary Figure 166. ^{1}H **NMR (400 MHz,** $CDCl_3$**) spectrum for 62**

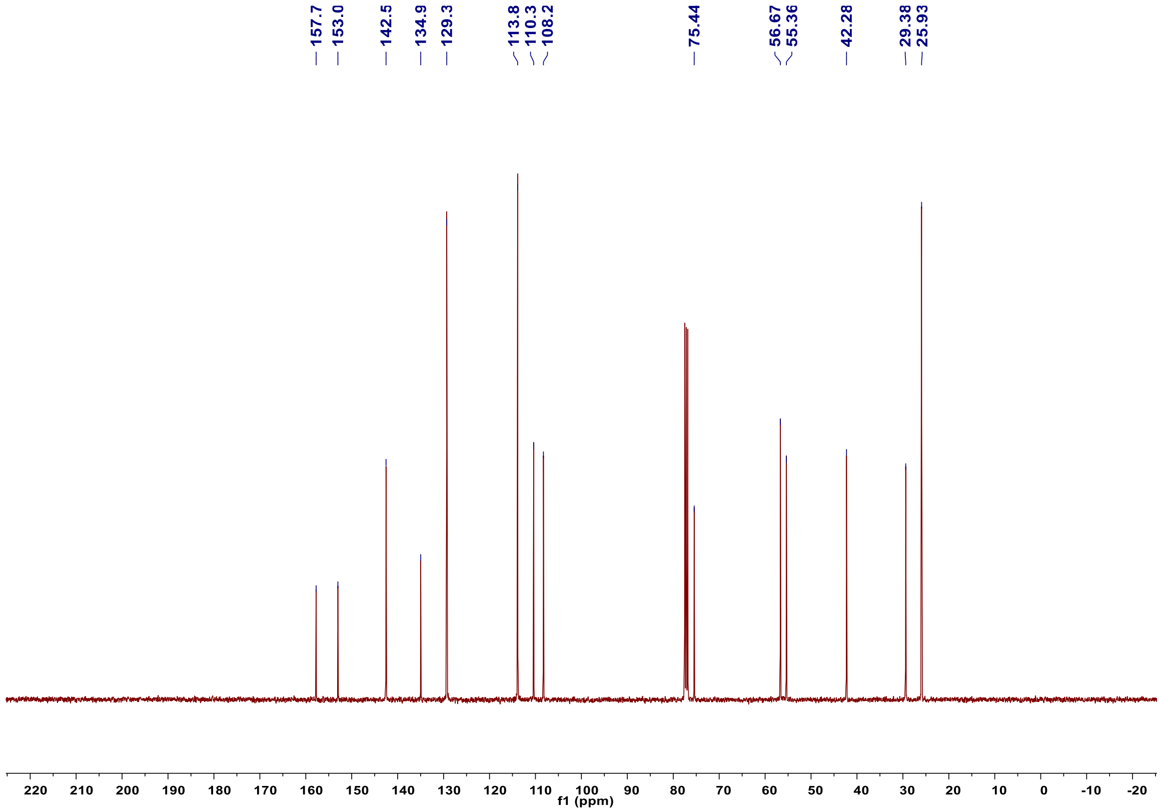

Supplementary Figure 167. ^{13}C **NMR (100 MHz,** $CDCl_3$**) spectrum for 62**

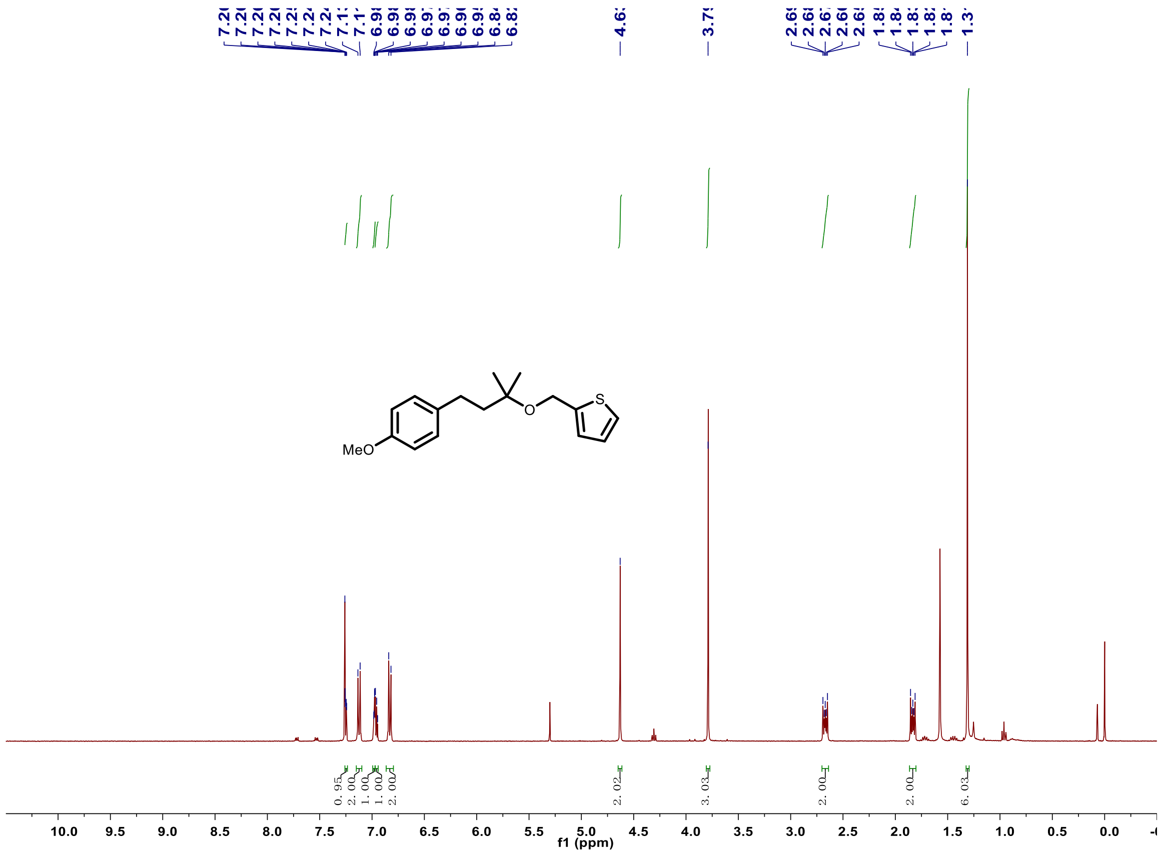

Supplementary Figure 168. ^{1}H NMR (400 MHz, $CDCl_3$) spectrum for 63

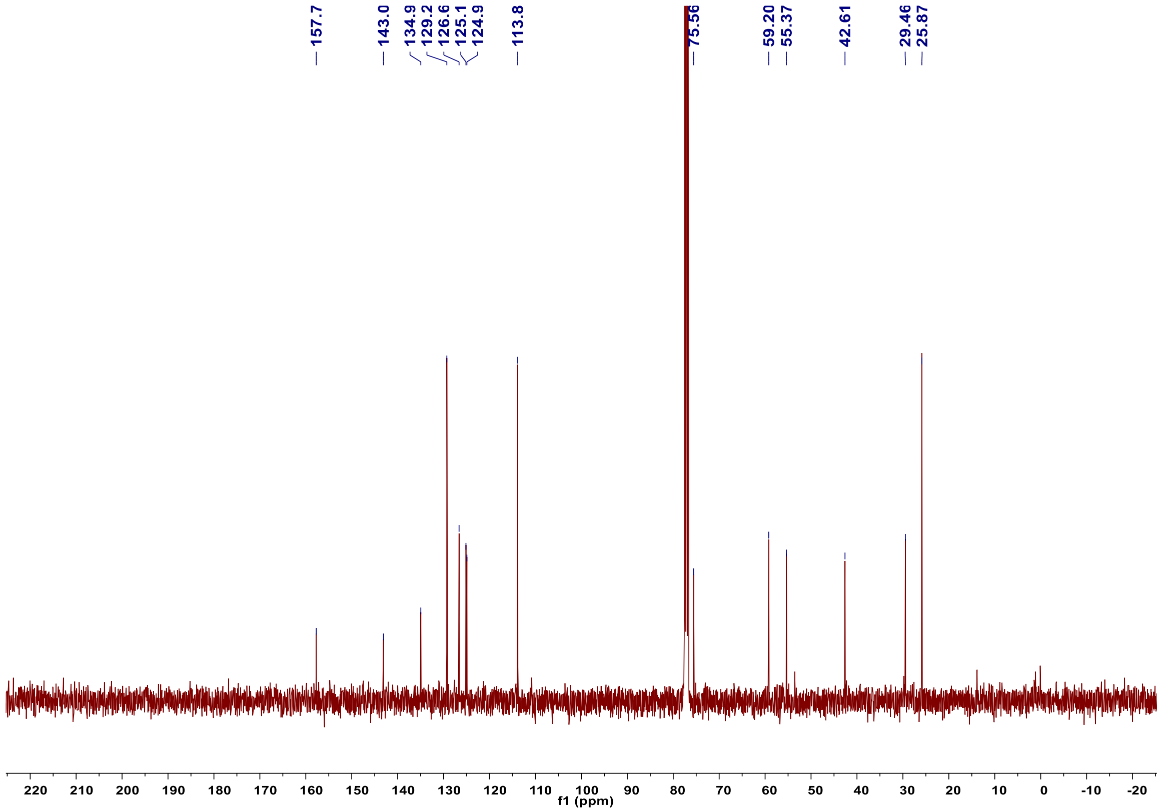

Supplementary Figure 169. ^{13}C NMR (100 MHz, $CDCl_3$) spectrum for 63

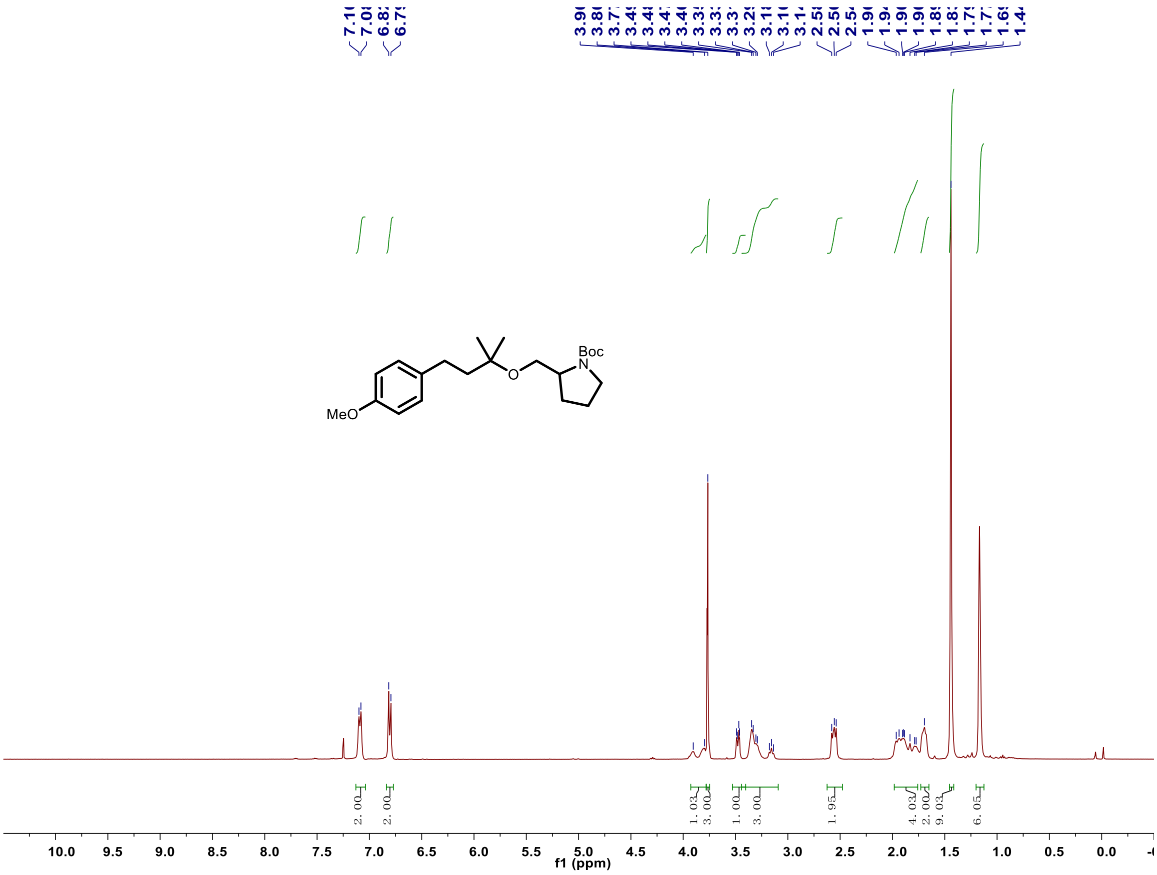

Supplementary Figure 170. ¹H NMR (400 MHz, $CDCl_3$) spectrum for 64

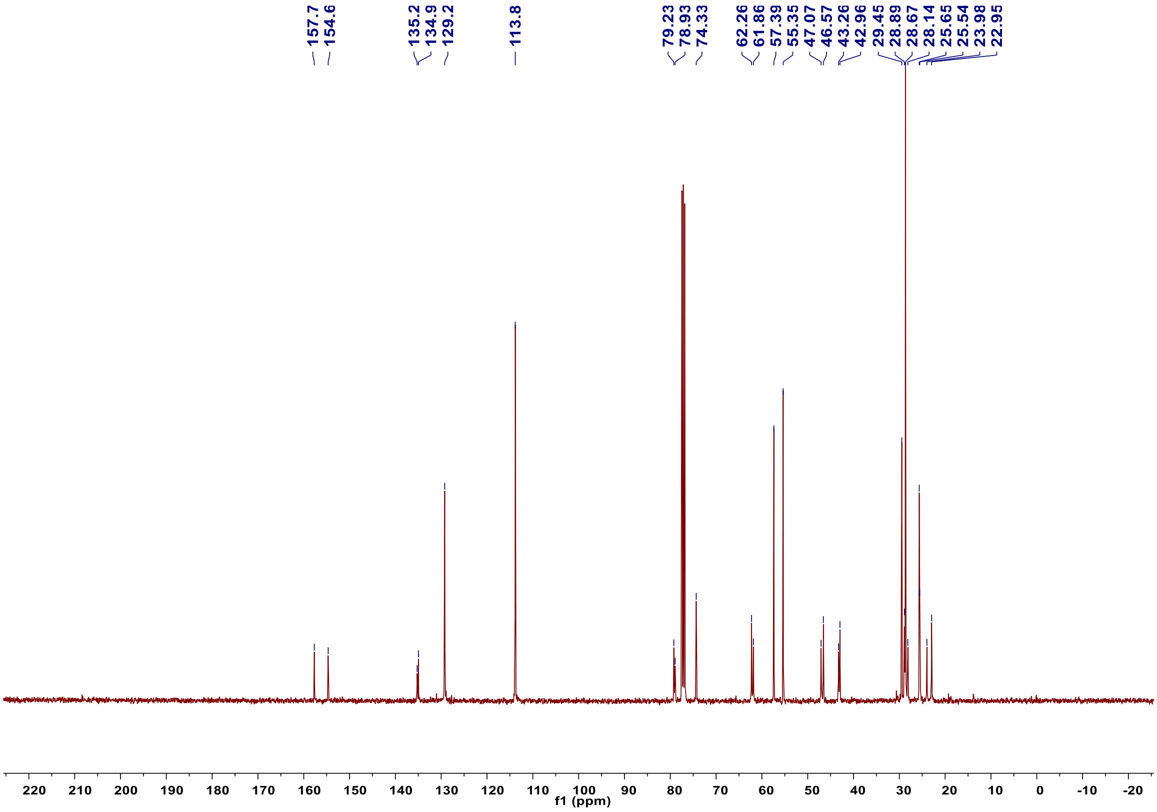

Supplementary Figure 171. ¹³C NMR (100 MHz, $CDCl_3$) spectrum for 64

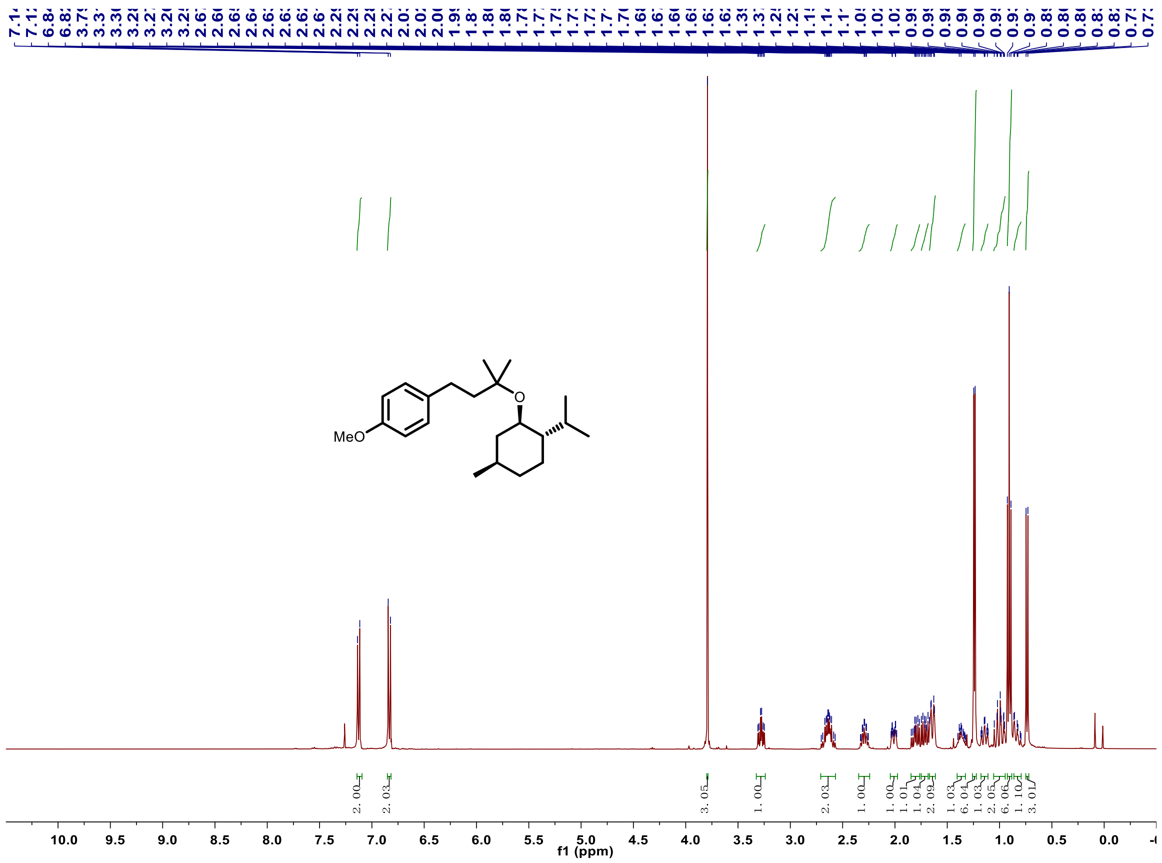

Supplementary Figure 172. ^{1}H NMR (400 MHz, $CDCl_3$) spectrum for 65

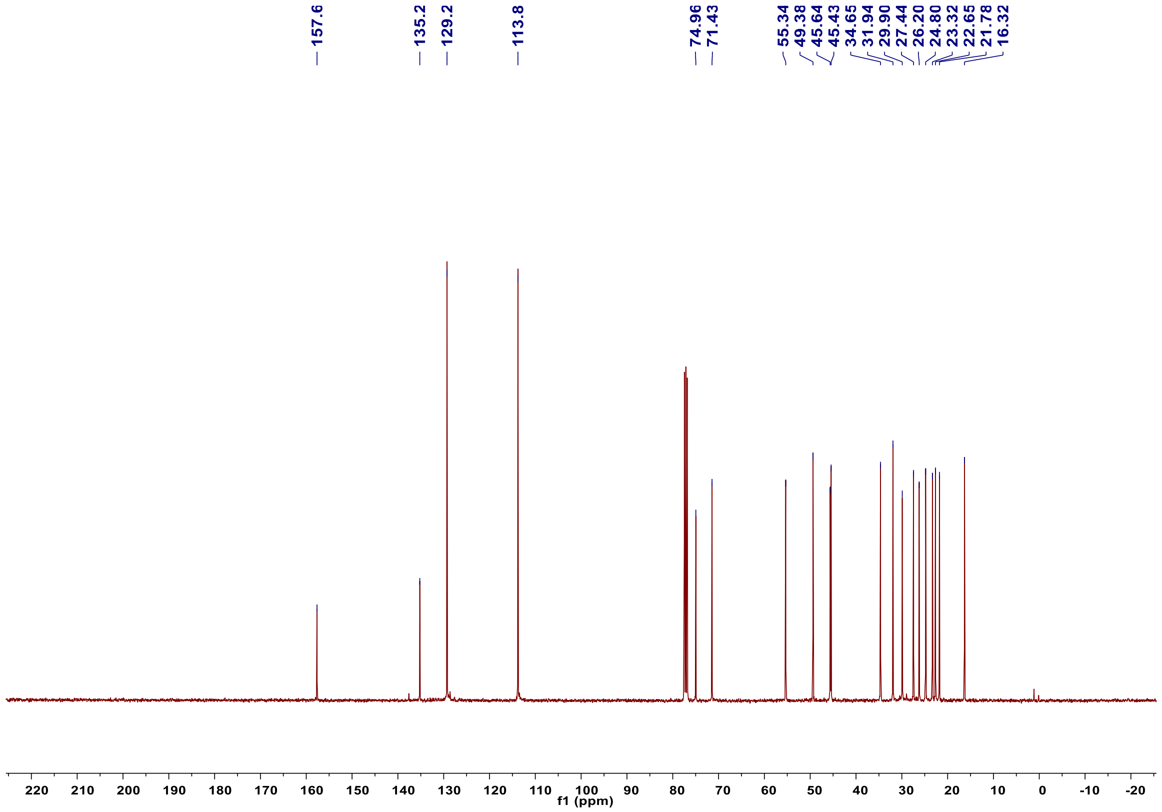

Supplementary Figure 173. ^{13}C NMR (100 MHz, $CDCl_3$) spectrum for 65

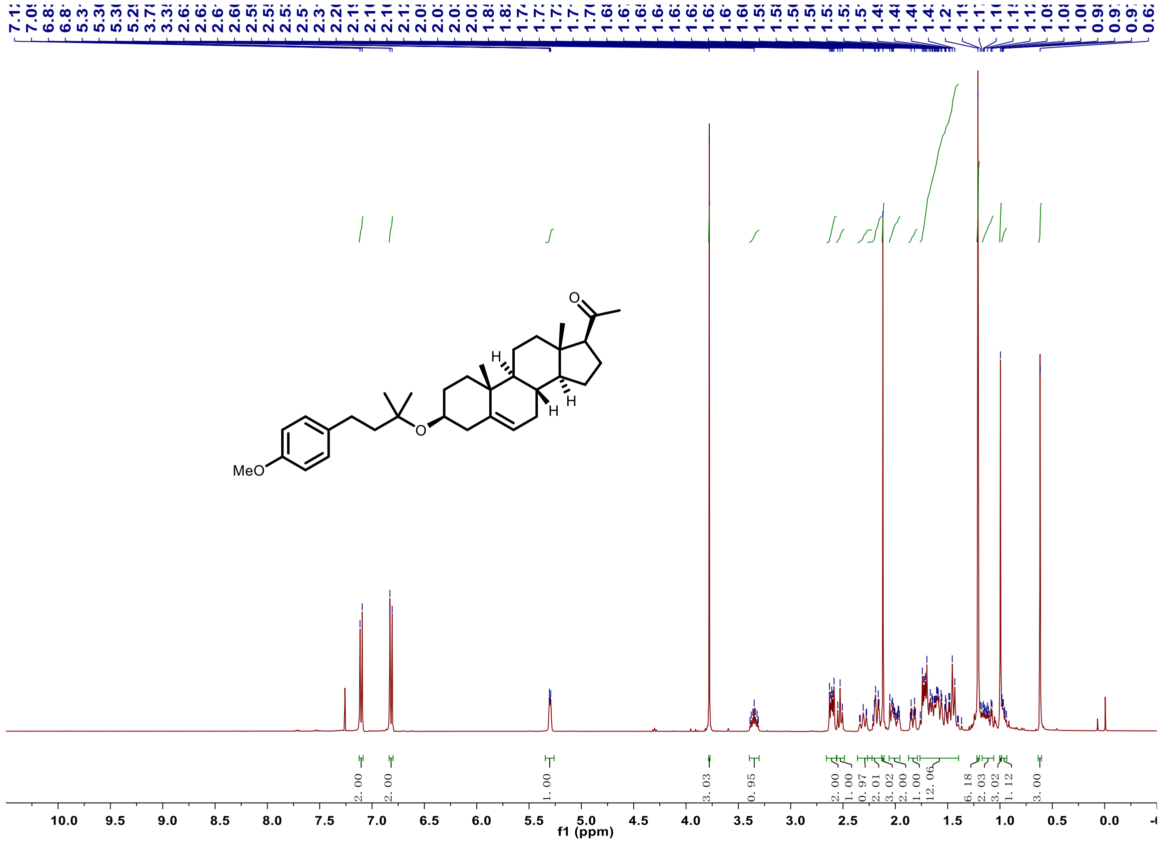

Supplementary Figure 174. ^{1}H NMR (400 MHz, $CDCl_3$) spectrum for 66

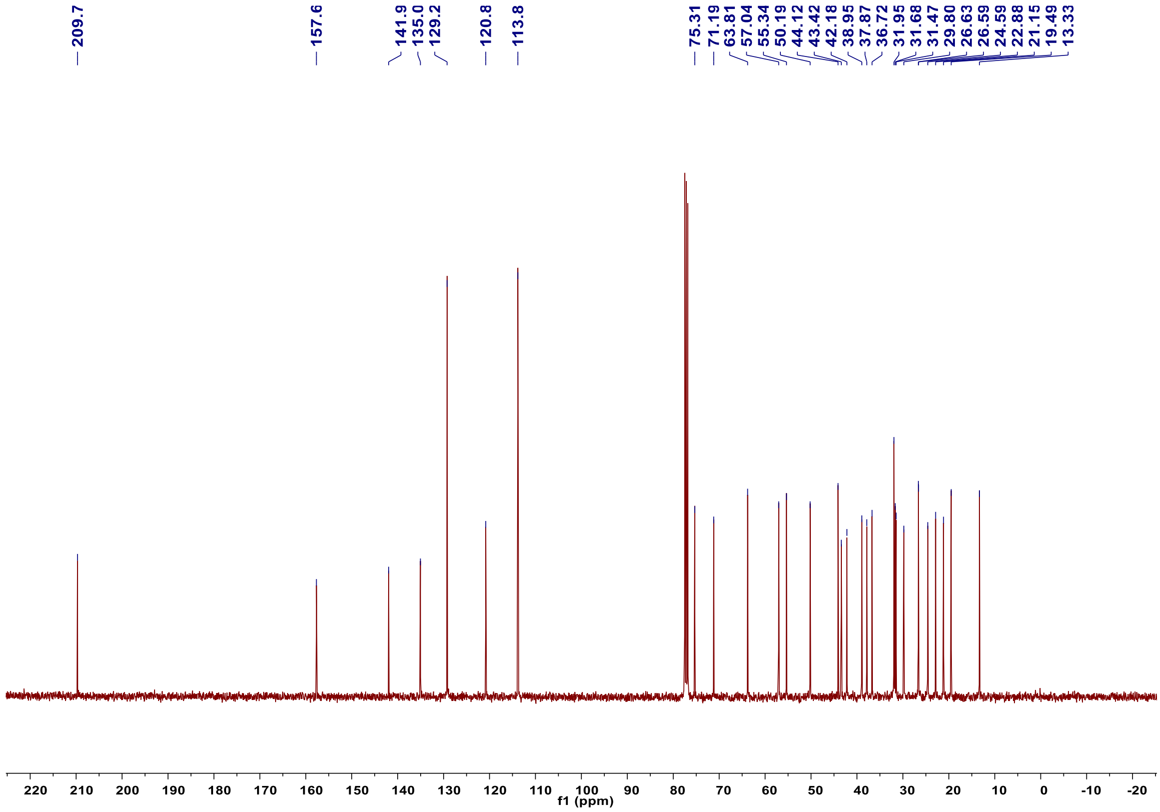

Supplementary Figure 175. ^{13}C NMR (100 MHz, $CDCl_3$) spectrum for 66

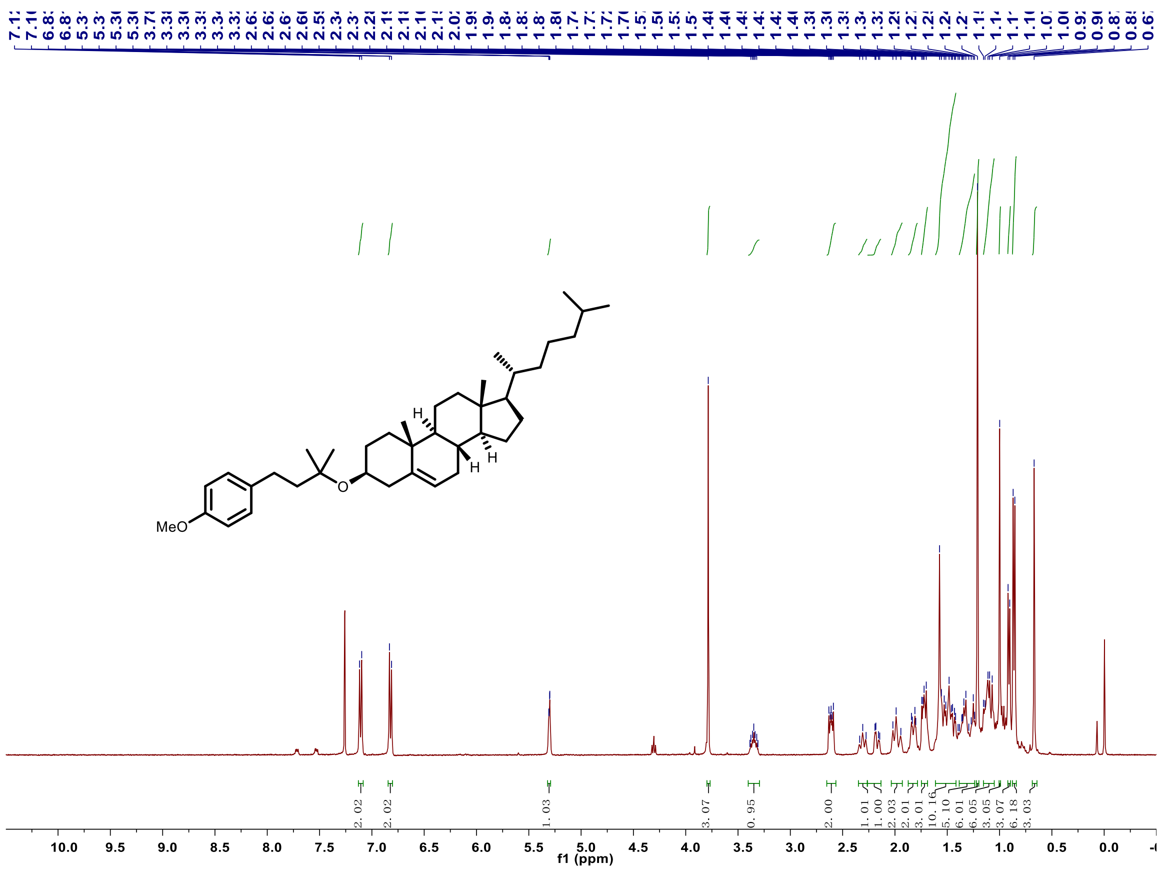

Supplementary Figure 176. ^{1}H NMR (400 MHz, $CDCl_3$) spectrum for 67

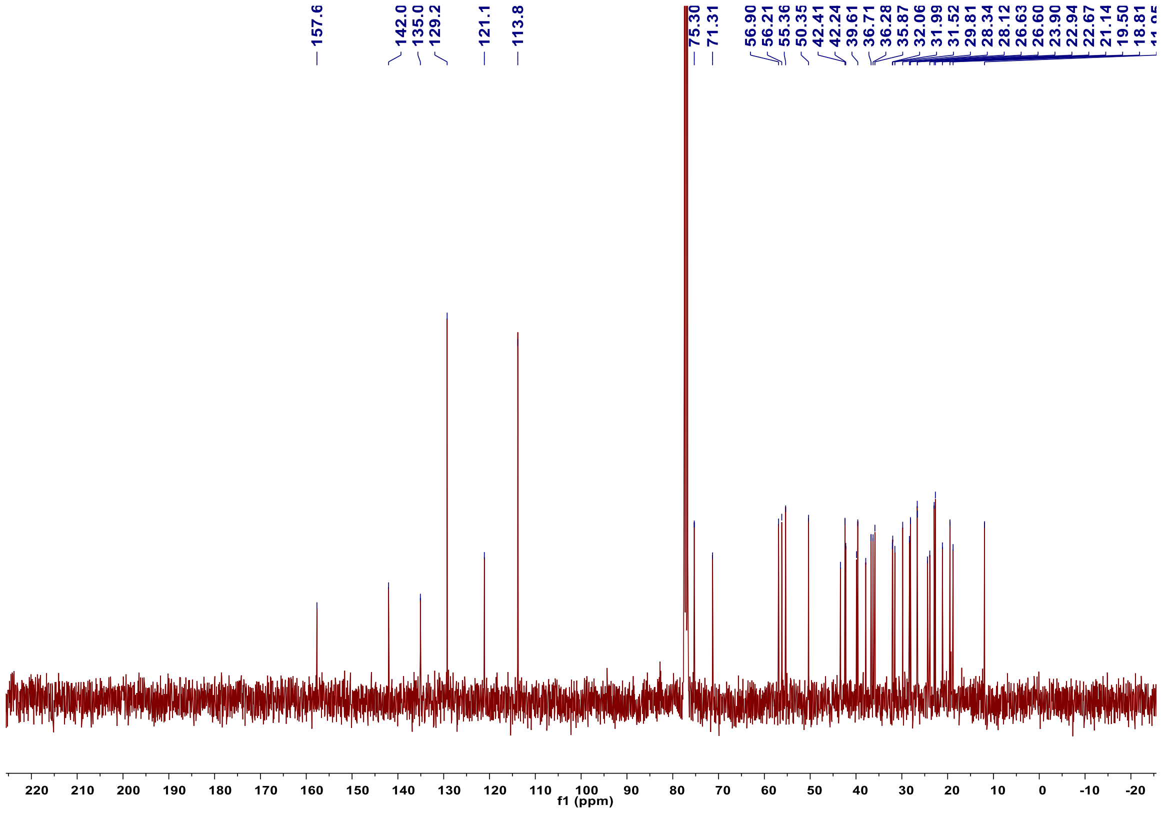

Supplementary Figure 177. ^{13}C NMR (100 MHz, $CDCl_3$) spectrum for 67

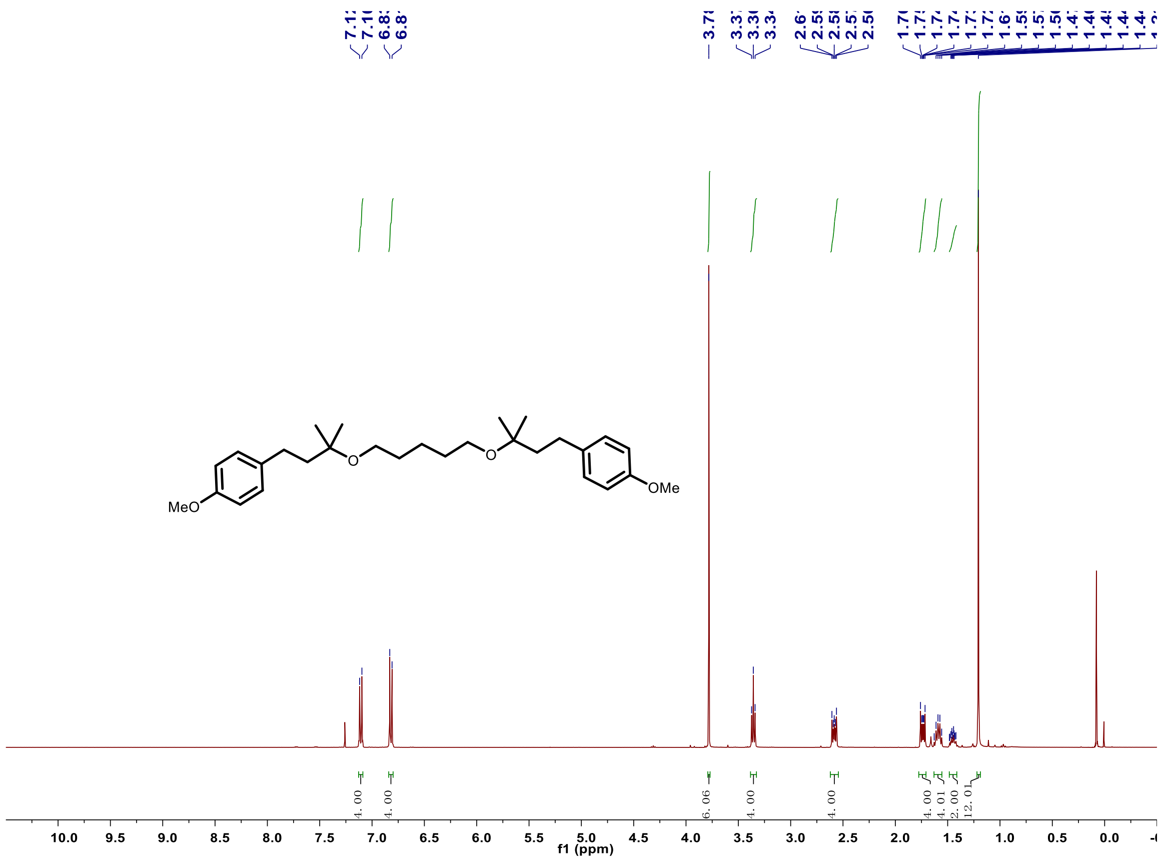

Supplementary Figure 178. ¹H NMR (400 MHz, $CDCl_3$) spectrum for 68

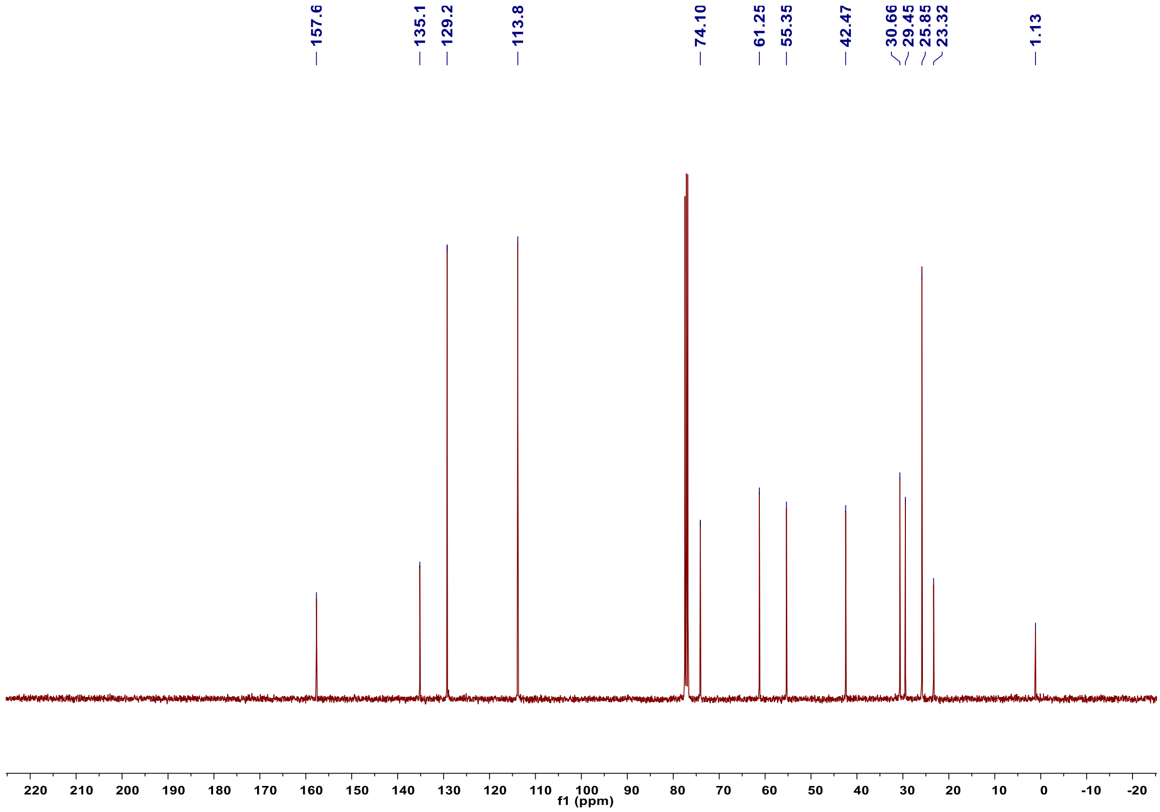

Supplementary Figure 179. ¹³C NMR (100 MHz, $CDCl_3$) spectrum for 68

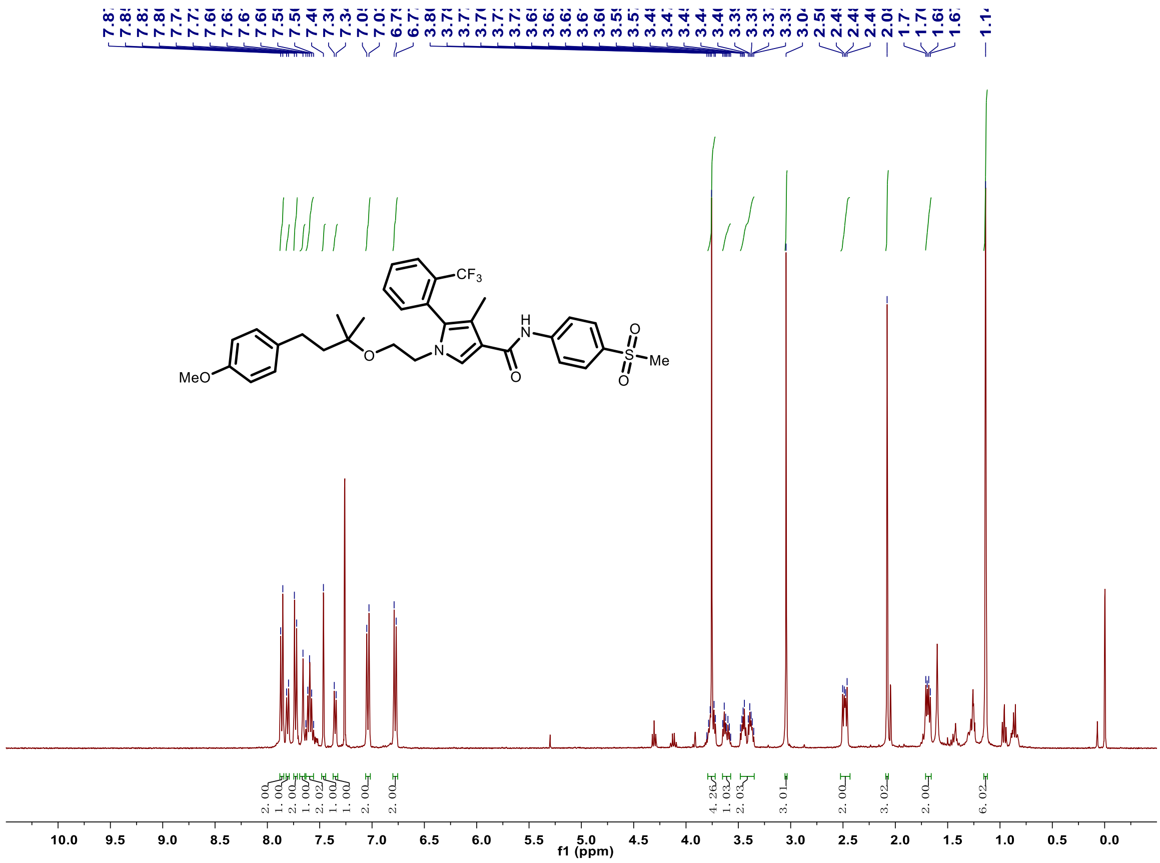

Supplementary Figure 180. ^{1}H NMR (400 MHz, $CDCl_3$) spectrum for 69

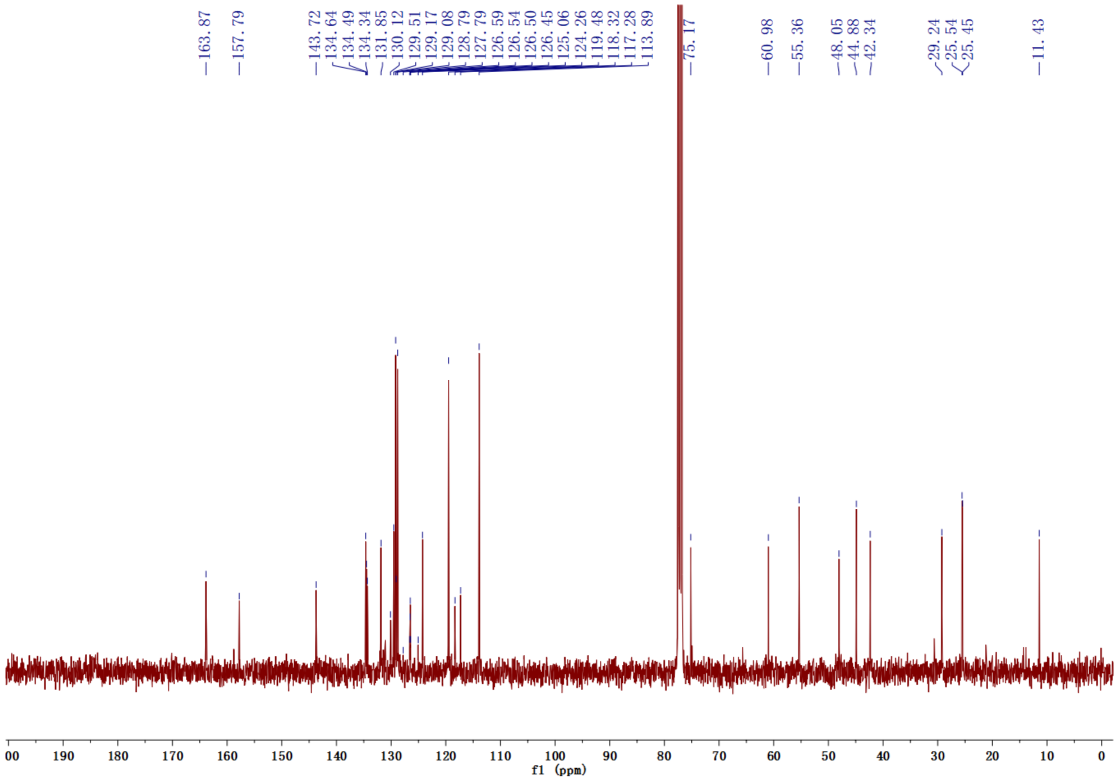

Supplementary Figure 181. ^{13}C NMR (100 MHz, $CDCl_3$) spectrum for 69

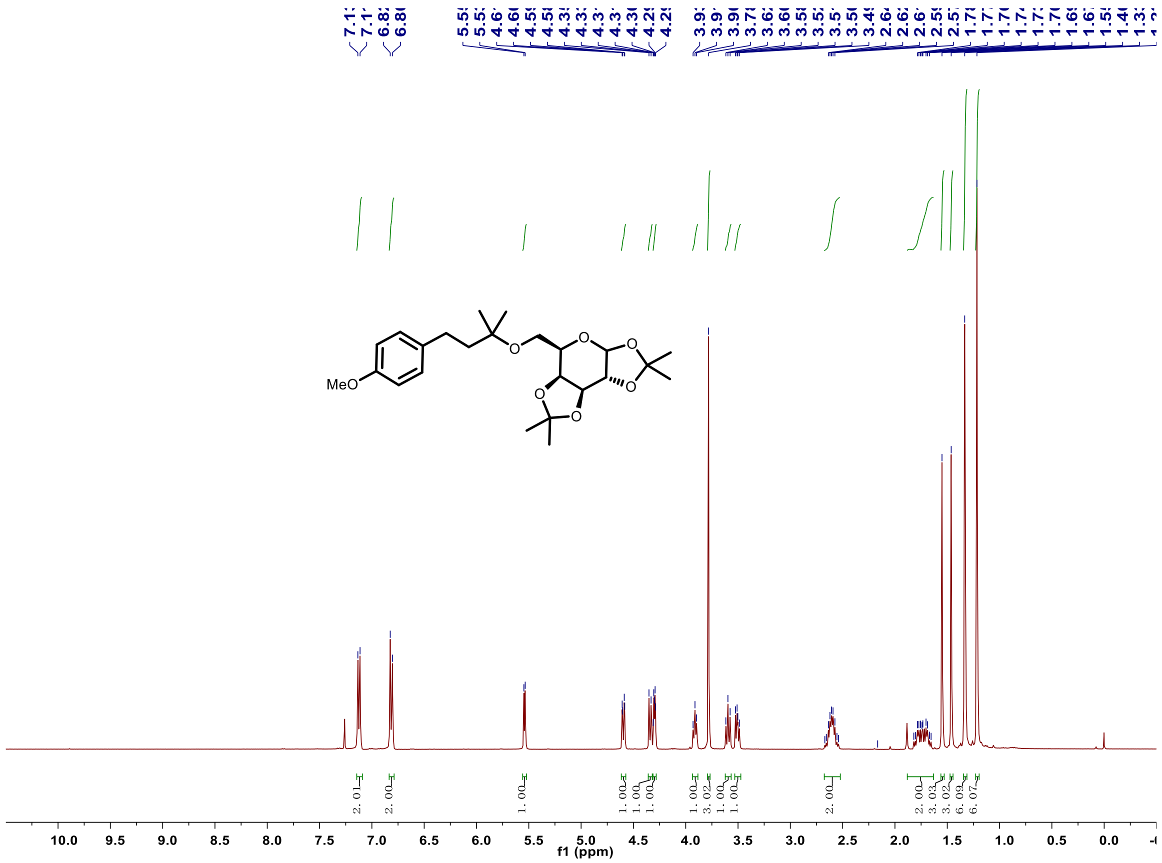

Supplementary Figure 182. ^{1}H NMR (400 MHz, $CDCl_3$) spectrum for 70

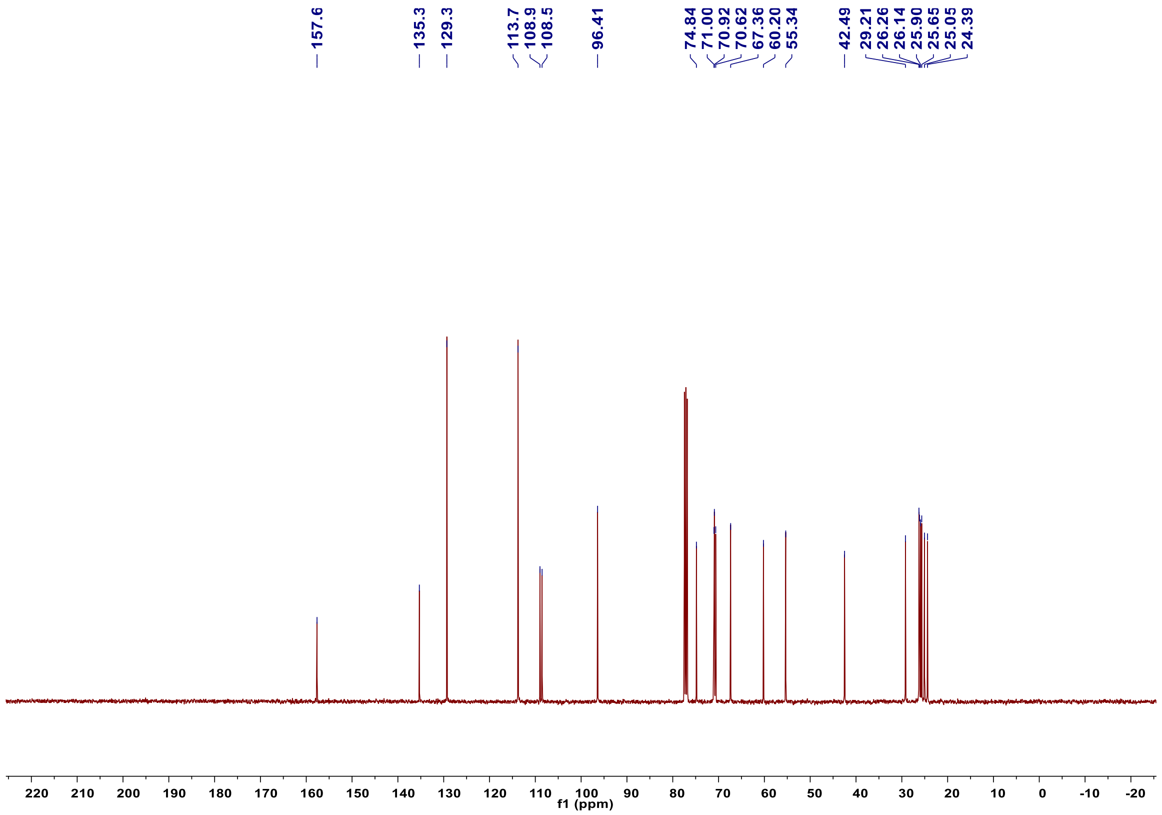

Supplementary Figure 183. ^{13}C NMR (100 MHz, $CDCl_3$) spectrum for 70

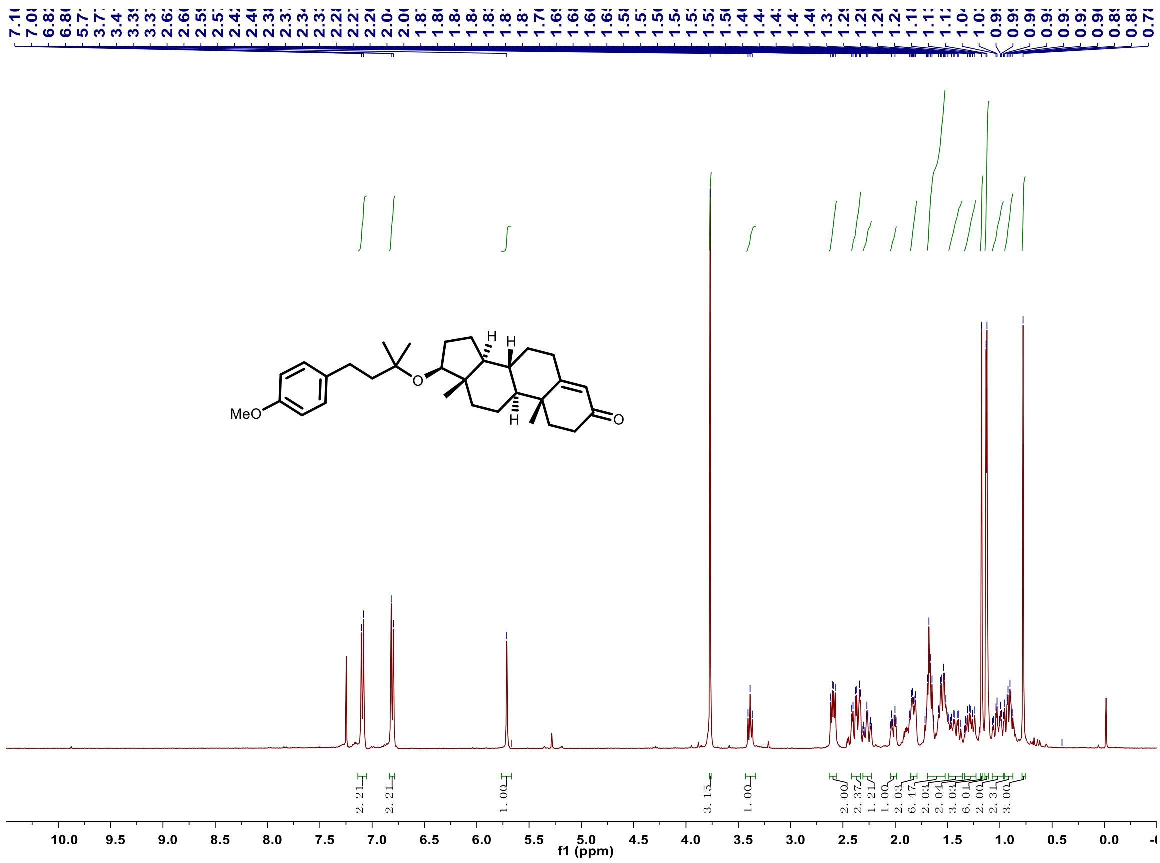

Supplementary Figure 184. ^{1}H NMR (400 MHz, $CDCl_3$) spectrum for 71

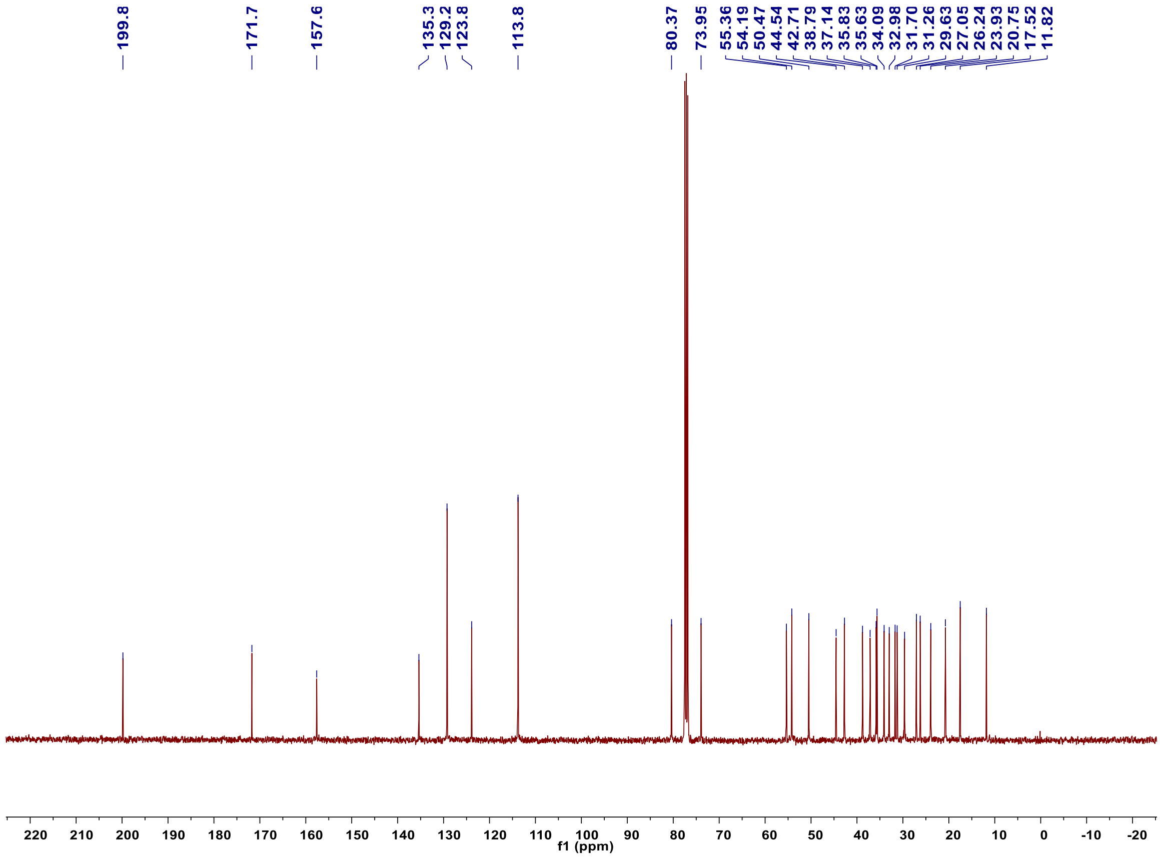

Supplementary Figure 185. ^{13}C NMR (100 MHz, $CDCl_3$) spectrum for 71

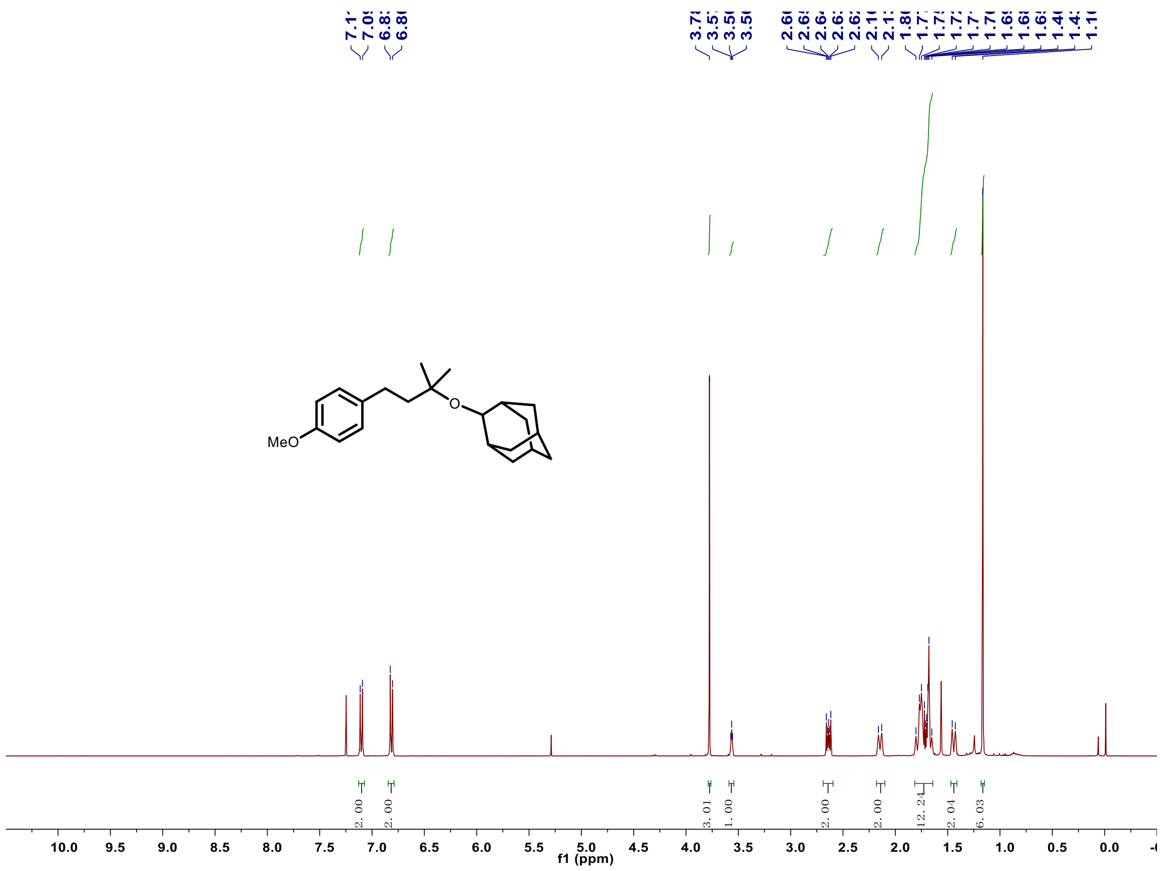

Supplementary Figure 186. ^{1}H NMR (400 MHz, $CDCl_3$) spectrum for 72

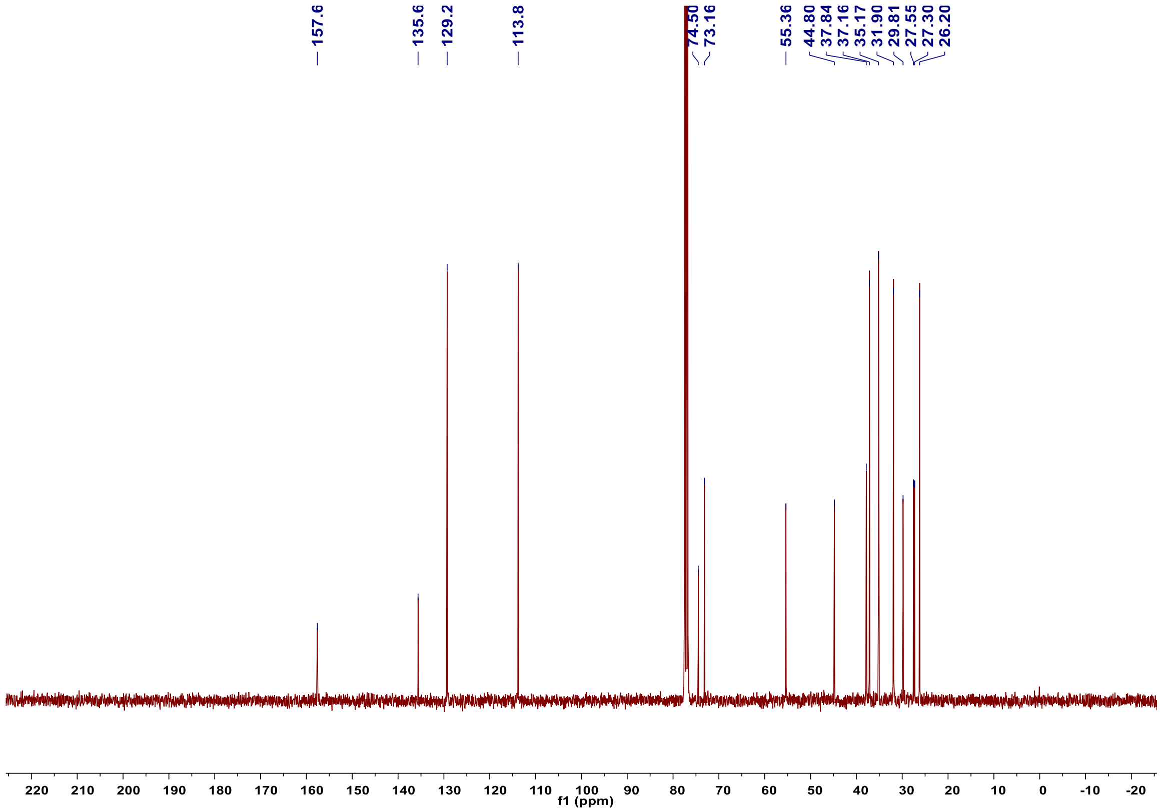

Supplementary Figure 187. ^{13}C NMR (100 MHz, $CDCl_3$) spectrum for 72

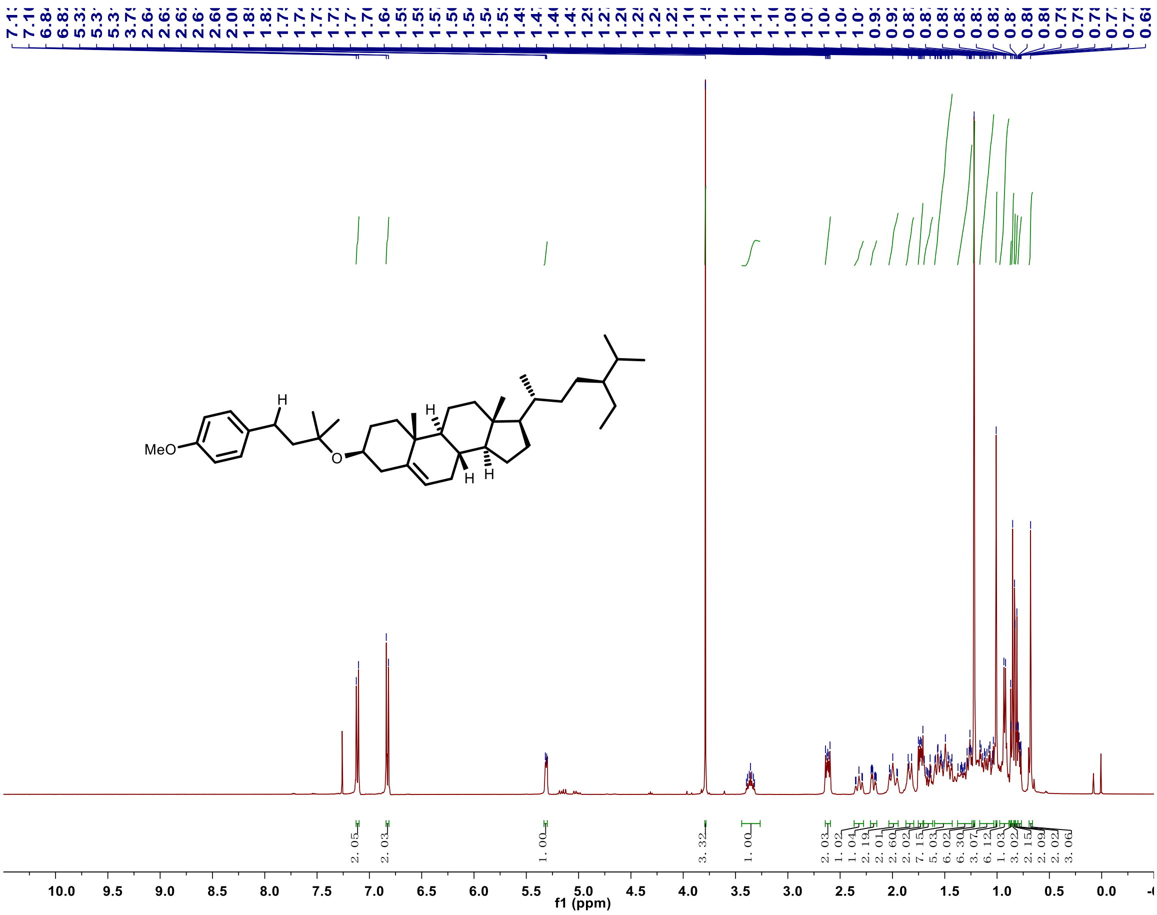

Supplementary Figure 188. ^{1}H **NMR (400 MHz,** $CDCl_3$**) spectrum for 73**

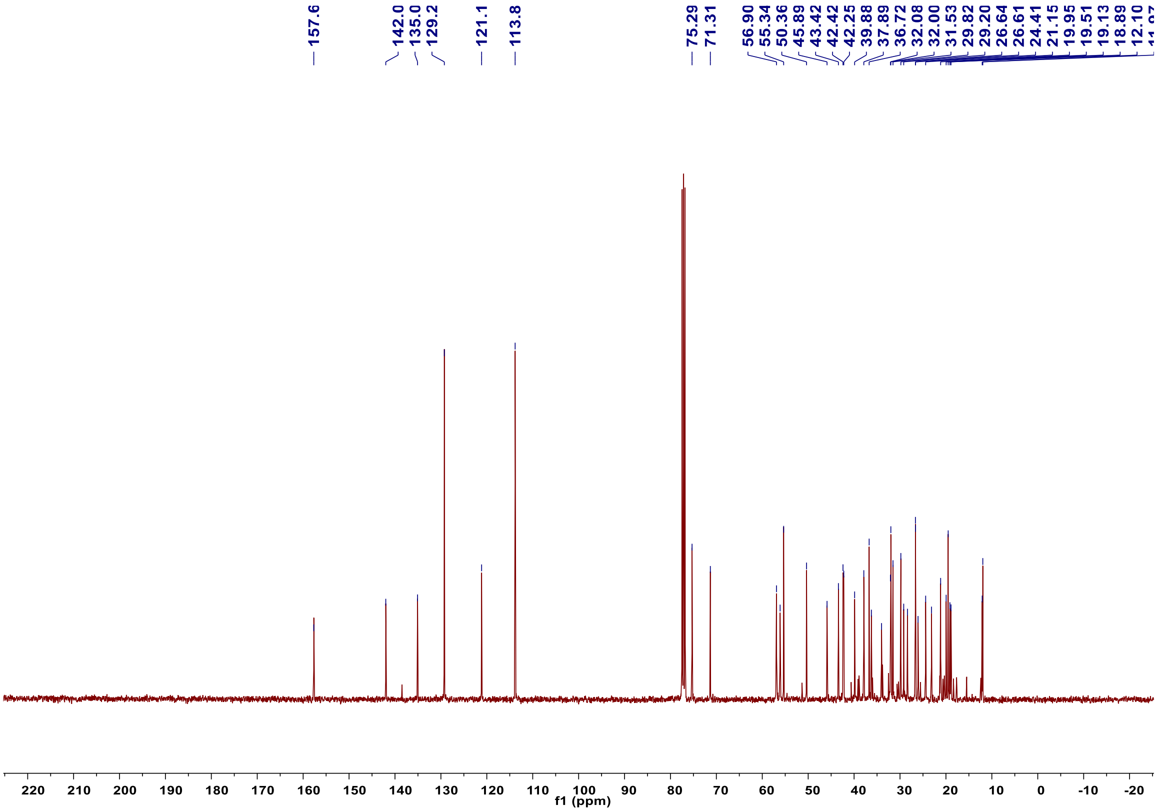

Supplementary Figure 189. ^{13}C **NMR (100 MHz,** $CDCl_3$**) spectrum for 73**

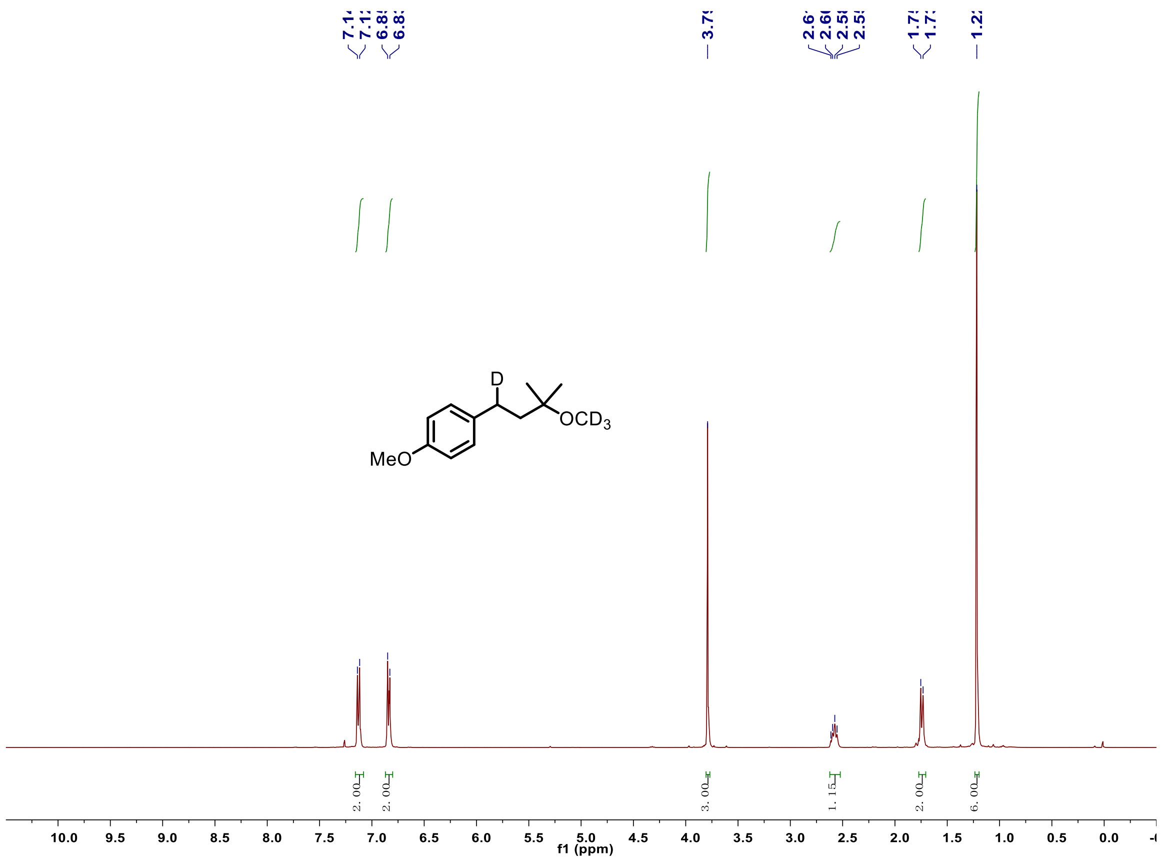

Supplementary Figure 190. ^{1}H NMR (400 MHz, $CDCl_3$) spectrum for 113

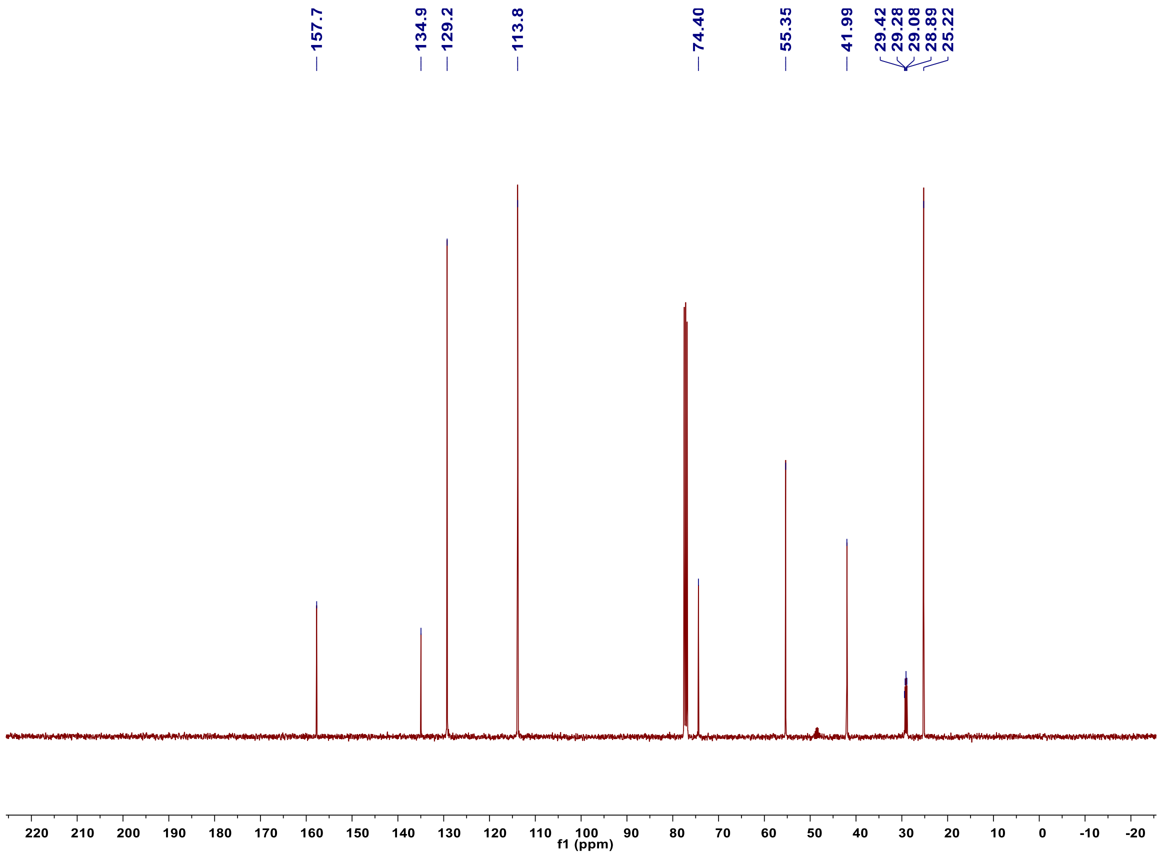

Supplementary Figure 191. ^{13}C NMR (100 MHz, $CDCl_3$) spectrum for 113

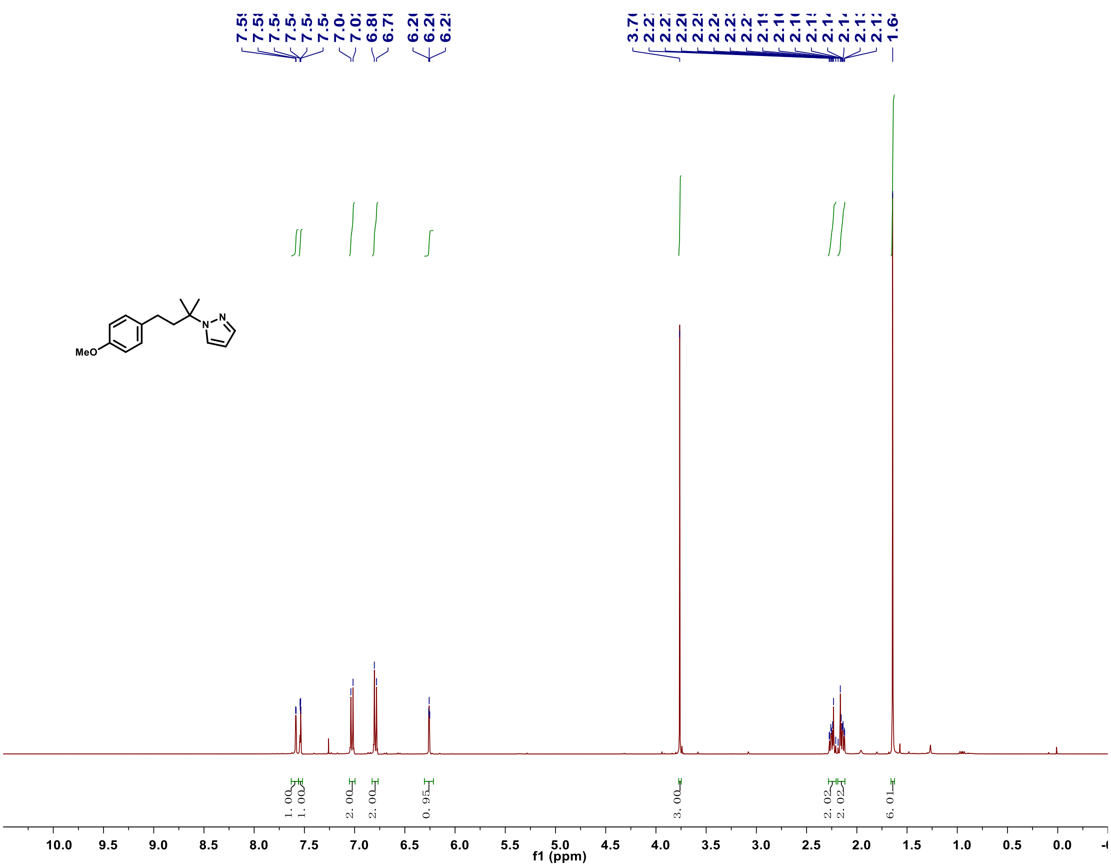

Supplementary Figure 192. ^{1}H NMR (400 MHz, $CDCl_3$) spectrum for 27

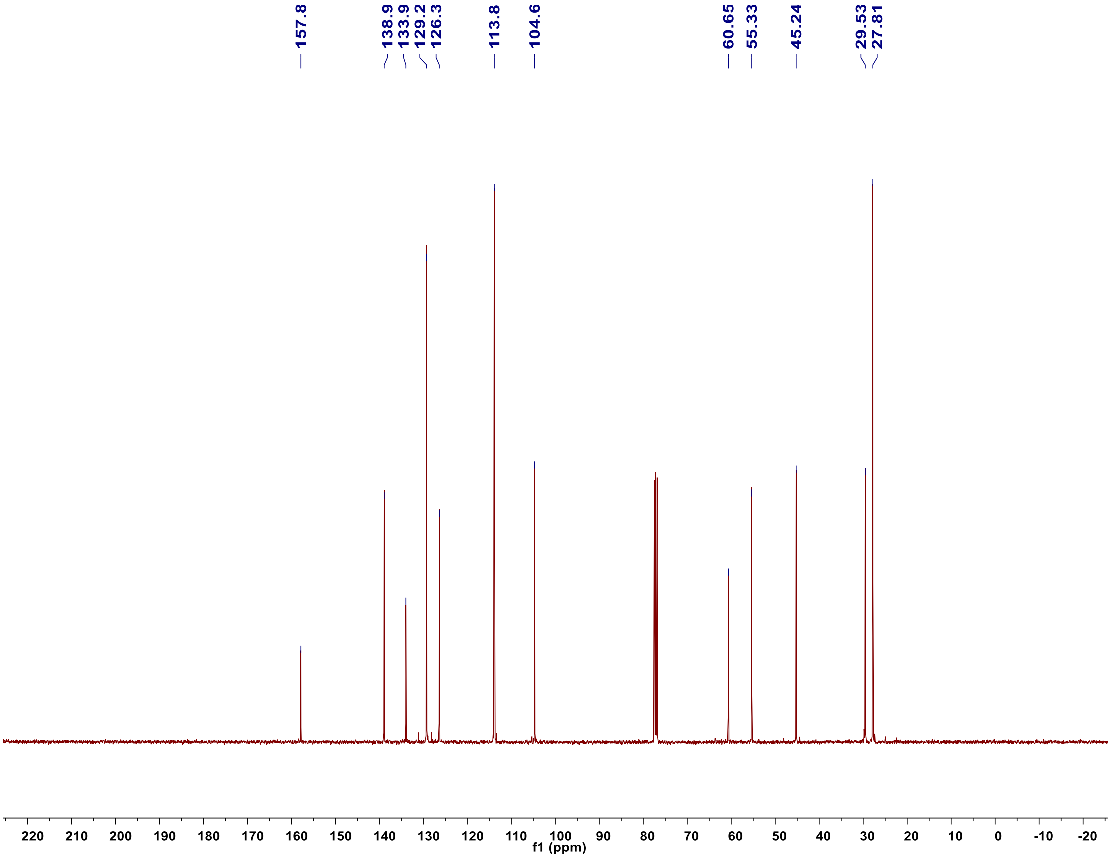

Supplementary Figure 193. ^{13}C NMR (100 MHz, $CDCl_3$) spectrum for 27

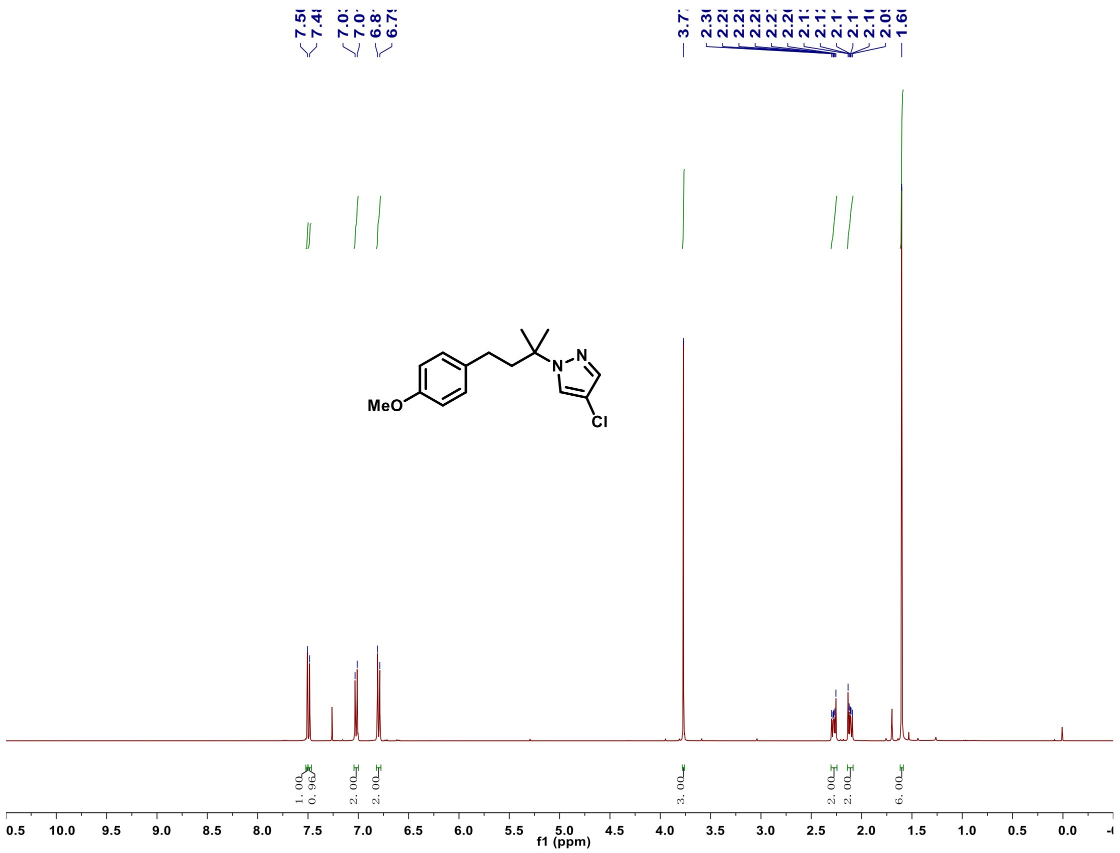

Supplementary Figure 194. ^{1}H NMR (400 MHz, $CDCl_3$) spectrum for 88

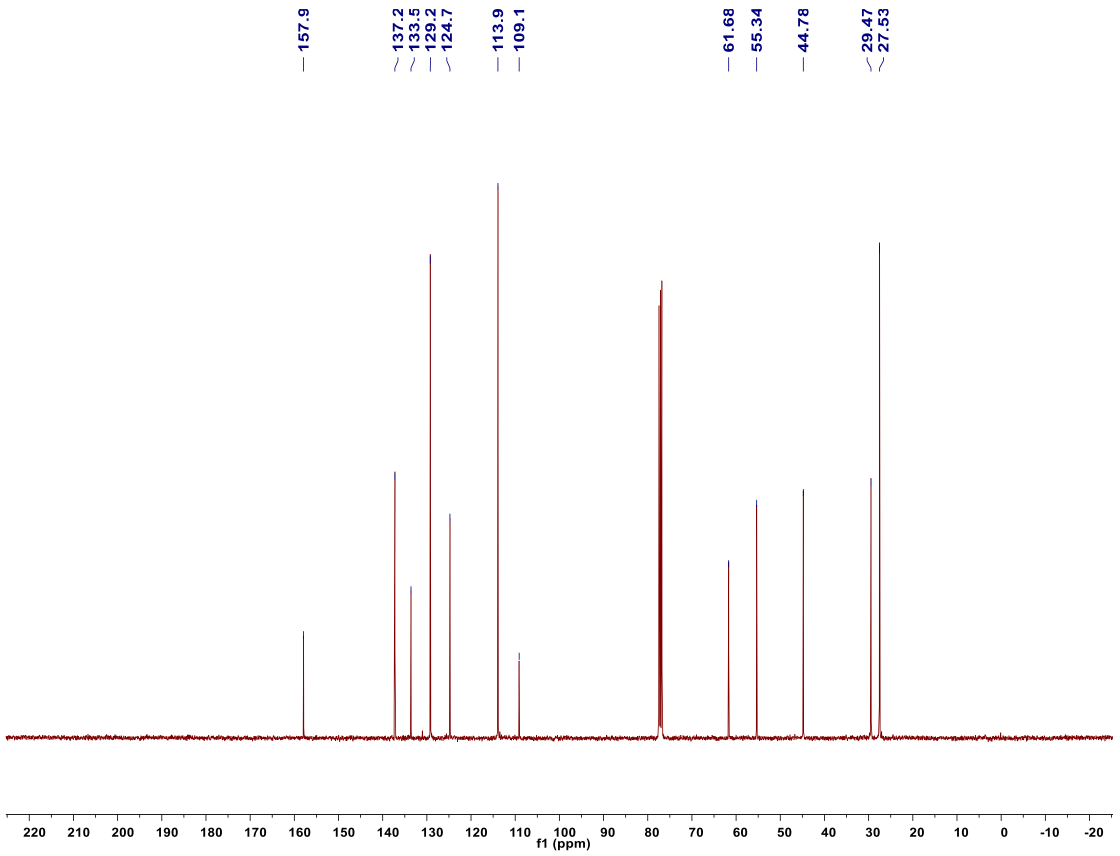

Supplementary Figure 195. ^{13}C NMR (100 MHz, $CDCl_3$) spectrum for 88

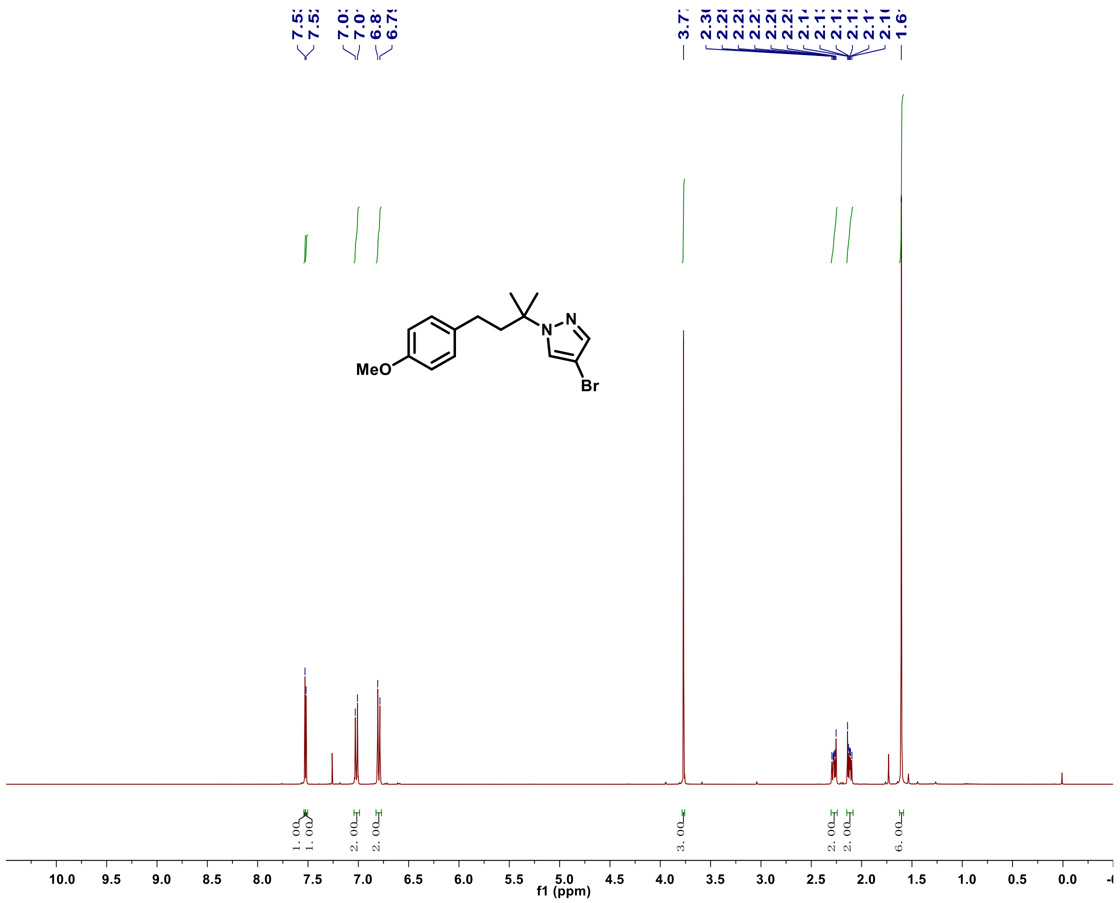

Supplementary Figure 196. ^{1}H NMR (400 MHz, $CDCl_3$) spectrum for 89

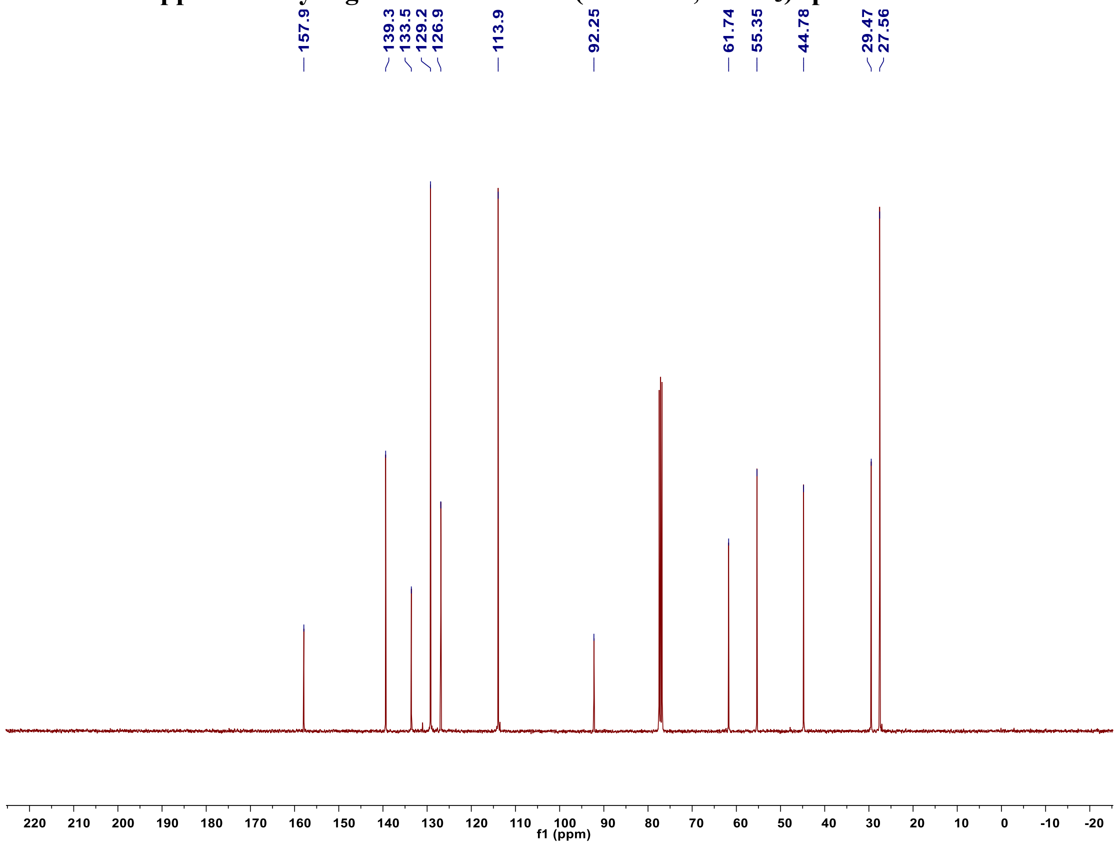

Supplementary Figure 197. ^{13}C NMR (100 MHz, $CDCl_3$) spectrum for 89

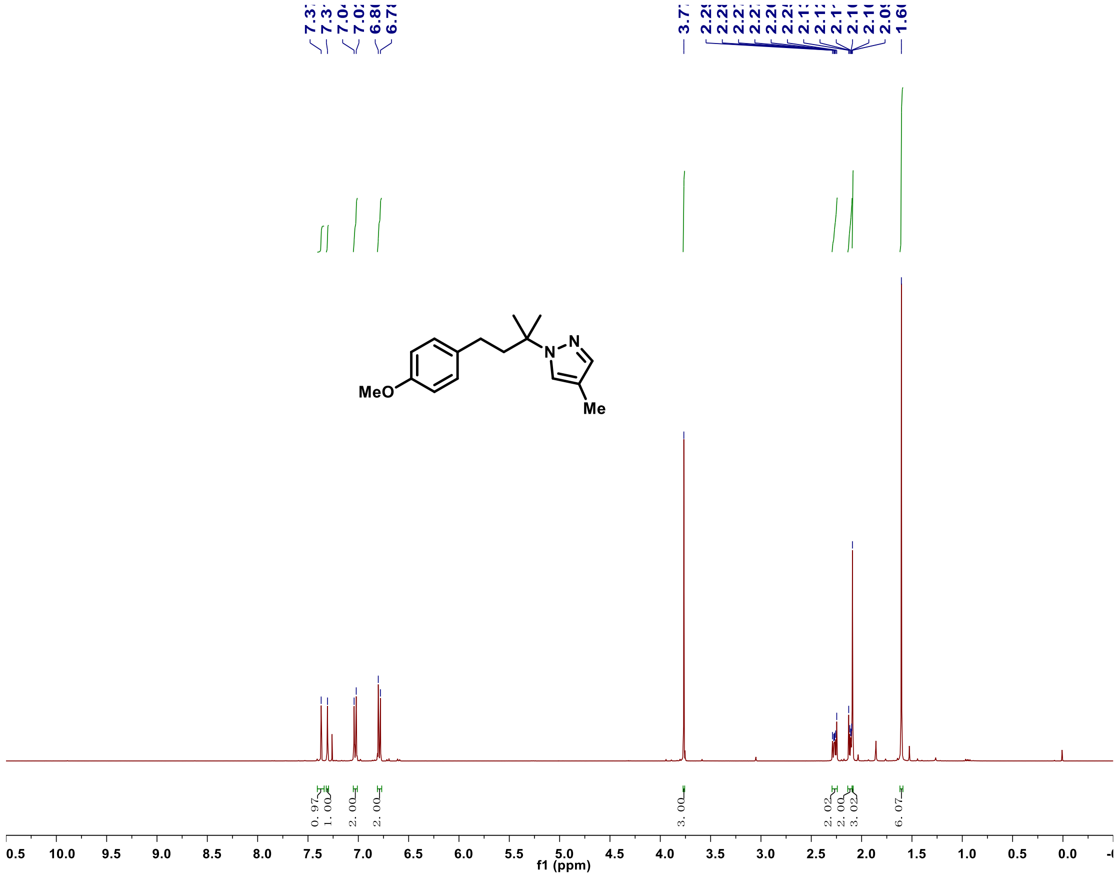

Supplementary Figure 198. ^{1}H NMR (400 MHz, $CDCl_3$) spectrum for 90

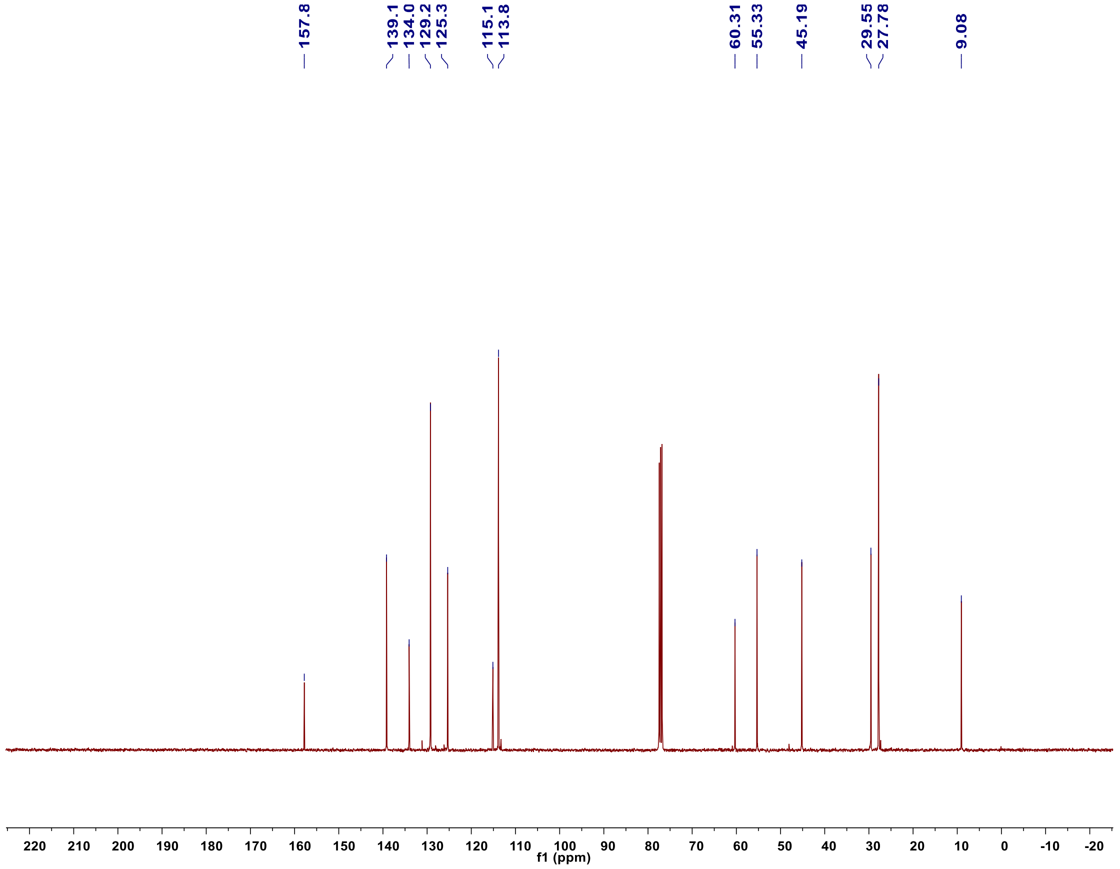

Supplementary Figure 199. ^{13}C NMR (100 MHz, $CDCl_3$) spectrum for 90

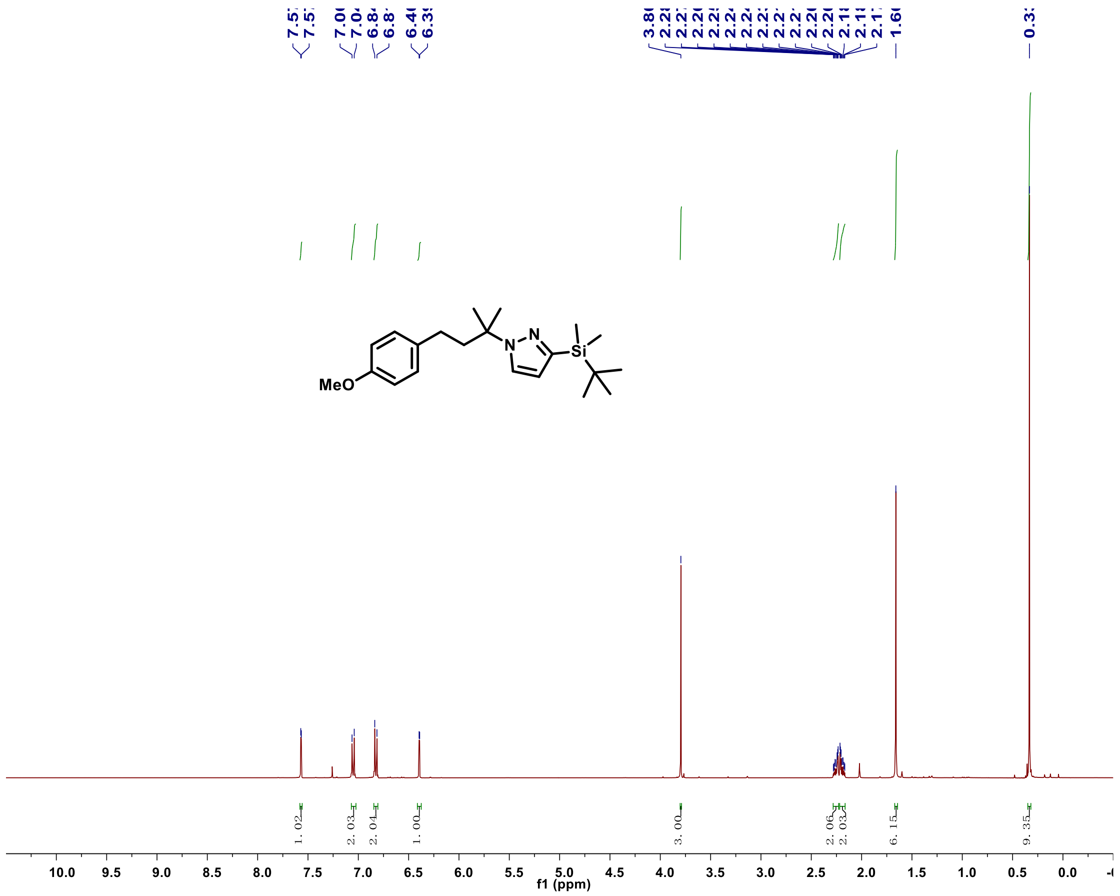

Supplementary Figure 200. **^{1}H NMR (400 MHz, $CDCl_3$) spectrum for 91**

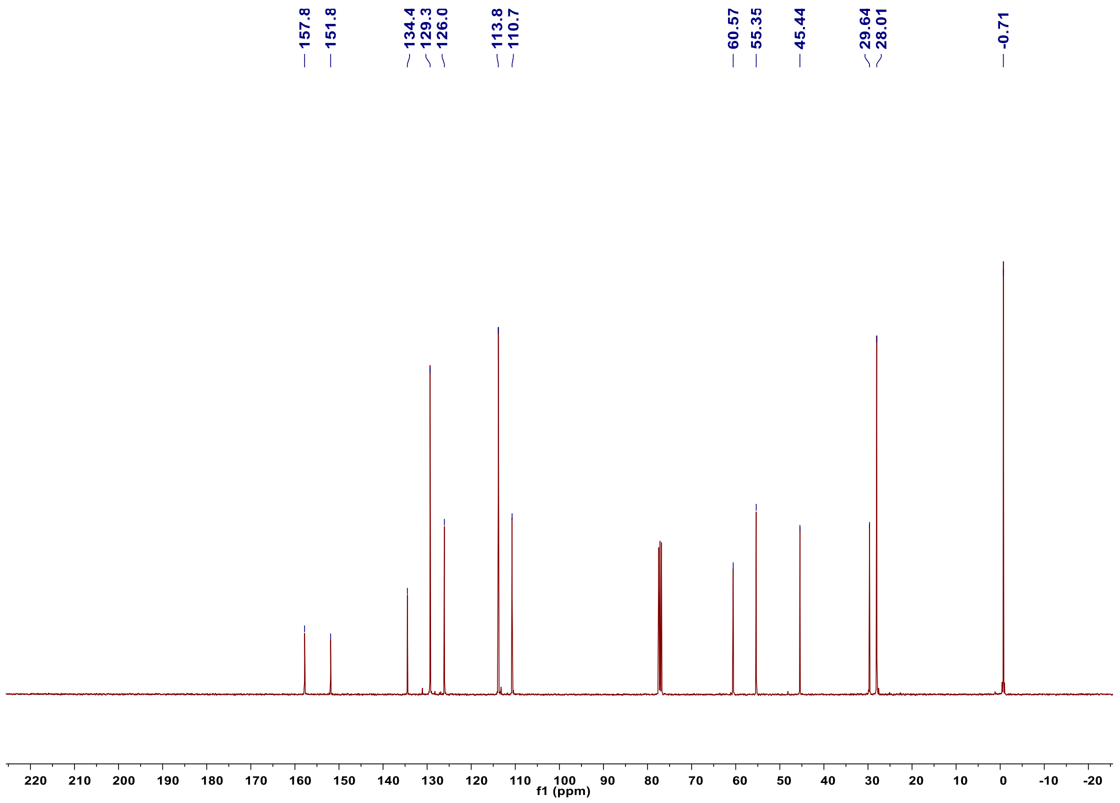

Supplementary Figure 201. **^{13}C NMR (100 MHz, $CDCl_3$) spectrum for 91**

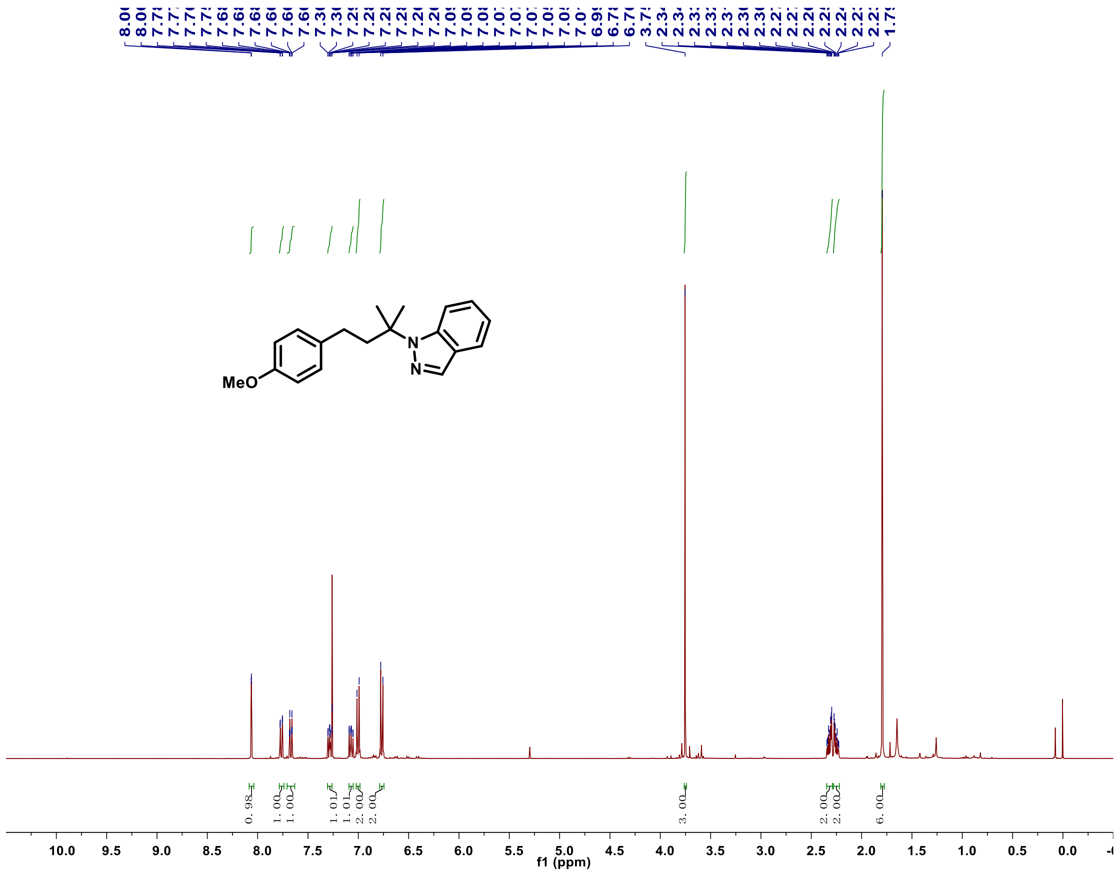

Supplementary Figure 202. ^{1}**H NMR (400 MHz,** $CDCl_3$**) spectrum for 92**

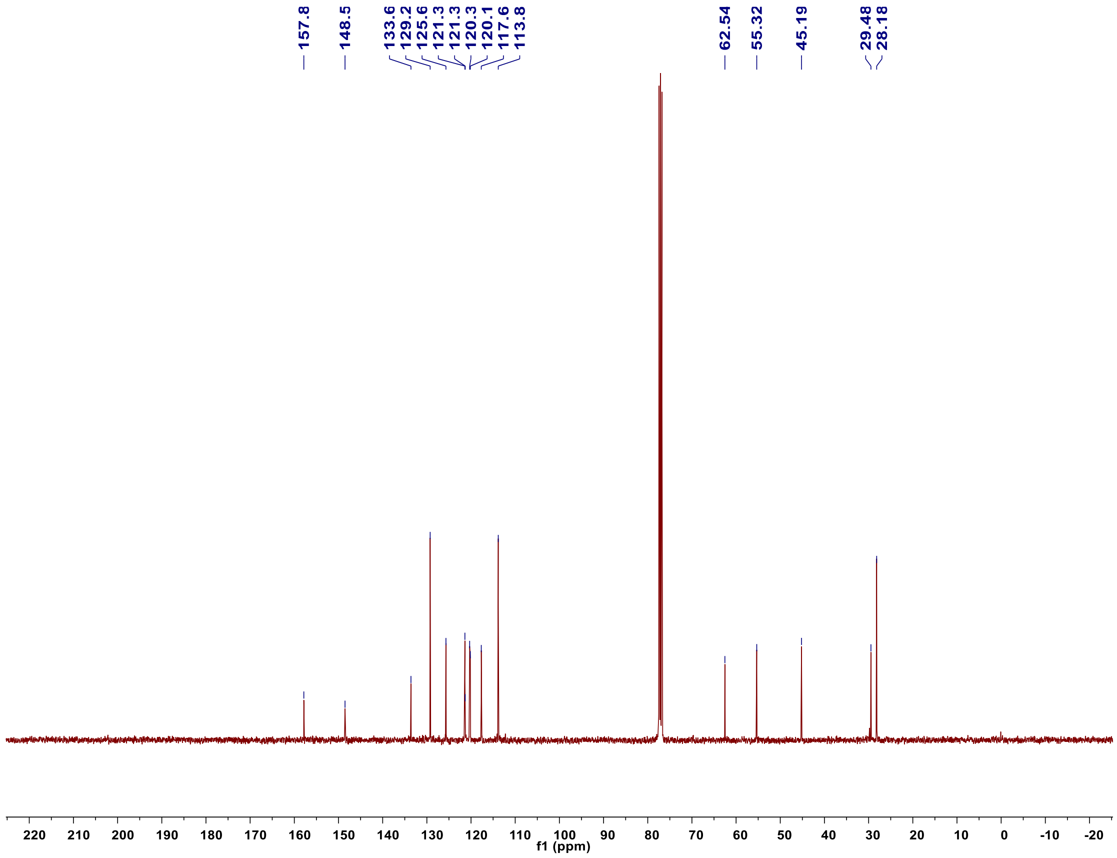

Supplementary Figure 203. ^{13}**C NMR (100 MHz,** $CDCl_3$**) spectrum for 92**

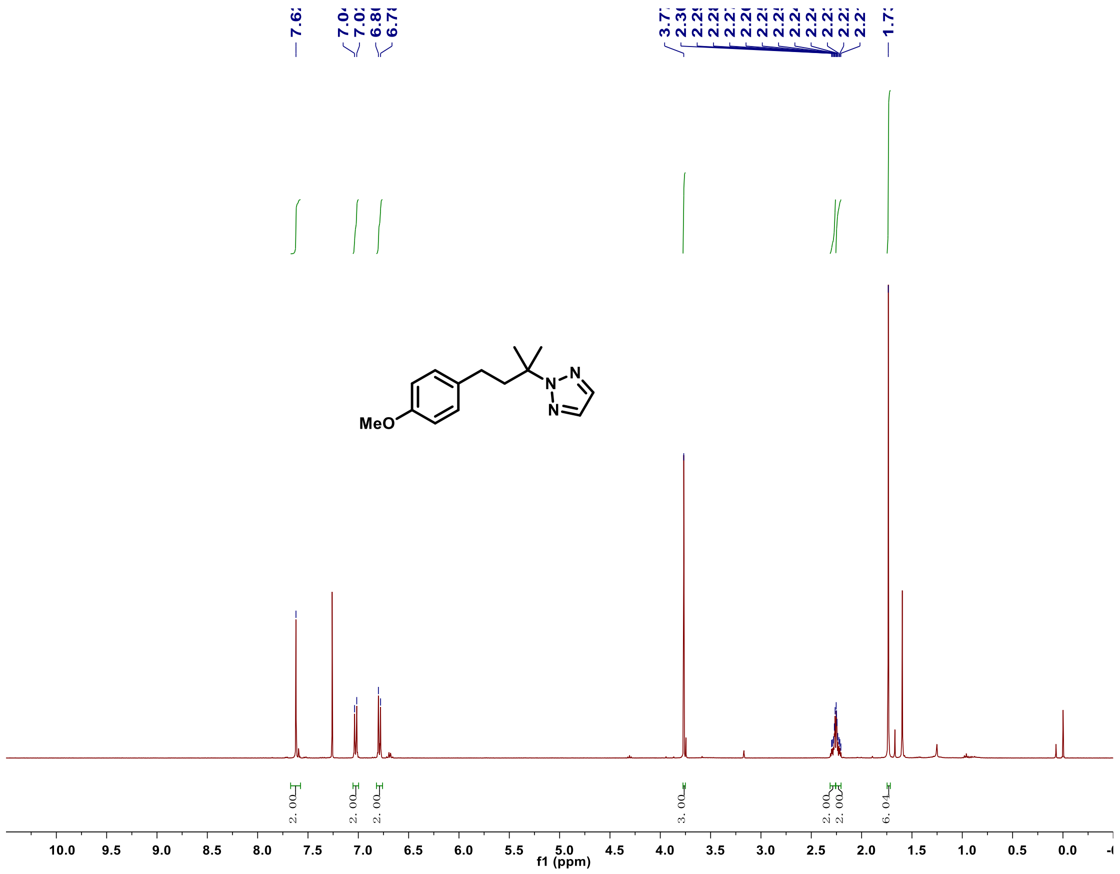

Supplementary Figure 204. ^{1}H NMR (400 MHz, $CDCl_3$) spectrum for 93

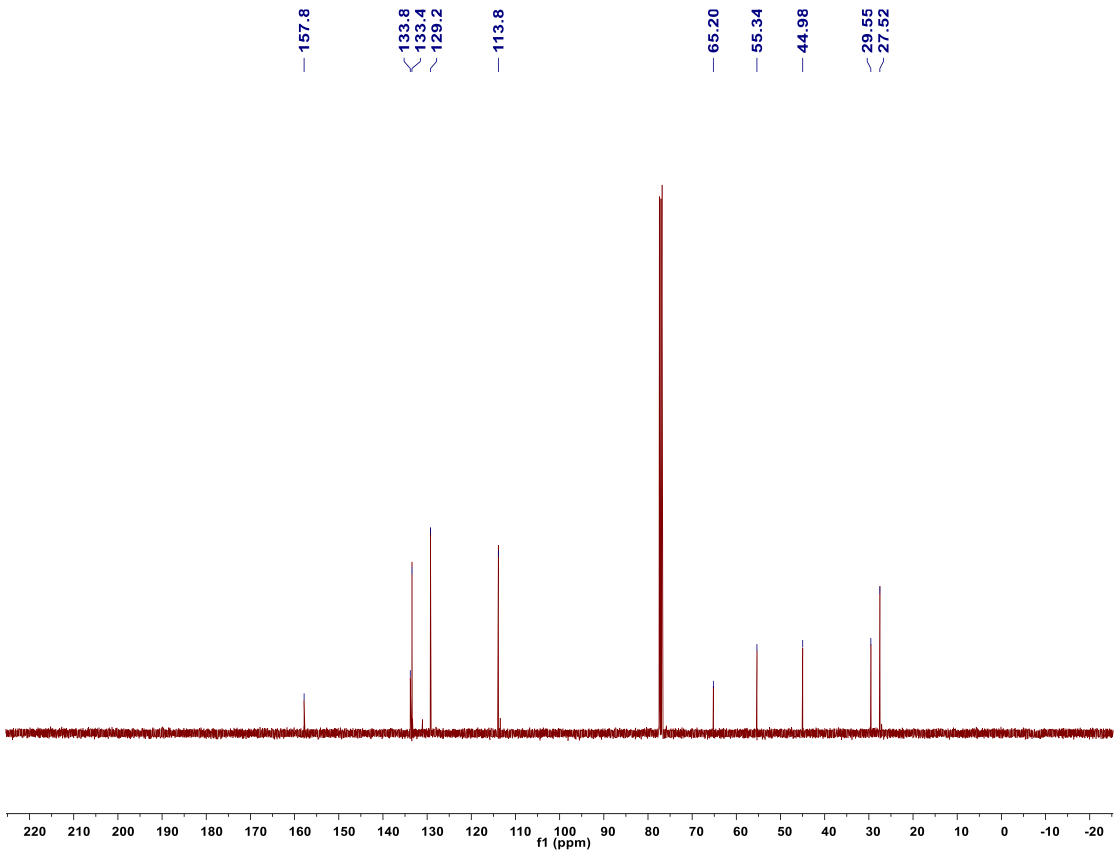

Supplementary Figure 205. ^{13}C NMR (100 MHz, $CDCl_3$) spectrum for 93

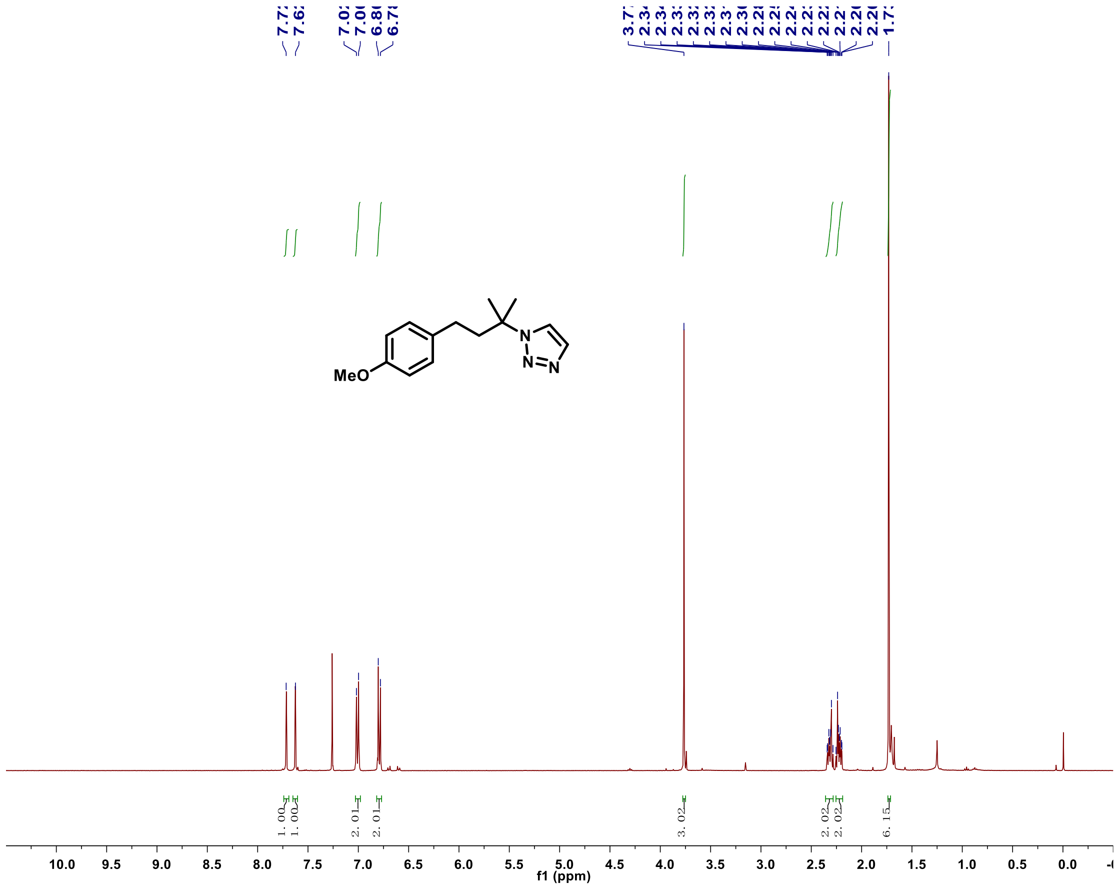

Supplementary Figure 206. ^{1}H NMR (400 MHz, $CDCl_3$) spectrum for 94

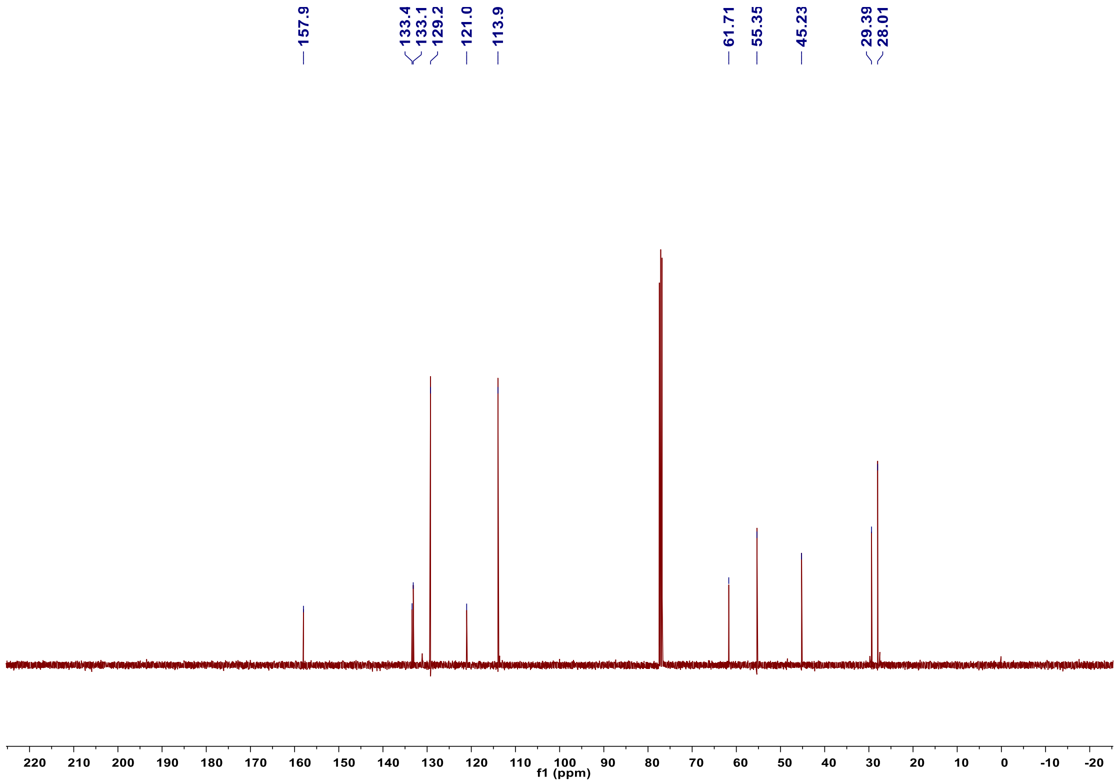

Supplementary Figure 207. ^{13}C NMR (100 MHz, $CDCl_3$) spectrum for 94

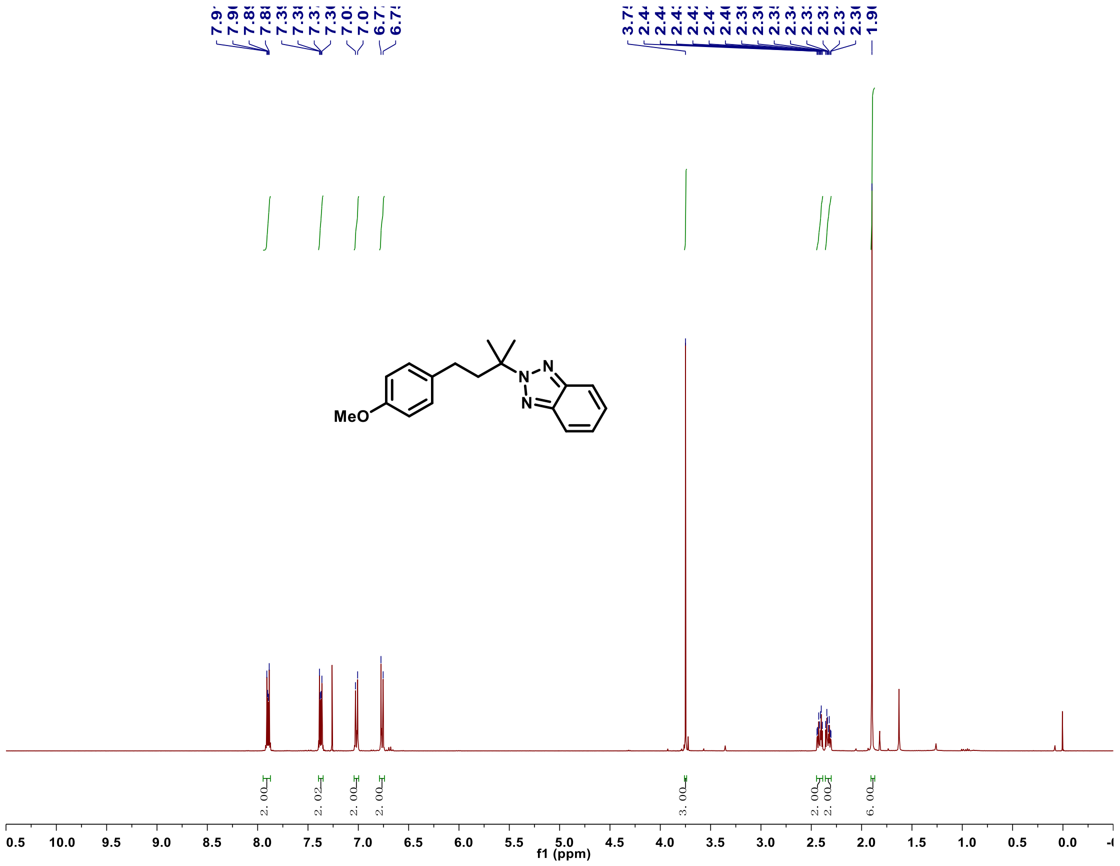

Supplementary Figure 208. ^{1}H NMR (400 MHz, $CDCl_3$) spectrum for 95

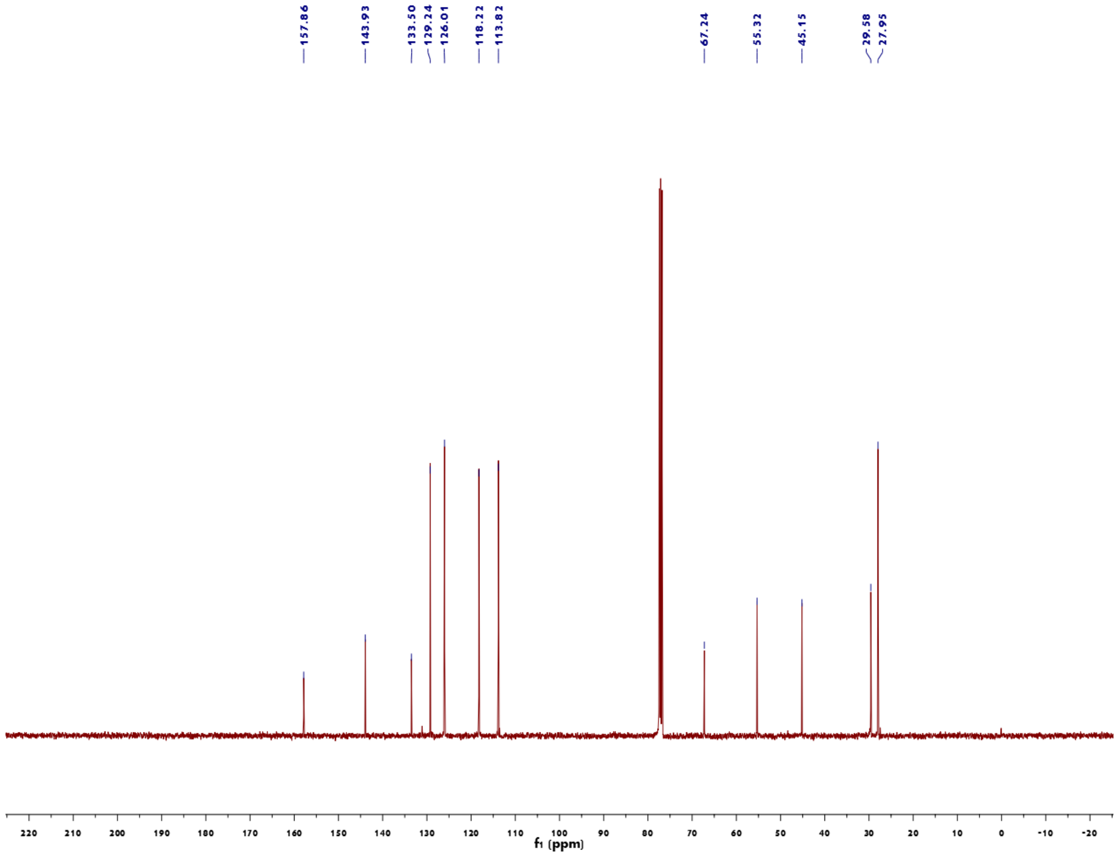

Supplementary Figure 209. ^{13}C NMR (100 MHz, $CDCl_3$) spectrum for 95

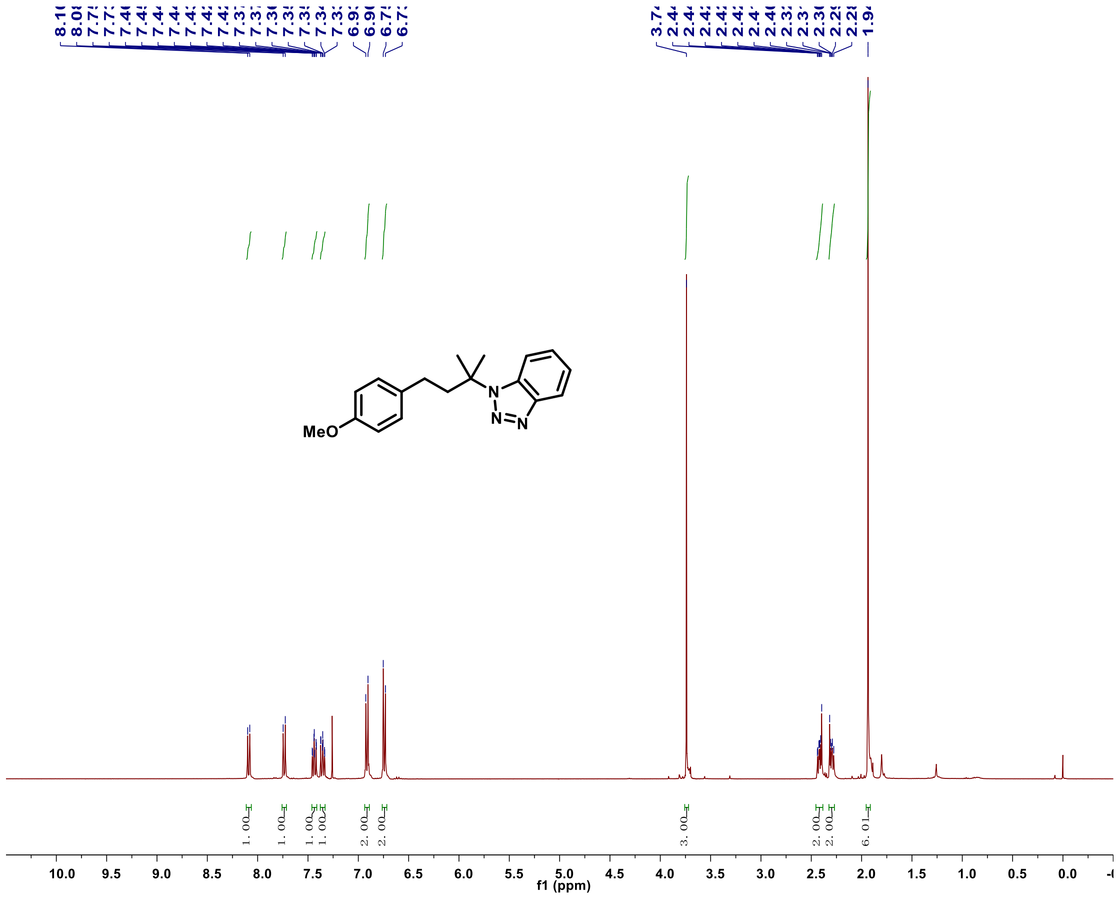

Supplementary Figure 210. ^{1}H NMR (400 MHz, $CDCl_3$) spectrum for 96

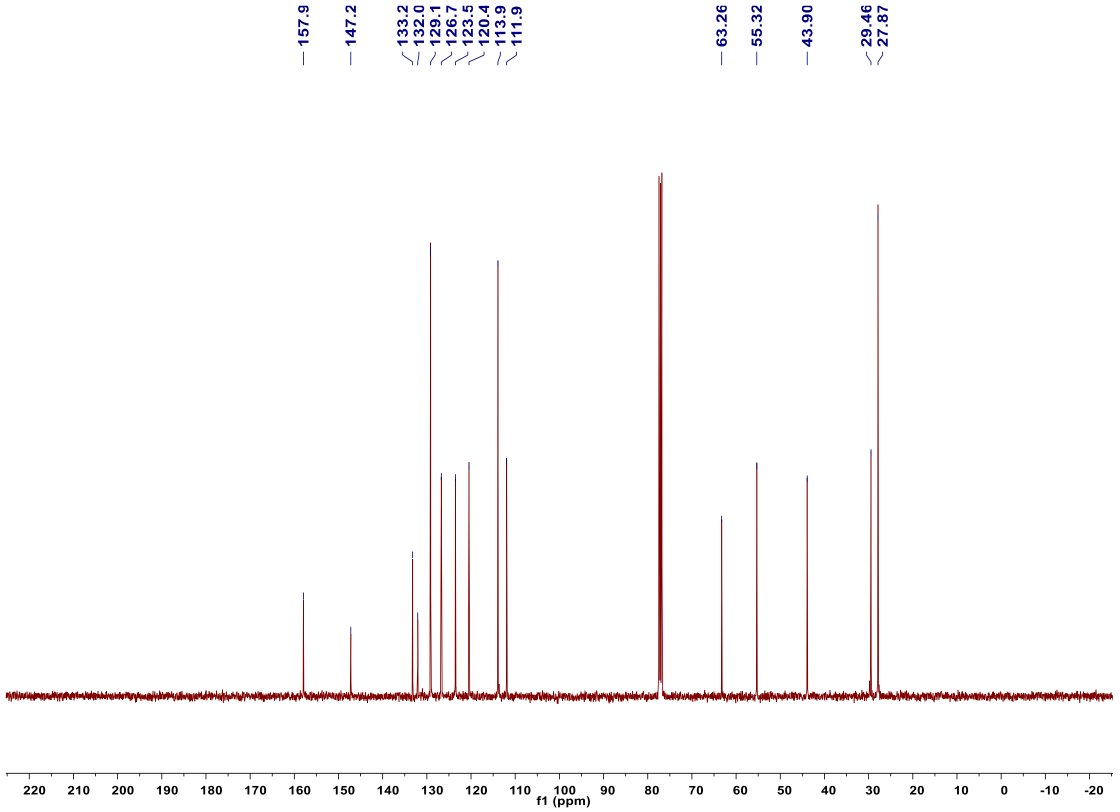

Supplementary Figure 211. ^{13}C NMR (100 MHz, $CDCl_3$) spectrum for 96

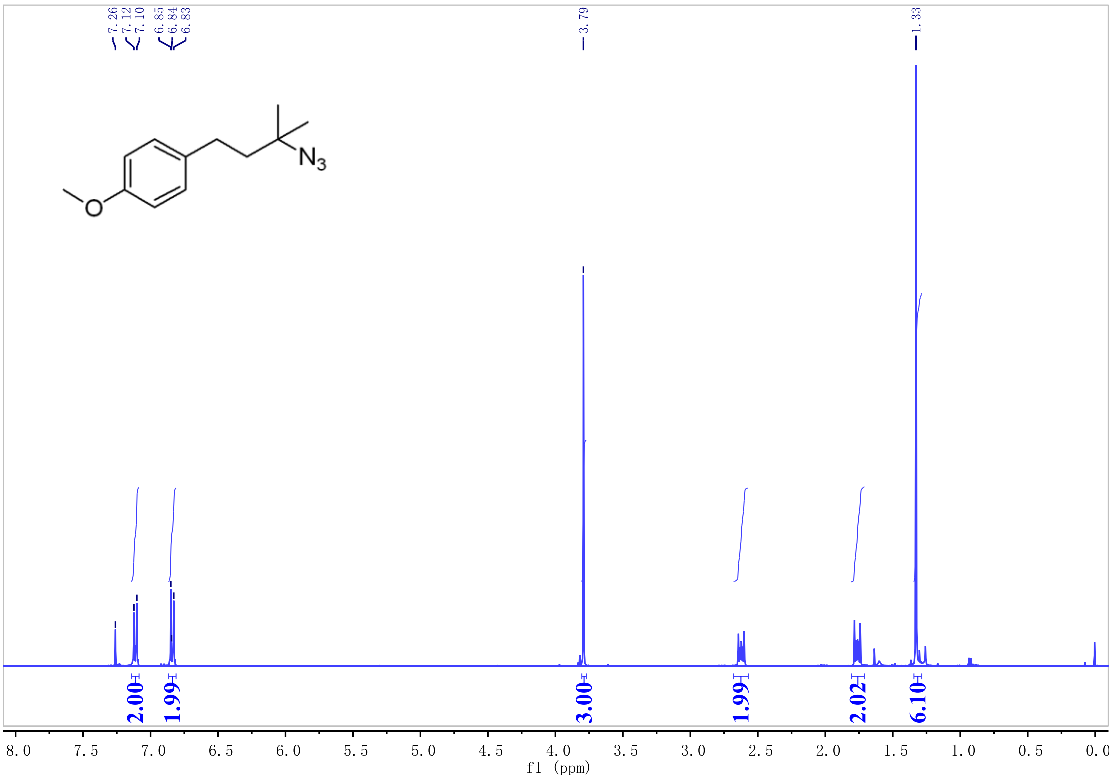

Supplementary Figure 212. ^{1}H NMR (400 MHz, $CDCl_3$) spectrum for 97

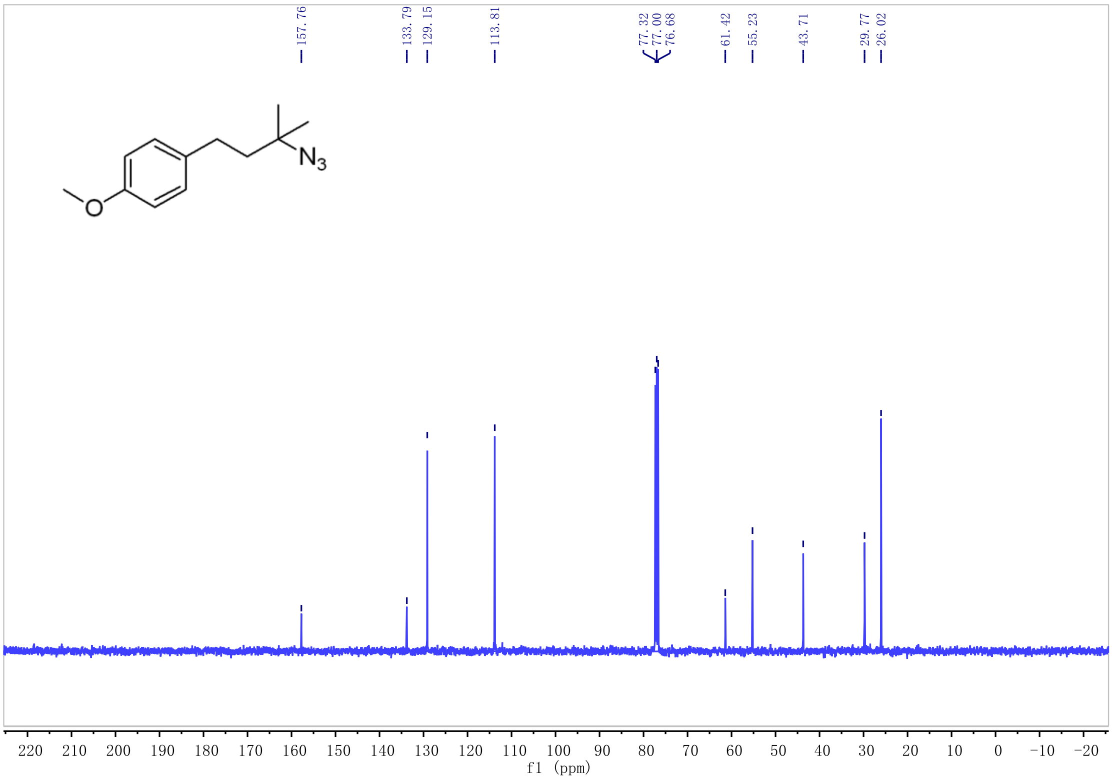

Supplementary Figure 213. ^{13}C NMR (100 MHz, $CDCl_3$) spectrum for 97

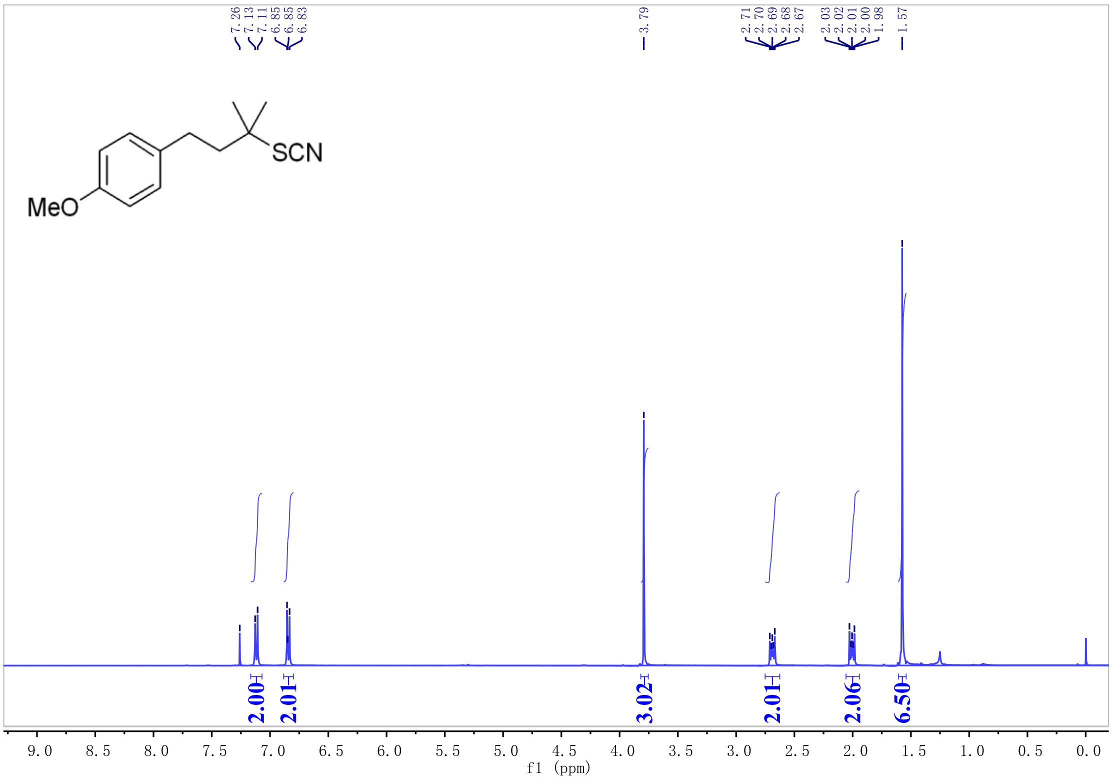

Supplementary Figure 214. ^{1}H NMR (400 MHz, $CDCl_3$) spectrum for **98**

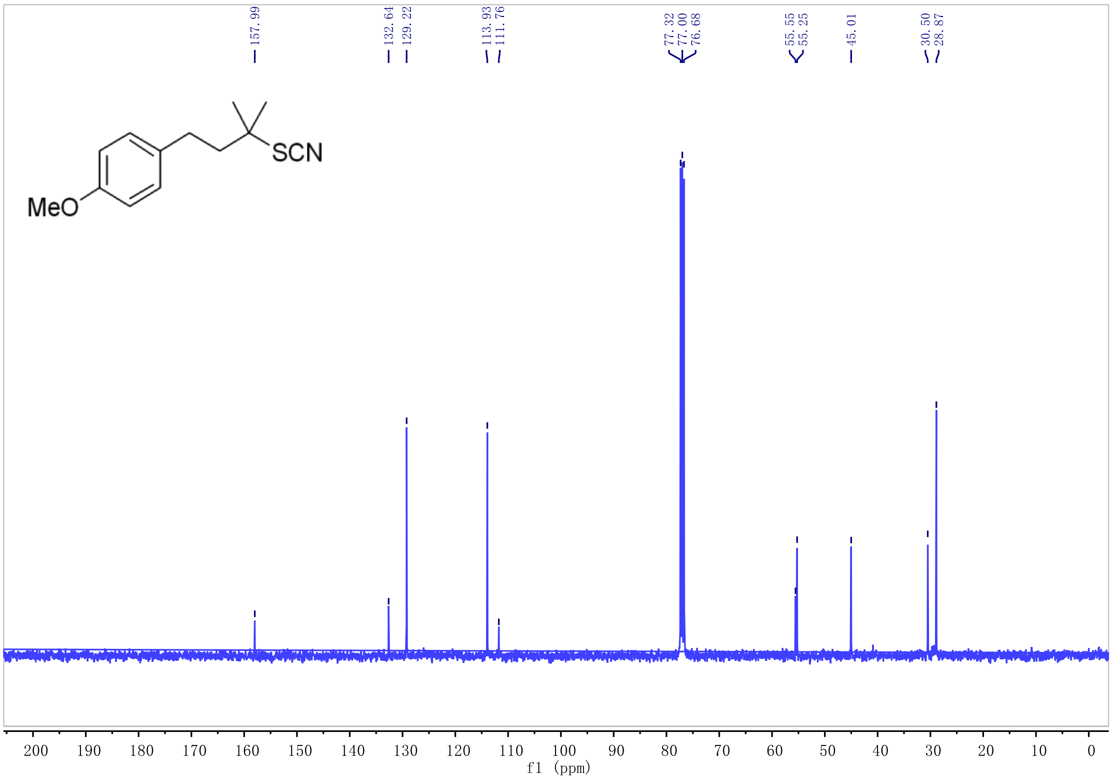

Supplementary Figure 215. ^{13}C NMR (100 MHz, $CDCl_3$) spectrum for **98**

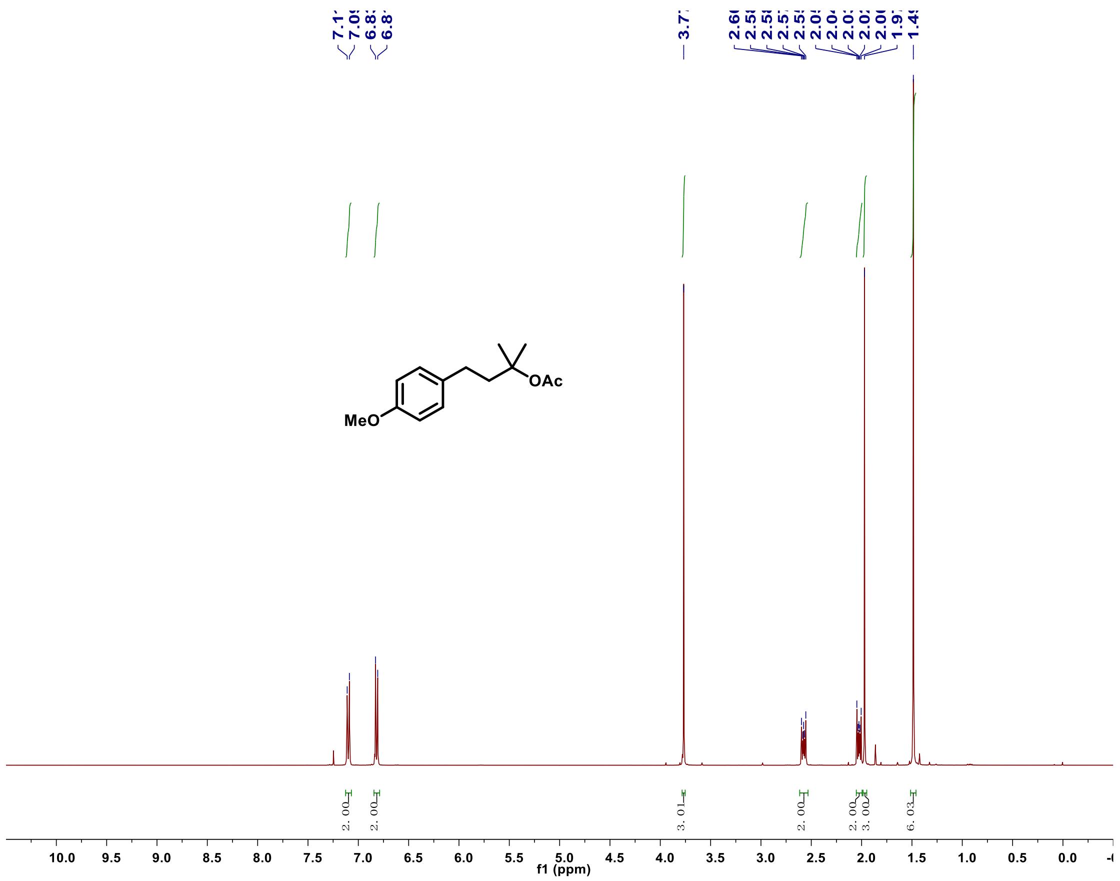

Supplementary Figure 216. ^{1}H **NMR (400 MHz,** $CDCl_3$**) spectrum for 74**

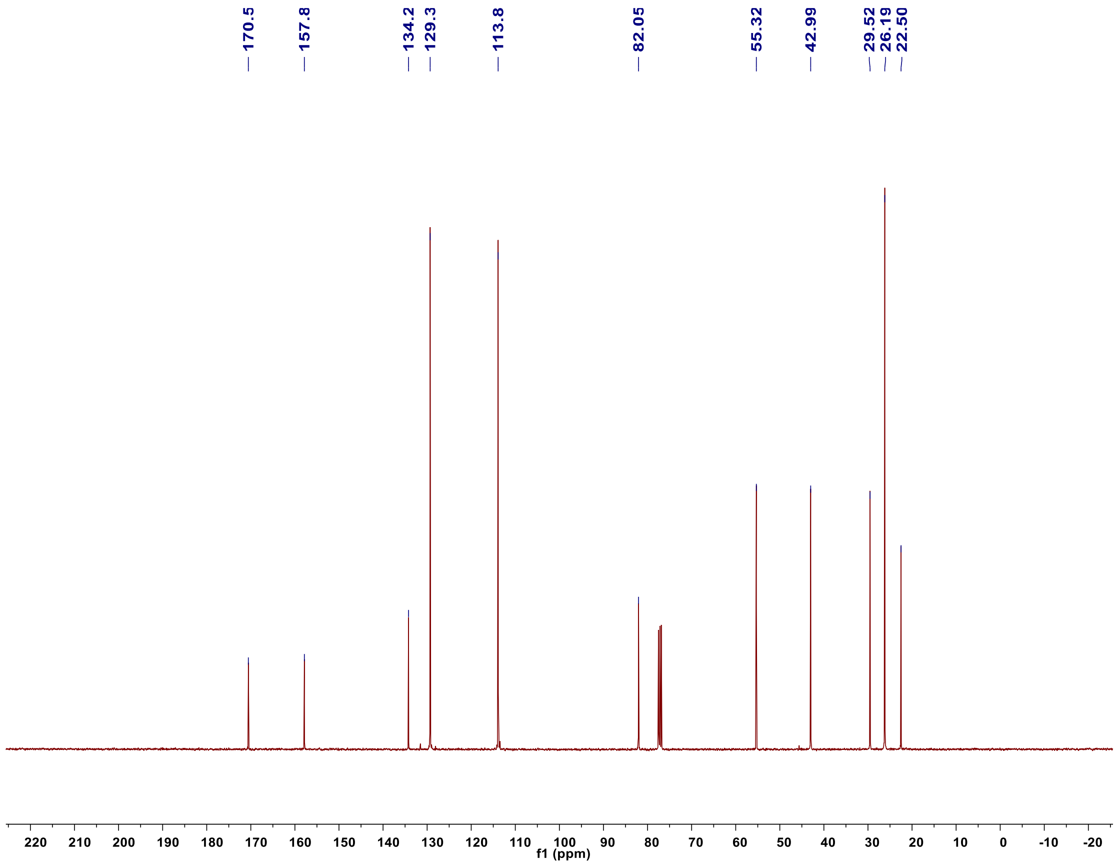

Supplementary Figure 217. ^{13}C **NMR (100 MHz,** $CDCl_3$**) spectrum for 74**

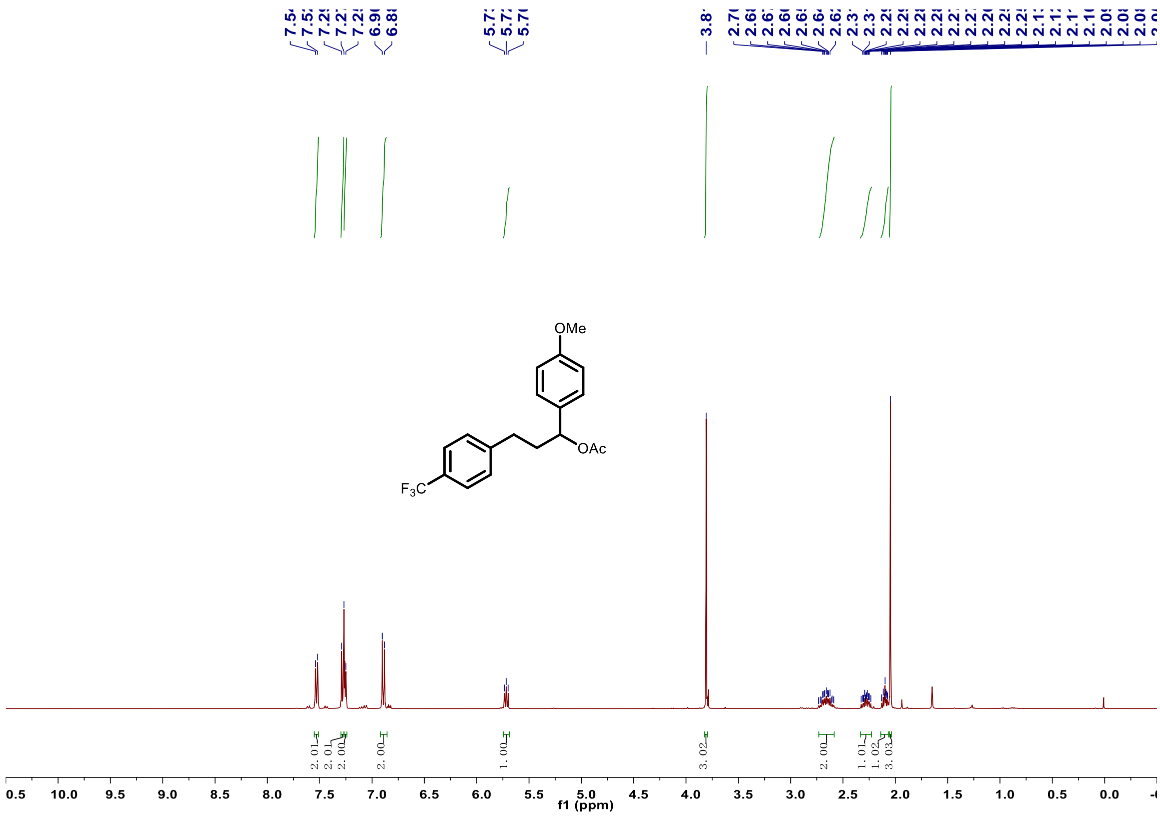

Supplementary Figure 218. ^{1}H NMR (400 MHz, $CDCl_3$) spectrum for 75

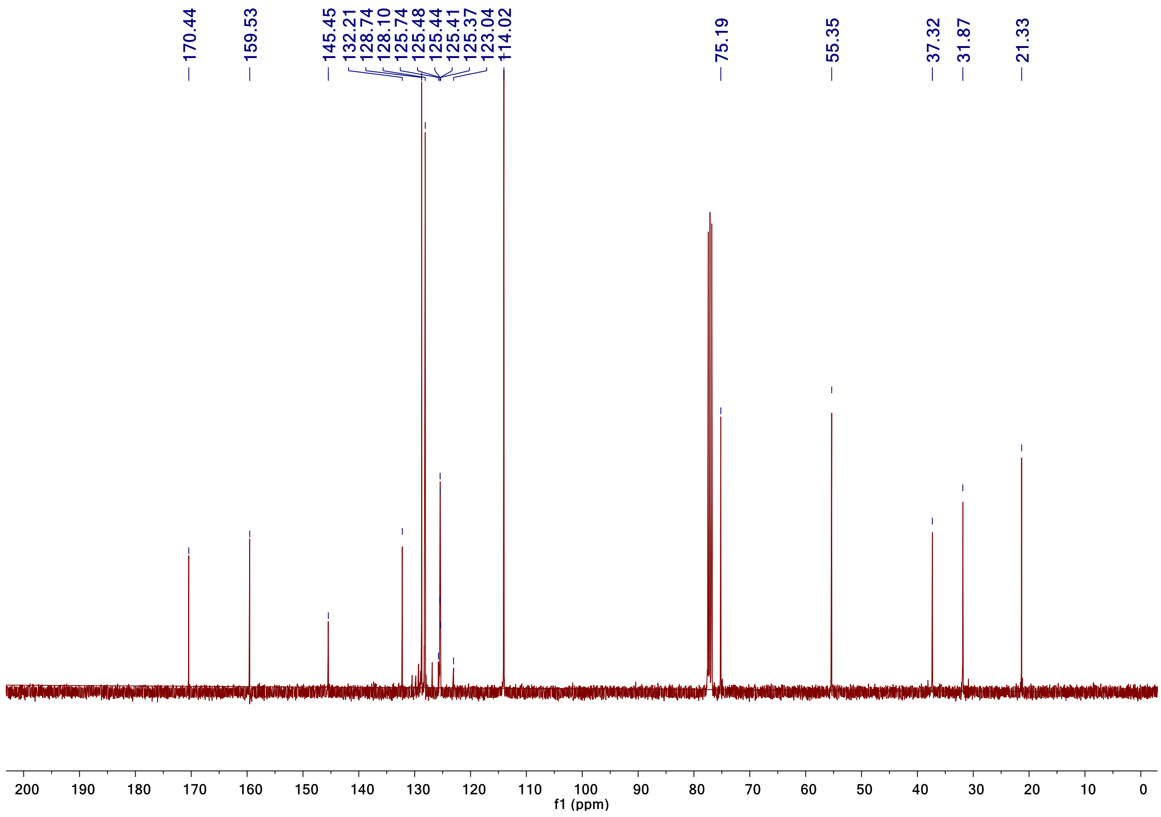

Supplementary Figure 219. ^{13}C NMR (100 MHz, $CDCl_3$) spectrum for 75

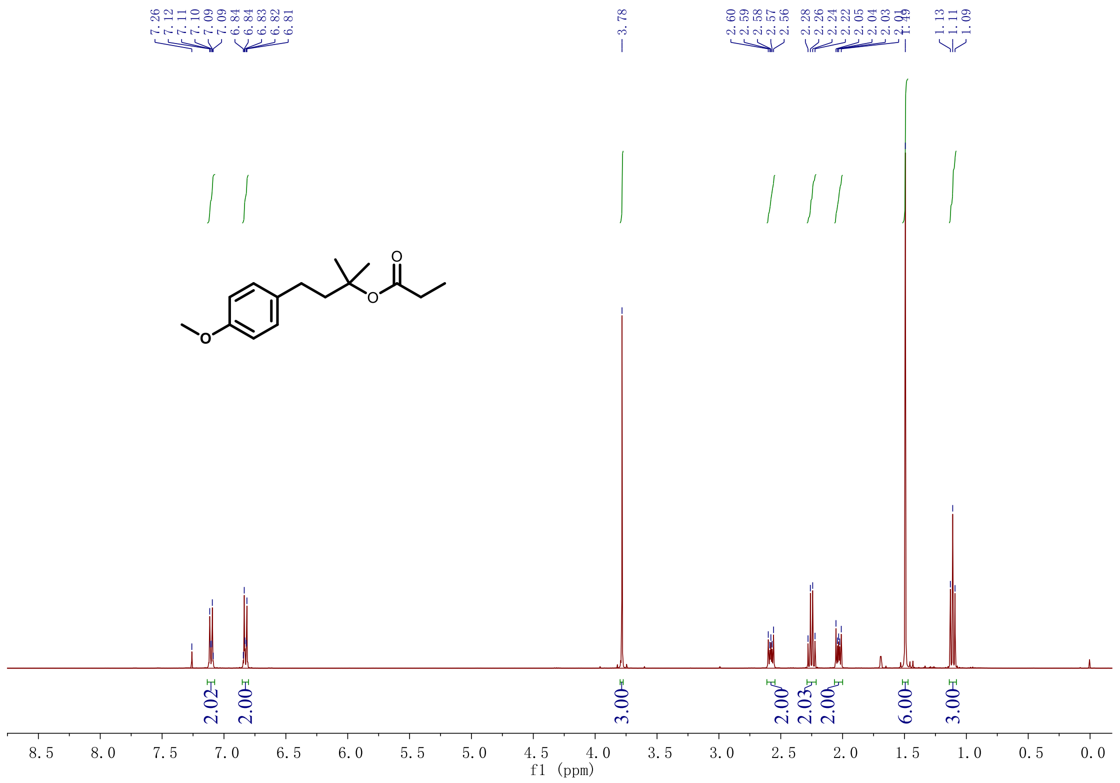

Supplementary Figure 220. ^{1}H NMR (400 MHz, $CDCl_3$) spectrum for 76

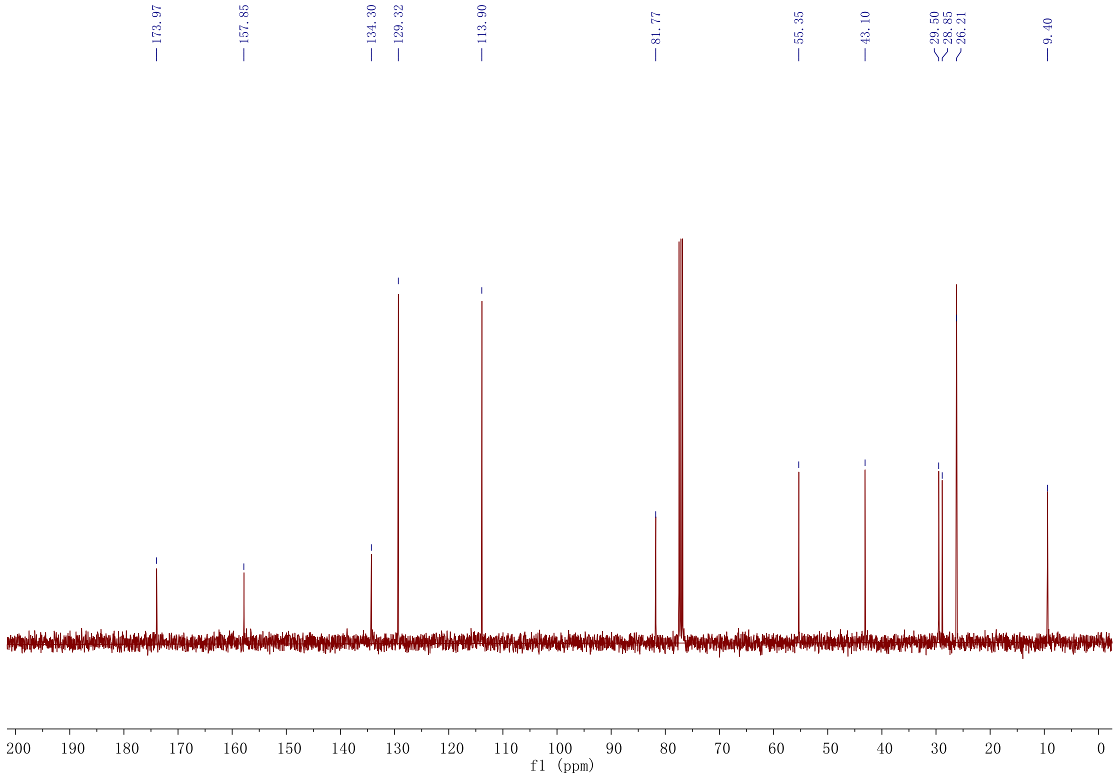

Supplementary Figure 221. ^{13}C NMR (100 MHz, $CDCl_3$) spectrum for 76

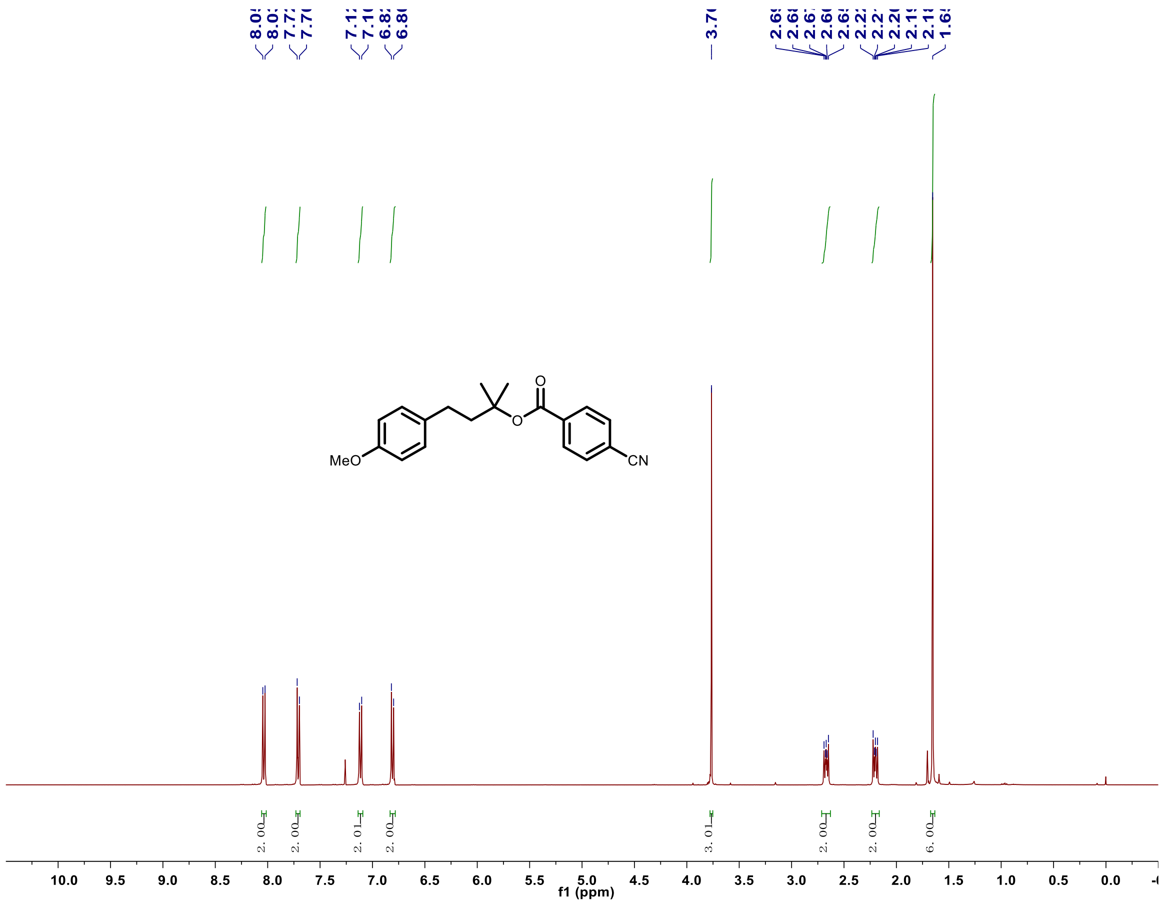

Supplementary Figure 222. ^{1}H NMR (400 MHz, $CDCl_3$) spectrum for 77

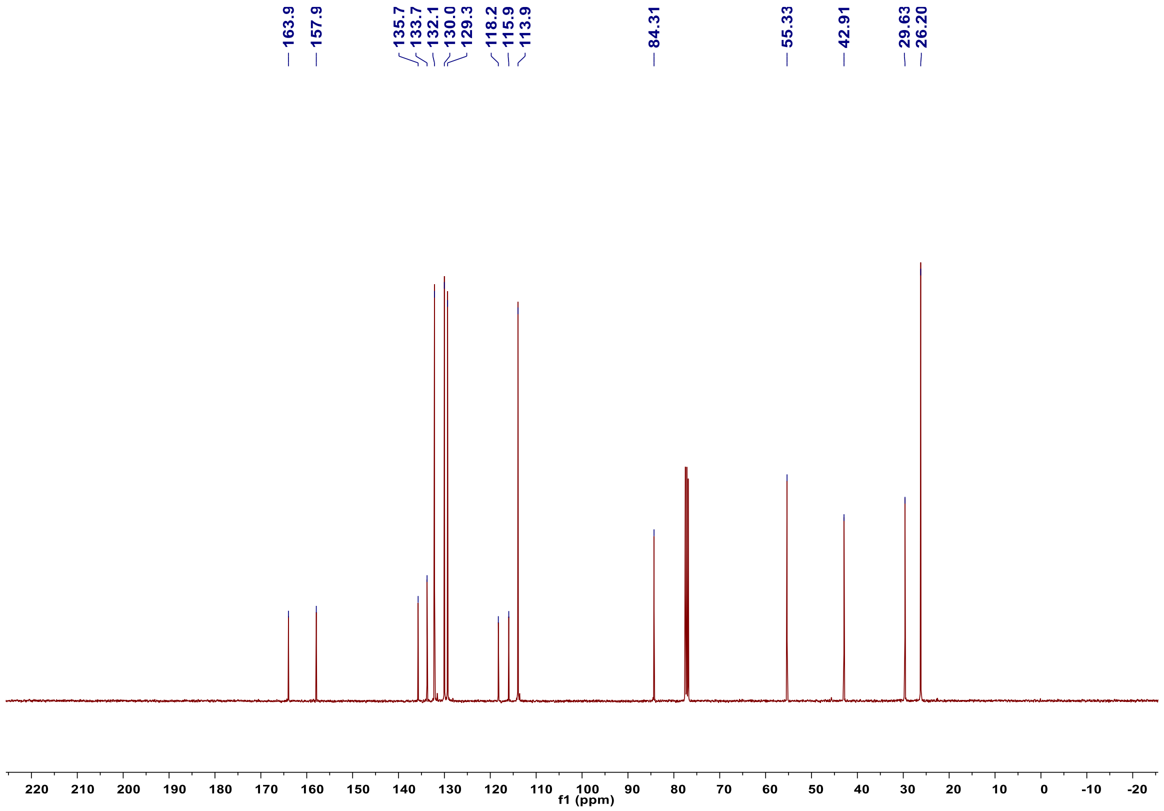

Supplementary Figure 223. ^{13}C NMR (100 MHz, $CDCl_3$) spectrum for 77

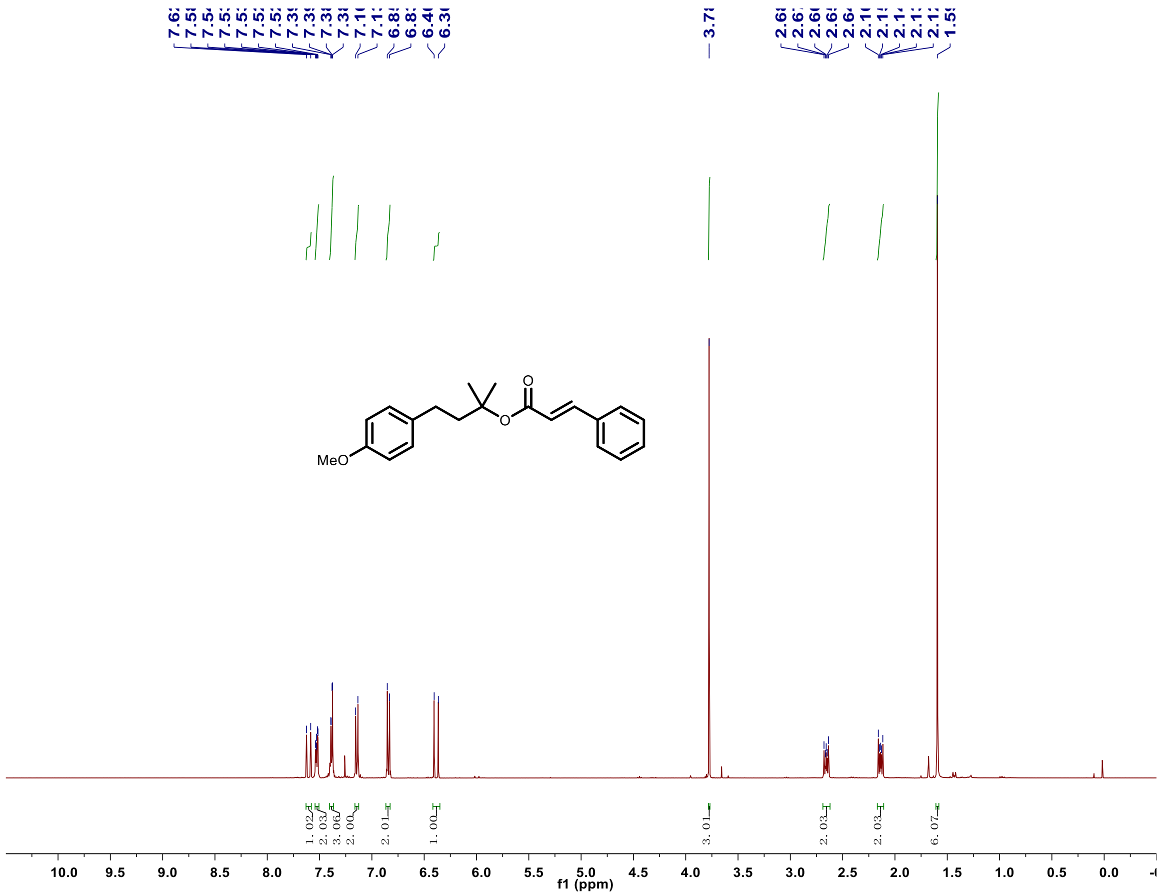

Supplementary Figure 224. ¹H NMR (400 MHz, CDCl₃) spectrum for 78

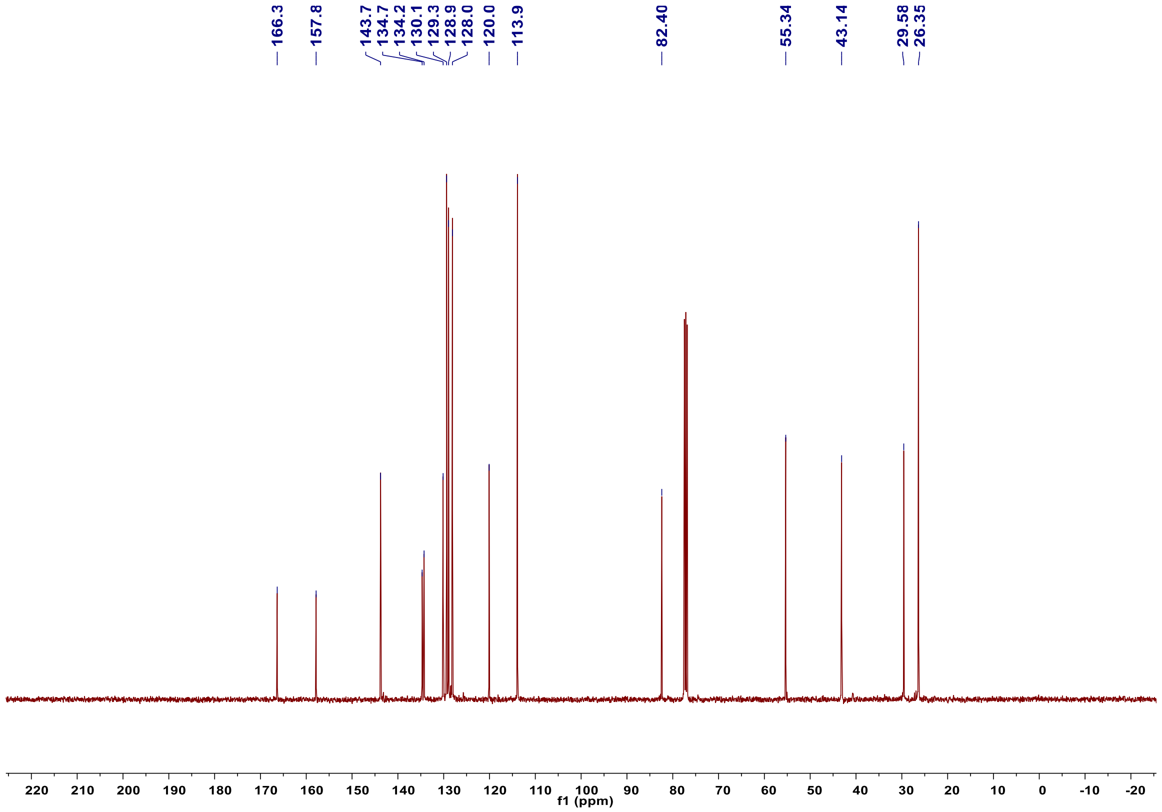

Supplementary Figure 225. ¹³C NMR (100 MHz, CDCl₃) spectrum for 78

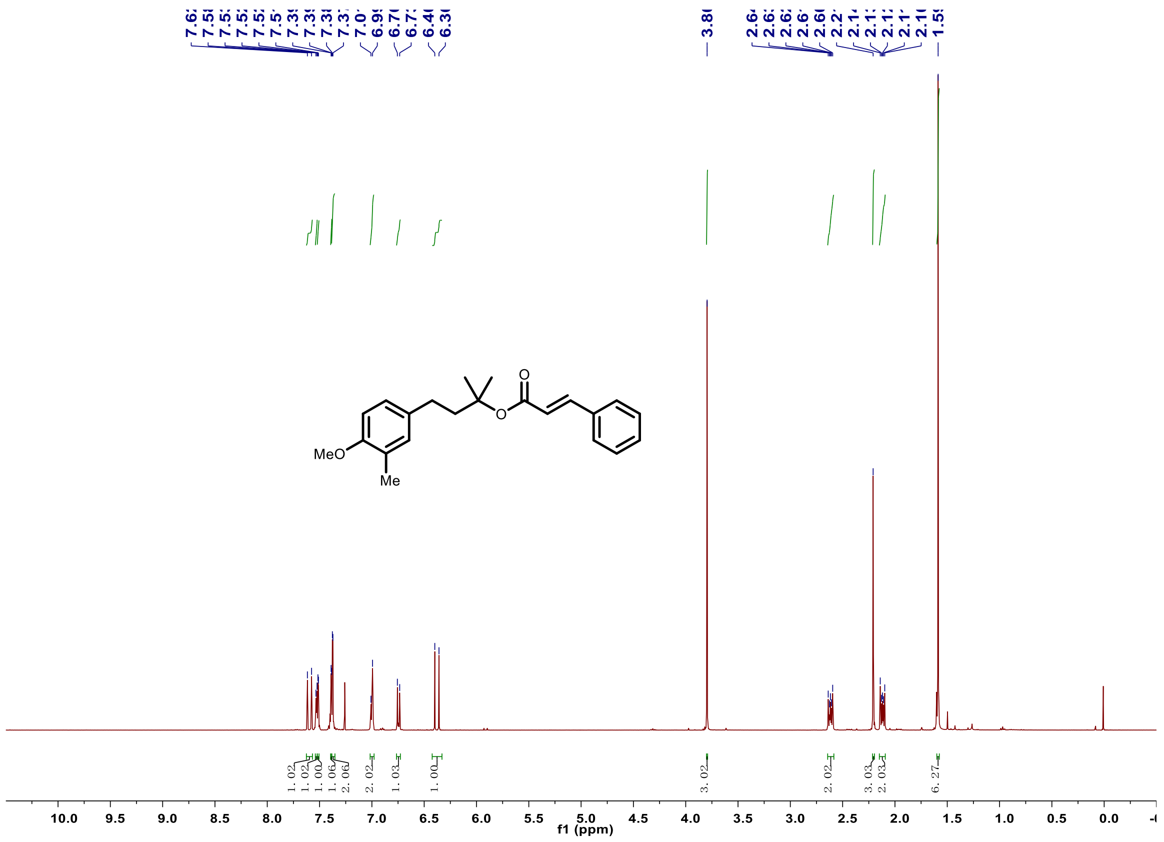

Supplementary Figure 226. ^{1}H NMR (400 MHz, $CDCl_3$) spectrum for 79

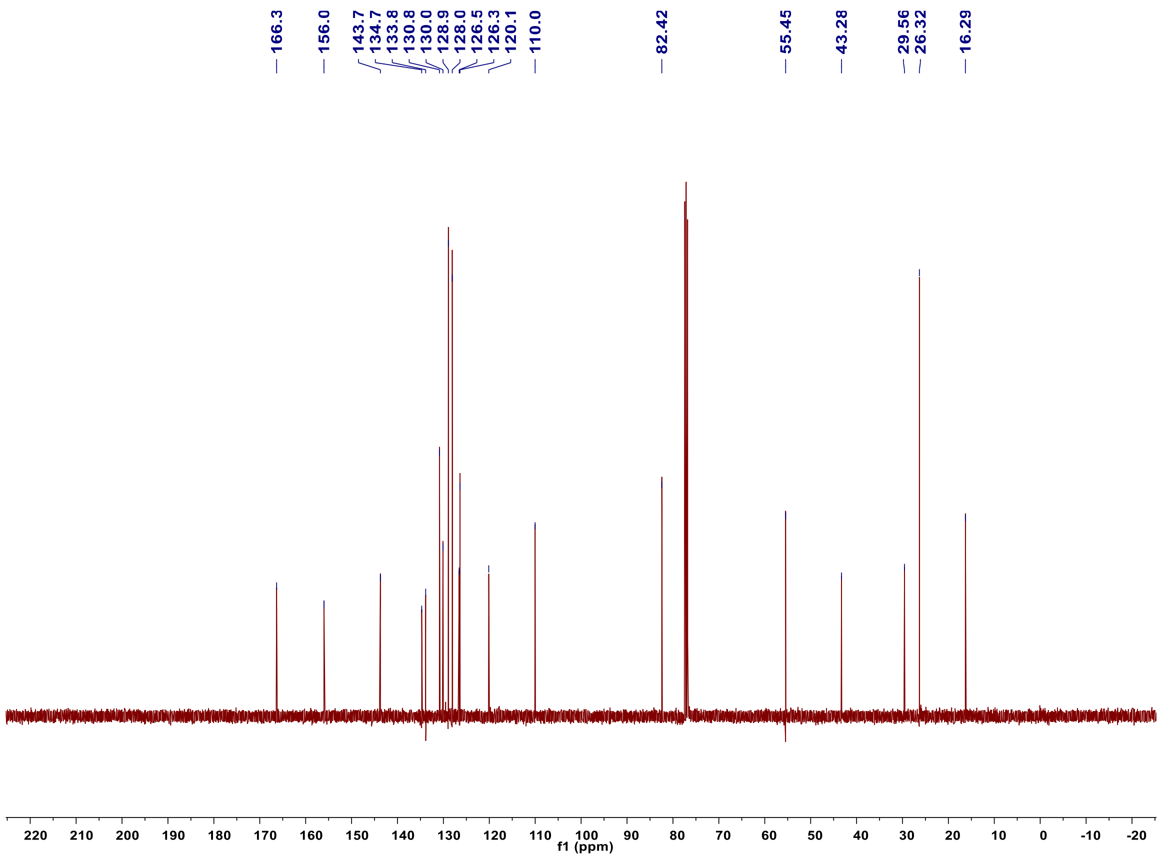

Supplementary Figure 227. ^{13}C NMR (100 MHz, $CDCl_3$) spectrum for 79

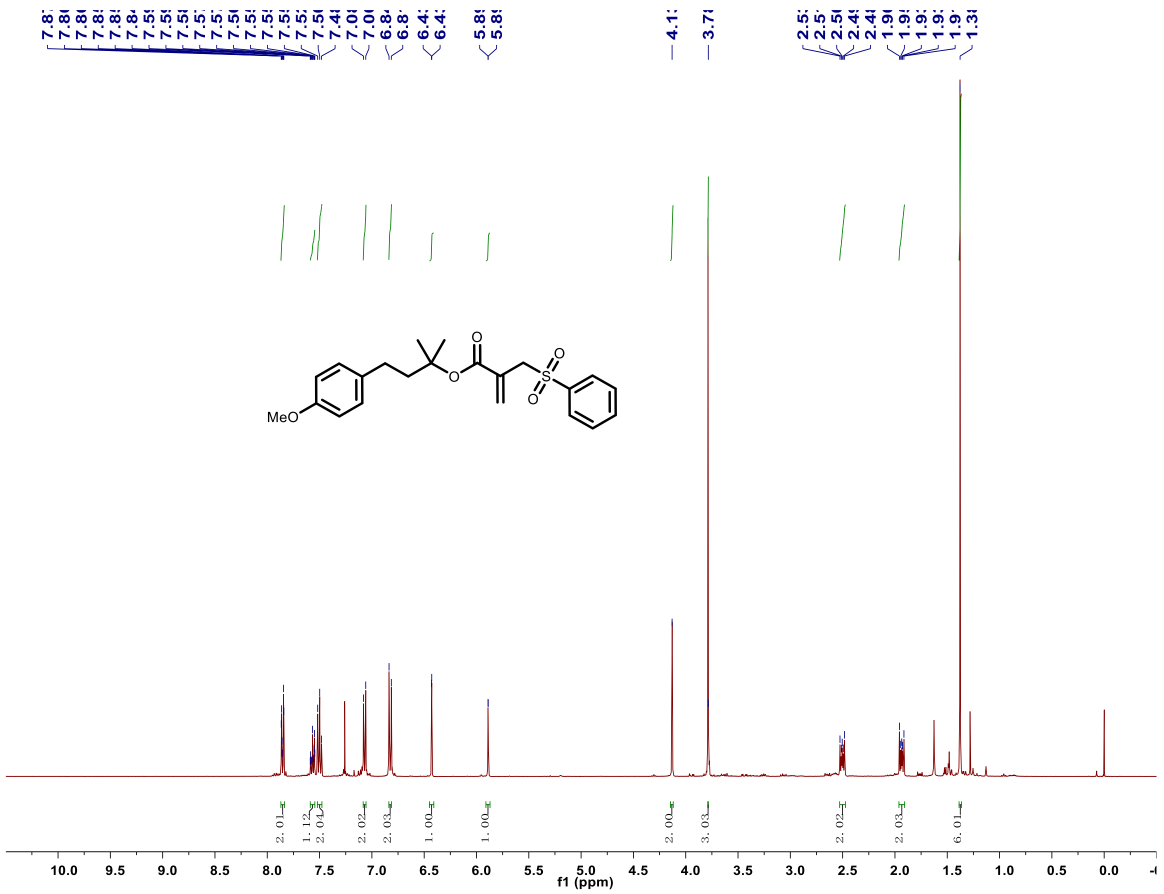

Supplementary Figure 228. ^{1}H NMR (400 MHz, $CDCl_3$) spectrum for **80**

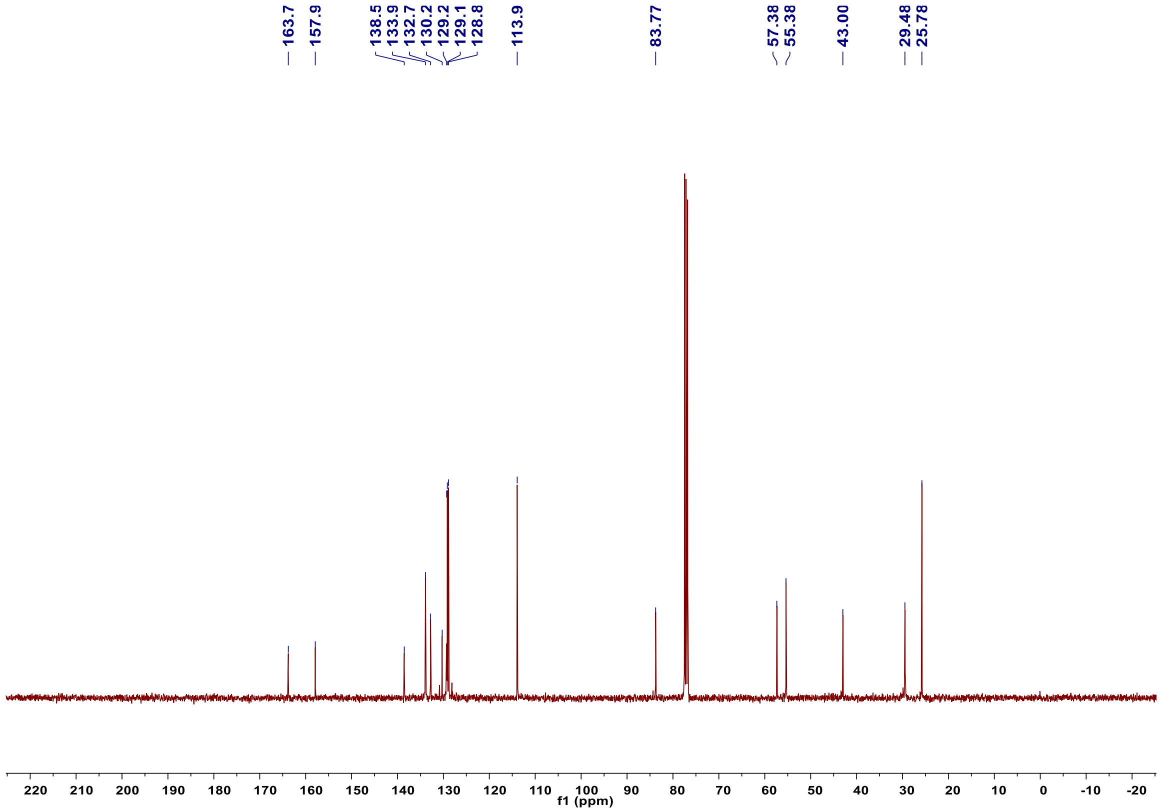

Supplementary Figure 229. ^{13}C NMR (100 MHz, $CDCl_3$) spectrum for **80**

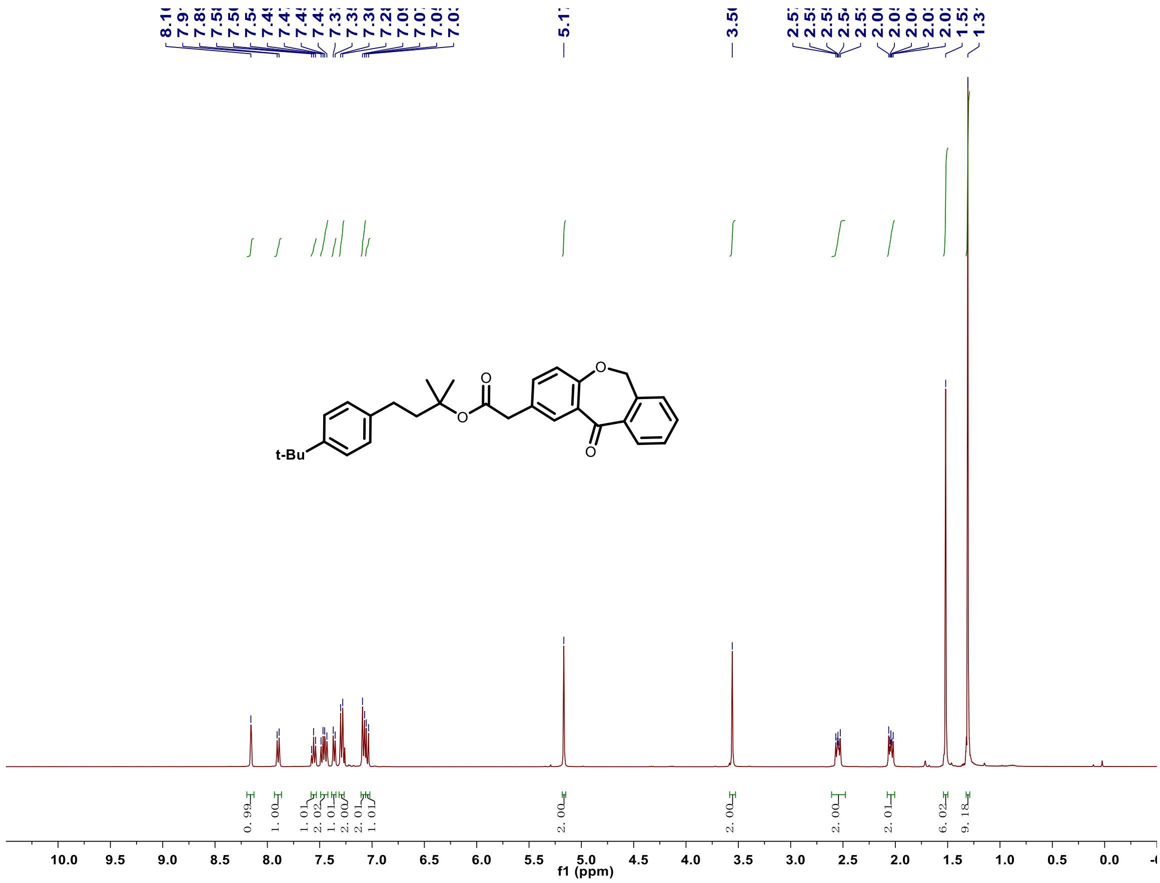

Supplementary Figure 230. ^{1}H NMR (400 MHz, $CDCl_3$) spectrum for 81

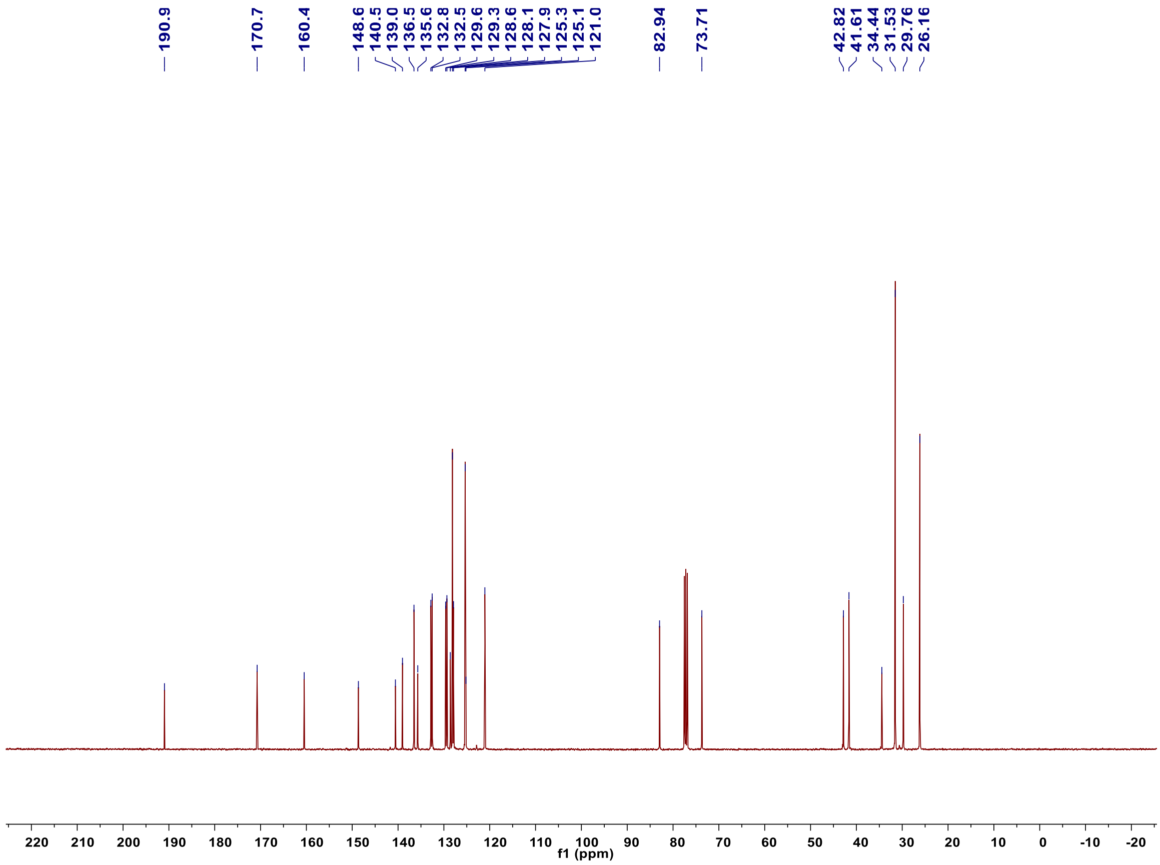

Supplementary Figure 231. ^{13}C NMR (100 MHz, $CDCl_3$) spectrum for 81

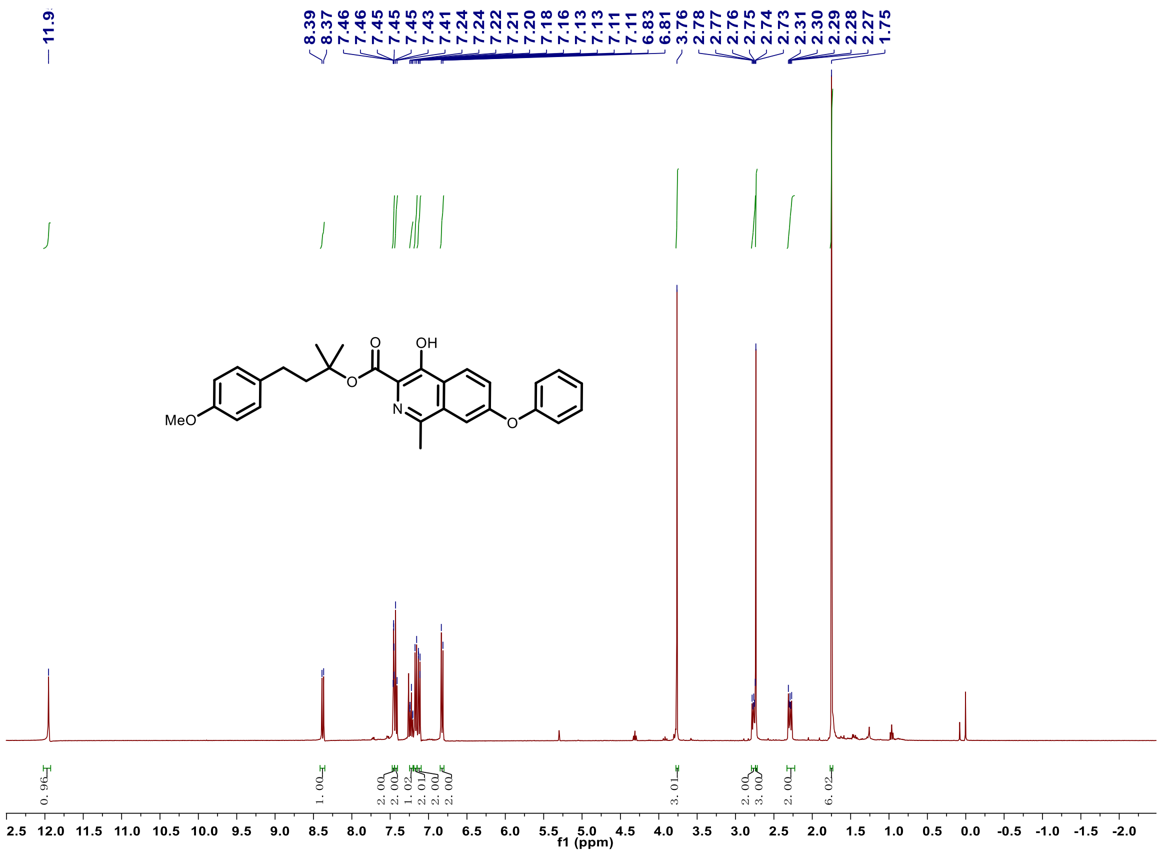

Supplementary Figure 232. ^{1}H NMR (400 MHz, $CDCl_3$) spectrum for 82

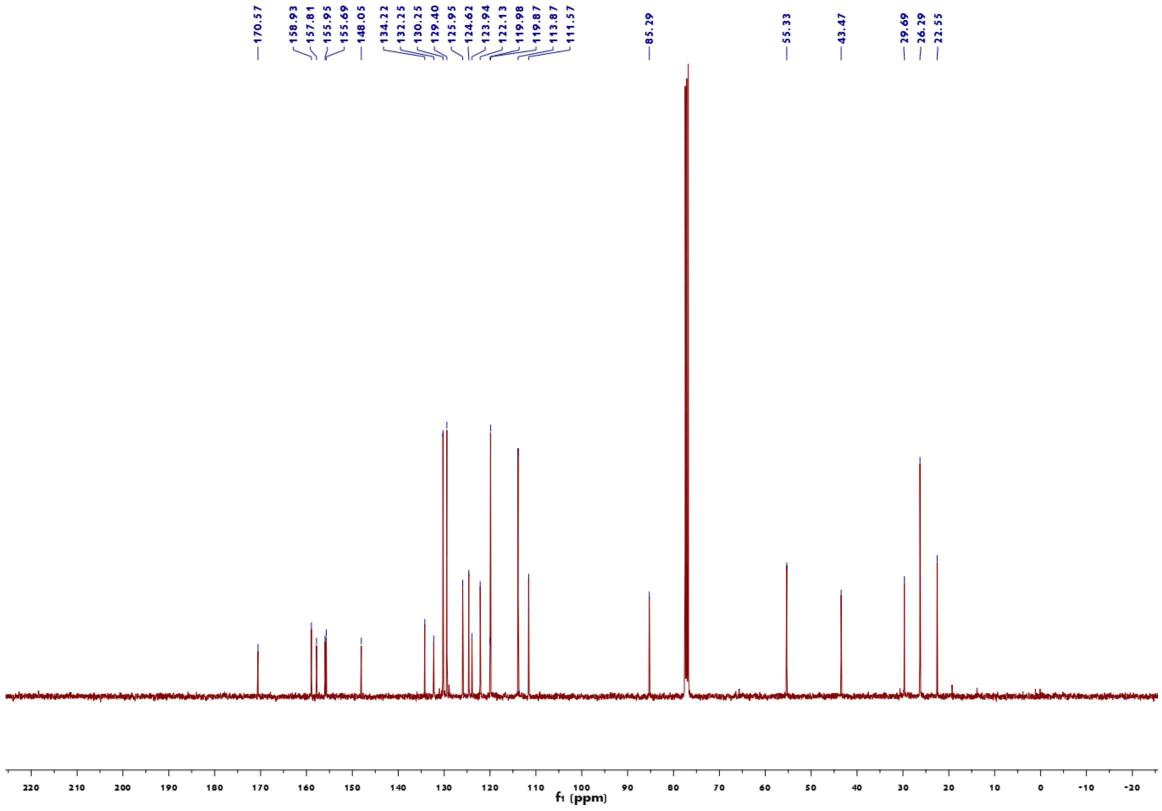

Supplementary Figure 233. ^{13}C NMR (100 MHz, $CDCl_3$) spectrum for 82

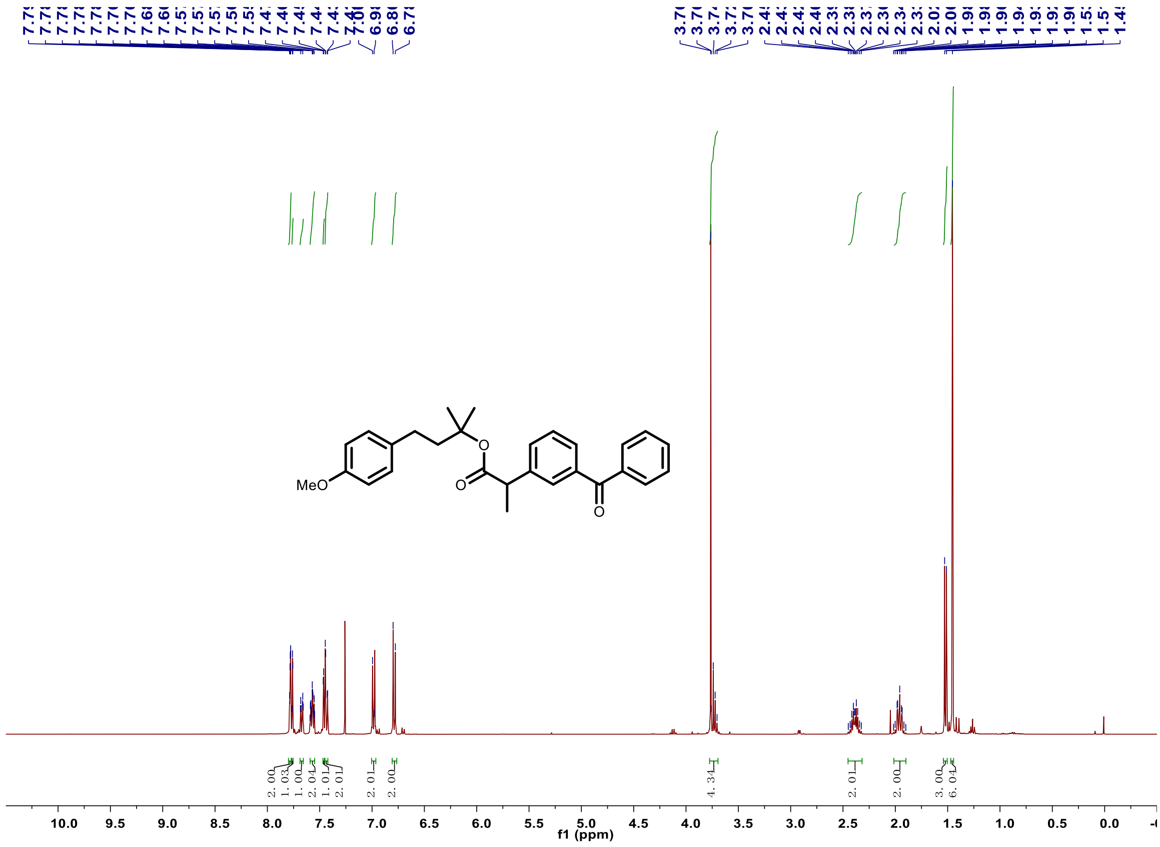

Supplementary Figure 234. ^{1}H NMR (400 MHz, $CDCl_3$) spectrum for 83

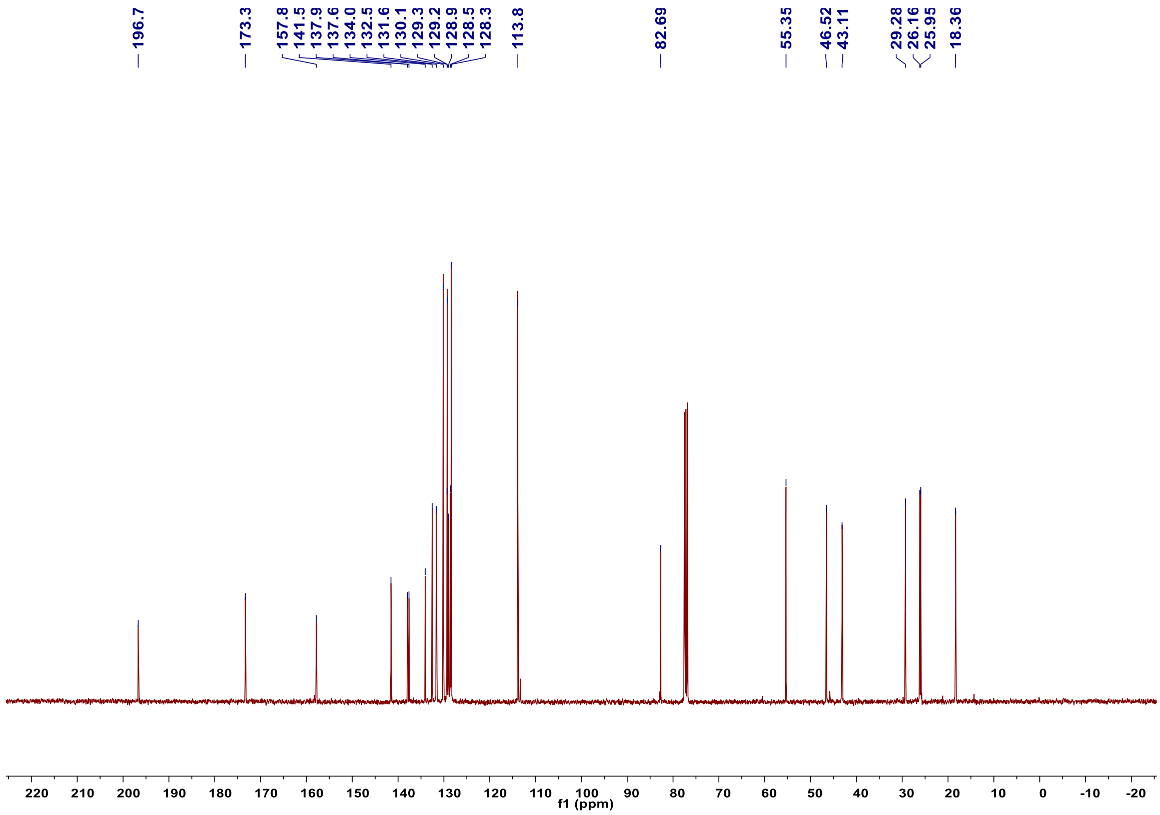

Supplementary Figure 235. ^{13}C NMR (100 MHz, $CDCl_3$) spectrum for 83

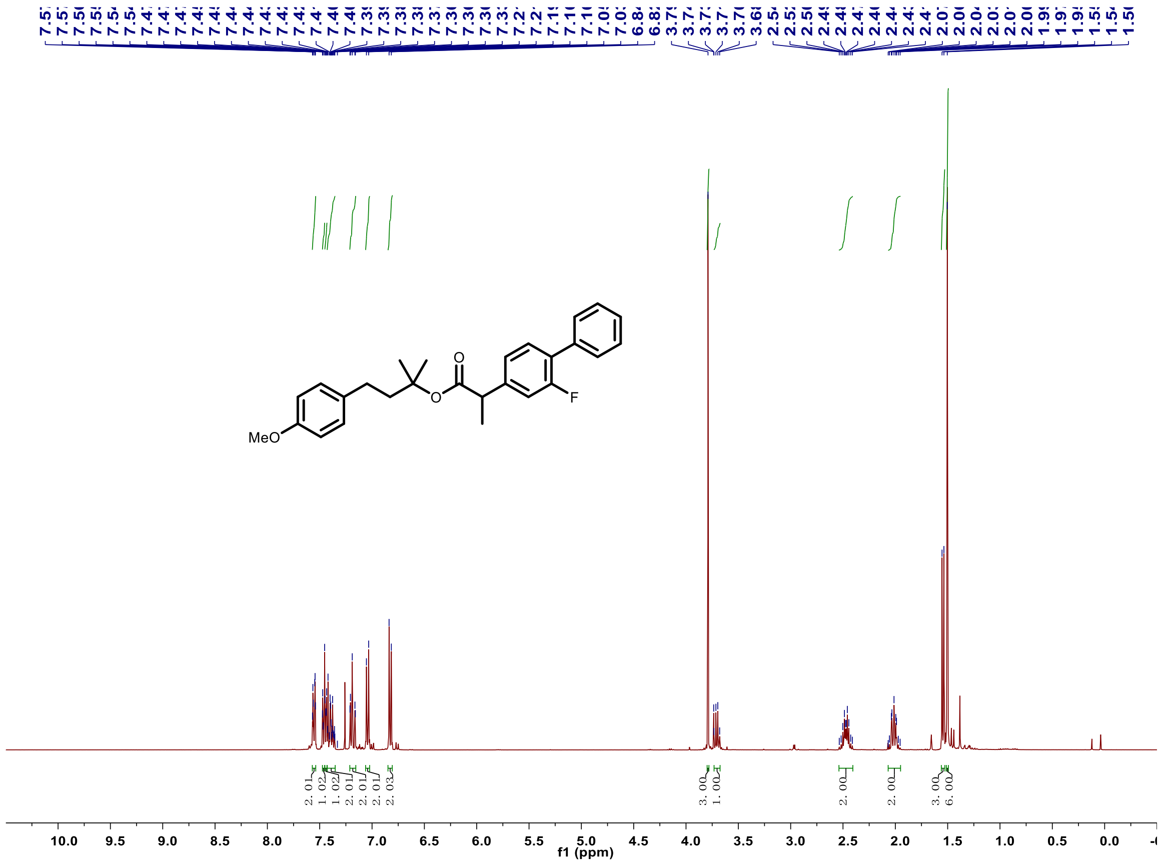

Supplementary Figure 236. ^{1}H NMR (400 MHz, $CDCl_3$) spectrum for 84

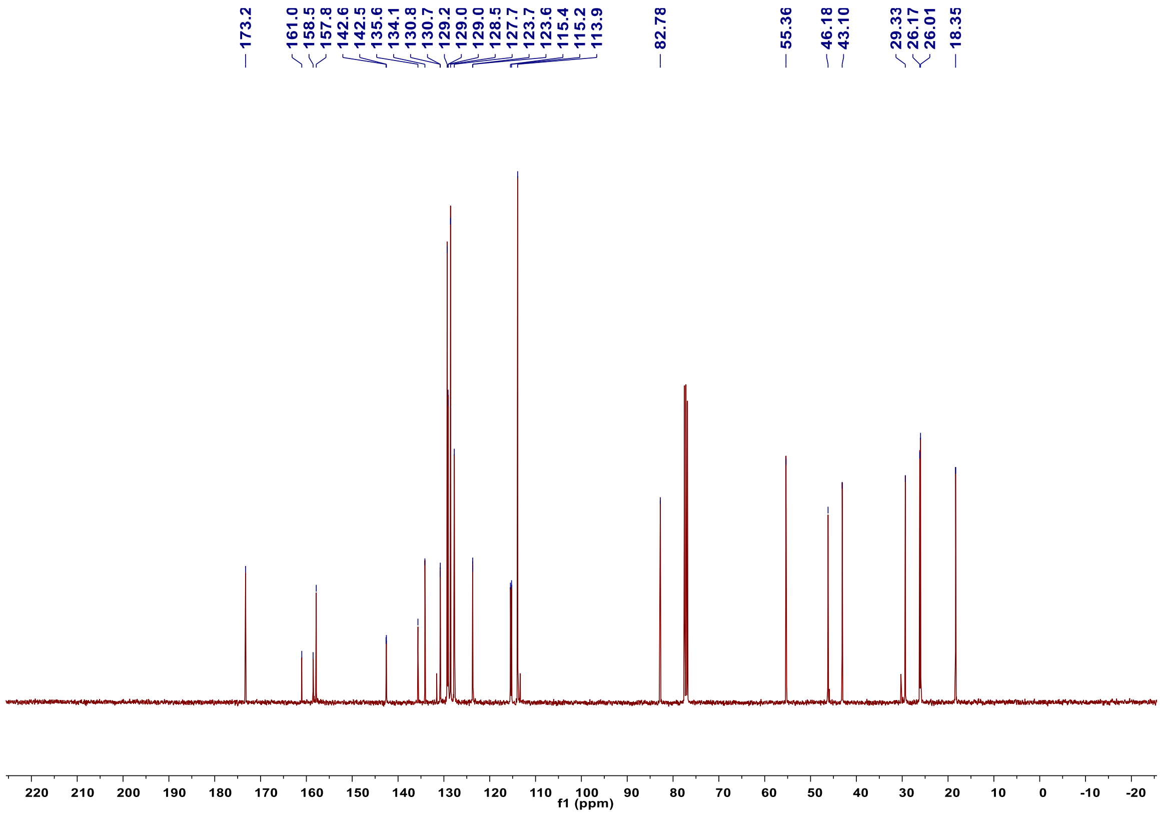

Supplementary Figure 237. ^{13}C NMR (100 MHz, $CDCl_3$) spectrum for 84

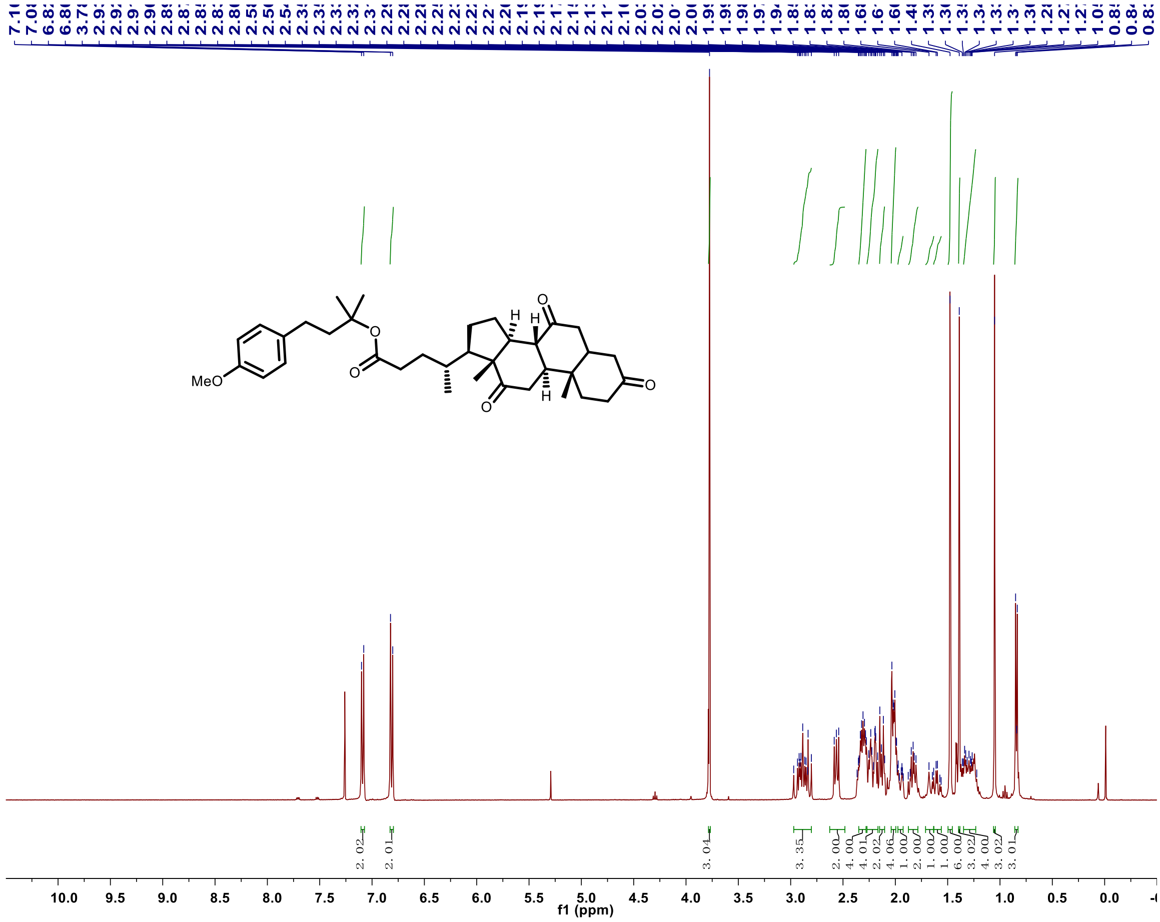

Supplementary Figure 238. ^{1}H NMR (400 MHz, $CDCl_3$) spectrum for 85

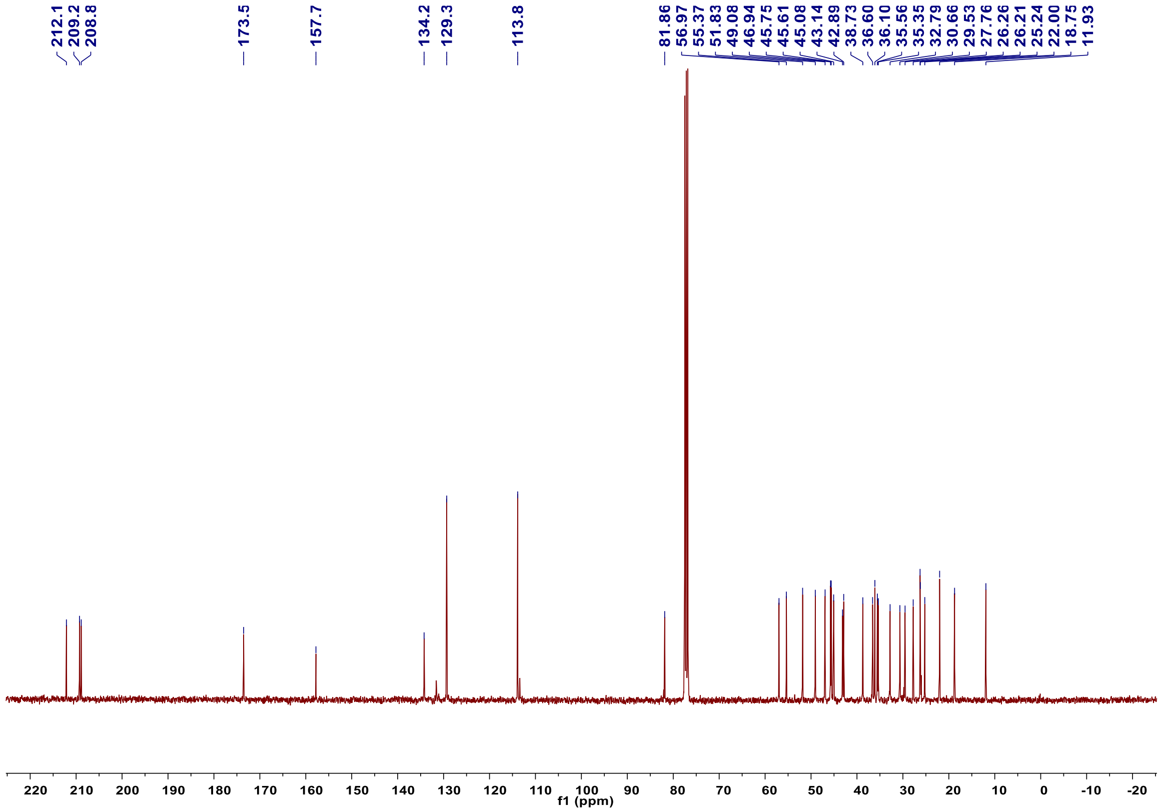

Supplementary Figure 239. ^{13}C NMR (100 MHz, $CDCl_3$) spectrum for 85

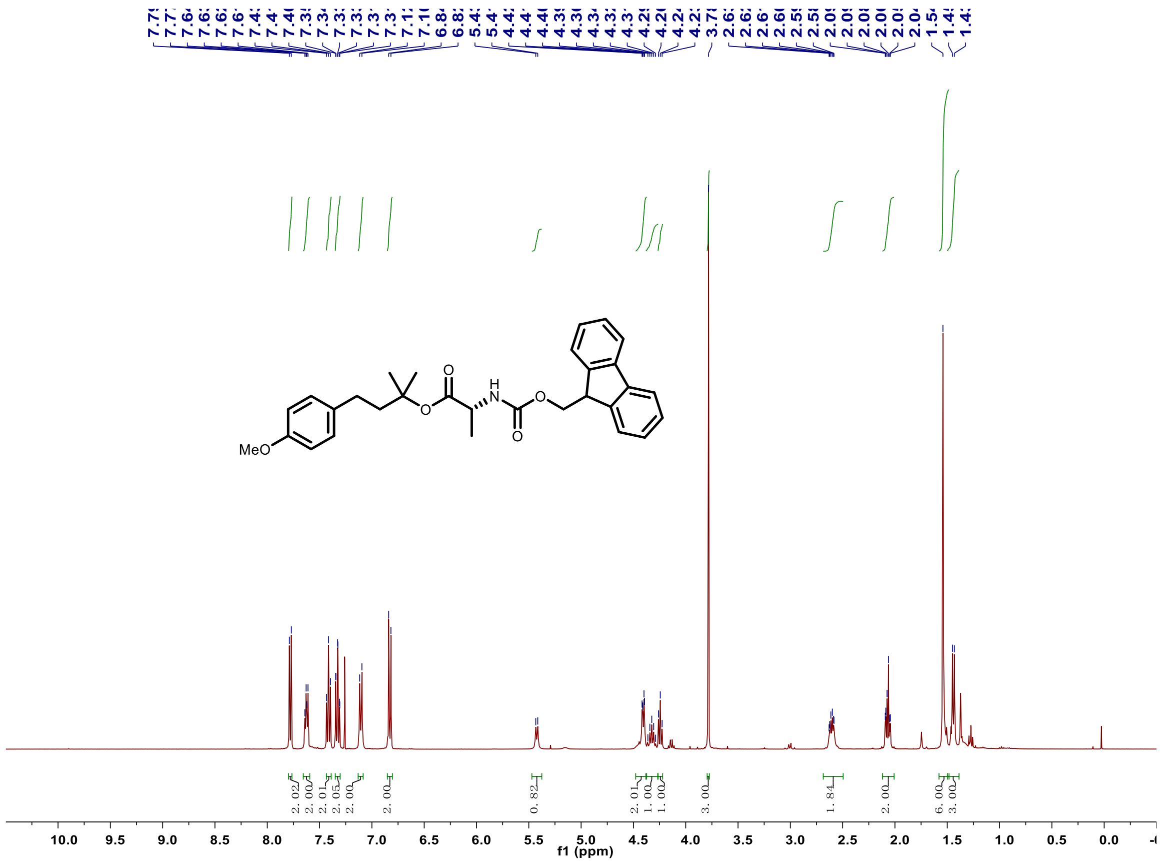

Supplementary Figure 240. ^{1}H NMR (400 MHz, $CDCl_3$) spectrum for 86

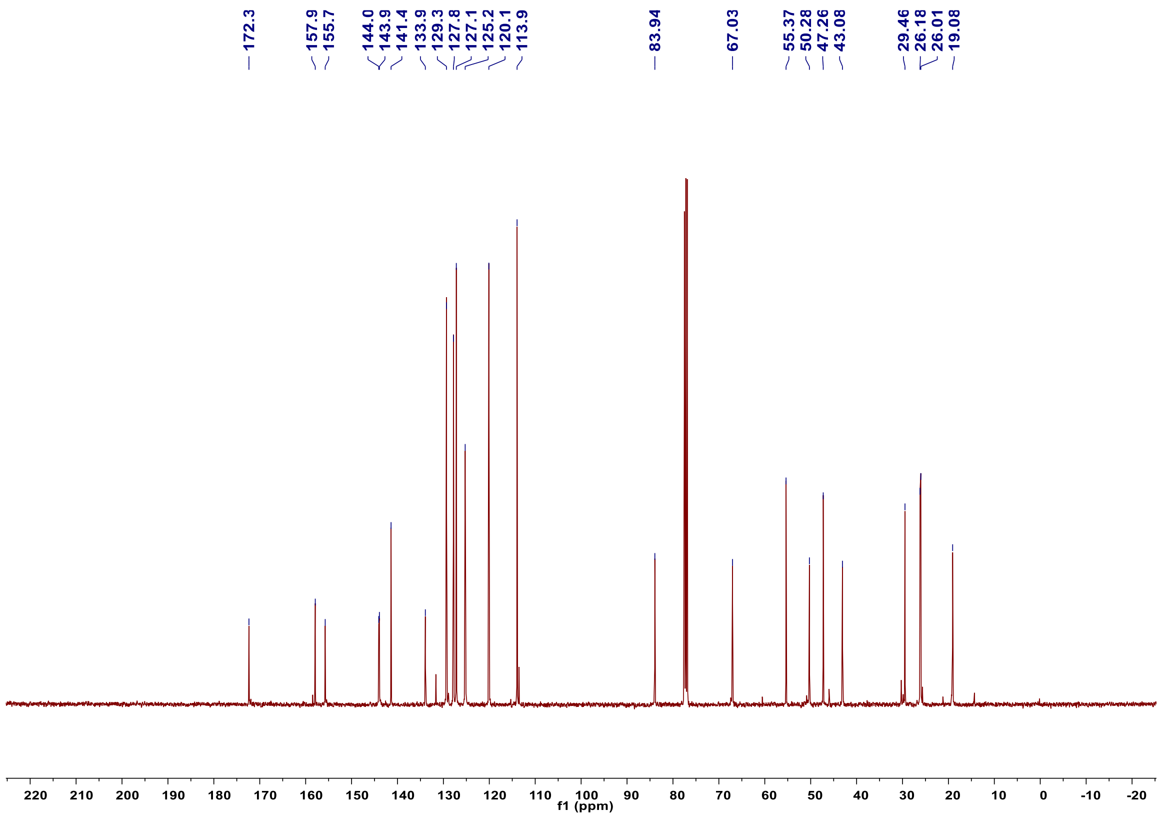

Supplementary Figure 241. ^{13}C NMR (100 MHz, $CDCl_3$) spectrum for 86

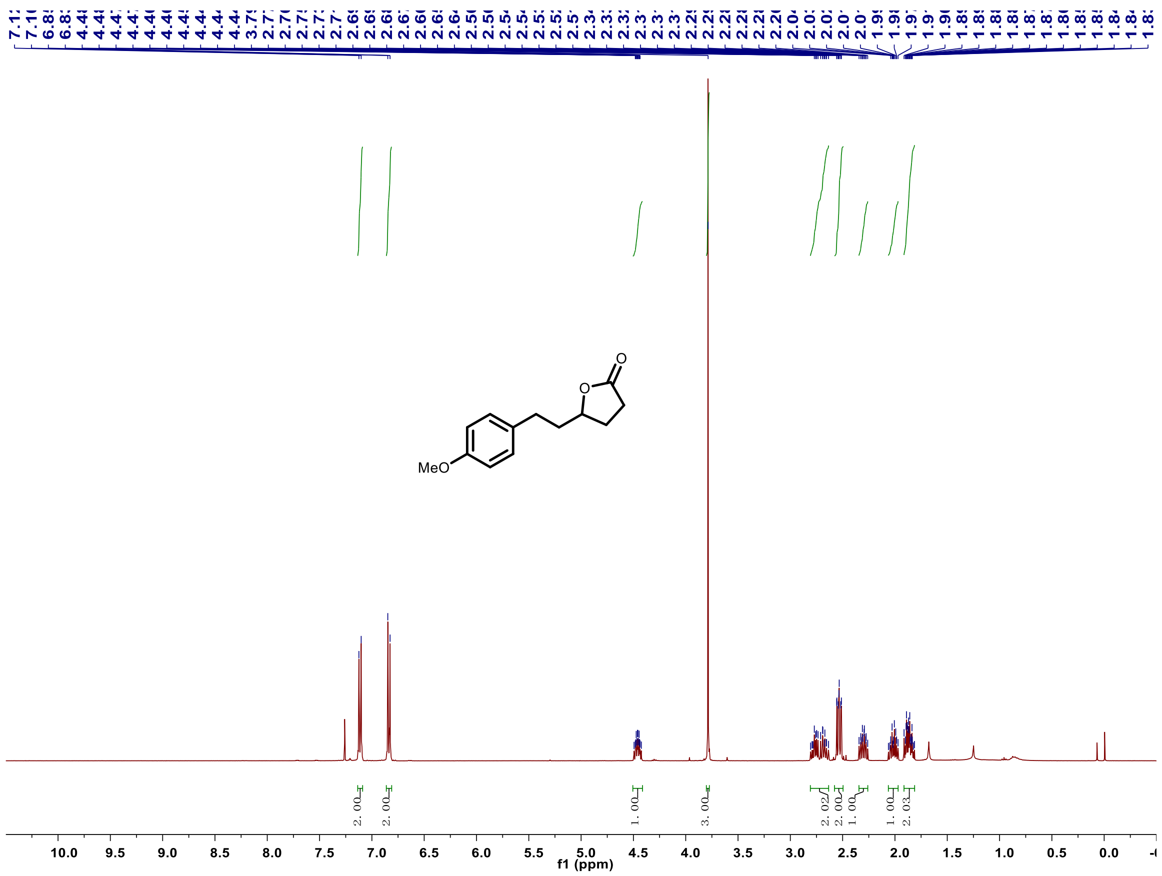

Supplementary Figure 242. ^{1}H NMR (400 MHz, $CDCl_3$) spectrum for 87

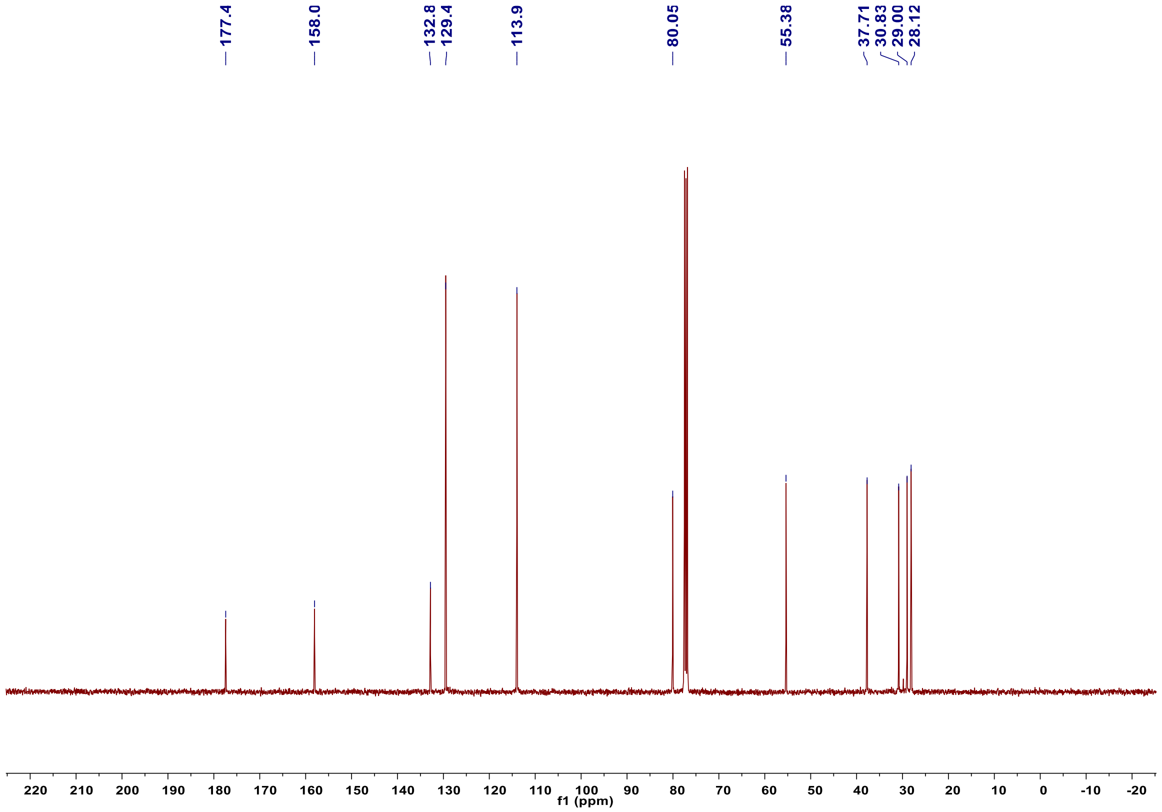

Supplementary Figure 243. ^{13}C NMR (100 MHz, $CDCl_3$) spectrum for 87

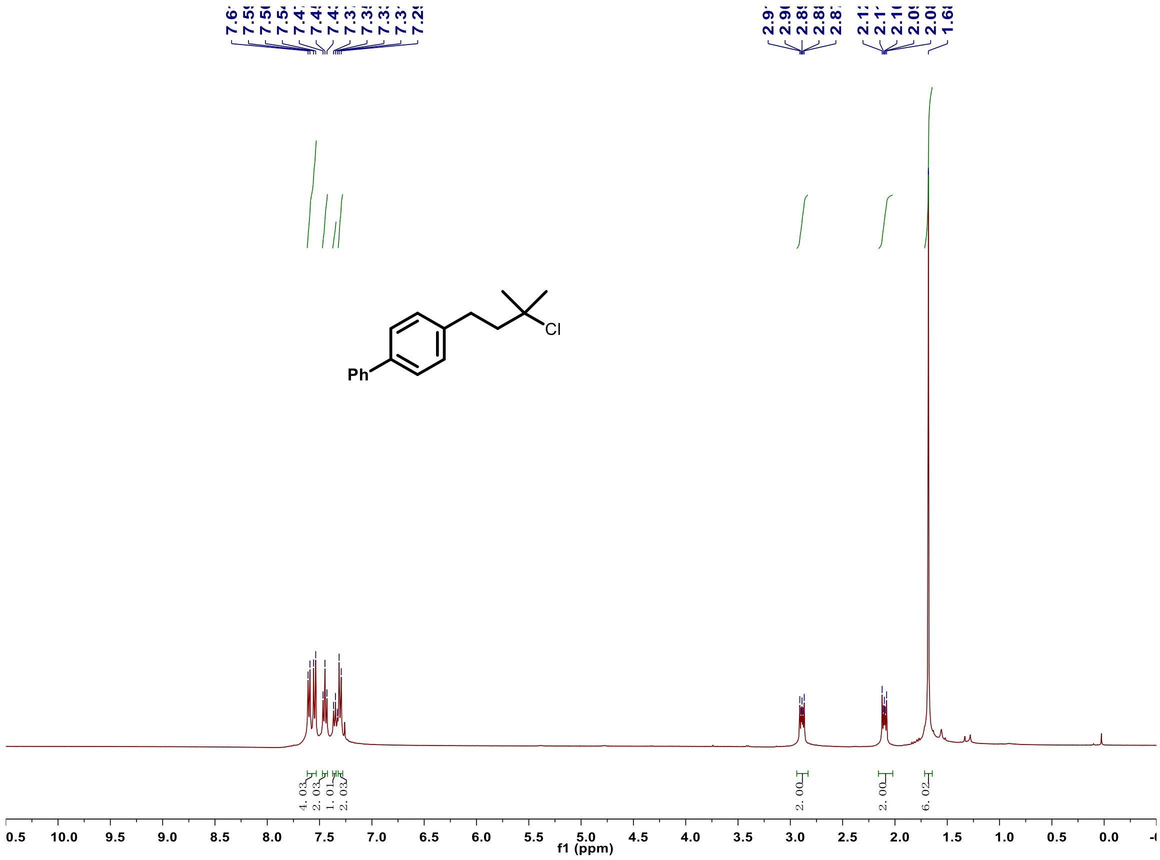

Supplementary Figure 244. ^{1}H **NMR (400 MHz,** $CDCl_3$**) spectrum for 99**

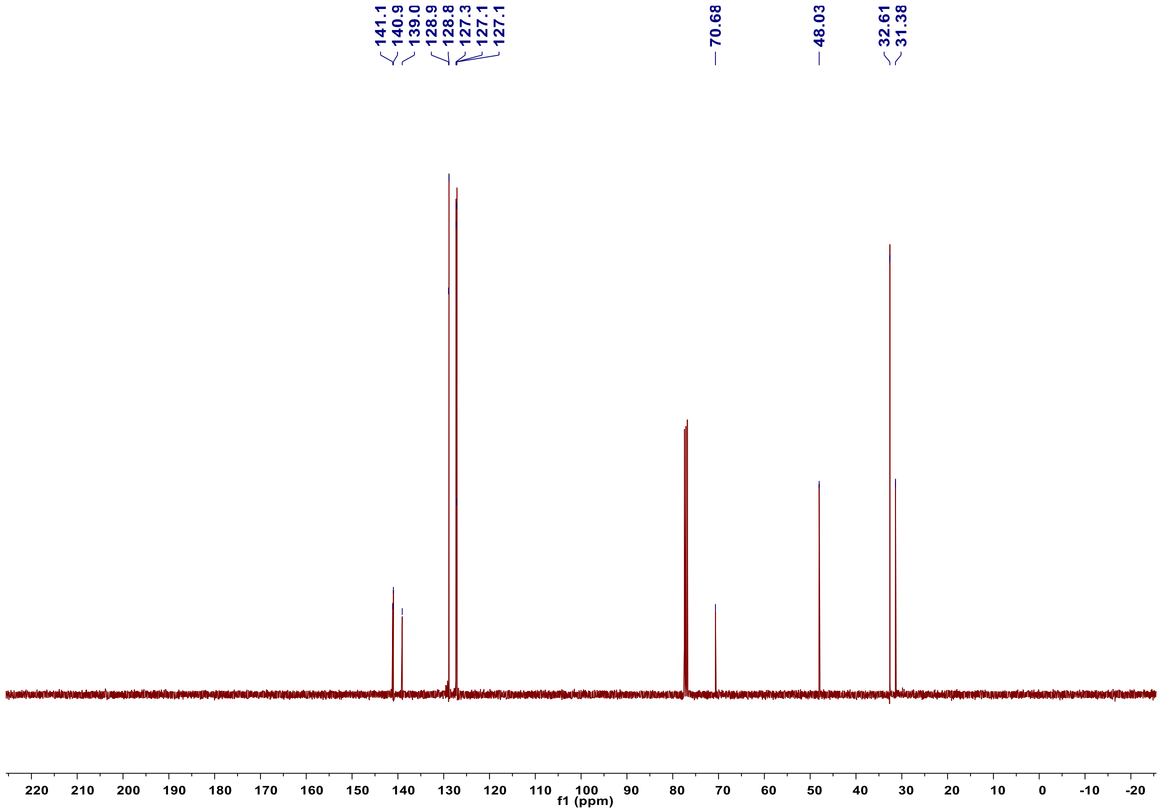

Supplementary Figure 245. ^{13}C **NMR (100 MHz,** $CDCl_3$**) spectrum for 99**

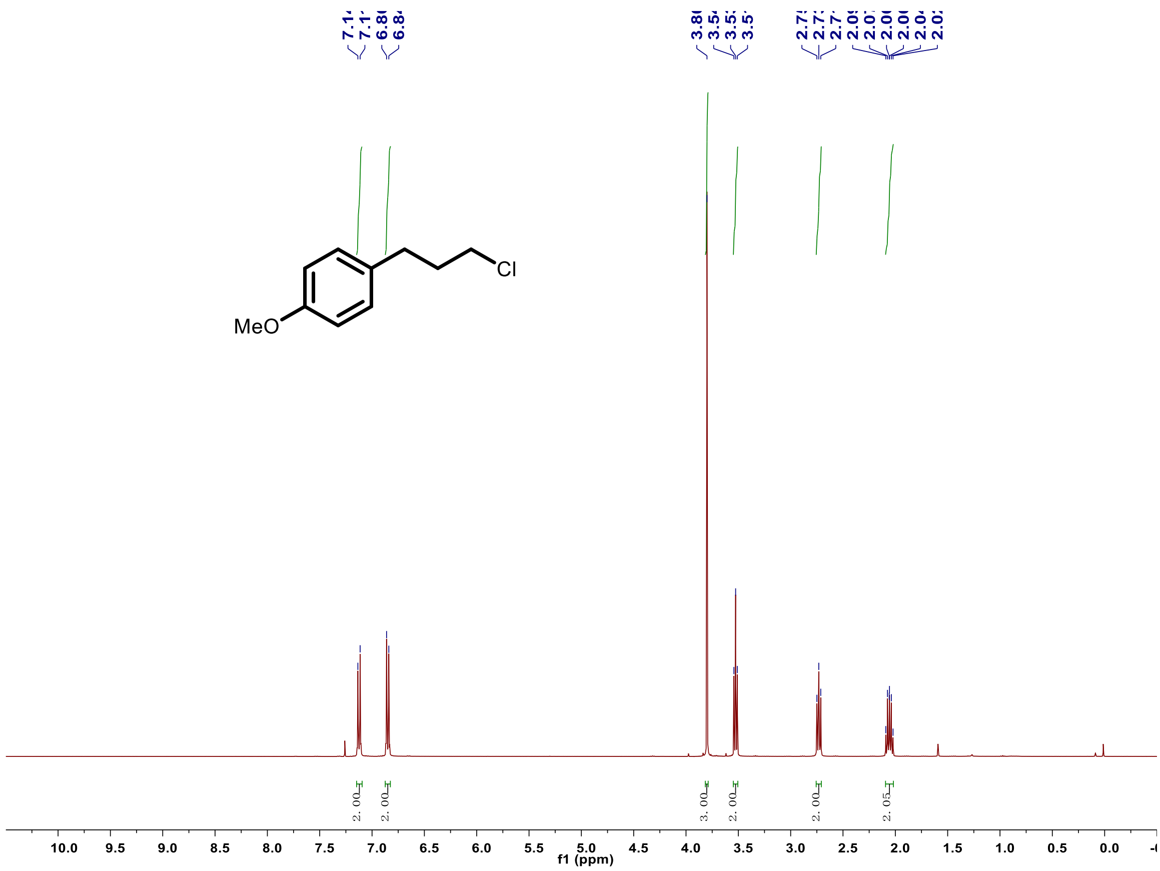

Supplementary Figure 246. **^{1}H NMR (400 MHz, CDCl$_3$) spectrum for 100**

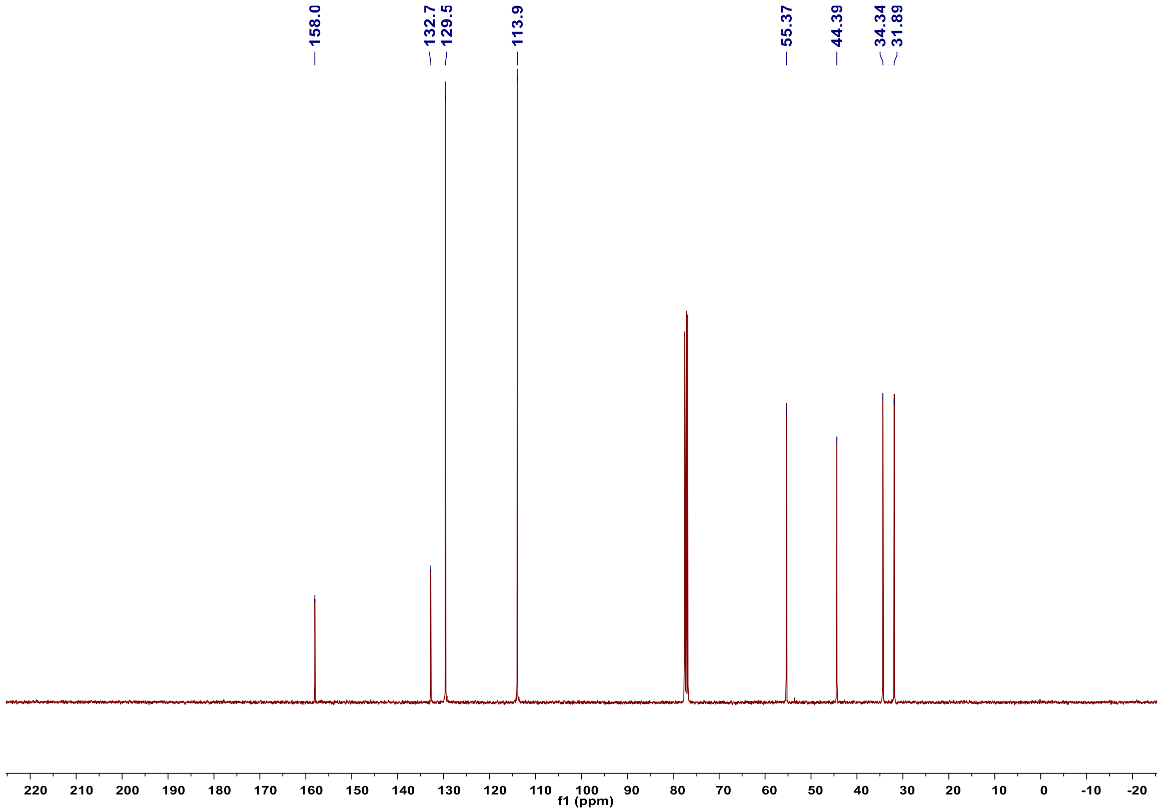

Supplementary Figure 247. **^{13}C NMR (100 MHz, CDCl$_3$) spectrum for 100**

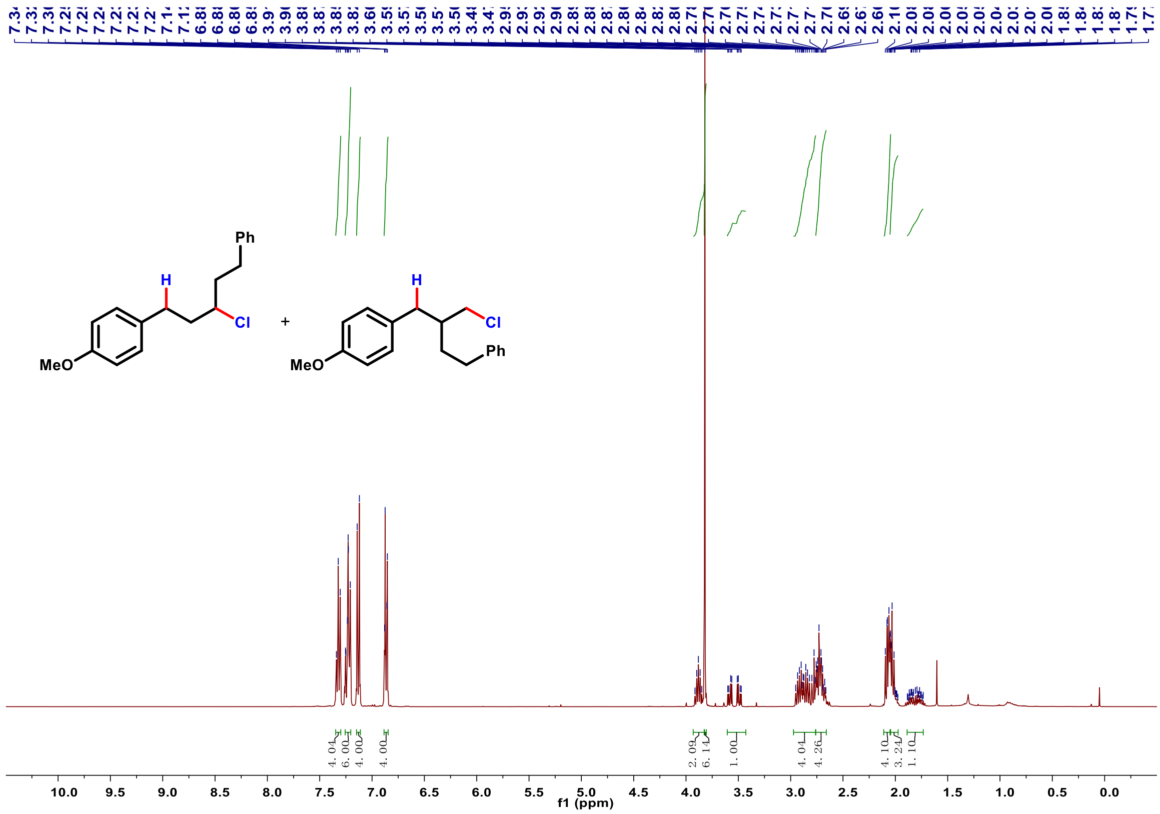

Supplementary Figure 248. ^{1}H NMR (400 MHz, $CDCl_3$) spectrum for 104 & 105

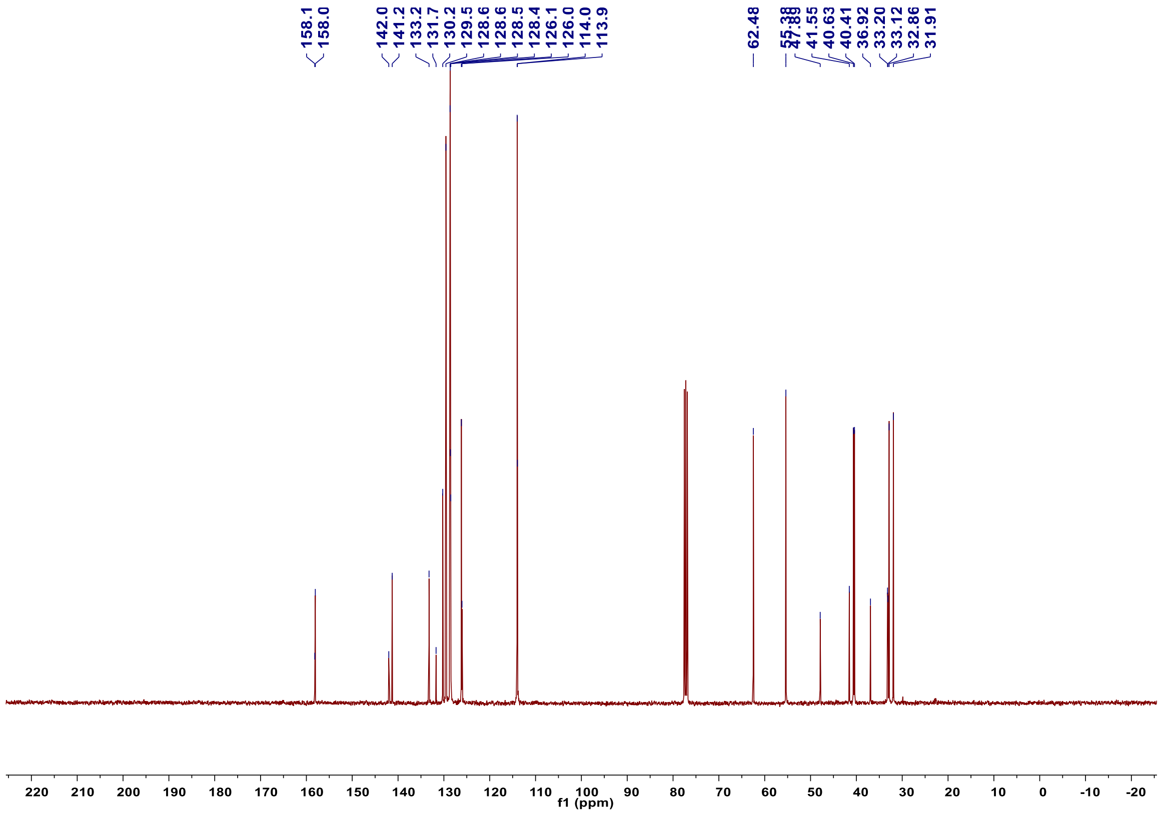

Supplementary Figure 249. ^{13}C NMR (100 MHz, $CDCl_3$) spectrum for 104 & 105

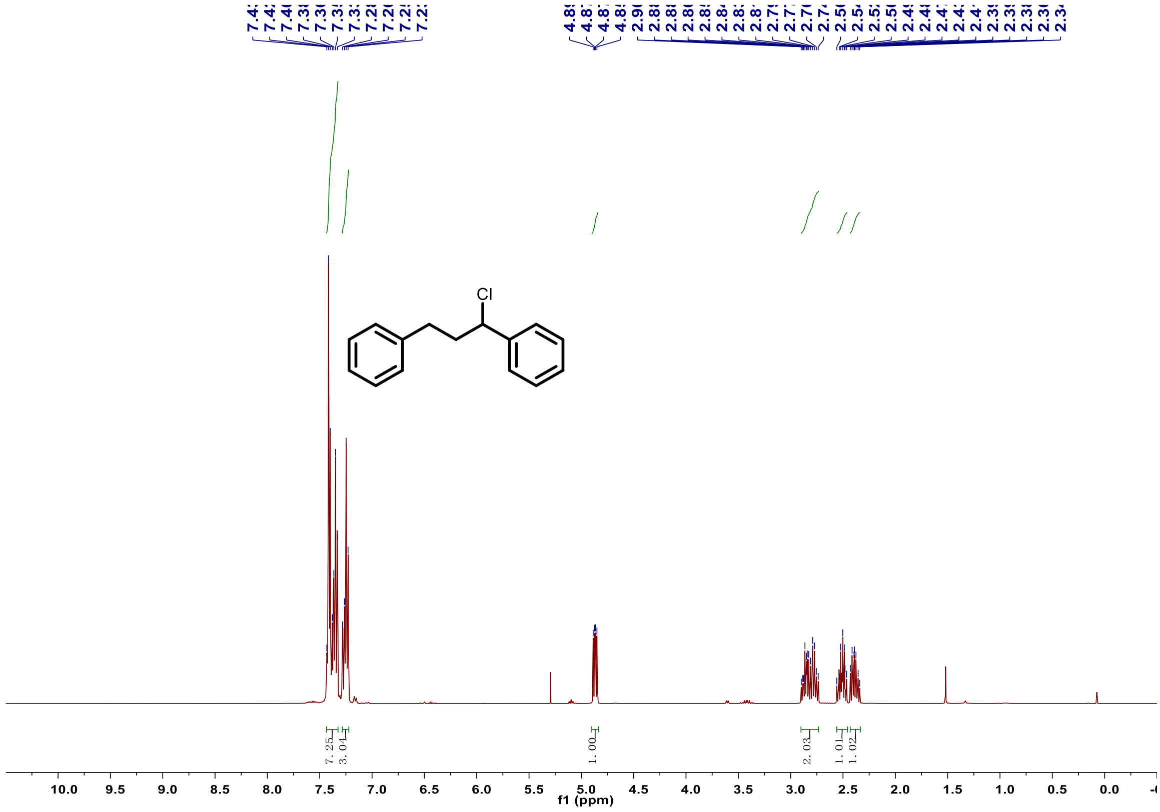

Supplementary Figure 250. ^{1}H NMR (400 MHz, $CDCl_3$) spectrum for 101

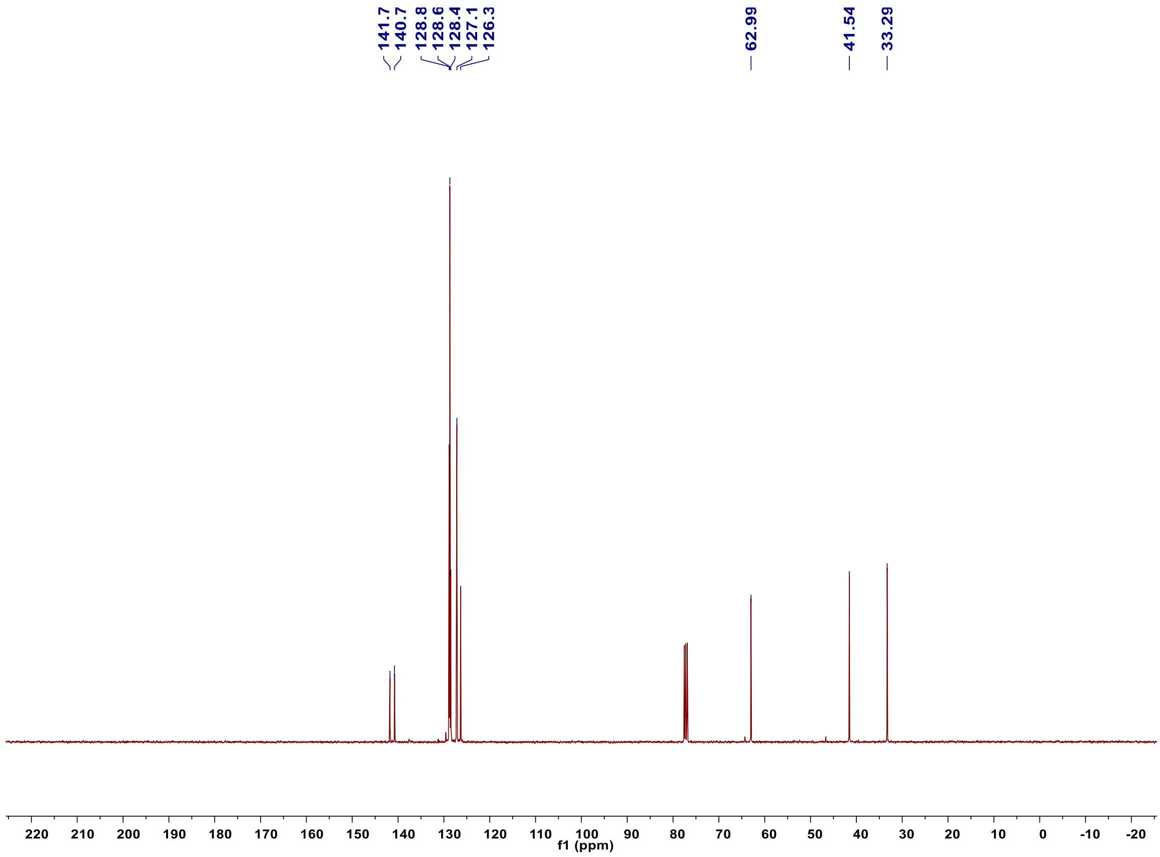

Supplementary Figure 251. ^{13}C NMR (100 MHz, $CDCl_3$) spectrum for 101

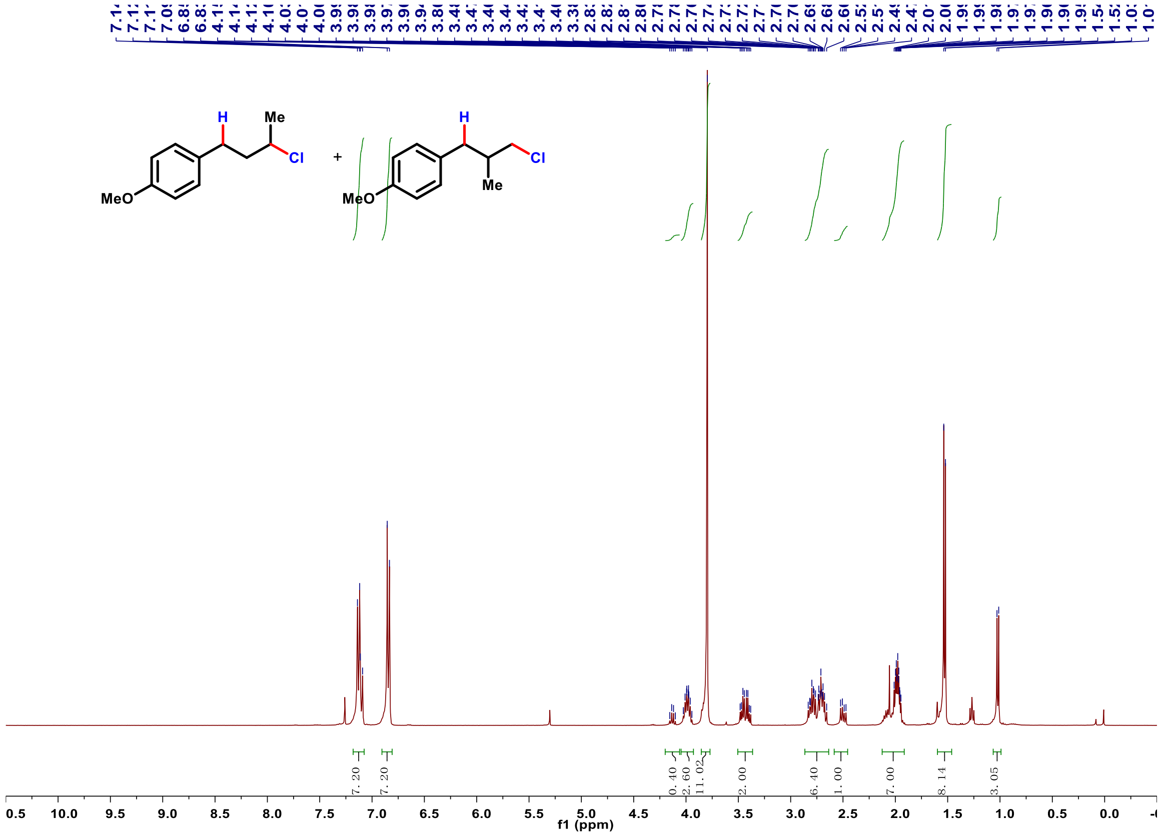

Supplementary Figure 252. **^{1}H NMR (400 MHz, $CDCl_3$) spectrum for 106 & 107**

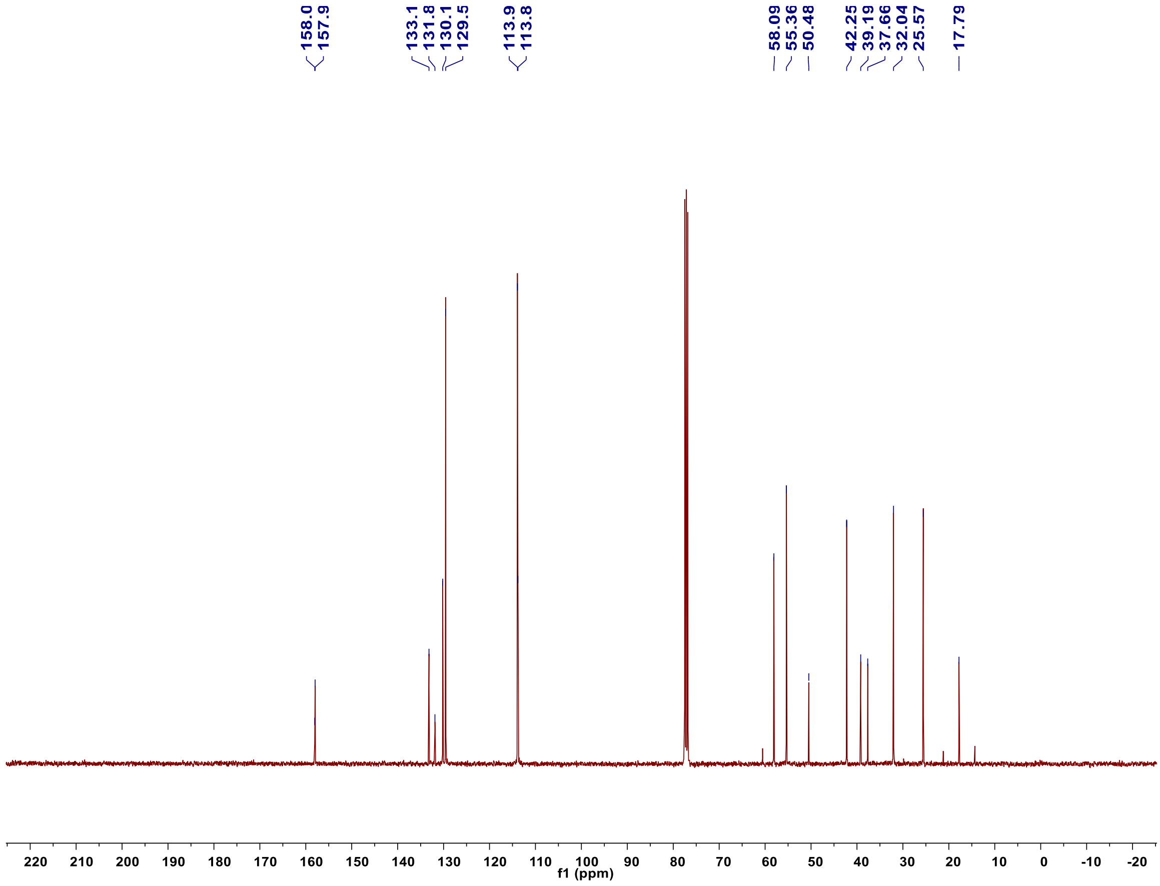

Supplementary Figure 253. **^{13}C NMR (100 MHz, $CDCl_3$) spectrum for 106 & 107**

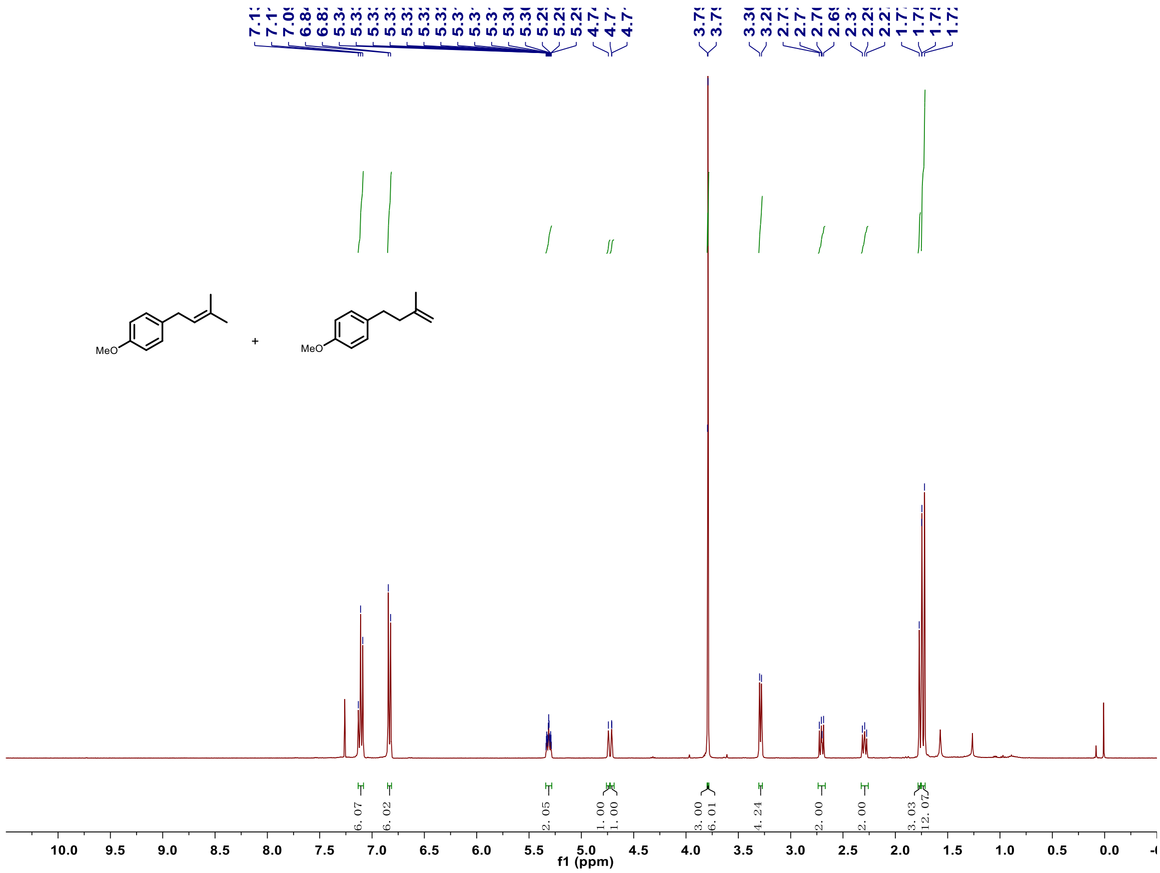

Supplementary Figure 254. ^{1}H **NMR (400 MHz, $CDCl_3$) spectrum for 109 & 110**

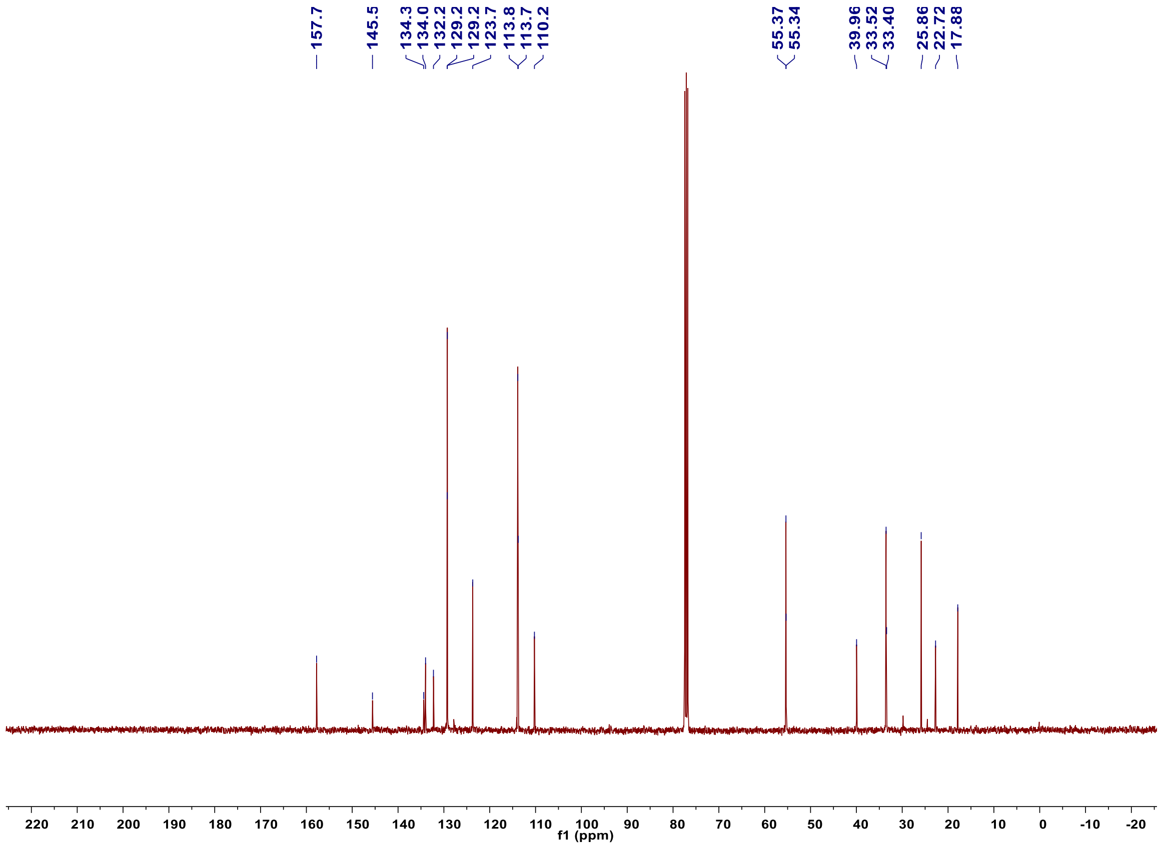

Supplementary Figure 255. ^{13}C **NMR (100 MHz, $CDCl_3$) spectrum for 109 & 110**

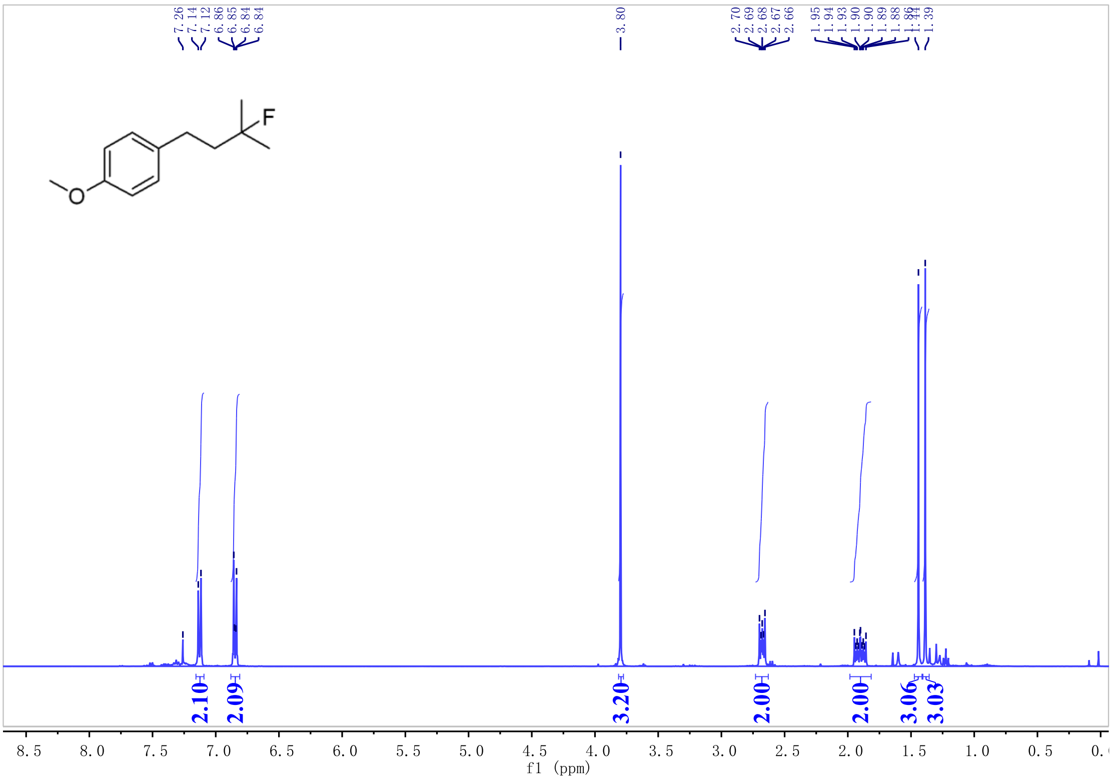

Supplementary Figure 256. ^{1}H NMR (400 MHz, $CDCl_3$) spectrum for 102

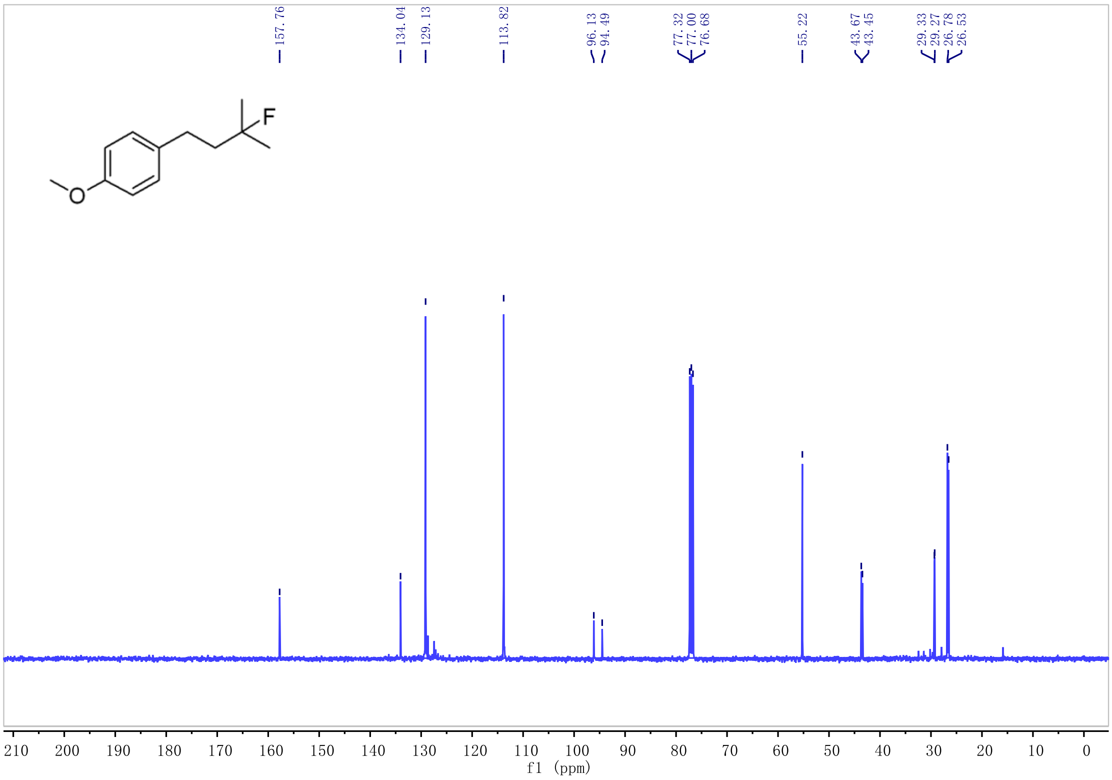

Supplementary Figure 257. ^{13}C NMR (100 MHz, $CDCl_3$) spectrum for 102

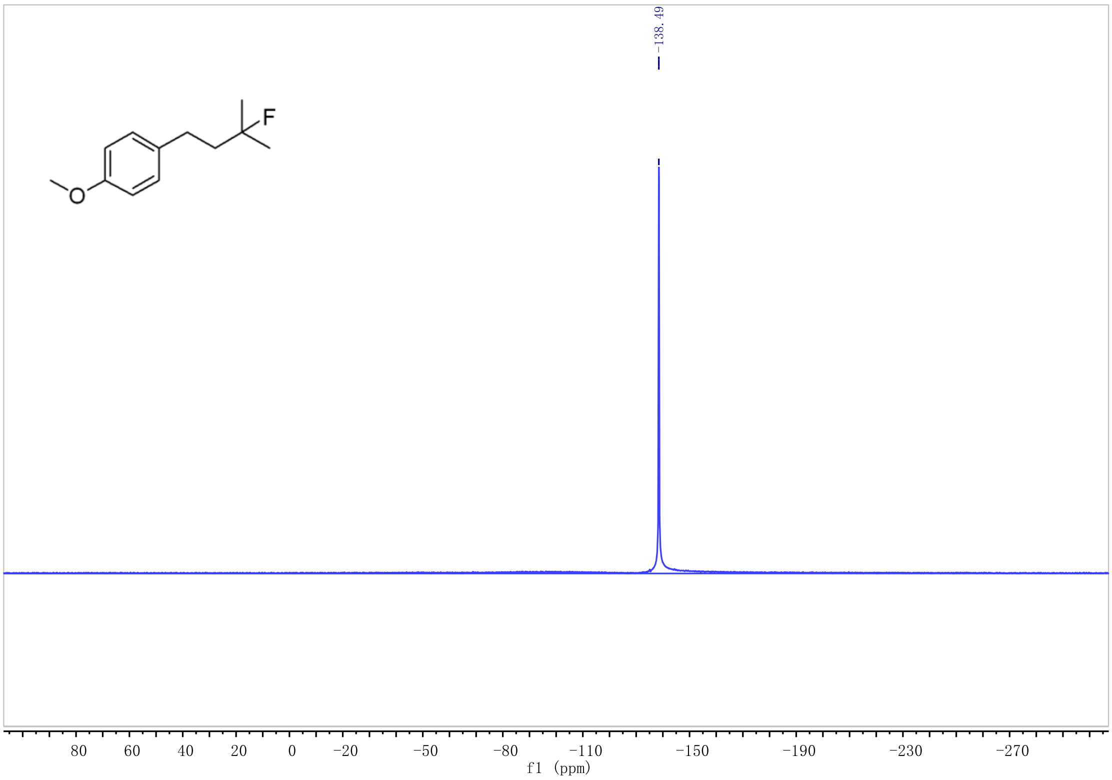

Supplementary Figure 258. **^{19}F NMR (377 MHz, CDCl$_3$) spectrum for 102**

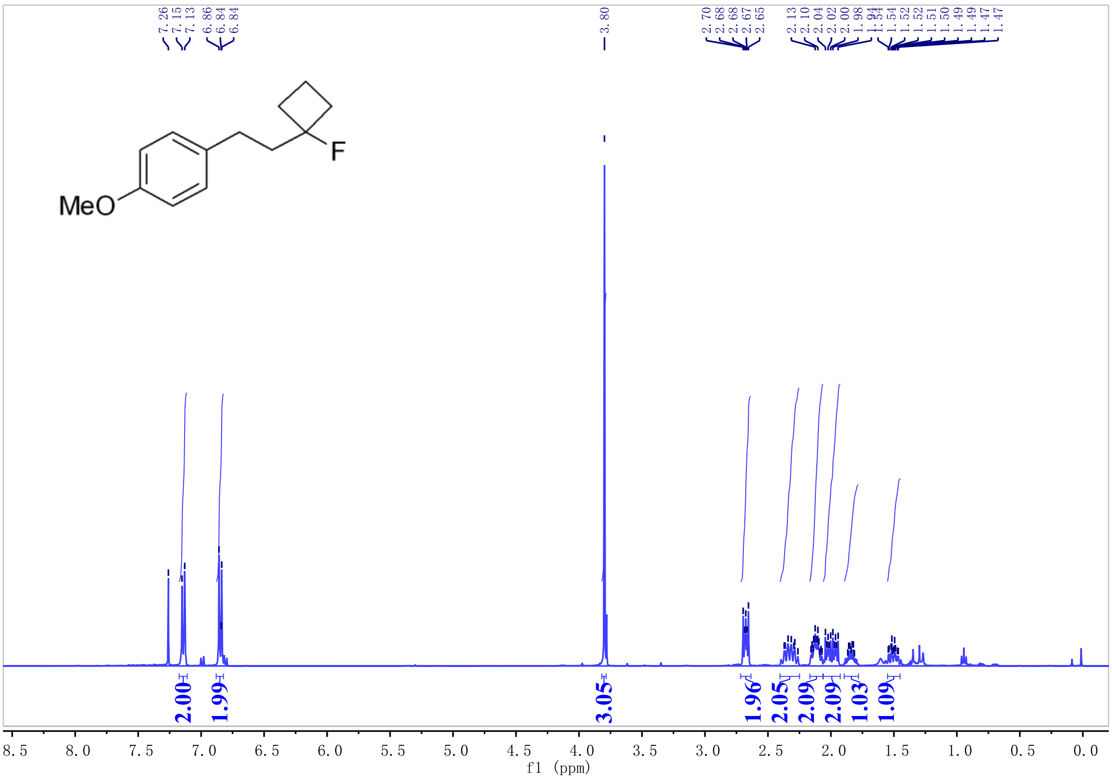

Supplementary Figure 259. **^{1}H NMR (400 MHz, CDCl$_3$) spectrum for 103**

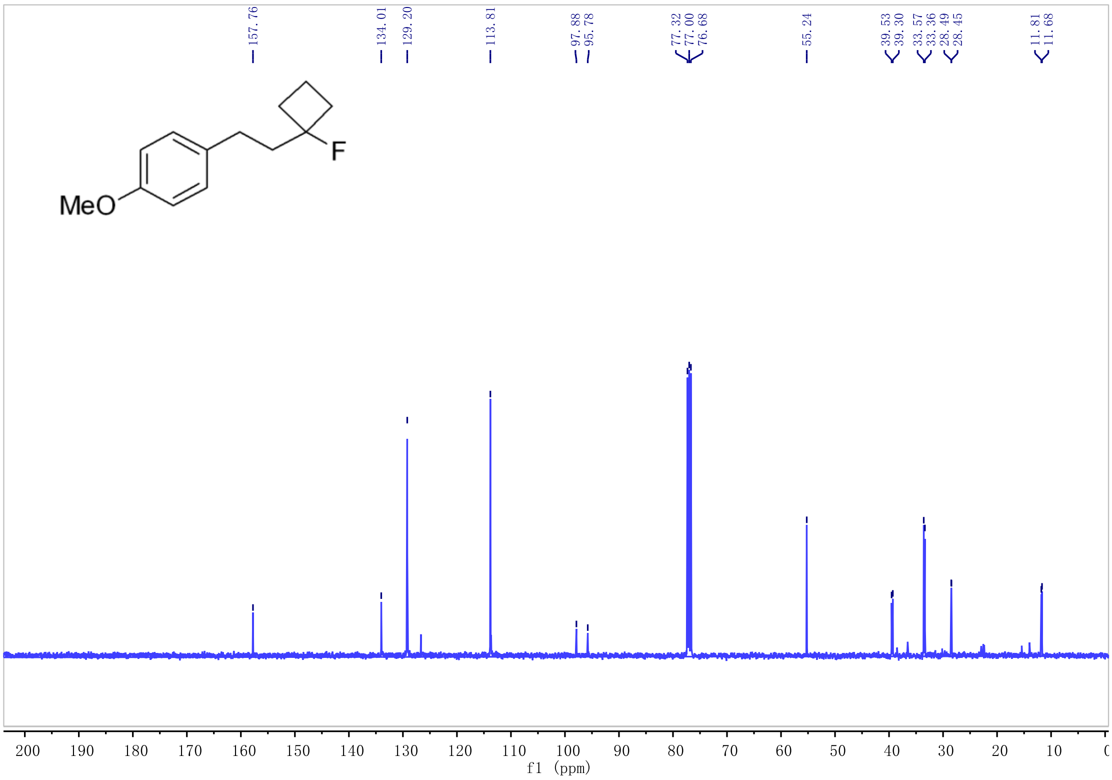

Supplementary Figure 260. ^{13}**C NMR (100 MHz, CDCl$_3$) spectrum for 103**

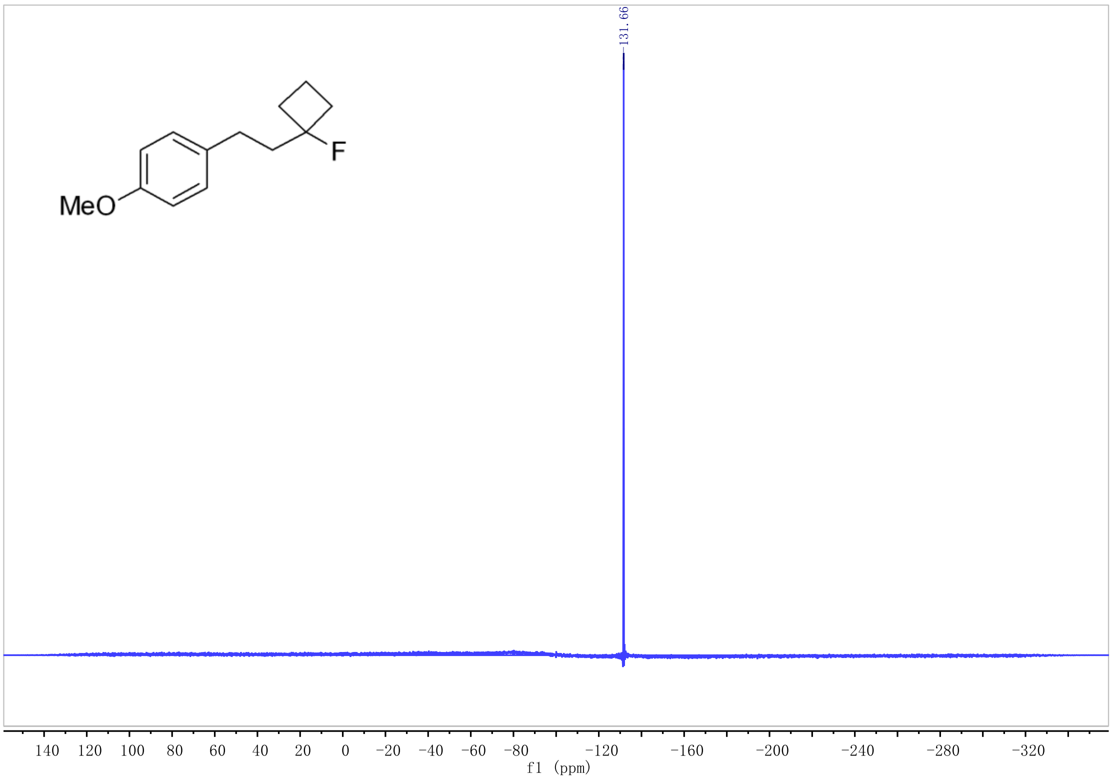

Supplementary Figure 261. ^{19}**F NMR (377 MHz, CDCl$_3$) spectrum for 103**

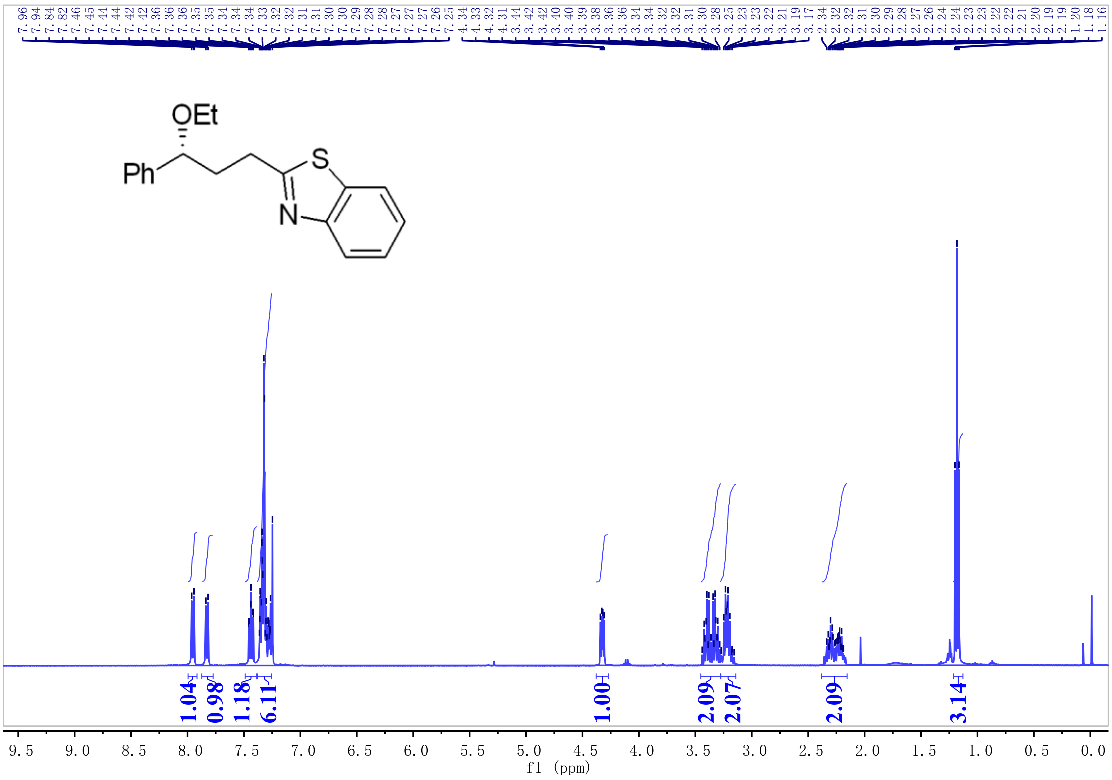

Supplementary Figure 262. ¹H NMR (400 MHz, $CDCl_3$) spectrum for 116

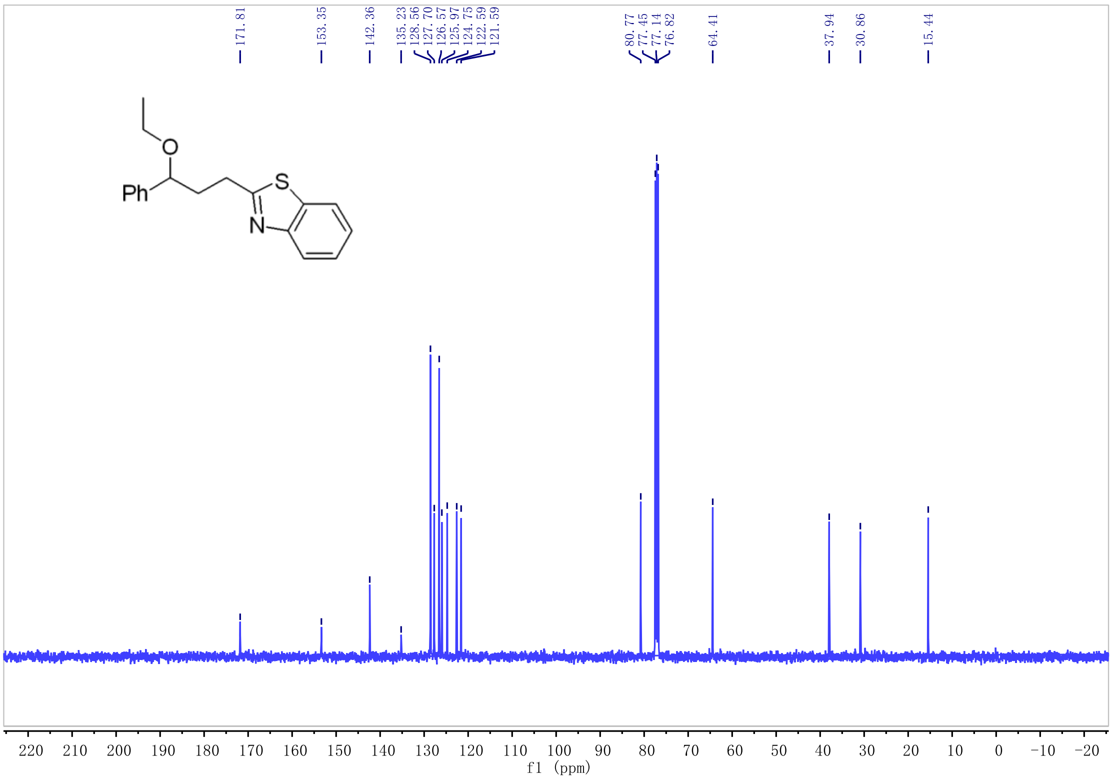

Supplementary Figure 263. ¹³C NMR (100 MHz, $CDCl_3$) spectrum for 116

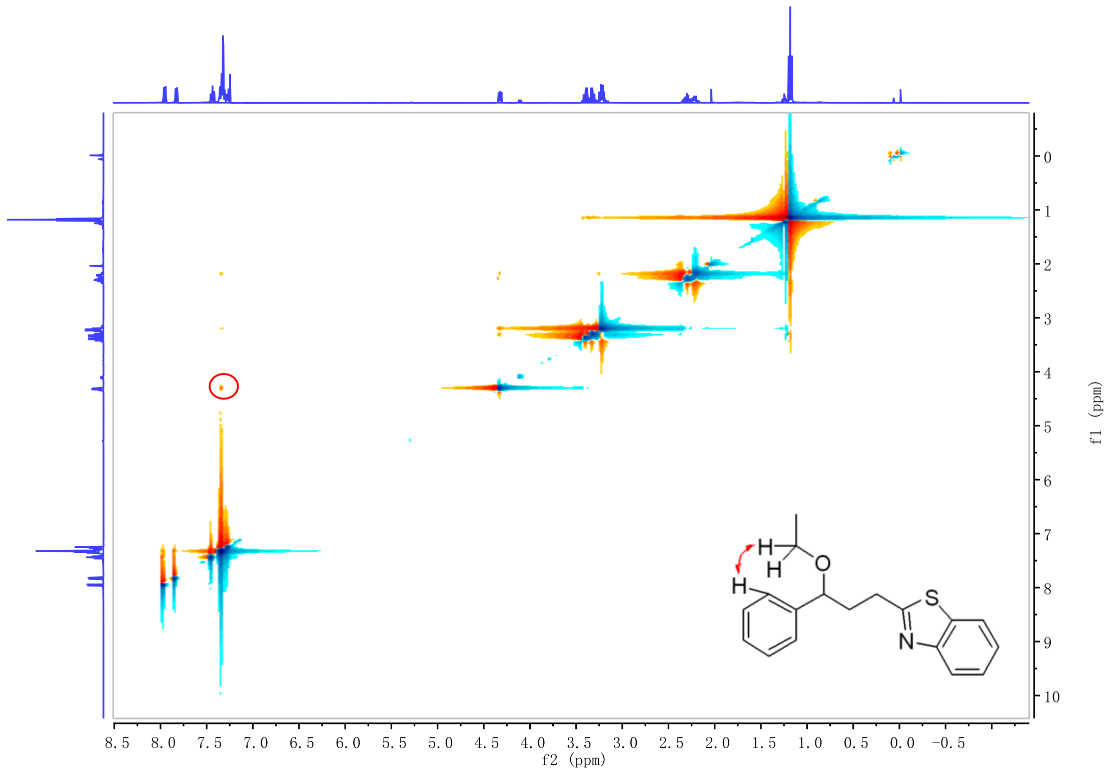

Supplementary Figure 264. NOESY spectrum for 116

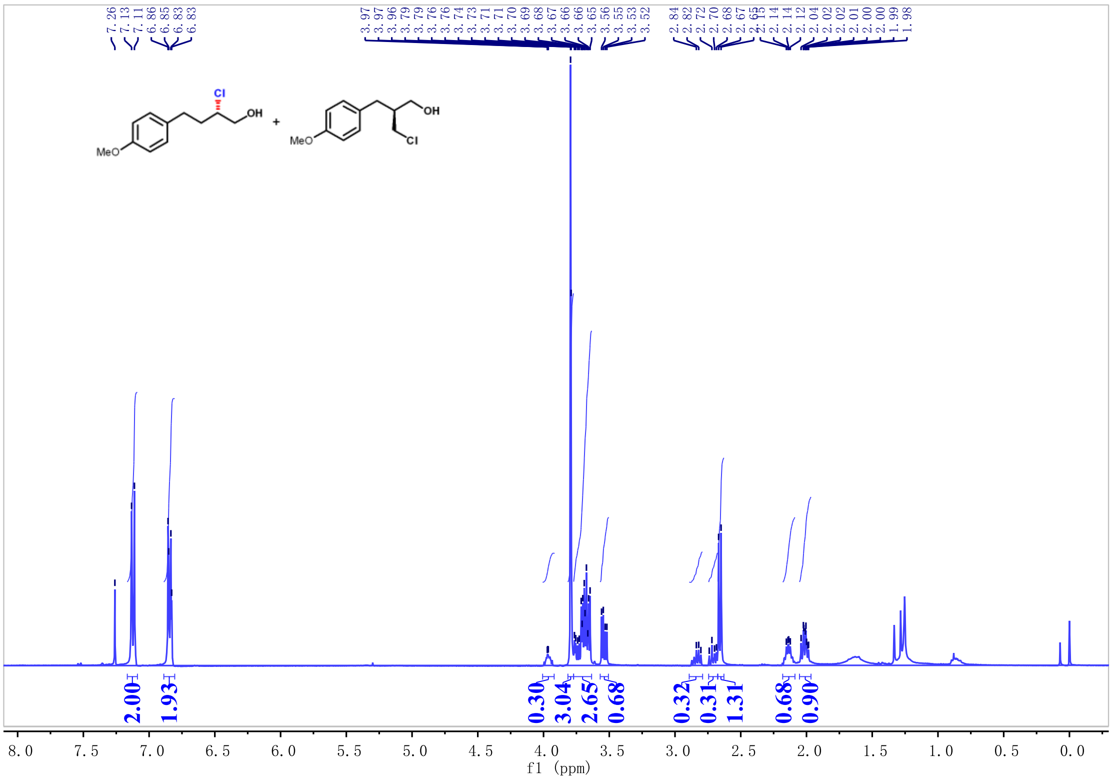

Supplementary Figure 265. ^{1}H NMR (400 MHz, $CDCl_3$) spectrum for 118 and 119

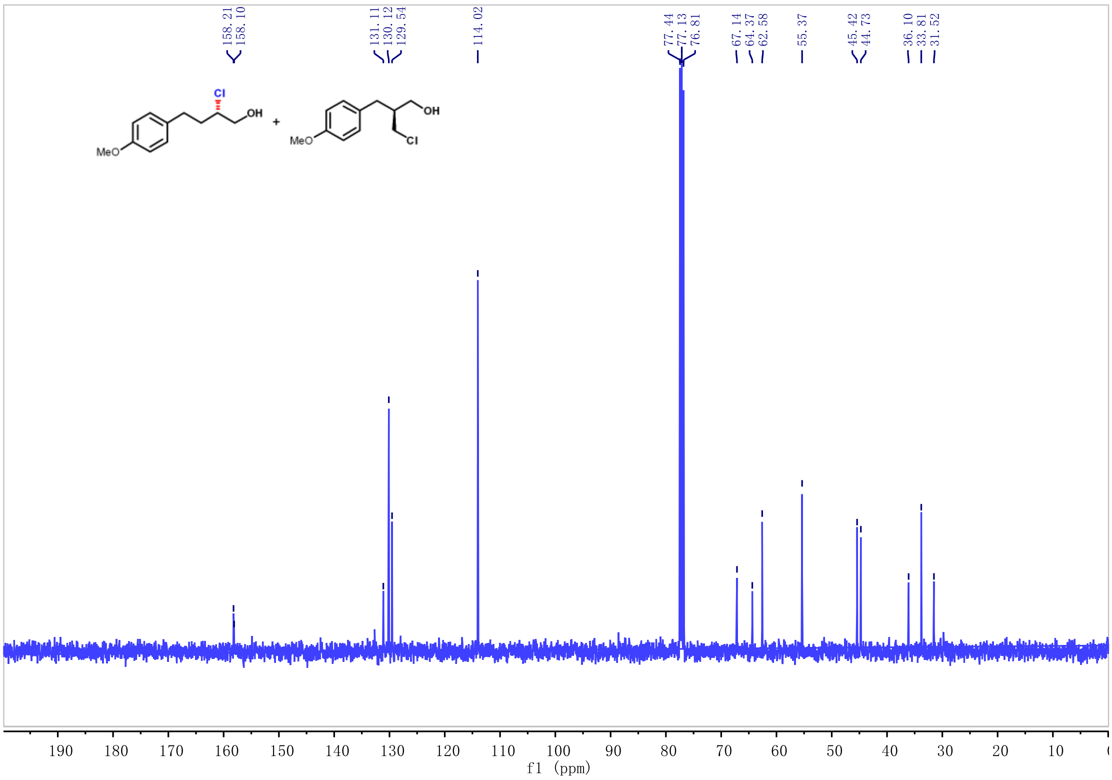

Supplementary Figure 266. ^{13}**C NMR (100 MHz,** $CDCl_3$**) spectrum for 118 and 119**

Characterization of Structurally Novel Compounds

1-(3-ethoxy-3-methylbutyl)-4-methoxybenzene (3)

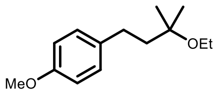

Following the General Procedure B, **3** was obtained in 91% yield as a colorless oil; **^{1}H NMR (400 MHz, Chloroform-*d*)**: δ 7.12 (d, *J* = 8.5 Hz, 2H), 6.83 (d, *J* = 8.6 Hz, 2H), 3.79 (s, 3H), 3.42 (q, *J* = 7.0 Hz, 2H), 2.66 – 2.45 (m, 2H), 1.83 – 1.63 (m, 2H), 1.22 (s, 6H), 1.20 (t, *J* = 7.0 Hz, 3H) ppm; **^{13}C NMR (101 MHz, Chloroform-*d*)**: δ 157.7, 135.1, 129.2, 113.9, 74.3, 56.5, 55.4, 42.3, 29.5, 25.9, 16.3 ppm. **HRMS (ESI, m/z)**: calculated for $[M+H]^+$: 223.1698, found: 223.1670. **IR (KBr)**: 2973, 1512, 1247, 1073, 1040, 821 cm^{-1}.

1,1,1-trifluoro-N-(4-(4-methoxyphenyl)-2-methylbutan-2-yl)methanesulfonamide (2)

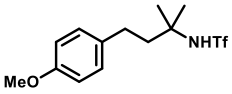

Following the General Procedure A, **2** was obtained in 88% yield as a colorless oil; **^{1}H NMR (400 MHz, Chloroform-*d*)**: δ 7.13 – 7.08 (m, 2H), 6.87 – 6.81 (m, 2H), 4.94 (s, 1H), 3.79 (s, 3H), 2.67 – 2.59 (m, 2H), 1.95 – 1.88 (m, 2H), 1.46 (s, 6H) ppm; **^{13}C NMR (101 MHz, Chloroform-*d*)**: δ 158.1, 133.0, 129.3, 119.3 (q, *J* = 320.9 Hz), 114.1, 60.7, 55.4, 45.5, 29.6, 27.9 ppm; **^{19}F NMR (376 MHz, Chloroform-d)**: δ -77.67 ppm. **HRMS (ESI, m/z)**: calculated for $[M+H]^+$: 326.1038, found: 326.1035. **IR (KBr)**: 3296, 2939, 1712, 1612, 1514, 824, 628, 586 cm^{-1}.

4-(4-methoxyphenyl)-2-methylbutan-2-amine (2')

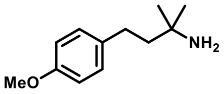

2' was obtained in 75% yield as a colorless oil; **^{1}H NMR (400 MHz, Chloroform-*d*)**: δ 7.15 – 7.09 (m, 2H), 6.88 – 6.81 (m, 2H), 4.32 (s, 2H), 3.79 (s, 3H), 2.74 – 2.51 (m, 2H), 2.23 – 1.91 (m, 2H), 1.65 (s, 6H) ppm; **^{13}C NMR (101 MHz, Chloroform-*d*)**: δ 158.2, 132.5, 129.3, 114.2, 68.1, 55.4, 44.0, 35.1, 29.9, 27.7 ppm; **HRMS (ESI, m/z)**: calculated for $[M+H]^+$: 194.1545, found: 194.1542.

4-(3-ethoxy-3-methylbutyl)-1-methoxy-2-methylbenzene (4)

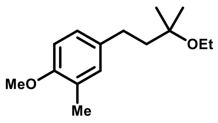

Following the General Procedure B, **4** was obtained in 71% yield as a colorless oil; **^{1}H NMR (400 MHz, Chloroform-*d*)**: δ 7.00 – 6.95 (m, 2H), 6.76 – 6.72 (m, 1H), 3.81 (s, 3H), 3.42 (q, *J* = 7.0 Hz, 2H), 2.61 – 2.50 (m, 2H), 2.20 (s, 3H), 1.79 – 1.69 (m, 2H), 1.22 (s, 6H), 1.20 (t, *J* = 7.0 Hz, 3H) ppm; **^{13}C NMR (101 MHz, Chloroform-*d*)**: δ 155.9, 134.7, 130.8, 126.5, 126.3, 110.0, 74.3, 56.5, 55.5, 42.3, 29.5, 26.0, 16.3 ppm. **HRMS (ESI, m/z)**: calculated for $[M+H]^+$: 237.1855, found: 237.1861. **IR (KBr)**: 2973, 1742, 1506, 1252, 1073, 1039 cm^{-1}.

1,1,1-trifluoro-N-(4-(4-methoxy-3-methylphenyl)-2-methylbutan-2-yl)methanesulfonamide (28)

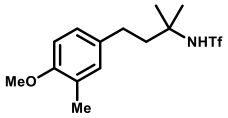

Following the General Procedure A, **28** was obtained in 59% yield as a colorless oil; **^{1}H NMR (400 MHz, Chloroform-*d*)**: δ 7.00 – 6.93 (m, 2H), 6.78 – 6.73 (m, 1H), 4.90 (s, 1H), 3.81 (s, 3H), 2.73 – 2.45 (m, 2H), 2.20 (s, 3H), 1.98 – 1.76 (m, 2H), 1.46 (s, 6H) ppm; **^{13}C NMR (101 MHz, Chloroform-*d*)**: δ 156.3, 132.6, 130.7, 126.9, 126.4, 119.3 (q, *J* = 320.8 Hz), 110.2, 60.7, 55.5, 45.6, 29.6, 27.8, 16.3 ppm; **^{19}F NMR (376 MHz, Chloroform-d)**: δ -77.68 ppm. **HRMS (ESI, m/z)**: calculated for $[M+H]^+$: 340.1194, found: 340.1192. **IR (KBr)**: 2949, 1506, 1426, 1369, 1255, 1228, 1194, 1144, 1000, 627 cm^{-1}.

4-(3-ethoxy-3-methylbutyl)-1,2-dimethylbenzene (5)

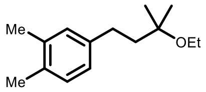

Following the General Procedure B, **5** was obtained in 82% yield as a colorless oil; **^{1}H NMR (400 MHz, Chloroform-*d*)**: δ 7.09 – 7.04 (m, 1H), 7.00 (s, 1H), 6.98 – 6.93 (m, 1H), 3.44 (q, *J* = 7.0 Hz, 2H), 2.65 – 2.50 (m, 2H), 2.26 (s, 3H), 2.25 (s, 3H), 1.83 – 1.72 (m, 2H), 1.24 (s, 6H), 1.22-1.20 (t, *J* = 7.0 Hz, 3H) ppm; **^{13}C NMR (100 MHz, Chloroform-*d*)**: δ 140.5, 136.6, 133.8, 129.8, 129.7, 125.7, 74.3, 56.5, 42.2, 29.9, 26.0, 19.9, 19.4, 16.3 ppm. **HRMS (ESI, m/z):** calculated for $[M+H]^+$: 221.1905, found: 221.1902. **IR (KBr)**: 2973, 1505, 1455, 1362, 1116, 1073, 817 cm^{-1}.

N-(4-(3,4-dimethylphenyl)-2-methylbutan-2-yl)-1,1,1-trifluoromethanesulfonamide (30)

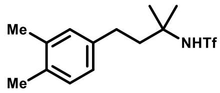

Following the General Procedure A, **30** was obtained in 82% yield as a colorless oil; **^{1}H NMR (400 MHz, Chloroform-*d*)**: δ 7.12 – 7.08 (m, 1H), 7.03 – 7.01 (m, 1H), 7.00 – 6.95 (m, 1H), 5.22 (s, 1H), 2.71 – 2.63 (m, 2H), 2.29 (s, 3H), 2.28 (s, 3H), 2.00 – 1.92 (m, 2H), 1.50 (s, 6H) ppm; **^{13}C NMR (100 MHz, Chloroform-*d*)**: δ 138.5, 136.9, 134.5, 130.0, 129.8, 125.8, 119.4 (q, *J* = 320.9 Hz), 60.8, 45.6, 30.0, 27.8, 19.8, 19.4 ppm; **^{19}F NMR (376 MHz, Chloroform-d)**: δ -77.66 ppm. **HRMS (ESI, m/z)**: calculated for [M+H]+: 324.1245, found: 324.1249. **IR (KBr)**: 3294, 2940, 1505, 1370, 1229, 1193, 1145, 1001, 628 cm^{-1}.

(3-ethoxy-3-methylbutyl)benzene (6)

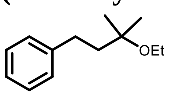

Following the General Procedure B, **6** was obtained in 37% yield as a colorless oil; **^{1}H NMR (400 MHz, Chloroform-*d*)**: δ 7.32 – 7.25 (m, 2H), 7.22 – 7.18 (m, 3H), 3.43 (q, *J* = 7.0 Hz, 2H), 2.71 – 2.60 (m, 2H), 1.88 – 1.64 (m, 2H), 1.23 (s, 6H), 1.20 (t, *J* = 7.0 Hz, 3H) ppm; **^{13}C NMR (100 MHz, Chloroform-*d*)**: δ 143.1, 128.4, 125.7, 74.3, 56.5, 42.0, 30.4, 26.0, 16.3 ppm. **HRMS (ESI, m/z)**: calculated for $[M+H]^+$: 193.1592, found: 193.1595. **IR (KBr)**: 2970, 1454, 1261, 1072, 700 cm^{-1}.

1,1,1-trifluoro-N-(2-methyl-4-phenylbutan-2-yl)methanesulfonamide (31)

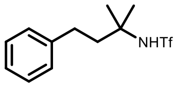

Following the General Procedure A, **31** was obtained in 55% yield as a colorless oil; **^{1}H NMR (400 MHz, Chloroform-*d*)**: δ 7.42 – 6.97 (m,

5H), 4.77 (s, 1H), 2.76 – 2.57 (m, 2H), 2.01 – 1.85 (m, 2H), 1.47 (s, 6H) ppm; **^{13}C NMR (100 MHz, Chloroform-*d*)**: δ 140.9, 128.7, 128.4, 126.3, 119.3 (q, *J* = 320.7 Hz), 60.7, 45.3, 30.5, 27.9 ppm; **^{19}F NMR (377 MHz, Chloroform-d)**: δ -77.65 ppm. **HRMS (ESI, m/z)**: calculated for $[M+H]^+$: 296.0932, found: 296.0926. **IR (KBr)**: 2933, 1426, 1368, 1229, 1194, 1144, 1001 cm^{-1}.

4-(3-ethoxy-3-methylbutyl)-1,1'-biphenyl (7)

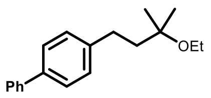

Following the General Procedure B, **7** was obtained in 86% yield as a colorless oil; **^{1}H NMR (400 MHz, Chloroform-*d*)**: δ 7.64 – 7.60 (m, 2H), 7.58 – 7.54 (m, 2H), 7.49 – 7.44 (m, 2H), 7.39 – 7.34 (m, 1H), 7.33 – 7.29 (m, 2H), 3.48 (q, *J* = 7.0 Hz, 2H), 2.78 – 2.67 (m, 2H), 1.90 – 1.79 (m, 2H), 1.29 (s, 6H), 1.25 (t, *J* = 7.0 Hz, 3H) ppm; **^{13}C NMR (100 MHz, Chloroform-*d*)**: δ 142.3, 141.2, 138.8, 128.9, 128.9, 127.2, 127.1, 74.3, 56.6, 42.0, 30.1, 26.0, 16.4 ppm. **HRMS (ESI, m/z)**: calculated for $[M+H]^+$: 269.1905, found: 269.1907. **IR (KBr)**: 2974, 1742, 1486, 1374, 1243, 1073, 697 cm^{-1}.

N-(4-([1,1'-biphenyl]-4-yl)-2-methylbutan-2-yl)-1,1,1-trifluoromethanesulfonamide (32)

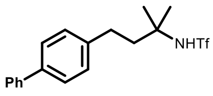

Following the General Procedure A, **32** was obtained in 77% yield as colorless oil; **^{1}H NMR (400 MHz, Chloroform-*d*)**: δ 7.62 – 7.58 (m, 2H), 7.57 – 7.53 (m, 2H), 7.49 – 7.42 (m, 2H), 7.39 – 7.33 (m, 1H), 7.28 (d, *J* = 8.3 Hz, 2H), 5.06 (s, 1H), 2.80 – 2.69 (m, 2H), 2.05 – 1.95 (m, 2H), 1.50 (s, 6H) ppm; **^{13}C NMR (100 MHz, Chloroform-*d*)**: δ 141.0, 140.1, 139.3, 128.9, 128.9, 127.4, 127.3, 127.1, 119.4 (q, *J* = 320.8 Hz), 60.7, 45.2, 30.2, 27. 9 ppm; **^{19}F NMR (377 MHz, Chloroform-d)**: δ -77.60 ppm. **HRMS (ESI, m/z):** calculated for $[M+H]^+$: 372.1245, found: 372.1243. **IR (KBr)**: 3295, 2983, 1740, 1371, 1188, 1146, 1003, 769, 626 cm^{-1}.

2-(3-ethoxy-3-methylbutyl)naphthalene (8)

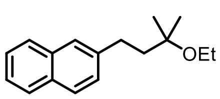

Following the General Procedure B, **8** was obtained in 68% yield as a colorless oil; **^{1}H NMR (400 MHz, Chloroform-*d*)**: δ 7.83 - 7.75 (m, 3H), 7.64 (s, 1H), 7.49 – 7.40 (m, 2H), 7.38 – 7.32 (m, 1H), 3.47 (q, *J* = 7.0 Hz, 2H), 2.86 – 2.75 (m, 2H), 1.93 – 1.83 (m, 2H), 1.27 (s, 6H), 1.23 (t, *J* = 7.0 Hz, 3H) ppm; **^{13}C NMR (100 MHz, Chloroform-*d*)**: δ 140.6, 133.8, 132.0, 127.9, 127.7, 127.5, 127.5, 126.2, 126.0, 125.1, 74.3, 56.6, 41.9, 30.6, 26.0, 16.3 ppm. **HRMS (ESI, m/z)**: calculated for $[M+H]^+$: 243.1749, found: 243.1755. **IR (KBr)**: 2973, 1362, 1115, 1073, 817, 741 cm^{-1}.

1,1,1-trifluoro-N-(2-methyl-4-(naphthalen-2-yl)butan-2-yl)methanesulfonamide (33)

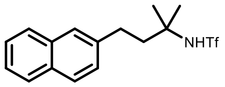

Following the General Procedure A, **33** was obtained in 37% yield as a colorless oil; **^{1}H NMR (400 MHz, Chloroform-*d*)**: δ 7.84 – 7.73 (m, 3H), 7.64 (s, 1H), 7.50 – 7.42 (m, 2H), 7.33 (dd, *J* = 8.4, 1.6 Hz, 1H), 4.97 (s, 1H), 2.95 – 2.66 (m, 2H), 2.26 – 1.96 (m, 2H), 1.50 (s, 6H) ppm;

^{13}C NMR (100 MHz, Chloroform-*d*): δ 138.5, 133.7, 132.2, 128.3, 127.7, 127.5, 127.1, 126.5, 126.2, 125.5, 119.3 (q, *J* = 320.8 Hz), 60.8, 45.1, 30.7, 27.9 ppm; **^{19}F NMR (377 MHz, Chloroform-d)**: δ -77.60 ppm. **HRMS (ESI, m/z):** calculated for $[M+H]^+$: 346.1089, found: 346.1085. **IR (KBr)**: 1425, 1370, 1195, 1004, 626 cm^{-1}.

1-(3-ethoxy-3-ethylpentyl)-4-methoxybenzene (9)

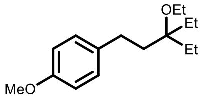

Following the General Procedure B, **9** was obtained in 95% yield as a colorless oil; **^{1}H NMR (400 MHz, Chloroform-*d*)**: δ 7.14 – 7. 08 (m, 2H), 6.86 – 6.79 (m, 2H), 3.78 (s, 3H), 3.34 (q, *J* = 7.0 Hz, 2H), 2.55 – 2.44 (m, 2H), 1.71 – 1.63 (m, 2H), 1.58 – 1.43 (m, 4H), 1.20 (t, *J* = 7.0 Hz, 3H), 0.84 (t, *J* = 7.5 Hz, 6H) ppm; **^{13}C NMR (100 MHz, Chloroform-*d*)**: δ 157.7, 135.2, 129.2, 113.9, 78.6, 55.4, 55.4, 36.4, 28.7, 26.6, 16.1, 7.7 ppm. **HRMS (ESI, m/z)**: calculated for $[M+H]^+$: 251.2011, found: 251.2015. **IR (KBr)**: 2969, 1512, 1463, 1246, 1177, 1076 cm^{-1}.

1-(2-(1-isopropoxycyclobutyl)ethyl)-4-methoxybenzene (10)

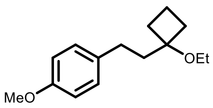

Following the General Procedure B, **10** was obtained in 89% yield as a colorless oil; **^{1}H NMR (400 MHz, Chloroform-*d*)**: δ 7.16 – 7.11 (m, 2H), 6.86 – 6.81 (m, 2H), 3.79 (s, 3H), 3.34 (q, *J* = 7.0 Hz, 2H), 2.61 – 2.52 (m, 2H), 2.18 – 2.05 (m, 2H), 1.98 – 1.87 (m, 4H), 1.84 – 1.71 (m, 1H), 1.66- 1.52 (m, 1H), 1.22 (t, *J* = 7.0 Hz, 3H) ppm; **^{13}C NMR (100 MHz, Chloroform-*d*)**: δ 157.8, 135.0, 129.3, 113.9, 78.8, 56.8, 55.4, 37.5, 32.3, 28.6, 16.0, 12.9 ppm. **HRMS (ESI, m/z)**: calculated for $[M+H]^+$: 235.1698, found: 235.1696. **IR (KBr)**: 2972, 1512, 1463, 1246, 1177, 1076 cm^{-1}.

1,1,1-trifluoro-N-(1-(4-methoxyphenethyl)cyclobutyl)methanesulfonamide (34)

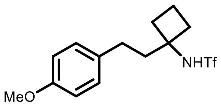

Following the General Procedure A, **34** was obtained in 42% yield as a colorless oil; **^{1}H NMR (400 MHz, Chloroform-*d*)**: δ 7.17 – 7.09 (m, 2H), 6.89 – 6.82 (m, 2H), 5.17 (s, 1H), 3.80 (s, 3H), 2.68 – 2.57 (m, 2H), 2.48 – 2.37 (m, 2H), 2.19 – 2.06 (m, 4H), 2.02 – 1.91 (m, 1H), 1.88 – 1.76 (m, 1H) ppm; **^{13}C NMR (100 MHz, Chloroform-*d*)**: δ 158.1, 133.1, 129.3, 119.3 (q, *J* = 321.0 Hz), 114.2, 62.1, 55.4, 41.2, 33.6, 29.1, 14.7 ppm; **^{19}F NMR (377 MHz, Chloroform-d)**: δ -77.99 ppm. **HRMS (ESI, m/z)**: calculated for $[M+H]^+$: 338.1038, found: 338.1041. **IR (KBr)**: 2947, 1513, 1421, 1243, 820 cm^{-1}.

1-(2-(1-ethoxycyclopentyl)ethyl)-4-methoxybenzene (11)

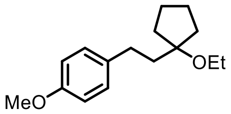

Following the General Procedure B, **11** was obtained in 86% yield as a colorless oil; **^{1}H NMR (400 MHz, Chloroform-*d*)**: δ 7.14 – 7.08 (m, 2H), 6.85 – 6.79 (m, 2H), 3.78 (s, 3H), 3.38 (q, *J* = 7.0 Hz, 2H), 2.76 – 2.46 (m, 2H), 1.91 – 1.78 (m, 4H), 1.78 – 1.69 (m, 2H), 1.64 – 1.52 (m, 2H), 1.50 – 1.40 (m, 2H), 1.20 (t, *J* = 7.0 Hz, 3H) ppm; **^{13}C NMR (100 MHz, Chloroform-*d*)**: δ 157.7, 135.2, 129.2, 113.9, 86.4, 57.0, 55.4, 38.8, 36.5, 30.0, 23.9, 16.3 ppm. **HRMS (ESI, m/z)**: calculated for $[M+H]^+$: 249.1855, found: 249.1856. **IR (KBr)**: 2955, 1512, 1246, 1073, 621 cm^{-1}.

1,1,1-trifluoro-N-(1-(4-methoxyphenethyl)cyclopentyl)methanesulfonamide (35)

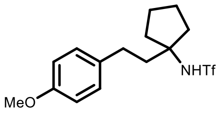

Following the General Procedure A, **35** was obtained in 45% yield as a colorless oil; **^{1}H NMR (400 MHz, Chloroform-*d*)**: δ 7.15 – 7.07 (m, 2H), 6.87 – 6.80 (m, 2H), 4.82 (s, 1H), 3.79 (s, 3H), 2.74 – 2.62 (m, 2H), 2.09 –1.97 (m, 4H), 1.89 – 1.68 (m, 6H) ppm; **^{13}C NMR (100 MHz, Chloroform-*d*)**: δ 158.0, 133.3, 129.3, 119.3 (q, *J* = 320.9 Hz), 114.1, 71.6, 55.4, 42.3, 38.6, 30.3, 22.9 ppm; **^{19}F NMR (377 MHz, Chloroform-d)**: δ -77.97 ppm. **HRMS (ESI, m/z)**: calculated for [M+H]$^+$: 352.1194, found: 352.1198. **IR (KBr)**: 2958, 1513, 1370, 1192, 1144, 1036, 611 cm^{-1}.

1-(2-(1-ethoxycyclohexyl)ethyl)-4-methoxybenzene (12)

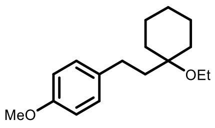

Following the General Procedure B, **12** was obtained in 77% yield as a colorless oil; **^{1}H NMR (400 MHz, Chloroform-*d*)**: δ 7.14 – 7.08 (m, 2H), 6.86 – 6.80 (m, 2H), 3.79 (s, 3H), 3.38 (q, *J* = 7.0 Hz, 2H), 2.62 – 2.50 (m, 2H), 1.82 – 1.74 (m, 2H), 1.73 – 1.67 (m, 2H), 1.63 – 1.53 (m, 3H), 1.49 – 1.40 (m, 2H), 1.37 – 1.27 (m, 3H), 1.24 (t, *J* = 7.0 Hz, 3H) ppm; **^{13}C NMR (100 MHz, Chloroform-*d*)**: δ 157.7, 135.4, 129.2, 113.9, 74.8, 55.4, 55.2, 39.4, 34.7, 28.3, 26.2, 22.1, 16.2 ppm. **HRMS (ESI, m/z)**: calculated for [M+H]$^+$: 263.2011, found: 263.2015. **IR (KBr)**: 2931, 1512, 1245, 1701, 1040, 821 cm^{-1}.

1,1,1-trifluoro-N-(1-(4-methoxyphenethyl)cyclohexyl)methanesulfonamide (36)

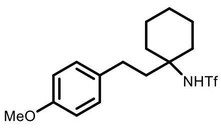

Following the General Procedure A, **36** was obtained in 51% yield as a colorless oil; **^{1}H NMR (400 MHz, Chloroform-*d*)**: δ 7.14 – 7.08 (m, 2H), 6.86 – 6.80 (m, 2H), 4.66 (s, 1H), 3.78 (s, 3H), 2.65 – 2.55 (m, 2H), 2.06 – 1.96 (m, 2H), 1.91 – 1.81 (m, 2H), 1.74 – 1.65 (m, 2H), 1.60 – 1.40 (m, 6H) ppm; **^{13}C NMR (100 MHz, Chloroform-*d*)**: δ 158.0, 133.4, 129.4, 119.3 (q, *J* = 321.4 Hz), 114.1, 63.7, 55.4, 40.8, 36.1, 28.5, 25.2, 21.9 ppm; **^{19}F NMR (377 MHz, Chloroform-d)**: δ -77.59 ppm. **HRMS (ESI, m/z):** calculated for [M+H]$^+$: 366.1351, found: 366.1354. **IR (KBr)**: 2938, 1513, 1374, 1247, 1225, 1192, 1144, 989, 630 cm^{-1}.

1,1,1-trifluoro-N-(3-(4-methoxyphenyl)propyl)methanesulfonamide (37)

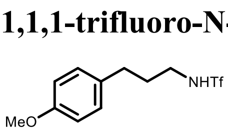

Following the General Procedure A, **37** was obtained in 26% yield as a colorless oil; **^{1}H NMR (400 MHz, Chloroform-*d*)**: δ 7.12 – 7.05 (m, 2H), 6.89 – 6.81 (m, 2H), 4.81 (br, 1H), 3.79 (s, 3H), 3.31 (q, *J* = 6.7 Hz, 2H), 2.73 – 2.50 (m, 2H), 1.96 – 1.87 (m, 2H) ppm; **^{13}C NMR (100 MHz, Chloroform-*d*)**: δ 158.3, 132. 2, 129.3, 119.7 (q, *J* = 321.0 Hz), 114.2, 55.4, 44.0, 32.0, 31.7; **^{19}F NMR (377 MHz, Chloroform-d)**: δ -77.17 ppm. **HRMS (ESI, m/z):** calculated for [M+H]$^+$: 298.0725, found: 298.0727. **IR (KBr)**: 2942, 1694, 1588, 1501, 1464, 1329, 1231, 1127, 1002, 835, 607 cm^{-1}.

methyl 11-(4-methoxyphenyl)-9-((trifluoromethyl)sulfonamido)undecanoate (38)

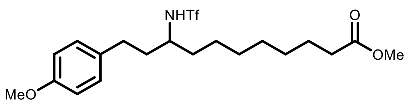

Following the General Procedure A, **38** was obtained in 55% yield as a colorless oil; **^{1}H NMR (400 MHz, Chloroform-*d*)**: δ 7.11 – 7.05 (m, 2H), 6.86 – 6.79 (m, 2H), 4.92 (d, *J* = 9.3 Hz, 1H), 3.78 (s, 3H), 3.65 (s, 3H), 3.62 – 3.51 (m, 1H), 2.72 – 2.52 (m, 2H), 2.29 (t, *J* = 7.5 Hz, 2H), 1.94 – 1.72 (m, 2H), 1.71 – 1.55 (m, 4H), 1.29 – 1.25 (m, 8H) ppm; **^{13}C NMR (100 MHz, Chloroform-*d*)**: δ 174.6, 158.1, 132.9, 129.3, 119.7 (q, *J* = 320.6 Hz), 114.1, 56.5, 55.4, 51.6, 37.5, 35.4, 34.1, 30.9, 29.2, 29.2, 29.1, 29.1, 25.2, 24.9 ppm; **^{19}F NMR (377 MHz, Chloroform-d)**: δ -77.42 ppm. **HRMS (ESI, m/z):** calculated for [M+H]$^+$: 454.1875, found: 454.1871. **IR (KBr)**: 2930, 1717, 1513, 1375, 1247, 1228, 1190, 1149 cm^{-1}.

1-(3-ethoxy-4-phenylbutyl)-4-methoxybenzene (13)

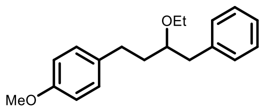

Following the General Procedure B, **13** was obtained in 44% yield; **^{1}H NMR (400 MHz, Chloroform-*d*)**: δ 7.30 – 7.25 (m, 2H), 7.23 – 7.16 (m, 3H), 7.09 – 7.03 (m, 2H), 6.84 – 6.78 (m, 2H), 3.78 (s, 3H), 3.53 – 3.33 (m, 3H), 2.87 (dd, *J* = 13.6, 6.1 Hz, 1H), 2.79 – 2.67 (m, 2H), 2.60 – 2.50 (m, 1H), 1.79 – 1.66 (m, 2H), 1.17 (t, *J* = 7.0 Hz, 3H) ppm; **^{13}C NMR (100 MHz, Chloroform-*d*)**: δ 157.7, 139.1, 134.4, 129.6, 129.4, 128.3, 126.1, 113.8, 80.1, 65.0, 55.4, 40.9, 36.3, 31.0, 15.7 ppm. **HRMS (ESI, m/z):** calculated for [M+H]$^+$: 285.1855, found: 285.1859. **IR (KBr)**: 2927, 1511, 1246, 1038, 700 cm^{-1}.

1-(3-ethoxy-5-phenylpentyl)-4-methoxybenzene (14)

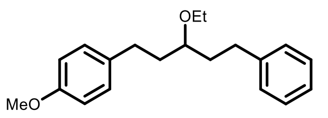

Following the General Procedure B, **14** was obtained in 50% yield as a colorless oil; **^{1}H NMR (400 MHz, Chloroform-*d*)**: δ 7.30 – 7.22 (m, 2H), 7.20 – 7.12 (m, 3H), 7.12 – 7.06 (m, 2H), 6.84 – 6.78 (m, 2H), 3.78 (s, 3H), 3.55 – 3.33 (m, 3H), 2.85 – 2.51 (m, 4H), 1.88 – 1.68 (m, 2H), 1.28 – 1.12 (m, 5H) ppm; **^{13}C NMR (100 MHz, Chloroform-*d*)**: δ 158.0, 142.5, 131.1, 130.5, 129.4, 128.5, 128.4, 125.8, 113.7, 80.2, 64.9, 55.3, 39.9, 35.9, 32.0, 15.7 ppm. **HRMS (ESI, m/z):** calculated for [M+H]$^+$: 299.2011, found: 299.2018. **IR (KBr)**: 2928, 1512, 1247, 1094, 1037, 700 cm^{-1}.

N-(1-ethoxy-3-(4-methoxyphenyl)propyl)-4-fluorobenzamide (15)

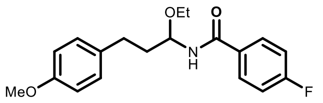

Following the General Procedure B, **15** was obtained in 92% yield as a colorless oil; **^{1}H NMR (400 MHz, Chloroform-*d*):** δ 7.71 – 7.66 (m, 2H), 7.14 – 7.03 (m, 4H), 6.84 – 6.78 (m, 2H), 6.40 (d, *J* = 9.5 Hz, 1H), 5.47 – 5.39 (m, 1H), 3.76 (s, 3H), 3.73 – 3.64 (m, 1H), 3.60 – 3.50 (m, 1H), 2.83 – 2.72 (m, 1H), 2.71 – 2.61 (m, 1H), 2.10 – 1.92 (m, 2H), 1.20 (t, *J* = 7.0 Hz, 3H) ppm; **^{13}C NMR (101 MHz, Chloroform-*d*):** δ 166.2, 163.7, 158.0, 133.4, 130.1 (d, *J* = 2.8 Hz), 129.6, 129.5, 115.7 (d, *J* = 21.9 Hz), 114.0, 79.8, 64.0, 55.3, 37.4, 30.3, 15.3 ppm. **HRMS (ESI, m/z)**: calculated for [M+H]$^+$: 332.1662, found: 332.1665. **IR (KBr)**: 1647, 1604, 1513, 1502, 1245, 852 cm^{-1}.

N-(3-(4-methoxyphenyl)-1-((trifluoromethyl)sulfonamido)propyl)benzamide (39)

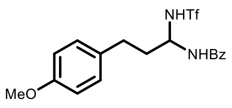

Following the General Procedure A, **39** was obtained in 64% yield as a white solid; **^{1}H NMR (400 MHz, DMSO-*d6*)**: δ 10.03 (s, 1H), 9.11 (d, *J* = 8.4 Hz, 1H), 7.85 (d, *J* = 7.3 Hz, 2H), 7.60 – 7.53 (m, 1H), 7.52 – 7.44 (m, 2H), 7.14 (d, *J* = 8.4 Hz, 2H), 6.85 (d, *J* = 8.4 Hz, 2H), 5.53 – 5.36 (m, 1H), 3.71 (s, 3H), 2.65 – 2.63 (m, 2H), 2.19 – 2.00 (m, 2H) ppm; **^{13}C NMR (100 MHz, DMSO-*d6*)**: δ 166.2, 158.1, 134.4, 132.9, 132.1, 129.8, 128.8, 128.0, 119.9 (q, *J* = 322.3 Hz), 114.3, 61.1, 55.5, 37.3, 30.8 ppm; **^{19}F NMR (377 MHz, DMSO-d6)**: δ -77.42 ppm. **HRMS (ESI, m/z)**: calculated for $[M+H]^+$: 417.1096, found: 417.1102. **IR (KBr)**: 3443, 2254, 2128, 1660, 1026, 1004, 826, 764 cm^{-1}.

benzyl (1-ethoxy-3-(4-methoxyphenyl)propyl)carbamate (16)

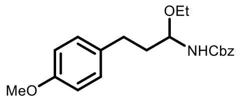

Following the General Procedure B, **16** was obtained in 73% yield as a white solid; **^{1}H NMR (400 MHz, Chloroform-*d*)**: δ 7.50 – 7.26 (m, 5H), 7.15 – 7.00 (m, 2H), 6.88 – 6.80 (m, 2H), 5.25 – 4.90 (m, 4H), 3.78 (s, 3H), 3.75 – 3.45 (m, 2H), 2.76 – 2.54 (m, 2H), 2.00 – 1.70 (m, 2H), 1.21 (t, *J* = 7.0 Hz, 3H) ppm; **^{13}C NMR (100 MHz, Chloroform-*d*)**: δ 158.0, 156.1, 136.4, 133.3, 129.4, 128.7, 128.4, 128.3, 114.0, 81.6, 67.0, 63.5, 55.3, 37.8, 30.5, 15.3 ppm. **HRMS (ESI, m/z)**: calculated for $[M+H]^+$: 344.1862, found: 344.1866. **IR (KBr)**: 3303, 2953, 1706, 1512, 1245, 1178, 1037, 698 cm^{-1}.

benzyl (3-(4-methoxyphenyl)-1-((trifluoromethyl)sulfonamido)propyl)carbamate (40)

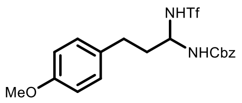

Following the General Procedure A, **40** was obtained in 51% yield as a white solid; **^{1}H NMR (400 MHz, DMSO-*d6*)**: δ 9.96 (d, *J* = 4.4 Hz, 1H), 8.22 (d, *J* = 8.6 Hz, 1H), 7.44 – 7.27 (m, 5H), 7.14 – 7.01 (m, 2H), 6.89 – 6.74 (m, 2H), 5.06 (s, 2H), 5.03 – 4.90 (m, 1H), 3.71 (s, 3H), 2.60 – 2.42 (m, 2H), 2.05 – 1.84 (m, 2H) ppm; **^{13}C NMR (100 MHz, DMSO-d6)**: δ158.1, 155.3, 137.9, 137.4, 132.81, 129.8, 128.9, 128.4, 128.3, 128.2, 119.9 (q, *J* = 321.9 Hz), 114.3, 66.1, 63.0, 55.5, 37.6, 30.7 ppm; **^{19}F NMR (377 MHz, DMSO-d6)**: δ -77.55 ppm. **HRMS (ESI, m/z):** calculated for $[M+H]^+$: 447.1202, found: 447.1211.

2,2,2-trifluoroethyl (1-ethoxy-3-(4-methoxyphenyl)propyl)carbamate (17)

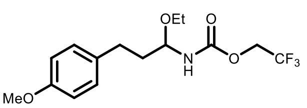

Following the General Procedure B, **17** was obtained in 59% yield as a white solid; **^{1}H NMR (400 MHz, Chloroform-*d*):** δ 7.09 (d, *J* = 8.4 Hz, 2H), 6.83 (d, *J* = 8.3 Hz, 2H), 5.19 (d, *J* = 9.9 Hz, 1H), 5.00 – 4.86 (m, 1H), 4.66 – 4.30 (m, 2H), 3.78 (s, 3H), 3.72 – 3.41 (m, 2H), 2.75 – 2.53 (m, 2H), 2.03 – 1.78 (m, 2H), 1.20 (t, *J* = 7.0 Hz, 3H) ppm; **^{13}C NMR (100 MHz, Chloroform-*d*):** δ 158.0, 154.2, 133.0, 129.4, 123.1 (q, *J* = 277.6 Hz), 114.0, 81.9, 63.8, 61.0 (q, *J* = 36.5 Hz), 55.3, 37.7, 30.3, 15.2 ppm. **HRMS (ESI, m/z):** calculated for $[M+H]^+$: 344.1862, found: 344.1866. **IR (KBr)**: 1732, 1514, 1285, 1247, 1175, 1052 cm^{-1}.

2,2,2-trifluoroethyl (3-(4-methoxyphenyl)-1-((trifluoromethyl)sulfonamido)propyl)carbamate (41)

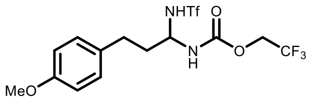

Following the General Procedure A, **41** was obtained in 43% yield as a white solid; **^{1}H NMR (400 MHz, DMSO-*d6*)**: δ 10.07 (s, 1H), 8.63 (d, *J* = 8.7 Hz, 1H), 7.10 (d, *J* = 8.4 Hz, 2H), 6.85 (d, *J* = 8.5 Hz, 2H), 5.02 – 4.89 (m, 1H), 4.80 – 4.58 (m, 2H), 3.71 (s, 3H), 2.60 – 2.44 (m, 2H), 2.03 – 1.86 (m, 2H) ppm; **^{13}C NMR (100 MHz, DMSO-*d6*)**: δ 158.1, 153.7, 132.7, 129.8, 124.2 (q, *J* = 277.2 Hz), 119.8 (q, *J* = 322.1 Hz), 114.3, 63.0, 60.3 (q, *J* = 34.3 Hz), 55.5, 37.4, 30.6 ppm; **^{19}F NMR (377 MHz, DMSO-d6)**: δ -72.77 , -77.69 ppm. **HRMS (ESI, m/z)**: calculated for $[M+H]^+$: 439.0762, found: 439.0765. **IR (KBr)**: 2555, 2128, 1652, 1184, 1026, 1004, 826, 764 cm^{-1}.

(1-ethoxypropane-1,3-diyl)dibenzene (38)

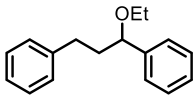

Following the General Procedure B, **38** was obtained in 88% yield as a colorless oil; **^{1}H NMR (400 MHz, Chloroform-*d*)**: δ 7.42 – 7.28 (m, 7H), 7.26 – 7.18 (m, 3H), 4.28 – 4.16 (m, 1H), 3.48 – 3.24 (m, 2H), 2.86 – 2.61 (m, 2H), 2.26 – 2.06 (m, 1H), 2.03 – 1.84 (m, 1H), 1.29 – 1.21 (m, 3H) ppm; **^{13}C NMR (100 MHz, Chloroform-*d*)**: δ 143.1, 142.1, 128.6, 128.5, 128.5, 127.5, 126.7, 125.9, 81.2, 64.3, 40.0, 32.2, 15.6 ppm. **HRMS (ESI, m/z)**: calculated for $[M+H]^+$: 241.1592, found: 241.1597. **IR (KBr)**: 2973, 1494, 1454, 1098, 747, 700 cm^{-1}.

N-(1,3-diphenylpropyl)-1,1,1-trifluoromethanesulfonamide (42)

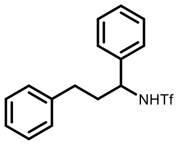

Following the General Procedure A, **42** was obtained in 55% yield as a colorless oil; **^{1}H NMR (400 MHz, Chloroform-*d*)**: δ 7.52 – 6.88 (m, 10H), 5.64 (d, *J* =8.1 Hz, 1H), 4.65 – 4.55 (m, 1H), 2.72 – 2.52 (m, 2H), 2.37 – 1.99 (m, 2H) ppm; **^{13}C NMR (100 MHz, Chloroform-*d*)**: δ 140.3, 139.9, 129.2, 128.7, 128.5, 128.4, 126.4, 126.3, 119.5 (q, *J* = 321.0 Hz), 59.7, 39.3, 32.2 ppm; **^{19}F NMR (377 MHz, Chloroform-d)**: δ -77.35 ppm. **HRMS (ESI, m/z)**: calculated for $[M+H]^+$: 344.0932, found: 344.0930. **IR (KBr)**: 3203, 1456, 1375, 1229, 1197, 1146, 699 cm^{-1}.

1-(1-ethoxy-3-phenylpropyl)-4-methoxybenzene (19)

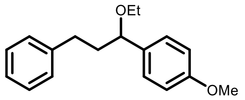

Following the General Procedure B, **19** was obtained in 60% yield as a colorless oil; **^{1}H NMR (400 MHz, Chloroform-*d*)**: δ 7.32 – 7.21 (m, 2H), 7.24 – 7.20 (m, 2H), 7.19 – 7.14 (m, 3H), 6.91 – 6.85 (m, 2H), 4.13 (dd, *J* = 7.9, 5.6 Hz, 1H), 3.80 (s, 3H), 3.40 – 3.31 (m, 1H), 3.31 – 3.22 (m, 1H), 2.76 – 2.67 (m, 1H), 2.67 – 2.58 (m, 1H), 2.19 – 2.08 (m, 1H), 1.96 – 1.85 (m, 1H), 1.18 (t, *J* = 7.0 Hz, 3H) ppm; **^{13}C NMR (100 MHz, Chloroform-*d*)**: δ 159.0, 142.2, 135.0, 128.6, 128.4, 127.9, 125.8, 113.8, 80.8, 63.9, 55.4, 39.8, 32.2, 15.5 ppm. **HRMS (ESI, m/z)**: calculated for $[M+H]^+$: 271.1698, found: 271.1702. **IR (KBr)**: 1610, 1511, 1248, 1092, 1036, 831 cm^{-1}.

1-(1-(4-methoxyphenyl)-3-phenylpropyl)-1H-pyrazole (43)

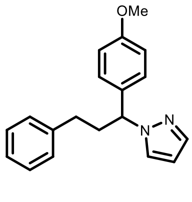

Following the General Procedure A, **43** was obtained in 42% yield as a colorless oil; **^{1}H NMR (400 MHz, Chloroform-*d*)**: δ 7.59 – 7.57 (m, 1H), 7.41 – 7.38 (m, 1H), 7.34 – 7.27 (m, 2H), 7.26 – 7.19 (m, 3H), 7.18 – 7.14 (m, 2H), 6.90 – 6.84 (m, 2H), 6.27 (t, *J* = 2.1 Hz, 1H), 5.22 (dd, *J* = 8.8, 6.5 Hz, 1H), 3.79 (s, 3H), 2.85 – 2.73 (m, 1H), 2.62 – 2.53 (m, 2H), 2.51 – 2.42 (m, 1H) ppm; **^{13}C NMR (100 MHz, Chloroform-*d*)**: δ 159.3, 141.1, 139.3, 132.8, 128.6, 128.6, 128.3, 128.2, 126.2, 114.1, 105.4, 64.6, 55.4, 37.0, 32.6 ppm. **HRMS (ESI, m/z)**: calculated for $[M+H]^+$: 293.1654, found: 293.1651. **IR (KBr)**: 2933, 1514, 1250, 1178, 1034, 751, 701 cm^{-1}.

1-(1-ethoxy-3-(4-(trifluoromethyl)phenyl)propyl)-4-methoxybenzene (20)

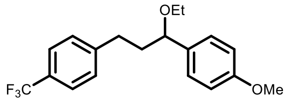

Following the General Procedure B, **20** was obtained in 40% yield as a colorless oil; **^{1}H NMR (400 MHz, Chloroform-*d*)**: δ 7.52 (d, *J* = 8.0 Hz, 2H), 7.28 (d, *J* = 7.9 Hz, 2H), 7.23 – 7.17 (m, 2H), 6.91 – 6.86 (m, 2H), 4.12 (dd, *J* = 8.0, 5.4 Hz, 1H), 3.81 (s, 3H), 3.41 – 3.31 (m, 1H), 3.31 – 3.21 (m, 1H), 2.84 – 2.63 (m, 2H), 2.20 – 2.07 (m, 1H), 1.96 – 1.85 (m, 1H), 1.18 (t, *J* = 7.0 Hz, 3H) pm; **^{13}C NMR (100 MHz, Chloroform-*d*):** δ 159.1, 146.3, 134.6, 128.9, 127.9, 124.5 (q, *J* = 271.7 Hz), 125.3, 125.3, 113.9, 80.6, 64.0, 55.4, 39.5, 32.1, 15.5 ppm. **HRMS (ESI, m/z):** calculated for $[M+H]^+$: 339.1572, found: 339.1577. **IR (KBr)**: 1613, 1512, 1326, 1249, 1163, 1123, 1068, 830 cm^{-1}.

1,1,1-trifluoro-N-(1-(4-methoxyphenyl)-3-(4-(trifluoromethyl)phenyl)propyl)methanesulfonamide (44)

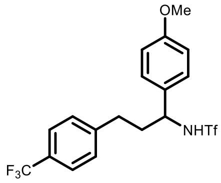

Following the General Procedure A, **44** was obtained in 45% yield as a colorless oil; **^{1}H NMR (400 MHz, Chloroform-*d*)**: δ 7.53 (d, *J* = 8.0 Hz, 2H), 7.23 (d, *J* = 8.0 Hz, 2H), 7.20 – 7.14 (m, 2H), 6.94 – 6.88 (m, 2H), 5.59 (d, *J* = 7.9 Hz, 1H), 4.58 – 4.47 (m, 1H), 3.81 (s, 3H), 2.73 – 2.55 (m, 2H), 2.33 – 2.22 (m, 1H), 2.22 – 2.12 (m, 1H) ppm; **^{13}C NMR (100 MHz, Chloroform-*d*)**: δ 159.8, 144.5, 131.4, 128.7, 127.6, 125.6, 125.6, 124.3 (q, *J* = 271.0 Hz), 119.5 (q, *J* = 321.0 Hz), 114.6, 59.2, 55.4, 38.9, 32.2 ppm; **^{19}F NMR (377 MHz, Chloroform-d)**: δ -62.27, -77.35 ppm. **HRMS (ESI, m/z)**: calculated for $[M+H]^+$: 442.0912, found: 442.0917. **IR (KBr)**: 3299, 2939, 1708, 1614, 1515, 1375, 1327, 831, 616 cm^{-1}.

1-(1-(4-methoxyphenyl)-3-(4-(trifluoromethyl)phenyl)propyl)-1H-pyrazole (45)

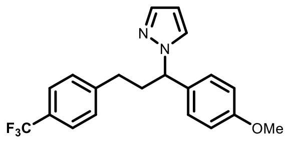

Following the General Procedure A, **45** was obtained in 52% yield as a colorless oil; **^{1}H NMR (400 MHz, Chloroform-*d*)**: δ 7.59 – 7.57 (m, 1H), 7.56 – 7.51 (m, 2H), 7.39 – 7.36 (m, 1H), 7.28 – 7.20 (m, 2H), 6.90 – 6.84 (m, 2H), 6.28 – 6.23 (m, 1H), 5.18 (dd, *J* = 8.9, 6.5 Hz, 1H), 3.78 (s, 3H), 2.88 – 2.76 (m, 1H), 2.65 – 2.58 (m, 2H), 2.51 – 2.40 (m, 1H) ppm; **^{13}C NMR (100 MHz, Chloroform-*d*)**: δ 159.4, 145.2, 139.4, 132.4, 128.9, 128.4, 128.2, 125.5, 125.5, 124.4 (q, *J* = 271.8 Hz), 114.2, 105.6, 64.6,

55.4, 36.7, 32.5 ppm. **HRMS (ESI, m/z)**: calculated for $[M+H]^+$: 361.1528, found: 361.1525. **IR (KBr)**: 2935, 1613, 1514, 1327, 1252, 1164, 1123, 1068, 831, 752 cm^{-1}.

2-ethoxy-2-ethyl-1,2,3,4-tetrahydronaphthalene (21)

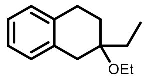

Following the General Procedure B, **21** was obtained in 54% yield as a colorless oil; **^{1}H NMR (400 MHz, Chloroform-*d*)**: δ 7.15 – 7.01 (m, 4H), 3.48 – 3.33 (m, 2H), 2.98 – 2.87 (m, 2H), 2.78 – 2.65 (m, 2H), 2.02 – 1.91 (m, 1H), 1.80 – 1.66 (m, 1H), 1.66 – 1.52 (m, 2H), 1.13 (t, *J* = 7.0 Hz, 3H), 0.93 (t, *J* = 7.4 Hz, 3H) ppm; **^{13}C NMR (100 MHz, Chloroform-*d*)**: δ 136.4, 135.2, 129.5, 128.5, 125.6, 125.6, 74.7, 55.9, 39.1, 30.9, 28.2, 26.4, 16.1, 7.3 ppm. **HRMS (ESI, m/z)**: calculated for $[M+H]^+$: 205.1592, found: 205.1595. **IR (KBr)**: 2925, 1770, 1454, 1246, 1066 cm^{-1}.

1,1,1-trifluoro-N-(1,2,3,4-tetrahydronaphthalen-2-yl)methanesulfonamide (46)

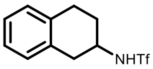

Following the General Procedure A, **46** was obtained in 44% yield as a colorless oil; **^{1}H NMR (400 MHz, Chloroform-*d*)**: δ 7.24 – 7.20 (m, 2H), 7.20 – 7.15 (m, 2H), 5.00 (br, 1H), 3.39 (t, *J* = 6.3 Hz, 2H), 3.20 – 3.08 (m, 2H), 2.78 – 2.62 (m, 3H) ppm; **^{13}C NMR (100 MHz, Chloroform-*d*)**: δ 141.5, 126.9, 124.8, 119.7 (q, *J* = 321.1 Hz), 48.8, 39.7, 36.5 ppm; **^{19}F NMR (377 MHz, Chloroform-d)**: δ -77.03 ppm. **HRMS (ESI, m/z)**: calculated for $[M+H]^+$: 280.0619, found: 280.0622. **IR (KBr)**: 2938, 1435, 1373, 1232, 1193, 1146, 745, 607 cm^{-1}.

7-ethoxy-6,7,8,9-tetrahydro-5H-benzo[7]annulene (22)

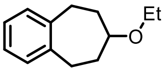

Following the General Procedure B, **22** was obtained in 62% yield as a colorless oil; **^{1}H NMR (400 MHz, Chloroform-*d*)**: δ 7.14 – 7.06 (m, 4H), 3.53 (q, *J* = 7.0 Hz, 2H), 3.46 – 3.34 (m, 2H), 2.95 – 2.86 (m, 1H), 2.86 – 2.79 (m, 2H), 2.49 (dd, *J* = 16.5, 10.6 Hz, 1H), 2.16 – 1.95 (m, 2H), 1.50 – 1.38 (m, 1H), 1.25 (t, *J* = 7.0 Hz, 3H) ppm; **^{13}C NMR (100 MHz, Chloroform-*d*)**: δ 137.0, 136.3, 129.3, 129.0, 125.6, 75.7, 66.6, 34.8, 33.0, 28.8, 26.5, 15.4 ppm. **HRMS (ESI, m/z)**: calculated for $[M+H]^+$: 191.1436, found: 191.1441. **IR (KBr)**: 2854, 1453, 1378, 1112, 743 cm^{-1}.

1,1,1-trifluoro-N-(6,7,8,9-tetrahydro-5H-benzo[7]annulen-7-yl)methanesulfonamide (47)

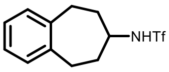

Following the General Procedure A, **47** was obtained in 50% yield as a colorless oil; **^{1}H NMR (400 MHz, Chloroform-*d*)**: δ 7.18 – 7.06 (m, 4H), 5.20 (br, 1H), 3.37 – 3.27 (m, 2H), 2.97 – 2.76 (m, 3H), 2.51 (dd, *J* = 16.2, 10.4 Hz, 1H), 2.13 – 1.95 (m, 2H), 1.54 – 1.40 (m, 1H) ppm; **^{13}C NMR (100 MHz, Chloroform-*d*)**: δ 136.1, 134.7, 129.3, 129.0, 126.2, 126.0, 119.8 (q, *J* = 321.1 Hz), 49.6, 35.1, 33.1, 28.4, 26.5 ppm; **^{19}F NMR (377 MHz, Chloroform-*d*)** δ -77.01 ppm. **HRMS (ESI, m/z)**: calculated for $[M+H]^+$: 294.0776, found: 294.0773. **IR (KBr)**: 1432, 1374, 1232, 1194, 1147, 607 cm^{-1}.

1-((2-ethoxycyclohexyl)methyl)-4-methoxybenzene (23)

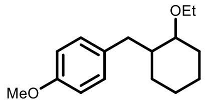

Following the General Procedure B, **23** was obtained in 31% yield as a colorless oil; **^{1}H NMR (400 MHz, Chloroform-*d*)**: δ 7.10 – 7.03 (m, 2H), 6.83 – 6.77 (m, 2H), 3.77 (s, 3H), 3.75 – 3.65 (m, 1H), 3.44 – 3.32 (m, 1H), 3.14 (dd, *J* = 13.4, 3.4 Hz, 1H), 2.90 – 2.80 (m, 1H), 2.21 (dd, *J* = 13.4, 9.5 Hz, 1H), 2.16 – 2.02 (m, 1H), 1.75 – 1.67 (m, 1H), 1.66 – 1.60 (m, 1H), 1.64 – 1.47 (m, 2H), 1.23 (t, *J* = 7.0 Hz, 3H), 1.20 – 1.12 (m, 2H), 1.12 – 1.00 (m, 1H), 0.93 – 0.80 (m, 1H) ppm; **^{13}C NMR (100 MHz, Chloroform-*d*)**: δ 157.7, 133.2, 130.4, 113.5, 81. 8, 64.0, 55.3, 45.3, 37.9, 31.5, 30.2, 25.5, 25.0, 15.9 ppm. **HRMS (ESI, m/z)**: calculated for $[M+H]^+$: 249.1855, found: 249.1857. **IR (KBr)**: 2929, 1511, 1245, 1106, 1038, 833 cm^{-1}.

1,1,1-trifluoro-N-(2-(4-methoxybenzyl)cyclohexyl)methanesulfonamide (48)

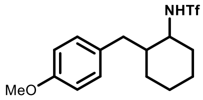

Following the General Procedure A, **48** was obtained in 45% yield as a colorless oil; **^{1}H NMR (400 MHz, Chloroform-*d*)**: δ 7.06 – 7.01 (m, 2H), 6.85 – 6.80 (m, 2H), 5.05 (d, *J* = 9.5 Hz, 1H), 3.79 (s, 3H), 3.32 – 3.20 (m, 1H), 3.16 (dd, *J* = 13.7, 3.6 Hz, 1H), 2.24 – 2.11 (m, 2H), 1.81 – 1.65 (m, 2H), 1.67 – 1.56 (m, 1H), 1.50 – 1.39 (m, 1H), 1.38 – 1.30 (m, 2H), 1.13 – 0.89 (m, 2H) ppm; **^{13}C NMR (100 MHz, Chloroform-*d*)**: δ 158.0, 131.8, 130.1, 119.7 (q, *J* = 320.7 Hz), 113. 9, 60.4, 55.4, 45.5, 38.2, 35.1, 30.6, 25.3, 24.9 ppm; **^{19}F NMR (377 MHz, Chloroform-d)**: δ -77.43 ppm. **HRMS (ESI, m/z)**: calculated for $[M+H]^+$: 352.1194, found: 352.1191. **IR (KBr)**: 2938, 2860, 1708, 1612, 1513, 1443, 1375, 1230, 1193, 1149, 1038, 613 cm^{-1}.

(4-ethoxybutane-1,1-diyl)dibenzene (24)

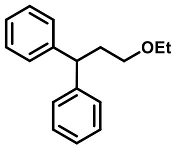

Following the General Procedure B, **24** was obtained in 52% yield as a colorless oil; **^{1}H NMR (400 MHz, Chloroform-*d*)**: δ 7.32 – 7.25 (m, 8H), 7.22 – 7.15 (m, 2H), 4.13 (t, *J* = 7.9 Hz, 1H), 3.42 (q, *J* = 7.0 Hz, 2H), 3.35 (t, *J* = 6.6 Hz, 2H), 2.42 – 2.28 (m, 2H), 1.20 (t, *J* = 7.0 Hz, 3H) ppm; **^{13}C NMR (100 MHz, Chloroform-*d*)**: δ 144.8, 128.5, 128.0, 126.3, 68.7, 66.3, 47.5, 35.5, 15.4 ppm. **HRMS (ESI, m/z)**: calculated for $[M+H]^+$: 241.1592, found: 241.1588. **IR (KBr)**: 2975, 2867, 1741, 1493, 1450, 1375, 1240, 1110, 700 cm^{-1}.

(3-ethoxy-3-(4-methoxyphenyl)propane-1,2-diyl)dibenzene (25)

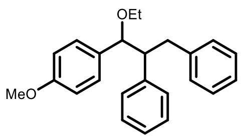

Following the General Procedure B, **25** was obtained in 43% yield as a white solid; **^{1}H NMR (400 MHz, Chloroform-*d*)**: δ 7.21 – 6.99 (m, 10H), 6.98 – 6.90 (m, 2H), 6.80 – 6.70 (m, 2H), 4.41 – 4.32 (m, 1H), 3.78 (s, 3H), 3.36 – 3.29 (m, 1H), 3.22 – 3.15 (m, 1H), 3.13 – 3.04 (m, 2H), 2.90 – 2.80 (m, 1H), 1.10 (t, *J* = 7.0 Hz, 3H) ppm; **^{13}C NMR (100 MHz, Chloroform-*d*)**: δ 158.8, 141.4, 140.8, 133.4, 129. 5, 129.2, 128.5, 128.1, 127.6, 126.1, 125.8, 113.3, 83.3, 64.2, 55.3, 55.2, 38.8, 15.3 ppm. **HRMS (ESI, m/z)**: calculated for $[M+H]^+$: 347.2011, found: 347.2013. **IR (KBr)**: 1511, 1453, 1248, 1095, 699 cm^{-1}.

2-(4-methoxyphenethyl)tetrahydrofuran (26)

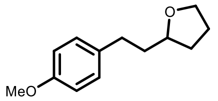

Following the General Procedure D, **26** was obtained in 78% yield as a colorless oil; **^{1}H NMR (400 MHz, Chloroform-*d*)**: δ 7.13 (d, *J* = 8.4 Hz, 2H), 6.83 (d, *J* = 8.1 Hz, 2H), 3.93 – 3.85 (m, 1H), 3.85 – 3.77 (m, 4H) , 3.77 – 3.70 (m, 1H), 2.78 – 2.66 (m, 1H), 2.65 – 2.55 (m, 1H), 2.03 – 1.67 (m, 5H), 1.53 – 1.40 (m, 1H) ppm; **^{13}C NMR (100 MHz, Chloroform-*d*)**: δ 157.8, 134.3, 129.4, 113.8, 78.7, 67.8, 55.3, 37.8, 31.9, 31. 5, 25.8 ppm. **HRMS (ESI, m/z)**: calculated for $[M+H]^+$: 207.1385, found: 207.1388. **IR (KBr)**: 1740, 1512, 1373, 1245, 1177, 1040 cm^{-1}.

(E)-1-(6-ethoxy-6-methylhept-3-en-4-yl)-4-methoxybenzene (112)

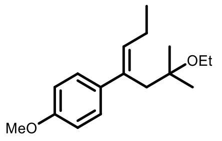

Following the General Procedure A, **112** was obtained in 88% yield as a colorless oil (alkene isomeric ratio = 3:1); Major isomer **^{1}H NMR (400 MHz, Chloroform-*d*)**: δ 7.25 – 7.20 (m, 2H), 6.84 – 6.79 (m, 2H), 5.55 (t, *J* = 7.2 Hz, 1H), 3.79 (s, 3H), 3.34 (q, *J* = 7.0 Hz, 2H), 2.73 (s, 2H), 2.22 (p, *J* = 7.4 Hz, 2H), 1.06 – 0.91 (m, 12H) ppm; **^{13}C NMR (100 MHz, Chloroform-*d*)**: δ 158.3, 138.2, 136.4, 134.0, 127.8, 113.5, 76.3, 56.6, 55.3, 40.1, 26.1, 22.8, 16.1, 14.3 ppm; Minor isomer **^{1}H NMR (400 MHz, Chloroform-*d*)**: δ 7.11 – 7.07 (m, 2H), 6.87 – 6.83 (m, 2H), 5.46 (t, *J* = 7.3 Hz, 1H), 3.80 (s, 3H), 3.26 (q, *J* = 7.0 Hz, 2H), 2.56 (s, 2H), 2.00 (p, *J* = 7.4 Hz, 2H), 1.06 – 0.91 (m, 12H) ppm; **^{13}C NMR (100 MHz, Chloroform-*d*)**: δ 158.0, 136.3, 134.4, 133.1, 129.8, 113.2, 75.4, 56.4, 55.3, 48.9, 26.2, 22.5, 16.0, 14.8 ppm. **HRMS (ESI, m/z)**: calculated for $[M+H]^+$: 263.2011, found: 263.2015. **IR (KBr)**: 2973, 1770, 1601, 1511, 1247, 832 cm^{-1}.

1-methoxy-4-(3-methoxy-3-methylbutyl)benzene (49)

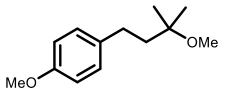

Following the General Procedure B, **49** was obtained in 89% yield as a colorless oil; **^{1}H NMR (400 MHz, Chloroform-*d*)**: δ 7.14 – 7.09 (m, 2H), 6.86 – 6.79 (m, 2H), 3.79 (s, 3H), 3.23 (s, 3H), 2.65 – 2.49 (m, 2H), 1.82 – 1.67 (m, 2H), 1.21 (s, 6H) ppm; **^{13}C NMR (100 MHz, Chloroform-*d*)**: δ 157.7, 135.0, 129.2, 113.9, 74.5, 55.4, 49.3, 42.0, 29.4, 25.2 ppm. **HRMS (ESI, m/z)**: calculated for $[M+H]^+$: 209.1542, found: 209.1546. **IR (KBr)**: 2972, 1717, 1513, 1100, 836 cm^{-1}.

1-(3-isopropoxy-3-methylbutyl)-4-methoxybenzene (50)

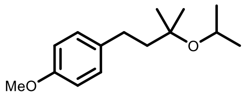

Following the General Procedure B, **50** was obtained in 90% yield as a colorless oil; **^{1}H NMR (400 MHz, Chloroform-*d*)**: δ 7.13 – 7.08 (m, 2H), 6.84 – 6.79 (m, 2H), 3.85 – 3.78 (m, 1H), 3.78 (s, 3H), 2.78 – 2.49 (m, 2H), 1.82 – 1.59 (m, 2H), 1.21 (s, 6H), 1.14 (d, *J* = 6.1 Hz, 6H) ppm; **^{13}C NMR (100 MHz, Chloroform-*d*)**: δ 157.7, 135.2, 129.2, 113.8, 75.0, 63.3, 55.3, 43.5, 29.8, 26.4, 25.3 ppm. **HRMS (ESI, m/z)**: calculated for $[M+H]^+$: 237.1855, found: 237.1857. **IR (KBr)**: 2971, 2934, 1612, 1513, 1247, 1176, 1115, 1039, 1007, 822 cm^{-1}.

((5-((4-([1,1'-biphenyl]-4-yl)-2-methylbutan-2-yl)oxy)pentyl)oxy)(tert-butyl)dimethylsilane (51)

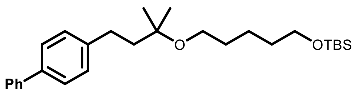

Following the General Procedure C, **51** was obtained in 56% yield as a colorless oil; **^{1}H NMR (400 MHz, Chloroform-*d*)**: δ 7.61 – 7.56 (m, 2H), 7.54 – 7.49 (m, 2H), 7.46 – 7.40 (m, 2H), 7.36 – 7.30 (m, 1H), 7.30 – 7.24 (m, 2H), 3.63 (t, *J* = 6.5 Hz, 2H), 3.37 (t, *J* = 6.6 Hz, 2H), 2.74 – 2.63 (m, 2H), 1.85 – 1.75 (m, 2H), 1.63 – 1.51 (m, 4H), 1.47 – 1.36 (m, 2H), 1.23 (s, 6H), 0.90 (s, 9H), 0.07 – 0.03 (m, 6H) ppm; **^{13}C NMR (100 MHz, Chloroform-*d*)**: δ 142.3, 141.2, 138.7, 128.8, 128.8, 127.2, 127.1, 74.1, 63.3, 61.3, 42.2, 32.9, 30.5, 30.0, 26.1, 25.9, 22.8, 18.5, -5.2 ppm. **HRMS (ESI, m/z)**: calculated for $[M+H]^+$: 441.3189, found: 441.3192. **IR (KBr)**: 2935, 1743, 1247, 1098, 1048, 836 cm^{-1}.

tert-butyl(2-((4-(4-methoxyphenyl)-2-methylbutan-2-yl)oxy)ethoxy)dimethylsilane (52)

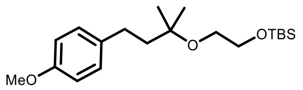

Following the General Procedure C, **52** was obtained in 60% yield as a colorless oil; **^{1}H NMR (400 MHz, Chloroform-*d*)**: δ 7.14 – 7.09 (m, 2H), 6.85 – 6.81 (m, 2H), 3.79 (s, 3H), 3.75 (t, *J* = 5.9 Hz, 2H), 3.44 (t, *J* = 5.9 Hz, 2H), 2.68 – 2.51 (m, 2H), 1.86 – 1.67 (m, 2H), 1.21 (s, 6H), 0.91 (s, 9H), 0.09 (s, 6H) ppm; **^{13}C NMR (100 MHz, Chloroform-*d*):** δ 157.7, 135.1, 129.3, 113.8, 74.4, 63.4, 63.0, 55.4, 42.6, 29.4, 26.1, 25.8, 18.6, -5.1 ppm. **HRMS (ESI, m/z):** calculated for $[M+H]^+$: 353.2512, found: 353.2515. **IR (KBr)**: 2930, 1513, 1248, 1133, 1100, 834, 777 cm^{-1}.

tert-butyl (2-((4-(4-methoxyphenyl)-2-methylbutan-2-yl)oxy)ethyl)carbamate (53)

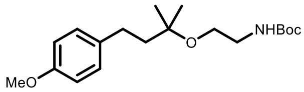

Following the General Procedure C, **53** was obtained in 54% yield as a colorless oil; **^{1}H NMR (400 MHz, Chloroform-*d*)**: δ 7.13 – 7.06 (m, 2H), 6.85 – 6.79 (m, 2H), 4.92 (br, 1H), 3.78 (s, 3H), 3.45 – 3.37 (m, 2H), 3.33 – 3.21 (m, 2H), 2.62 – 2.51 (m, 2H), 1.80 – 1.64 (m, 2H), 1.44 (s, 9H), 1.20 (s, 6H) ppm; **^{13}C NMR (100 MHz, Chloroform-*d*)**: δ 157.7, 156.1, 134.8, 129.2, 113.9, 79.3, 74.7, 60.5, 55.4, 42.5, 41.2, 29.4, 28.5, 25.7 ppm. **HRMS (ESI, m/z)**: calculated for $[M+H]^+$: 338.2331, found: 338.2333. **IR (KBr)**: 2975, 1716, 1513, 1365, 1247, 1176, 1099, 1039 cm^{-1}.

1-methoxy-4-(3-methyl-3-((4-(trifluoromethyl)benzyl)oxy)butyl)benzene (54)

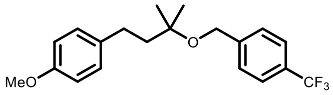

Following the General Procedure C, **54** was obtained in 92% yield as a colorless oil; **^{1}H NMR (400 MHz, Chloroform-*d*)**: δ 7.60 (d, *J* = 8.2 Hz, 2H), 7.49 (d, *J* = 8.1 Hz, 2H), 7.16 – 7.08 (m, 2H), 6.87 – 6.80 (m, 2H), 4.53 (s, 2H), 3.79 (s, 3H), 2.77 – 2.58 (m, 2H), 1.90 – 1.81 (m, 2H), 1.33 (s, 6H) ppm; **^{13}C NMR (100 MHz, Chloroform-*d*)**: δ 157.8, 144.1, 134.8, 129.2, 127.3, 125.3, 125.3, 124.4 (q, *J* = 271.9 Hz), 113.9, 75.5, 63.1, 55.4, 42.8, 29.5, 25.7 ppm; **^{19}F NMR (377 MHz, Chloroform-d)**: δ -62.27 ppm. **HRMS (ESI, m/z)**: calculated for $[M+H]^+$: 353.1728, found: 353.1733. **IR (KBr)**: 2972, 1513, 1327, 1247, 1163, 1124, 1066, 1018, 823 cm^{-1}.

1-(tert-butyl)-4-(3-methyl-3-((4-(trifluoromethyl)benzyl)oxy)butyl)benzene (55)

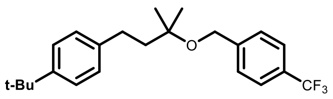

Following the General Procedure C, **55** was obtained in 90% yield as a colorless oil; **^{1}H NMR (400 MHz, Chloroform-*d*)**: δ 7.60 (d, *J* = 8.1 Hz, 2H), 7.49 (d, *J* = 8.0 Hz, 2H), 7.36 – 7.29 (m, 2H), 7.18 – 7.12 (m, 2H), 4.53 (s, 2H), 2.76 – 2.65 (m, 2H), 1.94 – 1.86 (m, 2H), 1.34 (s, 6H), 1.32 (s, 9H) ppm; **^{13}C NMR (100 MHz, Chloroform-*d*)**: δ 148.7, 144.1, 139.6, 128.0, 127.3, 125.4, 125.3, 125.3, 124.3 (q, *J* = 271.8 Hz), 75.5, 63.1, 42.3, 34.5, 31.5, 29.8, 25.8 ppm; **^{19}F NMR (377 MHz, Chloroform-d)**: δ -62.26 ppm. **HRMS (ESI, m/z)**: calculated for $[M+H]^+$: 379.2249, found: 379.2255. **IR (KBr)**: 2967, 1326, 1167, 1128, 1067, 1018 cm^{-1}.

5-(((4-(4-methoxyphenyl)-2-methylbutan-2-yl)oxy)methyl)benzo[d][1,3]dioxole (56)

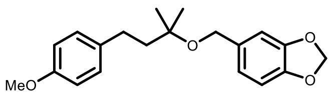

Following the General Procedure C, **56** was obtained in 70% yield as a colorless oil; **^{1}H NMR (400 MHz, Chloroform-*d*)**: δ 7.17 – 7.10 (m, 2H), 6.93 – 6.75 (m, 5H), 5.94 (s, 2H), 4.37 (s, 2H), 3.80 (s, 3H), 2.72 – 2.59 (m, 2H), 1.90 – 1.79 (m, 2H), 1.32 (s, 6H) ppm; **^{13}C NMR (100 MHz, Chloroform-*d*)**: δ 157.8, 147.8, 146.8, 135.0, 133.7, 129.3, 120.8, 113.9, 108.4, 108.2, 101.0, 75.1, 63.8, 55.4, 42.7, 29.5, 25.9 ppm. **HRMS (ESI, m/z)**: calculated for $[M+H]^+$: 329.1753, found: 329.1760. **IR (KBr)**: 2970, 1611, 1512, 1491, 1443, 1247, 1098, 1039, 932, 809 cm^{-1}.

4-(4-methoxyphenyl)-2-methylbutan-2-ol (57)

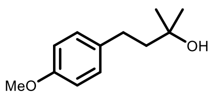

Following the General Procedure B, **57** was obtained in 77% yield as a colorless oil; **^{1}H NMR (400 MHz, Chloroform-*d*)**: δ 7.15 – 7.08 (m, 2H), 6.86 – 6.80 (m, 2H), 3.97 – 3.90 (m, 1H), 3.78 (s, 3H), 2.73 – 2.54 (m, 2H), 1.81 – 1.65 (m, 2H), 1.28 (s, 6H) ppm; **^{13}C NMR (100 MHz, Chloroform-*d*)**: δ 157.8, 134.6, 129.3, 113.9, 71.0, 55.4, 46.1, 29.9, 29.4 ppm. **HRMS (ESI, m/z)**: calculated for $[M+H]^+$: 195.1385, found: 195.1381. **IR (KBr)**: 2970, 1742, 1374, 1240, 1046 cm^{-1}.

2-methyl-4-(p-tolyl)butan-2-ol (58)

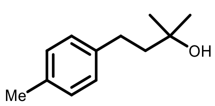

Following the General Procedure G, **58** was obtained in 61% yield as a colorless oil; **^{1}H NMR (400 MHz, Chloroform-*d*)**: δ 7.11 (s, 4H), 2.74 – 2.60 (m, 2H), 2.33 (s, 3H), 1.84 – 1.71 (m, 2H), 1.29 (s, 6H) ppm; **^{13}C NMR (100 MHz, Chloroform-*d*)**: δ 139.4, 135.2, 129.1, 128.2, 70.9, 45.9, 30.3, 29.3, 20.9 ppm. **HRMS (ESI, m/z)**: calculated for $[M+Na]^+$: 201.1250, found: 201.1257. **IR (KBr)**: 2970, 2927, 1742, 1515, 1374, 1240, 1046, 809 cm^{-1}.

3,3-diphenylpropan-1-ol (59)

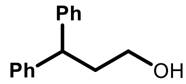

Following the General Procedure G, **59** was obtained in 75% yield as a colorless oil; **^{1}H NMR (400 MHz, Chloroform-*d*)**: δ 7.34 – 7.22 (m, 8H), 7.21 – 7.14 (m, 2H), 4.13 (t, *J* = 7.9 Hz, 1H), 3.61 (t, *J* = 6.4 Hz, 2H), 2.38 – 2.35 (m, 2H) ppm; **^{13}C NMR (100 MHz, Chloroform-*d*)**: δ 144.4,

128.5, 127.8, 126.3, 61.1, 47.3, 38.2 ppm. **HRMS (ESI, m/z)**: calculated for $[M+Na]^+$: 235.1093, found: 235.1088. **IR (KBr)**: 2984, 1741, 1374, 1240, 1046, 700 cm^{-1}.

(E)-1-(3-(but-2-en-1-yloxy)-3-methylbutyl)-4-methoxybenzene (60)

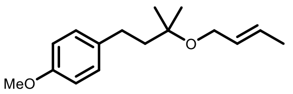

Following the General Procedure C, **60** was obtained in 83% yield as a colorless oil; **^{1}H NMR (400 MHz, Chloroform-*d*)**: δ 7.15 – 7.08 (m, 2H), 6.86 – 6.80 (m, 2H), 5.78 – 5.67 (m, 1H), 5.65 – 5.56 (m, 1H), 3.85 (d, *J* = 6.0 Hz, 2H), 3.79 (s, 3H), 2.74 – 2.41 (m, 2H), 1.80 – 1.74 (m, 2H), 1.74 – 1.69 (m, 3H), 1.24 (s, 6H) ppm; **^{13}C NMR (100 MHz, Chloroform-*d*)**: δ 157.7, 135.0, 129.3, 128.8, 128.4, 113.9, 74.7, 62.5, 55.4, 42.2, 29.5, 26.0, 18.0 ppm. **HRMS (ESI, m/z)**: calculated for $[M+H]^+$: 249.1855, found: 249.1849. **IR (KBr)**: 2969, 1612, 1513, 1247, 1177, 1039, 966, 821 cm^{-1}.

1-methoxy-4-(3-methyl-3-((2-methylallyl)oxy)butyl)benzene (61)

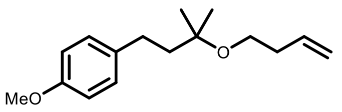

Following the General Procedure C, **61** was obtained in 78% yield as colorless oil; **^{1}H NMR (400 MHz, Chloroform-*d*)**: δ 7.15 – 7.08 (m, 2H), 6.87 – 6.81 (m, 2H), 5.94 – 5.82 (m, 1H), 5.15 – 5.08 (m, 1H), 5.07 – 5.02 (m, 1H), 3.79 (s, 3H), 3.41 (t, *J* = 6.9 Hz, 2H), 2.66 – 2.51 (m, 2H), 2.37 – 2.27 (m, 2H), 1.79 – 1.72 (m, 2H), 1.22 (s, 6H) ppm; **^{13}C NMR (100 MHz, Chloroform-*d*)**: δ 157.7, 135.9, 135.1, 129.3, 116.2, 113.9, 74.3, 60.9, 55.4, 42.5, 35.3, 29.4, 25.8 ppm. **HRMS (ESI, m/z)**: calculated for $[M+H]^+$: 249.1855, found: 249.1858. **IR (KBr)**: 2972, 1513, 1247, 1177, 1077, 1039, 822 cm^{-1}.

2-(((4-(4-methoxyphenyl)-2-methylbutan-2-yl)oxy)methyl)furan (62)

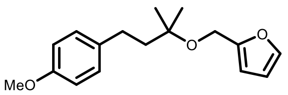

Following the General Procedure C, **62** was obtained in 59% yield as a colorless oil; **^{1}H NMR (400 MHz, Chloroform-*d*)**: δ 7.45 – 7.38 (m, 1H), 7.18 – 7.10 (m, 2H), 6.88 – 6.80 (m, 2H), 6.39 – 6.33 (m, 1H), 6.33 – 6.27 (m, 1H), 4.42 (s, 2H), 3.80 (s, 3H), 2.70 – 2.58 (m, 2H), 1.88 – 1.77 (m, 2H), 1.31 (s, 6H) ppm; **^{13}C NMR (100 MHz, Chloroform-*d*)**: δ 157.8, 153.0, 142.5, 135.0, 129.3, 113.9, 110.4, 108.3, 75.4, 56.7, 55.4, 42.3, 29.4, 25.9 ppm. **HRMS (ESI, m/z)**: calculated for $[M+H]^+$: 275.1647, found: 275.1655. **IR (KBr)**: 2971, 1513, 1247, 1177, 1056, 1038, 820, 739 cm^{-1}.

2-(((4-(4-methoxyphenyl)-2-methylbutan-2-yl)oxy)methyl)thiophene (63)

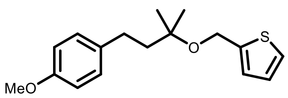

Following the General Procedure C, **63** was obtained in 46% yield as a colorless oil; **^{1}H NMR (400 MHz, Chloroform-*d*)**: δ 7.27 – 7.24 (m, 1H), 7.15 – 7.10 (m, 2H), 7.00 – 6.94 (m, 2H), 6.86 – 6.79 (m, 2H), 4.63 (s, 2H), 3.79 (s, 3H), 2.75 – 2.52 (m, 2H), 1.96 – 1.72 (m, 2H), 1.31 (s, 6H) ppm; **^{13}C NMR (100 MHz, Chloroform-*d*)**: δ 157.7, 143.1, 135.0, 129.3, 126.6, 125.1, 124.9, 113.9, 75.6, 59.2, 55.4, 42.6, 29.5, 25.9 ppm. **HRMS (ESI, m/z)**: calculated for $[M+H]^+$: 291.1419, found: 291.1425. **IR (KBr)**: 2932, 1512, 1246, 1037, 821, 700 cm^{-1}.

tert-butyl 2-(((4-(4-methoxyphenyl)-2-methylbutan-2-yl)oxy)methyl)pyrrolidine-1-carboxylate (64)

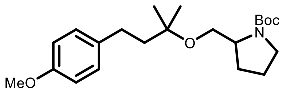

Following the General Procedure C, **64** was obtained in 62% yield as a colorless oil, mixture of two rotation isomers. **^{1}H NMR (400 MHz, Chloroform-*d*)**: δ 7.09 (d, *J* = 7.4 Hz, 2H), 6.83 – 6.77 (m, 2H), 3.98 – 3.78 (m, 1H), 3.77 (s, 3H), 3.53 – 3.42 (m, 1H), 3.40 – 3.10 (m, 3H), 2.76 – 2.45 (m, 2H), 2.01 – 1.74 (m, 4H), 1.74 – 1.65 (m, 2H), 1.44 (s, 9H), 1.17 (s, 6H) ppm; **Major isomer ^{13}C NMR (100 MHz, Chloroform-*d*)**: δ 157.7, 154.7, 135.0, 129.2, 113.8, 79.2, 74.3, 62.3, 55.4, 46.6, 43.0, 29.5, 28.9, 28.7, 25.7, 23.0 ppm; **Minor isomer ^{13}C NMR (101 MHz, Chloroform-*d*)**: δ 157.7, 154.7, 135.3, 129.2, 113.8, 78.9, 74.3, 61.9, 57.4, 47.1, 43.3, 29.5, 28.7, 28.1, 25.5, 24.0 ppm. **HRMS (ESI, m/z):** calculated for $[M+H]^+$: 353.2512, found: 353.2515. **IR (KBr)**: 2973, 1694, 1513, 1394, 1365, 1247, 1176, 1105 cm^{-1}.

1-(3-(((1R,2S,5R)-2-isopropyl-5-methylcyclohexyl)oxy)-3-methylbutyl)-4-methoxybenzene (65)

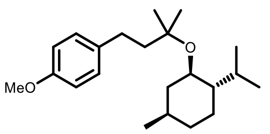

Following the General Procedure C, **65** was obtained in 64% yield as a colorless oil; **^{1}H NMR (400 MHz, Chloroform-*d*)**: δ 7.16 – 7.09 (m, 2H), 6.87 – 6.80 (m, 2H), 3.79 (s, 3H), 3.28 (td, *J* = 10.2, 4.2 Hz, 1H), 2.72 – 2.55 (m, 2H), 2.35 – 2.24 (m, 1H), 2.05 – 1.96 (m, 1H), 1.85 – 1.60 (m, 4H), 1.42 – 1.31 (m, 1H), 1.24 (d, *J* = 5.4 Hz, 6H), 1.18 – 1.10 (m, 1H), 1.07 – 0.94 (m, 2H), 0.91 (t, *J* = 6.9 Hz, 6H), 0.87 – 0.79 (m, 1H), 0.74 (d, *J* = 6.9 Hz, 3H) ppm; **^{13}C NMR (100 MHz, Chloroform-*d*)**: δ 157.7, 135.2, 129.3, 113.8, 75.0, 71.4, 55.3, 49.4, 45.6, 45.4, 34.7, 31.9, 29.9, 27.4, 26.2, 24.8, 23.3, 22.7, 21.8, 16.3 ppm. **HRMS (ESI, m/z)**: calculated for $[M+H]^+$: 333.2794, found: 333.2799. **IR (KBr)**: 2953, 1512, 1456, 1246, 1177, 1044 cm^{-1}.

1-((3S,8S,9S,10R,13S,14S,17S)-3-((4-(4-methoxyphenyl)-2-methylbutan-2-yl)oxy)-10,13-dimethyl-2,3,4,7,8,9,10,11,12,13,14,15,16,17-tetradecahydro-1H-cyclopenta[a]phenanthren-17-yl)ethan-1-one (66)

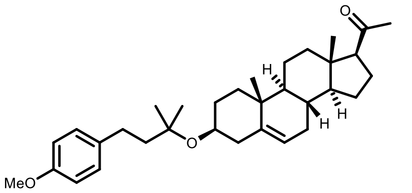

Following the General Procedure C, **66** was obtained in 66% yield as a white solid; **^{1}H NMR (400 MHz, Chloroform-*d*)**: δ 7.14 – 7.08 (m, 2H), 6.86 – 6.79 (m, 2H), 5.32 – 5.27 (m, 1H), 3.78 (s, 3H), 3.42 – 3.28 (m, 1H), 2.66 – 2.56 (m, 2H), 2.56 – 2.48 (m, 1H), 2.37 – 2.25 (m, 1H), 2.25 – 2.12 (m, 2H), 2.12 (s, 3H), 2.09 – 1.93 (m, 2H), 1.87 – 1.79 (m, 1H), 1.80 – 1.38 (m, 12H), 1.21 (s, 6H), 1.18 – 1.06 (m, 2H), 1.00 (s, 3H), 1.02 – 0.90 (m, 1H), 0.62 (s, 3H) ppm; **^{13}C NMR (100 MHz, Chloroform-*d*)**: δ 209.7, 157.7, 142.0, 135.0, 129.2, 120.8, 113.8, 75.3, 71.2, 63.8, 57.0, 55.3, 50.2, 44.1, 43.4, 42.2, 39.0, 37.9, 36.7, 32.0, 31.7, 31.5, 29.8, 26.6, 26.6, 24.6, 22.9, 21.2, 19.5, 13.3 ppm. **HRMS (ESI, m/z)**: calculated for $[M+H]^+$: 493.3682, found: 493.3686. **IR (KBr)**: 2937, 1742, 1700, 1514, 1364, 1247 cm^{-1}.

(3S,8S,9S,10R,13R,14S,17R)-3-((4-(4-methoxyphenyl)-2-methylbutan-2-yl)oxy)-10,13-dimethyl-17-((R)-6-methylheptan-2-yl)-2,3,4,7,8,9,10,11,12,13,14,15,16,17-tetradecahydro-1H-cyclopenta[a]phenanthrene (67)

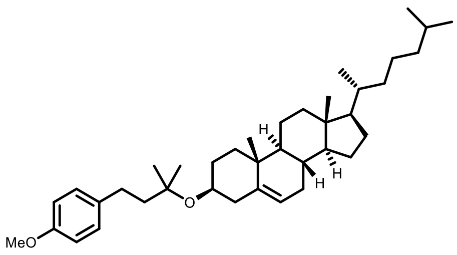

Following the General Procedure C, **67** was obtained in 31% yield as a white solid; **^{1}H NMR (400 MHz, Chloroform-*d*)**: δ 7.11 (d, *J* = 8.2 Hz, 2H), 6.82 (d, *J* = 8.2 Hz, 2H), 5.40 – 5.19 (m, 1H), 3.78 (s, 3H), 3.42 – 3.29 (m, 1H), 2.69 – 2.53 (m, 2H), 2.39 – 2.25 (m, 1H), 2.23 – 2.12 (m, 1H), 2.05 – 1.91 (m, 2H), 1.89 – 1.78 (m, 2H), 1.75 – 1.65 (m, 3H), 1.64 – 1.41 (m, 10H), 1.43 – 1.24 (m, 5H), 1.21 (s, 6H), 1.17 – 1.05 (m, 6H), 1.00 (s, 3H), 0.91 (d, *J* = 6.3 Hz, 3H), 0.86 (d, *J* = 6.5 Hz, 6H), 0.67 (s, 3H) ppm; **^{13}C NMR (100 MHz, Chloroform-*d*)**: δ 157.7, 142.0, 135.1, 129.3, 121.1, 113.8, 75.3, 71.3, 56.9, 56.2, 55.4, 50.4, 43.4, 42.4, 42.2, 39.9, 39.6, 37.9, 36.7, 36.3, 35.9, 32.0, 32.0, 31.5, 29.8, 28.3, 28.1, 26.6, 26.6, 24.4, 23.9, 22.9, 22.7, 21.1, 19.5, 18.8, 12.0 ppm. **HRMS (ESI, m/z)**: calculated for $[M+H]^+$: 563.4828, found: 563.4833. **IR (KBr)**: 2931, 1770, 1759, 1512, 1247, 1062 cm^{-1}.

1,5-bis((4-(4-methoxyphenyl)-2-methylbutan-2-yl)oxy)pentane (68)

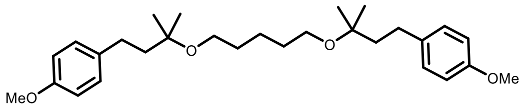

Following the given procedure, **68** was obtained in 61% yield as a colorless oil; **^{1}H NMR (400 MHz, Chloroform-*d*)**: δ 7.14 – 7.08 (m, 4H), 6.86 – 6.79 (m, 4H), 3.78 (s, 6H), 3.36 (t, *J* = 6.6 Hz, 4H), 2.73 – 2.38 (m, 4H), 1.80 – 1.67 (m, 4H), 1.64 – 1.54 (m, 4H), 1.49 – 1.41 (m, 2H), 1.21 (s, 12H) ppm; **^{13}C NMR (100 MHz, Chloroform-*d*)**: δ 157.7, 135.2, 129.2, 113.8, 74.1, 61.3, 55.4, 42.5, 30.7, 29.5, 25.9, 23.3ppm. **HRMS (ESI, m/z)**: calculated for $[M+H]^+$: 457.3318, found: 457.3315. **IR (KBr)**: 2935, 1512, 1246, 1176, 1038, 820 cm^{-1}.

1-(2-((4-(4-methoxyphenyl)-2-methylbutan-2-yl)oxy)ethyl)-4-methyl-N-(4-(methylsulfonyl)phenyl)-5-(2-(trifluoromethyl)phenyl)-1H-pyrrole-3-carboxamide (69)

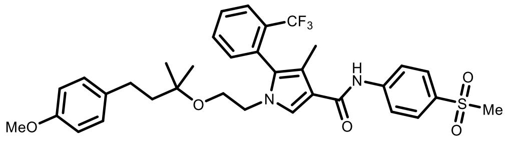

Following the General Procedure C, **69** was obtained in 27 % yield as a white solid; **^{1}H NMR (400 MHz, Chloroform-*d*)**: δ 7.89 – 7.83 (m, 2H), 7.83 – 7.78 (m, 1H), 7.76 – 7.70 (m, 2H), 7.66 (s, 1H), 7.64 – 7.54 (m, 2H), 7.46 (s, 1H), 7.38 – 7.32 (m, 1H), 7.07 – 7.00 (m, 2H), 6.81 – 6.74 (m, 2H), 3.83 – 3.70 (m, 4H), 3.67 – 3.56 (m, 1H), 3.50 – 3.32 (m, 2H), 3.04 (s, 3H), 2.54 – 2.42 (m, 2H), 2.08 (s, 3H), 1.78 – 1.64 (m, 2H), 1.14 (s, 6H) ppm; **^{13}C NMR (100 MHz, Chloroform-*d*)**: δ 163.9, 157.8, 143.7, 134.6, 134.5, 134.3, 131.9, 130.1, 129.5, 129.2, 129.1, 128.8, 126.5, 126.5, 126.4 (q, *J* = 274.4 Hz), 124.3, 119.5, 118.3, 117.3, 113.9, 75.17, 61.0, 55.4, 48.1, 44.9, 42.3, 29.2, 25.5, 25.5, 11.4 ppm. **HRMS (ESI, m/z)**: calculated for $[M+H]^+$: 643.2454, found: 643.2453. **IR (KBr)**: 2984, 1740, 1513, 1374, 1315, 1245, 1147, 1047 cm^{-1}.

(5R,5aS,8aS,8bR)-5-(((4-(4-methoxyphenyl)-2-methylbutan-2-yl)oxy)methyl)-2,2,7,7-tetramethyltetrahydro-5H-bis([1,3]dioxolo)[4,5-b:4',5'-d]pyran (70)

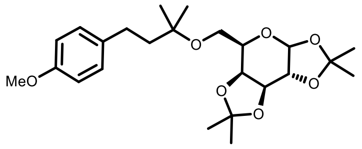

Following the General Procedure C, **70** was obtained in 77% yield as a colorless oil; **^{1}H NMR (400 MHz, Chloroform-*d*)**: δ 7.16 – 7.09 (m, 2H), 6.86 – 6.79 (m, 2H), 5.54 (d, *J* = 5.0 Hz, 1H), 4.62 – 4.56 (m, 1H), 4.37 – 4.32 (m, 1H), 4.31 – 4.27 (m, 1H), 3.94 – 3.88 (m, 1H), 3.78 (s, 3H), 3.63 – 3.56 (m, 1H), 3.54 – 3.47 (m, 1H), 2.69 – 2.52 (m, 2H), 1.84 – 1.63 (m, 2H), 1.55 (s, 3H), 1.46 (s, 3H), 1.33 (s, 6H), 1.22 (s, 6H) ppm; **^{13}C NMR (100 MHz, Chloroform-*d*)**: δ 157.7, 135.3, 129.3, 113.8, 109.0, 108.5, 96.4, 74.8, 71.0, 70.9, 70.6, 67.4, 60.2, 55.3, 42.5, 29.2, 26.3, 26.1, 25.9, 25.7, 25.1, 24.4 ppm. **HRMS (ESI, m/z)**: calculated for $[M+H]^+$: 437.2539, found: 437.2544. **IR (KBr)**: 2978, 1513, 1381, 1248, 1211, 1175, 1100, 1070, 1000 cm^{-1}.

(8R,9S,10R,13S,14S,17S)-17-((4-(4-methoxyphenyl)-2-methylbutan-2-yl)oxy)-10,13-dimethyl-1,2,6,7,8,9,10,11,12,13,14,15,16,17-tetradecahydro-3H-cyclopenta[a]phenanthren-3-one (71)

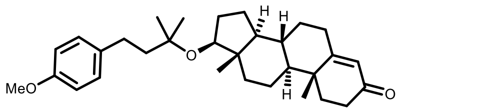

Following the General Procedure C, **71** was obtained in 39% yield as a white solid; **^{1}H NMR (400 MHz, Chloroform-*d*)**: δ 7.14 – 7.05 (m, 2H), 6.86 – 6.75 (m, 2H), 5.71 (s, 1H), 3.77 (s, 3H), 3.39 (t, *J* = 8.1 Hz, 1H), 2.64 – 2.55 (m, 2H), 2.43 – 2.31 (m, 2H), 2.31 – 2.20 (m, 1H), 2.06 – 1.98 (m, 1H), 1.88 – 1.78 (m, 2H), 1.72 – 1.48 (m, 6H), 1.48 – 1.35 (m, 2H), 1.34 – 1.23 (m, 2H), 1.18 (s, 3H), 1.13 (d, *J* = 3.5 Hz, 6H), 1.08 – 0.97 (m, 2H), 0.96 – 0.87 (m, 2H), 0.78 (s, 3H) ppm; **^{13}C NMR (100 MHz, Chloroform-*d*)**: δ 199.8, 171.7, 157.6, 135.4, 129.3, 123.9, 113.8, 80.4, 74.0, 55.4, 54.2, 50.5, 44.5, 42.7, 38.8, 37.1, 35.8, 35.6, 34.1, 33.0, 31.7, 31.3, 29.6, 27.1, 26.2, 23.9, 20.8, 17.5, 11.8 ppm. **HRMS (ESI, m/z)**: calculated for $[M+H]^+$: 465.3369, found: 465.3372. **IR (KBr)**: 2938, 1675, 1613, 1512, 1246, 1071, 1038, 821 cm^{-1}.

(1r,3r,5r,7r)-2-((4-(4-methoxyphenyl)-2-methylbutan-2-yl)oxy)adamantane (72)

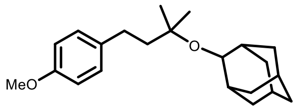

Following the General Procedure C, **72** was obtained in 36% yield as a white solid; **^{1}H NMR (400 MHz, Chloroform-*d*)**: δ 7.13 – 7.07 (m, 2H), 6.86 – 6.75 (m, 2H), 3.78 (s, 3H), 3.57 (br, 1H), 2.70 – 2.56 (m, 2H), 2.15 (d, *J* = 12.4 Hz, 2H), 1.85 – 1.61 (m, 12H), 1.44 (d, *J* = 11.8 Hz, 2H), 1.16 (s, 6H) ppm; **^{13}C NMR (100 MHz, Chloroform-*d*)**: δ 157.6, 135.6, 129.3, 113.8, 74.5, 73.2, 55.4, 44.8, 37.8, 37.2, 35.2, 31.9, 29.8, 27.6, 27.3, 26.2 ppm. **HRMS (ESI, m/z)**: calculated for $[M+H]^+$: 329.2481, found: 329.2485. **IR (KBr)**: 2904, 1512, 1246, 1101, 1064, 1033 cm^{-1}.

(3S,8S,9S,10R,13R,14S,17R)-17-((2R,5S)-5-ethyl-6-methylheptan-2-yl)-3-((4-(4-methoxyphenyl)-2-methylbutan-2-yl)oxy)-10,13-dimethyl-2,3,4,7,8,9,10,11,12,13,14,15,16,17-tetradecahydro-1H-cyclopenta[a]phenanthrene (73)

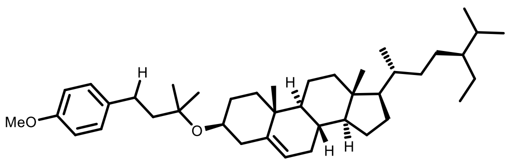

Following the General Procedure C, **73** was obtained in 36% yield as a white solid; **^{1}H NMR (400 MHz, Chloroform-*d*)**: δ 7.14 – 7.08 (m, 2H), 6.86 – 6.80 (m, 2H), 5.34 – 5.29 (m, 1H), 3.79 (s, 3H), 3.43 – 3.28 (m, 1H), 2.69 – 2.57 (m, 2H), 2.37 – 2.26 (m, 1H), 2.22 – 2.14 (m, 1H), 2.06 – 1.92 (m, 2H), 1.88 – 1.78 (m, 2H), 1.77 – 1.71 (m, 2H), 1.70 – 1.61 (m, 2H), 1.61 – 1.42 (m, 7H), 1.39 – 1.24 (m, 5H), 1.22 (s, 6H), 1.16 – 1.03 (m, 6H), 1.01 (s, 3H), 0.97 – 0.89 (m, 6H), 0.88 – 0.86 (m, 1H), 0.85 (s, 3H), 0.84 – 0.82 (m, 2H), 0.82 – 0.80 (m, 2H), 0.80 – 0.77 (m, 2H), 0.68 (s, 3H) ppm; **^{13}C NMR (100 MHz, Chloroform-*d*)**: δ 157.7, 142.0, 135.1, 129.3, 121.1, 113.8, 75.3, 71.3, 56.9, 56.1, 55.3, 50.4, 45.9, 43.4, 42.4, 42.3, 39.9, 37.9, 36.7, 36.2, 34.0, 32.1, 32.0, 31.5, 29.8, 29.2, 28.4, 26.6, 26.6, 26.1, 24.4, 23.1, 21.1, 20.0, 19.5, 19.1, 18.9, 12.1, 12.0 ppm. **HRMS (ESI, m/z)**: calculated for $[M+H]^+$: 591.5141, found: 591.5146. **IR (KBr)**: 2936, 1743, 1514, 1460, 1383, 1367, 1250, 1074, 1036 cm^{-1}.

1-methoxy-4-(3-(methoxy-d3)-3-methylbutyl-1-d)benzene (113)

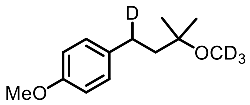

Following the General Procedure B, **113** was obtained in 84% yield as a colorless oil; **^{1}H NMR (400 MHz, Chloroform-*d*):** δ 7.16 – 7.08 (m, 2H), 6.87 – 6.80 (m, 2H), 3.79 (s, 3H), 2.63 – 2.52 (m, 1H), 1.74 (d, J = 8.8 Hz, 2H), 1.22 (s, 6H) ppm; **^{13}C NMR (100 MHz, Chloroform-*d*):** δ 157.7, 135.0, 129.3, 113.9, 74.4, 55.4, 42.0, 29.4, 29.0, 25.2 ppm. **HRMS (ESI, m/z):** calculated for $[M+H]^+$: 213.3334, found: 213.3339. **IR (KBr)**: 2971, 2062, 1513, 1245, 1121, 1039, 825 cm^{-1}.

1-(4-(4-methoxyphenyl)-2-methylbutan-2-yl)-1H-pyrazole (27)

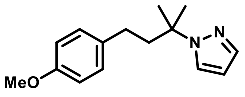

Following the General Procedure A, **27** was obtained in 85% yield as a colorless oil; **^{1}H NMR (400 MHz, Chloroform-*d*)**: δ 7.58 (d, J = 1.7 Hz, 1H), 7.54 (dd, J = 2.3, 0.5 Hz, 1H), 7.06 – 7.00 (m, 2H), 6.82 – 6.76 (m, 2H), 6.27 – 6.24 (m, 1H), 3.76 (s, 3H), 2.28 – 2.20 (m, 2H), 2.20 – 2.08 (m, 2H), 1.64 (s, 6H) ppm; **^{13}C NMR (100 MHz, Chloroform-*d*)**: δ 157.8, 138.9, 134.0, 129.3, 126.4, 113.9, 104.7, 60.7, 55.3, 45.2, 29.5, 27.8 ppm; **HRMS (ESI, m/z)**: calculated for $[M+H]^+$: 245.1654, found: 245.1649. **IR (KBr)**: 2977, 2933, 1612, 1512, 1245, 1178, 1037, 824, 747 cm^{-1}.

4-chloro-1-(4-(4-methoxyphenyl)-2-methylbutan-2-yl)-1H-pyrazole (88)

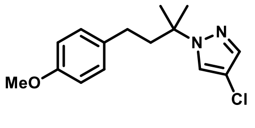

Following the General Procedure A, **88** was obtained in 71% yield as a colorless oil; **^{1}H NMR (400 MHz, Chloroform-*d*):** δ 7.51 (d, J = 0.5 Hz, 1H), 7.48 (s, 1H), 7.05 – 7.00 (m, 2H), 6.82 – 6.76 (m, 2H), 3.77 (s, 3H), 2.36 – 2.22 (m, 2H), 2.15 – 2.08 (m, 2H), 1.60 (s, 6H) ppm; **^{13}C NMR (100 MHz, Chloroform-*d*):** δ 157.9, 137.2, 133.6, 129.2, 124.8, 113.9, 109.1, 61.7, 55.3, 44.8, 29.5, 27.5 ppm; **HRMS (ESI, m/z):** calculated for $[M+H]^+$: 279.1264, found: 279.1261. **IR (KBr)**: 2979, 1612, 1513, 1327, 1246, 1178, 1037, 968, 825 cm^{-1}.

4-bromo-1-(4-(4-methoxyphenyl)-2-methylbutan-2-yl)-1H-pyrazole (89)

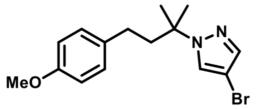

Following the General Procedure A, **89** was obtained in 66% yield as a colorless oil; **^{1}H NMR (400 MHz, Chloroform-*d*)**: δ 7.54 – 7.52 (m, 2H), 7.05 – 6.99 (m, 2H), 6.82 – 6.76 (m, 2H), 3.77 (s, 3H), 2.30 – 2.24 (m, 2H), 2.15 – 2.08 (m, 2H), 1.61 (s, 6H) ppm; **^{13}C NMR (100 MHz, Chloroform-*d*)**: δ 157.9, 139.4, 133.6, 129.2, 126.9, 113.9, 92.3, 61.7, 55.4, 44.8, 29.5, 27.6 ppm; **HRMS (ESI, m/z)**: calculated for $[M+H]^+$: 323.0759, found: 323.0757. **IR (KBr)**: 2978, 1612, 1512, 1246, 1178, 1037, 953 cm^{-1}.

1-(4-(4-methoxyphenyl)-2-methylbutan-2-yl)-4-methyl-1H-pyrazole (90)

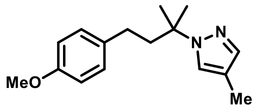

Following the General Procedure A, **90** was obtained in 53% yield as a colorless oil; **^{1}H NMR (400 MHz, Chloroform-*d*)**: δ 7.37 (s, 1H), 7.31 (s, 1H), 7.07 – 7.00 (m, 2H), 6.82 – 6.76 (m, 2H), 3.77 (s, 3H), 2.30 – 2.24 (m, 2H), 2.13 – 2.10 (m, 2H), 2.09 (s, 3H), 1.60 (s, 6H) ppm; **^{13}C NMR (100 MHz, Chloroform-*d*)**: δ 157.8, 139.2, 134.1, 129.3, 125.3, 115.1, 113.8, 60.3, 55.3, 45.2, 29.6, 27.8, 9.1 ppm; **HRMS (ESI, m/z)**: calculated for $[M+H]^+$: 259.1810, found: 259.1807. **IR (KBr)**: 2976, 1611, 1512, 1246, 1177, 1037, 978, 825 cm^{-1}.

3-(tert-butyldimethylsilyl)-1-(4-(4-methoxyphenyl)-2-methylbutan-2-yl)-1H-pyrazole (91)

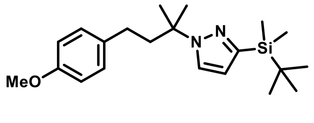

Following the General Procedure A, **91** was obtained in 74% yield as a colorless oil; **^{1}H NMR (400 MHz, Chloroform-*d*)**: δ 7.57 (d, *J* = 2.2 Hz, 1H), 7.08 – 7.02 (m, 2H), 6.86 – 6.80 (m, 2H), 6.39 (d, *J* = 2.2 Hz, 1H), 3.80 (s, 3H), 2.28 – 2.23 (m, 2H), 2.22 – 2.16 (m, 2H), 1.66 (s, 6H), 0.33 (s, 9H) ppm; **^{13}C NMR (100 MHz, Chloroform-*d*)**: δ 157.8, 151.9, 134.5, 129.3, 126.1, 113.9, 110.7, 60.6, 55.4, 45.4, 29.6, 28.0, -0.7 ppm; **HRMS (ESI, m/z)**: calculated for $[M+H]^+$: 359.2519, found: 359.2522. **IR (KBr)**: 2955, 1512, 1246, 841, 753 cm^{-1}.

1-(4-(4-methoxyphenyl)-2-methylbutan-2-yl)-1H-indazole (92)

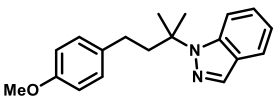

Following the General Procedure A, **92** was obtained in 52% yield as a colorless oil; **^{1}H NMR (400 MHz, Chloroform-*d*)**: δ 8.06 (d, *J* = 0.9 Hz, 1H), 7.78 – 7.74 (m, 1H), 7.69 – 7.65 (m, 1H), 7.34 – 7.23 (m, 1H), 7.10 – 7.04 (m, 1H), 7.03 – 6.97 (m, 2H), 6.79 – 6.74 (m, 2H), 3.75 (s, 3H), 2.36 – 2.29 (m, 2H), 2.28 – 2.22 (m, 2H), 1.79 (s, 6H) ppm; **^{13}C NMR (100 MHz, Chloroform-*d*)**: δ 157.9, 148.5, 133.6, 129.3, 125.7, 121.4, 121.3, 120.3, 120.2, 117.7, 113.8, 62.5, 55.3, 45.2, 29.5, 28.2 ppm; **HRMS (ESI, m/z)**: calculated for $[M+H]^+$: 295.1810, found: 295.1812. **IR (KBr)**: 2929, 1512, 1246, 1177, 1036, 756 cm^{-1}.

2-(4-(4-methoxyphenyl)-2-methylbutan-2-yl)-2H-1,2,3-triazole (93)

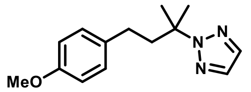

Following the General Procedure A, **93** was obtained in 22% yield as a colorless oil; **^{1}H NMR (400 MHz, Chloroform-*d*)**: δ 7.62 (s, 2H), 7.06 – 7.00 (m, 2H), 6.82 – 6.76 (m, 2H), 3.77 (s, 3H), 2.31 – 2.26 (m, 2H), 2.26 – 2.20 (m, 2H), 1.73 (s, 6H) ppm; **^{13}C NMR (100 MHz, Chloroform-*d*)**: δ 157.9, 133.8, 133.4, 129.2, 113.9, 65.2, 55.3, 45.0, 29.6, 27.5 ppm; **HRMS (ESI, m/z)**: calculated for $[M+H]^+$: 246.1606, found: 246.1603. **IR (KBr)**: 2984, 1740, 1513, 1246, 1177, 1038, 963, 820 cm^{-1}.

1-(4-(4-methoxyphenyl)-2-methylbutan-2-yl)-1H-1,2,3-triazole (94)

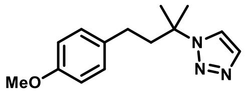

Following the General Procedure A, **94** was obtained in 37% yield as a colorless oil; **^{1}H NMR (400 MHz, Chloroform-*d*)**: δ 7.72 (s, 1H), 7.62 (s, 1H), 7.04 – 6.98 (m, 2H), 6.82 – 6.76 (m, 2H), 3.77 (s, 3H), 2.38 – 2.25 (m, 2H), 2.26 – 2.18 (m, 2H), 1.73 (s, 6H) ppm; **^{13}C NMR (100 MHz, Chloroform-*d*)**: δ 158.0, 133.4, 133.1, 129.2, 121.1, 114.0, 61.7, 55.4, 45.2, 29.4, 28.0 ppm; **HRMS (ESI, m/z)**: calculated for $[M+H]^+$: 246.1606, found: 246.1599. **IR (KBr)**: 2984, 1513, 1245, 1041, 825, 809 cm^{-1}.

2-(4-(4-methoxyphenyl)-2-methylbutan-2-yl)-2H-benzo[d][1,2,3]triazole (95)

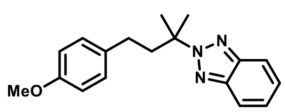

Following the General Procedure A, **95** was obtained in 31% yield as a colorless oil; **^{1}H NMR (400 MHz, Chloroform-*d*)**: δ 7.94 – 7.86 (m, 2H), 7.40 – 7.34 (m, 2H), 7.04 – 6.98 (m, 2H), 6.80 – 6.74 (m, 2H), 3.75 (s, 3H), 2.45 – 2.38 (m, 2H), 2.37 – 2.30 (m, 2H), 1.90 (s, 6H) ppm; **^{13}C NMR (100 MHz, Chloroform-*d*)**: δ 157.9, 143.9, 133.5, 129.2, 126.0, 118.2, 113.8, 67.2, 55.3, 45.2, 29.6, 28.0 ppm; **HRMS (ESI, m/z)**: calculated for $[M+H]^+$: 296.1763, found: 296.1764. **IR (KBr)**: 2988, 1740, 1513, 1246, 1040, 829, 756 cm^{-1}.

1-(4-(4-methoxyphenyl)-2-methylbutan-2-yl)-1H-benzo[d][1,2,3]triazole (96)

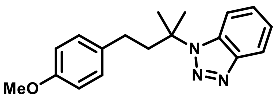

Following the General Procedure A, **96** was obtained in 35% yield as a colorless oil; **^{1}H NMR (400 MHz, Chloroform-*d*)**: δ 8.11 – 8.06 (m, 1H), 7.76 – 7.71 (m, 1H), 7.47 – 7.41 (m, 1H), 7.39 – 7.33 (m, 1H), 6.94 – 6.89 (m, 2H), 6.77 – 6.70 (m, 2H), 3.74 (s, 3H), 2.45 – 2.38 (m, 2H), 2.33 – 2.25 (m, 2H), 1.94 (s, 6H) ppm; **^{13}C NMR (100 MHz, Chloroform-*d*)**: δ 157.9, 147.2, 133.2, 132.1, 129.2, 126.7, 123.5, 120.5, 113.9, 111.9, 63.3, 55.3, 43.9, 29.5, 27.9 ppm; **HRMS (ESI, m/z)**: calculated for $[M+H]^+$: 296.1763, found: 296.1758. **IR (KBr)**: 2985, 1740, 1513, 1373, 1247, 1039, 747 cm^{-1}.

1-(3-azido-3-methylbutyl)-4-methoxybenzene (97)

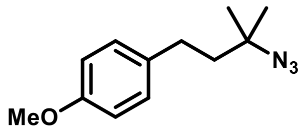

Following the General Procedure I, **97** was obtained in 87% yield as a colorless oil; **^{1}H NMR (400 MHz, Chloroform-*d*)**: δ 7.15 – 7.08 (m, 2H), 6.87 – 6.81 (m, 2H), 3.79 (s, 3H), 2.68 – 2.57 (m, 2H), 1.81 – 1.71 (m, 2H), 1.33 (s, 6H) ppm; **^{13}C NMR (100 MHz, Chloroform-*d*)**: δ 157.8, 133.8, 129.1, 113.8, 61.4, 55.2, 43.8, 29.8,

26.0 ppm; **HRMS (ESI, m/z)**: calculated for [M+H]$^+$: 220.1444, found: 220.1440. **IR (KBr)**: 2096, 1514, 1464, 1421, 913, 764, 749 cm^{-1}.

1-methoxy-4-(3-methyl-3-thiocyanatobutyl)benzene (98)

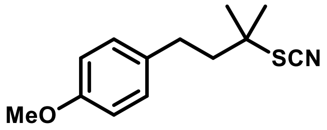

Following the General Procedure J, **98** was obtained in 88% yield as a colorless oil; **^{1}H NMR (400 MHz, Chloroform-*d*)**: δ 7.17 – 7.07 (m, 2H), 6.88 – 6.80 (m, 2H), 3.79 (s, 3H), 2.75 – 2.63 (m, 2H), 2.06 – 1.94 (m, 2H), 1.57 (s, 6H)ppm; **^{13}C NMR (100 MHz, Chloroform-*d*)**: δ 158.0, 132.6, 129.2, 113.9, 111.8, 55.6, 55.3, 45.0, 30.5, 28.9 ppm; **HRMS (ESI, m/z)**: calculated for [M+H]$^+$: 236.1104, found: 236.1105. **IR (KBr)**: 2931, 2083, 1613, 1512, 1247, 1177, 1036 cm^{-1}.

4-(4-methoxyphenyl)-2-methylbutan-2-yl acetate (74)

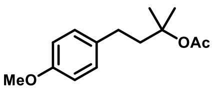

Following the General Procedure E, **74** was obtained in 77% yield as a colorless oil; **^{1}H NMR (400 MHz, Chloroform-*d*)**: δ 7.13 – 7.07 (m, 2H), 6.85 – 6.79 (m, 2H), 3.77 (s, 3H), 2.67 – 2.48 (m, 2H), 2.06 – 2.00 (m, 2H), 1.97 (s, 3H), 1.49 (s, 6H) ppm; **^{13}C NMR (100 MHz, Chloroform-*d*)**: δ 170.6, 157.9, 134.2, 129.3, 113.9, 82.1, 55.3, 43.0, 29.5, 26.2, 22.5 ppm; **HRMS (ESI, m/z)**: calculated for [M+H]+: 237.1491, found: 237.1487. **IR (KBr)**: 2977, 2936, 1732, 1613, 1513, 1367, 1247, 1203, 1177, 1125, 1037, 829 cm^{-1}.

1-(4-methoxyphenyl)-3-(4-(trifluoromethyl)phenyl)propyl acetate (75)

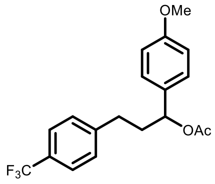

Following the General Procedure E, **75** was obtained in 53% yield as a colorless oil; **^{1}H NMR (400 MHz, Chloroform-*d*)**: δ 7.53 (d, *J* = 8.1 Hz, 2H), 7.31 – 7.24 (m, 4H), 6.92 – 6.86 (m, 2H), 5.74 – 5.68 (m, 1H), 3.81 (s, 3H), 2.75 – 2.56 (m, 2H), 2.35 – 2.22 (m, 1H), 2.15 – 2.06 (m, 1H), 2.05 (s, 3H) ppm; **^{13}C NMR (100 MHz, Chloroform-*d*)**: δ 170.4, 159.5, 145.5, 132.2, 128.7, 128.1, 125.43 (q, *J* = 3.8 Hz), 124.4 (q, *J* = 271.8 Hz), 114.2, 75.2, 55.4, 37.3, 31.9, 21.3 ppm. **HRMS (ESI, m/z)**: calculated for [M+H]$^+$: 353.1365, found: 353.1366. **IR (KBr)**: 2960, 1740, 1610, 1510, 1370, 1330, 1250, 1120, 1070, 832 cm^{-1}.

4-(4-methoxyphenyl)-2-methylbutan-2-yl propionate (76)

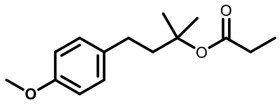

Following the General Procedure E, **76** was obtained in 73% yield as a colorless oil; **^{1}H NMR (400 MHz, Chloroform-*d*):** δ 7.13 – 7.08 (m, 2H), 6.85 – 6.79 (m, 2H), 3.78 (s, 3H), 2.63 – 2.53 (m, 2H), 2.25 (q, *J* = 7.6 Hz, 2H), 2.07 – 2.00 (m, 2H), 1.49 (s, 6H), 1.11 (t, *J* = 7.6 Hz, 3H) ppm; **^{13}C NMR (100 MHz, Chloroform-*d*):** δ 174.0, 157.9, 134.3, 129.3, 113.9, 81.8, 55.4, 43.1, 29.5, 28.9, 26.2, 9.4 ppm. **HRMS (ESI, m/z):** calculated for [M+Na]$^+$: 273.1467, found: 273.1463. **IR (KBr)**: 2973, 1738, 1486, 1113, 1073, 763, 697 cm^{-1}.

4-(4-methoxyphenyl)-2-methylbutan-2-yl 4-cyanobenzoate (77)

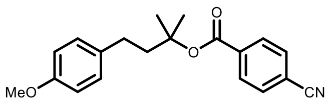

Following the General Procedure E, **77** was obtained in 86% yield as a colorless oil; **^{1}H NMR (400 MHz, Chloroform-*d*)**: δ 8.04 (d, *J* = 8.6 Hz, 2H), 7.71 (d, *J* = 8.5 Hz, 2H), 7.15 – 7.08 (m, 2H), 6.84 – 6.78 (m, 2H), 3.76 (s, 3H), 2.71 – 2.63 (m, 2H), 2.24 – 2.16 (m, 2H), 1.65 (s, 6H) ppm; **^{13}C NMR (100 MHz, Chloroform-*d*)**: δ 164.0, 157.9, 135.7, 133.8, 132.2, 130.0, 129.3, 118.3, 116.0, 114.0, 84.3, 55.3, 42.9, 29.6, 26.2 ppm. **HRMS (ESI, m/z)**: calculated for $[M+H]^+$: 324.1600, found: 324.1597. **IR (KBr)**: 2935, 2231, 1717, 1513, 1297, 1247, 1176, 1117, 768 cm^{-1}.

4-(4-methoxyphenyl)-2-methylbutan-2-yl cinnamate (78)

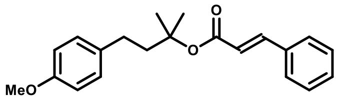

Following the General Procedure E, **78** was obtained in 81% yield as a colorless oil; **^{1}H NMR (400 MHz, Chloroform-*d*)**: δ 7.60 (d, *J* = 16.0 Hz, 1H), 7.56 – 7.48 (m, 2H), 7.41 – 7.36 (m, 3H), 7.17 – 7.12 (m, 2H), 6.87 – 6.81 (m, 2H), 6.38 (d, *J* = 16.0 Hz, 1H), 3.78 (s, 3H), 2.90 – 2.57 (m, 2H), 2.22 – 2.03 (m, 2H), 1.59 (s, 6H) ppm; **^{13}C NMR (101 MHz, Chloroform-*d*)**: δ 166.3, 157.8, 143.8, 134.7, 134.3, 130.1, 129.4, 128.9, 128.1, 120.1, 113.9, 82.4, 55.3, 43.1, 29.6, 26.4 ppm. **HRMS (ESI, m/z)**: calculated for $[M+H]^+$: 325.1804, found: 325.1806. **IR (KBr)**: 2980, 2942, 1739, 1708, 1637, 1505, 1253 cm^{-1}.

4-(4-methoxy-3-methylphenyl)-2-methylbutan-2-yl cinnamate (79)

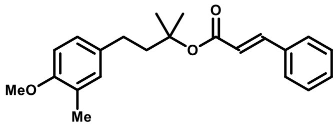

Following the General Procedure E, **79** was obtained in 67% yield as a colorless oil; **^{1}H NMR (400 MHz, Chloroform-*d*)**: δ 7.60 (d, *J* = 16.0 Hz, 1H), 7.55 – 7.49 (m, 2H), 7.42 – 7.34 (m, 3H), 7.03 – 6.97 (m, 2H), 6.76 – 6.72 (m, 1H), 6.38 (d, *J* = 16.0 Hz, 1H), 3.80 (s, 3H), 2.67 – 2.56 (m, 2H), 2.21 (s, 3H), 2.16 – 2.06 (m, 2H), 1.59 (s, 6H) ppm; **^{13}C NMR (101 MHz, Chloroform-*d*)**: δ 166.3, 156.0, 143.7, 134.7, 133.9, 130.9, 130.1, 128.9, 128.1, 126.6, 126.4, 120.1, 110.0, 82.4, 55.5, 43.3, 29.6, 26.3, 16.3 ppm. **HRMS (ESI, m/z)**: calculated for $[M+H]^+$: 339.1960, found: 339.1962. **IR (KBr)**: 2975, 1738, 1706, 1635, 1512, 1248 cm^{-1}.

4-(4-methoxyphenyl)-2-methylbutan-2-yl 2-((phenylsulfonyl)methyl)acrylate (80)

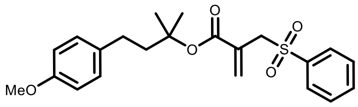

Following the General Procedure E, **80** was obtained in 74% yield as a white solid; **^{1}H NMR (400 MHz, Chloroform-*d*)**: δ 7.89 – 7.79 (m, 2H), 7.60 – 7.54 (m, 1H), 7.53 – 7.47 (m, 2H), 7.10 – 7.04 (m, 2H), 6.86 – 6.79 (m, 2H), 6.43 (d, *J* = 0.8 Hz, 1H), 5.89 (d, *J* = 0.7 Hz, 1H), 4.13 (s, 2H), 3.78 (s, 3H), 2.61 – 2.38 (m, 2H), 2.07 – 1.86 (m, 2H), 1.38 (s, 6H) ppm; **^{13}C NMR (101 MHz, Chloroform-*d*)**: δ 163.8, 157.9, 138.5, 133.9, 132.8, 130.3, 129.3, 129.1, 128.9, 113.9, 83.77, 57.38, 55.38, 43.00, 29.48, 25.78 ppm. **HRMS (ESI, m/z)**: calculated for $[M+H]^+$: 403.1579, found: 403.1583. **IR (KBr)**: 2934, 1716, 1513, 1320, 1247, 1148 cm^{-1}.

4-(4-(tert-butyl)phenyl)-2-methylbutan-2-yl 2-(11-oxo-6,11-dihydrodibenzo[b,e]oxepin-2-yl)acetate (81)

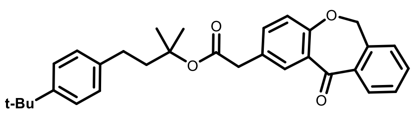

Following the General Procedure E, **81** was obtained in 58% yield as a white solid; **^{1}H NMR (400 MHz, Chloroform-*d*)**: ^{1}H NMR (400 MHz, Chloroform-d) δ 8.18 – 8.13 (m, 1H), 7.90 (d, *J* = 7.7 Hz, 1H), 7.59 – 7.53 (m, 1H), 7.49 – 7.42 (m, 2H), 7.36 (d, *J* = 7.4 Hz, 1H), 7.32 – 7.25 (m, 2H), 7.11 – 7.06 (m, 2H), 7.06 – 7.01 (m, 1H), 5.17 (s, 2H), 3.56 (s, 2H), 2.60 – 2.44 (m, 2H), 2.09 – 2.00 (m, 2H), 1.52 (s, 6H), 1.31 (s, 9H) ppm; **^{13}C NMR (100 MHz, Chloroform-*d*)**: δ 191.0, 170.7, 160.5, 148.7, 140.6, 139.0, 136.5, 135.7, 132.8, 132.6, 129.6, 129.3, 128.6, 128.1, 127.9, 125.4, 125.2, 121.1, 82.9, 73.7, 42.8, 41.6, 34.4, 31.5, 29.8, 26.2 ppm. **HRMS (ESI, m/z)**: calculated for [M+H]$^{+}$: 471.2535, found: 471.2531.

4-(4-methoxyphenyl)-2-methylbutan-2-yl-4-hydroxy-1-methyl-7-phenoxyisoquinoline-3-carboxylate (82)

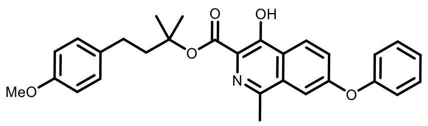

Following the General Procedure E, **82** was obtained in 37% yield as a white solid; **^{1}H NMR (400 MHz, Chloroform-*d*)**: δ 11.95 (s, 1H), 8.38 (d, *J* = 8.8 Hz, 1H), 7.48 – 7.44 (m, 2H), 7.44 – 7.40 (m, 2H), 7.25 – 7.20 (m, 1H), 7.19 – 7.14 (m, 2H), 7.14 – 7.09 (m, 2H), 6.85 – 6.80 (m, 2H), 3.76 (s, 3H), 2.80 – 2.74 (m, 2H), 2.73 (s, 3H), 2.33 – 2.22 (m, 2H), 1.75 (s, 6H) ppm; **^{13}C NMR (100 MHz, Chloroform-*d*)**: δ 170.6, 158.9, 157.8, 156.0, 155.7, 148.1, 134.2, 132.3, 130.3, 129.4, 126.0, 124.6, 123.9, 122.1, 120.0, 119.9, 113.9, 111.6, 85.3, 55.3, 43.5, 29.7, 26.3, 22.6 ppm. **HRMS (ESI, m/z)**: calculated for [M+H]$^{+}$: 472.2124, found: 472.2129. **IR (KBr)**: 1651, 1512, 1489, 1420, 1328, 1226, 1161 cm^{-1}.

4-(4-methoxyphenyl)-2-methylbutan-2-yl 2-(3-benzoylphenyl)propanoate (83)

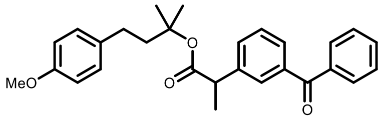

Following the General Procedure E, **83** was obtained in 78% yield as a white solid; **^{1}H NMR (400 MHz, Chloroform-*d*)**: δ 7.79 – 7.77 (m, 2H), 7.77 – 7.75 (m, 1H), 7.69 – 7.65 (m, 1H), 7.60 – 7.54 (m, 2H), 7.47 – 7.41 (m, 3H), 7.01 – 6.96 (m, 2H), 6.81 – 6.76 (m, 2H), 3.76 (s, 3H), 3.76 – 3.69 (m, 1H), 2.48 – 2.26 (m, 2H), 2.03 – 1.88 (m, 2H), 1.52 (d, *J* = 7.2 Hz, 3H), 1.45 (s, 6H) ppm; **^{13}C NMR (100 MHz, Chloroform-*d*)**: δ 196.7, 173.3, 157.8, 141.6, 137.9, 137.6, 134.1, 132.6, 131.6, 130.1, 129.3, 129.3, 129.0, 128.6, 128.4, 113.9, 82.7, 55.4, 46.5, 43.1, 29.3, 26.2, 26.0, 18.4 ppm. **HRMS (ESI, m/z)**: calculated for [M+H]$^{+}$: 431.2222, found: 431.2219. **IR (KBr)**: 1727, 1661, 1513, 1514, 1247, 1179 cm^{-1}.

4-(4-methoxyphenyl)-2-methylbutan-2-yl 2-(2-fluoro-[1,1'-biphenyl]-4-yl)propanoate (84)

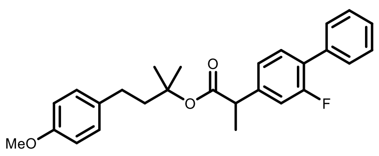

Following the General Procedure E, **84** was obtained in 75% yield as a white solid; **^{1}H NMR (400 MHz, Chloroform-*d*)**: δ 7.58 – 7.53 (m, 2H), 7.48 – 7.45 (m, 1H), 7.45 – 7.43 (m, 1H), 7.43 – 7.35 (m, 2H), 7.22 – 7.14 (m, 2H), 7.07 – 7.02 (m, 2H), 6.85 – 6.80 (m, 2H), 3.79 (s, 3H), 3.77 – 3.65 (m, 1H), 2.54 – 2.39 (m, 2H), 2.09 – 1.92 (m, 2H), 1.54 (d, *J* = 7.2 Hz, 3H), 1.50 (s, 6H)

ppm; **^{13}C NMR (100 MHz, Chloroform-*d*)**: δ 173.3, 161.0, 158.5, 157.9, 142.6 (d, *J* = 7.6 Hz), 135.7, 134.1, 130.8 (d, *J* = 3.8 Hz), 129.3, 129.1 (d, *J* = 2.6 Hz), 128.5, 127.7, 123.7 (d, *J* = 3.0 Hz), 115.4 (d, *J* = 23.7 Hz), 113.9, 82.8, 55.4, 46.2, 43.1, 29.3, 26.2, 26.0, 18.4 ppm. **HRMS (ESI, m/z)**: calculated for $[M+H]^+$: 421.2179, found: 421.2185. **IR (KBr)**: 1727, 1513, 1246, 1179, 1122, 766, 698 cm^{-1}.

4-(4-methoxyphenyl)-2-methylbutan-2-yl (4R)-4-((8R,9S,10S,13R,14S,17R)-10,13-dimethyl-3,7,12-trioxohexadecahydro-1H-cyclopenta[a]phenanthren-17-yl)pentanoate (85)

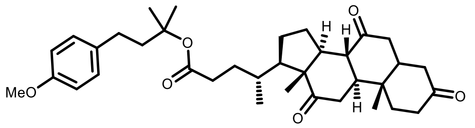

Following the General Procedure E, **85** was obtained in 36% yield as a white solid; **^{1}H NMR (400 MHz, Chloroform-*d*)**: δ 7.12 – 7.06 (m, 2H), 6.85 – 6.78 (m, 2H), 3.78 (s, 3H), 2.99 – 2.79 (m, 3H), 2.62 – 2.49 (m, 2H), 2.37 – 2.26 (m, 4H), 2.26 – 2.17 (m, 4H), 2.16 – 2.09 (m, 2H), 2.07 – 1.98 (m, 4H), 1.98 – 1.90 (m, 1H), 1.89 – 1.76 (m, 2H), 1.72 – 1.54 (m, 2H), 1.48 (d, *J* = 1.9 Hz, 6H), 1.39 (s, 3H), 1.36 – 1.20 (m, 4H), 1.05 (s, 3H), 0.84 (d, *J* = 6.4 Hz, 3H) ppm; **^{13}C NMR (100 MHz, Chloroform-*d*)**: δ 212.11, 209.26, 208.89, 173.53, 157.79, 134.24, 129.31, 113.87, 81.86, 56.97, 55.37, 51.83, 49.08, 46.94, 45.75, 45.61, 45.08, 43.14, 42.89, 38.73, 36.60, 36.10, 35.56, 35.35, 32.79, 30.66, 29.53, 27.76, 26.26, 26.21, 25.24, 22.00, 18.75, 11.93 pm. **HRMS (ESI, m/z)**: calculated for $[M+H]^+$: 579.3686, found: 579.3691. **IR (KBr)**: 2971, 2868, 1733, 1700, 1512, 1247, 1163 cm^{-1}.

4-(4-methoxyphenyl)-2-methylbutan-2-yl (((9H-fluoren-9-yl)methoxy)carbonyl)-D-alaninate (86)

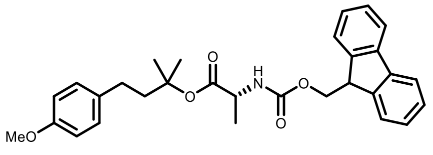

Following the General Procedure E, **86** was obtained in 65% yield as a white solid; **^{1}H NMR (400 MHz, Chloroform-*d*)**: δ 7.78 (d, *J* = 7.5 Hz, 2H), 7.66 – 7.59 (m, 2H), 7.41 (t, *J* = 7.5 Hz, 2H), 7.36 – 7.30 (m, 2H), 7.14 – 7.07 (m, 2H), 6.87 – 6.79 (m, 2H), 5.42 (d, *J* = 7.6 Hz, 1H), 4.47 – 4.38 (m, 2H), 4.37 – 4.28 (m, 1H), 4.28 – 4.20 (m, 1H), 3.78 (s, 3H), 2.69 – 2.51 (m, 2H), 2.13 – 2.03 (m, 2H), 1.54 (s, 6H), 1.44 (d, *J* = 7.1 Hz, 3H) ppm; **^{13}C NMR (100 MHz, Chloroform-*d*)**: δ 172.4, 157.9, 155.7, 144.1, 143.9, 141.4, 133.9, 129.3, 127.8, 127.2, 125.3, 120.1, 114.0, 83.9, 67.0, 55.4, 50.3, 47.3, 43.1, 29.5, 26.2, 26.0, 19.1 ppm. **HRMS (ESI, m/z)**: calculated for $[M+H]^+$: 488.2437, found: 488.2439. **IR (KBr)**: 1724, 1513, 1451, 1246, 1076, 1037, 741 cm^{-1}.

5-(4-methoxyphenethyl)dihydrofuran-2(3H)-one (87)

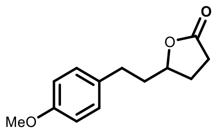

Following the General Procedure D, **87** was obtained in 91% yield as a colorless oil; **^{1}H NMR (400 MHz, Chloroform-*d*)**: δ 7.14 – 7.09 (m, 2H), 6.86 – 6.81 (m, 2H), 4.51 – 4.41 (m, 1H), 3.79 (s, 3H), 2.82 – 2.62 (m, 2H), 2.57 – 2.50 (m, 2H), 2.35 – 2.24 (m, 1H), 2.08 – 1.95 (m, 1H), 1.92 – 1.80 (m, 2H) ppm; **^{13}C NMR (100 MHz, Chloroform-*d*)**: δ

177.4, 158.1, 132.8, 129.5, 114.0, 80.1, 55.4, 37.7, 30.8, 29.0, 28.1 ppm. **HRMS (ESI, m/z)**: calculated for $[M+H]^+$: 221.1178, found: 221.1182. **IR (KBr)**: 1771, 1611, 1513, 1244, 1176, 1034 cm^{-1}.

4-(3-chloro-3-methylbutyl)-1,1'-biphenyl (99)

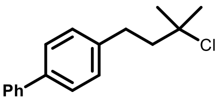

Following the General Procedure F, **99** was obtained in 89% yield as a colorless oil; **^{1}H NMR (400 MHz, Chloroform-*d*)**: δ 7.67 – 7.52 (m, 4H), 7.49 – 7.41 (m, 2H), 7.38 – 7.33 (m, 1H), 7.33 – 7.27 (m, 2H), 3.01 – 2.66 (m, 2H), 2.24 – 1.99 (m, 2H), 1.68 (s, 6H) ppm; **^{13}C NMR (100 MHz, Chloroform-*d*)**: δ 141.1, 141.0, 139.0, 128.9, 128.8, 127.3, 127.2, 127.1, 70.7, 48.0, 32.6, 31.4 ppm. **HRMS (ESI, m/z)**: calculated for $[M+H]^+$: 259.1254, found: 259.1257. **IR (KBr)**: 2971, 1487, 1370, 825, 761, 697 cm^{-1}.

1-(3-chloropropyl)-4-methoxybenzene (100)

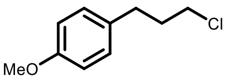

Following the General Procedure F, **100** was obtained in 95% yield as a colorless oil; **^{1}H NMR (400 MHz, Chloroform-*d*)**: δ 7.15 – 7.09 (m, 2H), 6.89 – 6.82 (m, 2H), 3.80 (s, 3H), 3.53 (t, *J* = 6.5 Hz, 2H), 2.73 (t, *J* = 7.4 Hz, 2H), 2.13 – 1.94 (m, 2H) ppm; **^{13}C NMR (100 MHz, Chloroform-*d*)**: δ 158.0, 132.8, 129.6, 114.0, 55.4, 44.4, 34.3, 31.9 ppm. **HRMS (ESI, m/z)**: calculated for $[M+H]^+$: 185.0733, found: 185.0735. **IR (KBr)**: 2935, 1513, 1245, 1037 cm^{-1}.

1-(3-chloro-5-phenylpentyl)-4-methoxybenzene (104) & 1-(2-(chloromethyl)-4-phenylbutyl)-4-methoxybenzene (105)

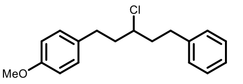

104

+

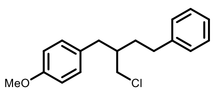

105

Following the General Procedure F, a mixture of **104** and **105** (3.3:1) was obtained in 96% yield as a colorless oil; **104**: **^{1}H NMR (400 MHz, Chloroform-*d*)**: δ 7.36 – 7.29 (m, 2H), 7.27 – 7.19 (m, 3H), 7.16 – 7.10 (m, 2H), 6.90 – 6.84 (m, 2H), 3.94 – 3.84 (m, 1H), 3.82 (s, 3H), 2.98 – 2.87 (m, 2H), 2.80 – 2.65 (m, 2H), 2.10 – 1.99 (m, 4H) ppm; **^{13}C NMR (100 MHz, Chloroform-*d*)**: δ 158.1, 141.3 , 133.2, 129.6, 128.7 , 128.6, 114.0, 62.5, 55.4, 40.6, 40.4, 32.9, 31.9 ppm; **105**: **^{1}H NMR (400 MHz, Chloroform-*d*)**: δ 7.36 – 7.29 (m, 2H), 7.27 – 7.19 (m, 3H), 7.16 – 7.10 (m, 2H), 6.90 – 6.84 (m, 2H), 3.94 – 3.84 (m, 1H), 3.82 (s, 3H), 3.58 (dd, *J* = 11.0, 4.4 Hz, 1H), 3.49 (dd, *J* = 11.0, 4.1 Hz, 1H), 2.98 – 2.87 (m, 2H), 2.80 – 2.65 (m, 2H), 1.92 – 1.65 (m, 2H) ppm; **^{13}C NMR (100 MHz, Chloroform-*d*)**: δ 158.2 , 142.0, 131.7, 130.2, 128.6, 128.5, 126.1, 114.0, 47.9, 41.6, 36.9, 33.2, 33.1 ppm. **HRMS (ESI, m/z)**: calculated for $[M+H]^+$: 289.1359, found: 289.1367.

(1-chloropropane-1,3-diyl)dibenzene (101)

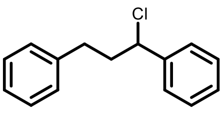

Following the General Procedure F, **3sD** was obtained in 91% yield as a colorless oil; **^{1}H NMR (400 MHz, Chloroform-*d*)**: δ 7.56 – 6.97 (m, 10H), 4.87 (dd, *J* = 8.6, 5.9 Hz, 1H), 2.95 – 2.68 (m, 2H), 2.56 – 2.45 (m, 1H), 2.44 – 2.32 (m, 1H) ppm; **^{13}C NMR (100 MHz, Chloroform-*d*)**:

δ 141.8, 140.8, 128.8, 128.7, 128.5, 127.2, 126.3, 63.0, 41.5, 33.3 ppm. **HRMS (ESI, m/z)**: calculated for $[M+H]^+$: 231.0941, found: 231.0944. **IR (KBr)**: 3208, 1496, 1454, 761, 747, 697 cm^{-1}.

1-(3-chlorobutyl)-4-methoxybenzene (106) & 1-(3-chloro-2-methylpropyl)-4-methoxybenzene (107)

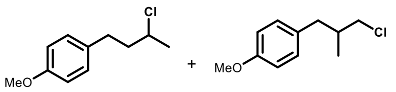

106 **107**

Following the General Procedure F, a mixture of **106** and **107** (2.3:1) was obtained in 95% yield as a colorless oil; **106**: **^{1}H NMR (400 MHz, Chloroform-*d*)**: δ 7.18 – 7.07 (m, 2H), 6.91 – 6.81 (m, 2H), 4.03 – 3.94 (m, 1H), 3.80 (s, 3H), 2.84 – 2.76 (m, 1H), 2.74 – 2.65 (m, 1H), 2.12 – 1.91 (m, 2H), 1.53 (d, *J* = 6.6 Hz, 3H) ppm; **^{13}C NMR (100 MHz, Chloroform-*d*)**: δ 158.0, 133.2, 129.5, 113.9, 58.1, 55.4, 42.3, 32.0, 25.6 ppm; **107**: **^{1}H NMR (400 MHz, Chloroform-*d*)**: δ 7.18 – 7.07 (m, 2H), 6.91 – 6.81 (m, 2H), 3.80 (s, 3H), 3.49 – 3.36 (m, 2H), 2.74 – 2.66 (m, 1H), 2.49 (dd, *J* = 13.7, 7.1 Hz, 1H), 2.13 – 1.91 (m, 1H), 1.01 (d, *J* = 6.7 Hz, 3H) ppm; **^{13}C NMR (100 MHz, Chloroform-*d*)**: δ 158.1, 131.9, 130.2, 113.8, 55.4, 50.5, 39.2, 37.7, 17.8 ppm. **HRMS (ESI, m/z)**: calculated for $[M+H]^+$: 199.0890, found: 199.0896. **IR (KBr)**: 1513, 1247, 1178, 1038, 837 cm^{-1}.

1-methoxy-4-(3-methylbut-2-en-1-yl)benzene (109) & 1-methoxy-4-(3-methylbut-3-en-1-yl)benzene (110)

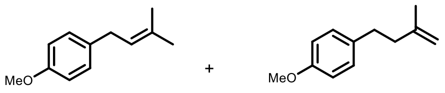

109 **110**

Following the General Procedure F, a mixture of **109** and **110** (2:1) was obtained in 73% yield as a colorless oil; **3aE**: **^{1}H NMR (400 MHz, Chloroform-*d*)**: δ 7.12 – 7.07 (m, 2H), 6.87 – 6.81 (m, 2H), 5.37 – 5.25 (m, 1H), 3.79 (s, 3H), 3.29 (d, *J* = 7.3 Hz, 2H), 1.75 – 1.71 (m, 6H) ppm; **^{13}C NMR (100 MHz, Chloroform-*d*)**: δ 157.8, 134.0, 132.3, 129.3, 123.7, 113.9, 55.4, 33.5, 25.9, 17.9 ppm; **3aE'**: **^{1}H NMR (400 MHz, Chloroform-*d*)**: δ 7.15 – 7.07 (m, 2H), 6.87 – 6.81 (m, 2H), 4.76 – 4.69 (m, 2H), 3.79 (s, 3H), 2.74 – 2.67 (m, 2H), 2.42 – 2.13 (m, 2H), 1.76 (s, 3H) ppm; **^{13}C NMR (100 MHz, Chloroform-*d*)**: δ 157.8, 145.6, 134.4, 129.3, 113.8, 110.2, 55.3, 40.0, 33.4, 22.7 ppm. **HRMS (ESI, m/z)**: calculated for $[M+H]^+$: 177.1279, found: 177.1278. **IR (KBr)**: 2934, 1699, 1600, 1512, 1247, 1161, 1034, 832 cm^{-1}.

1-(3-fluoro-3-methylbutyl)-4-methoxybenzene (102)

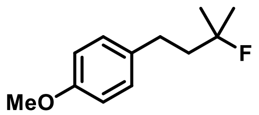

Following the General Procedure H, **102** was obtained in 75% yield as a colorless oil; **^{1}H NMR (400 MHz, Chloroform-*d*)**: δ 7.16 – 7.09 (m, 2H), 6.88 – 6.81 (m, 2H), 3.80 (s, 3H), 2.72 – 2.62 (m, 2H), 1.98 – 1.82 (m, 2H), 1.44 (s, 3H), 1.39 (s, 3H)ppm; **^{13}C NMR (100 MHz, Chloroform-*d*)**: δ 157.8, 134.0, 129.1, 113.8, 95.3 (d, *J* = 165.4 Hz), 55.2, 43.6 (d, *J* = 22.8 Hz), 29.3 (d, *J* = 5.4 Hz), 26.7 (d, *J* = 24.8 Hz) ppm. **^{19}F NMR (376 MHz, Chloroform-*d*)**: δ -138.49 ppm. **HRMS (ESI, m/z)**: calculated for $[M+Na]^+$: 219.1156, found: 219.1141. **IR (KBr)**: 2980, 1513, 1246, 1178, 1037, 822 cm^{-1}.

1-(2-(1-fluorocyclobutyl)ethyl)-4-methoxybenzene (103)

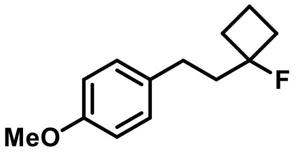

Following the General Procedure H, **103** was obtained in 71% yield as a colorless oil; **^{1}H NMR (400 MHz, Chloroform-*d*)**: δ 7.18 – 7.11 (m, 2H), 6.88 – 6.82 (m, 2H), 3.80 (s, 3H), 2.71 – 2.63 (m, 2H), 2.41 – 2.24 (m, 2H), 2.17 – 2.06 (m, 2H), 2.06 – 1.93 (m, 2H), 1.91 – 1.78 (m, 1H), 1.55 – 1.45 (m, 1H)ppm; **^{13}C NMR (100 MHz, Chloroform-*d*)**: δ 157.8, 134.0, 129.2, 113.8, 96.8 (d, *J* = 211.6 Hz), 55.2, 39.4 (d, *J* = 22.8 Hz), 33.5 (d, *J* = 21.7 Hz), 28.5 (d, *J* = 3.7 Hz), 11.7 (d, *J* = 13.5 Hz) ppm. **^{19}F NMR (376 MHz, Chloroform-d)**: δ -131.66 ppm. **HRMS (ESI, m/z)**: calculated for $[M+Na]^+$: 231.1156, found: 231.1168. **IR (KBr)**: 2938, 1513, 1246, 1177, 1037 cm^{-1}.

(R)-2-(3-ethoxy-3-phenylpropyl)benzo[d]thiazole ((R)-116)

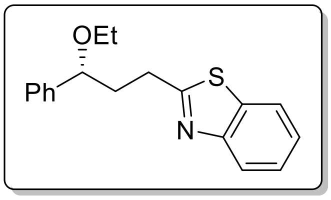

Following the General Procedure C, **116** was obtained in 80% yield as a colorless oil; **^{1}H NMR (400 MHz, Chloroform-*d*)**: δ 7.95 (d, *J* = 8.0 Hz, 1H), 7.83 (d, *J* = 8.0 Hz, 1H), 7.49 – 7.39 (m, 1H), 7.38 – 7.25 (m, 6H), 4.32 (dd, *J* = 8.3, 4.9 Hz, 1H), 3.45 – 3.27 (m, 2H), 3.28 – 3.14 (m, 2H), 2.38 – 2.16 (m, 2H), 1.18 (t, *J* = 7.0 Hz, 3H) ppm; **^{13}C NMR (100 MHz, Chloroform-*d*)**: δ 171.8, 153.4, 142.4, 135.2, 128.6, 127.7, 126.6, 126.0, 124.8, 122.6, 121.6, 80.8, 64.4, 37.9, 30.9, 15.4 ppm. **HRMS (ESI, m/z)**: calculated for $[M+H]^+$: 298.1266, found: 298.1268. **IR (KBr)**: 2971, 1519, 1454, 1437, 1098, 759, 701 cm^{-1}.

(S)-2-chloro-4-(4-methoxyphenyl)butan-1-ol (118) and (S)-3-chloro-2-(4-methoxybenzyl)propan-1-ol (119)

Following the General Procedure F, **118** and **119** were obtained as an inseparable mixture in 75% combined yield as a colorless oil; **^{1}H NMR (400 MHz, Chloroform-*d*)**: δ 7.17 – 7.09 (m, 2H), 6.89 – 6.81 (m, 2H), 4.01 – 3.92 (m, 0.32H), 3.82 – 3.77 (m, 3H), 3.77 – 3.63 (m, 2.65H), 3.54 (dd, *J* = 11.0, 5.3 Hz, 0.68H), 2.89 – 2.79 (m, 0.32H), 2.75 – 2.67 (m, 0.31H), 2.66 (d, *J* = 7.4 Hz, 1.31H), 2.19 – 2.09 (m, 0.68H), 2.06 – 1.97 (m, 0.90H)ppm; **^{13}C NMR (100 MHz, Chloroform-*d*)**: δ 158.2, 158.1, 131.1, 130.1, 129.5, 114.0, 67.1, 64.4, 62.6, 55.4, 45.4, 44.7, 36.1, 33.8, 31.5ppm. **HRMS (ESI, m/z)**: calculated for $[M+H]^+$: 215.0833, found: 215.0844. **IR (KBr)**: 1513, 1247, 1178, 1035 cm^{-1}.

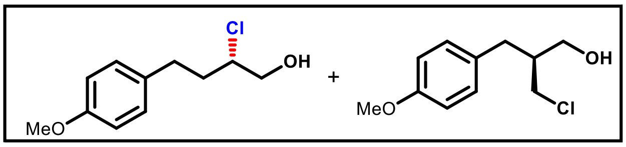

HPLC Spectra

Supplementary Figure 267. HPLC chromatography of racemic **115**

(Chiralpak ADH 250*4.6 mm/5 μm column, isopropanol / hexane = 10 : 90, flow rate = 0.8 mL/min, λ = 254 nm, retention time: t_1 = 6.83 min, t_2 = 9.94 min)

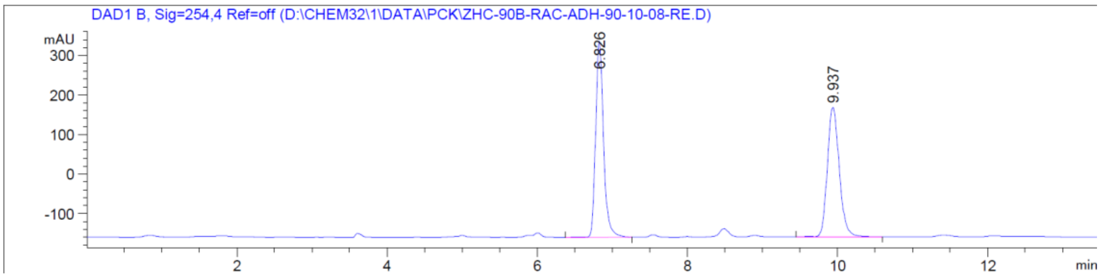

Peak #	RetTime [min]	Type	Width [min]	Area [mAU*s]	Height [mAU]	Area %
1	6.826	VB R	0.1110	3621.02295	496.77594	50.0921
2	9.937	VB R	0.1705	3607.71411	327.61078	49.9079
Totals :				7228.73706	824.38672	

Supplementary Figure 268. HPLC chromatography of chiral **115**: (1R,2R)-**115**

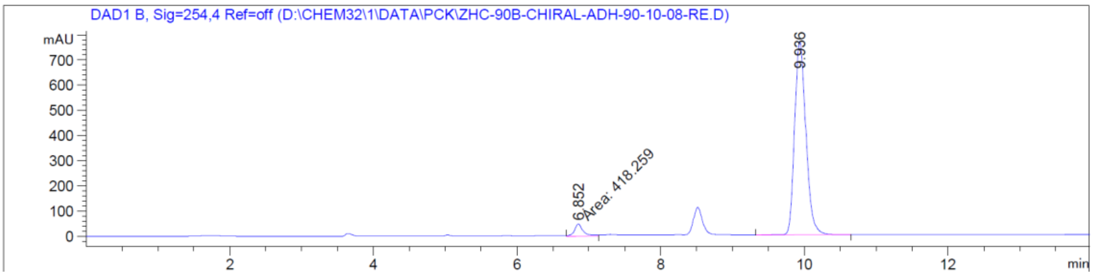

Peak #	RetTime [min]	Type	Width [min]	Area [mAU*s]	Height [mAU]	Area %
1	6.852	MM	0.1426	418.25879	48.88787	4.7227
2	9.936	BB	0.1698	8438.17969	770.15161	95.2773
Totals :				8856.43848	819.03949	

Supplementary Figure 269. HPLC chromatography of racemic **116**

(Chiralpak ODH 250*4.6 mm/5 μm column, isopropanol / hexane = 10 : 90, flow rate = 0.8 mL/min, λ = 250 nm, retention time: t_1 = 7.18 min, t_2 = 7.96 min)

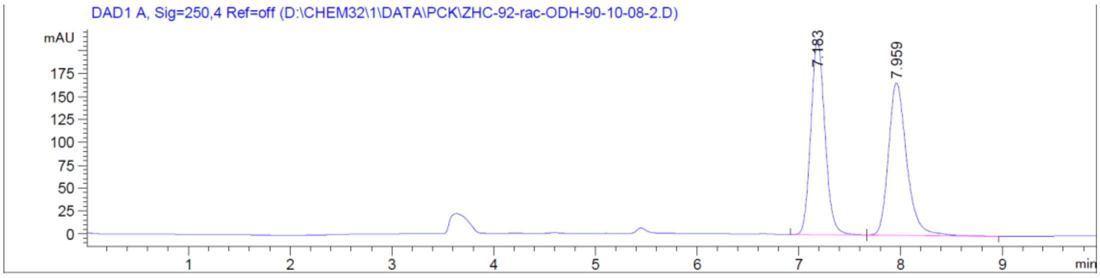

Peak #	RetTime [min]	Type	Width [min]	Area [mAU*s]	Height [mAU]	Area %
1	7.183	BV	0.1487	2059.79346	213.29445	49.6688
2	7.959	VB	0.1901	2087.26123	166.63515	50.3312
Totals :				4147.05469	379.92960	

Supplementary Figure 270. HPLC chromatography of (R)-**116**:

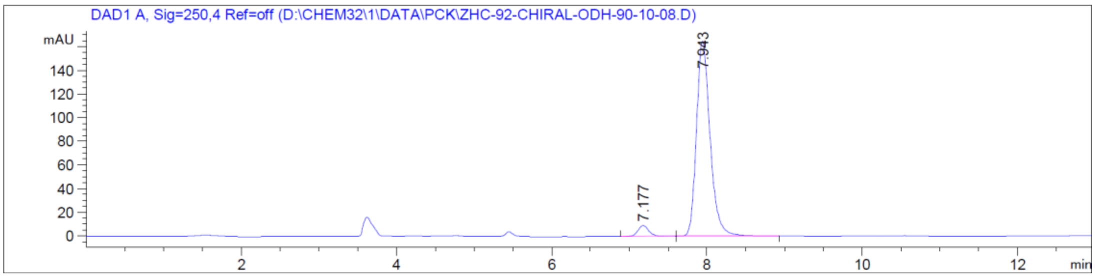

Peak #	RetTime [min]	Type	Width [min]	Area [mAU*s]	Height [mAU]	Area %
1	7.177	BB	0.1468	88.12836	9.11874	4.1959
2	7.943	BB	0.1842	2012.20032	165.04211	95.8041
Totals :				2100.32867	174.16085	

Supplementary Figure 271. HPLC chromatography of racemic **117**
(Chiralpak ODH 250*4.6 mm/5 μm column, isopropanol / hexane = 5 : 95, flow rate = 0.8 mL/min, λ = 230 nm, retention time: t_1 = 14.83 min, t_2 = 16.40 min)

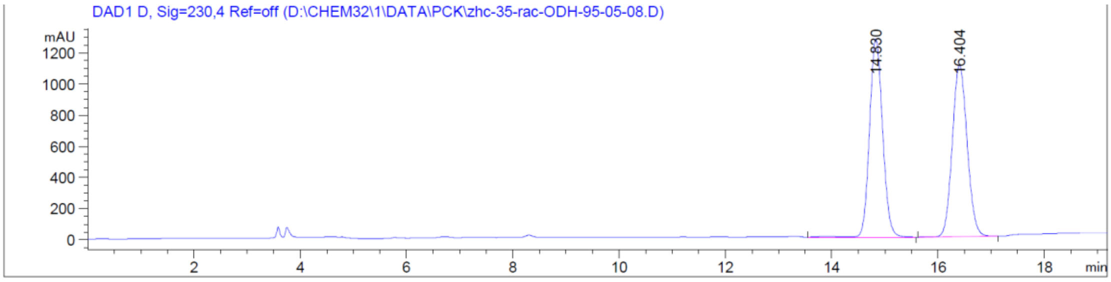

Peak #	RetTime [min]	Type	Width [min]	Area [mAU*s]	Height [mAU]	Area %
1	14.830	VB R	0.2640	2.18489e4	1274.89148	50.5501
2	16.404	BB	0.3021	2.13734e4	1102.91174	49.4499
Totals :				4.32224e4	2377.80322	

Supplementary Figure 272. HPLC chromatography of (1S, 2S)-**117**

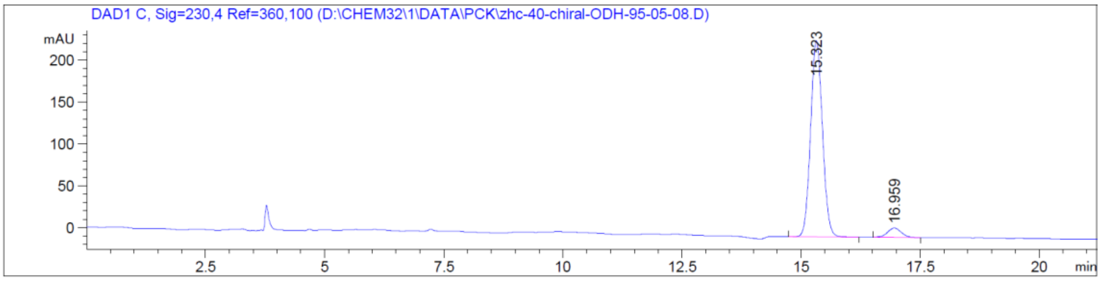

Peak #	RetTime [min]	Type	Width [min]	Area [mAU*s]	Height [mAU]	Area %
1	15.323	BB	0.2704	4064.15454	234.28584	94.9116
2	16.959	BB	0.3055	217.88588	11.07388	5.0884
Totals :				4282.04042	245.35972	

Supplementary Figure 273. HPLC chromatography of racemic **118**

(Chiralpak OBH 250*4.6 mm/5 μm column, isopropanol / hexane = 10 : 90, flow rate = 1.0 mL/min, λ = 220 nm, retention time: t_1 = 10.69 min, t_2 = 11.58 min)

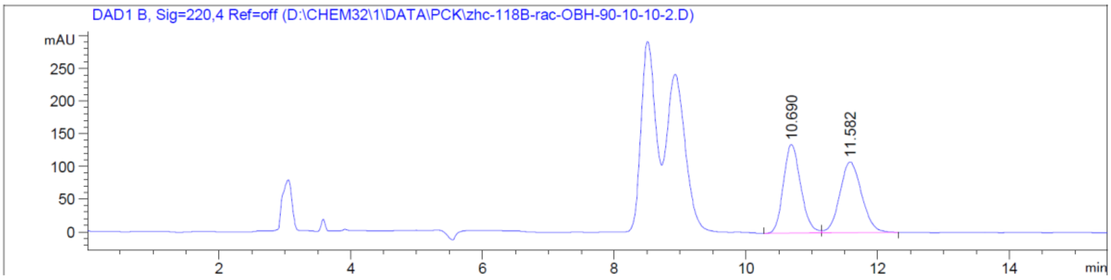

Peak #	RetTime [min]	Type	Width [min]	Area [mAU*s]	Height [mAU]	Area %
1	10.690	BV	0.2839	2484.63379	135.51595	49.9012
2	11.582	VB	0.3596	2494.47070	107.96228	50.0988

Totals : 4979.10449 243.47823

Supplementary Figure 274. HPLC chromatography of (S)-**118**

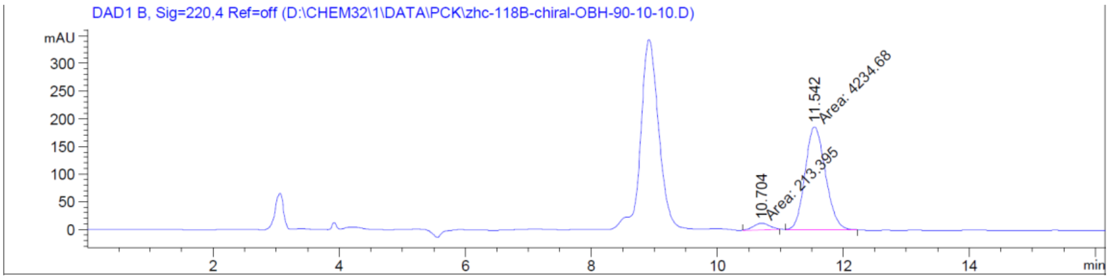

Peak #	RetTime [min]	Type	Width [min]	Area [mAU*s]	Height [mAU]	Area %
1	10.704	MM	0.2978	213.39507	11.94192	4.7975
2	11.542	MM	0.3781	4234.68018	186.68420	95.2025

Totals : 4448.07524 198.62613

DFT Study

The DFT calculations were performed with the Gaussian 09 program[13]. Geometries of the minimum energy structures were optimized at the B3-LYP level of theory with the 6-31G(d, p) basis. After optimizing the structure, we obtained its wavefuction, and the electronic structure analysis was performed with the Multiwfn (Multifunctional Wavefunction Analyzer)[14] including orbital composition analysis with Mulliken partition[15]. In this way, the coefficients on each carbon atom were not simply squared and summed up. Here, the Mulliken partition for calculating orbital components corresponded to the method used by Mulliken population method. In Mulliken population method, the population of a basis function of an orbit was the contribution value of the basis function multiplied by the orbital occupancy.

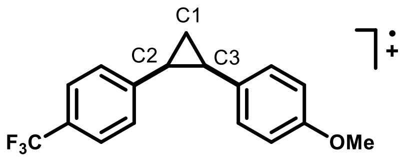

120-Int

Calculated Mulliken charge distribution of cation radical of 120

Atom	value
C1	-0.227
C2	-0.141
C3	-0.145

Orbital	76 is alpha-HOMO, energy:	-0.330799 a.u.	-9.001510 eV
Orbital	465 is beta-HOMO, energy:	-0.384118 a.u.	-10.452385 eV
Orbital	77 is alpha-LUMO, energy:	-0.229432 a.u.	-6.243161 eV
Orbital	466 is beta-LUMO, energy:	-0.272294 a.u.	-7.409487 eV

Orbital 77 is alpha-LUMO

Composition of each atom:

Atom	1(C) :	0.86662%
Atom	2(C) :	18.20249%
Atom	3(C) :	26.94939%

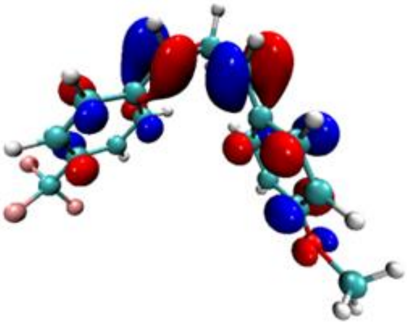

alpha-LUMO
120-Int

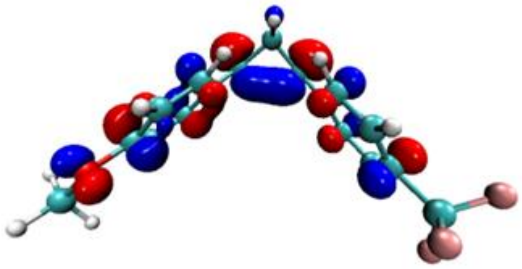

beta LUMO
120-Int

1 2

C	0.88349300	3.22317700	-0.78659600
C	-0.18654800	2.87574400	0.19495200
C	1.89944800	2.58250900	0.09925500
H	0.77361000	2.76178600	-1.76624400
H	1.03193700	4.30027900	-0.89423500
H	-0.23856900	3.50572100	1.07907500
H	2.19946500	3.17200500	0.96215100
C	2.48371400	1.31180200	-0.02555000
C	2.29897000	0.45171000	-1.15498700
C	3.35582600	0.85858500	1.01319000
C	2.93210200	-0.76086400	-1.22957700
H	1.66950100	0.76625000	-1.97866500
C	3.99909200	-0.35593700	0.94682600
H	3.51421400	1.49648300	1.87777900
C	3.79112100	-1.18739000	-0.18037000
H	2.80942900	-1.41700200	-2.08389400
H	4.65512000	-0.66556700	1.75030700
C	-1.11603400	1.80191300	0.12206800
C	-1.94611600	1.54896900	1.24942200
C	-1.28903100	0.99149100	-1.03236000

C	-2.88951600	0.53619300	1.22752500
H	-1.83230000	2.16118700	2.13896600
C	-2.23470000	-0.01897600	-1.04960900
H	-0.70171100	1.17719400	-1.92394000
C	-3.03381300	-0.25294400	0.07912600
H	-3.51220200	0.34559700	2.09395800
H	-2.36454100	-0.63265800	-1.93381800
O	4.35346000	-2.37610100	-0.35792500
C	5.25702300	-2.91310800	0.62677500
H	5.56677800	-3.87944400	0.23379400
H	6.12820900	-2.26253200	0.74388600
H	4.74668900	-3.04738400	1.58466500
C	-4.09910300	-1.32665500	0.02834900
F	-5.24320600	-0.83467200	-0.48317200
F	-3.71160200	-2.35573400	-0.74987700
F	-4.36840200	-1.80630800	1.25626900

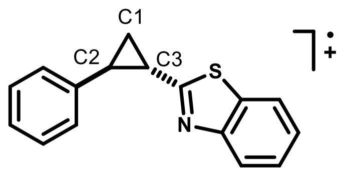

115-Int

Orbital 66 is alpha-HOMO, energy: -0.352777 a.u. -9.599542 eV

Orbital 404 is beta-HOMO, energy: -0.366150 a.u. -9.963438 eV

Orbital 67 is alpha-LUMO, energy: -0.200533 a.u. -5.456776 eV

Orbital 405 is beta-LUMO, energy: -0.302214 a.u. -8.223653 eV

HOMO-LUMO gap of alpha orbitals: 0.152244 a.u. 4.142766 eV

HOMO-LUMO gap of beta orbitals: 0.063936 a.u. 1.739785 eV

Orbital 67 is alpha-LUMO

Composition of each atom:

Atom	1(C) :	2.27929%
Atom	2(C) :	14.08735%
Atom	3(C) :	5.40518%

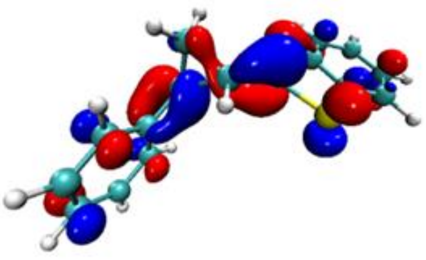

alpha-LUMO
115-Int

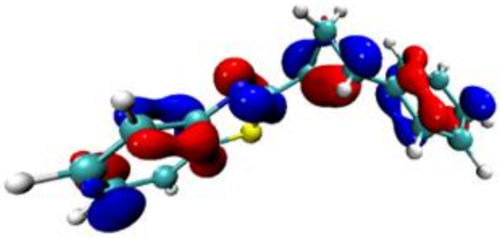

beta LUMO
115-Int

1 2

C	1.22592900	1.97562500	-0.88532600
C	1.67302400	1.08851900	0.21110400
C	0.61962100	0.62584400	-1.12372000
H	0.51818100	2.74779200	-0.59887700
H	1.96436300	2.28626300	-1.61875300
H	1.12994900	-0.05461400	-1.79530400
C	2.92928000	0.37570600	0.29522900
C	3.21236000	-0.34498700	1.48319900
C	3.89150100	0.38047900	-0.74609700
C	4.41405000	-1.02051100	1.63002200
H	2.47945100	-0.35530800	2.28457400
C	5.08748500	-0.30166400	-0.59525200
H	3.70495900	0.93017900	-1.66310200
C	5.35363500	-1.00367300	0.59108800
H	4.62549900	-1.56125700	2.54626500
H	5.82365000	-0.28709900	-1.39191200
H	1.06957900	1.12109700	1.11246100
H	6.29375100	-1.53417700	0.70362600
C	-0.70942400	0.30705200	-0.72190600

N	-1.44470100	1.09362800	0.05325100
S	-1.48868500	-1.21087900	-1.22443900
C	-2.66569200	0.55826000	0.28041300
C	-2.90787900	-0.71858900	-0.33925500
C	-3.68427200	1.16655600	1.05838800
C	-4.12887500	-1.37012600	-0.19528000
C	-4.88776000	0.51129000	1.19670300
H	-3.49276800	2.12897100	1.51914800
C	-5.10861300	-0.74535800	0.57604900
H	-4.31937700	-2.33016300	-0.66152600
H	-5.68323300	0.95587700	1.78469300
H	-6.06835200	-1.23543100	0.70480400

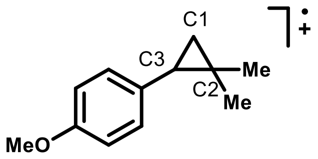

1-Int

Calculated Mulliken charge distribution of cation radical of 1

Atom	value
C1	-0.19562
C2	0.07557
C3	-0.21318

Orbital 48 is alpha-HOMO, energy: -0.360945 a.u. -9.821823 eV

Orbital 322 is beta-HOMO, energy: -0.400915 a.u. -10.909447 eV

Orbital 49 is alpha-LUMO, energy: -0.174612 a.u. -4.751435 eV

Orbital 323 is beta-LUMO, energy: -0.298670 a.u. -8.127212 eV

Orbital 49 is alpha-LUMO

Composition of each atom:

Atom 1(C) : 1.75852%

Atom	2(C) :	10.84285%
Atom	3(C) :	2.49653%

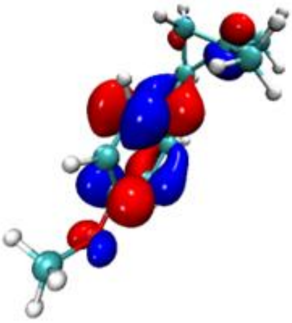

alpha-LUMO
1-Int

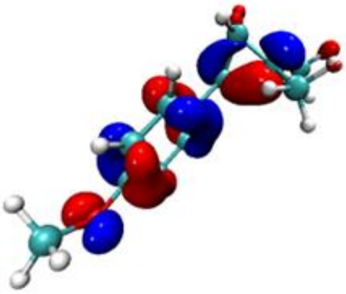

beta LUMO
1-Int

1 2

C	2.70396500	0.49590100	-1.24846500
C	2.95954600	0.31755100	0.19520300
C	1.77084100	-0.56244100	-0.70837200
H	2.25016600	1.43217500	-1.56221600
H	3.45485100	0.12198200	-1.94166000
H	2.12289100	-1.58648100	-0.77352000
C	0.37072600	-0.42153500	-0.44679500
C	-0.35765500	-1.57097900	0.00019600
C	-0.35848400	0.79290800	-0.62707100
C	-1.70051400	-1.51129600	0.25748900
H	0.17438500	-2.50713500	0.13752100
C	-1.70925900	0.86812600	-0.37639100
H	0.14888600	1.67517400	-0.99999800
C	-2.40300200	-0.28622200	0.07655100
H	-2.25891600	-2.37618100	0.59759500
H	-2.23743700	1.79916900	-0.53955300
O	-3.69730800	-0.32918200	0.34890800
C	-4.52944700	0.84085500	0.20213500

H	-5.52694200	0.51788300	0.49254000
H	-4.18590000	1.63848700	0.86604900
H	-4.53308200	1.17692500	-0.83810500
C	4.08599100	-0.57478200	0.64848700
H	4.96674600	0.05205600	0.84228800
H	3.84474500	-1.08955500	1.58332400
H	4.36691100	-1.31395200	-0.10514300
C	2.47616000	1.32805900	1.20096300
H	1.62629900	1.91510800	0.85124100
H	2.21205600	0.85607100	2.15187800
H	3.29959200	2.02486200	1.40291600

Supplementary References

1. Guo, W., Ding, H., Gu, C., Liu, Y., Jiang, X., Su, B. & Shao, Y. Potential-resolved multicolor electrochemiluminescence for multiplex immunoassay in a single sample. *J. Am. Chem. Soc.* **140**, 15904–15915 (2018).
2. White, A. R., Wang, L. & Nicewicz, D. A. Synthesis and characterization of acridinium dyes for photoredox catalysis. *Synlett* **30**, 827–832 (2019).
3. Huang, H., Li, X.-M., Yu, C.-G., Zhang, Y.-T., Mariano, P.S. & Wang, W. Visible-light-promoted nickel- and organic-dye-cocatalyzed formylation reaction of aryl halides and triflates and vinyl bromides with diethoxyacetic acid as a formyl wquivalent. *Angew. Chem. Int. Ed.* **56**, 1500–1505 (2017).
4. Gini, A., Uygur, M., Rigotti, T., Alemán, J. & Mancheño, O. G. Novel oxidative Ugi reaction for the synthesis of highly active, visible-light, imide-acridinium organophotocatalysts. *Chem. Eur. J.* **24**, 12509–12514 (2018).
5. White, A. R., Wang, L.-F. & Nicewicz, D. A. Synthesis and characterization of acridinium dyes for photoredox catalysis. *Synlett.* **30**, 827–832 (2019).
6. Ge, L., Wang D.-X., Xing, R., Ma, D., Walsh, P.-J. & Feng, C. Photoredox-catalyzed oxo-amination of aryl cyclopropanes. *Nat. Commun.* **10**, 4367 (2019).
7. Charette, A. B. & Juteau, H. Design of amphoteric bifunctional ligands: application to the enantioselective Simmons-Smith cyclopropanation of allylic alcohols. *J. Am. Chem. Soc.* **116**, 2651-2652 (1994).
8. Postils, V., Rodríguez, M., Sabenya, G., Conde, A., Díaz-Requejo, M. M., Pérez, P. J., Costas, M., Solà, M. & Luis, J. M. Mechanism of the selective Fe-catalyzed arene carbon–hydrogen bond functionalization. *ACS Catal.* **8**, 4313–4322 (2018).

9. Sugawara, M. & Yoshida, J.-I. Remarkable γ-effect of tin: acid-promoted cyclopropanation reactions of α-((alkoxycarbonyl)oxy)stannanes with alkenes. *J. Am. Chem. Soc.* **119**, 11986–11987 (1997).

10. Allegretti, P. A. & Ferreira, E. M. Generation of α,β-unsaturated platinum carbenes from homopropargylic alcohols: rearrangements to polysubstituted furans. *Org. Lett.* **13**, 5924–5927 (2011).

11. Onodera, S., Ishikawa, S., Kochi, T. & Kakiuchi, F. Direct alkenylation of allylbenzenes via chelation-assisted C–C Bond Cleavage. *J. Am. Chem. Soc.* **140**, 9788–9792 (2018).

12. Alam, M., Wise, C., Baxter, C. A., Cleator, E. & Walkinshaw, A. Development of a robust procedure for the copper-catalyzed ring-opening of epoxides with Grignard reagents. *Org. Process Res. Dev.* **16**, 435–441 (2012).

13. Frischi, M. J.; Trucks, G. W.; Schlegel, H. B.; Scuseria, G. E.; Robb, M. A.; Cheeseman, J. R.; Scalmani, G.; Barone, V.; Mennucii, B.; Petersson, G. A.; Nakatsuji, H.; Caricato, M.; Li, X.; Hratchian, H. P.; Izmaylov, A. F.; Bloino, J.; Zheng, G.; Sonnenberg J. L.; Hada, M.; Ehara, M.; Toyota, K.; Fukuda, R.; Hasegawa, J.; Ishida, M.; Nakajima, T.; Honda, Y.; Kitao, O.; Nakai, H.; Vreven, T.; Montgomery, J. A.; Jr., Peralta, J. E.; Ogliaro, F.; Bearpark, M.; Heyd, J. J.; Brothers, E.; Kudin, K. N.; Staroverov, V. N.; Keith, T.; Kobayashi, R.; Normand, J.; Raghavachari, K.; Rendell, A.; Burant, J. C.; Iyengar, S. S.; Tomasi, J.; Cossi, M.; Rega, N.; Millam, J. M.; Klene, M.; Knox, J. E.; Cross, J. B.; Bakken, V.; Adamo, C.; Jaramillo, J.; Gomperts, R.; Stratmann, R. E.; Yazyev, O.; Austin, A. J.; Cammi, R.; Pomelli, C.; Ochterski, J. W.; Martin, R. L.; Morokuma, K.; Zakrzewski, V. G.; Voth, G. A.; Salvador, P.; Dannenberg, J. J.; Dapprich, S.; Daniels, A. D.; Farkas, O.; Foresman, J. B.; Ortiz, J. V.; Cioslowski, J.; Fox, D. J.; Gaussian 09, Revision D.01;

14. Lu T. & Chen, F. Multiwfn: A multifunctional wavefunction analyzer. *J. Comput. Chem.* **33**, 580–592 (2012).

15. T. Lu and F. Calculation of Molecular Orbital Composition. *Acta Chim. Sinica* **69**, 2393–2406 (2011).
